# Microbial Profiling of Amniotic Fluid, Umbilical Blood and Placenta of the Foaling Mare

**DOI:** 10.3390/ani13122029

**Published:** 2023-06-18

**Authors:** Elisabeth Hemberg, Adnan Niazi, Yongzhi Guo, Viktória J. Debnár, Boglarka Vincze, Jane M. Morrell, Gabriella Kútvölgyi

**Affiliations:** 1 Herrgården Hjortkvarn, SE-697 93 Hjortkvarn, Sweden; envab@telia.com; 2 SLU-Global Bioinformatics Centre, Department of Animal Breeding and Genetics, Swedish University of Agricultural Sciences (SLU), SE-750 07 Uppsala, Sweden; adnan.niazi@slu.se; 3 National Bioinformatics Infrastructure Sweden (NBIS), Science for Life Laboratory, Uppsala University, SE-752 36 Uppsala, Sweden; 4 Department of Clinical Sciences, Faculty of Veterinary Medicine and Animal Science, Swedish University of Agricultural Sciences (SLU), SE-750 07 Uppsala, Sweden; yongzhi.guo@slu.se; 5 Department of Precision Livestock Farming and Animal Biotechnics, Institute of Animal Sciences, Kaposvár Campus, Hungarian University of Agriculture and Life Sciences, H-2100 Gödöllő, Hungary; debnar.viktoria.johanna@uni-mate.hu (V.J.D.); kutvolgyi.gabriella@uni-mate.hu (G.K.); 6 Department of Obstetrics and Food Animal Medicine, University of Veterinary Medicine, H-1078 Budapest, Hungary; vincze.boglarka@univet.hu

**Keywords:** placental microbiome, equine pregnancy, foal health, 16S rRNA sequencing, bacterial isolation, polymorphonucleocytes

## Abstract

**Simple Summary:**

Although previously the placenta was assumed to be sterile, with microbes only being present in association with pathology, recent studies have suggested that this assumption may not be correct. Some researchers argue for the presence of a microbial community in the placenta, which is important to help the foal to adapt to life outside the uterus. Therefore, we examined the placenta, amniotic fluid and umbilical blood of 24 foaling mares, as well as jugular blood from the foals. All of the mares and foals were healthy, and foaling was normal. Some bacterial growth was isolated in most of the umbilical blood samples. Bacterial DNA was extracted and sequenced from placental samples. The most abundant phyla were Proteobacteria (approximately 44%) and Actinobacteria (approximately 28%). In conclusion, bacteria were found in the fetal compartments and placenta of healthy equine pregnancies, perhaps lending support to the theory that the placenta has its own bacterial community.

**Abstract:**

The presence of a microbiome/microbiota in the placenta is hotly debated. In previous studies, the presence of bacteria in equine amniotic fluid and umbilical blood was independent of foal health. The objective of the present study was to determine if the same bacteria are present in the equine placenta as in amniotic fluid and umbilical blood. Samples were obtained from 24 parturient mares and foals. Placental bacterial DNA was extracted, and the microbiome was identified using 16S rRNA sequencing. All amniotic fluid samples contained some polymorphonucleocytes; bacteria were isolated from four samples. Aerobic or anaerobic growth was found in 18 and 3 umbilical blood samples, respectively. Serum amyloid A was <5 mg/L in all 24 samples, total WBC varied between 2900 and 10,700/µL, and fibrinogen varied between 0 and 5.16 g/L. In jugular blood, serum amyloid A was <5 mg/L in all 24 foals, total white blood count was 3200 to 8100/µL, and fibrinogen was 0.44 to 4.42 g/L. The diversity of bacterial microbiota was similar in all placental regions at the phylum level but differed at the genus level; the most abundant phyla were Proteobacteria (42–46.26%) and Actinobacteria (26.91–29.96%). In conclusion, bacteria were found in the fetal compartments and placenta of healthy equine pregnancies; however, we can neither prove nor disprove the hypothesis that the placenta has its own microbiome.

## 1. Introduction

The loss of a pregnancy, or the birth of a sickly foal requiring constant attention during neonatal life, represent an economic burden to the equine breeding industry [1,2]. The health of the equine placenta might be implicated in both of these phenomena. In a previous study, it was shown that a significant proportion of mares with endometritis produced compromised foals [3], while in another study, live bacteria were found to be present in the amniotic fluid and umbilical blood of healthy newborn foals [4].

The presence of a microbiome (bacterial DNA) or microbiota (a viable microbial community) in human fetal compartments such as amniotic fluid, umbilical blood and the placenta is still controversial, despite an abundance of publications on the presence of a microbiome in both healthy and nonhealthy pregnancies. However, the methodology varies considerably among studies, making drawing a meaningful conclusion difficult. In animals, only a few studies have investigated microbiota/microbiomes in the fetal compartments, e.g., in equine amniotic fluid [5]. Bacteria have been detected in the meconium passed by the foal, suggesting that colonization started in utero [6]. A recent study [7], in which preterm and postpartum placental samples were subjected to 16S rRNA sequencing, suggested that bacteria may be present in the healthy equine placenta. However, the mechanism by which microbiota is transferred into the pregnant uterus is still unknown.

In a previous study by Hemberg et al. [4], bacteria (mainly coagulase negative staphylococcus) were found in the fetal compartments such as the amniotic fluid and umbilical blood, regardless of the health status of the foal. In studies on the placenta of other species, such as humans, bovines, canines and murines, bacteria have been identified in healthy individuals as normal microbiota/microbiomes, although some medical researchers consider the bacteria to be contaminants [8,9,10,11]. Our hypothesis is that the equine placenta does have its own microbiome. The aims of the present study were (i) to type the bacteria in the amniotic fluid and the umbilical blood; and (ii) to determine if the same bacteria, or additional bacteria, could be detected in three different locations of the equine placenta using 16S rRNA sequencing.

## 2. Materials and Methods

### 2.1. Animals

Samples from 24 foaling mares from a Lipizzaner stud in Hungary were collected during the foaling season in 2017. The mares were 6–21 years old; all reproductive groups were represented (maiden, foaled and mares that had not foaled in previous years). Parturition was spontaneous in all cases. The mares were foaling among other mares (60–70 mares and foals were loose-housed overnight in a large barn). After parturition, the mare and newborn foal were taken into a separate box by the groom. Length of pregnancy, time to stand and nurse, postpartum complications, health status of the foal and the time between foaling and the expulsion of the placenta were recorded.

### 2.2. Sampling

**Figure 1:**
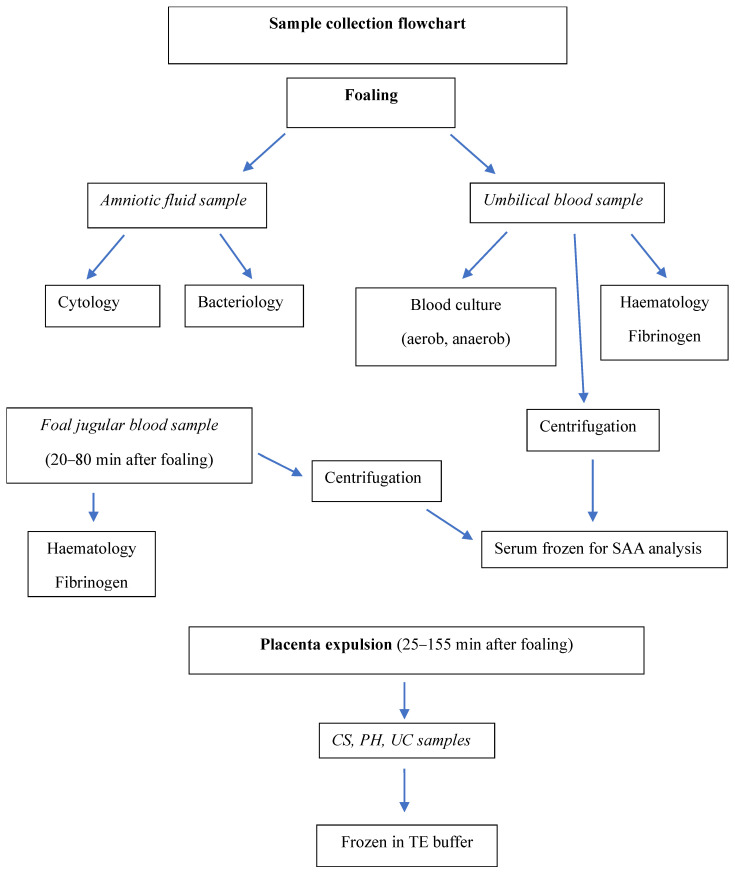
Flow chart depicting the sampling protocol (*n* = 24).

The sampling protocol is shown in Figure 1.

#### 2.2.1. Amniotic Fluid

The amniotic fluid was collected when the amniotic sac was clearly visible; it was swabbed with 70% ethanol three times at the lowest point. The sample was drawn into a 20 mL syringe using a sterile 18-gauge needle. Two aliquots of amniotic fluid (8–10 mL each) were placed in plastic tubes for bacteriological culture and cytological examination. Samples were refrigerated until they were transported to the laboratory.

#### 2.2.2. Umbilical Vein Blood

After the birth of the foal, the umbilicus over the umbilical vein was washed three times with 70% ethanol more than 10 cm from the natural breaking point, and 20 mL blood was aspirated using a sterile syringe and 18 G needle. A small hematoma was seen within the umbilical tissue after the puncture. One blood sample was collected into a tube containing ethylene diaminetetraacetic acid (EDTA) for hematology examination, and one into a tube containing sodium citrate for fibrinogen analysis; they were refrigerated until transport. One additional serum tube was filled with blood and centrifuged at 1000× *g* for 15 min within 30 min of taking the sample. The separated serum was pipetted into a plastic cryotube and frozen for subsequent serum amyloid A (SAA) analysis. For aerobic and anaerobic blood culture, 3 mL of blood was added to two glass bottles containing a blood culture medium.

#### 2.2.3. Placenta Sample

The time between foaling and the expulsion of the placenta was recorded. By the time the placenta was expelled, the mare and foal had been moved to a separate clean box. The placenta was laid in an “F” shape and examined for completeness and any pathological findings. No disinfectant was used during the processing of the placenta. Sugar lump-sized samples were collected using sterile scissors from the following areas: (1) The umbilical cord at the attachment to the amniotic sac (UC); (2) cervical star (CS), from two sites opposite each other; (3) pregnant horn, mid-part (PH). The samples were frozen in Tris-EDTA (TE) buffer.

#### 2.2.4. Jugular Blood from the Foal

Blood samples were taken from the foal’s jugular vein 20 min to 80 min after parturition (mean ± SD 40 ± 16 min; *n* = 23). Blood samples were collected into an EDTA tube for hematology examination and a citrate tube for fibrinogen analysis; they were refrigerated until analysis. One additional serum tube was centrifuged at 1000× *g* for 15 min. The separated serum was pipetted into a plastic cryotube, then stored at −20 °C until SAA analysis.

### 2.3. Processing of Samples

#### 2.3.1. Cytospin Preparation of the Amniotic Fluid and Clinical Chemistry

Cytospin preparations and clinical chemistry analyses were performed in the clinical Chemistry laboratory of the University of Veterinary Medicine, Budapest. The samples (umbilical blood, foal blood and amniotic fluid) were transported to the laboratory chilled in an insulated box. Hematology analyses were performed with an Advia 120 Hematology Analyzer (Siemens, Germany) using the equine profile. Fibrinogen was measured with a KC4 coagulation analyzer (Amelung, Germany) using Dia-FIB reagent (Diagon, Hungary).

The total numbers in cytospin preparations, including epithelial cells and leukocytes, were evaluated by microscopy. The same clinical pathologist evaluated all samples.

Cytologic evaluation of the samples was made manually by microscopic examination of cytospin preparations after staining with May–Grunwald–Giemsa. The total number of cells was determined semi-quantitatively by an experienced cytologist and was reported as low, moderate, large or very large. The percentage of the different cell types was calculated. The proportion of PMNLs was reported according to the following categories: 0%, 1–14%, 15–29% and ≥30%.

#### 2.3.2. Bacteriology

All samples for bacteriology (umbilical blood and amniotic fluid) were transported to the Duo-Bakt Veterinary Microbiological Laboratory (Budapest, Hungary) laboratory in an insulated box at room temperature (blood culture bottles) or with a cold pack (amniotic fluid), and were cultured within 24 h of collection.

Amniotic fluid samples were cultured on 5% horse blood agar plates (Bak-Teszt Ltd., Budapest, Hungary), and MacConkey agar (bioMérieux, Marcy l’Etoile, France). Bacterial growth was examined after incubation for 24 and 48 h at 37 °C in 5% CO_2_. Based on the number of colonies on the agar plates, bacterial growths on the plates were classified as no growth, sparse (*n* < 20), moderate (20 to 100) or profuse (>100). Colonies were identified by colony morphology, Gram stain and biochemical tests, according to standard laboratory procedures. The bacteria were identified by latex agglutination (streptococci); by coagulase, DNase, mannitol (staphylococci); and by API-systems (bioMérieux, Marcy l’Etoile, France) (coliform bacteria and other strains) before final identification. The final identification was performed by MALDI-TOF (matrix-assisted laser desorption/ionization–time of flight) mass spectrometry (Bruker Daltonics Microflex, MALDI Biotyper, Bremen, Germany) at an external institute (Semmelweis University, Institute of Laboratory Medicine, Budapest, Hungary).

Umbilical blood was inoculated into aerobic and anaerobic vials of BactAlert Blood Culture System (BioMérieux, Marcy l’Etoile, France), and incubated at 37 °C for seven days. If growth was detected, the bottle was opened, and broth was inoculated on agar plates. Both the aerobic and anaerobic broths were cultured aerobically on horse blood agar plates and MacConkey agar plates, as described previously. The anaerobic broth was also cultured on Wilkins–Chalgren agar plates with 5% defibrinated horse blood (Bak-teszt Ltd., Budapest, Hungary). Colonies of anaerobic strains were identified by their macro- and micromorphology, Gram stain, antimicrobial susceptibility and MALDI-TOF, according to standard laboratory procedures.

#### 2.3.3. Serum Amyloid A (SAA)

The serum concentration of SAA was determined in umbilical blood and jugular blood from the foal using the Vet-SAA Eiken kit (Eiken SAA, Eiken Chemical Co., Ltd., Tokyo, Japan). The analyses were performed on turbidimetric immunoassay (TIA) using latex Agglutination by Cobas 701 (Roche Diagnostics International, Rotkreuz, Switzerland) clinical chemistry analyzer. A reference level of <5 mg/L was used.

#### 2.3.4. DNA Extraction

First, pieces (250 ± 50 mg) of placenta were excised, placed in sterile 15 mL centrifuge tubes and washed twice in sterile DPBS (Gibco 14040-091). The samples were cut into small pieces (approximately 0.25 mm^3^) in a single-use Petri dish on ice using a sterile surgical blade. Then, DNA was extracted using the QIAamp^®^ DNA Mini Kit (Qiagen, Cat No 51304, Redwood, CA, USA) following the manufacturer’s instructions. The quantity and quality of the DNA was tested using NanoDrop 8000 Spectrophotometer (Thermo Scientific, Waltham, MA, USA), and the ratio of absorbance at 260 and 280 nm (A260/280) for all samples was between 1.75–1.90. The purified DNA samples were stored at −80 °C prior to sequencing.

#### 2.3.5. Bacterial 16S rRNA Sequencing

Extracted DNA samples were sent to the National Genomic Infrastructure laboratory at SciLife Lab, Uppsala for library preparation and sequencing. Briefly, the 16S region was amplified with 16S Ion Metagenomics Kit ™ (Thermo Fisher Scientific, Waltham, MA, USA) by two separate PCR reactions using primer set V2, V4, V8 and V3, V6-7, V9. The bacterial 16S content of the sample was estimated by qPCR, and sample volumes were then normalized for the library preparation. Library preparation was performed on the AB Library Builder System according to the manufacturer’s instructions. The final libraries were assessed and quantified with the Fragment Analyzer system, using the DNF-474 High Sensitivity NGS Fragment analysis kit (Advanced Analytical Technologies, Orangeburg, NY, USA). Template preparation and chip loading were performed on the Ion Chef system using the Ion 530 chip; sequencing was performed on the Ion S5™ XL system, following the Ion 520™ & 530™ Kit–Chef user guide (rev D.0).

### 2.4. Data Processing and Taxonomic Classification

Raw data were uploaded to Ion Reporter in BAM format and analyzed using Metagenomics 16S v1.1 workflow under default parameters. Both Curated MicIEQ (R) 16S Reference Library v2013.1 and Curated Greengenes v13.5 databases (Thermo Fisher Scientific) were chosen as references to classify the sequences into operational taxonomic units (OTUs). Mean read lengths ranged between 241 bp and 210 bp for all samples.

To characterize microbial biomass accurately, we applied a stringent approach to remove potentially erroneous and contaminated sequences. Only those OTUs with a minimum count of ≥10 in at least three samples in each group were retained for further analysis. Furthermore, any OTUs with mean relative abundance ≥ 90th percentile in each sample group were removed.

### 2.5. Statistical Analysis

For microbiome data, alpha and beta diversity were calculated using an R package Phyloseq [12] and analyzed with R statistical software (R Core Team, 2020, Vienna, Austria; v 4.0.3).

Alpha diversity significance was determined using Kruskal–Wallis tests with a false discovery rate corrected *p*-value < 0.05. Beta diversity significance, which measures the microbial composition between the samples, was determined using overall and pairwise PERMANOVA tests, with a false discovery rate corrected *p*-value < 0.05 as well.

## 3. Results

### 3.1. Foaling

Twenty-four healthy foals were born, according to Rossdale [12]. Nineteen foals nursed within 2 h; five foals needed more than 2 h to stand and nurse. The length of the gestation of the mares varied between 311 and 341 days (mean ± SD 321 ± 7 days; only one mare foaled at 311 days and one at 341 days). Expulsion of the placenta occurred within 3 h in all the mares. The mean (±SD) time of expulsion was 67 ± 36 min.

### 3.2. Polymorphonuclear Leucocytes (PMNLs) in the Amniotic Fluid

All cytospin preparations contained cells, including mature squamous cells, keratin scales and PMNLs. All amniotic fluid samples contained some PMNLs, with a small-to-average number of PMNLs being observed in most cases (Table 1) and a high number in only five samples. However, the PMNLs were often rolled up and difficult to evaluate, making it impossible to assess if any intracellular bacteria were present, or even, in some cases, to obtain an exact number of PMNLs.

### 3.3. Bacteriology of Amniotic Fluid and Umbilical Blood

The majority of the amniotic fluid samples (18/24 samples) contained no bacteria, whereas a pure culture of either *Acinetobacter lwoffii*, *Streptococcus equi* ssp. *zooepidemicus, Enterococcus faecalis* or coagulase-negative staphylococci (CNS), such as *Staphylococcus xylosus*, was detected in four samples (Table 2). The remaining two samples produced mixed growth, which was considered contamination, and was therefore not subjected to further analysis.

No bacterial growth was observed in 4 umbilical blood samples; aerobic growth was seen in 18 samples and anaerobic growth in 3 samples; 2 aerobic samples, where profuse mixed bacterial growth was observed, even though there was no bacterial growth in anaerobic samples (even with aerobic culture), were regarded as contaminated, and were, therefore excluded. The most common aerobic bacterial growth was CNS such as *Staph. sciuri* in 12 samples and *Staph. lentus* in 1 sample; nonclassified CNS in 1 sample; *Ent. faecalis* in 1 sample; *E. coli* in 2 samples; *Bacillus* sp. in 1 sample. Two anaerobic samples had bacterial growth of *E. coli* and *Bacillus* sp.

### 3.4. White Blood Cell Count, Serum Amyloid A and Fibrinogen in Umbilical Blood and Jugular Blood

The SAA was <5 mg/L in all samples of umbilical and jugular blood. The total white blood cell count (WBC) in umbilical blood varied between 2900 and 10,700/µL, and fibrinogen varied between 0 and 5.16 g/L (Table 2).

In jugular blood, total WBC was 3200 to 8100/µL, and fibrinogen was 0.44 to 4.42 g/L (Table 2). For foals with a low total WBC in jugular blood, there was also a low total WBC in the umbilical blood, except for one foal. One foal had a high fibrinogen value in both jugular and umbilical samples—4.4 and 5.2 g/L, respectively—but this foal had the lowest total WBC in the blood (3200/µL). Both WBC and fibrinogen in umbilical blood and jugular blood were strongly correlated (WBC: r = 0.76, *p* < 0.001; fibrinogen: r = 0.855, *p* < 0.001, respectively).

### 3.5. Ion Torrent Sequencing

**Figure 2:**
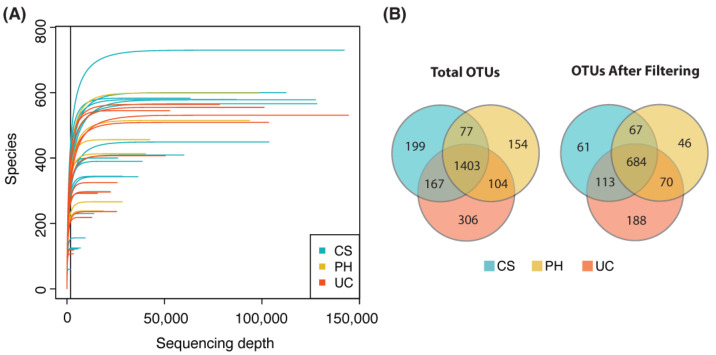
Rarefaction curves for sequencing data and number of OTUs detected. (**A**) Rarefaction curves of all samples rarefied at minimum sequencing depth of 10,000 reads per sample taken from cervical star (CS (yellow)), pregnant horn (PH (blue)) and umbilical cord (UC (red)) regions. (**B**) Venn diagram showing number of OTUs shared between CS, PH and UC samples before and after filtering.

In total, 25,389,075 reads were obtained from 49 samples following the quality filtering (Q ≥ 20), including 16 from cervical star (CS), 15 from pregnant horn (PH), and 18 from umbilical cord (UC). Each sample had between 54,402 and 1,071,610 reads, with an average of 497,825 reads per sample. When analyzing the microbiome between the three placental regions CS, PH and UC, a total of 2410 operational taxonomic units (OTUs) were identified, of which 1846 OTUs were found in CS, 1738 in PH and 1980 in UC (Supplementary Table S1). Any OTUs not found in ≥3 samples in each group, as well as those with high mean relative abundance, which might be contamination sequences, were excluded. After this filtering, 925 OTUs in CS, 867 in PH and 1055 in UC were kept for further analysis. Most rarefaction curves for each sample reached the saturation plateau (Figure 2A), which indicated that there was sufficient sequence coverage to accurately describe the bacterial composition of each group. Among the most common OTUs, defined as being present in at least three samples in each group, 61, 46 and 188 OTUs were unique to the CS, PH and UC regions, respectively (Figure 2B).

### 3.6. Diversity of Bacterial Communities

**Figure 3:**
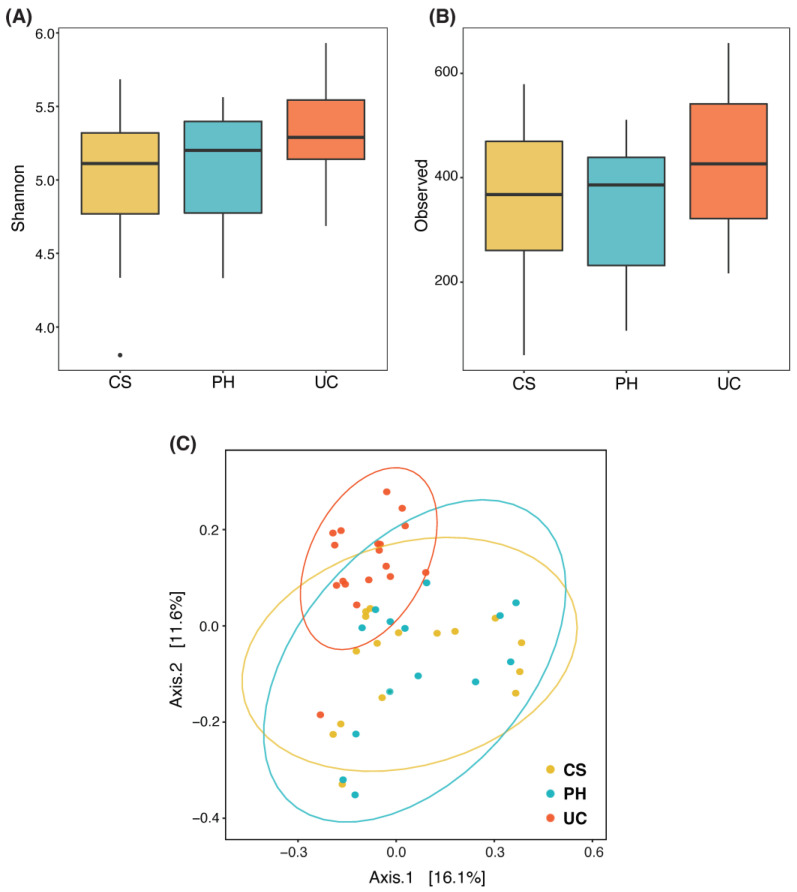
Bacterial richness and beta diversity statistics. (**A**) Plot showing estimates of diversity (Shannon index) and observed species (**B**), in samples analyzed by 16S rRNA sequencing. (**C**) Principal coordinate analysis (PCoA) plot of the Bray–Curtis distances at species level for the samples displaying orientation of the cervical star (yellow), pregnant horn (blue) and umbilical cord (red) sample types.

Indices of alpha diversity, which is the bacterial diversity within the samples, showed that there was no significant difference in microbiome diversity [Shannon index (Figure 3A)], richness [observed OTUs (Figure 3B)] or community evenness (Pielou) between the three placental regions (Kruskal–Wallis *p* > 0.05). However, the placental bacterial community structure (beta diversity), determined by Bray–Curtis dissimilarity and weighted and unweighted UniFrac using PERMANOVA, was significantly different between all three placental groups (*p* < 0.05, Figure 3C).

### 3.7. Taxonomic Composition in Placental Regions

**Figure 4:**
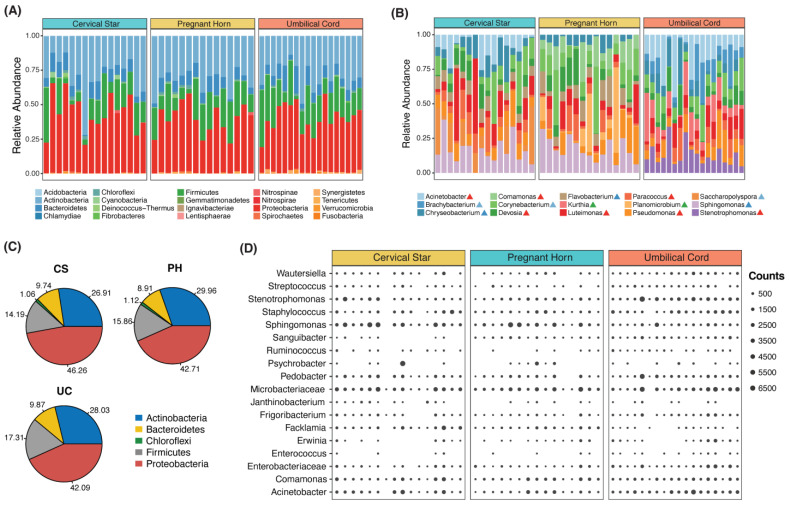
Microbiota composition and abundance of phyla and genera of bacteria present in equine placental tissues. (**A**) Relative abundance of total bacterial taxa identified at the phylum level for each sample in CS, PH and UC. (**B**) Relative abundance of top ten bacterial genera is shown for each sample in the group. Each colored triangle icon beside genus name corresponds to the phylum it belongs to in Figure 4A. (**C**) Average distribution of phyla composition in CS, PH and UC sample groups. (**D**) Bubble plot showing presence of bacterial OTUs reported in Quercia et al. [5] detected in our samples. The size of the dots is relative to read counts showing the abundance of OTUs in each sample.

Twenty phyla were identified in all placental regions (Figure 4A). Proteobacteria, Actinobacteria, Firmicutes and Bacteroidetes were the dominant phyla present in the CS, PH and UC samples. At the genus level, OTUs were assigned to 469, 427 and 527 different genera in the CS, PH and UC region, respectively. From the 10 most abundant bacterial genera identified in each group (Figure 3B and Supplementary Table S2), we observed that *Sphingomonas* was predominant, with an abundance of 3.39%, followed by *Paracoccus* (2.35%), *Luteimonas* (2.18%), *Corynebacterium* (2.14%) and *Saccharopolyspora* (2%) in the CS group. In contrast, in the PH group, the most abundant genera were *Sphingomonas* (3.59%), *Corynebacterium* (2.20%), *Paracoccus* (2.17%), *Devosia* (2.1%) and *Planomicrobium* (1.74%). In the UC samples, the most abundant sequences were those related to *Stenotrophomonas* (2.39%), *Acinetobacter* (2.1%), *Corynebacterium* (1.93%), *Chryseobacterium* (1.9%) and *Luteimonas* (1.82%). We noted that the overall composition of bacterial phyla was similar across all the placental regions (Figure 4C), but a notable shift in microbial community abundance in the UC samples was observed at genera level (Figure 4B).

Unique genera were present in different regions, e.g., in CS, *Saccharopolyspora*; in PH, *Comamonas* and *Planomicrobium*; and in UC, *Stenotrophomonas, Kurthia* and *Brachybacterium.* Proteobacteria were the most abundant phylum (42–46.26%), followed by Actinobacteria (26.91–29.96%), Firmicutes (14.19–17.31%) and Bacteroidetes (8.91–9.87%) (Figure 4C). However, there was a very small representation of some phyla, with only one or two species classified in the three regions (Table 3). The bacteria we found in the amniotic fluid and umbilical blood (Table 2) were detected with 16S sequencing in different locations of the placenta samples. There were also other bacterial taxa found in our samples, as shown in Figure 4D.

## 4. Discussion

The purpose of this study was to examine the possibility of the presence of a microbiome in the amniotic fluid and umbilical blood of healthy foals, as well as in the normal equine placenta.

All foals were healthy with regard to post-foaling parameters, such as being able to stand and nurse within 2 h [13], and SAA values ≤ 5 mg/L [14]. The SAA is a useful tool to monitor the onset of nonspecific inflammatory responses, being low in healthy individuals but rising quickly in response to inflammation [15]. For foals with normal levels of SAA, a lower WBC count is not an indication of illness [16]. The WBC count was 5482–6264/µL blood for most of the foals, which is considered normal [17], although six had 3200–4900 WBC/µL blood. Only five foals needed longer than two hours to stand; a total of 79% were able to stand and nurse within two hours, which is considered to be normal perinatal behavior [13]. In contrast, in a previous study in Sweden, the majority of foals needed at least two hours to stand [4].

The amniotic fluid contained a small number of PMNLs in most mares, in agreement with a previous study, where they were shown to originate from the fetus [18]. Interestingly, even the mares with a high proportion of PMNLs in their amniotic fluid gave birth to healthy foals. The amniotic fluid of five mares had PMNLs ≥ 30%, although this did not appear to be reflected in foal health.

In this study, the bacteriology of the amniotic and the umbilical blood samples was performed by culture. Bacterial growth was found in only 4/22 amniotic samples. The bacteria were *Staph. xylosus, A. lwoffii*, *Str. equi* subsp. *zooepidemicus* and *Ent. faecalis*. Of these, *Acinetobacter lwoffi* and *Enterococcus faecalis* were also found in the feces of the mare, as well as the amniotic fluid and meconium, in a study by Quercia et al. [5]. The low number of samples containing bacteria in the present study could be due to their efficient removal by PMNLs in the amniotic fluid, since there were only five amniotic samples with a high number of PMNLs. Mares with high levels of neutrophils in the amniotic fluid might be responding to a pathogen through the innate immune system, or these neutrophils may be sentinels [8]. In one sample, intracellular bacteria were found, as was also observed in our previous study [4].

Alternatively, the few bacteria found could reflect the problems of trying to culture bacteria; it is not always possible to provide the correct culture conditions for all bacteria to grow, particularly where it is not known which bacteria are likely to be present, and some bacteria are inhibited from growing in the presence of others. This is particularly the case in a clinical veterinary microbiology laboratory where culture is routinely used for the detection of pathogens rather than to discover which bacteria are actually present. These difficulties can be overcome by using a method of identification that is not dependent on first culturing the organism.

In a previous study on murine amniotic fluid, only 1/42 cultures produced bacterial growth [8]. In another study, where samples were taken during elective or emergency cesarean sections in dogs at term, swabs from the uterus, amniotic fluid and meconium produced bacterial growth such as Acinetobacter and Bacillus, whereas control samples were negative [9]. Our results on amniotic fluid and umbilical blood are consistent with their findings. However, we cannot exclude the possibility of contamination, even though care was taken to collect the samples aseptically. Theoretically, it would be possible to transfer bacteria from the surface of the tissue into the amniotic fluid, as has been shown to occur in intraarticular manipulations [19]. However, in such a case, one might have expected more than one or two bacteria to be transferred from the surface of the tissue into the fluid. In support of our findings, it has been suggested by others that fetuses are consistently colonized in utero, with one of the sources being the amniotic fluid [5].

Mols et al. suggested that in utero colonization of the fetal gastrointestinal tract (GIT) is an internal mother–fetus microbiological interaction. For in utero colonization to happen, there would have to be an interaction between the amniotic fluid and the fetus during the development of the foal’s gastrointestinal tract, with the fetus swallowing amniotic fluid [6]. There are several routes by which the fetal compartments could become colonized by bacteria, through the cervix, through the placenta, dendritic cell sampling or via the peripheral circulation [6]. It would be possible to determine if they were maternal or fetal in origin. However, such an investigation is beyond the scope of the present study. In addition, there are anatomical differences between the placenta in horses, mice and dogs, which might result in considerable species differences in a transfer of microbes via the circulation.

A thorough study in bovine fetuses of 5–7 months’ gestation investigated the presence of microbial communities in fetal fluids and tissues (amniotic fluid, tissues and fluid of rumen and cecum). There were differences between tissues and fluids indicating location-specific microbial selection, and the abundance of bacterial and archaeal communities changed between the different gestational times [20].

There is an early bacterial colonization of the fetus [10], as shown when oral inoculation of pregnant mice with a genetically labeled *Enterococcus faecium* strain resulted in this bacterium being isolated in the offspring delivered by cesarian section. The mouse study provides an indicator that bacteria from the amniotic fluid could be in contact with the fetal gut throughout gestation [21].

Values for SAA were ≤5 mg/L in all 24 samples of umbilical blood samples. The most common aerobic growth in the umbilical blood was CNS in 14/22 (64%), mainly being *Staph. sciuri* in 12/14 (87%), which is an opportunistic pathogen of controversial clinical significance [22]. There was no evidence that this bacterium caused any pathogenic effect in the present study. In studies of the bacterial communities of healthy, full-term babies born by cesarian section, commensal bacteria such as *Enterococcus* and *Staphylococcus* were found [11].

Sixteen mares (67%) passed the placenta in less than one hour; no mare had a retained placenta. The normal expulsion time is within three hours. Our results from 16S rRNA sequencing showed an abundance of bacteria in the placenta, with some variation between the different regions. Van Heule et al. [7] also observed a large number of bacteria in the postpartum placenta, more than in preterm samples, albeit from different mares. They attributed the large number of genera seen in postpartum samples to a contribution from the caudal reproductive microbiome. In our study, we observed a large number of reads, implying that some contamination had occurred. However, some bacteria were present either in the amniotic fluid and placenta, or in the umbilical blood and placenta, suggesting that these bacteria, at least, may be part of a microbiome. These results are consistent with a recent study on dog and cat material harvested during cesarean section [23], where a few bacteria were found in amniotic fluid and rather more in the uterus, despite strict hygienic sampling procedures. 

Several studies indicate the presence of a microbiota in the placenta, uterus and fetus [4,5]. There is a similarity between the oral and the placental microbiota, suggesting that bacteria may pass from the oral cavity to the placenta [6,24,25]. Human studies showed that there are distinct differences between the fetal membrane and basal plate region of the placenta based on 16S abundance, as well as bacterial diversity at the phyla and species level. The basal plate, the area where the placenta attaches to the endometrium, is of maternal origin, and is dominated by Proteobacteria and Bacteroidetes [26,27]. However, fetal membranes are dominated by Firmicutes [28]. A unique placental microbiome niche, composed of nonpathogenic commensal microbiota from the Firmicutes, Tenericutes, Proteobacteria, Bacteroidetes and Fusobacteria phyla, was identified [29]. These phyla were found in all the species investigated to date. Three of these phyla (Firmicutes, Proteobacteria, Bacteroidetes) were found in the equine placenta [30,31]. In cases where these phyla were classified to genus level, there were differences within animal species, including in the equine reproductive tract. This was also shown to be the case in human patients, i.e., showing differences in human origin and living conditions. The phyla detected were very similar between studies, differing in location, number and genus among host species. Interestingly, the 10 most abundant bacterial genera identified in PH, CS and UC samples were different, despite the overall composition of bacterial phyla being similar across all the placental regions. There were unique genera present in different regions (Figure 3).

It would have been interesting to analyze the amniotic fluid and fetal blood samples by 16S sequencing in the present study; this analysis should be included in a future study. Although the results of culture show which microorganisms were able to grow in the conditions available during incubation, other microorganisms may well have been present but were prevented from growing because of adverse conditions or because of inhibition by other microorganisms. On the other hand, 16S sequencing reveals the bacterial DNA that is present; it does not indicate whether the DNA represents viable bacteria. Ideally, a future study would incorporate both of the techniques on all samples to aid in interpretation.

Taken together, the results from the present study suggest that amniotic fluid and fetal blood may have their own microbiome in healthy equine pregnancies, as suggested for other species. This microbiome may help to prepare the newborn for a life outside the uterus. Our results overlap with other studies in which the microbiota was reported [4,5,28,29]. However, in our study, the OTUs were very high, suggesting the presence of some contaminants. Even so, the genera identified in the three regions of the placenta in our study were different; it seems unlikely that contamination would have been so localized. Therefore, our results could indicate that the placenta might have its own microbiome, and that it differs in different regions. The difficulties we faced were that the mares foaled spontaneously in a crowded environment, although by the time of placental expulsion, the mare and foal had been moved to a separate box, and the samples were taken as cleanly as possible. However, to take the placenta samples in a completely sterile manner, cesarean section would be required, which was beyond the scope of the present study. Despite the challenges of sampling in such an environment, it was interesting to note that our results were similar to those obtained by sampling during cesarean section in women [29], mice [11] and dogs [10], as well as during reproductive tract surgery in chickens [32].

## 5. Conclusions

In this pilot study, bacteria were isolated from samples of amniotic fluid and umbilical blood from normal-term equine pregnancies. Extraction of DNA from the placenta of the same mares followed by 16S rRNA sequencing enabled several bacterial communities to be identified. There were similarities to the microbiome identified in other species in which the samples were obtained during cesarean section. The high number of bacteria observed suggests that most of these bacteria were likely to be contaminants. However, different bacteria were found in different placental regions; such localization seems unlikely to be due to contamination. Therefore, we can neither prove nor disprove the hypothesis that the placenta has its own microbiome.

## Figures and Tables

**Table 1:** Number and distribution of polymorphonuclear leukocytes (cytology scores) in the amniotic fluid of mares.

Cytological Scores	Number of Amniotic Samples
0	-
1–14%	11 ^§^
15–29%	8
≥30%	5

Note: ^§^ 9 samples contained ≤5% polymorphonuclear leukocytes.

**Table 2:** Bacteria in amnion and umbilicus; white blood cells, serum amyloid A and fibrinogen in foal and umbilicus; length of time to stand and suck, plus time to passing of placenta for 24 foaling mares.

Stand/Suck (h)	Bacteria in AF	Bacteria in UB A/An	WBC × 10^3^/µL ^§^	Fibrinogen g/L ^§^	Placenta min
<1/<1	0	0/0	8.0/10.7	0.63/0.97	40
<1/>2	0	Enterococcus fecalis/0	6.5/7.0	0.76/0.90	60
<1/<2	0	CNS Staph sciuri/ bacillus sp	6.2/6.0	1.83/1.52	55
>1/<2	0	CNS Staph lentus/0	7.6/6.1	1.46/1.54	60
<1/<2	0	CNS Staph sciuri/0	5.7/5.0	1.13/1.13	40
<1/<2	contamination	CNS Staph sciuri/0	3.6/5.0	0.44/0.76	60
<2/<2	*Strept equi* ssp. zooepidemicus	*E coli*/*E coli*	4.2/2.9	0.56/0.90	35
<1/<2	0	0/0	7.7/4.7	0.73/1.35	55
<1/>2	CNS Staph xylosus	Staph sciuri/0	8.1/9.9	0.97/0.88	130
<1/<2	0	*E coli*/*E coli*	6.2/clot, normal	0.79/<<0	50
<1/<2	0	CNS Staph sciuri/0	4.0/4.1	1.37/1.32	45
<2/<2	0	contamination/0	5.7/4.5	1.10/1.31	95
<1/<2	contamination	CNS Staph sciuri/0	4.6/3.4	1.42/1.64	130
<1/<2	0	CNS Staph sciuri/0	7.6/7.3	1.12/0.87	30
<2/<2	0	Bacillus/0	7.4/6.3	1.44/1.72	50
<1/<2	0	CNS Staph sciuri/0	7.4/7.4	2.35/1.32	25
<1/<2	0	0/0	6.4/4.9	1.78/1.81	70
<1/<2	0	CNS -/0	5.8/4.7	2.20/1.59	60
<1/<1	0	CNS Staph sciuri/0	4.9/4.2	1.38/1.27	60
<1/<2	0	CNS Staph sciuri/0	4.9/4.5	1.37/1.47	155
<2/<2	0	CNS Staph sciuri/0	4.7/5.5	1.89/1.01	105
<2/>2	Acinetobacter lwoffii	contamination/0	3.2/3.4	4.42/5.16	35
<1/<2	*Enterococcus faecalis*	CNS Staph sciuri/0	5.5/4.8	1.65/1.20	120
<1/<2	0	0/0	6.6/5.7	0.77/1.36	40

Notes: AF = amniotic fluid; UB = umbilical blood. A/An = aerobic/anaerobic bacteria in umbilical blood; WBC = white blood cells (leukocytes); ^§^ = value for foal/value in umbilical blood; placenta = time taken to pass placenta. << = under threshold of detection.

**Table 3:** Composition of least abundant phyla in three regions of the equine placenta.

Phyla	Abundance % in CS, PH, UC			Family/Genus	Species
Acidobacteria	0.11	0.06	0.05	Acidobacteriaceae, Solibacteraceae	-
Chlamydiae	0.02	x	0.03	Waddliaceae, Criblamydiaceae	-
Cyanobacteria	0.33	0.23	0.21	Gloeobacteraceae, Phormidiaceae	-
Deinococcus-Thermus	0.4	0.21	0.09	Deinococcaceae, Trueperaceae	-
Fibrobacteres	0.16	x	0.06	Fibrobacter, Fibrobacter, Fibrobacteraceae	*Fibrobacter succinogenes*, -, -
Fusobacteria	x	x	0.16	Leptotrichiaceae, Fusobacteriaceae	-
Gemmatimonadetes	0.02	0.02	0.01	Gemmatimonadaceae	-
Ignavibacteriae	0.01	0.02	x	Ignavibacteriaceae	-
Lentisphaerae	0.01	x	x	Victivallaceae	-
Nitrospinae	0.05	0.08	0.03	Nitrospinaceae	-
Nitrospirae	0.01	x	x	Nitrospiraceae	-
Spirochaetes	0.32	0.38	0.2	Treponema, Brachyspiraceae, Spirochaetaceae	*Treponema calligyrum*, -, -
Synergistetes	0.02	0.01	0.56	Synergistaceae, Synergistes, Dethiosulfovibrionaceae	-
Tenericutes	0.1	0.2	0.07	Acholeplasmataceae, CandidatusPhytoplasma	-
Verrucomicrobia	0.28	0.23	0.41	Puniceicoccaceae, Verrucomicrobiaceae	-

Notes: CS = cervical star; PH = pregnant horn; UC = regions adjacent to umbilical cord. x, - = not available.

## Data Availability

The data presented in this study are available on request from the corresponding author.

## Supplementary Materials

The following supporting information can be downloaded at: https://www.mdpi.com/article/10.3390/ani13122029/s1, Table S1: Raw counts of Taxonomic OTUs across all samples; Table S2: Top 10 abundant genera in each placental region.
