# Supplementary material for: Microbial Profiling of Amniotic Fluid, Umbilical Blood and Placenta of the Foaling Mare
Source: Animals (Basel). 2023 Jun 18;13(12):2029. doi: 10.3390/ani13122029 (PMC10295694; doi:10.3390/ani13122029)
Supplement: Supplementary file 1 [file animals-13-02029-s001.zip › animals-2438886-supplementary.pdf]

Phylum	Class	Order
Acidobacteria	Acidobacteriia	Acidobacteriales
Actinobacteria	Acidimicrobiia	Acidimicrobiales
Actinobacteria	Actinobacteria	Acidimicrobiales
Actinobacteria	Actinobacteria	Acidimicrobiales
Actinobacteria	Actinobacteria	Acidimicrobiales
Actinobacteria	Actinobacteria	Acidimicrobiales
Actinobacteria	Actinobacteria	Acidimicrobiales
Actinobacteria	Actinobacteria	Actinomycetales
Actinobacteria	Actinobacteria	Actinomycetales
Actinobacteria	Actinobacteria	Actinomycetales
Actinobacteria	Actinobacteria	Actinomycetales
Actinobacteria	Actinobacteria	Actinomycetales
Actinobacteria	Actinobacteria	Actinomycetales
Actinobacteria	Actinobacteria	Actinomycetales
Actinobacteria	Actinobacteria	Actinomycetales
Actinobacteria	Actinobacteria	Actinomycetales
Actinobacteria	Actinobacteria	Actinomycetales
Actinobacteria	Actinobacteria	Actinomycetales
Actinobacteria	Actinobacteria	Actinomycetales
Actinobacteria	Actinobacteria	Actinomycetales
Actinobacteria	Actinobacteria	Actinomycetales
Actinobacteria	Actinobacteria	Actinomycetales
Actinobacteria	Actinobacteria	Actinomycetales
Actinobacteria	Actinobacteria	Actinomycetales
Actinobacteria	Actinobacteria	Actinomycetales
Actinobacteria	Actinobacteria	Actinomycetales
Actinobacteria	Actinobacteria	Actinomycetales
Actinobacteria	Actinobacteria	Actinomycetales
Actinobacteria	Actinobacteria	Actinomycetales
Actinobacteria	Actinobacteria	Actinomycetales
Actinobacteria	Actinobacteria	Actinomycetales
Actinobacteria	Actinobacteria	Actinomycetales
Actinobacteria	Actinobacteria	Actinomycetales
Actinobacteria	Actinobacteria	Actinomycetales
Actinobacteria	Actinobacteria	Actinomycetales
Actinobacteria	Actinobacteria	Actinomycetales
Actinobacteria	Actinobacteria	Actinomycetales
Actinobacteria	Actinobacteria	Actinomycetales
Actinobacteria	Actinobacteria	Actinomycetales
Actinobacteria	Actinobacteria	Actinomycetales
Actinobacteria	Actinobacteria	Actinomycetales
Actinobacteria	Actinobacteria	Actinomycetales

Actinobacteria	Actinobacteria	Actinomycetales
Actinobacteria	Actinobacteria	Actinomycetales
Actinobacteria	Actinobacteria	Actinomycetales
Actinobacteria	Actinobacteria	Actinomycetales
Actinobacteria	Actinobacteria	Actinomycetales
Actinobacteria	Actinobacteria	Actinomycetales
Actinobacteria	Actinobacteria	Actinomycetales
Actinobacteria	Actinobacteria	Actinomycetales
Actinobacteria	Actinobacteria	Actinomycetales
Actinobacteria	Actinobacteria	Actinomycetales
Actinobacteria	Actinobacteria	Actinomycetales
Actinobacteria	Actinobacteria	Actinomycetales
Actinobacteria	Actinobacteria	Actinomycetales
Actinobacteria	Actinobacteria	Actinomycetales
Actinobacteria	Actinobacteria	Actinomycetales
Actinobacteria	Actinobacteria	Actinomycetales
Actinobacteria	Actinobacteria	Actinomycetales
Actinobacteria	Actinobacteria	Actinomycetales
Actinobacteria	Actinobacteria	Actinomycetales
Actinobacteria	Actinobacteria	Actinomycetales
Actinobacteria	Actinobacteria	Actinomycetales
Actinobacteria	Actinobacteria	Actinomycetales
Actinobacteria	Actinobacteria	Actinomycetales
Actinobacteria	Actinobacteria	Actinomycetales
Actinobacteria	Actinobacteria	Actinomycetales
Actinobacteria	Actinobacteria	Actinomycetales
Actinobacteria	Actinobacteria	Actinomycetales
Actinobacteria	Actinobacteria	Actinomycetales
Actinobacteria	Actinobacteria	Actinomycetales
Actinobacteria	Actinobacteria	Actinomycetales
Actinobacteria	Actinobacteria	Actinomycetales
Actinobacteria	Actinobacteria	Actinomycetales
Actinobacteria	Actinobacteria	Actinomycetales
Actinobacteria	Actinobacteria	Actinomycetales
Actinobacteria	Actinobacteria	Actinomycetales
Actinobacteria	Actinobacteria	Actinomycetales
Actinobacteria	Actinobacteria	Actinomycetales
Actinobacteria	Actinobacteria	Actinomycetales
Actinobacteria	Actinobacteria	Actinomycetales
Actinobacteria	Actinobacteria	Actinomycetales
Actinobacteria	Actinobacteria	Actinomycetales
Actinobacteria	Actinobacteria	Actinomycetales
Actinobacteria	Actinobacteria	Actinomycetales
Actinobacteria	Actinobacteria	Actinomycetales
Actinobacteria	Actinobacteria	Actinomycetales
Actinobacteria	Actinobacteria	Actinomycetales

Actinobacteria	Actinobacteria	Actinomycetales
Actinobacteria	Actinobacteria	Actinomycetales
Actinobacteria	Actinobacteria	Actinomycetales
Actinobacteria	Actinobacteria	Actinomycetales
Actinobacteria	Actinobacteria	Actinomycetales
Actinobacteria	Actinobacteria	Actinomycetales
Actinobacteria	Actinobacteria	Actinomycetales
Actinobacteria	Actinobacteria	Actinomycetales
Actinobacteria	Actinobacteria	Actinomycetales
Actinobacteria	Actinobacteria	Actinomycetales
Actinobacteria	Actinobacteria	Actinomycetales
Actinobacteria	Actinobacteria	Actinomycetales
Actinobacteria	Actinobacteria	Actinomycetales
Actinobacteria	Actinobacteria	Actinomycetales
Actinobacteria	Actinobacteria	Actinomycetales
Actinobacteria	Actinobacteria	Actinomycetales
Actinobacteria	Actinobacteria	Actinomycetales
Actinobacteria	Actinobacteria	Actinomycetales
Actinobacteria	Actinobacteria	Actinomycetales
Actinobacteria	Actinobacteria	Actinomycetales
Actinobacteria	Actinobacteria	Actinomycetales
Actinobacteria	Actinobacteria	Actinomycetales
Actinobacteria	Actinobacteria	Actinomycetales
Actinobacteria	Actinobacteria	Actinomycetales
Actinobacteria	Actinobacteria	Actinomycetales
Actinobacteria	Actinobacteria	Actinomycetales
Actinobacteria	Actinobacteria	Actinomycetales
Actinobacteria	Actinobacteria	Actinomycetales
Actinobacteria	Actinobacteria	Actinomycetales
Actinobacteria	Actinobacteria	Actinomycetales
Actinobacteria	Actinobacteria	Actinomycetales
Actinobacteria	Actinobacteria	Actinomycetales
Actinobacteria	Actinobacteria	Actinomycetales
Actinobacteria	Actinobacteria	Actinomycetales
Actinobacteria	Actinobacteria	Actinomycetales
Actinobacteria	Actinobacteria	Actinomycetales
Actinobacteria	Actinobacteria	Actinomycetales
Actinobacteria	Actinobacteria	Actinomycetales
Actinobacteria	Actinobacteria	Actinomycetales
Actinobacteria	Actinobacteria	Actinomycetales
Actinobacteria	Actinobacteria	Actinomycetales
Actinobacteria	Actinobacteria	Actinomycetales
Actinobacteria	Actinobacteria	Actinomycetales
Actinobacteria	Actinobacteria	Actinomycetales
Actinobacteria	Actinobacteria	Actinomycetales
Actinobacteria	Actinobacteria	Actinomycetales

Actinobacteria	Actinobacteria	Actinomycetales
Actinobacteria	Actinobacteria	Actinomycetales
Actinobacteria	Actinobacteria	Actinomycetales
Actinobacteria	Actinobacteria	Actinomycetales
Actinobacteria	Actinobacteria	Actinomycetales
Actinobacteria	Actinobacteria	Actinomycetales
Actinobacteria	Actinobacteria	Actinomycetales
Actinobacteria	Actinobacteria	Actinomycetales
Actinobacteria	Actinobacteria	Actinomycetales
Actinobacteria	Actinobacteria	Actinomycetales
Actinobacteria	Actinobacteria	Actinomycetales
Actinobacteria	Actinobacteria	Actinomycetales
Actinobacteria	Actinobacteria	Actinomycetales
Actinobacteria	Actinobacteria	Actinomycetales
Actinobacteria	Actinobacteria	Actinomycetales
Actinobacteria	Actinobacteria	Actinomycetales
Actinobacteria	Actinobacteria	Actinomycetales
Actinobacteria	Actinobacteria	Actinomycetales
Actinobacteria	Actinobacteria	Actinomycetales
Actinobacteria	Actinobacteria	Actinomycetales
Actinobacteria	Actinobacteria	Actinomycetales
Actinobacteria	Actinobacteria	Actinomycetales
Actinobacteria	Actinobacteria	Actinomycetales
Actinobacteria	Actinobacteria	Actinomycetales
Actinobacteria	Actinobacteria	Actinomycetales
Actinobacteria	Actinobacteria	Actinomycetales
Actinobacteria	Actinobacteria	Actinomycetales
Actinobacteria	Actinobacteria	Actinomycetales
Actinobacteria	Actinobacteria	Actinomycetales
Actinobacteria	Actinobacteria	Actinomycetales
Actinobacteria	Actinobacteria	Actinomycetales
Actinobacteria	Actinobacteria	Actinomycetales
Actinobacteria	Actinobacteria	Actinomycetales
Actinobacteria	Actinobacteria	Actinomycetales
Actinobacteria	Actinobacteria	Actinomycetales
Actinobacteria	Actinobacteria	Actinomycetales
Actinobacteria	Actinobacteria	Actinomycetales
Actinobacteria	Actinobacteria	Actinomycetales
Actinobacteria	Actinobacteria	Actinomycetales
Actinobacteria	Actinobacteria	Actinomycetales
Actinobacteria	Actinobacteria	Actinomycetales
Actinobacteria	Actinobacteria	Actinomycetales
Actinobacteria	Actinobacteria	Actinomycetales
Actinobacteria	Actinobacteria	Actinomycetales
Actinobacteria	Actinobacteria	Actinomycetales
Actinobacteria	Actinobacteria	Actinomycetales

Actinobacteria	Actinobacteria	Actinomycetales
Actinobacteria	Actinobacteria	Actinomycetales
Actinobacteria	Actinobacteria	Actinomycetales
Actinobacteria	Actinobacteria	Actinomycetales
Actinobacteria	Actinobacteria	Actinomycetales
Actinobacteria	Actinobacteria	Actinomycetales
Actinobacteria	Actinobacteria	Actinomycetales
Actinobacteria	Actinobacteria	Actinomycetales
Actinobacteria	Actinobacteria	Actinomycetales
Actinobacteria	Actinobacteria	Actinomycetales
Actinobacteria	Actinobacteria	Actinomycetales
Actinobacteria	Actinobacteria	Actinomycetales
Actinobacteria	Actinobacteria	Actinomycetales
Actinobacteria	Actinobacteria	Actinomycetales
Actinobacteria	Actinobacteria	Actinomycetales
Actinobacteria	Actinobacteria	Actinomycetales
Actinobacteria	Actinobacteria	Actinomycetales
Actinobacteria	Actinobacteria	Coriobacteriales
Actinobacteria	Actinobacteria	Solirubrobacterales
Actinobacteria	Thermoleophilia	Thermoleophilales
Bacteroidetes	BacteroidetesOrderII.Incertaesedis	BacteroidetesOrderII.Incertaesedis
Bacteroidetes	Bacteroidia	Bacteroidales
Bacteroidetes	Cytophagia	Cytophagales
Bacteroidetes	Cytophagia	Cytophagales
Bacteroidetes	Cytophagia	Cytophagales
Bacteroidetes	Cytophagia	Cytophagales
Bacteroidetes	Cytophagia	Cytophagales
Bacteroidetes	Cytophagia	Cytophagales
Bacteroidetes	Cytophagia	Cytophagales
Bacteroidetes	Cytophagia	Cytophagales
Bacteroidetes	Cytophagia	Cytophagales
Bacteroidetes	Cytophagia	Cytophagales
Bacteroidetes	Cytophagia	Cytophagales
Bacteroidetes	Cytophagia	Cytophagales
Bacteroidetes	Cytophagia	Cytophagales
Bacteroidetes	Flavobacteriia	Flavobacteriales
Bacteroidetes	Flavobacteriia	Flavobacteriales
Bacteroidetes	Flavobacteriia	Flavobacteriales
Bacteroidetes	Flavobacteriia	Flavobacteriales
Bacteroidetes	Flavobacteriia	Flavobacteriales
Bacteroidetes	Flavobacteriia	Flavobacteriales
Bacteroidetes	Flavobacteriia	Flavobacteriales
Bacteroidetes	Flavobacteriia	Flavobacteriales
Bacteroidetes	Flavobacteriia	Flavobacteriales
Bacteroidetes	Flavobacteriia	Flavobacteriales
Bacteroidetes	Flavobacteriia	Flavobacteriales

Bacteroidetes	Flavobacteriia	Flavobacteriales
Bacteroidetes	Flavobacteriia	Flavobacteriales
Bacteroidetes	Flavobacteriia	Flavobacteriales
Bacteroidetes	Flavobacteriia	Flavobacteriales
Bacteroidetes	Flavobacteriia	Flavobacteriales
Bacteroidetes	Flavobacteriia	Flavobacteriales
Bacteroidetes	Flavobacteriia	Flavobacteriales
Bacteroidetes	Flavobacteriia	Flavobacteriales
Bacteroidetes	Flavobacteriia	Flavobacteriales
Bacteroidetes	Flavobacteriia	Flavobacteriales
Bacteroidetes	Flavobacteriia	Flavobacteriales
Bacteroidetes	Flavobacteriia	Flavobacteriales
Bacteroidetes	Flavobacteriia	Flavobacteriales
Bacteroidetes	Flavobacteriia	Flavobacteriales
Bacteroidetes	Flavobacteriia	Flavobacteriales
Bacteroidetes	Flavobacteriia	Flavobacteriales
Bacteroidetes	Flavobacteriia	Flavobacteriales
Bacteroidetes	Flavobacteriia	Flavobacteriales
Bacteroidetes	Flavobacteriia	Flavobacteriales
Bacteroidetes	Flavobacteriia	Flavobacteriales
Bacteroidetes	Flavobacteriia	Flavobacteriales
Bacteroidetes	Flavobacteriia	Flavobacteriales
Bacteroidetes	Flavobacteriia	Flavobacteriales
Bacteroidetes	Sphingobacteriia	Sphingobacteriales
Bacteroidetes	Sphingobacteriia	Sphingobacteriales
Bacteroidetes	Sphingobacteriia	Sphingobacteriales
Bacteroidetes	Sphingobacteriia	Sphingobacteriales
Bacteroidetes	Sphingobacteriia	Sphingobacteriales
Bacteroidetes	Sphingobacteriia	Sphingobacteriales
Bacteroidetes	Sphingobacteriia	Sphingobacteriales
Bacteroidetes	Sphingobacteriia	Sphingobacteriales
Bacteroidetes	Sphingobacteriia	Sphingobacteriales
Bacteroidetes	Sphingobacteriia	Sphingobacteriales
Bacteroidetes	Sphingobacteriia	Sphingobacteriales
Bacteroidetes	Sphingobacteriia	Sphingobacteriales
Bacteroidetes	Sphingobacteriia	Sphingobacteriales
Bacteroidetes	Sphingobacteriia	Sphingobacteriales
Bacteroidetes	Sphingobacteriia	Sphingobacteriales
Chlamydiae	Chlamydiia	Chlamydiales
Chloroflexi	Anaerolineae	Ardenscatenales
Chloroflexi	Ktedonobacteria	Thermogemmatisporales
Chloroflexi	Thermomicrobia	Sphaerobacterales
Cyanobacteria	Gloeobacterophycideae	Gloeobacterales
Cyanobacteria	Nostocophycideae	Nostocales
Deinococcus-Thermus	Deinococci	Deinococcales
Firmicutes	Bacilli	Bacillales

Firmicutes	Bacilli	Bacillales
Firmicutes	Bacilli	Bacillales
Firmicutes	Bacilli	Bacillales
Firmicutes	Bacilli	Bacillales
Firmicutes	Bacilli	Bacillales
Firmicutes	Bacilli	Bacillales
Firmicutes	Bacilli	Bacillales
Firmicutes	Bacilli	Bacillales
Firmicutes	Bacilli	Bacillales
Firmicutes	Bacilli	Bacillales
Firmicutes	Bacilli	Bacillales
Firmicutes	Bacilli	Bacillales
Firmicutes	Bacilli	Bacillales
Firmicutes	Bacilli	Bacillales
Firmicutes	Bacilli	Bacillales
Firmicutes	Bacilli	Bacillales
Firmicutes	Bacilli	Bacillales
Firmicutes	Bacilli	Bacillales
Firmicutes	Bacilli	Bacillales
Firmicutes	Bacilli	Bacillales
Firmicutes	Bacilli	Bacillales
Firmicutes	Bacilli	Bacillales
Firmicutes	Bacilli	Bacillales
Firmicutes	Bacilli	Bacillales
Firmicutes	Bacilli	Bacillales
Firmicutes	Bacilli	Bacillales
Firmicutes	Bacilli	Bacillales
Firmicutes	Bacilli	Bacillales
Firmicutes	Bacilli	Bacillales
Firmicutes	Bacilli	Bacillales
Firmicutes	Bacilli	Bacillales
Firmicutes	Bacilli	Bacillales
Firmicutes	Bacilli	Bacillales
Firmicutes	Bacilli	Bacillales
Firmicutes	Bacilli	Bacillales
Firmicutes	Bacilli	Lactobacillales
Firmicutes	Bacilli	Lactobacillales
Firmicutes	Bacilli	Lactobacillales
Firmicutes	Bacilli	Lactobacillales
Firmicutes	Bacilli	Lactobacillales
Firmicutes	Bacilli	Lactobacillales
Firmicutes	Bacilli	Lactobacillales
Firmicutes	Bacilli	Lactobacillales
Firmicutes	Bacilli	Lactobacillales
Firmicutes	Bacilli	Lactobacillales
Firmicutes	Bacilli	Lactobacillales

Firmicutes	Clostridia	Clostridiales
Firmicutes	Clostridia	Clostridiales
Firmicutes	Clostridia	Clostridiales
Firmicutes	Clostridia	Clostridiales
Firmicutes	Clostridia	Clostridiales
Firmicutes	Clostridia	Clostridiales
Firmicutes	Clostridia	Clostridiales
Firmicutes	Clostridia	Clostridiales
Firmicutes	Clostridia	Clostridiales
Firmicutes	Clostridia	Clostridiales
Firmicutes	Clostridia	Clostridiales
Firmicutes	Clostridia	Clostridiales
Firmicutes	Clostridia	Clostridiales
Firmicutes	Clostridia	Natranaerobiales
Gemmatimonadetes	Gemmatimonadetes	Gemmatimonadales
Ignavibacteriae	Ignavibacteria	Ignavibacteriales
Nitrospinae	Nitrospinia	Nitrospinales
Proteobacteria	Alphaproteobacteria	Caulobacterales
Proteobacteria	Alphaproteobacteria	Caulobacterales
Proteobacteria	Alphaproteobacteria	Caulobacterales
Proteobacteria	Alphaproteobacteria	Caulobacterales
Proteobacteria	Alphaproteobacteria	Caulobacterales
Proteobacteria	Alphaproteobacteria	Caulobacterales
Proteobacteria	Alphaproteobacteria	Caulobacterales
Proteobacteria	Alphaproteobacteria	Caulobacterales
Proteobacteria	Alphaproteobacteria	Caulobacterales
Proteobacteria	Alphaproteobacteria	Caulobacterales
Proteobacteria	Alphaproteobacteria	Caulobacterales
Proteobacteria	Alphaproteobacteria	Caulobacterales
Proteobacteria	Alphaproteobacteria	Caulobacterales
Proteobacteria	Alphaproteobacteria	Caulobacterales
Proteobacteria	Alphaproteobacteria	Caulobacterales
Proteobacteria	Alphaproteobacteria	Caulobacterales
Proteobacteria	Alphaproteobacteria	Caulobacterales
Proteobacteria	Alphaproteobacteria	Caulobacterales
Proteobacteria	Alphaproteobacteria	Caulobacterales
Proteobacteria	Alphaproteobacteria	Caulobacterales
Proteobacteria	Alphaproteobacteria	Caulobacterales
Proteobacteria	Alphaproteobacteria	Caulobacterales
Proteobacteria	Alphaproteobacteria	Rhizobiales
Proteobacteria	Alphaproteobacteria	Rhizobiales
Proteobacteria	Alphaproteobacteria	Rhizobiales
Proteobacteria	Alphaproteobacteria	Rhizobiales
Proteobacteria	Alphaproteobacteria	Rhizobiales
Proteobacteria	Alphaproteobacteria	Rhizobiales
Proteobacteria	Alphaproteobacteria	Rhizobiales

Proteobacteria	Alphaproteobacteria	Rhizobiales
Proteobacteria	Alphaproteobacteria	Rhizobiales
Proteobacteria	Alphaproteobacteria	Rhizobiales
Proteobacteria	Alphaproteobacteria	Rhizobiales
Proteobacteria	Alphaproteobacteria	Rhizobiales
Proteobacteria	Alphaproteobacteria	Rhizobiales
Proteobacteria	Alphaproteobacteria	Rhizobiales
Proteobacteria	Alphaproteobacteria	Rhizobiales
Proteobacteria	Alphaproteobacteria	Rhizobiales
Proteobacteria	Alphaproteobacteria	Rhizobiales
Proteobacteria	Alphaproteobacteria	Rhizobiales
Proteobacteria	Alphaproteobacteria	Rhizobiales
Proteobacteria	Alphaproteobacteria	Rhizobiales
Proteobacteria	Alphaproteobacteria	Rhizobiales
Proteobacteria	Alphaproteobacteria	Rhizobiales
Proteobacteria	Alphaproteobacteria	Rhizobiales
Proteobacteria	Alphaproteobacteria	Rhizobiales
Proteobacteria	Alphaproteobacteria	Rhizobiales
Proteobacteria	Alphaproteobacteria	Rhizobiales
Proteobacteria	Alphaproteobacteria	Rhizobiales
Proteobacteria	Alphaproteobacteria	Rhizobiales
Proteobacteria	Alphaproteobacteria	Rhizobiales
Proteobacteria	Alphaproteobacteria	Rhizobiales
Proteobacteria	Alphaproteobacteria	Rhizobiales
Proteobacteria	Alphaproteobacteria	Rhizobiales
Proteobacteria	Alphaproteobacteria	Rhizobiales
Proteobacteria	Alphaproteobacteria	Rhizobiales
Proteobacteria	Alphaproteobacteria	Rhizobiales
Proteobacteria	Alphaproteobacteria	Rhizobiales
Proteobacteria	Alphaproteobacteria	Rhizobiales
Proteobacteria	Alphaproteobacteria	Rhizobiales
Proteobacteria	Alphaproteobacteria	Rhizobiales
Proteobacteria	Alphaproteobacteria	Rhizobiales
Proteobacteria	Alphaproteobacteria	Rhizobiales
Proteobacteria	Alphaproteobacteria	Rhizobiales
Proteobacteria	Alphaproteobacteria	Rhizobiales
Proteobacteria	Alphaproteobacteria	Rhizobiales
Proteobacteria	Alphaproteobacteria	Rhizobiales
Proteobacteria	Alphaproteobacteria	Rhizobiales
Proteobacteria	Alphaproteobacteria	Rhizobiales
Proteobacteria	Alphaproteobacteria	Rhizobiales
Proteobacteria	Alphaproteobacteria	Rhizobiales
Proteobacteria	Alphaproteobacteria	Rhizobiales
Proteobacteria	Alphaproteobacteria	Rhizobiales
Proteobacteria	Alphaproteobacteria	Rhizobiales
Proteobacteria	Alphaproteobacteria	Rhizobiales

Proteobacteria	Alphaproteobacteria	Rhizobiales
Proteobacteria	Alphaproteobacteria	Rhizobiales
Proteobacteria	Alphaproteobacteria	Rhizobiales
Proteobacteria	Alphaproteobacteria	Rhizobiales
Proteobacteria	Alphaproteobacteria	Rhizobiales
Proteobacteria	Alphaproteobacteria	Rhizobiales
Proteobacteria	Alphaproteobacteria	Rhizobiales
Proteobacteria	Alphaproteobacteria	Rhizobiales
Proteobacteria	Alphaproteobacteria	Rhizobiales
Proteobacteria	Alphaproteobacteria	Rhizobiales
Proteobacteria	Alphaproteobacteria	Rhizobiales
Proteobacteria	Alphaproteobacteria	Rhizobiales
Proteobacteria	Alphaproteobacteria	Rhizobiales
Proteobacteria	Alphaproteobacteria	Rhizobiales
Proteobacteria	Alphaproteobacteria	Rhizobiales
Proteobacteria	Alphaproteobacteria	Rhizobiales
Proteobacteria	Alphaproteobacteria	Rhizobiales
Proteobacteria	Alphaproteobacteria	Rhizobiales
Proteobacteria	Alphaproteobacteria	Rhizobiales
Proteobacteria	Alphaproteobacteria	Rhizobiales
Proteobacteria	Alphaproteobacteria	Rhizobiales
Proteobacteria	Alphaproteobacteria	Rhizobiales
Proteobacteria	Alphaproteobacteria	Rhizobiales
Proteobacteria	Alphaproteobacteria	Rhodobacterales
Proteobacteria	Alphaproteobacteria	Rhodobacterales
Proteobacteria	Alphaproteobacteria	Rhodobacterales
Proteobacteria	Alphaproteobacteria	Rhodobacterales
Proteobacteria	Alphaproteobacteria	Rhodobacterales
Proteobacteria	Alphaproteobacteria	Rhodobacterales
Proteobacteria	Alphaproteobacteria	Rhodobacterales
Proteobacteria	Alphaproteobacteria	Rhodobacterales
Proteobacteria	Alphaproteobacteria	Rhodobacterales
Proteobacteria	Alphaproteobacteria	Rhodobacterales
Proteobacteria	Alphaproteobacteria	Rhodobacterales
Proteobacteria	Alphaproteobacteria	Rhodobacterales
Proteobacteria	Alphaproteobacteria	Rhodobacterales
Proteobacteria	Alphaproteobacteria	Rhodobacterales
Proteobacteria	Alphaproteobacteria	Rhodobacterales
Proteobacteria	Alphaproteobacteria	Rhodobacterales
Proteobacteria	Alphaproteobacteria	Rhodobacterales
Proteobacteria	Alphaproteobacteria	Rhodobacterales
Proteobacteria	Alphaproteobacteria	Rhodobacterales
Proteobacteria	Alphaproteobacteria	Rhodobacterales
Proteobacteria	Alphaproteobacteria	Rhodobacterales
Proteobacteria	Alphaproteobacteria	Rhodobacterales
Proteobacteria	Alphaproteobacteria	Rhodobacterales

Proteobacteria	Alphaproteobacteria	Rhodobacterales
Proteobacteria	Alphaproteobacteria	Rhodobacterales
Proteobacteria	Alphaproteobacteria	Rhodobacterales
Proteobacteria	Alphaproteobacteria	Rhodobacterales
Proteobacteria	Alphaproteobacteria	Rhodobacterales
Proteobacteria	Alphaproteobacteria	Rhodobacterales
Proteobacteria	Alphaproteobacteria	Rhodobacterales
Proteobacteria	Alphaproteobacteria	Rhodobacterales
Proteobacteria	Alphaproteobacteria	Rhodobacterales
Proteobacteria	Alphaproteobacteria	Rhodobacterales
Proteobacteria	Alphaproteobacteria	Rhodobacterales
Proteobacteria	Alphaproteobacteria	Rhodobacterales
Proteobacteria	Alphaproteobacteria	Rhodobacterales
Proteobacteria	Alphaproteobacteria	Rhodobacterales
Proteobacteria	Alphaproteobacteria	Rhodospirillales
Proteobacteria	Alphaproteobacteria	Rhodospirillales
Proteobacteria	Alphaproteobacteria	Rhodospirillales
Proteobacteria	Alphaproteobacteria	Rhodospirillales
Proteobacteria	Alphaproteobacteria	Rhodospirillales
Proteobacteria	Alphaproteobacteria	Rhodospirillales
Proteobacteria	Alphaproteobacteria	Rhodospirillales
Proteobacteria	Alphaproteobacteria	Rickettsiales
Proteobacteria	Alphaproteobacteria	Sphingomonadales
Proteobacteria	Alphaproteobacteria	Sphingomonadales
Proteobacteria	Alphaproteobacteria	Sphingomonadales
Proteobacteria	Alphaproteobacteria	Sphingomonadales
Proteobacteria	Alphaproteobacteria	Sphingomonadales
Proteobacteria	Alphaproteobacteria	Sphingomonadales
Proteobacteria	Alphaproteobacteria	Sphingomonadales
Proteobacteria	Alphaproteobacteria	Sphingomonadales
Proteobacteria	Alphaproteobacteria	Sphingomonadales
Proteobacteria	Alphaproteobacteria	Sphingomonadales
Proteobacteria	Alphaproteobacteria	Sphingomonadales
Proteobacteria	Alphaproteobacteria	Sphingomonadales
Proteobacteria	Alphaproteobacteria	Sphingomonadales
Proteobacteria	Alphaproteobacteria	Sphingomonadales
Proteobacteria	Alphaproteobacteria	Sphingomonadales
Proteobacteria	Alphaproteobacteria	Sphingomonadales
Proteobacteria	Alphaproteobacteria	Sphingomonadales
Proteobacteria	Alphaproteobacteria	Sphingomonadales
Proteobacteria	Alphaproteobacteria	Sphingomonadales
Proteobacteria	Alphaproteobacteria	Sphingomonadales
Proteobacteria	Alphaproteobacteria	Sphingomonadales
Proteobacteria	Alphaproteobacteria	Sphingomonadales
Proteobacteria	Alphaproteobacteria	Sphingomonadales
Proteobacteria	Alphaproteobacteria	Sphingomonadales

Proteobacteria	Alphaproteobacteria	Sphingomonadales
Proteobacteria	Alphaproteobacteria	Sphingomonadales
Proteobacteria	Alphaproteobacteria	Sphingomonadales
Proteobacteria	Alphaproteobacteria	Sphingomonadales
Proteobacteria	Alphaproteobacteria	Sphingomonadales
Proteobacteria	Alphaproteobacteria	Sphingomonadales
Proteobacteria	Alphaproteobacteria	Sphingomonadales
Proteobacteria	Alphaproteobacteria	Sphingomonadales
Proteobacteria	Alphaproteobacteria	Sphingomonadales
Proteobacteria	Alphaproteobacteria	Sphingomonadales
Proteobacteria	Alphaproteobacteria	Sphingomonadales
Proteobacteria	Alphaproteobacteria	Sphingomonadales
Proteobacteria	Alphaproteobacteria	Sphingomonadales
Proteobacteria	Alphaproteobacteria	Sphingomonadales
Proteobacteria	Alphaproteobacteria	Sphingomonadales
Proteobacteria	Alphaproteobacteria	Sphingomonadales
Proteobacteria	Alphaproteobacteria	Sphingomonadales
Proteobacteria	Alphaproteobacteria	Sphingomonadales
Proteobacteria	Alphaproteobacteria	Sphingomonadales
Proteobacteria	Alphaproteobacteria	Sphingomonadales
Proteobacteria	Alphaproteobacteria	Sphingomonadales
Proteobacteria	Alphaproteobacteria	Sphingomonadales
Proteobacteria	Alphaproteobacteria	Sphingomonadales
Proteobacteria	Alphaproteobacteria	Sphingomonadales
Proteobacteria	Alphaproteobacteria	Sphingomonadales
Proteobacteria	Alphaproteobacteria	Sphingomonadales
Proteobacteria	Alphaproteobacteria	Sphingomonadales
Proteobacteria	Alphaproteobacteria	Sphingomonadales
Proteobacteria	Alphaproteobacteria	Sphingomonadales
Proteobacteria	Alphaproteobacteria	Sphingomonadales
Proteobacteria	Alphaproteobacteria	Sphingomonadales
Proteobacteria	Alphaproteobacteria	Sphingomonadales
Proteobacteria	Betaproteobacteria	Burkholderiales
Proteobacteria	Betaproteobacteria	Burkholderiales
Proteobacteria	Betaproteobacteria	Burkholderiales
Proteobacteria	Betaproteobacteria	Burkholderiales
Proteobacteria	Betaproteobacteria	Burkholderiales
Proteobacteria	Betaproteobacteria	Burkholderiales
Proteobacteria	Betaproteobacteria	Burkholderiales
Proteobacteria	Betaproteobacteria	Burkholderiales
Proteobacteria	Betaproteobacteria	Burkholderiales
Proteobacteria	Betaproteobacteria	Burkholderiales
Proteobacteria	Betaproteobacteria	Burkholderiales
Proteobacteria	Betaproteobacteria	Burkholderiales
Proteobacteria	Betaproteobacteria	Burkholderiales
Proteobacteria	Betaproteobacteria	Burkholderiales

Proteobacteria	Betaproteobacteria	Burkholderiales
Proteobacteria	Betaproteobacteria	Burkholderiales
Proteobacteria	Betaproteobacteria	Burkholderiales
Proteobacteria	Betaproteobacteria	Burkholderiales
Proteobacteria	Betaproteobacteria	Burkholderiales
Proteobacteria	Betaproteobacteria	Burkholderiales
Proteobacteria	Betaproteobacteria	Burkholderiales
Proteobacteria	Betaproteobacteria	Burkholderiales
Proteobacteria	Betaproteobacteria	Burkholderiales
Proteobacteria	Betaproteobacteria	Burkholderiales
Proteobacteria	Betaproteobacteria	Burkholderiales
Proteobacteria	Betaproteobacteria	Burkholderiales
Proteobacteria	Betaproteobacteria	Burkholderiales
Proteobacteria	Betaproteobacteria	Burkholderiales
Proteobacteria	Betaproteobacteria	Burkholderiales
Proteobacteria	Betaproteobacteria	Burkholderiales
Proteobacteria	Betaproteobacteria	Burkholderiales
Proteobacteria	Betaproteobacteria	Burkholderiales
Proteobacteria	Betaproteobacteria	Burkholderiales
Proteobacteria	Betaproteobacteria	Burkholderiales
Proteobacteria	Betaproteobacteria	Burkholderiales
Proteobacteria	Betaproteobacteria	Burkholderiales
Proteobacteria	Betaproteobacteria	Burkholderiales
Proteobacteria	Betaproteobacteria	Burkholderiales
Proteobacteria	Betaproteobacteria	Burkholderiales
Proteobacteria	Betaproteobacteria	Burkholderiales
Proteobacteria	Betaproteobacteria	Burkholderiales
Proteobacteria	Betaproteobacteria	Burkholderiales
Proteobacteria	Betaproteobacteria	Burkholderiales
Proteobacteria	Betaproteobacteria	Burkholderiales
Proteobacteria	Betaproteobacteria	Burkholderiales
Proteobacteria	Betaproteobacteria	Neisseriales
Proteobacteria	Betaproteobacteria	Neisseriales
Proteobacteria	Betaproteobacteria	Rhodocyclales
Proteobacteria	Deltaproteobacteria	Bdellovibrionales
Proteobacteria	Deltaproteobacteria	Bdellovibrionales
Proteobacteria	Deltaproteobacteria	Bdellovibrionales
Proteobacteria	Deltaproteobacteria	Bdellovibrionales
Proteobacteria	Deltaproteobacteria	Bdellovibrionales
Proteobacteria	Deltaproteobacteria	Desulfobacterales
Proteobacteria	Deltaproteobacteria	Myxococcales
Proteobacteria	Epsilonproteobacteria	Campylobacterales
Proteobacteria	Gammaproteobacteria	Alteromonadales
Proteobacteria	Gammaproteobacteria	Alteromonadales
Proteobacteria	Gammaproteobacteria	Alteromonadales
Proteobacteria	Gammaproteobacteria	Alteromonadales

Proteobacteria	Gammaproteobacteria	Alteromonadales
Proteobacteria	Gammaproteobacteria	Alteromonadales
Proteobacteria	Gammaproteobacteria	Alteromonadales
Proteobacteria	Gammaproteobacteria	Alteromonadales
Proteobacteria	Gammaproteobacteria	Chromatiales
Proteobacteria	Gammaproteobacteria	Chromatiales
Proteobacteria	Gammaproteobacteria	Chromatiales
Proteobacteria	Gammaproteobacteria	Enterobacteriales
Proteobacteria	Gammaproteobacteria	Enterobacteriales
Proteobacteria	Gammaproteobacteria	Enterobacteriales
Proteobacteria	Gammaproteobacteria	Enterobacteriales
Proteobacteria	Gammaproteobacteria	Legionellales
Proteobacteria	Gammaproteobacteria	Legionellales
Proteobacteria	Gammaproteobacteria	Methylococcales
Proteobacteria	Gammaproteobacteria	Methylococcales
Proteobacteria	Gammaproteobacteria	Methylococcales
Proteobacteria	Gammaproteobacteria	Oceanospirillales
Proteobacteria	Gammaproteobacteria	Oceanospirillales
Proteobacteria	Gammaproteobacteria	Oceanospirillales
Proteobacteria	Gammaproteobacteria	Oceanospirillales
Proteobacteria	Gammaproteobacteria	Oceanospirillales
Proteobacteria	Gammaproteobacteria	Oceanospirillales
Proteobacteria	Gammaproteobacteria	Oceanospirillales
Proteobacteria	Gammaproteobacteria	Pasteurellales
Proteobacteria	Gammaproteobacteria	Pasteurellales
Proteobacteria	Gammaproteobacteria	Pseudomonadales
Proteobacteria	Gammaproteobacteria	Pseudomonadales
Proteobacteria	Gammaproteobacteria	Pseudomonadales
Proteobacteria	Gammaproteobacteria	Pseudomonadales
Proteobacteria	Gammaproteobacteria	Pseudomonadales
Proteobacteria	Gammaproteobacteria	Pseudomonadales
Proteobacteria	Gammaproteobacteria	Pseudomonadales
Proteobacteria	Gammaproteobacteria	Pseudomonadales
Proteobacteria	Gammaproteobacteria	Pseudomonadales
Proteobacteria	Gammaproteobacteria	Pseudomonadales
Proteobacteria	Gammaproteobacteria	Pseudomonadales
Proteobacteria	Gammaproteobacteria	Pseudomonadales
Proteobacteria	Gammaproteobacteria	Pseudomonadales
Proteobacteria	Gammaproteobacteria	Pseudomonadales
Proteobacteria	Gammaproteobacteria	Pseudomonadales
Proteobacteria	Gammaproteobacteria	Pseudomonadales
Proteobacteria	Gammaproteobacteria	Pseudomonadales
Proteobacteria	Gammaproteobacteria	Pseudomonadales
Proteobacteria	Gammaproteobacteria	Pseudomonadales
Proteobacteria	Gammaproteobacteria	Pseudomonadales
Proteobacteria	Gammaproteobacteria	Pseudomonadales

Proteobacteria	Gammaproteobacteria	Pseudomonadales
Proteobacteria	Gammaproteobacteria	Pseudomonadales
Proteobacteria	Gammaproteobacteria	Pseudomonadales
Proteobacteria	Gammaproteobacteria	Pseudomonadales
Proteobacteria	Gammaproteobacteria	Pseudomonadales
Proteobacteria	Gammaproteobacteria	Pseudomonadales
Proteobacteria	Gammaproteobacteria	Pseudomonadales
Proteobacteria	Gammaproteobacteria	Pseudomonadales
Proteobacteria	Gammaproteobacteria	Pseudomonadales
Proteobacteria	Gammaproteobacteria	Thiotrichales
Proteobacteria	Gammaproteobacteria	Thiotrichales
Proteobacteria	Gammaproteobacteria	Thiotrichales
Proteobacteria	Gammaproteobacteria	Xanthomonadales
Proteobacteria	Gammaproteobacteria	Xanthomonadales
Proteobacteria	Gammaproteobacteria	Xanthomonadales
Proteobacteria	Gammaproteobacteria	Xanthomonadales
Proteobacteria	Gammaproteobacteria	Xanthomonadales
Proteobacteria	Gammaproteobacteria	Xanthomonadales
Proteobacteria	Gammaproteobacteria	Xanthomonadales
Proteobacteria	Gammaproteobacteria	Xanthomonadales
Proteobacteria	Gammaproteobacteria	Xanthomonadales
Proteobacteria	Gammaproteobacteria	Xanthomonadales
Proteobacteria	Gammaproteobacteria	Xanthomonadales
Proteobacteria	Gammaproteobacteria	Xanthomonadales
Proteobacteria	Gammaproteobacteria	Xanthomonadales
Proteobacteria	Gammaproteobacteria	Xanthomonadales
Proteobacteria	Gammaproteobacteria	Xanthomonadales
Proteobacteria	Gammaproteobacteria	Xanthomonadales
Proteobacteria	Gammaproteobacteria	Xanthomonadales
Proteobacteria	Gammaproteobacteria	Xanthomonadales
Proteobacteria	Gammaproteobacteria	Xanthomonadales
Proteobacteria	Gammaproteobacteria	Xanthomonadales
Proteobacteria	Gammaproteobacteria	Xanthomonadales
Proteobacteria	Gammaproteobacteria	Xanthomonadales
Proteobacteria	Gammaproteobacteria	Xanthomonadales
Proteobacteria	Gammaproteobacteria	Xanthomonadales
Proteobacteria	Gammaproteobacteria	Xanthomonadales
Proteobacteria	Gammaproteobacteria	Xanthomonadales
Proteobacteria	Gammaproteobacteria	Xanthomonadales
Proteobacteria	Gammaproteobacteria	Xanthomonadales
Proteobacteria	Gammaproteobacteria	Xanthomonadales
Proteobacteria	Gammaproteobacteria	Xanthomonadales
Proteobacteria	Gammaproteobacteria	Xanthomonadales
Proteobacteria	Gammaproteobacteria	Xanthomonadales
Proteobacteria	Gammaproteobacteria	Xanthomonadales
Proteobacteria	Gammaproteobacteria	Xanthomonadales

Proteobacteria	Gammaproteobacteria	Xanthomonadales
Proteobacteria	Gammaproteobacteria	unclassifiedGammaproteobacteria
Spirochaetes	Spirochaetia	Spirochaetales
Spirochaetes	Spirochaetia	Spirochaetales
Tenericutes	Mollicutes	Acholeplasmatales
Tenericutes	Mollicutes	Acholeplasmatales
Tenericutes	Mollicutes	Acholeplasmatales
Tenericutes	Mollicutes	Mycoplasmatales
Verrucomicrobia	Opitutae	Puniceicoccales
Verrucomicrobia	Verrucomicrobiae	Verrucomicrobiales
Acidobacteria	Solibacteres	Solibacterales
Actinobacteria	Actinobacteria	Actinomycetales
Actinobacteria	Actinobacteria	Actinomycetales
Actinobacteria	Actinobacteria	Actinomycetales
Actinobacteria	Actinobacteria	Actinomycetales
Actinobacteria	Actinobacteria	Actinomycetales
Actinobacteria	Actinobacteria	Actinomycetales
Actinobacteria	Actinobacteria	Actinomycetales
Actinobacteria	Actinobacteria	Actinomycetales
Actinobacteria	Actinobacteria	Actinomycetales
Actinobacteria	Actinobacteria	Actinomycetales
Actinobacteria	Actinobacteria	Actinomycetales
Actinobacteria	Actinobacteria	Actinomycetales
Actinobacteria	Actinobacteria	Actinomycetales
Actinobacteria	Actinobacteria	Actinomycetales
Actinobacteria	Actinobacteria	Actinomycetales
Actinobacteria	Actinobacteria	Actinomycetales
Actinobacteria	Actinobacteria	Actinomycetales
Actinobacteria	Actinobacteria	Actinomycetales
Actinobacteria	Actinobacteria	Actinomycetales
Actinobacteria	Actinobacteria	Actinomycetales
Actinobacteria	Actinobacteria	Actinomycetales
Actinobacteria	Actinobacteria	Actinomycetales
Actinobacteria	Actinobacteria	Actinomycetales
Actinobacteria	Actinobacteria	Actinomycetales
Actinobacteria	Actinobacteria	Actinomycetales
Actinobacteria	Actinobacteria	Actinomycetales
Actinobacteria	Actinobacteria	Actinomycetales
Actinobacteria	Actinobacteria	Actinomycetales
Actinobacteria	Actinobacteria	Actinomycetales
Actinobacteria	Actinobacteria	Actinomycetales
Actinobacteria	Actinobacteria	Actinomycetales
Actinobacteria	Actinobacteria	Actinomycetales
Actinobacteria	Actinobacteria	Actinomycetales
Actinobacteria	Actinobacteria	Actinomycetales
Actinobacteria	Actinobacteria	Actinomycetales
Actinobacteria	Actinobacteria	Actinomycetales

Actinobacteria	Actinobacteria	Actinomycetales
Actinobacteria	Actinobacteria	Actinomycetales
Actinobacteria	Actinobacteria	Actinomycetales
Actinobacteria	Actinobacteria	Actinomycetales
Actinobacteria	Actinobacteria	Actinomycetales
Actinobacteria	Actinobacteria	Actinomycetales
Actinobacteria	Actinobacteria	Actinomycetales
Actinobacteria	Actinobacteria	Actinomycetales
Actinobacteria	Actinobacteria	Actinomycetales
Actinobacteria	Actinobacteria	Actinomycetales
Actinobacteria	Actinobacteria	Actinomycetales
Actinobacteria	Actinobacteria	Actinomycetales
Actinobacteria	Actinobacteria	Actinomycetales
Actinobacteria	Actinobacteria	Actinomycetales
Actinobacteria	Actinobacteria	Actinomycetales
Actinobacteria	Actinobacteria	Actinomycetales
Actinobacteria	Actinobacteria	Actinomycetales
Actinobacteria	Actinobacteria	Actinomycetales
Actinobacteria	Actinobacteria	Actinomycetales
Actinobacteria	Actinobacteria	Actinomycetales
Actinobacteria	Actinobacteria	Actinomycetales
Actinobacteria	Actinobacteria	Actinomycetales
Actinobacteria	Actinobacteria	Actinomycetales
Actinobacteria	Actinobacteria	Actinomycetales
Actinobacteria	Actinobacteria	Actinomycetales
Actinobacteria	Actinobacteria	Actinomycetales
Actinobacteria	Actinobacteria	Actinomycetales
Actinobacteria	Actinobacteria	Actinomycetales
Actinobacteria	Actinobacteria	Actinomycetales
Actinobacteria	Actinobacteria	Actinomycetales
Actinobacteria	Actinobacteria	Actinomycetales
Actinobacteria	Actinobacteria	Actinomycetales
Actinobacteria	Actinobacteria	Actinomycetales
Actinobacteria	Actinobacteria	Actinomycetales
Actinobacteria	Actinobacteria	Actinomycetales
Actinobacteria	Actinobacteria	Actinomycetales
Actinobacteria	Actinobacteria	Actinomycetales
Actinobacteria	Actinobacteria	Actinomycetales
Actinobacteria	Actinobacteria	Actinomycetales
Actinobacteria	Actinobacteria	Actinomycetales
Actinobacteria	Actinobacteria	Actinomycetales
Actinobacteria	Actinobacteria	Actinomycetales
Actinobacteria	Actinobacteria	Actinomycetales
Actinobacteria	Actinobacteria	Actinomycetales
Actinobacteria	Actinobacteria	Actinomycetales
Actinobacteria	Actinobacteria	Actinomycetales

Actinobacteria	Actinobacteria	Actinomycetales
Actinobacteria	Actinobacteria	Actinomycetales
Actinobacteria	Actinobacteria	Actinomycetales
Actinobacteria	Actinobacteria	Actinomycetales
Actinobacteria	Actinobacteria	Actinomycetales
Actinobacteria	Actinobacteria	Actinomycetales
Actinobacteria	Actinobacteria	Actinomycetales
Actinobacteria	Actinobacteria	Actinomycetales
Actinobacteria	Actinobacteria	Actinomycetales
Actinobacteria	Actinobacteria	Actinomycetales
Actinobacteria	Actinobacteria	Bifidobacteriales
Actinobacteria	Actinobacteria	Solirubrobacterales
Bacteroidetes	Bacteroidia	Bacteroidales
Bacteroidetes	Bacteroidia	Bacteroidales
Bacteroidetes	Bacteroidia	Bacteroidales
Bacteroidetes	Bacteroidia	Bacteroidales
Bacteroidetes	Bacteroidia	Bacteroidales
Bacteroidetes	Bacteroidia	Bacteroidales
Bacteroidetes	Bacteroidia	Bacteroidales
Bacteroidetes	Bacteroidia	Bacteroidales
Bacteroidetes	Bacteroidia	Bacteroidales
Bacteroidetes	Bacteroidia	Bacteroidales
Bacteroidetes	Bacteroidia	Bacteroidales
Bacteroidetes	Cytophagia	Cytophagales
Bacteroidetes	Cytophagia	Cytophagales
Bacteroidetes	Flavobacteriia	Flavobacteriales
Bacteroidetes	Flavobacteriia	Flavobacteriales
Bacteroidetes	Flavobacteriia	Flavobacteriales
Bacteroidetes	Flavobacteriia	Flavobacteriales
Bacteroidetes	Flavobacteriia	Flavobacteriales
Bacteroidetes	Flavobacteriia	Flavobacteriales
Bacteroidetes	Flavobacteriia	Flavobacteriales
Bacteroidetes	Flavobacteriia	Flavobacteriales
Bacteroidetes	Flavobacteriia	Flavobacteriales
Bacteroidetes	Sphingobacteriia	Sphingobacteriales
Bacteroidetes	Sphingobacteriia	Sphingobacteriales
Bacteroidetes	Sphingobacteriia	Sphingobacteriales
Bacteroidetes	Sphingobacteriia	Sphingobacteriales
Bacteroidetes	Sphingobacteriia	Sphingobacteriales
Bacteroidetes	Sphingobacteriia	Sphingobacteriales
Bacteroidetes	Sphingobacteriia	Sphingobacteriales
Bacteroidetes	Sphingobacteriia	Sphingobacteriales
Bacteroidetes	Sphingobacteriia	Sphingobacteriales
Chlamydiae	Chlamydiia	Chlamydiales
Cyanobacteria	Nostocophycideae	Nostocales
Cyanobacteria	Oscillatoriophycideae	Oscillatoriales

Deinococcus-Thermus	Deinococci	Deinococcales
Fibrobacteres	Fibrobacteria	Fibrobacterales
Firmicutes	Bacilli	Bacillales
Firmicutes	Bacilli	Bacillales
Firmicutes	Bacilli	Bacillales
Firmicutes	Bacilli	Bacillales
Firmicutes	Bacilli	Bacillales
Firmicutes	Bacilli	Bacillales
Firmicutes	Bacilli	Bacillales
Firmicutes	Bacilli	Bacillales
Firmicutes	Bacilli	Bacillales
Firmicutes	Bacilli	Bacillales
Firmicutes	Bacilli	Bacillales
Firmicutes	Bacilli	Bacillales
Firmicutes	Bacilli	Bacillales
Firmicutes	Bacilli	Bacillales
Firmicutes	Bacilli	Bacillales
Firmicutes	Bacilli	Bacillales
Firmicutes	Bacilli	Bacillales
Firmicutes	Bacilli	Bacillales
Firmicutes	Bacilli	Bacillales
Firmicutes	Bacilli	Bacillales
Firmicutes	Bacilli	Bacillales
Firmicutes	Bacilli	Bacillales
Firmicutes	Bacilli	Bacillales
Firmicutes	Bacilli	Bacillales
Firmicutes	Bacilli	Bacillales
Firmicutes	Bacilli	Bacillales
Firmicutes	Bacilli	Bacillales
Firmicutes	Bacilli	Bacillales
Firmicutes	Bacilli	Bacillales
Firmicutes	Bacilli	Bacillales
Firmicutes	Bacilli	Bacillales
Firmicutes	Bacilli	Bacillales
Firmicutes	Bacilli	Bacillales
Firmicutes	Bacilli	Bacillales
Firmicutes	Bacilli	Bacillales
Firmicutes	Bacilli	Bacillales
Firmicutes	Bacilli	Bacillales
Firmicutes	Bacilli	Bacillales
Firmicutes	Bacilli	Bacillales
Firmicutes	Bacilli	Bacillales
Firmicutes	Bacilli	Bacillales
Firmicutes	Bacilli	Bacillales
Firmicutes	Bacilli	Bacillales
Firmicutes	Bacilli	Bacillales

Firmicutes	Bacilli	Bacillales
Firmicutes	Bacilli	Bacillales
Firmicutes	Bacilli	Bacillales
Firmicutes	Bacilli	Bacillales
Firmicutes	Bacilli	Bacillales
Firmicutes	Bacilli	Lactobacillales
Firmicutes	Bacilli	Lactobacillales
Firmicutes	Bacilli	Lactobacillales
Firmicutes	Bacilli	Lactobacillales
Firmicutes	Bacilli	Lactobacillales
Firmicutes	Bacilli	Lactobacillales
Firmicutes	Bacilli	Lactobacillales
Firmicutes	Bacilli	Lactobacillales
Firmicutes	Bacilli	Lactobacillales
Firmicutes	Bacilli	Lactobacillales
Firmicutes	Bacilli	Lactobacillales
Firmicutes	Bacilli	Lactobacillales
Firmicutes	Bacilli	Lactobacillales
Firmicutes	Bacilli	Lactobacillales
Firmicutes	Bacilli	Lactobacillales
Firmicutes	Bacilli	Lactobacillales
Firmicutes	Bacilli	Lactobacillales
Firmicutes	Bacilli	Lactobacillales
Firmicutes	Bacilli	Lactobacillales
Firmicutes	Bacilli	Lactobacillales
Firmicutes	Clostridia	Clostridiales
Firmicutes	Clostridia	Clostridiales
Firmicutes	Clostridia	Clostridiales
Firmicutes	Clostridia	Clostridiales
Firmicutes	Clostridia	Clostridiales
Firmicutes	Clostridia	Clostridiales
Firmicutes	Clostridia	Clostridiales
Firmicutes	Clostridia	Clostridiales
Firmicutes	Clostridia	Clostridiales
Firmicutes	Clostridia	Clostridiales
Firmicutes	Clostridia	Clostridiales
Firmicutes	Clostridia	Clostridiales
Firmicutes	Clostridia	Clostridiales
Firmicutes	Clostridia	Clostridiales
Firmicutes	Clostridia	Clostridiales
Firmicutes	Clostridia	Clostridiales
Firmicutes	Clostridia	Clostridiales
Firmicutes	Clostridia	Clostridiales
Firmicutes	Clostridia	Clostridiales
Firmicutes	Clostridia	Clostridiales
Firmicutes	Clostridia	Clostridiales

Firmicutes	Clostridia	Clostridiales
Firmicutes	Clostridia	Clostridiales
Firmicutes	Clostridia	Clostridiales
Firmicutes	Clostridia	Clostridiales
Firmicutes	Erysipelotrichi	Erysipelotrichales
Firmicutes	Negativicutes	Selenomonadales
Nitrospirae	Nitrospira	Nitrospirales
Proteobacteria	Alphaproteobacteria	Magnetococcales
Proteobacteria	Alphaproteobacteria	Rhizobiales
Proteobacteria	Alphaproteobacteria	Rhizobiales
Proteobacteria	Alphaproteobacteria	Rhizobiales
Proteobacteria	Alphaproteobacteria	Rhizobiales
Proteobacteria	Alphaproteobacteria	Rhizobiales
Proteobacteria	Alphaproteobacteria	Rhizobiales
Proteobacteria	Alphaproteobacteria	Rhizobiales
Proteobacteria	Alphaproteobacteria	Rhizobiales
Proteobacteria	Alphaproteobacteria	Rhizobiales
Proteobacteria	Alphaproteobacteria	Rhizobiales
Proteobacteria	Alphaproteobacteria	Rhizobiales
Proteobacteria	Alphaproteobacteria	Rhizobiales
Proteobacteria	Alphaproteobacteria	Rhizobiales
Proteobacteria	Alphaproteobacteria	Rhizobiales
Proteobacteria	Alphaproteobacteria	Rhodobacterales
Proteobacteria	Alphaproteobacteria	Rhodobacterales
Proteobacteria	Alphaproteobacteria	Rhodobacterales
Proteobacteria	Alphaproteobacteria	Rhodobacterales
Proteobacteria	Alphaproteobacteria	Rhodobacterales
Proteobacteria	Alphaproteobacteria	Rhodobacterales
Proteobacteria	Alphaproteobacteria	Sphingomonadales
Proteobacteria	Alphaproteobacteria	Sphingomonadales
Proteobacteria	Alphaproteobacteria	Sphingomonadales
Proteobacteria	Alphaproteobacteria	Sphingomonadales
Proteobacteria	Alphaproteobacteria	Sphingomonadales
Proteobacteria	Alphaproteobacteria	Sphingomonadales
Proteobacteria	Betaproteobacteria	Burkholderiales
Proteobacteria	Betaproteobacteria	Burkholderiales
Proteobacteria	Betaproteobacteria	Burkholderiales
Proteobacteria	Betaproteobacteria	Burkholderiales
Proteobacteria	Betaproteobacteria	Burkholderiales
Proteobacteria	Betaproteobacteria	Burkholderiales
Proteobacteria	Betaproteobacteria	Burkholderiales
Proteobacteria	Betaproteobacteria	Burkholderiales
Proteobacteria	Betaproteobacteria	Burkholderiales
Proteobacteria	Betaproteobacteria	Burkholderiales
Proteobacteria	Betaproteobacteria	Burkholderiales
Proteobacteria	Betaproteobacteria	Burkholderiales

Proteobacteria	Betaproteobacteria	Burkholderiales
Proteobacteria	Betaproteobacteria	Burkholderiales
Proteobacteria	Betaproteobacteria	Burkholderiales
Proteobacteria	Betaproteobacteria	Burkholderiales
Proteobacteria	Betaproteobacteria	Burkholderiales
Proteobacteria	Betaproteobacteria	Burkholderiales
Proteobacteria	Betaproteobacteria	Burkholderiales
Proteobacteria	Betaproteobacteria	Burkholderiales
Proteobacteria	Betaproteobacteria	Burkholderiales
Proteobacteria	Betaproteobacteria	Burkholderiales
Proteobacteria	Betaproteobacteria	Burkholderiales
Proteobacteria	Betaproteobacteria	Burkholderiales
Proteobacteria	Betaproteobacteria	Burkholderiales
Proteobacteria	Betaproteobacteria	Burkholderiales
Proteobacteria	Betaproteobacteria	Burkholderiales
Proteobacteria	Betaproteobacteria	Burkholderiales
Proteobacteria	Betaproteobacteria	Burkholderiales
Proteobacteria	Betaproteobacteria	Hydrogenophilales
Proteobacteria	Betaproteobacteria	Neisseriales
Proteobacteria	Betaproteobacteria	Neisseriales
Proteobacteria	Betaproteobacteria	Rhodocyclales
Proteobacteria	Betaproteobacteria	Rhodocyclales
Proteobacteria	Betaproteobacteria	Rhodocyclales
Proteobacteria	Deltaproteobacteria	Desulfobacterales
Proteobacteria	Deltaproteobacteria	Desulfovibrionales
Proteobacteria	Deltaproteobacteria	Desulfuromonadales
Proteobacteria	Deltaproteobacteria	Desulfuromonadales
Proteobacteria	Deltaproteobacteria	Syntrophobacterales
Proteobacteria	Deltaproteobacteria	Syntrophobacterales
Proteobacteria	Epsilonproteobacteria	Campylobacterales
Proteobacteria	Epsilonproteobacteria	Campylobacterales
Proteobacteria	Epsilonproteobacteria	Campylobacterales
Proteobacteria	Epsilonproteobacteria	Campylobacterales
Proteobacteria	Epsilonproteobacteria	Campylobacterales
Proteobacteria	Epsilonproteobacteria	Campylobacterales
Proteobacteria	Gammaproteobacteria	Aeromonadales
Proteobacteria	Gammaproteobacteria	Aeromonadales
Proteobacteria	Gammaproteobacteria	Alteromonadales
Proteobacteria	Gammaproteobacteria	Alteromonadales
Proteobacteria	Gammaproteobacteria	Alteromonadales
Proteobacteria	Gammaproteobacteria	Alteromonadales
Proteobacteria	Gammaproteobacteria	Alteromonadales
Proteobacteria	Gammaproteobacteria	Alteromonadales
Proteobacteria	Gammaproteobacteria	Chromatiales
Proteobacteria	Gammaproteobacteria	Chromatiales
Proteobacteria	Gammaproteobacteria	Enterobacteriales

Proteobacteria	Gammaproteobacteria	Enterobacteriales
Proteobacteria	Gammaproteobacteria	Enterobacteriales
Proteobacteria	Gammaproteobacteria	Enterobacteriales
Proteobacteria	Gammaproteobacteria	Enterobacteriales
Proteobacteria	Gammaproteobacteria	Enterobacteriales
Proteobacteria	Gammaproteobacteria	Enterobacteriales
Proteobacteria	Gammaproteobacteria	Enterobacteriales
Proteobacteria	Gammaproteobacteria	Enterobacteriales
Proteobacteria	Gammaproteobacteria	Enterobacteriales
Proteobacteria	Gammaproteobacteria	Enterobacteriales
Proteobacteria	Gammaproteobacteria	Enterobacteriales
Proteobacteria	Gammaproteobacteria	Enterobacteriales
Proteobacteria	Gammaproteobacteria	Legionellales
Proteobacteria	Gammaproteobacteria	Legionellales
Proteobacteria	Gammaproteobacteria	Methylococcales
Proteobacteria	Gammaproteobacteria	Oceanospirillales
Proteobacteria	Gammaproteobacteria	Oceanospirillales
Proteobacteria	Gammaproteobacteria	Oceanospirillales
Proteobacteria	Gammaproteobacteria	Oceanospirillales
Proteobacteria	Gammaproteobacteria	Oceanospirillales
Proteobacteria	Gammaproteobacteria	Oceanospirillales
Proteobacteria	Gammaproteobacteria	Orbales
Proteobacteria	Gammaproteobacteria	Pasteurellales
Proteobacteria	Gammaproteobacteria	Pasteurellales
Proteobacteria	Gammaproteobacteria	Pasteurellales
Proteobacteria	Gammaproteobacteria	Pasteurellales
Proteobacteria	Gammaproteobacteria	Pasteurellales
Proteobacteria	Gammaproteobacteria	Pseudomonadales
Proteobacteria	Gammaproteobacteria	Pseudomonadales
Proteobacteria	Gammaproteobacteria	Pseudomonadales
Proteobacteria	Gammaproteobacteria	Pseudomonadales
Proteobacteria	Gammaproteobacteria	Pseudomonadales
Proteobacteria	Gammaproteobacteria	Pseudomonadales
Proteobacteria	Gammaproteobacteria	Pseudomonadales
Proteobacteria	Gammaproteobacteria	Pseudomonadales
Proteobacteria	Gammaproteobacteria	Pseudomonadales
Proteobacteria	Gammaproteobacteria	Pseudomonadales
Proteobacteria	Gammaproteobacteria	Pseudomonadales
Proteobacteria	Gammaproteobacteria	Pseudomonadales
Proteobacteria	Gammaproteobacteria	Pseudomonadales
Proteobacteria	Gammaproteobacteria	Pseudomonadales
Proteobacteria	Gammaproteobacteria	Pseudomonadales
Proteobacteria	Gammaproteobacteria	Pseudomonadales
Proteobacteria	Gammaproteobacteria	Pseudomonadales
Proteobacteria	Gammaproteobacteria	Pseudomonadales
Proteobacteria	Gammaproteobacteria	Pseudomonadales

Proteobacteria	Gammaproteobacteria	Pseudomonadales
Proteobacteria	Gammaproteobacteria	Pseudomonadales
Proteobacteria	Gammaproteobacteria	Pseudomonadales
Proteobacteria	Gammaproteobacteria	Pseudomonadales
Proteobacteria	Gammaproteobacteria	Pseudomonadales
Proteobacteria	Gammaproteobacteria	Pseudomonadales
Proteobacteria	Gammaproteobacteria	Pseudomonadales
Proteobacteria	Gammaproteobacteria	Pseudomonadales
Proteobacteria	Gammaproteobacteria	Pseudomonadales
Proteobacteria	Gammaproteobacteria	Thiotrichales
Proteobacteria	Gammaproteobacteria	Thiotrichales
Proteobacteria	Gammaproteobacteria	Thiotrichales
Proteobacteria	Gammaproteobacteria	Vibrionales
Proteobacteria	Gammaproteobacteria	Vibrionales
Proteobacteria	Gammaproteobacteria	Xanthomonadales
Proteobacteria	Gammaproteobacteria	Xanthomonadales
Spirochaetes	Spirochaetia	Spirochaetales
Tenericutes	Mollicutes	Acholeplasmatales
Actinobacteria	Actinobacteria	Actinomycetales
Actinobacteria	Actinobacteria	Actinomycetales
Actinobacteria	Actinobacteria	Actinomycetales
Actinobacteria	Actinobacteria	Actinomycetales
Actinobacteria	Actinobacteria	Actinomycetales
Actinobacteria	Actinobacteria	Actinomycetales
Actinobacteria	Actinobacteria	Actinomycetales
Actinobacteria	Actinobacteria	Actinomycetales
Actinobacteria	Actinobacteria	Actinomycetales
Actinobacteria	Actinobacteria	Actinomycetales
Actinobacteria	Actinobacteria	Actinomycetales
Actinobacteria	Actinobacteria	Actinomycetales
Firmicutes	Bacilli	Bacillales
Firmicutes	Bacilli	Bacillales
Firmicutes	Bacilli	Bacillales
Firmicutes	Bacilli	Lactobacillales
Firmicutes	Bacilli	Lactobacillales
Firmicutes	Clostridia	Clostridiales
Firmicutes	Clostridia	Clostridiales
Firmicutes	Clostridia	Clostridiales
Proteobacteria	Alphaproteobacteria	Rhodobacterales
Proteobacteria	Alphaproteobacteria	Rhodobacterales
Proteobacteria	Alphaproteobacteria	Rhodobacterales
Proteobacteria	Alphaproteobacteria	Rhodobacterales
Proteobacteria	Alphaproteobacteria	Rhodobacterales
Proteobacteria	Alphaproteobacteria	Rhodobacterales
Proteobacteria	Alphaproteobacteria	Sphingomonadales
Proteobacteria	Betaproteobacteria	Burkholderiales

Proteobacteria	Betaproteobacteria	Burkholderiales
Proteobacteria	Betaproteobacteria	Burkholderiales
Proteobacteria	Gammaproteobacteria	Acidithiobacillales
Proteobacteria	Gammaproteobacteria	Aeromonadales
Proteobacteria	Gammaproteobacteria	Alteromonadales
Proteobacteria	Gammaproteobacteria	Alteromonadales
Proteobacteria	Gammaproteobacteria	Alteromonadales
Proteobacteria	Gammaproteobacteria	Alteromonadales
Proteobacteria	Gammaproteobacteria	Chromatiales
Proteobacteria	Gammaproteobacteria	Enterobacteriales
Proteobacteria	Gammaproteobacteria	Oceanospirillales
Proteobacteria	Gammaproteobacteria	Pseudomonadales
Proteobacteria	Gammaproteobacteria	unclassifiedGammaproteobacteria
Actinobacteria	Actinobacteria	Actinomycetales
Actinobacteria	Actinobacteria	Actinomycetales
Actinobacteria	Actinobacteria	Actinomycetales
Actinobacteria	Actinobacteria	Actinomycetales
Actinobacteria	Actinobacteria	Actinomycetales
Actinobacteria	Actinobacteria	Actinomycetales
Actinobacteria	Actinobacteria	Actinomycetales
Actinobacteria	Actinobacteria	Actinomycetales
Actinobacteria	Actinobacteria	Actinomycetales
Actinobacteria	Actinobacteria	Actinomycetales
Actinobacteria	Actinobacteria	Actinomycetales
Actinobacteria	Actinobacteria	Actinomycetales
Actinobacteria	Actinobacteria	Actinomycetales
Actinobacteria	Actinobacteria	Actinomycetales
Actinobacteria	Actinobacteria	Actinomycetales
Actinobacteria	Actinobacteria	Actinomycetales
Actinobacteria	Actinobacteria	Actinomycetales
Actinobacteria	Actinobacteria	Actinomycetales
Actinobacteria	Actinobacteria	Actinomycetales
Actinobacteria	Actinobacteria	Actinomycetales
Actinobacteria	Actinobacteria	Actinomycetales
Actinobacteria	Actinobacteria	Actinomycetales
Actinobacteria	Actinobacteria	Actinomycetales
Actinobacteria	Actinobacteria	Actinomycetales
Actinobacteria	Actinobacteria	Actinomycetales
Actinobacteria	Actinobacteria	Actinomycetales
Actinobacteria	Actinobacteria	Actinomycetales
Actinobacteria	Actinobacteria	Actinomycetales
Actinobacteria	Actinobacteria	Actinomycetales
Actinobacteria	Actinobacteria	Actinomycetales
Actinobacteria	Actinobacteria	Actinomycetales
Actinobacteria	Actinobacteria	Actinomycetales
Bacteroidetes	Cytophagia	Cytophagales

Bacteroidetes	Flavobacteriia	Flavobacteriales
Bacteroidetes	Flavobacteriia	Flavobacteriales
Bacteroidetes	Flavobacteriia	Flavobacteriales
Bacteroidetes	Flavobacteriia	Flavobacteriales
Bacteroidetes	Flavobacteriia	Flavobacteriales
Bacteroidetes	Sphingobacteriia	Sphingobacteriales
Firmicutes	Bacilli	Bacillales
Firmicutes	Bacilli	Bacillales
Firmicutes	Bacilli	Bacillales
Firmicutes	Bacilli	Bacillales
Firmicutes	Bacilli	Lactobacillales
Firmicutes	Bacilli	Lactobacillales
Firmicutes	Bacilli	Lactobacillales
Firmicutes	Bacilli	Lactobacillales
Firmicutes	Bacilli	Lactobacillales
Firmicutes	Bacilli	Lactobacillales
Firmicutes	Bacilli	Lactobacillales
Firmicutes	Clostridia	Clostridiales
Firmicutes	Clostridia	Clostridiales
Firmicutes	Clostridia	Clostridiales
Fusobacteria	Fusobacteriia	Fusobacteriales
Proteobacteria	Alphaproteobacteria	Rhodobacterales
Proteobacteria	Alphaproteobacteria	Rhodobacterales
Proteobacteria	Alphaproteobacteria	Rhodobacterales
Proteobacteria	Alphaproteobacteria	Sphingomonadales
Proteobacteria	Alphaproteobacteria	Sphingomonadales
Proteobacteria	Epsilonproteobacteria	Campylobacterales
Proteobacteria	Gammaproteobacteria	Pseudomonadales
Proteobacteria	Gammaproteobacteria	Pseudomonadales
Proteobacteria	Gammaproteobacteria	Thiotrichales
Proteobacteria	Gammaproteobacteria	Thiotrichales
Proteobacteria	Gammaproteobacteria	Thiotrichales
Proteobacteria	Gammaproteobacteria	Xanthomonadales
Proteobacteria	Gammaproteobacteria	unclassifiedGammaproteobacteria
Synergistetes	Synergistia	Synergistales
Synergistetes	Synergistia	Synergistales
Synergistetes	Synergistia	Synergistales
Actinobacteria	Actinobacteria	Actinomycetales
Actinobacteria	Actinobacteria	Actinomycetales
Actinobacteria	Actinobacteria	Actinomycetales
Actinobacteria	Actinobacteria	Actinomycetales
Actinobacteria	Actinobacteria	Actinomycetales
Actinobacteria	Actinobacteria	Actinomycetales
Actinobacteria	Actinobacteria	Actinomycetales
Actinobacteria	Actinobacteria	Actinomycetales
Actinobacteria	Actinobacteria	Actinomycetales

Actinobacteria	Actinobacteria	Actinomycetales
Actinobacteria	Actinobacteria	Actinomycetales
Actinobacteria	Actinobacteria	Actinomycetales
Actinobacteria	Actinobacteria	Actinomycetales
Actinobacteria	Actinobacteria	Actinomycetales
Actinobacteria	Actinobacteria	Actinomycetales
Actinobacteria	Actinobacteria	Actinomycetales
Actinobacteria	Actinobacteria	Actinomycetales
Actinobacteria	Actinobacteria	Actinomycetales
Actinobacteria	Actinobacteria	Actinomycetales
Actinobacteria	Actinobacteria	Actinomycetales
Actinobacteria	Actinobacteria	Actinomycetales
Actinobacteria	Actinobacteria	Actinomycetales
Actinobacteria	Actinobacteria	Actinomycetales
Actinobacteria	Actinobacteria	Actinomycetales
Actinobacteria	Actinobacteria	Solirubrobacterales
Actinobacteria	Actinobacteria	Solirubrobacterales
Bacteroidetes	Bacteroidia	Bacteroidales
Bacteroidetes	Bacteroidia	Bacteroidales
Bacteroidetes	Sphingobacteriia	Sphingobacteriales
Firmicutes	Bacilli	Bacillales
Firmicutes	Bacilli	Bacillales
Firmicutes	Bacilli	Bacillales
Firmicutes	Bacilli	Bacillales
Firmicutes	Bacilli	Bacillales
Firmicutes	Bacilli	Bacillales
Firmicutes	Bacilli	Bacillales
Firmicutes	Bacilli	Bacillales
Firmicutes	Bacilli	Bacillales
Firmicutes	Bacilli	Bacillales
Firmicutes	Bacilli	Bacillales
Firmicutes	Bacilli	Bacillales
Firmicutes	Bacilli	Bacillales
Firmicutes	Bacilli	Bacillales
Firmicutes	Bacilli	Bacillales
Firmicutes	Bacilli	Bacillales
Firmicutes	Bacilli	Lactobacillales
Firmicutes	Bacilli	Lactobacillales
Firmicutes	Bacilli	Lactobacillales
Firmicutes	Bacilli	Lactobacillales
Firmicutes	Bacilli	Lactobacillales
Firmicutes	Bacilli	Lactobacillales
Firmicutes	Bacilli	Lactobacillales
Firmicutes	Bacilli	Lactobacillales
Firmicutes	Bacilli	Lactobacillales
Firmicutes	Bacilli	Lactobacillales

Firmicutes	Bacilli	Lactobacillales
Firmicutes	Bacilli	Lactobacillales
Firmicutes	Bacilli	Lactobacillales
Firmicutes	Clostridia	Clostridiales
Firmicutes	Clostridia	Clostridiales
Firmicutes	Clostridia	Clostridiales
Firmicutes	Clostridia	Clostridiales
Firmicutes	Clostridia	Clostridiales
Firmicutes	Clostridia	Clostridiales
Firmicutes	Clostridia	Clostridiales
Firmicutes	Clostridia	Clostridiales
Firmicutes	Clostridia	Clostridiales
Firmicutes	Clostridia	Clostridiales
Firmicutes	Clostridia	Clostridiales
Firmicutes	Clostridia	Clostridiales
Firmicutes	Clostridia	Clostridiales
Firmicutes	Negativicutes	Selenomonadales
Firmicutes	Negativicutes	Selenomonadales
Firmicutes	Negativicutes	Selenomonadales
Firmicutes	Negativicutes	Selenomonadales
Firmicutes	Negativicutes	Selenomonadales
Firmicutes	Negativicutes	Selenomonadales
Firmicutes	Negativicutes	Selenomonadales
Proteobacteria	Alphaproteobacteria	Caulobacterales
Proteobacteria	Alphaproteobacteria	Caulobacterales
Proteobacteria	Alphaproteobacteria	Caulobacterales
Proteobacteria	Alphaproteobacteria	Rhizobiales
Proteobacteria	Alphaproteobacteria	Rhizobiales
Proteobacteria	Alphaproteobacteria	Rhodobacterales
Proteobacteria	Betaproteobacteria	Burkholderiales
Proteobacteria	Betaproteobacteria	Burkholderiales
Proteobacteria	Betaproteobacteria	Burkholderiales
Proteobacteria	Betaproteobacteria	Burkholderiales
Proteobacteria	Betaproteobacteria	Hydrogenophilales
Proteobacteria	Betaproteobacteria	Neisseriales
Proteobacteria	Betaproteobacteria	Neisseriales
Proteobacteria	Epsilonproteobacteria	Campylobacterales
Proteobacteria	Gammaproteobacteria	Pasteurellales
Proteobacteria	Gammaproteobacteria	Pseudomonadales
Proteobacteria	Gammaproteobacteria	Pseudomonadales
Proteobacteria	Gammaproteobacteria	Pseudomonadales
Proteobacteria	Gammaproteobacteria	Vibrionales
Proteobacteria	Gammaproteobacteria	Xanthomonadales
Acidobacteria	Holophagae	Holophagales
Actinobacteria	Actinobacteria	Actinomycetales
Actinobacteria	Actinobacteria	Actinomycetales

Actinobacteria	Actinobacteria	Actinomycetales
Actinobacteria	Actinobacteria	Actinomycetales
Actinobacteria	Actinobacteria	Actinomycetales
Actinobacteria	Actinobacteria	Actinomycetales
Actinobacteria	Actinobacteria	Actinomycetales
Actinobacteria	Actinobacteria	Actinomycetales
Actinobacteria	Actinobacteria	Actinomycetales
Actinobacteria	Actinobacteria	Actinomycetales
Actinobacteria	Actinobacteria	Actinomycetales
Actinobacteria	Actinobacteria	Actinomycetales
Actinobacteria	Actinobacteria	Actinomycetales
Actinobacteria	Actinobacteria	Actinomycetales
Actinobacteria	Actinobacteria	Actinomycetales
Actinobacteria	Actinobacteria	Actinomycetales
Actinobacteria	Actinobacteria	Actinomycetales
Actinobacteria	Actinobacteria	Actinomycetales
Actinobacteria	Actinobacteria	Actinomycetales
Actinobacteria	Actinobacteria	Actinomycetales
Actinobacteria	Actinobacteria	Actinomycetales
Actinobacteria	Actinobacteria	Actinomycetales
Actinobacteria	Actinobacteria	Actinomycetales
Actinobacteria	Actinobacteria	Actinomycetales
Actinobacteria	Actinobacteria	Actinomycetales
Actinobacteria	Actinobacteria	Actinomycetales
Actinobacteria	Actinobacteria	Actinomycetales
Actinobacteria	Actinobacteria	Actinomycetales
Actinobacteria	Actinobacteria	Actinomycetales
Actinobacteria	Actinobacteria	Actinomycetales
Actinobacteria	Actinobacteria	Actinomycetales
Actinobacteria	Actinobacteria	Actinomycetales
Actinobacteria	Actinobacteria	Actinomycetales
Actinobacteria	Actinobacteria	Actinomycetales
Actinobacteria	Actinobacteria	Actinomycetales
Actinobacteria	Actinobacteria	Actinomycetales
Actinobacteria	Actinobacteria	Actinomycetales
Actinobacteria	Actinobacteria	Actinomycetales
Actinobacteria	Actinobacteria	Gaiellales
Bacteroidetes	Bacteroidia	Bacteroidales
Bacteroidetes	Bacteroidia	Bacteroidales
Bacteroidetes	Cytophagia	Cytophagales
Bacteroidetes	Cytophagia	Cytophagales
Bacteroidetes	Cytophagia	Cytophagales
Bacteroidetes	Flavobacteriia	Flavobacteriales
Bacteroidetes	Flavobacteriia	Flavobacteriales
Bacteroidetes	Flavobacteriia	Flavobacteriales
Bacteroidetes	Flavobacteriia	Flavobacteriales

Bacteroidetes	Flavobacteriia	Flavobacteriales
Bacteroidetes	Sphingobacteriia	Sphingobacteriales
Bacteroidetes	Sphingobacteriia	Sphingobacteriales
Chloroflexi	Dehalococcoidia	Dehalococcoidales
Chloroflexi	Dehalococcoidia	unclassifiedDehalococcoidia
Fibrobacteres	Fibrobacteria	Fibrobacterales
Fibrobacteres	Fibrobacteria	Fibrobacterales
Fibrobacteres	Fibrobacteria	Fibrobacterales
Firmicutes	Bacilli	Bacillales
Firmicutes	Bacilli	Bacillales
Firmicutes	Bacilli	Bacillales
Firmicutes	Bacilli	Bacillales
Firmicutes	Bacilli	Bacillales
Firmicutes	Bacilli	Bacillales
Firmicutes	Bacilli	Bacillales
Firmicutes	Bacilli	Bacillales
Firmicutes	Bacilli	Bacillales
Firmicutes	Bacilli	Bacillales
Firmicutes	Bacilli	Bacillales
Firmicutes	Bacilli	Bacillales
Firmicutes	Bacilli	Bacillales
Firmicutes	Bacilli	Bacillales
Firmicutes	Bacilli	Bacillales
Firmicutes	Bacilli	Bacillales
Firmicutes	Bacilli	Lactobacillales
Firmicutes	Bacilli	Lactobacillales
Firmicutes	Bacilli	Lactobacillales
Firmicutes	Bacilli	Lactobacillales
Firmicutes	Bacilli	Lactobacillales
Firmicutes	Bacilli	Lactobacillales
Firmicutes	Bacilli	Lactobacillales
Firmicutes	Bacilli	Lactobacillales
Firmicutes	Bacilli	Lactobacillales
Firmicutes	Clostridia	Clostridiales
Firmicutes	Clostridia	Clostridiales
Firmicutes	Clostridia	Clostridiales
Firmicutes	Clostridia	Clostridiales
Firmicutes	Clostridia	Clostridiales
Firmicutes	Clostridia	Clostridiales
Firmicutes	Clostridia	Clostridiales
Firmicutes	Clostridia	Clostridiales
Firmicutes	Clostridia	Clostridiales
Firmicutes	Clostridia	Clostridiales
Firmicutes	Clostridia	Clostridiales
Firmicutes	Clostridia	Clostridiales
Firmicutes	Clostridia	Clostridiales

Fusobacteria	Fusobacteriia	Fusobacteriales
Proteobacteria	Alphaproteobacteria	Rhizobiales
Proteobacteria	Alphaproteobacteria	Rhizobiales
Proteobacteria	Alphaproteobacteria	Rhizobiales
Proteobacteria	Alphaproteobacteria	Rhizobiales
Proteobacteria	Alphaproteobacteria	Rhizobiales
Proteobacteria	Alphaproteobacteria	Rhizobiales
Proteobacteria	Alphaproteobacteria	Rhizobiales
Proteobacteria	Alphaproteobacteria	Rhizobiales
Proteobacteria	Alphaproteobacteria	Rhodobacterales
Proteobacteria	Alphaproteobacteria	Rhodobacterales
Proteobacteria	Alphaproteobacteria	Sphingomonadales
Proteobacteria	Alphaproteobacteria	Sphingomonadales
Proteobacteria	Alphaproteobacteria	Sphingomonadales
Proteobacteria	Alphaproteobacteria	Sphingomonadales
Proteobacteria	Alphaproteobacteria	Sphingomonadales
Proteobacteria	Betaproteobacteria	Burkholderiales
Proteobacteria	Betaproteobacteria	Burkholderiales
Proteobacteria	Betaproteobacteria	Burkholderiales
Proteobacteria	Betaproteobacteria	Burkholderiales
Proteobacteria	Betaproteobacteria	Burkholderiales
Proteobacteria	Betaproteobacteria	Burkholderiales
Proteobacteria	Betaproteobacteria	Burkholderiales
Proteobacteria	Betaproteobacteria	Neisseriales
Proteobacteria	Betaproteobacteria	Nitrosomonadales
Proteobacteria	Deltaproteobacteria	Myxococcales
Proteobacteria	Gammaproteobacteria	Aeromonadales
Proteobacteria	Gammaproteobacteria	Alteromonadales
Proteobacteria	Gammaproteobacteria	Alteromonadales
Proteobacteria	Gammaproteobacteria	Alteromonadales
Proteobacteria	Gammaproteobacteria	Alteromonadales
Proteobacteria	Gammaproteobacteria	Alteromonadales
Proteobacteria	Gammaproteobacteria	Arenicellales
Proteobacteria	Gammaproteobacteria	Chromatiales
Proteobacteria	Gammaproteobacteria	Chromatiales
Proteobacteria	Gammaproteobacteria	Chromatiales
Proteobacteria	Gammaproteobacteria	Chromatiales
Proteobacteria	Gammaproteobacteria	Chromatiales
Proteobacteria	Gammaproteobacteria	Chromatiales
Proteobacteria	Gammaproteobacteria	Enterobacteriales
Proteobacteria	Gammaproteobacteria	Enterobacteriales
Proteobacteria	Gammaproteobacteria	Legionellales
Proteobacteria	Gammaproteobacteria	Oceanospirillales
Proteobacteria	Gammaproteobacteria	Oceanospirillales
Proteobacteria	Gammaproteobacteria	Oceanospirillales
Proteobacteria	Gammaproteobacteria	Pseudomonadales

Proteobacteria	Gammaproteobacteria	Thiotrichales
Proteobacteria	Gammaproteobacteria	Xanthomonadales
Spirochaetes	Spirochaetia	Spirochaetales
Actinobacteria	Actinobacteria	Acidimicrobiales
Actinobacteria	Actinobacteria	Acidimicrobiales
Actinobacteria	Actinobacteria	Actinomycetales
Actinobacteria	Actinobacteria	Actinomycetales
Actinobacteria	Actinobacteria	Actinomycetales
Actinobacteria	Actinobacteria	Actinomycetales
Actinobacteria	Actinobacteria	Actinomycetales
Actinobacteria	Actinobacteria	Actinomycetales
Actinobacteria	Actinobacteria	Actinomycetales
Actinobacteria	Actinobacteria	Actinomycetales
Actinobacteria	Actinobacteria	Actinomycetales
Actinobacteria	Actinobacteria	Actinomycetales
Actinobacteria	Actinobacteria	Actinomycetales
Actinobacteria	Actinobacteria	Actinomycetales
Actinobacteria	Actinobacteria	Actinomycetales
Actinobacteria	Actinobacteria	Actinomycetales
Actinobacteria	Actinobacteria	Actinomycetales
Actinobacteria	Actinobacteria	Actinomycetales
Actinobacteria	Actinobacteria	Actinomycetales
Actinobacteria	Actinobacteria	Actinomycetales
Actinobacteria	Actinobacteria	Actinomycetales
Actinobacteria	Actinobacteria	Actinomycetales
Actinobacteria	Actinobacteria	Actinomycetales
Actinobacteria	Actinobacteria	Actinomycetales
Actinobacteria	Actinobacteria	Actinomycetales
Actinobacteria	Actinobacteria	Actinomycetales
Actinobacteria	Actinobacteria	Actinomycetales
Actinobacteria	Actinobacteria	Actinomycetales
Actinobacteria	Actinobacteria	Actinomycetales
Actinobacteria	Actinobacteria	Actinomycetales
Actinobacteria	Actinobacteria	Actinomycetales
Actinobacteria	Actinobacteria	Actinomycetales
Actinobacteria	Actinobacteria	Actinomycetales
Actinobacteria	Actinobacteria	Actinomycetales
Actinobacteria	Actinobacteria	Actinomycetales
Actinobacteria	Actinobacteria	Actinomycetales
Actinobacteria	Actinobacteria	Actinomycetales
Actinobacteria	Actinobacteria	Actinomycetales
Actinobacteria	Actinobacteria	Actinomycetales
Actinobacteria	Actinobacteria	Actinomycetales
Actinobacteria	Actinobacteria	Actinomycetales
Actinobacteria	Actinobacteria	Actinomycetales
Actinobacteria	Actinobacteria	Actinomycetales

Actinobacteria	Actinobacteria	Actinomycetales
Actinobacteria	Actinobacteria	Gaiellales
Actinobacteria	Actinobacteria	Rubrobacterales
Actinobacteria	Actinobacteria	Solirubrobacterales
Bacteroidetes	Bacteroidia	Bacteroidales
Bacteroidetes	Bacteroidia	Bacteroidales
Bacteroidetes	Flavobacteriia	Flavobacteriales
Bacteroidetes	Flavobacteriia	Flavobacteriales
Chlamydiae	Chlamydiia	Chlamydiales
Chloroflexi	Chloroflexia	Herpetosiphonales
Firmicutes	Bacilli	Bacillales
Firmicutes	Bacilli	Bacillales
Firmicutes	Bacilli	Bacillales
Firmicutes	Bacilli	Bacillales
Firmicutes	Bacilli	Bacillales
Firmicutes	Bacilli	Bacillales
Firmicutes	Bacilli	Bacillales
Firmicutes	Bacilli	Bacillales
Firmicutes	Bacilli	Bacillales
Firmicutes	Bacilli	Bacillales
Firmicutes	Bacilli	Bacillales
Firmicutes	Bacilli	Bacillales
Firmicutes	Bacilli	Bacillales
Firmicutes	Bacilli	Bacillales
Firmicutes	Bacilli	Bacillales
Firmicutes	Bacilli	Bacillales
Firmicutes	Bacilli	Bacillales
Firmicutes	Bacilli	Bacillales
Firmicutes	Bacilli	Bacillales
Firmicutes	Bacilli	Lactobacillales
Firmicutes	Bacilli	Lactobacillales
Firmicutes	Clostridia	Clostridiales
Firmicutes	Clostridia	Clostridiales
Firmicutes	Clostridia	Clostridiales
Firmicutes	Clostridia	Clostridiales
Firmicutes	Clostridia	Clostridiales
Firmicutes	Clostridia	Clostridiales
Firmicutes	Clostridia	Clostridiales
Nitrospirae	Nitrospira	Nitrospirales
Proteobacteria	Alphaproteobacteria	Rhizobiales
Proteobacteria	Alphaproteobacteria	Rhizobiales
Proteobacteria	Alphaproteobacteria	Rhizobiales
Proteobacteria	Alphaproteobacteria	Rhizobiales
Proteobacteria	Alphaproteobacteria	Rhizobiales
Proteobacteria	Alphaproteobacteria	Rhizobiales
Proteobacteria	Alphaproteobacteria	Rhizobiales

Proteobacteria	Alphaproteobacteria	Rhodobacterales
Proteobacteria	Alphaproteobacteria	Rhodospirillales
Proteobacteria	Alphaproteobacteria	Rickettsiales
Proteobacteria	Alphaproteobacteria	unclassifiedAlphaproteobacteria
Proteobacteria	Betaproteobacteria	Hydrogenophilales
Proteobacteria	Betaproteobacteria	Neisseriales
Proteobacteria	Deltaproteobacteria	Desulfovibrionales
Proteobacteria	Deltaproteobacteria	Myxococcales
Proteobacteria	Gammaproteobacteria	Enterobacteriales
Proteobacteria	Gammaproteobacteria	Enterobacteriales
Proteobacteria	Gammaproteobacteria	Enterobacteriales
Proteobacteria	Gammaproteobacteria	Xanthomonadales
Actinobacteria	Actinobacteria	Actinomycetales
Actinobacteria	Actinobacteria	Actinomycetales
Actinobacteria	Actinobacteria	Actinomycetales
Actinobacteria	Actinobacteria	Actinomycetales
Actinobacteria	Actinobacteria	Actinomycetales
Actinobacteria	Actinobacteria	Actinomycetales
Bacteroidetes	Sphingobacteriia	Sphingobacteriales
Firmicutes	Bacilli	Lactobacillales
Proteobacteria	Deltaproteobacteria	Myxococcales
Proteobacteria	Gammaproteobacteria	Enterobacteriales
Proteobacteria	Gammaproteobacteria	Enterobacteriales
Proteobacteria	Gammaproteobacteria	Pseudomonadales
Proteobacteria	Gammaproteobacteria	Xanthomonadales
Actinobacteria	Actinobacteria	Coriobacteriales
Bacteroidetes	Cytophagia	Cytophagales
Bacteroidetes	Flavobacteriia	Flavobacteriales
Bacteroidetes	Flavobacteriia	Flavobacteriales
Bacteroidetes	Flavobacteriia	Flavobacteriales
Bacteroidetes	Flavobacteriia	Flavobacteriales
Bacteroidetes	Flavobacteriia	Flavobacteriales
Bacteroidetes	Flavobacteriia	Flavobacteriales
Bacteroidetes	Flavobacteriia	Flavobacteriales
Chloroflexi	Ardenticatenia	Ardenticatenales
Firmicutes	Bacilli	Bacillales
Firmicutes	Bacilli	Bacillales
Proteobacteria	Alphaproteobacteria	Rhizobiales
Proteobacteria	Alphaproteobacteria	Rhizobiales
Proteobacteria	Alphaproteobacteria	Rhodobacterales
Proteobacteria	Alphaproteobacteria	Sphingomonadales
Proteobacteria	Alphaproteobacteria	Sphingomonadales
Proteobacteria	Alphaproteobacteria	Sphingomonadales
Proteobacteria	Betaproteobacteria	Burkholderiales
Proteobacteria	Betaproteobacteria	Burkholderiales
Proteobacteria	Betaproteobacteria	Hydrogenophilales

Proteobacteria	Deltaproteobacteria	Myxococcales
Proteobacteria	Gammaproteobacteria	Chromatiales
Proteobacteria	Gammaproteobacteria	Enterobacteriales
Proteobacteria	Gammaproteobacteria	Enterobacteriales
Proteobacteria	Gammaproteobacteria	Enterobacteriales
Proteobacteria	Gammaproteobacteria	Enterobacteriales
Proteobacteria	Gammaproteobacteria	Oceanospirillales
Proteobacteria	Gammaproteobacteria	Pseudomonadales
Proteobacteria	Gammaproteobacteria	Pseudomonadales
Proteobacteria	Gammaproteobacteria	Thiotrichales
Proteobacteria	Gammaproteobacteria	Xanthomonadales
Proteobacteria	Gammaproteobacteria	Xanthomonadales
Verrucomicrobia	Opitutae	Opitutales
Actinobacteria	Actinobacteria	Actinomycetales
Actinobacteria	Actinobacteria	Actinomycetales
Actinobacteria	Actinobacteria	Actinomycetales
Actinobacteria	Actinobacteria	Actinomycetales
Actinobacteria	Actinobacteria	Actinomycetales
Actinobacteria	Actinobacteria	Actinomycetales
Actinobacteria	Actinobacteria	Actinomycetales
Actinobacteria	Actinobacteria	Actinomycetales
Actinobacteria	Actinobacteria	Solirubrobacterales
Bacteroidetes	Cytophagia	Cytophagales
Bacteroidetes	Flavobacteriia	Flavobacteriales
Bacteroidetes	Flavobacteriia	Flavobacteriales
Bacteroidetes	Flavobacteriia	Flavobacteriales
Bacteroidetes	Flavobacteriia	Flavobacteriales
Bacteroidetes	Flavobacteriia	Flavobacteriales
Bacteroidetes	Flavobacteriia	Flavobacteriales
Bacteroidetes	Flavobacteriia	Flavobacteriales
Chlamydiae	Chlamydiia	Chlamydiales
Firmicutes	Bacilli	Bacillales
Firmicutes	Bacilli	Bacillales
Firmicutes	Bacilli	Bacillales
Firmicutes	Bacilli	Lactobacillales
Proteobacteria	Alphaproteobacteria	Rhizobiales
Proteobacteria	Alphaproteobacteria	Rhizobiales
Proteobacteria	Alphaproteobacteria	Rhizobiales
Proteobacteria	Alphaproteobacteria	Rhizobiales
Proteobacteria	Alphaproteobacteria	Rhizobiales
Proteobacteria	Alphaproteobacteria	Sphingomonadales
Proteobacteria	Alphaproteobacteria	Sphingomonadales
Proteobacteria	Alphaproteobacteria	Sphingomonadales
Proteobacteria	Betaproteobacteria	Burkholderiales
Proteobacteria	Betaproteobacteria	Burkholderiales
Proteobacteria	Betaproteobacteria	Burkholderiales

Proteobacteria	Betaproteobacteria	Burkholderiales
Proteobacteria	Betaproteobacteria	Burkholderiales
Proteobacteria	Betaproteobacteria	Burkholderiales
Proteobacteria	Betaproteobacteria	Burkholderiales
Proteobacteria	Betaproteobacteria	Burkholderiales
Proteobacteria	Betaproteobacteria	Burkholderiales
Proteobacteria	Deltaproteobacteria	Bdellovibrionales
Proteobacteria	Deltaproteobacteria	Myxococcales
Proteobacteria	Gammaproteobacteria	Alteromonadales
Proteobacteria	Gammaproteobacteria	Alteromonadales
Proteobacteria	Gammaproteobacteria	Enterobacteriales
Proteobacteria	Gammaproteobacteria	Legionellales
Proteobacteria	Gammaproteobacteria	Oceanospirillales
Proteobacteria	Gammaproteobacteria	Oceanospirillales
Proteobacteria	Gammaproteobacteria	Oceanospirillales
Proteobacteria	Gammaproteobacteria	Pseudomonadales
Proteobacteria	Gammaproteobacteria	Pseudomonadales
Proteobacteria	Gammaproteobacteria	Pseudomonadales
Proteobacteria	Gammaproteobacteria	Pseudomonadales
Proteobacteria	Gammaproteobacteria	Pseudomonadales
Proteobacteria	Gammaproteobacteria	Vibrionales
Proteobacteria	Gammaproteobacteria	Xanthomonadales
Proteobacteria	Gammaproteobacteria	Xanthomonadales
Acidobacteria	Acidobacteria	unclassifiedAcidobacteria
Actinobacteria	Actinobacteria	Acidimicrobiales
Actinobacteria	Actinobacteria	Actinomycetales
Actinobacteria	Actinobacteria	Actinomycetales
Actinobacteria	Actinobacteria	Actinomycetales
Actinobacteria	Actinobacteria	Actinomycetales
Actinobacteria	Actinobacteria	Actinomycetales
Actinobacteria	Actinobacteria	Actinomycetales
Actinobacteria	Actinobacteria	Actinomycetales
Actinobacteria	Actinobacteria	Actinomycetales
Actinobacteria	Actinobacteria	Actinomycetales
Actinobacteria	Actinobacteria	Actinomycetales
Actinobacteria	Actinobacteria	Actinomycetales
Actinobacteria	Actinobacteria	Actinomycetales
Actinobacteria	Actinobacteria	Actinomycetales
Actinobacteria	Actinobacteria	Actinomycetales
Actinobacteria	Actinobacteria	Actinomycetales
Actinobacteria	Actinobacteria	Actinomycetales
Actinobacteria	Actinobacteria	Actinomycetales
Actinobacteria	Actinobacteria	Actinomycetales
Actinobacteria	Actinobacteria	Actinomycetales
Actinobacteria	Actinobacteria	Actinomycetales
Actinobacteria	Actinobacteria	Actinomycetales

Actinobacteria	Actinobacteria	Actinomycetales
Actinobacteria	Actinobacteria	Actinomycetales
Actinobacteria	Actinobacteria	Coriobacteriales
Armatimonadetes	Armatimonadia	Armatimonadales
Bacteroidetes	Flavobacteriia	Flavobacteriales
Bacteroidetes	Sphingobacteriia	Sphingobacteriales
Chloroflexi	Chloroflexia	Chloroflexales
Cyanobacteria	Oscillatoriophycideae	Chroococcales
Cyanobacteria	Oscillatoriophycideae	Oscillatoriales
Cyanobacteria	Oscillatoriophycideae	Oscillatoriales
Firmicutes	Bacilli	Bacillales
Firmicutes	Bacilli	Bacillales
Firmicutes	Bacilli	Bacillales
Firmicutes	Bacilli	Bacillales
Firmicutes	Bacilli	Bacillales
Firmicutes	Bacilli	Bacillales
Firmicutes	Bacilli	Bacillales
Firmicutes	Bacilli	Bacillales
Firmicutes	Bacilli	Bacillales
Firmicutes	Bacilli	Bacillales
Firmicutes	Bacilli	Lactobacillales
Firmicutes	Clostridia	Clostridiales
Firmicutes	Clostridia	Clostridiales
Firmicutes	Clostridia	Clostridiales
Firmicutes	Clostridia	Clostridiales
Firmicutes	Clostridia	Clostridiales
Firmicutes	Clostridia	Clostridiales
Firmicutes	Clostridia	Clostridiales
Firmicutes	Clostridia	Clostridiales
Firmicutes	Clostridia	Clostridiales
Proteobacteria	Alphaproteobacteria	Parvularculales
Proteobacteria	Alphaproteobacteria	Rhizobiales
Proteobacteria	Alphaproteobacteria	Rhizobiales
Proteobacteria	Alphaproteobacteria	Rhizobiales
Proteobacteria	Alphaproteobacteria	Rhizobiales
Proteobacteria	Alphaproteobacteria	Rhodobacterales
Proteobacteria	Alphaproteobacteria	Rhodospirillales
Proteobacteria	Alphaproteobacteria	Rhodospirillales
Proteobacteria	Alphaproteobacteria	Sphingomonadales
Proteobacteria	Alphaproteobacteria	Sphingomonadales
Proteobacteria	Betaproteobacteria	Burkholderiales
Proteobacteria	Betaproteobacteria	Burkholderiales
Proteobacteria	Betaproteobacteria	Burkholderiales
Proteobacteria	Betaproteobacteria	Burkholderiales
Proteobacteria	Betaproteobacteria	Burkholderiales
Proteobacteria	Betaproteobacteria	Burkholderiales

Proteobacteria	Betaproteobacteria	Hydrogenophilales
Proteobacteria	Betaproteobacteria	Methylophilales
Proteobacteria	Betaproteobacteria	Methylophilales
Proteobacteria	Betaproteobacteria	Rhodocyclales
Proteobacteria	Betaproteobacteria	Rhodocyclales
Proteobacteria	Deltaproteobacteria	Desulfuromonadales
Proteobacteria	Deltaproteobacteria	Desulfuromonadales
Proteobacteria	Deltaproteobacteria	Myxococcales
Proteobacteria	Deltaproteobacteria	Myxococcales
Proteobacteria	Deltaproteobacteria	Myxococcales
Proteobacteria	Gammaproteobacteria	Alteromonadales
Proteobacteria	Gammaproteobacteria	Alteromonadales
Proteobacteria	Gammaproteobacteria	Enterobacteriales
Proteobacteria	Gammaproteobacteria	Enterobacteriales
Proteobacteria	Gammaproteobacteria	Enterobacteriales
Proteobacteria	Gammaproteobacteria	Xanthomonadales
Proteobacteria	Gammaproteobacteria	Xanthomonadales
Verrucomicrobia	Spartobacteria	Spartobacteria
Actinobacteria	Actinobacteria	Actinomycetales
Actinobacteria	Actinobacteria	Actinomycetales
Bacteroidetes	Bacteroidia	Bacteroidales
Bacteroidetes	Flavobacteriia	Flavobacteriales
Bacteroidetes	Flavobacteriia	Flavobacteriales
Bacteroidetes	Flavobacteriia	Flavobacteriales
Firmicutes	Clostridia	Clostridiales
Firmicutes	Clostridia	Clostridiales
Lentisphaerae	Oligosphaeria	Oligosphaerales
Proteobacteria	Betaproteobacteria	Burkholderiales
Proteobacteria	Betaproteobacteria	Burkholderiales
Proteobacteria	Betaproteobacteria	Rhodocyclales
Proteobacteria	Epsilonproteobacteria	Campylobacterales
Proteobacteria	Gammaproteobacteria	Oceanospirillales
Proteobacteria	Gammaproteobacteria	Thiotrichales
Proteobacteria	Gammaproteobacteria	Xanthomonadales
Proteobacteria	Gammaproteobacteria	Xanthomonadales
Actinobacteria	Actinobacteria	Actinomycetales
Actinobacteria	Actinobacteria	Actinomycetales
Actinobacteria	Actinobacteria	Actinomycetales
Actinobacteria	Actinobacteria	Actinomycetales
Proteobacteria	Alphaproteobacteria	Rhizobiales
Proteobacteria	Alphaproteobacteria	Rhodobacterales
Proteobacteria	Betaproteobacteria	Methylophilales
Proteobacteria	Deltaproteobacteria	Desulfovibrionales
Proteobacteria	Gammaproteobacteria	Cardiobacteriales
Proteobacteria	Gammaproteobacteria	Xanthomonadales
Proteobacteria	Gammaproteobacteria	Xanthomonadales

Proteobacteria	Zetaproteobacteria	Mariprofundales
Spirochaetes	Spirochaetia	Spirochaetales
Actinobacteria	Actinobacteria	Actinomycetales
Actinobacteria	Actinobacteria	Actinomycetales
Actinobacteria	Actinobacteria	Actinomycetales
Actinobacteria	Actinobacteria	Actinomycetales
Bacteroidetes	Cytophagia	Cytophagales
Chloroflexi	Caldilineae	Caldilineales
Firmicutes	Bacilli	Lactobacillales
Firmicutes	Bacilli	Lactobacillales
Firmicutes	Bacilli	Lactobacillales
Firmicutes	Bacilli	Lactobacillales
Firmicutes	Bacilli	Lactobacillales
Proteobacteria	Alphaproteobacteria	Rhizobiales
Proteobacteria	Alphaproteobacteria	Rhizobiales
Proteobacteria	Alphaproteobacteria	Rhizobiales
Proteobacteria	Alphaproteobacteria	Rhodobacterales
Proteobacteria	Alphaproteobacteria	Rhodobacterales
Proteobacteria	Alphaproteobacteria	Sphingomonadales
Proteobacteria	Betaproteobacteria	Burkholderiales
Proteobacteria	Betaproteobacteria	Burkholderiales
Proteobacteria	Betaproteobacteria	Burkholderiales
Proteobacteria	Betaproteobacteria	Burkholderiales
Proteobacteria	Betaproteobacteria	Burkholderiales
Proteobacteria	Betaproteobacteria	Neisseriales
Proteobacteria	Gammaproteobacteria	Acidithiobacillales
Proteobacteria	Gammaproteobacteria	Chromatiales
Proteobacteria	Gammaproteobacteria	Enterobacteriales
Proteobacteria	Gammaproteobacteria	Enterobacteriales
Proteobacteria	Gammaproteobacteria	Enterobacteriales
Proteobacteria	Gammaproteobacteria	Legionellales
Proteobacteria	Gammaproteobacteria	Methylococcales
Proteobacteria	Gammaproteobacteria	Oceanospirillales
Proteobacteria	Gammaproteobacteria	Pseudomonadales
Proteobacteria	Gammaproteobacteria	Pseudomonadales
Tenericutes	Mollicutes	Acholeplasmatales
Actinobacteria	Actinobacteria	Actinomycetales
Actinobacteria	Actinobacteria	Actinomycetales
Actinobacteria	Actinobacteria	Actinomycetales
Actinobacteria	Actinobacteria	Actinomycetales
Actinobacteria	Actinobacteria	Actinomycetales
Actinobacteria	Actinobacteria	Actinomycetales
Actinobacteria	Actinobacteria	Actinomycetales
Actinobacteria	Actinobacteria	Actinomycetales
Actinobacteria	Actinobacteria	Actinomycetales
Actinobacteria	Actinobacteria	Actinomycetales

Actinobacteria	Actinobacteria	Nitriliruptorales
Bacteroidetes	Bacteroidia	Bacteroidales
Bacteroidetes	Flavobacteriia	Flavobacteriales
Firmicutes	Bacilli	Bacillales
Lentisphaerae	Lentisphaeria	Victivallales
Proteobacteria	Alphaproteobacteria	Caulobacterales
Proteobacteria	Alphaproteobacteria	Caulobacterales
Proteobacteria	Alphaproteobacteria	Caulobacterales
Proteobacteria	Alphaproteobacteria	Rhizobiales
Proteobacteria	Alphaproteobacteria	Rhizobiales
Proteobacteria	Alphaproteobacteria	Rhizobiales
Proteobacteria	Alphaproteobacteria	Rhizobiales
Proteobacteria	Alphaproteobacteria	Rhizobiales
Proteobacteria	Alphaproteobacteria	Rhizobiales
Proteobacteria	Alphaproteobacteria	Rhizobiales
Proteobacteria	Alphaproteobacteria	Rhodobacterales
Proteobacteria	Alphaproteobacteria	Rhodobacterales
Proteobacteria	Alphaproteobacteria	Rhodospirillales
Proteobacteria	Alphaproteobacteria	Sphingomonadales
Proteobacteria	Alphaproteobacteria	Sphingomonadales
Proteobacteria	Betaproteobacteria	Burkholderiales
Proteobacteria	Betaproteobacteria	Burkholderiales
Proteobacteria	Betaproteobacteria	Neisseriales
Proteobacteria	Betaproteobacteria	Neisseriales
Proteobacteria	Gammaproteobacteria	Alteromonadales
Proteobacteria	Gammaproteobacteria	Alteromonadales
Proteobacteria	Gammaproteobacteria	Alteromonadales
Proteobacteria	Gammaproteobacteria	Alteromonadales
Proteobacteria	Gammaproteobacteria	Oceanospirillales
Proteobacteria	Gammaproteobacteria	Pasteurellales
Proteobacteria	Gammaproteobacteria	Pseudomonadales
Proteobacteria	Gammaproteobacteria	Xanthomonadales
Proteobacteria	Gammaproteobacteria	Xanthomonadales
Proteobacteria	Gammaproteobacteria	unclassifiedGammaproteobacteria
Verrucomicrobia	Opitutae	Opitutales
Actinobacteria	Actinobacteria	Actinomycetales
Actinobacteria	Actinobacteria	Actinomycetales
Actinobacteria	Actinobacteria	Actinomycetales
Actinobacteria	Actinobacteria	Actinomycetales
Actinobacteria	Actinobacteria	Actinomycetales
Actinobacteria	Actinobacteria	Actinomycetales
Actinobacteria	Actinobacteria	Actinomycetales
Actinobacteria	Actinobacteria	Actinomycetales
Actinobacteria	Actinobacteria	Actinomycetales
Actinobacteria	Actinobacteria	Actinomycetales
Actinobacteria	Actinobacteria	Actinomycetales

Actinobacteria	Actinobacteria	Actinomycetales
Actinobacteria	Actinobacteria	Actinomycetales
Actinobacteria	Actinobacteria	Actinomycetales
Actinobacteria	Actinobacteria	Actinomycetales
Actinobacteria	Actinobacteria	Actinomycetales
Actinobacteria	Actinobacteria	Actinomycetales
Actinobacteria	Actinobacteria	Actinomycetales
Aquificae	Aquificae	Aquificales
Bacteroidetes	Cytophagia	Cytophagales
Bacteroidetes	Flavobacteriia	Flavobacteriales
Bacteroidetes	Flavobacteriia	Flavobacteriales
Bacteroidetes	Flavobacteriia	Flavobacteriales
Bacteroidetes	Flavobacteriia	Flavobacteriales
Bacteroidetes	Flavobacteriia	Flavobacteriales
Bacteroidetes	Sphingobacteriia	Sphingobacteriales
Cyanobacteria	Synechococcophycideae	Pseudanabaenales
Firmicutes	Bacilli	Bacillales
Firmicutes	Bacilli	Bacillales
Firmicutes	Bacilli	Bacillales
Firmicutes	Bacilli	Bacillales
Firmicutes	Bacilli	Bacillales
Firmicutes	Bacilli	Bacillales
Firmicutes	Bacilli	Bacillales
Firmicutes	Bacilli	Bacillales
Firmicutes	Bacilli	Lactobacillales
Firmicutes	Bacilli	Lactobacillales
Firmicutes	Bacilli	Lactobacillales
Firmicutes	Bacilli	Lactobacillales
Firmicutes	Bacilli	Lactobacillales
Firmicutes	Bacilli	Lactobacillales
Firmicutes	Bacilli	Lactobacillales
Firmicutes	Bacilli	Lactobacillales
Firmicutes	Bacilli	Lactobacillales
Firmicutes	Bacilli	Lactobacillales
Firmicutes	Bacilli	Lactobacillales
Firmicutes	Bacilli	Lactobacillales
Firmicutes	Bacilli	Lactobacillales
Firmicutes	Bacilli	Lactobacillales
Firmicutes	Bacilli	Lactobacillales
Firmicutes	Clostridia	Clostridiales
Firmicutes	Clostridia	Clostridiales
Firmicutes	Erysipelotrichia	Erysipelotrichales
Proteobacteria	Alphaproteobacteria	Caulobacterales
Proteobacteria	Alphaproteobacteria	Rhizobiales
Proteobacteria	Alphaproteobacteria	Rhizobiales
Proteobacteria	Alphaproteobacteria	Rhodobacterales

Proteobacteria	Betaproteobacteria	Burkholderiales
Proteobacteria	Betaproteobacteria	Burkholderiales
Proteobacteria	Betaproteobacteria	Methylophilales
Proteobacteria	Betaproteobacteria	Methylophilales
Proteobacteria	Betaproteobacteria	Neisseriales
Proteobacteria	Betaproteobacteria	Neisseriales
Proteobacteria	Betaproteobacteria	Neisseriales
Proteobacteria	Betaproteobacteria	Neisseriales
Proteobacteria	Betaproteobacteria	Neisseriales
Proteobacteria	Betaproteobacteria	Neisseriales
Proteobacteria	Betaproteobacteria	Neisseriales
Proteobacteria	Betaproteobacteria	Neisseriales
Proteobacteria	Betaproteobacteria	Neisseriales
Proteobacteria	Betaproteobacteria	Neisseriales
Proteobacteria	Betaproteobacteria	Neisseriales
Proteobacteria	Betaproteobacteria	Rhodocyclales
Proteobacteria	Betaproteobacteria	Rhodocyclales
Proteobacteria	Deltaproteobacteria	Bdellovibrionales
Proteobacteria	Epsilonproteobacteria	Campylobacterales
Proteobacteria	Gammaproteobacteria	Cardiobacteriales
Proteobacteria	Gammaproteobacteria	Enterobacteriales
Proteobacteria	Gammaproteobacteria	Enterobacteriales
Proteobacteria	Gammaproteobacteria	Enterobacteriales
Proteobacteria	Gammaproteobacteria	Legionellales
Proteobacteria	Gammaproteobacteria	Oceanospirillales
Proteobacteria	Gammaproteobacteria	Pasteurellales
Proteobacteria	Gammaproteobacteria	Pasteurellales
Proteobacteria	Gammaproteobacteria	Pasteurellales
Proteobacteria	Gammaproteobacteria	Pasteurellales
Proteobacteria	Gammaproteobacteria	Pasteurellales
Proteobacteria	Gammaproteobacteria	Pasteurellales
Proteobacteria	Gammaproteobacteria	Pasteurellales
Proteobacteria	Gammaproteobacteria	Pasteurellales
Proteobacteria	Gammaproteobacteria	Pseudomonadales
Proteobacteria	Gammaproteobacteria	Pseudomonadales
Proteobacteria	Gammaproteobacteria	Xanthomonadales
Spirochaetes	Spirochaetia	Spirochaetales
Spirochaetes	Spirochaetia	Spirochaetales
Tenericutes	Mollicutes	Acholeplasmatales
Tenericutes	Mollicutes	Acholeplasmatales
Tenericutes	Mollicutes	Entomoplasmatales
Actinobacteria	Actinobacteria	Actinomycetales
Actinobacteria	Actinobacteria	Actinomycetales
Actinobacteria	Actinobacteria	Actinomycetales
Actinobacteria	Actinobacteria	Actinomycetales
Actinobacteria	Actinobacteria	Actinomycetales

Actinobacteria	Actinobacteria	Actinomycetales
Actinobacteria	Actinobacteria	Actinomycetales
Actinobacteria	Actinobacteria	Actinomycetales
Actinobacteria	Actinobacteria	Actinomycetales
Actinobacteria	Actinobacteria	Coriobacteriales
Bacteroidetes	Bacteroidia	Bacteroidales
Bacteroidetes	Flavobacteriia	Flavobacteriales
Bacteroidetes	Flavobacteriia	Flavobacteriales
Bacteroidetes	Flavobacteriia	Flavobacteriales
Bacteroidetes	Flavobacteriia	Flavobacteriales
Bacteroidetes	Flavobacteriia	Flavobacteriales
Bacteroidetes	Flavobacteriia	Flavobacteriales
Bacteroidetes	Flavobacteriia	Flavobacteriales
Bacteroidetes	Flavobacteriia	Flavobacteriales
Bacteroidetes	Sphingobacteriia	Sphingobacteriales
Firmicutes	Bacilli	Bacillales
Firmicutes	Bacilli	Bacillales
Firmicutes	Bacilli	Bacillales
Firmicutes	Bacilli	Bacillales
Firmicutes	Clostridia	Clostridiales
Firmicutes	Clostridia	Clostridiales
Firmicutes	Clostridia	Clostridiales
Proteobacteria	Alphaproteobacteria	Rhizobiales
Proteobacteria	Alphaproteobacteria	Rhizobiales
Proteobacteria	Alphaproteobacteria	Rhizobiales
Proteobacteria	Alphaproteobacteria	Rhizobiales
Proteobacteria	Alphaproteobacteria	Rhizobiales
Proteobacteria	Alphaproteobacteria	Rhizobiales
Proteobacteria	Alphaproteobacteria	Rhizobiales
Proteobacteria	Alphaproteobacteria	Rhodobacterales
Proteobacteria	Alphaproteobacteria	Rhodobacterales
Proteobacteria	Alphaproteobacteria	Rhodospirillales
Proteobacteria	Alphaproteobacteria	Rickettsiales
Proteobacteria	Betaproteobacteria	Burkholderiales
Proteobacteria	Betaproteobacteria	Burkholderiales
Proteobacteria	Betaproteobacteria	Burkholderiales
Proteobacteria	Betaproteobacteria	Methylophilales
Proteobacteria	Betaproteobacteria	Nitrosomonadales
Proteobacteria	Betaproteobacteria	Rhodocyclales
Proteobacteria	Betaproteobacteria	Rhodocyclales
Proteobacteria	Betaproteobacteria	Rhodocyclales
Proteobacteria	Deltaproteobacteria	Myxococcales
Proteobacteria	Gammaproteobacteria	Chromatiales
Proteobacteria	Gammaproteobacteria	Enterobacteriales
Proteobacteria	Gammaproteobacteria	Enterobacteriales
Proteobacteria	Gammaproteobacteria	Enterobacteriales

Proteobacteria	Gammaproteobacteria	Enterobacteriales
Proteobacteria	Gammaproteobacteria	Methylococcales
Proteobacteria	Gammaproteobacteria	Methylococcales
Proteobacteria	Gammaproteobacteria	Pseudomonadales
Proteobacteria	Gammaproteobacteria	Pseudomonadales
Proteobacteria	Gammaproteobacteria	Pseudomonadales
Proteobacteria	Gammaproteobacteria	Vibrionales
Proteobacteria	Gammaproteobacteria	unclassifiedGammaproteobacteria
Synergistetes	Synergistia	Synergistales
Actinobacteria	Actinobacteria	Actinomycetales
Actinobacteria	Actinobacteria	Actinomycetales
Actinobacteria	Actinobacteria	Actinomycetales
Actinobacteria	Actinobacteria	Actinomycetales
Actinobacteria	Actinobacteria	Actinomycetales
Actinobacteria	Actinobacteria	Actinomycetales
Actinobacteria	Actinobacteria	Actinomycetales
Bacteroidetes	Cytophagia	Cytophagales
Firmicutes	Clostridia	Clostridiales
Proteobacteria	Alphaproteobacteria	Rhizobiales
Proteobacteria	Alphaproteobacteria	Sphingomonadales
Proteobacteria	Betaproteobacteria	Burkholderiales
Proteobacteria	Gammaproteobacteria	Alteromonadales
Proteobacteria	Gammaproteobacteria	Methylococcales
Proteobacteria	Gammaproteobacteria	Methylococcales
Proteobacteria	Betaproteobacteria	Burkholderiales
Proteobacteria	Betaproteobacteria	Burkholderiales
Proteobacteria	Betaproteobacteria	Burkholderiales
Proteobacteria	Betaproteobacteria	Burkholderiales
Proteobacteria	Gammaproteobacteria	Pseudomonadales
Actinobacteria	Actinobacteria	Actinomycetales
Actinobacteria	Actinobacteria	Actinomycetales
Actinobacteria	Actinobacteria	Actinomycetales
Actinobacteria	Actinobacteria	Actinomycetales
Actinobacteria	Actinobacteria	Actinomycetales
Actinobacteria	Actinobacteria	Actinomycetales
Actinobacteria	Actinobacteria	Actinomycetales
Actinobacteria	Actinobacteria	Actinomycetales
Actinobacteria	Actinobacteria	Actinomycetales
Actinobacteria	Actinobacteria	Actinomycetales
Actinobacteria	Actinobacteria	Actinomycetales
Actinobacteria	Actinobacteria	Actinomycetales
Actinobacteria	Actinobacteria	Actinomycetales
Actinobacteria	Actinobacteria	Actinomycetales
Actinobacteria	Actinobacteria	Actinomycetales
Actinobacteria	Actinobacteria	Actinomycetales
Actinobacteria	Actinobacteria	Actinomycetales

Actinobacteria	Actinobacteria	Actinomycetales
Actinobacteria	Actinobacteria	Actinomycetales
Actinobacteria	Actinobacteria	Actinomycetales
Actinobacteria	Actinobacteria	Actinomycetales
Actinobacteria	Actinobacteria	Actinomycetales
Actinobacteria	Actinobacteria	Actinomycetales
Actinobacteria	Actinobacteria	Actinomycetales
Actinobacteria	Actinobacteria	Actinomycetales
Bacteroidetes	Cytophagia	Cytophagales
Bacteroidetes	Flavobacteriia	Flavobacteriales
Bacteroidetes	Flavobacteriia	Flavobacteriales
Bacteroidetes	Flavobacteriia	Flavobacteriales
Bacteroidetes	Flavobacteriia	Flavobacteriales
Bacteroidetes	Sphingobacteriia	Sphingobacteriales
Bacteroidetes	Sphingobacteriia	Sphingobacteriales
Bacteroidetes	Sphingobacteriia	Sphingobacteriales
Bacteroidetes	Sphingobacteriia	Sphingobacteriales
Bacteroidetes	Sphingobacteriia	Sphingobacteriales
Bacteroidetes	Sphingobacteriia	Sphingobacteriales
Bacteroidetes	Sphingobacteriia	Sphingobacteriales
Bacteroidetes	Sphingobacteriia	Sphingobacteriales
Bacteroidetes	Sphingobacteriia	Sphingobacteriales
Bacteroidetes	Sphingobacteriia	Sphingobacteriales
Bacteroidetes	Sphingobacteriia	Sphingobacteriales
Firmicutes	Bacilli	Bacillales
Firmicutes	Bacilli	Bacillales
Firmicutes	Bacilli	Bacillales
Firmicutes	Bacilli	Bacillales
Firmicutes	Bacilli	Bacillales
Firmicutes	Bacilli	Lactobacillales
Planctomycetes	Planctomycetia	Planctomycetales
Proteobacteria	Alphaproteobacteria	Rhizobiales
Proteobacteria	Alphaproteobacteria	Rhizobiales
Proteobacteria	Alphaproteobacteria	Rhizobiales
Proteobacteria	Alphaproteobacteria	Rhizobiales
Proteobacteria	Alphaproteobacteria	Rhizobiales
Proteobacteria	Alphaproteobacteria	Rhizobiales
Proteobacteria	Alphaproteobacteria	Rhodospirillales
Proteobacteria	Alphaproteobacteria	Sphingomonadales
Proteobacteria	Alphaproteobacteria	Sphingomonadales
Proteobacteria	Betaproteobacteria	Burkholderiales
Proteobacteria	Betaproteobacteria	Burkholderiales
Proteobacteria	Betaproteobacteria	Burkholderiales
Proteobacteria	Betaproteobacteria	Burkholderiales
Proteobacteria	Betaproteobacteria	Burkholderiales
Proteobacteria	Betaproteobacteria	Burkholderiales

Proteobacteria	Betaproteobacteria	Burkholderiales
Proteobacteria	Betaproteobacteria	Gallionellales
Proteobacteria	Deltaproteobacteria	Myxococcales
Proteobacteria	Gammaproteobacteria	Enterobacteriales
Proteobacteria	Gammaproteobacteria	Enterobacteriales
Proteobacteria	Gammaproteobacteria	Enterobacteriales
Proteobacteria	Gammaproteobacteria	Enterobacteriales
Proteobacteria	Gammaproteobacteria	Legionellales
Proteobacteria	Gammaproteobacteria	Oceanospirillales
Proteobacteria	Gammaproteobacteria	Pseudomonadales
Proteobacteria	Gammaproteobacteria	Pseudomonadales
Proteobacteria	Gammaproteobacteria	Xanthomonadales
Proteobacteria	Gammaproteobacteria	Xanthomonadales
Actinobacteria	Actinobacteria	Actinomycetales
Proteobacteria	Alphaproteobacteria	Rhizobiales
Proteobacteria	Alphaproteobacteria	Rhizobiales
Proteobacteria	Alphaproteobacteria	Rhizobiales
Proteobacteria	Alphaproteobacteria	Sphingomonadales
Proteobacteria	Alphaproteobacteria	Sphingomonadales
Proteobacteria	Alphaproteobacteria	Sphingomonadales
Proteobacteria	Alphaproteobacteria	Sphingomonadales
Proteobacteria	Betaproteobacteria	Burkholderiales
Proteobacteria	Betaproteobacteria	Burkholderiales
Proteobacteria	Betaproteobacteria	Burkholderiales
Proteobacteria	Betaproteobacteria	Burkholderiales
Proteobacteria	Betaproteobacteria	Burkholderiales
Proteobacteria	Gammaproteobacteria	Enterobacteriales
Proteobacteria	Gammaproteobacteria	Pseudomonadales
Actinobacteria	Actinobacteria	Actinomycetales
Actinobacteria	Actinobacteria	Actinomycetales
Actinobacteria	Actinobacteria	Actinomycetales
Bacteroidetes	Flavobacteriia	Flavobacteriales
Firmicutes	Bacilli	Bacillales
Firmicutes	Bacilli	Bacillales
Proteobacteria	Alphaproteobacteria	Rhizobiales
Proteobacteria	Alphaproteobacteria	Rhodobacterales
Proteobacteria	Betaproteobacteria	Burkholderiales
Actinobacteria	Actinobacteria	Actinomycetales
Actinobacteria	Actinobacteria	Actinomycetales
Firmicutes	Bacilli	Bacillales
Proteobacteria	Gammaproteobacteria	Enterobacteriales
Actinobacteria	Actinobacteria	Actinomycetales
Actinobacteria	Actinobacteria	Actinomycetales
Actinobacteria	Actinobacteria	Actinomycetales
Cyanobacteria	Prochlorales	Prochlorales
Proteobacteria	Alphaproteobacteria	Rhodobacterales

Proteobacteria	Alphaproteobacteria	Rhodobacterales
Proteobacteria	Betaproteobacteria	Burkholderiales
Proteobacteria	Deltaproteobacteria	Desulfurellales
Proteobacteria	Deltaproteobacteria	Myxococcales
Proteobacteria	Deltaproteobacteria	Myxococcales
Proteobacteria	Gammaproteobacteria	Xanthomonadales
Actinobacteria	Actinobacteria	Actinomycetales
Actinobacteria	Actinobacteria	Actinomycetales
Actinobacteria	Actinobacteria	Actinomycetales
Actinobacteria	Actinobacteria	Actinomycetales
Actinobacteria	Actinobacteria	Actinomycetales
Actinobacteria	Actinobacteria	Actinomycetales
Actinobacteria	Actinobacteria	Actinomycetales
Bacteroidetes	Flavobacteriia	Flavobacteriales
Bacteroidetes	Flavobacteriia	Flavobacteriales
Chlamydiae	Chlamydiia	Chlamydiales
Chlorobi	Chlorobia	Chlorobiales
Firmicutes	Clostridia	Clostridiales
Fusobacteria	Fusobacteriia	Fusobacteriales
Proteobacteria	Alphaproteobacteria	Caulobacterales
Proteobacteria	Alphaproteobacteria	Rhizobiales
Proteobacteria	Alphaproteobacteria	Rhizobiales
Proteobacteria	Alphaproteobacteria	Rhizobiales
Proteobacteria	Alphaproteobacteria	Rhizobiales
Proteobacteria	Alphaproteobacteria	Rhizobiales
Proteobacteria	Alphaproteobacteria	Rhizobiales
Proteobacteria	Alphaproteobacteria	Rhizobiales
Proteobacteria	Alphaproteobacteria	Sphingomonadales
Proteobacteria	Alphaproteobacteria	Sphingomonadales
Proteobacteria	Deltaproteobacteria	Desulfovibrionales
Proteobacteria	Deltaproteobacteria	Myxococcales
Proteobacteria	Gammaproteobacteria	Pseudomonadales
Proteobacteria	Gammaproteobacteria	Xanthomonadales
Verrucomicrobia	Verrucomicrobiae	Verrucomicrobiales
Actinobacteria	Actinobacteria	Actinomycetales
Actinobacteria	Actinobacteria	Actinomycetales
Actinobacteria	Actinobacteria	Actinomycetales
Actinobacteria	Actinobacteria	Actinomycetales
Bacteroidetes	Sphingobacteriia	Sphingobacteriales
Bacteroidetes	Sphingobacteriia	Sphingobacteriales
Proteobacteria	Alphaproteobacteria	Sphingomonadales
Proteobacteria	Betaproteobacteria	Hydrogenophilales
Proteobacteria	Gammaproteobacteria	Alteromonadales
Proteobacteria	Gammaproteobacteria	Alteromonadales
Proteobacteria	Gammaproteobacteria	Oceanospirillales
Firmicutes	Clostridia	Clostridiales

Actinobacteria	Actinobacteria	Actinomycetales
Actinobacteria	Actinobacteria	Actinomycetales
Actinobacteria	Actinobacteria	Actinomycetales
Actinobacteria	Actinobacteria	Actinomycetales
Actinobacteria	Actinobacteria	Actinomycetales
Actinobacteria	Actinobacteria	Actinomycetales
Actinobacteria	Actinobacteria	Actinomycetales
Bacteroidetes	Bacteroidia	Bacteroidales
Bacteroidetes	Cytophagia	Cytophagales
Firmicutes	Bacilli	Bacillales
Firmicutes	Bacilli	Bacillales
Firmicutes	Bacilli	Bacillales
Firmicutes	Bacilli	Bacillales
Firmicutes	Bacilli	Bacillales
Firmicutes	Bacilli	Bacillales
Firmicutes	Bacilli	Bacillales
Firmicutes	Bacilli	Lactobacillales
Planctomycetes	Planctomycetia	Planctomycetales
Proteobacteria	Alphaproteobacteria	Rhizobiales
Proteobacteria	Alphaproteobacteria	Rhizobiales
Proteobacteria	Alphaproteobacteria	Rhizobiales
Proteobacteria	Alphaproteobacteria	Rhodospirillales
Proteobacteria	Alphaproteobacteria	Sphingomonadales
Proteobacteria	Alphaproteobacteria	Sphingomonadales
Proteobacteria	Alphaproteobacteria	Sphingomonadales
Proteobacteria	Alphaproteobacteria	Sphingomonadales
Proteobacteria	Alphaproteobacteria	Sphingomonadales
Proteobacteria	Betaproteobacteria	Burkholderiales
Proteobacteria	Betaproteobacteria	Burkholderiales
Proteobacteria	Betaproteobacteria	Burkholderiales
Proteobacteria	Betaproteobacteria	Burkholderiales
Proteobacteria	Betaproteobacteria	Burkholderiales
Proteobacteria	Betaproteobacteria	Burkholderiales
Proteobacteria	Betaproteobacteria	Burkholderiales
Proteobacteria	Betaproteobacteria	Hydrogenophilales
Proteobacteria	Betaproteobacteria	Rhodocyclales
Proteobacteria	Deltaproteobacteria	Myxococcales
Proteobacteria	Deltaproteobacteria	Myxococcales
Proteobacteria	Gammaproteobacteria	Methylococcales
Actinobacteria	Actinobacteria	Actinomycetales
Actinobacteria	Actinobacteria	Actinomycetales
Actinobacteria	Actinobacteria	Actinomycetales
Actinobacteria	Actinobacteria	Actinomycetales
Actinobacteria	Actinobacteria	Actinomycetales
Actinobacteria	Actinobacteria	Actinomycetales
Actinobacteria	Actinobacteria	Actinomycetales

Actinobacteria	Actinobacteria	Actinomycetales
Actinobacteria	Actinobacteria	Actinomycetales
Actinobacteria	Actinobacteria	Actinomycetales
Bacteroidetes	Flavobacteriia	Flavobacteriales
Bacteroidetes	Flavobacteriia	Flavobacteriales
Bacteroidetes	Sphingobacteriia	Sphingobacteriales
Firmicutes	Bacilli	Bacillales
Firmicutes	Bacilli	Lactobacillales
Firmicutes	Clostridia	Clostridiales
Proteobacteria	Alphaproteobacteria	Rhizobiales
Proteobacteria	Alphaproteobacteria	Rhizobiales
Proteobacteria	Alphaproteobacteria	Rhodobacterales
Proteobacteria	Alphaproteobacteria	Rhodobacterales
Proteobacteria	Alphaproteobacteria	Rhodobacterales
Proteobacteria	Alphaproteobacteria	Sphingomonadales
Proteobacteria	Betaproteobacteria	Burkholderiales
Proteobacteria	Betaproteobacteria	Burkholderiales
Proteobacteria	Betaproteobacteria	Rhodocyclales
Proteobacteria	Gammaproteobacteria	Enterobacteriales
Actinobacteria	Actinobacteria	Actinomycetales
Actinobacteria	Actinobacteria	Actinomycetales
Actinobacteria	Actinobacteria	Actinomycetales
Actinobacteria	Actinobacteria	Actinomycetales
Bacteroidetes	Flavobacteriia	Flavobacteriales
Chloroflexi	Anaerolineae	Anaerolineales
Chloroflexi	Ktedonobacteria	Ktedonobacterales
Deinococcus-Thermus	Deinococci	Deinococcales
Firmicutes	Bacilli	Bacillales
Firmicutes	Clostridia	Clostridiales
Firmicutes	Clostridia	Clostridiales
Firmicutes	Clostridia	Thermoanaerobacterales
Planctomycetes	Phycisphaerae	Phycisphaerales
Proteobacteria	Alphaproteobacteria	Caulobacterales
Proteobacteria	Alphaproteobacteria	Rhizobiales
Proteobacteria	Alphaproteobacteria	Rhizobiales
Proteobacteria	Alphaproteobacteria	Rhizobiales
Proteobacteria	Alphaproteobacteria	Rhizobiales
Proteobacteria	Alphaproteobacteria	Rhizobiales
Proteobacteria	Alphaproteobacteria	Rhizobiales
Proteobacteria	Alphaproteobacteria	Rhizobiales
Proteobacteria	Alphaproteobacteria	Rhizobiales
Proteobacteria	Alphaproteobacteria	Rhizobiales
Proteobacteria	Alphaproteobacteria	Rhizobiales
Proteobacteria	Alphaproteobacteria	Rhizobiales
Proteobacteria	Alphaproteobacteria	Rhizobiales
Proteobacteria	Alphaproteobacteria	Rhodobacterales

Proteobacteria	Alphaproteobacteria	Rhodobacterales
Proteobacteria	Alphaproteobacteria	Sneathiellales
Proteobacteria	Alphaproteobacteria	Sphingomonadales
Proteobacteria	Betaproteobacteria	Burkholderiales
Proteobacteria	Betaproteobacteria	Burkholderiales
Proteobacteria	Betaproteobacteria	Burkholderiales
Proteobacteria	Betaproteobacteria	Burkholderiales
Proteobacteria	Betaproteobacteria	Methylophilales
Proteobacteria	Deltaproteobacteria	Bdellovibrionales
Proteobacteria	Deltaproteobacteria	Bdellovibrionales
Proteobacteria	Deltaproteobacteria	Desulfovibrionales
Proteobacteria	Deltaproteobacteria	Myxococcales
Proteobacteria	Deltaproteobacteria	Myxococcales
Proteobacteria	Deltaproteobacteria	Myxococcales
Proteobacteria	Gammaproteobacteria	Chromatiales
Proteobacteria	Gammaproteobacteria	Chromatiales
Proteobacteria	Gammaproteobacteria	Legionellales
Proteobacteria	Gammaproteobacteria	Oceanospirillales
Proteobacteria	Gammaproteobacteria	Pseudomonadales
Proteobacteria	Gammaproteobacteria	Salinisphaerales
Proteobacteria	Gammaproteobacteria	Xanthomonadales
Tenericutes	Mollicutes	Acholeplasmatales
Tenericutes	Mollicutes	Acholeplasmatales
Tenericutes	Mollicutes	Acholeplasmatales
Verrucomicrobia	Opitutae	Opitutales
Actinobacteria	Actinobacteria	Actinomycetales
Actinobacteria	Actinobacteria	Actinomycetales
Bacteroidetes	Flavobacteriia	Flavobacteriales
Proteobacteria	Betaproteobacteria	Burkholderiales
Proteobacteria	Deltaproteobacteria	Bdellovibrionales
Proteobacteria	Deltaproteobacteria	Bdellovibrionales
Proteobacteria	Gammaproteobacteria	Pseudomonadales
Actinobacteria	Actinobacteria	Actinomycetales
Actinobacteria	Actinobacteria	Actinomycetales
Bacteroidetes	Flavobacteriia	Flavobacteriales
Firmicutes	Bacilli	Bacillales
Firmicutes	Bacilli	Lactobacillales
Proteobacteria	Alphaproteobacteria	Rhizobiales
Proteobacteria	Alphaproteobacteria	Rhizobiales
Proteobacteria	Alphaproteobacteria	Rhizobiales
Proteobacteria	Alphaproteobacteria	Rhodobacterales
Proteobacteria	Betaproteobacteria	Burkholderiales
Proteobacteria	Betaproteobacteria	Burkholderiales
Proteobacteria	Betaproteobacteria	Burkholderiales
Proteobacteria	Gammaproteobacteria	Aeromonadales
Proteobacteria	Gammaproteobacteria	Aeromonadales

Proteobacteria	Gammaproteobacteria	Enterobacteriales
Proteobacteria	Gammaproteobacteria	Thiotrichales
Proteobacteria	Gammaproteobacteria	Xanthomonadales
Proteobacteria	Gammaproteobacteria	Xanthomonadales
Actinobacteria	Actinobacteria	Actinomycetales
Actinobacteria	Actinobacteria	Actinomycetales
Firmicutes	Clostridia	Clostridiales
Firmicutes	Clostridia	Clostridiales
Proteobacteria	Alphaproteobacteria	Rhizobiales
Proteobacteria	Alphaproteobacteria	Rhodospirillales
Proteobacteria	Epsilonproteobacteria	Campylobacterales
Proteobacteria	Gammaproteobacteria	Enterobacteriales
Acidobacteria	Acidobacteriia	Acidobacteriales
Acidobacteria	Acidobacteriia	Acidobacteriales
Acidobacteria	Acidobacteriia	Acidobacteriales
Actinobacteria	Actinobacteria	Actinomycetales
Actinobacteria	Actinobacteria	Actinomycetales
Bacteroidetes	Sphingobacteriia	Sphingobacteriales
Proteobacteria	Betaproteobacteria	Burkholderiales
Proteobacteria	Betaproteobacteria	Nitrosomonadales
Bacteroidetes	Flavobacteriia	Flavobacteriales
Proteobacteria	Alphaproteobacteria	Caulobacterales
Actinobacteria	Actinobacteria	Actinomycetales
Actinobacteria	Actinobacteria	Actinomycetales
Bacteroidetes	Flavobacteriia	Flavobacteriales
Bacteroidetes	Sphingobacteriia	Sphingobacteriales
Cyanobacteria	Oscillatoriophycideae	Oscillatoriales
Firmicutes	Bacilli	Lactobacillales
Proteobacteria	Alphaproteobacteria	Rhizobiales
Proteobacteria	Alphaproteobacteria	Rhodobacterales
Proteobacteria	Alphaproteobacteria	Rickettsiales
Proteobacteria	Betaproteobacteria	Burkholderiales
Actinobacteria	Actinobacteria	Actinomycetales
Actinobacteria	Actinobacteria	Actinomycetales
Actinobacteria	Actinobacteria	Actinomycetales
Bacteroidetes	Cytophagia	Cytophagales
Bacteroidetes	Sphingobacteriia	Sphingobacteriales
Firmicutes	Bacilli	Bacillales
Firmicutes	Bacilli	Bacillales
Firmicutes	Bacilli	Bacillales
Proteobacteria	Alphaproteobacteria	Rhodobacterales
Proteobacteria	Alphaproteobacteria	Rhodospirillales
Proteobacteria	Alphaproteobacteria	Sphingomonadales
Proteobacteria	Betaproteobacteria	Rhodocyclales
Proteobacteria	Gammaproteobacteria	Enterobacteriales
Bacteroidetes	Sphingobacteriia	Sphingobacteriales

Proteobacteria	Betaproteobacteria	Burkholderiales
Proteobacteria	Gammaproteobacteria	Enterobacteriales
Actinobacteria	Actinobacteria	Actinomycetales
Bacteroidetes	Bacteroidia	Bacteroidales
Proteobacteria	Alphaproteobacteria	Rhizobiales
Proteobacteria	Alphaproteobacteria	Rhizobiales
Proteobacteria	Alphaproteobacteria	Rhizobiales
Proteobacteria	Alphaproteobacteria	Rhodobacterales
Proteobacteria	Alphaproteobacteria	Rhodospirillales
Proteobacteria	Betaproteobacteria	Burkholderiales
Proteobacteria	Betaproteobacteria	Nitrosomonadales
Proteobacteria	Gammaproteobacteria	Xanthomonadales
Actinobacteria	Actinobacteria	Actinomycetales
Actinobacteria	Actinobacteria	Actinomycetales
Actinobacteria	Actinobacteria	Actinomycetales
Actinobacteria	Actinobacteria	Actinomycetales
Actinobacteria	Actinobacteria	Actinomycetales
Actinobacteria	Actinobacteria	Actinomycetales
Actinobacteria	Actinobacteria	Actinomycetales
Actinobacteria	Actinobacteria	Actinomycetales
Actinobacteria	Actinobacteria	Actinomycetales
Bacteroidetes	Cytophagia	Cytophagales
Bacteroidetes	Cytophagia	Cytophagales
Bacteroidetes	Flavobacteriia	Flavobacteriales
Firmicutes	Bacilli	Bacillales
Proteobacteria	Alphaproteobacteria	Rhizobiales
Proteobacteria	Alphaproteobacteria	Rhizobiales
Proteobacteria	Epsilonproteobacteria	Campylobacterales
Proteobacteria	Gammaproteobacteria	Enterobacteriales
Proteobacteria	Gammaproteobacteria	Legionellales
Proteobacteria	Gammaproteobacteria	Pseudomonadales
Proteobacteria	Gammaproteobacteria	Xanthomonadales
Proteobacteria	Gammaproteobacteria	unclassifiedGammaproteobacteria
Actinobacteria	Actinobacteria	Actinomycetales
Actinobacteria	Actinobacteria	Actinomycetales
Actinobacteria	Actinobacteria	Actinomycetales
Actinobacteria	Actinobacteria	Actinomycetales
Bacteroidetes	Flavobacteriia	Flavobacteriales
Bacteroidetes	Flavobacteriia	Flavobacteriales
Firmicutes	Bacilli	Bacillales
Firmicutes	Bacilli	Bacillales
Firmicutes	Bacilli	Bacillales
Firmicutes	Bacilli	Bacillales
Proteobacteria	Alphaproteobacteria	Rhizobiales
Proteobacteria	Alphaproteobacteria	Rhizobiales
Proteobacteria	Alphaproteobacteria	Rhizobiales

Proteobacteria	Alphaproteobacteria	Rhodobacterales
Proteobacteria	Betaproteobacteria	Methylophilales
Proteobacteria	Betaproteobacteria	Sulfuricellales
Proteobacteria	Gammaproteobacteria	Aeromonadales
Proteobacteria	Gammaproteobacteria	Enterobacteriales
Proteobacteria	Gammaproteobacteria	Legionellales
Proteobacteria	Gammaproteobacteria	Methylococcales
Proteobacteria	Gammaproteobacteria	Pasteurellales
Actinobacteria	Actinobacteria	Actinomycetales
Actinobacteria	Actinobacteria	Actinomycetales
Actinobacteria	Actinobacteria	Actinomycetales
Actinobacteria	Actinobacteria	Actinomycetales
Actinobacteria	Actinobacteria	Actinomycetales
Actinobacteria	Actinobacteria	Actinomycetales
Bacteroidetes	Bacteroidia	Bacteroidales
Firmicutes	Bacilli	Bacillales
Proteobacteria	Alphaproteobacteria	Rhodospirillales
Proteobacteria	Betaproteobacteria	Burkholderiales
Proteobacteria	Gammaproteobacteria	Aeromonadales
Proteobacteria	Gammaproteobacteria	Aeromonadales
Actinobacteria	Actinobacteria	Actinomycetales
Actinobacteria	Actinobacteria	Actinomycetales
Proteobacteria	Gammaproteobacteria	Xanthomonadales

Taxonomy
**Family**
Acidobacteriaceae
Microthrixaceae
Acidimicrobiaceae
Acidimicrobiaceae
Acidimicrobiaceae
Acidimicrobiaceae
Iamiaceae
Actinomycetaceae
Beutenbergiaceae
Beutenbergiaceae
Beutenbergiaceae
Beutenbergiaceae
Bogoriellaceae
Bogoriellaceae
Bogoriellaceae
Bogoriellaceae
Brevibacteriaceae
Brevibacteriaceae
Brevibacteriaceae
Cellulomonadaceae
Cellulomonadaceae
Cellulomonadaceae
Cellulomonadaceae
Cellulomonadaceae
Corynebacteriaceae
Corynebacteriaceae
Corynebacteriaceae
Corynebacteriaceae
Corynebacteriaceae
Corynebacteriaceae
Corynebacteriaceae
Corynebacteriaceae
Corynebacteriaceae
Demequinaceae
Demequinaceae
Demequinaceae
Dermabacteraceae
Dermabacteraceae
Dermabacteraceae
Dermabacteraceae
Dermabacteraceae

Dermabacteraceae
Dermabacteraceae
Dermabacteraceae
Dermabacteraceae
Dermabacteraceae
Dermacoccaceae
Dermacoccaceae
Dermatophilaceae
Dermatophilaceae
Dermatophilaceae
Dermatophilaceae
Dermatophilaceae
Dermatophilaceae
Dermatophilaceae
Dermatophilaceae
Dermatophilaceae
Dietziaceae
Dietziaceae
Dietziaceae
Dietziaceae
Dietziaceae
Dietziaceae
Frankiaceae
Geodermatophilaceae
Geodermatophilaceae
Geodermatophilaceae
Geodermatophilaceae
Geodermatophilaceae
Geodermatophilaceae
Geodermatophilaceae
Intrasporangiaceae
Intrasporangiaceae
Intrasporangiaceae
Intrasporangiaceae
Intrasporangiaceae
Intrasporangiaceae
Intrasporangiaceae
Intrasporangiaceae
Intrasporangiaceae
Intrasporangiaceae
Intrasporangiaceae
Intrasporangiaceae
Intrasporangiaceae
Intrasporangiaceae
Intrasporangiaceae
Intrasporangiaceae

Intrasporangiaceae
Intrasporangiaceae
Intrasporangiaceae
Intrasporangiaceae
Intrasporangiaceae
Intrasporangiaceae
Intrasporangiaceae
Intrasporangiaceae
Intrasporangiaceae
Intrasporangiaceae
Intrasporangiaceae
Kineosporiaceae
Microbacteriaceae
Microbacteriaceae
Microbacteriaceae
Microbacteriaceae
Microbacteriaceae
Microbacteriaceae
Microbacteriaceae
Microbacteriaceae
Microbacteriaceae
Microbacteriaceae
Microbacteriaceae
Microbacteriaceae
Microbacteriaceae
Microbacteriaceae
Microbacteriaceae
Microbacteriaceae
Microbacteriaceae
Microbacteriaceae
Microbacteriaceae
Microbacteriaceae
Microbacteriaceae
Microbacteriaceae
Microbacteriaceae
Microbacteriaceae
Microbacteriaceae
Microbacteriaceae
Microbacteriaceae
Microbacteriaceae
Microbacteriaceae
Microbacteriaceae
Micrococcaceae
Micrococcaceae
Micrococcaceae
Micrococcaceae

Micrococcaceae
Micrococcaceae
Micrococcaceae
Micrococcaceae
Micrococcaceae
Micrococcaceae
Micrococcaceae
Micrococcaceae
Micrococcaceae
Micrococcaceae
Micrococcaceae
Micrococcaceae
Micrococcaceae
Micrococcaceae
Micrococcaceae
Micromonosporaceae
Micromonosporaceae
Motilibacteraceae
Motilibacteraceae
Nocardiaceae
Nocardiaceae
Nocardiaceae
Nocardiaceae
Nocardiaceae
Nocardiaceae
Nocardioidaceae
Nocardioidaceae
Nocardioidaceae
Nocardioidaceae
Nocardioidaceae
Nocardioidaceae
Nocardioidaceae
Nocardioidaceae
Nocardioidaceae
Nocardioidaceae
Propionibacteriaceae
Propionibacteriaceae
Propionibacteriaceae
Propionibacteriaceae
Propionibacteriaceae
Propionibacteriaceae
Propionibacteriaceae
Propionibacteriaceae
Propionibacteriaceae
Pseudonocardiaceae
Pseudonocardiaceae

Pseudonocardiaceae
Pseudonocardiaceae
Pseudonocardiaceae
Pseudonocardiaceae
Ruaniaceae
Sanguibacteraceae
Sanguibacteraceae
Sanguibacteraceae
Sanguibacteraceae
Segniliparaceae
Sporichthyaceae
Sporichthyaceae
Streptomycetaceae
Streptomycetaceae
Streptomycetaceae
Streptomycetaceae
Streptosporangiaceae
Coriobacteriaceae
Conexibacteraceae
Thermoleophilaceae
Rhodothermaceae
Porphyromonadaceae
Cyclobacteriaceae
Cyclobacteriaceae
Cyclobacteriaceae
Cyclobacteriaceae
Cytophagaceae
Cytophagaceae
Cytophagaceae
Cytophagaceae
Cytophagaceae
Cytophagaceae
Cytophagaceae
Cytophagaceae
Flammeovirgaceae
Cryomorphaceae
Flavobacteriaceae
Flavobacteriaceae
Flavobacteriaceae
Flavobacteriaceae
Flavobacteriaceae
Flavobacteriaceae
Flavobacteriaceae
Flavobacteriaceae
Flavobacteriaceae
Flavobacteriaceae

Flavobacteriaceae
Flavobacteriaceae
Flavobacteriaceae
Flavobacteriaceae
Flavobacteriaceae
Flavobacteriaceae
Flavobacteriaceae
Flavobacteriaceae
Flavobacteriaceae
Flavobacteriaceae
Flavobacteriaceae
Flavobacteriaceae
Flavobacteriaceae
Flavobacteriaceae
Flavobacteriaceae
Flavobacteriaceae
Flavobacteriaceae
Flavobacteriaceae
Flavobacteriaceae
Flavobacteriaceae
Flavobacteriaceae
Flavobacteriaceae
Flavobacteriaceae
Chitinophagaceae
Sphingobacteriaceae
Sphingobacteriaceae
Sphingobacteriaceae
Sphingobacteriaceae
Sphingobacteriaceae
Sphingobacteriaceae
Sphingobacteriaceae
Sphingobacteriaceae
Sphingobacteriaceae
Sphingobacteriaceae
Sphingobacteriaceae
Sphingobacteriaceae
Sphingobacteriaceae
Sphingobacteriaceae
Waddliaceae
Ardenscatenaceae
Thermogemmatisporaceae
Sphaerobacteraceae
Gloeobacteraceae
Nostocaceae
Deinococcaceae
Bacillaceae

Bacillaceae
Bacillaceae
Bacillaceae
Bacillaceae
Bacillaceae
Bacillaceae
Bacillaceae
Bacillaceae
Bacillaceae
Bacillaceae
Bacillalesincertaesedis
Planococcaceae
Planococcaceae
Planococcaceae
Planococcaceae
Planococcaceae
Planococcaceae
Planococcaceae
Planococcaceae
Planococcaceae
Planococcaceae
Staphylococcaceae
Staphylococcaceae
Staphylococcaceae
Staphylococcaceae
Staphylococcaceae
Staphylococcaceae
Staphylococcaceae
Staphylococcaceae
Staphylococcaceae
Staphylococcaceae
Staphylococcaceae
Staphylococcaceae
Staphylococcaceae
Staphylococcaceae
Aerococcaceae
Aerococcaceae
Aerococcaceae
Aerococcaceae
Aerococcaceae
Carnobacteriaceae
Carnobacteriaceae
Carnobacteriaceae
Leuconostocaceae
Streptococcaceae
Streptococcaceae

Clostridiaceae
Clostridiaceae
Clostridiaceae
Clostridiaceae
Clostridiaceae
ClostridialesFamilyXII.IncertaeSedis
ClostridialesFamilyXIII.IncertaeSedis
Lachnospiraceae
Peptococcaceae
Peptostreptococcaceae
Peptostreptococcaceae
Ruminococcaceae
Syntrophomonadaceae
Natranaerobiaceae
Gemmatimonadaceae
Ignavibacteriaceae
Nitrospinaceae
Caulobacteraceae
Caulobacteraceae
Caulobacteraceae
Caulobacteraceae
Caulobacteraceae
Caulobacteraceae
Caulobacteraceae
Caulobacteraceae
Caulobacteraceae
Caulobacteraceae
Caulobacteraceae
Caulobacteraceae
Caulobacteraceae
Caulobacteraceae
Caulobacteraceae
Caulobacteraceae
Caulobacteraceae
Caulobacteraceae
Caulobacteraceae
Caulobacteraceae
Caulobacteraceae
Caulobacteraceae
Aurantimonadaceae
Aurantimonadaceae
Aurantimonadaceae
Aurantimonadaceae
Aurantimonadaceae
Beijerinckiaceae
Beijerinckiaceae

Beijerinckiaceae
Beijerinckiaceae
Bradyrhizobiaceae
Bradyrhizobiaceae
Bradyrhizobiaceae
Bradyrhizobiaceae
Brucellaceae
Brucellaceae
Brucellaceae
Brucellaceae
Hyphomicrobiaceae
Hyphomicrobiaceae
Hyphomicrobiaceae
Hyphomicrobiaceae
Hyphomicrobiaceae
Hyphomicrobiaceae
Hyphomicrobiaceae
Hyphomicrobiaceae
Hyphomicrobiaceae
Hyphomicrobiaceae
Hyphomicrobiaceae
Hyphomicrobiaceae
Hyphomicrobiaceae
Hyphomicrobiaceae
Hyphomicrobiaceae
Hyphomicrobiaceae
Hyphomicrobiaceae
Hyphomicrobiaceae
Methylobacteriaceae
Methylobacteriaceae
Methylobacteriaceae
Methylobacteriaceae
Methylobacteriaceae
Methylocystaceae
Methylocystaceae
Methylocystaceae
Phyllobacteriaceae
Phyllobacteriaceae
Phyllobacteriaceae
Phyllobacteriaceae
Phyllobacteriaceae
Phyllobacteriaceae
Phyllobacteriaceae
Phyllobacteriaceae
Phyllobacteriaceae
Phyllobacteriaceae

Phyllobacteriaceae
Phyllobacteriaceae
Phyllobacteriaceae
Phyllobacteriaceae
Phyllobacteriaceae
Phyllobacteriaceae
Phyllobacteriaceae
Rhizobiaceae
Rhizobiaceae
Rhizobiaceae
Rhizobiaceae
Rhizobiaceae
Rhizobiaceae
Rhizobiaceae
Rhizobiaceae
Rhizobiaceae
Rhizobiaceae
Rhizobiaceae
Rhizobiaceae
Rhodobiaceae
Rhodobiaceae
Xanthobacteraceae
unclassifiedRhizobiales
Hyphomonadaceae
Rhodobacteraceae
Rhodobacteraceae
Rhodobacteraceae
Rhodobacteraceae
Rhodobacteraceae
Rhodobacteraceae
Rhodobacteraceae
Rhodobacteraceae
Rhodobacteraceae
Rhodobacteraceae
Rhodobacteraceae
Rhodobacteraceae
Rhodobacteraceae
Rhodobacteraceae
Rhodobacteraceae
Rhodobacteraceae
Rhodobacteraceae
Rhodobacteraceae
Rhodobacteraceae
Rhodobacteraceae
Rhodobacteraceae
Rhodobacteraceae

Rhodobacteraceae
Rhodobacteraceae
Rhodobacteraceae
Rhodobacteraceae
Rhodobacteraceae
Rhodobacteraceae
Rhodobacteraceae
Rhodobacteraceae
Rhodobacteraceae
Rhodobacteraceae
Rhodobacteraceae
Rhodobacteraceae
Rhodobacteraceae
Rhodobacteraceae
Acetobacteraceae
Acetobacteraceae
Acetobacteraceae
Acetobacteraceae
Acetobacteraceae
Acetobacteraceae
Rhodospirillaceae
Rickettsiaceae
Erythrobacteraceae
Erythrobacteraceae
Erythrobacteraceae
Erythrobacteraceae
Erythrobacteraceae
Erythrobacteraceae
Erythrobacteraceae
Erythrobacteraceae
Erythrobacteraceae
Erythrobacteraceae
Erythrobacteraceae
Erythrobacteraceae
Erythrobacteraceae
Erythrobacteraceae
Erythrobacteraceae
Sphingomonadaceae
Sphingomonadaceae
Sphingomonadaceae
Sphingomonadaceae
Sphingomonadaceae
Sphingomonadaceae
Sphingomonadaceae
Sphingomonadaceae
Sphingomonadaceae

Sphingomonadaceae
Sphingomonadaceae
Sphingomonadaceae
Sphingomonadaceae
Sphingomonadaceae
Sphingomonadaceae
Sphingomonadaceae
Sphingomonadaceae
Sphingomonadaceae
Sphingomonadaceae
Sphingomonadaceae
Sphingomonadaceae
Sphingomonadaceae
Sphingomonadaceae
Sphingomonadaceae
Sphingomonadaceae
Sphingomonadaceae
Sphingomonadaceae
Sphingomonadaceae
Sphingomonadaceae
Sphingomonadaceae
Sphingomonadaceae
Sphingomonadaceae
Sphingomonadaceae
Sphingomonadaceae
Sphingomonadaceae
Sphingomonadaceae
Sphingomonadaceae
Sphingomonadaceae
Sphingomonadaceae
Sphingomonadaceae
Sphingomonadaceae
Alcaligenaceae
Alcaligenaceae
Alcaligenaceae
Alcaligenaceae
Alcaligenaceae
Alcaligenaceae
Alcaligenaceae
Alcaligenaceae
Alcaligenaceae
Burkholderiaceae
Burkholderiaceae
Comamonadaceae
Comamonadaceae
Comamonadaceae

Comamonadaceae
Comamonadaceae
Comamonadaceae
Comamonadaceae
Comamonadaceae
Comamonadaceae
Comamonadaceae
Comamonadaceae
Comamonadaceae
Comamonadaceae
Comamonadaceae
Comamonadaceae
Comamonadaceae
Comamonadaceae
Comamonadaceae
Comamonadaceae
Comamonadaceae
Comamonadaceae
Comamonadaceae
Oxalobacteraceae
Oxalobacteraceae
Oxalobacteraceae
Oxalobacteraceae
Oxalobacteraceae
Oxalobacteraceae
Oxalobacteraceae
Oxalobacteraceae
Oxalobacteraceae
unclassifiedBurkholderiales
unclassifiedBurkholderiales
unclassifiedBurkholderiales
Neisseriaceae
Neisseriaceae
Rhodocyclaceae
Bacteriovoracaceae
Bacteriovoracaceae
Bdellovibrionaceae
Bdellovibrionaceae
Bdellovibrionaceae
Desulfobulbaceae
Polyangiaceae
Campylobacteraceae
Alteromonadaceae
Alteromonadaceae
Alteromonadaceae
Alteromonadalesgeneraincertaesedis

Idiomarinaceae
Idiomarinaceae
Idiomarinaceae
Shewanellaceae
Chromatiaceae
Chromatiaceae
Chromatiaceae
Enterobacteriaceae
Enterobacteriaceae
Enterobacteriaceae
Enterobacteriaceae
Legionellaceae
Legionellaceae
Methylococcaceae
Methylococcaceae
Methylococcaceae
Alcanivoracaceae
Hahellaceae
Hahellaceae
Halomonadaceae
Oceanospirillaceae
Oceanospirillaceae
Saccharospirillaceae
Pasteurellaceae
Pasteurellaceae
Moraxellaceae
Moraxellaceae
Moraxellaceae
Moraxellaceae
Moraxellaceae
Moraxellaceae
Moraxellaceae
Moraxellaceae
Moraxellaceae
Moraxellaceae
Moraxellaceae
Pseudomonadaceae
Pseudomonadaceae
Pseudomonadaceae
Pseudomonadaceae
Pseudomonadaceae
Pseudomonadaceae
Pseudomonadaceae
Pseudomonadaceae
Pseudomonadaceae
Pseudomonadaceae

Pseudomonadaceae
Pseudomonadaceae
Pseudomonadaceae
Pseudomonadaceae
Pseudomonadaceae
Pseudomonadaceae
Pseudomonadaceae
Pseudomonadaceae
Pseudomonadaceae
Piscirickettsiaceae
Piscirickettsiaceae
Piscirickettsiaceae
Sinobacteraceae
Sinobacteraceae
Xanthomonadaceae
Xanthomonadaceae
Xanthomonadaceae
Xanthomonadaceae
Xanthomonadaceae
Xanthomonadaceae
Xanthomonadaceae
Xanthomonadaceae
Xanthomonadaceae
Xanthomonadaceae
Xanthomonadaceae
Xanthomonadaceae
Xanthomonadaceae
Xanthomonadaceae
Xanthomonadaceae
Xanthomonadaceae
Xanthomonadaceae
Xanthomonadaceae
Xanthomonadaceae
Xanthomonadaceae
Xanthomonadaceae
Xanthomonadaceae
Xanthomonadaceae
Xanthomonadaceae
Xanthomonadaceae
Xanthomonadaceae
Xanthomonadaceae
Xanthomonadaceae
Xanthomonadaceae
Xanthomonadaceae
Xanthomonadaceae
Xanthomonadaceae

Xanthomonadaceae
unclassifiedGammaproteobacteria
Brachyspiraceae
Spirochaetaceae
Acholeplasmataceae
Acholeplasmataceae
Acholeplasmataceae
Mycoplasmataceae
Puniceicoccaceae
Verrucomicrobiaceae
Solibacteraceae
Beutenbergiaceae
Brevibacteriaceae
Brevibacteriaceae
Brevibacteriaceae
Brevibacteriaceae
Brevibacteriaceae
Brevibacteriaceae
Cellulomonadaceae
Cellulomonadaceae
Corynebacteriaceae
Corynebacteriaceae
Corynebacteriaceae
Corynebacteriaceae
Corynebacteriaceae
Corynebacteriaceae
Corynebacteriaceae
Corynebacteriaceae
Corynebacteriaceae
Demequinaceae
Demequinaceae
Dermacoccaceae
Dietziaceae
Dietziaceae
Frankiaceae
Geodermatophilaceae
Gordoniaceae
Gordoniaceae
Gordoniaceae
Gordoniaceae
Gordoniaceae
Gordoniaceae
Gordoniaceae
Intrasporangiaceae
Intrasporangiaceae
Jonesiaceae

Jonesiaceae
Kineosporiaceae
Kineosporiaceae
Microbacteriaceae
Microbacteriaceae
Microbacteriaceae
Microbacteriaceae
Microbacteriaceae
Microbacteriaceae
Microbacteriaceae
Microbacteriaceae
Microbacteriaceae
Microbacteriaceae
Microbacteriaceae
Microbacteriaceae
Microbacteriaceae
Microbacteriaceae
Microbacteriaceae
Micrococcaceae
Micrococcaceae
Micrococcaceae
Micrococcaceae
Micrococcaceae
Micrococcaceae
Micrococcaceae
Nakamurellaceae
Nocardioidaceae
Nocardioidaceae
Nocardioidaceae
Nocardioidaceae
Nocardioidaceae
Nocardioidaceae
Nocardioidaceae
Nocardioidaceae
Nocardiopsaceae
Nocardiopsaceae
Nocardiopsaceae
Nocardiopsaceae
Nocardiopsaceae
Nocardiopsaceae
Promicromonosporaceae
Promicromonosporaceae
Promicromonosporaceae
Propionibacteriaceae
Pseudonocardiaceae
Pseudonocardiaceae

Pseudonocardiaceae
Pseudonocardiaceae
Pseudonocardiaceae
Pseudonocardiaceae
Pseudonocardiaceae
Pseudonocardiaceae
Sanguibacteraceae
Streptomycetaceae
Streptomycetaceae
Streptomycetaceae
Bifidobacteriaceae
Patulibacteraceae
Bacteroidaceae
Bacteroidaceae
Bacteroidaceae
Porphyromonadaceae
Porphyromonadaceae
Porphyromonadaceae
Prevotellaceae
Prevotellaceae
Prevotellaceae
Prevotellaceae
Prevotellaceae
Cytophagaceae
Cytophagaceae
Cryomorphaceae
Flavobacteriaceae
Flavobacteriaceae
Flavobacteriaceae
Flavobacteriaceae
Flavobacteriaceae
Flavobacteriaceae
Flavobacteriaceae
Flavobacteriaceae
Sphingobacteriaceae
Sphingobacteriaceae
Sphingobacteriaceae
Sphingobacteriaceae
Sphingobacteriaceae
Sphingobacteriaceae
Sphingobacteriaceae
Sphingobacteriaceae
Sphingobacteriaceae
Criblamydiaceae
Nostocaceae
Phormidiaceae

Deinococcaceae
Fibrobacteraceae
Alicyclobacillaceae
Bacillaceae
Bacillaceae
Bacillaceae
Bacillaceae
Bacillaceae
Bacillaceae
Bacillaceae
Bacillaceae
Bacillaceae
Bacillaceae
Bacillaceae
Bacillaceae
Bacillaceae
Bacillaceae
Bacillaceae
Bacillaceae
Bacillalesincertaesedis
Bacillalesincertaesedis
Paenibacillaceae
Paenibacillaceae
Paenibacillaceae
Planococcaceae
Planococcaceae
Planococcaceae
Planococcaceae
Planococcaceae
Planococcaceae
Planococcaceae
Planococcaceae
Planococcaceae
Planococcaceae
Planococcaceae
Planococcaceae
Planococcaceae
Planococcaceae
Planococcaceae
Staphylococcaceae
Staphylococcaceae
Staphylococcaceae
Staphylococcaceae
Staphylococcaceae
Staphylococcaceae
Staphylococcaceae

Staphylococcaceae
Staphylococcaceae
Staphylococcaceae
Staphylococcaceae
Thermoactinomycetaceae
Aerococcaceae
Aerococcaceae
Aerococcaceae
Aerococcaceae
Carnobacteriaceae
Carnobacteriaceae
Carnobacteriaceae
Carnobacteriaceae
Carnobacteriaceae
Carnobacteriaceae
Enterococcaceae
Enterococcaceae
Enterococcaceae
Lactobacillaceae
Lactobacillaceae
Leuconostocaceae
Streptococcaceae
Streptococcaceae
Streptococcaceae
Streptococcaceae
Clostridiaceae
Clostridiaceae
ClostridialesFamilyXI.IncertaeSedis
ClostridialesFamilyXI.IncertaeSedis
ClostridialesFamilyXI.IncertaeSedis
ClostridialesFamilyXI.IncertaeSedis
ClostridialesFamilyXI.IncertaeSedis
ClostridialesFamilyXI.IncertaeSedis
ClostridialesFamilyXI.IncertaeSedis
ClostridialesFamilyXI.IncertaeSedis
ClostridialesFamilyXI.IncertaeSedis
Lachnospiraceae
Lachnospiraceae
Lachnospiraceae
Lachnospiraceae
Lachnospiraceae
Peptoniphilaceae
Peptoniphilaceae
Peptoniphilaceae
Peptoniphilaceae
Peptoniphilaceae

Peptostreptococcaceae
Ruminococcaceae
Ruminococcaceae
unclassifiedClostridiales
Erysipelotrichaceae
Veillonellaceae
Nitrospiraceae
Magnetococcaceae
Aurantimonadaceae
Bartonellaceae
Hyphomicrobiaceae
Hyphomicrobiaceae
Phyllobacteriaceae
Phyllobacteriaceae
Phyllobacteriaceae
Rhizobiaceae
Rhizobiaceae
Rhizobiaceae
Xanthobacteraceae
Xanthobacteraceae
unclassifiedRhizobiales
unclassifiedRhizobiales
Rhodobacteraceae
Rhodobacteraceae
Rhodobacteraceae
Rhodobacteraceae
Rhodobacteraceae
Rhodobacteraceae
Sphingomonadaceae
Sphingomonadaceae
Sphingomonadaceae
Sphingomonadaceae
Sphingomonadaceae
Sphingomonadaceae
Alcaligenaceae
Alcaligenaceae
Alcaligenaceae
Alcaligenaceae
Alcaligenaceae
Alcaligenaceae
Alcaligenaceae
Alcaligenaceae
Burkholderiaceae
Burkholderiaceae
Comamonadaceae
Comamonadaceae

Comamonadaceae
Comamonadaceae
Comamonadaceae
Comamonadaceae
Comamonadaceae
Comamonadaceae
Comamonadaceae
Comamonadaceae
Oxalobacteraceae
Oxalobacteraceae
Oxalobacteraceae
Oxalobacteraceae
Oxalobacteraceae
Oxalobacteraceae
Oxalobacteraceae
Oxalobacteraceae
Oxalobacteraceae
Hydrogenophilaceae
Neisseriaceae
Neisseriaceae
Rhodocyclaceae
Rhodocyclaceae
Rhodocyclaceae
Desulfobacteraceae
Desulfovibrionaceae
Geobacteraceae
Pelobacteraceae
Syntrophaceae
Syntrophobacteraceae
Campylobacteraceae
Campylobacteraceae
Campylobacteraceae
Campylobacteraceae
Campylobacteraceae
Helicobacteraceae
Aeromonadaceae
Aeromonadaceae
Alteromonadaceae
Alteromonadaceae
Alteromonadaceae
Alteromonadaceae
Alteromonadalesgeneraincertaesedis
Idiomarinaceae
Ectothiorhodospiraceae
Halothiobacillaceae
Enterobacteriaceae

Enterobacteriaceae
Enterobacteriaceae
Enterobacteriaceae
Enterobacteriaceae
Enterobacteriaceae
Enterobacteriaceae
Enterobacteriaceae
Enterobacteriaceae
Enterobacteriaceae
Enterobacteriaceae
Enterobacteriaceae
Enterobacteriaceae
Legionellaceae
Legionellaceae
Methylothermaceae
Alcanivoracaceae
Hahellaceae
Halomonadaceae
Halomonadaceae
Oceanospirillaceae
Oceanospirillaceae
Orbaceae
Pasteurellaceae
Pasteurellaceae
Pasteurellaceae
Pasteurellaceae
Pasteurellaceae
Moraxellaceae
Moraxellaceae
Moraxellaceae
Moraxellaceae
Moraxellaceae
Moraxellaceae
Moraxellaceae
Moraxellaceae
Moraxellaceae
Moraxellaceae
Pseudomonadaceae
Pseudomonadaceae
Pseudomonadaceae
Pseudomonadaceae
Pseudomonadaceae
Pseudomonadaceae
Pseudomonadaceae
Pseudomonadaceae
Pseudomonadaceae

Pseudomonadaceae
Pseudomonadaceae
Pseudomonadaceae
Pseudomonadaceae
Pseudomonadaceae
Pseudomonadaceae
Pseudomonadaceae
Pseudomonadaceae
Pseudomonadaceae
Piscirickettsiaceae
Thiotrichaceae
Thiotrichaceae
Pseudoalteromonadaceae
Vibrionaceae
Xanthomonadaceae
Xanthomonadaceae
Spirochaetaceae
Acholeplasmataceae
Corynebacteriaceae
Corynebacteriaceae
Dermabacteraceae
Microbacteriaceae
Microbacteriaceae
Micrococcaceae
Micrococcaceae
Micrococcaceae
Nocardioidaceae
Nocardioidaceae
Nocardioidaceae
Nocardioidaceae
Bacillaceae
Planococcaceae
Staphylococcaceae
Carnobacteriaceae
Enterococcaceae
Eubacteriaceae
Eubacteriaceae
Peptostreptococcaceae
Rhodobacteraceae
Rhodobacteraceae
Rhodobacteraceae
Rhodobacteraceae
Rhodobacteraceae
Rhodobacteraceae
Sphingomonadaceae
Alcaligenaceae

Alcaligenaceae
Comamonadaceae
Acidithiobacillaceae
Aeromonadaceae
Alteromonadaceae
Alteromonadaceae
Idiomarinaceae
Shewanellaceae
Ectothiorhodospiraceae
Enterobacteriaceae
Halomonadaceae
Pseudomonadaceae
unclassifiedGammaproteobacteria
Bogoriellaceae
Corynebacteriaceae
Dermabacteraceae
Dietziaceae
Dietziaceae
Glycomycetaceae
Intrasporangiaceae
Intrasporangiaceae
Intrasporangiaceae
Microbacteriaceae
Microbacteriaceae
Microbacteriaceae
Micrococcaceae
Micrococcaceae
Micrococcaceae
Micrococcaceae
Mycobacteriaceae
Nakamurellaceae
Nocardiaceae
Nocardioidaceae
Nocardiopsaceae
Nocardiopsaceae
Promicromonosporaceae
Promicromonosporaceae
Propionibacteriaceae
Propionibacteriaceae
Propionibacteriaceae
Pseudonocardiaceae
Ruaniaceae
Ruaniaceae
Streptomycetaceae
Streptomycetaceae
Cytophagaceae

Flavobacteriaceae
Flavobacteriaceae
Flavobacteriaceae
Flavobacteriaceae
Flavobacteriaceae
Sphingobacteriaceae
Bacillaceae
Bacillaceae
Bacillaceae
Planococcaceae
Aerococcaceae
Carnobacteriaceae
Carnobacteriaceae
Enterococcaceae
Enterococcaceae
Enterococcaceae
Leuconostocaceae
Catabacteriaceae
Eubacteriaceae
Ruminococcaceae
Leptotrichiaceae
Rhodobacteraceae
Rhodobacteraceae
Rhodobacteraceae
Sphingomonadaceae
Sphingomonadaceae
Campylobacteraceae
Moraxellaceae
Pseudomonadaceae
Piscirickettsiaceae
Piscirickettsiaceae
Piscirickettsiaceae
Xanthomonadaceae
unclassifiedGammaproteobacteria
Dethiosulfovibrionaceae
Synergistaceae
Synergistaceae
Actinomycetaceae
Actinomycetaceae
Actinomycetaceae
Actinomycetaceae
Actinomycetaceae
Corynebacteriaceae
Corynebacteriaceae
Corynebacteriaceae
Corynebacteriaceae

Dermacoccaceae
Dietziaceae
Dietziaceae
Microbacteriaceae
Microbacteriaceae
Microbacteriaceae
Micrococcaceae
Micrococcaceae
Micrococcaceae
Micrococcaceae
Nocardioidaceae
Nocardiopsaceae
Propionibacteriaceae
Pseudonocardiaceae
Pseudonocardiaceae
Patulibacteraceae
Solirubrobacteraceae
Porphyromonadaceae
Prevotellaceae
Sphingobacteriaceae
Bacillaceae
Bacillaceae
Bacillalesincertaesedis
Bacillalesincertaesedis
Bacillalesincertaesedis
Bacillalesincertaesedis
Listeriaceae
Paenibacillaceae
Planococcaceae
Planococcaceae
Staphylococcaceae
Staphylococcaceae
Staphylococcaceae
Staphylococcaceae
Staphylococcaceae
Thermoactinomycetaceae
Aerococcaceae
Carnobacteriaceae
Lactobacillaceae
Lactobacillaceae
Lactobacillaceae
Streptococcaceae
Streptococcaceae
Streptococcaceae
Streptococcaceae
Streptococcaceae

Streptococcaceae
Streptococcaceae
Streptococcaceae
ClostridialesFamilyXI.IncertaeSedis
ClostridialesFamilyXI.IncertaeSedis
ClostridialesFamilyXI.IncertaeSedis
ClostridialesFamilyXIII.IncertaeSedis
ClostridialesFamilyXIII.IncertaeSedis
Lachnospiraceae
Lachnospiraceae
Peptoniphilaceae
Peptostreptococcaceae
Peptostreptococcaceae
Ruminococcaceae
Ruminococcaceae
Veillonellaceae
Acidaminococcaceae
Veillonellaceae
Veillonellaceae
Veillonellaceae
Veillonellaceae
Veillonellaceae
Veillonellaceae
Caulobacteraceae
Caulobacteraceae
Caulobacteraceae
Hyphomicrobiaceae
Methylobacteriaceae
Rhodobacteraceae
Alcaligenaceae
Burkholderiaceae
Oxalobacteraceae
unclassifiedBurkholderiales
Hydrogenophilaceae
Neisseriaceae
Neisseriaceae
Campylobacteraceae
Pasteurellaceae
Moraxellaceae
Moraxellaceae
Moraxellaceae
Vibrionaceae
Xanthomonadaceae
Holophagaceae
Actinomycetaceae
Actinomycetaceae

Actinomycetaceae
Actinomycetaceae
Bogoriellaceae
Brevibacteriaceae
Corynebacteriaceae
Corynebacteriaceae
Corynebacteriaceae
Dermabacteraceae
Geodermatophilaceae
Intrasporangiaceae
Kineosporiaceae
Kineosporiaceae
Kineosporiaceae
Kineosporiaceae
Kineosporiaceae
Microbacteriaceae
Microbacteriaceae
Microbacteriaceae
Microbacteriaceae
Microbacteriaceae
Microbacteriaceae
Micrococcaceae
Micrococcaceae
Micrococcaceae
Nocardiaceae
Nocardiaceae
Nocardioidaceae
Nocardioidaceae
Nocardioidaceae
Nocardioidaceae
Promicromonosporaceae
Propionibacteriaceae
Propionibacteriaceae
Pseudonocardiaceae
Pseudonocardiaceae
Streptomycetaceae
Gaiellaceae
Bacteroidaceae
Prevotellaceae
Cytophagaceae
Cytophagaceae
Cytophagaceae
Flavobacteriaceae
Flavobacteriaceae
Flavobacteriaceae
Flavobacteriaceae

Flavobacteriaceae
Saprospiraceae
Sphingobacteriaceae
Dehalococcoidaceae
unclassifiedDehalococcoidia
Fibrobacteraceae
Fibrobacteraceae
Fibrobacteraceae
Bacillaceae
Bacillaceae
Bacillaceae
Bacillaceae
Bacillaceae
Bacillaceae
Paenibacillaceae
Paenibacillaceae
Planococcaceae
Planococcaceae
Planococcaceae
Planococcaceae
Staphylococcaceae
Staphylococcaceae
Thermoactinomycetaceae
Thermoactinomycetaceae
Aerococcaceae
Aerococcaceae
Aerococcaceae
Carnobacteriaceae
Enterococcaceae
Enterococcaceae
Leuconostocaceae
Leuconostocaceae
Streptococcaceae
Christensenellaceae
Eubacteriaceae
Gracilibacteraceae
Lachnospiraceae
Lachnospiraceae
Lachnospiraceae
Lachnospiraceae
Oscillospiraceae
Peptoniphilaceae
Proteinivoraceae
Ruminococcaceae
Ruminococcaceae
Ruminococcaceae

Fusobacteriaceae
Beijerinckiaceae
Brucellaceae
Brucellaceae
Methylobacteriaceae
Methylobacteriaceae
Methylobacteriaceae
Methylobacteriaceae
Methylobacteriaceae
Rhodobacteraceae
Rhodobacteraceae
Sphingomonadaceae
Sphingomonadaceae
Sphingomonadaceae
Sphingomonadaceae
Sphingomonadaceae
Alcaligenaceae
Alcaligenaceae
Alcaligenaceae
Alcaligenaceae
Comamonadaceae
Oxalobacteraceae
Sutterellaceae
Neisseriaceae
Nitrosomonadaceae
Myxococcaceae
Succinivibrionaceae
Idiomarinaceae
Idiomarinaceae
Pseudoalteromonadaceae
Psychromonadaceae
Shewanellaceae
Arenicellaceae
Chromatiaceae
Chromatiaceae
Chromatiaceae
Chromatiaceae
Chromatiaceae
Thioalkalispiraceae
Enterobacteriaceae
Enterobacteriaceae
Coxiellaceae
Halomonadaceae
Halomonadaceae
Halomonadaceae
Pseudomonadaceae

Thiotrichaceae
Xanthomonadaceae
Spirochaetaceae
Acidimicrobiaceae
Iamiaceae
Acidothermaceae
Actinomycetaceae
Actinopolysporaceae
Catenulisporaceae
Geodermatophilaceae
Intrasporangiaceae
Intrasporangiaceae
Jiangellaceae
Microbacteriaceae
Microbacteriaceae
Microbacteriaceae
Micromonosporaceae
Mycobacteriaceae
Nocardioidaceae
Nocardioidaceae
Nocardioidaceae
Nocardioidaceae
Nocardioidaceae
Nocardiopsaceae
Nocardiopsaceae
Nocardiopsaceae
Nocardiopsaceae
Nocardiopsaceae
Nocardiopsaceae
Nocardiopsaceae
Promicromonosporaceae
Promicromonosporaceae
Pseudonocardiaceae
Pseudonocardiaceae
Pseudonocardiaceae
Pseudonocardiaceae
Pseudonocardiaceae
Pseudonocardiaceae
Pseudonocardiaceae
Pseudonocardiaceae
Pseudonocardiaceae
Streptomycetaceae
Streptomycetaceae
Streptomycetaceae
Streptomycetaceae
Streptomycetaceae

Thermomonosporaceae
Gaiellaceae
Rubrobacteraceae
Solirubrobacteraceae
Marinilabiliaceae
Rikenellaceae
Flavobacteriaceae
Flavobacteriaceae
Rhabdochlamydiaceae
Herpetosiphonaceae
Bacillaceae
Bacillaceae
Bacillaceae
Bacillaceae
Bacillaceae
Bacillaceae
Bacillaceae
Bacillaceae
Bacillaceae
Bacillaceae
Bacillaceae
Paenibacillaceae
Paenibacillaceae
Planococcaceae
Thermoactinomycetaceae
Thermoactinomycetaceae
Thermoactinomycetaceae
Thermoactinomycetaceae
Thermoactinomycetaceae
Enterococcaceae
Leuconostocaceae
Clostridiaceae
ClostridialesFamilyXII.IncertaeSedis
Eubacteriaceae
Lachnospiraceae
Ruminococcaceae
Ruminococcaceae
Ruminococcaceae
Nitrospiraceae
Bradyrhizobiaceae
Bradyrhizobiaceae
Bradyrhizobiaceae
Hyphomicrobiaceae
Phyllobacteriaceae
Rhizobiaceae
unclassifiedRhizobiales

Rhodobacteraceae
Rhodospirillaceae
Holosporaceae
Geminicoccus
Hydrogenophilaceae
Chromobacteriaceae
Desulfonatronumaceae
Phaselicystidaceae
Enterobacteriaceae
Enterobacteriaceae
Enterobacteriaceae
Xanthomonadaceae
Frankiaceae
Glycomycetaceae
Propionibacteriaceae
Pseudonocardiaceae
Sanguibacteraceae
Streptomycetaceae
Sphingobacteriaceae
Lactobacillaceae
Polyangiaceae
Enterobacteriaceae
Enterobacteriaceae
Pseudomonadaceae
Xanthomonadaceae
Coriobacteriaceae
Cytophagaceae
Flavobacteriaceae
Flavobacteriaceae
Flavobacteriaceae
Flavobacteriaceae
Flavobacteriaceae
Flavobacteriaceae
Flavobacteriaceae
Ardenticatenaceae
Bacillaceae
Paenibacillaceae
Aurantimonadaceae
Phyllobacteriaceae
Rhodobacteraceae
Sphingomonadaceae
Sphingomonadaceae
Sphingomonadaceae
Oxalobacteraceae
unclassifiedBurkholderiales
Hydrogenophilaceae

Sandaracinaceae
Chromatiaceae
Enterobacteriaceae
Enterobacteriaceae
Enterobacteriaceae
Enterobacteriaceae
Oceanospirillaceae
Pseudomonadaceae
Pseudomonadaceae
Piscirickettsiaceae
Sinobacteraceae
Xanthomonadaceae
Opitutaceae
Geodermatophilaceae
Kineosporiaceae
Microbacteriaceae
Microbacteriaceae
Micrococcaceae
Micrococcaceae
Promicromonosporaceae
Sporichthyaceae
Patulibacteraceae
Cytophagaceae
Cryomorphaceae
Flavobacteriaceae
Flavobacteriaceae
Flavobacteriaceae
Flavobacteriaceae
Flavobacteriaceae
Flavobacteriaceae
Rhabdochlamydiaceae
Paenibacillaceae
Staphylococcaceae
Thermoactinomycetaceae
Carnobacteriaceae
Aurantimonadaceae
Bradyrhizobiaceae
Brucellaceae
Brucellaceae
Phyllobacteriaceae
Sphingomonadaceae
Sphingomonadaceae
Sphingomonadaceae
Alcaligenaceae
Alcaligenaceae
Alcaligenaceae

Comamonadaceae
Comamonadaceae
Comamonadaceae
Comamonadaceae
Oxalobacteraceae
Oxalobacteraceae
Bacteriovoracaceae
Cystobacteraceae
Alteromonadaceae
Alteromonadalesgeneraincertaesedis
Enterobacteriaceae
Legionellaceae
Halomonadaceae
Halomonadaceae
Halomonadaceae
Pseudomonadaceae
Pseudomonadaceae
Pseudomonadaceae
Pseudomonadaceae
Pseudomonadaceae
Vibrionaceae
Xanthomonadaceae
Xanthomonadaceae
Bryobacter
Iamiaceae
Catenulisporaceae
Cryptosporangiaceae
Geodermatophilaceae
Geodermatophilaceae
Intrasporangiaceae
Intrasporangiaceae
Jiangellaceae
Kineosporiaceae
Microbacteriaceae
Micrococcaceae
Micromonosporaceae
Nakamurellaceae
Nocardiaceae
Nocardioidaceae
Nocardiopsaceae
Nocardiopsaceae
Pseudonocardiaceae
Pseudonocardiaceae
Pseudonocardiaceae
Pseudonocardiaceae
Streptomycetaceae

Streptomycetaceae
unclassifiedPseudonocardineae
Coriobacteriaceae
Armatimonadaceae
Flavobacteriaceae
Sphingobacteriaceae
Roseiflexaceae
Spirulinaceae
unclassifiedOscillatoriales
unclassifiedOscillatoriales
Bacillaceae
Bacillaceae
Bacillaceae
Bacillaceae
Bacillaceae
Paenibacillaceae
Paenibacillaceae
Paenibacillaceae
Paenibacillaceae
Planococcaceae
Leuconostocaceae
Clostridiaceae
Clostridiaceae
Clostridiaceae
ClostridialesFamilyXII.IncertaeSedis
Eubacteriaceae
Lachnospiraceae
Lachnospiraceae
Lachnospiraceae
Peptoniphilaceae
Parvularculaceae
Beijerinckiaceae
Hyphomicrobiaceae
Methylobacteriaceae
Methylobacteriaceae
Rhodobacteraceae
Rhodospirillaceae
Rhodospirillaceae
Sphingomonadaceae
Sphingomonadaceae
Burkholderiaceae
Comamonadaceae
Comamonadaceae
Comamonadaceae
Comamonadaceae
Oxalobacteraceae

Hydrogenophilaceae
Methylophilaceae
Methylophilaceae
Rhodocyclaceae
Rhodocyclaceae
Desulfuromonadaceae
Geobacteraceae
Anaeromyxobacteraceae
Kofleriaceae
Myxococcaceae
Alteromonadaceae
Alteromonadaceae
Enterobacteriaceae
Enterobacteriaceae
Enterobacteriaceae
Sinobacteraceae
Xanthomonadaceae
Chthoniobacter
Micrococcaceae
Pseudonocardiaceae
Marinilabiliaceae
Flavobacteriaceae
Flavobacteriaceae
Flavobacteriaceae
Lachnospiraceae
Oscillospiraceae
Oligosphaeraceae
Alcaligenaceae
Comamonadaceae
Rhodocyclaceae
Campylobacteraceae
Alcanivoracaceae
Thiotrichaceae
Xanthomonadaceae
Xanthomonadaceae
Actinomycetaceae
Corynebacteriaceae
Intrasporangiaceae
Propionibacteriaceae
Rhizobiaceae
Rhodobacteraceae
Methylophilaceae
Desulfovibrionaceae
Cardiobacteriaceae
Xanthomonadaceae
Xanthomonadaceae

Mariprofundaceae
Spirochaetaceae
Beutenbergiaceae
Microbacteriaceae
Microbacteriaceae
Micrococcaceae
Cytophagaceae
Caldilineaceae
Carnobacteriaceae
Carnobacteriaceae
Carnobacteriaceae
Enterococcaceae
Enterococcaceae
Hyphomicrobiaceae
Hyphomicrobiaceae
Hyphomicrobiaceae
Rhodobacteraceae
Rhodobacteraceae
Sphingomonadaceae
Alcaligenaceae
Alcaligenaceae
Comamonadaceae
Comamonadaceae
Comamonadaceae
Neisseriaceae
Thermithiobacillaceae
Ectothiorhodospiraceae
Enterobacteriaceae
Enterobacteriaceae
Enterobacteriaceae
Legionellaceae
Methylothermaceae
Oceanospirillaceae
Moraxellaceae
Pseudomonadaceae
Acholeplasmataceae
Geodermatophilaceae
Jonesiaceae
Microbacteriaceae
Nocardiaceae
Nocardioidaceae
Pseudonocardiaceae
Ruaniaceae
Sanguibacteraceae
Streptomycetaceae
Streptomycetaceae

Nitriliruptoraceae
Bacteroidaceae
Flavobacteriaceae
Planococcaceae
Victivallaceae
Caulobacteraceae
Caulobacteraceae
Caulobacteraceae
Bartonellaceae
Hyphomicrobiaceae
Methylobacteriaceae
Phyllobacteriaceae
Rhizobiaceae
Xanthobacteraceae
unclassifiedRhizobiales
Rhodobacteraceae
Rhodobacteraceae
Acetobacteraceae
Sphingomonadaceae
Sphingomonadaceae
Alcaligenaceae
Oxalobacteraceae
Chromobacteriaceae
Neisseriaceae
Alteromonadaceae
Alteromonadaceae
Alteromonadaceae
Pseudoalteromonadaceae
Halomonadaceae
Pasteurellaceae
unclassifiedPseudomonadales
Xanthomonadaceae
Xanthomonadaceae
OMGgroup
Opitutaceae
Brevibacteriaceae
Corynebacteriaceae
Demequinaceae
Dermabacteraceae
Geodermatophilaceae
Intrasporangiaceae
Microbacteriaceae
Microbacteriaceae
Microbacteriaceae
Microbacteriaceae
Microbacteriaceae

Microbacteriaceae
Micrococcaceae
Micrococcaceae
Micrococcaceae
Micrococcaceae
Micrococcaceae
Streptosporangiaceae
Hydrogenothermaceae
Cytophagaceae
Flavobacteriaceae
Flavobacteriaceae
Flavobacteriaceae
Flavobacteriaceae
Flavobacteriaceae
Sphingobacteriaceae
Pseudanabaenaceae
Bacillaceae
Bacillaceae
Bacillaceae
Planococcaceae
Staphylococcaceae
Staphylococcaceae
Staphylococcaceae
Staphylococcaceae
Aerococcaceae
Aerococcaceae
Carnobacteriaceae
Enterococcaceae
Streptococcaceae
Streptococcaceae
Streptococcaceae
Streptococcaceae
Streptococcaceae
Streptococcaceae
Streptococcaceae
Streptococcaceae
Streptococcaceae
Streptococcaceae
Streptococcaceae
Clostridiaceae
Peptostreptococcaceae
Erysipelotrichaceae
Caulobacteraceae
Phyllobacteriaceae
Rhodobiaceae
Rhodobacteraceae

Burkholderiaceae
Burkholderiaceae
Methylophilaceae
Methylophilaceae
Chromobacteriaceae
Neisseriaceae
Neisseriaceae
Neisseriaceae
Neisseriaceae
Neisseriaceae
Neisseriaceae
Neisseriaceae
Neisseriaceae
Neisseriaceae
Neisseriaceae
Rhodocyclaceae
Rhodocyclaceae
Bacteriovoracaceae
Campylobacteraceae
Cardiobacteriaceae
Enterobacteriaceae
Enterobacteriaceae
Enterobacteriaceae
Legionellaceae
Oceanospirillaceae
Pasteurellaceae
Pasteurellaceae
Pasteurellaceae
Pasteurellaceae
Pasteurellaceae
Pasteurellaceae
Pasteurellaceae
Pasteurellaceae
Moraxellaceae
Moraxellaceae
Xanthomonadaceae
Spirochaetaceae
Spirochaetaceae
Acholeplasmataceae
Acholeplasmataceae
Spiroplasmataceae
Corynebacteriaceae
Geodermatophilaceae
Geodermatophilaceae
Jonesiaceae
Jonesiaceae

Nocardioidaceae
Pseudonocardiaceae
Sanguibacteraceae
Sanguibacteraceae
Coriobacteriaceae
Prolixibacteraceae
Cryomorphaceae
Flavobacteriaceae
Flavobacteriaceae
Flavobacteriaceae
Flavobacteriaceae
Flavobacteriaceae
Flavobacteriaceae
Flavobacteriaceae
Sphingobacteriaceae
Bacillaceae
Paenibacillaceae
Paenibacillaceae
Planococcaceae
Clostridiaceae
ClostridialesFamilyXIII.IncertaeSedis
Eubacteriaceae
Cohaesibacteraceae
Hyphomicrobiaceae
Phyllobacteriaceae
Phyllobacteriaceae
Rhizobiaceae
Rhodobiaceae
Xanthobacteraceae
Hyphomonadaceae
Rhodobacteraceae
Acetobacteraceae
Rickettsiaceae
Alcaligenaceae
Burkholderiaceae
unclassifiedBurkholderiales
Methylophilaceae
Nitrosomonadaceae
Rhodocyclaceae
Rhodocyclaceae
Rhodocyclaceae
Nannocystaceae
Chromatiaceae
Enterobacteriaceae
Enterobacteriaceae
Enterobacteriaceae

Enterobacteriaceae
Methylococcaceae
Methylococcaceae
Moraxellaceae
Pseudomonadaceae
Pseudomonadaceae
Vibrionaceae
Alkalimonas
Dethiosulfovibrionaceae
Intrasporangiaceae
Microbacteriaceae
Nocardiopsaceae
Nocardiopsaceae
Promicromonosporaceae
Promicromonosporaceae
Streptomycetaceae
Cytophagaceae
Clostridiaceae
Rhizobiaceae
Erythrobacteraceae
Comamonadaceae
Alteromonadalesgeneraincertaesedis
Methylococcaceae
Methylococcaceae
Oxalobacteraceae
Oxalobacteraceae
Oxalobacteraceae
Oxalobacteraceae
Pseudomonadaceae
Beutenbergiaceae
Brevibacteriaceae
Microbacteriaceae
Microbacteriaceae
Microbacteriaceae
Microbacteriaceae
Microbacteriaceae
Microbacteriaceae
Microbacteriaceae
Microbacteriaceae
Microbacteriaceae
Microbacteriaceae
Microbacteriaceae
Microbacteriaceae
Micromonosporaceae
Mycobacteriaceae
Mycobacteriaceae

Mycobacteriaceae
Nocardioidaceae
Pseudonocardiaceae
Pseudonocardiaceae
Pseudonocardiaceae
Pseudonocardiaceae
Pseudonocardiaceae
Streptomycetaceae
Cytophagaceae
Flavobacteriaceae
Flavobacteriaceae
Flavobacteriaceae
Flavobacteriaceae
Sphingobacteriaceae
Sphingobacteriaceae
Sphingobacteriaceae
Sphingobacteriaceae
Sphingobacteriaceae
Sphingobacteriaceae
Sphingobacteriaceae
Sphingobacteriaceae
Sphingobacteriaceae
Sphingobacteriaceae
Sphingobacteriaceae
Bacillaceae
Bacillaceae
Bacillaceae
Bacillaceae
Planococcaceae
Leuconostocaceae
Planctomycetaceae
Methylocystaceae
Phyllobacteriaceae
Rhizobiaceae
Rhizobiaceae
Rhizobiaceae
Rhizobiaceae
Acetobacteraceae
Sphingomonadaceae
Sphingomonadaceae
Alcaligenaceae
Alcaligenaceae
Alcaligenaceae
Comamonadaceae
Comamonadaceae
Oxalobacteraceae

Oxalobacteraceae
Gallionellaceae
Nannocystaceae
Enterobacteriaceae
Enterobacteriaceae
Enterobacteriaceae
Enterobacteriaceae
Legionellaceae
Oceanospirillaceae
Moraxellaceae
Pseudomonadaceae
Xanthomonadaceae
Xanthomonadaceae
Microbacteriaceae
Aurantimonadaceae
Methylobacteriaceae
Rhizobiaceae
Sphingomonadaceae
Sphingomonadaceae
Sphingomonadaceae
Sphingomonadaceae
Burkholderiaceae
Comamonadaceae
Comamonadaceae
Comamonadaceae
Oxalobacteraceae
Enterobacteriaceae
Moraxellaceae
Microbacteriaceae
Microbacteriaceae
Micrococcaceae
Flavobacteriaceae
Planococcaceae
Planococcaceae
Hyphomicrobiaceae
Rhodobacteraceae
Oxalobacteraceae
Micrococcaceae
Promicromonosporaceae
Bacillaceae
Enterobacteriaceae
Cryptosporangiaceae
Microbacteriaceae
Pseudonocardiaceae
Prochlorococcaceae
Rhodobacteraceae

Rhodobacteraceae
Comamonadaceae
Desulfurellaceae
Cystobacteraceae
Polyangiaceae
Xanthomonadaceae
Intrasporangiaceae
Microbacteriaceae
Propionibacteriaceae
Sanguibacteraceae
Sanguibacteraceae
Sporichthyaceae
Sporichthyaceae
Flavobacteriaceae
Flavobacteriaceae
Parachlamydiaceae
Chlorobiaceae
ClostridialesFamilyXIX.IncertaeSedis
Fusobacteriaceae
Caulobacteraceae
Bartonellaceae
Beijerinckiaceae
Bradyrhizobiaceae
Phyllobacteriaceae
Phyllobacteriaceae
Rhizobiaceae
unclassifiedRhizobiales
Erythrobacteraceae
Sphingomonadaceae
Desulfovibrionaceae
Cystobacteraceae
Pseudomonadaceae
Xanthomonadaceae
Verrucomicrobiaceae
Brevibacteriaceae
Microbacteriaceae
Microbacteriaceae
Promicromonosporaceae
Sphingobacteriaceae
Sphingobacteriaceae
Sphingomonadaceae
Hydrogenophilaceae
Pseudoalteromonadaceae
Pseudoalteromonadaceae
Oceanospirillaceae
Clostridiaceae

Jiangellaceae
Jiangellaceae
Kineosporiaceae
Nocardiopsaceae
Pseudonocardiaceae
Pseudonocardiaceae
Streptomycetaceae
Porphyromonadaceae
Cytophagaceae
Alicyclobacillaceae
Bacillalesincertaesedis
Thermoactinomycetaceae
Thermoactinomycetaceae
Thermoactinomycetaceae
Thermoactinomycetaceae
Thermoactinomycetaceae
Streptococcaceae
Planctomycetaceae
Brucellaceae
Cohaesibacteraceae
Rhizobiaceae
Acetobacteraceae
Erythrobacteraceae
Erythrobacteraceae
Erythrobacteraceae
Sphingomonadaceae
Sphingomonadaceae
Alcaligenaceae
Comamonadaceae
Comamonadaceae
Comamonadaceae
Comamonadaceae
Oxalobacteraceae
Oxalobacteraceae
Hydrogenophilaceae
Rhodocyclaceae
Cystobacteraceae
Cystobacteraceae
Methylococcaceae
Bogoriellaceae
Dermacoccaceae
Microbacteriaceae
Microbacteriaceae
Micrococcaceae
Micrococcaceae
Micrococcaceae

Micrococcaceae
Micrococcaceae
Streptomycetaceae
Flavobacteriaceae
Flavobacteriaceae
Sphingobacteriaceae
Planococcaceae
Enterococcaceae
Clostridiaceae
Bradyrhizobiaceae
Brucellaceae
Rhodobacteraceae
Rhodobacteraceae
Rhodobacteraceae
Sphingomonadaceae
Comamonadaceae
Comamonadaceae
Rhodocyclaceae
Enterobacteriaceae
Cellulomonadaceae
Cellulomonadaceae
Microbacteriaceae
Ruaniaceae
Flavobacteriaceae
Anaerolineaceae
Thermosporotrichaceae
Trueperaceae
Pasteuriaceae
ClostridialesFamilyXVII.IncertaeSedis
Heliobacteriaceae
Thermoanaerobacteraceae
Phycisphaeraceae
Caulobacteraceae
Bartonellaceae
Brucellaceae
Hyphomicrobiaceae
Hyphomicrobiaceae
Phyllobacteriaceae
Phyllobacteriaceae
Rhizobiaceae
Rhizobiaceae
Rhizobiaceae
Rhizobiaceae
Rhodobiaceae
unclassifiedRhizobiales
Rhodobacteraceae

Rhodobacteraceae
Sneathiellaceae
Erythrobacteraceae
Alcaligenaceae
Alcaligenaceae
Comamonadaceae
Comamonadaceae
Methylophilaceae
Bacteriovoracaceae
Bacteriovoracaceae
Desulfohalobiaceae
Cystobacteraceae
Cystobacteraceae
Nannocystaceae
Granulosicoccaceae
Halothiobacillaceae
Legionellaceae
Alcanivoracaceae
Pseudomonadaceae
Salinisphaeraceae
Xanthomonadaceae
Acholeplasmataceae
Acholeplasmataceae
Acholeplasmataceae
Opitutaceae
Micrococcaceae
Streptomycetaceae
Flavobacteriaceae
Comamonadaceae
Bdellovibrionaceae
Bdellovibrionaceae
Moraxellaceae
Streptomycetaceae
Williamsiaceae
Flavobacteriaceae
Thermoactinomycetaceae
Aerococcaceae
Brucellaceae
Brucellaceae
Brucellaceae
Rhodobacteraceae
Alcaligenaceae
Alcaligenaceae
Alcaligenaceae
Aeromonadaceae
Aeromonadaceae

Enterobacteriaceae
Piscirickettsiaceae
Xanthomonadaceae
Xanthomonadaceae
Microbacteriaceae
Nocardioidaceae
Clostridiaceae
Peptostreptococcaceae
Methylobacteriaceae
Rhodospirillaceae
Helicobacteraceae
Enterobacteriaceae
Acidobacteriaceae
Acidobacteriaceae
Acidobacteriaceae
Nocardiaceae
Streptomycetaceae
Sphingobacteriaceae
Comamonadaceae
Nitrosomonadaceae
Flavobacteriaceae
Caulobacteraceae
Corynebacteriaceae
Kineosporiaceae
Flavobacteriaceae
Chitinophagaceae
Phormidiaceae
Carnobacteriaceae
Methylobacteriaceae
Rhodobacteraceae
Anaplasmataceae
unclassifiedBurkholderiales
Nocardioidaceae
Pseudonocardiaceae
Pseudonocardiaceae
Cytophagaceae
Sphingobacteriaceae
Paenibacillaceae
Thermoactinomycetaceae
Thermoactinomycetaceae
Rhodobacteraceae
Acetobacteraceae
Sphingomonadaceae
Rhodocyclaceae
Enterobacteriaceae
Sphingobacteriaceae

Oxalobacteraceae
Enterobacteriaceae
Corynebacteriaceae
Prevotellaceae
Methylocystaceae
Phyllobacteriaceae
Rhizobiaceae
Rhodobacteraceae
Rhodospirillaceae
Alcaligenaceae
Nitrosomonadaceae
Sinobacteraceae
Microbacteriaceae
Microbacteriaceae
Micrococcaceae
Micrococcaceae
Micromonosporaceae
Micromonosporaceae
Pseudonocardiaceae
Streptomycetaceae
Streptomycetaceae
Cyclobacteriaceae
Cyclobacteriaceae
Flavobacteriaceae
Planococcaceae
Aurantimonadaceae
Bartonellaceae
Campylobacteraceae
Enterobacteriaceae
Coxiellaceae
Pseudomonadaceae
Xanthomonadaceae
Alkalimonas
Corynebacteriaceae
Micrococcaceae
Nocardioidaceae
Pseudonocardiaceae
Flavobacteriaceae
Flavobacteriaceae
Bacillalesincertaesedis
Bacillalesincertaesedis
Paenibacillaceae
Staphylococcaceae
Bradyrhizobiaceae
Brucellaceae
Methylobacteriaceae

Rhodobacteraceae
Methylophilaceae
Sulfuricellaceae
Aeromonadaceae
Enterobacteriaceae
Coxiellaceae
Methylococcaceae
Pasteurellaceae
Actinomycetaceae
Corynebacteriaceae
Geodermatophilaceae
Geodermatophilaceae
Nocardiopsaceae
Nocardiopsaceae
Prevotellaceae
Paenibacillaceae
Acetobacteraceae
Burkholderiaceae
Aeromonadaceae
Aeromonadaceae
Micrococcaceae
Streptomycetaceae
Xanthomonadaceae

Table S1. Raw Counts of Taxanomic OTUs across all samples.

Genus
unknown_Genus_of_Acidobacteriaceae
unknown_Genus_of_Microthrixaceae
unknown_Genus_of_Acidimicrobiaceae
Aciditerrimonas
Aciditerrimonas
Ilumatobacter
unknown_Genus_of_Iamiaceae
unknown_Genus_of_Actinomycetaceae
unknown_Genus_of_Beutenbergiaceae
ambiguous_Genus_of_Beutenbergiaceae
Beutenbergia
Salana
unknown_Genus_of_Bogoriellaceae
Georgenia
Georgenia
Georgenia
Brevibacterium
Brevibacterium
Brevibacterium
ambiguous_Genus_of_Cellulomonadaceae
Cellulomonas
Cellulomonas
Cellulomonas
Cellulomonas
Corynebacterium
Corynebacterium
Corynebacterium
Corynebacterium
Corynebacterium
Corynebacterium
Corynebacterium
Corynebacterium
Corynebacterium
ambiguous_Genus_of_Demequinaceae
Demequina
Demequina
unknown_Genus_of_Dermabacteraceae
ambiguous_Genus_of_Dermabacteraceae
Brachybacterium
Brachybacterium
Brachybacterium

Brachybacterium
Brachybacterium
Brachybacterium
Brachybacterium
Brachybacterium
unknown_Genus_of_Dermacoccaceae
ambiguous_Genus_of_Dermacoccaceae
unknown_Genus_of_Dermatophilaceae
Austwickia
Dermatophilus
Kineosphaera
Mobilicoccus
Mobilicoccus
Piscicoccus
Piscicoccus
Tonsilliphilus
ambiguous_Genus_of_Dietziaceae
Dietzia
Dietzia
Dietzia
Dietzia
Dietzia
unknown_Genus_of_Frankiaceae
ambiguous_Genus_of_Geodermatophilaceae
Blastococcus
Blastococcus
Blastococcus
Blastococcus
Blastococcus
Geodermatophilus
unknown_Genus_of_Intrasporangiaceae
ambiguous_Genus_of_Intrasporangiaceae
Aquipuribacter
Janibacter
Janibacter
Janibacter
Janibacter
Knoellia
Knoellia
Kribbia
Kribbia
Ornithinibacter
Ornithinicoccus
Ornithinicoccus
Ornithinimicrobium
Ornithinimicrobium

Ornithinimicrobium
Ornithinimicrobium
Serinicoccus
Serinicoccus
Serinicoccus
Terrabacter
Terrabacter
Tetrasphaera
Tetrasphaera
Tetrasphaera
Tetrasphaera
ambiguous_Genus_of_Kineosporiaceae
ambiguous_Genus_of_Microbacteriaceae
Agrococcus
Agrococcus
Agrococcus
Agrococcus
Agrococcus
Agrococcus
Agrococcus
Agrococcus
Agrococcus
Agrococcus
Clavibacter
Curtobacterium
Frigoribacterium
Frigoribacterium
Leucobacter
Microbacterium
Microbacterium
Microbacterium
Microbacterium
Microbacterium
Microbacterium
Microbacterium
Microbacterium
Okibacterium
Plantibacter
Pseudoclavibacter
Rathayibacter
Rathayibacter
Rathayibacter
unknown_Genus_of_Micrococcaceae
ambiguous_Genus_of_Micrococcaceae
Arthrobacter
Arthrobacter

Arthrobacter
Enteractinococcus
Enteractinococcus
Kocuria
Kocuria
Kocuria
Kocuria
Kocuria
Kocuria
Microbispora
Micrococcus
Micrococcus
Micrococcus
Yaniella
Yaniella
unknown_Genus_of_Micromonosporaceae
ambiguous_Genus_of_Micromonosporaceae
Motilibacter
Motilibacter
unknown_Genus_of_Nocardiaceae
ambiguous_Genus_of_Nocardiaceae
Rhodococcus
Rhodococcus
Rhodococcus
Rhodococcus
unknown_Genus_of_Nocardioidaceae
ambiguous_Genus_of_Nocardioidaceae
Aeromicrobium
Aeromicrobium
Aeromicrobium
Marmoricola
Nocardioides
Nocardioides
Nocardioides
Nocardioides
unknown_Genus_of_Propionibacteriaceae
ambiguous_Genus_of_Propionibacteriaceae
Aestuariimicrobium
Luteococcus
Luteococcus
Propionibacterium
Tessaracoccus
Tessaracoccus
Tessaracoccus
unknown_Genus_of_Pseudonocardiaceae
Prauserella

Prauserella
Saccharopolyspora
Saccharopolyspora
Saccharopolyspora
Haloactinobacterium
ambiguous_Genus_of_Sanguibacteraceae
Sanguibacter
Sanguibacter
Sanguibacter
unknown_Genus_of_Segniliparaceae
unknown_Genus_of_Sporichthyaceae
Sporichthya
unknown_Genus_of_Streptomycetaceae
ambiguous_Genus_of_Streptomycetaceae
Streptomyces
Streptomyces
unknown_Genus_of_Streptosporangiaceae
unknown_Genus_of_Coriobacteriaceae
unknown_Genus_of_Conexibacteraceae
unknown_Genus_of_Thermoleophilaceae
unknown_Genus_of_Rhodothermaceae
unknown_Genus_of_Porphyromonadaceae
unknown_Genus_of_Cyclobacteriaceae
ambiguous_Genus_of_Cyclobacteriaceae
Algoriphagus
Echinicola
unknown_Genus_of_Cytophagaceae
ambiguous_Genus_of_Cytophagaceae
Adhaeribacter
Adhaeribacter
Adhaeribacter
Adhaeribacter
Pontibacter
Rufibacter
unknown_Genus_of_Flammeovirgaceae
Fluviicola
unknown_Genus_of_Flavobacteriaceae
ambiguous_Genus_of_Flavobacteriaceae
Aequorivita
Aequorivita
Bergeyella
Chryseobacterium
Chryseobacterium
Chryseobacterium
Chryseobacterium
Chryseobacterium

Chryseobacterium
Chryseobacterium
Empedobacter
Empedobacter
Epilithonimonas
Flavobacterium
Flavobacterium
Flavobacterium
Flavobacterium
Flavobacterium
Flavobacterium
Flavobacterium
Flavobacterium
Flavobacterium
Flavobacterium
Gillisia
Gillisia
Gillisia
Myroides
Salinimicrobium
Ulvibacter
Vitellibacter
Wautersiella
unknown_Genus_of_Chitinophagaceae
unknown_Genus_of_Sphingobacteriaceae
ambiguous_Genus_of_Sphingobacteriaceae
Mucilaginibacter
Pedobacter
Pedobacter
Pedobacter
Pedobacter
Pedobacter
Pedobacter
Pedobacter
Sphingobacterium
Sphingobacterium
Sphingobacterium
Sphingobacterium
unknown_Genus_of_Waddliaceae
unknown_Genus_of_Ardenscatenaceae
unknown_Genus_of_Thermogemmatisporaceae
unknown_Genus_of_Sphaerobacteraceae
unknown_Genus_of_Gloeobacteraceae
unknown_Genus_of_Nostocaceae
Deinococcus
unknown_Genus_of_Bacillaceae

ambiguous_Genus_of_Bacillaceae
Amphibacillus
Amphibacillus
Bacillus
Bacillus
Bacillus
Bacillus
Oceanobacillus
Ornithinibacillus
Virgibacillus
Gemella
unknown_Genus_of_Planococcaceae
ambiguous_Genus_of_Planococcaceae
Caryophanon
Kurthia
Kurthia
Kurthia
Planomicrobium
Planomicrobium
Planomicrobium
Planomicrobium
Jeotgalicoccus
Jeotgalicoccus
Jeotgalicoccus
Jeotgalicoccus
Jeotgalicoccus
Staphylococcus
Staphylococcus
Staphylococcus
Staphylococcus
Staphylococcus
Staphylococcus
Staphylococcus
Staphylococcus
Staphylococcus
unknown_Genus_of_Aerococcaceae
ambiguous_Genus_of_Aerococcaceae
Aerococcus
Facklamia
Facklamia
unknown_Genus_of_Carnobacteriaceae
Atopostipes
Trichococcus
Weissella
Streptococcus
Streptococcus

unknown_Genus_of_Clostridiaceae
ambiguous_Genus_of_Clostridiaceae
Clostridium
Clostridium
Sarcina
unknown_Genus_of_ClostridialesFamilyXII.IncertaeSedis
unknown_Genus_of_ClostridialesFamilyXIII.IncertaeSedis
Anaerostipes
unknown_Genus_of_Peptococcaceae
unknown_Genus_of_Peptostreptococcaceae
Peptostreptococcus
unknown_Genus_of_Ruminococcaceae
unknown_Genus_of_Syntrophomonadaceae
unknown_Genus_of_Natranaerobiaceae
unknown_Genus_of_Gemmatimonadaceae
unknown_Genus_of_Ignavibacteriaceae
unknown_Genus_of_Nitrospinaceae
unknown_Genus_of_Caulobacteraceae
ambiguous_Genus_of_Caulobacteraceae
Asticcacaulis
Asticcacaulis
Asticcacaulis
Asticcacaulis
Brevundimonas
Brevundimonas
Brevundimonas
Brevundimonas
Brevundimonas
Brevundimonas
Brevundimonas
Brevundimonas
Brevundimonas
Brevundimonas
Brevundimonas
Brevundimonas
Brevundimonas
Caulobacter
Caulobacter
Phenylobacterium
ambiguous_Genus_of_Aurantimonadaceae
Aurantimonas
Aurantimonas
Aureimonas
Aureimonas
unknown_Genus_of_Beijerinckiaceae
ambiguous_Genus_of_Beijerinckiaceae

Chelatococcus
Methylocapsa
unknown_Genus_of_Bradyrhizobiaceae
ambiguous_Genus_of_Bradyrhizobiaceae
Bosea
Bosea
ambiguous_Genus_of_Brucellaceae
Daeguia
Ochrobactrum
Pseudochrobactrum
unknown_Genus_of_Hyphomicrobiaceae
ambiguous_Genus_of_Hyphomicrobiaceae
Devosia
Devosia
Devosia
Devosia
Devosia
Devosia
Devosia
Devosia
Devosia
Devosia
Devosia
Devosia
Devosia
Devosia
Pelagibacterium
Pelagibacterium
Methylobacterium
Methylobacterium
Methylobacterium
Methylobacterium
Methylobacterium
ambiguous_Genus_of_Methylocystaceae
Methylocystis
Methylosinus
unknown_Genus_of_Phyllobacteriaceae
ambiguous_Genus_of_Phyllobacteriaceae
Aminobacter
Aquamicrobium
Aquamicrobium
Hoeflea
Mesorhizobium
Mesorhizobium
Mesorhizobium
Mesorhizobium

Mesorhizobium
Mesorhizobium
Nitratireductor
Nitratireductor
Phyllobacterium
Phyllobacterium
Pseudaminobacter
unknown_Genus_of_Rhizobiaceae
ambiguous_Genus_of_Rhizobiaceae
Kaistia
Rhizobium
Rhizobium
Rhizobium
Rhizobium
Rhizobium
Rhizobium
Rhizobium
Rhizobium
Shinella
unknown_Genus_of_Rhodobiaceae
ambiguous_Genus_of_Rhodobiaceae
Labrys
Vasilyevaea
unknown_Genus_of_Hyphomonadaceae
unknown_Genus_of_Rhodobacteraceae
ambiguous_Genus_of_Rhodobacteraceae
Citreicella
Gemmobacter
Gemmobacter
Gemmobacter
Jannaschia
Jannaschia
Ketogulonicigenium
Loktanella
Natronohydrobacter
Oceaniovalibus
Paracoccus
Paracoccus
Paracoccus
Paracoccus
Paracoccus
Paracoccus
Paracoccus
Paracoccus
Paracoccus
Paracoccus

Paracoccus
Paracoccus
Paracoccus
Paracoccus
Pseudorhodobacter
Pseudorhodobacter
Pseudorhodobacter
Rhodobacter
Rhodobacter
Rhodobacter
Rhodobacter
Rhodobacter
Rubellimicrobium
Rubellimicrobium
unknown_Genus_of_Acetobacteraceae
ambiguous_Genus_of_Acetobacteraceae
Roseomonas
Roseomonas
Roseomonas
Roseomonas
unknown_Genus_of_Rhodospirillaceae
unknown_Genus_of_Rickettsiaceae
unknown_Genus_of_Erythrobacteraceae
ambiguous_Genus_of_Erythrobacteraceae
Altererythrobacter
Altererythrobacter
Altererythrobacter
Altererythrobacter
Erythrobacter
Erythrobacter
Erythrobacter
Erythrobacter
Erythrobacter
Erythromicrobium
Erythromicrobium
Porphyrobacter
Porphyrobacter
unknown_Genus_of_Sphingomonadaceae
ambiguous_Genus_of_Sphingomonadaceae
Altererythrobacter
Blastomonas
Lutibacterium
Novosphingobium
Novosphingobium
Novosphingobium
Novosphingobium

Novosphingobium
Novosphingobium
Novosphingobium
Novosphingobium
Sandaracinobacter
Sphingobium
Sphingobium
Sphingobium
Sphingobium
Sphingobium
Sphingobium
Sphingomonas
Sphingomonas
Sphingomonas
Sphingomonas
Sphingomonas
Sphingomonas
Sphingomonas
Sphingomonas
Sphingomonas
Sphingomonas
Sphingomonas
Sphingomonas
Sphingomonas
Sphingomonas
Sphingomonas
Sphingomonas
Sphingomonas
Sphingopyxis
Sphingopyxis
Sphingopyxis
Sphingosinicella
unknown_Genus_of_Alcaligenaceae
Achromobacter
Achromobacter
Derxia
Oligella
Oligella
Paenalcaligenes
Pelistega
Pusillimonas
ambiguous_Genus_of_Burkholderiaceae
Burkholderia
ambiguous_Genus_of_Comamonadaceae
Acidovorax
Acidovorax

Acidovorax
Acidovorax
Acidovorax
Acidovorax
Comamonas
Comamonas
Comamonas
Comamonas
Comamonas
Diaphorobacter
Limnohabitans
Pseudorhodoferax
Pseudorhodoferax
Rhodoferax
Rhodoferax
Rubrivivax
Rubrivivax
Variovorax
Xenophilus
unknown_Genus_of_Oxalobacteraceae
ambiguous_Genus_of_Oxalobacteraceae
Janthinobacterium
Massilia
Massilia
Massilia
Massilia
Massilia
Massilia
ambiguous_Genus_of_unclassifiedBurkholderiales
Aquabacterium
Piscinibacter
unknown_Genus_of_Neisseriaceae
Neisseria
unknown_Genus_of_Rhodocyclaceae
unknown_Genus_of_Bacteriovoracaceae
Peredibacter
unknown_Genus_of_Bdellovibrionaceae
Bdellovibrio
Bdellovibrio
unknown_Genus_of_Desulfobulbaceae
unknown_Genus_of_Polyangiaceae
Campylobacter
unknown_Genus_of_Alteromonadaceae
Marinimicrobium
Marinobacter
Teredinibacter

Idiomarina
Idiomarina
Idiomarina
Shewanella
unknown_Genus_of_Chromatiaceae
ambiguous_Genus_of_Chromatiaceae
Rheinheimera
unknown_Genus_of_Enterobacteriaceae
ambiguous_Genus_of_Enterobacteriaceae
Pantoea
Pantoea
Legionella
Legionella
unknown_Genus_of_Methylococcaceae
ambiguous_Genus_of_Methylococcaceae
Methylococcus
Alcanivorax
Hahella
Zooshikella
Halomonas
unknown_Genus_of_Oceanospirillaceae
ambiguous_Genus_of_Oceanospirillaceae
unknown_Genus_of_Saccharospirillaceae
ambiguous_Genus_of_Pasteurellaceae
Avibacterium
unknown_Genus_of_Moraxellaceae
ambiguous_Genus_of_Moraxellaceae
Acinetobacter
Acinetobacter
Acinetobacter
Acinetobacter
Acinetobacter
Acinetobacter
Acinetobacter
Acinetobacter
Psychrobacter
unknown_Genus_of_Pseudomonadaceae
ambiguous_Genus_of_Pseudomonadaceae
Cellvibrio
Cellvibrio
Cellvibrio
Cellvibrio
Cellvibrio
Cellvibrio
Cellvibrio
Cellvibrio

Pseudomonas
Pseudomonas
Pseudomonas
Pseudomonas
Pseudomonas
Pseudomonas
Pseudomonas
Pseudomonas
Rhizobacter
unknown_Genus_of_Piscirickettsiaceae
Methylophaga
Thiomicrospira
ambiguous_Genus_of_Sinobacteraceae
Steroidobacter
unknown_Genus_of_Xanthomonadaceae
ambiguous_Genus_of_Xanthomonadaceae
Luteimonas
Luteimonas
Luteimonas
Luteimonas
Luteimonas
Luteimonas
Luteimonas
Lysobacter
Lysobacter
Lysobacter
Lysobacter
Lysobacter
Lysobacter
Pseudoxanthomonas
Pseudoxanthomonas
Pseudoxanthomonas
Pseudoxanthomonas
Stenotrophomonas
Stenotrophomonas
Stenotrophomonas
Stenotrophomonas
Stenotrophomonas
Stenotrophomonas
Stenotrophomonas
Stenotrophomonas
Thermomonas
Thermomonas
Thermomonas
Thermomonas
Thermomonas

Xanthomonas
unknown_Genus_of_unclassifiedGammaproteobacteria
unknown_Genus_of_Brachyspiraceae
unknown_Genus_of_Spirochaetaceae
unknown_Genus_of_Acholeplasmataceae
CandidatusPhytoplasma
CandidatusPhytoplasma
unknown_Genus_of_Mycoplasmataceae
unknown_Genus_of_Puniceicoccaceae
unknown_Genus_of_Verrucomicrobiaceae
unknown_Genus_of_Solibacteraceae
Serinibacter
Brevibacterium
Brevibacterium
Brevibacterium
Brevibacterium
Brevibacterium
Brevibacterium
unknown_Genus_of_Cellulomonadaceae
Cellulomonas
unknown_Genus_of_Corynebacteriaceae
Corynebacterium
Corynebacterium
Corynebacterium
Corynebacterium
Corynebacterium
Corynebacterium
Corynebacterium
Corynebacterium
unknown_Genus_of_Demequinaceae
Demequina
Demetria
Dietzia
Dietzia
ambiguous_Genus_of_Frankiaceae
Geodermatophilus
unknown_Genus_of_Gordoniaceae
ambiguous_Genus_of_Gordoniaceae
Gordonia
Gordonia
Gordonia
Gordonia
Gordonia
Janibacter
Knoellia
Jonesia

Jonesia
unknown_Genus_of_Kineosporiaceae
Angustibacter
unknown_Genus_of_Microbacteriaceae
Agrococcus
Agromyces
Curtobacterium
Curtobacterium
Herbiconiux
Microbacterium
Microbacterium
Microbacterium
Microbacterium
Microbacterium
Microbacterium
Plantibacter
Rathayibacter
Rathayibacter
Arthrobacter
Arthrobacter
Arthrobacter
Micrococcus
Nesterenkonia
Yaniella
Yaniella
Nakamurella
Aeromicrobium
Aeromicrobium
Marmoricola
Nocardioides
Nocardioides
Nocardioides
Nocardioides
Nocardioides
ambiguous_Genus_of_Nocardiopsaceae
Murinocardiopsis
Nocardiopsis
Nocardiopsis
Thermobifida
Thermobifida
Promicromonospora
Promicromonospora
Promicromonospora
Brooklawnia
ambiguous_Genus_of_Pseudonocardiaceae
Actinomycetospora

Pseudonocardia
Pseudonocardia
Saccharomonospora
Saccharopolyspora
Saccharopolyspora
Saccharopolyspora
Sanguibacter
Streptomyces
Streptomyces
Streptomyces
unknown_Genus_of_Bifidobacteriaceae
Patulibacter
unknown_Genus_of_Bacteroidaceae
Bacteroides
Bacteroides
Porphyromonas
Porphyromonas
Proteiniphilum
unknown_Genus_of_Prevotellaceae
Alloprevotella
Prevotella
Prevotella
Prevotella
Hymenobacter
Hymenobacter
unknown_Genus_of_Cryomorphaceae
Chryseobacterium
Chryseobacterium
Empedobacter
Flavobacterium
Flavobacterium
Myroides
Myroides
Wautersiella
Mucilaginibacter
Pedobacter
Pedobacter
Pedobacter
Pedobacter
Sphingobacterium
Sphingobacterium
Sphingobacterium
Sphingobacterium
unknown_Genus_of_Criblamydiaceae
ambiguous_Genus_of_Nostocaceae
unknown_Genus_of_Phormidiaceae

unknown_Genus_of_Deinococcaceae
Fibrobacter
unknown_Genus_of_Alicyclobacillaceae
Amphibacillus
Bacillus
Bacillus
Bacillus
Bacillus
Bacillus
Bacillus
Gracilibacillus
Lysinibacillus
Lysinibacillus
Lysinibacillus
Lysinibacillus
Oceanobacillus
Oceanobacillus
Oceanobacillus
Virgibacillus
Gemella
Gemella
Paenibacillus
Paenibacillus
Paenibacillus
Caryophanon
Caryophanon
Kurthia
Kurthia
Kurthia
Lysinibacillus
Planococcus
Planococcus
Planococcus
Planococcus
Planococcus
Planomicrobium
Planomicrobium
Solibacillus
Viridibacillus
unknown_Genus_of_Staphylococcaceae
Jeotgalicoccus
Jeotgalicoccus
Macrococcus
Macrococcus
Salinicoccus
Salinicoccus

Staphylococcus
Staphylococcus
Staphylococcus
Staphylococcus
unknown_Genus_of_Thermoactinomycetaceae
Facklamia
Globicatella
Globicatella
Globicatella
ambiguous_Genus_of_Carnobacteriaceae
Atopostipes
Carnobacterium
Granulicatella
Granulicatella
Granulicatella
unknown_Genus_of_Enterococcaceae
ambiguous_Genus_of_Enterococcaceae
Enterococcus
unknown_Genus_of_Lactobacillaceae
Lactobacillus
Weissella
Streptococcus
Streptococcus
Streptococcus
Streptococcus
Clostridium
Proteiniclasticum
unknown_Genus_of_ClostridialesFamilyXI.IncertaeSedis
Anaerococcus
Anaerococcus
Anaerococcus
Finegoldia
Peptoniphilus
Peptoniphilus
Tissierella
Tissierella
unknown_Genus_of_Lachnospiraceae
ambiguous_Genus_of_Lachnospiraceae
Blautia
Coprococcus
Coprococcus
unknown_Genus_of_Peptoniphilaceae
ambiguous_Genus_of_Peptoniphilaceae
Anaerococcus
Finegoldia
Helcococcus

Peptoclostridium
Ruminococcus
Ruminococcus
unknown_Genus_of_unclassifiedClostridiales
unknown_Genus_of_Erysipelotrichaceae
unknown_Genus_of_Veillonellaceae
unknown_Genus_of_Nitrospiraceae
unknown_Genus_of_Magnetococcaceae
unknown_Genus_of_Aurantimonadaceae
ambiguous_Genus_of_Bartonellaceae
Cucumibacter
Pelagibacterium
Chelativorans
Nitratireductor
Thermovum
Rhizobium
Rhizobium
Shinella
unknown_Genus_of_Xanthobacteraceae
Xanthobacter
ambiguous_Genus_of_unclassifiedRhizobiales
Vasilyevaea
Rhodobacter
Rhodobacter
Roseivivax
Thioclava
Tranquillimonas
Yangia
Novosphingobium
Sphingomonas
Sphingomonas
Sphingomonas
Sphingomonas
Sphingomonas
ambiguous_Genus_of_Alcaligenaceae
Advenella
Alcaligenes
Bordetella
Paenalcaligenes
Pigmentiphaga
Pigmentiphaga
Pusillimonas
unknown_Genus_of_Burkholderiaceae
Burkholderia
Comamonas
Hydrogenophaga

Hydrogenophaga
Malikia
Pelomonas
Pelomonas
Pseudacidovorax
Ramlibacter
Variovorax
Variovorax
Duganella
Duganella
Duganella
Janthinobacterium
Janthinobacterium
Janthinobacterium
Massilia
Massilia
Massilia
Tepidiphilus
Neisseria
Neisseria
Azoarcus
Denitratisoma
Petrobacter
unknown_Genus_of_Desulfobacteraceae
unknown_Genus_of_Desulfovibrionaceae
unknown_Genus_of_Geobacteraceae
unknown_Genus_of_Pelobacteraceae
unknown_Genus_of_Syntrophaceae
unknown_Genus_of_Syntrophobacteraceae
Arcobacter
Arcobacter
Arcobacter
Arcobacter
Campylobacter
unknown_Genus_of_Helicobacteraceae
unknown_Genus_of_Aeromonadaceae
Aeromonas
Alishewanella
Alishewanella
Marinimicrobium
Microbulbifer
Teredinibacter
unknown_Genus_of_Idiomarinaceae
unknown_Genus_of_Ectothiorhodospiraceae
Halothiobacillus
Citrobacter

Enterobacter
Erwinia
Erwinia
Erwinia
Erwinia
Erwinia
Leclercia
Pantoea
Pantoea
Pantoea
Serratia
Serratia
ambiguous_Genus_of_Legionellaceae
Legionella
unknown_Genus_of_Methylothermaceae
unknown_Genus_of_Alcanivoracaceae
unknown_Genus_of_Hahellaceae
unknown_Genus_of_Halomonadaceae
Halomonas
Thalassolituus
Thalassolituus
unknown_Genus_of_Orbaceae
unknown_Genus_of_Pasteurellaceae
Actinobacillus
Actinobacillus
Actinobacillus
Actinobacillus
Acinetobacter
Acinetobacter
Acinetobacter
Acinetobacter
Acinetobacter
Alkanindiges
Moraxella
Moraxella
Moraxella
Moraxella
Azomonas
Cellvibrio
Pseudomonas
Pseudomonas
Pseudomonas
Pseudomonas
Pseudomonas
Pseudomonas
Pseudomonas

Pseudomonas
Pseudomonas
Pseudomonas
Pseudomonas
Pseudomonas
Pseudomonas
Pseudomonas
Pseudomonas
Pseudomonas
Methylophaga
unknown_Genus_of_Thiotrichaceae
Achromatium
Pseudoalteromonas
ambiguous_Genus_of_Vibrionaceae
Dokdonella
Pseudoxanthomonas
Sphaerochaeta
Acholeplasma
Corynebacterium
Corynebacterium
Brachybacterium
Leucobacter
Leucobacter
Micrococcus
Nesterenkonia
Zhihengliuella
Aeromicrobium
Nocardioides
Nocardioides
Nocardioides
Lysinibacillus
Sporosarcina
Staphylococcus
Atopococcus
Enterococcus
ambiguous_Genus_of_Eubacteriaceae
Eubacterium
ambiguous_Genus_of_Peptostreptococcaceae
Citreicella
Natronohydrobacter
Paenirhodobacter
Pseudorhodobacter
Roseibaca
Tabrizicola
Sphingopyxis
Bordetella

Castellaniella
Curvibacter
unknown_Genus_of_Acidithiobacillaceae
Zobellella
ambiguous_Genus_of_Alteromonadaceae
Marinimicrobium
Aliidiomarina
ambiguous_Genus_of_Shewanellaceae
ambiguous_Genus_of_Ectothiorhodospiraceae
Erwinia
ambiguous_Genus_of_Halomonadaceae
Pseudomonas
Spongiibacter
Georgenia
ambiguous_Genus_of_Corynebacteriaceae
Brachybacterium
unknown_Genus_of_Dietziaceae
Dietzia
Stackebrandtia
Janibacter
Knoellia
Knoellia
Microbacterium
Microbacterium
Microbacterium
Arthrobacter
Arthrobacter
Citricoccus
Citricoccus
Mycobacterium
unknown_Genus_of_Nakamurellaceae
Rhodococcus
Nocardioides
Nocardiopsis
Nocardiopsis
ambiguous_Genus_of_Promicromonosporaceae
Myceligenerans
Microlunatus
Propionibacterium
Propionibacterium
Saccharopolyspora
Haloactinobacterium
Ruania
Streptomyces
Streptomyces
Cytophaga

Flavobacterium
Galbibacter
Leeuwenhoekiella
Subsaximicrobium
Winogradskyella
Parapedobacter
Alkalibacillus
Bacillus
Ornithinibacillus
Sporosarcina
Aerococcus
Desemzia
Isobaculum
Enterococcus
Enterococcus
Enterococcus
Weissella
unknown_Genus_of_Catabacteriaceae
unknown_Genus_of_Eubacteriaceae
Saccharofermentans
unknown_Genus_of_Leptotrichiaceae
Ketogulonicigenium
Paracoccus
Paracoccus
Sphingopyxis
Sphingopyxis
unknown_Genus_of_Campylobacteraceae
Acinetobacter
Pseudomonas
Methylophaga
Methylophaga
Methylophaga
Luteimonas
Simiduia
unknown_Genus_of_Dethiosulfovibrionaceae
unknown_Genus_of_Synergistaceae
Synergistes
Actinomyces
Actinomyces
Actinomyces
Actinomyces
Actinomyces
Corynebacterium
Corynebacterium
Corynebacterium
Corynebacterium

Dermacoccus
Dietzia
Dietzia
Cryobacterium
Microbacterium
Mycetocola
Kocuria
Micrococcus
Rothia
Rothia
Aeromicrobium
Nocardiopsis
Propionibacterium
Saccharopolyspora
Saccharopolyspora
unknown_Genus_of_Patulibacteraceae
unknown_Genus_of_Solirubrobacteraceae
Porphyromonas
Prevotella
Olivibacter
Bacillus
Bacillus
Exiguobacterium
Gemella
Gemella
Gemella
Brochothrix
Paenibacillus
Ureibacillus
Ureibacillus
Staphylococcus
Staphylococcus
Staphylococcus
Staphylococcus
Staphylococcus
Laceyella
Abiotrophia
Granulicatella
ambiguous_Genus_of_Lactobacillaceae
Lactobacillus
Lactobacillus
Lactococcus
Lactococcus
Streptococcus
Streptococcus
Streptococcus

Streptococcus
Streptococcus
Streptococcus
Anaerococcus
Anaerococcus
Peptoniphilus
Eubacterium
Mogibacterium
Eubacterium
Roseburia
Peptoniphilus
Filifactor
Peptostreptococcus
Faecalibacterium
Ruminococcus
Veillonella
unknown_Genus_of_Acidaminococcaceae
Negativicoccus
Selenomonas
Veillonella
Veillonella
Veillonella
Veillonella
Brevundimonas
Caulobacter
Caulobacter
Gemmiger
Methylobacterium
Paracoccus
Bordetella
Ralstonia
Oxalobacter
Tepidimonas
ambiguous_Genus_of_Hydrogenophilaceae
Neisseria
Neisseria
Campylobacter
Haemophilus
Acinetobacter
Enhydrobacter
Moraxella
Photobacterium
Thermomonas
unknown_Genus_of_Holophagaceae
Flaviflexus
Mobiluncus

Mobiluncus
Mobiluncus
ambiguous_Genus_of_Bogoriellaceae
Brevibacterium
Corynebacterium
Corynebacterium
Corynebacterium
Dermabacter
Geodermatophilus
Knoellia
Kineococcus
Kineococcus
Kineococcus
Kineococcus
Pseudokineococcus
Agrococcus
Curtobacterium
Curtobacterium
Gulosibacter
Leucobacter
Microbacterium
Arthrobacter
Kocuria
Nesterenkonia
Nocardia
Rhodococcus
Nocardioides
Nocardioides
Nocardioides
Nocardioides
Promicromonospora
Luteococcus
Tessaracoccus
Actinoalloteichus
Pseudonocardia
Streptomyces
Gaiella
Bacteroides
Prevotella
Cytophaga
Hymenobacter
Hymenobacter
Chryseobacterium
Epilithonimonas
Myroides
Myroides

Myroides
unknown_Genus_of_Saprospiraceae
Sphingobacterium
unknown_Genus_of_Dehalococcoidaceae
unknown_Genus_of_unclassifiedDehalococcoidia
unknown_Genus_of_Fibrobacteraceae
Fibrobacter
Fibrobacter
Bacillus
Bacillus
Bacillus
Gracilibacillus
Oceanobacillus
Streptohalobacillus
Paenibacillus
Paenibacillus
Caryophanon
Planococcus
Rummeliibacillus
Rummeliibacillus
Jeotgalicoccus
Staphylococcus
Thermoactinomyces
Thermoactinomyces
Aerococcus
Alkalibacterium
Alkalibacterium
Atopococcus
Enterococcus
Enterococcus
Leuconostoc
Weissella
Lactococcus
unknown_Genus_of_Christensenellaceae
Eubacterium
unknown_Genus_of_Gracilibacteraceae
Dorea
Roseburia
[Ruminococcus]
[Ruminococcus]
unknown_Genus_of_Oscillospiraceae
Peptoniphilus
unknown_Genus_of_Proteinivoraceae
ambiguous_Genus_of_Ruminococcaceae
Faecalibacterium
Ruminococcus

unknown_Genus_of_Fusobacteriaceae
Camelimonas
Ochrobactrum
Pseudochrobactrum
ambiguous_Genus_of_Methylobacteriaceae
Methylobacterium
Methylobacterium
Microvirga
Microvirga
Donghicola
Paracoccus
Sphingomicrobium
Sphingomonas
Sphingomonas
Sphingomonas
Sphingomonas
Alcaligenes
Eoetvoesia
Parapusillimonas
Pusillimonas
unknown_Genus_of_Comamonadaceae
Massilia
unknown_Genus_of_Sutterellaceae
Iodobacter
unknown_Genus_of_Nitrosomonadaceae
unknown_Genus_of_Myxococcaceae
unknown_Genus_of_Succinivibrionaceae
Aliidiomarina
Idiomarina
ambiguous_Genus_of_Pseudoalteromonadaceae
ambiguous_Genus_of_Psychromonadaceae
Shewanella
unknown_Genus_of_Arenicellaceae
Rheinheimera
Rheinheimera
Rheinheimera
Rheinheimera
Rheinheimera
unknown_Genus_of_Thioalkalispiraceae
Enterobacter
Erwinia
unknown_Genus_of_Coxiellaceae
Halomonas
Halomonas
Halomonas
Pseudomonas

ambiguous_Genus_of_Thiotrichaceae
Lysobacter
Treponema
Illumatobacter
Iamia
unknown_Genus_of_Acidothermaceae
Actinomyces
unknown_Genus_of_Actinopolysporaceae
Catenulispora
unknown_Genus_of_Geodermatophilaceae
Arsenicicoccus
Tetrasphaera
Jiangella
Agromyces
Leucobacter
Mycetocola
Longispora
unknown_Genus_of_Mycobacteriaceae
Kribbella
Nocardioides
Nocardioides
Nocardioides
Nocardioides
Nocardiopsis
Nocardiopsis
Nocardiopsis
Nocardiopsis
Nocardiopsis
Nocardiopsis
Nocardiopsis
unknown_Genus_of_Promicromonosporaceae
Isoptericola
Actinomycetospora
Prauserella
Pseudonocardia
Saccharomonospora
Saccharopolyspora
Saccharopolyspora
Saccharopolyspora
Saccharopolyspora
Saccharopolyspora
Streptomyces
Streptomyces
Streptomyces
Streptomyces
Streptomyces

unknown_Genus_of_Thermomonosporaceae
unknown_Genus_of_Gaiellaceae
unknown_Genus_of_Rubrobacteraceae
Solirubrobacter
unknown_Genus_of_Marinilabiliaceae
unknown_Genus_of_Rikenellaceae
Chryseobacterium
Flavobacterium
unknown_Genus_of_Rhabdochlamydiaceae
unknown_Genus_of_Herpetosiphonaceae
Bacillus
Bacillus
Bacillus
Bacillus
Bacillus
Gracilibacillus
Marinococcus
Ornithinibacillus
Psychrobacillus
Terribacillus
Virgibacillus
unknown_Genus_of_Paenibacillaceae
ambiguous_Genus_of_Paenibacillaceae
Planococcus
Melghirimyces
Melghirimyces
Thermoactinomyces
Thermoactinomyces
Thermoflavimicrobium
Enterococcus
unknown_Genus_of_Leuconostocaceae
Clostridium
Acidaminobacter
Eubacterium
Roseburia
Acetivibrio
Ruminococcus
Ruminococcus
Nitrospira
Bradyrhizobium
Rhodoblastus
Salinarimonas
Rhodoplanes
Thermovum
Ensifer(Sinorhizobium)
Bauldia

Rhodovulum
Skermanella
unknown_Genus_of_Holosporaceae
unknown_Genus_of_Geminicoccus
Thiobacillus
unknown_Genus_of_Chromobacteriaceae
unknown_Genus_of_Desulfonatronumaceae
unknown_Genus_of_Phaselicystidaceae
Hafnia
Rosenbergiella
Serratia
Luteimonas
Frankia
unknown_Genus_of_Glycomycetaceae
Propionibacterium
Saccharomonospora
Sanguibacter
Streptomyces
Olivibacter
Lactobacillus
Jahnella
Cronobacter
Serratia
Pseudomonas
Pseudoxanthomonas
Eggerthella
Rufibacter
Aequorivita
Gelidibacter
Gelidibacter
Salegentibacter
Salegentibacter
Salinimicrobium
Vitellibacter
unknown_Genus_of_Ardenticatenaceae
Lysinibacillus
Saccharibacillus
Aureimonas
Phyllobacterium
Paracoccus
Sphingomonas
Sphingomonas
Sphingomonas
Oxalicibacterium
Aquabacterium
Tepidiphilus

unknown_Genus_of_Sandaracinaceae
Rheinheimera
Citrobacter
Escherichia
Pantoea
Yokenella
Amphritea
Pseudomonas
Pseudomonas
Thioalkalimicrobium
unknown_Genus_of_Sinobacteraceae
Stenotrophomonas
Opitutus
Modestobacter
Kineococcus
Leifsonia
Microbacterium
Arthrobacter
Yaniella
Promicromonospora
ambiguous_Genus_of_Sporichthyaceae
Patulibacter
Dyadobacter
Lishizhenia
Aquimarina
Empedobacter
Flavobacterium
Flavobacterium
Galbibacter
Ulvibacter
CandidatusRhabdochlamydia
Paenibacillus
Salinicoccus
Shimazuella
Carnobacterium
Aureimonas
Afipia
Pseudochrobactrum
Pseudochrobactrum
Aminobacter
Sphingobium
Sphingobium
Sphingobium
Advenella
Bordetella
Pusillimonas

Comamonas
Pseudorhodoferax
Simplicispira
Variovorax
Herbaspirillum
Herbaspirillum
ambiguous_Genus_of_Bacteriovoracaceae
unknown_Genus_of_Cystobacteraceae
Saccharophagus
unknown_Genus_of_Alteromonadalesgeneraincertaesedis
Serratia
unknown_Genus_of_Legionellaceae
Halomonas
Halomonas
Halotalea
Azotobacter
Pseudomonas
Pseudomonas
Pseudomonas
Pseudomonas
unknown_Genus_of_Vibrionaceae
Luteimonas
Lysobacter
unknown_Genus_of_Bryobacter
Iamia
ambiguous_Genus_of_Catenulisporaceae
Cryptosporangium
Geodermatophilus
Modestobacter
Humibacillus
Tetrasphaera
ambiguous_Genus_of_Jiangellaceae
Angustibacter
Frondihabitans
Arthrobacter
Virgisporangium
ambiguous_Genus_of_Nakamurellaceae
Rhodococcus
Nocardioides
unknown_Genus_of_Nocardiopsaceae
Nocardiopsis
Actinoalloteichus
Actinoalloteichus
Actinomycetospora
Saccharopolyspora
Streptacidiphilus

Streptomyces
unknown_Genus_of_unclassifiedPseudonocardineae
Denitrobacterium
unknown_Genus_of_Armatimonadaceae
Cloacibacterium
Pseudosphingobacterium
unknown_Genus_of_Roseiflexaceae
unknown_Genus_of_Spirulinaceae
Crinalium
Crinalium
Bacillus
Bacillus
Lysinibacillus
Oceanobacillus
Virgibacillus
Ammoniphilus
Ammoniphilus
Paenibacillus
Paenibacillus
Sporosarcina
Leuconostoc
Clostridium
Clostridium
Salimesophilobacter
Fusibacter
Eubacterium
Butyrivibrio
Lachnobacterium
Ruminococcus
Anaerococcus
Parvularcula
Methylocella
Hyphomicrobium
Methylobacterium
Microvirga
Methylarcula
Pelagibius
Skermanella
Sphingomonas
Sphingomonas
Polynucleobacter
Delftia
Methylibium
Polaromonas
Variovorax
Herminiimonas

Thiobacillus
Methylobacillus
Methylobacillus
CandidatusDactylopiibacterium
Thauera
unknown_Genus_of_Desulfuromonadaceae
Geobacter
unknown_Genus_of_Anaeromyxobacteraceae
unknown_Genus_of_Kofleriaceae
ambiguous_Genus_of_Myxococcaceae
Microbulbifer
Microbulbifer
Trabulsiella
Yersinia
Yersinia
Steroidobacter
Ignatzschineria
unknown_Genus_of_Chthoniobacter
Nesterenkonia
Prauserella
Geofilum
Mariniflexile
Sediminibacter
Zobellia
[Ruminococcus]
Oscillibacter
unknown_Genus_of_Oligosphaeraceae
Pelistega
Hydrogenophaga
Thauera
Arcobacter
Alcanivorax
Beggiatoa
Luteibacter
Luteimonas
Arcanobacterium
Corynebacterium
Oryzihumus
Propioniferax
Rhizobium
Ketogulonicigenium
ambiguous_Genus_of_Methylophilaceae
Desulfovibrio
unknown_Genus_of_Cardiobacteriaceae
Dyella
Fulvimonas

unknown_Genus_of_Mariprofundaceae
Treponema
Beutenbergia
Leifsonia
Microbacterium
Yaniella
Leadbetterella
unknown_Genus_of_Caldilineaceae
Atopostipes
Jeotgalibaca
Trichococcus
Enterococcus
Enterococcus
Devosia
Devosia
Devosia
Loktanella
Rhodovulum
Sphingopyxis
Bordetella
Parapusillimonas
Giesbergeria
Hydrogenophaga
Simplicispira
Vitreoscilla
unknown_Genus_of_Thermithiobacillaceae
Ectothiorhodosinus
Buttiauxella
Enterobacter
Plesiomonas
Legionella
Methylohalobius
Thalassolituus
Perlucidibaca
Pseudomonas
Acholeplasma
Modestobacter
unknown_Genus_of_Jonesiaceae
Labedella
Rhodococcus
Aeromicrobium
Actinomycetospora
unknown_Genus_of_Ruaniaceae
Sanguibacter
Streptacidiphilus
Streptomyces

unknown_Genus_of_Nitriliruptoraceae
Bacteroides
Gilvibacter
Paenisporosarcina
unknown_Genus_of_Victivallaceae
Brevundimonas
Brevundimonas
Phenylobacterium
Bartonella
Rhodoplanes
Methylobacterium
Aliihoeflea
Rhizobium
ambiguous_Genus_of_Xanthobacteraceae
unknown_Genus_of_unclassifiedRhizobiales
Paracoccus
Rhodobacter
Roseococcus
Sphingomonas
Stakelama
Achromobacter
Oxalicibacterium
ambiguous_Genus_of_Chromobacteriaceae
ambiguous_Genus_of_Neisseriaceae
Marinobacter
Marinobacter
Saccharophagus
unknown_Genus_of_Pseudoalteromonadaceae
Halomonas
Haemophilus
Dasania
Arenimonas
Xanthomonas
unknown_Genus_of_OMGgroup
unknown_Genus_of_Opitutaceae
ambiguous_Genus_of_Brevibacteriaceae
Corynebacterium
Demequina
Helcobacillus
Modestobacter
Janibacter
Leifsonia
Leucobacter
Microbacterium
Microbacterium
Pseudoclavibacter

Pseudoclavibacter
Arthrobacter
Arthrobacter
Micrococcus
Rothia
Rothia
ambiguous_Genus_of_Streptosporangiaceae
unknown_Genus_of_Hydrogenothermaceae
Hymenobacter
Aequorivita
Chryseobacterium
Mesonia
Salegentibacter
Salinimicrobium
Sphingobacterium
unknown_Genus_of_Pseudanabaenaceae
Bacillus
Bacillus
Marinococcus
Kurthia
ambiguous_Genus_of_Staphylococcaceae
Macrococcus
Salinicoccus
Salinicoccus
Facklamia
Ignavigranum
Marinilactibacillus
Bavariicoccus
unknown_Genus_of_Streptococcaceae
Lactococcus
Streptococcus
Streptococcus
Streptococcus
Streptococcus
Streptococcus
Streptococcus
Streptococcus
Streptococcus
Streptococcus
Clostridium
Peptostreptococcus
Erysipelothrix
Brevundimonas
Nitratireductor
Parvibaculum
Loktanella

Lautropia
Lautropia
Methylophilus
Methylovorus
Aquaspirillum
Alysiella
Alysiella
Alysiella
Bergeriella
Kingella
Neisseria
Neisseria
Neisseria
Neisseria
Uruburuella
Denitromonas
Thauera
Bacteriovorax
Arcobacter
Suttonella
Mangrovibacter
Photorhabdus
Xenorhabdus
Legionella
Pseudospirillum
Actinobacillus
Actinobacillus
Actinobacillus
Aggregatibacter
Avibacterium
Lonepinella
Mannheimia
Pasteurella
Moraxella
Moraxella
Pseudofulvimonas
Sphaerochaeta
Sphaerochaeta
Acholeplasma
Acholeplasma
unknown_Genus_of_Spiroplasmataceae
Corynebacterium
Blastococcus
Geodermatophilus
Jonesia
Jonesia

Nocardioides
Pseudonocardia
Sanguibacter
Sanguibacter
Adlercreutzia
unknown_Genus_of_Prolixibacteraceae
Cryomorpha
Flavobacterium
Flavobacterium
Flavobacterium
Mariniflexile
Mesonia
Muricauda
Sediminibacter
Sphingobacterium
Psychrobacillus
Paenibacillus
Paenibacillus
Crocinobacterium
Clostridium
Mogibacterium
Alkalibacter
unknown_Genus_of_Cohaesibacteraceae
Devosia
Nitratireductor
Phyllobacterium
Neorhizobium
Rhodoligotrophos
Xanthobacter
Hyphomonas
Amaricoccus
Roseomonas
Rickettsia
Candidimonas
Pandoraea
Inhella
Methylophilus
Nitrosomonas
Denitratisoma
Thauera
Uliginosibacterium
Nannocystis
Rheinheimera
Enterobacter
Erwinia
Salmonella

Serratia
Methylobacter
Methylomicrobium
Perlucidibaca
Pseudomonas
Pseudomonas
Vibrio
Alkalimonas
Pyramidobacter
Tetrasphaera
Microbacterium
Murinocardiopsis
Nocardiopsis
Promicromonospora
Promicromonospora
Streptomyces
Dyadobacter
Sarcina
Rhizobium
Porphyrobacter
Hydrogenophaga
Eionea
Methylobacter
Methylosarcina
Duganella
Duganella
Massilia
Massilia
Rugamonas
Serinibacter
Brevibacterium
Curtobacterium
Herbiconiux
Labedella
Labedella
Leifsonia
Microbacterium
Microbacterium
Microbacterium
Microbacterium
Mycetocola
Plantibacter
Salinibacterium
Catelliglobosispora
Mycobacterium
Mycobacterium

Mycobacterium
Nocardioides
Amycolatopsis
Amycolatopsis
Pseudonocardia
Pseudonocardia
Pseudonocardia
Streptomyces
Dyadobacter
Elizabethkingia
Epilithonimonas
Flavobacterium
Flavobacterium
Mucilaginibacter
Mucilaginibacter
Olivibacter
Parapedobacter
Pedobacter
Pedobacter
Pedobacter
Pedobacter
Pedobacter
Pedobacter
Pedobacter
Bacillus
Bacillus
Lysinibacillus
Oceanobacillus
Ureibacillus
Weissella
unknown_Genus_of_Planctomycetaceae
Methylopila
Phyllobacterium
Agrobacterium
Neorhizobium
Neorhizobium
Rhizobium
Roseomonas
Novosphingobium
Sphingomonas
Achromobacter
Azohydromonas
Pigmentiphaga
Acidovorax
Caldimonas
Massilia

Oxalicibacterium
CandidatusNitrotoga
unknown_Genus_of_Nannocystaceae
Arsenophonus
Enterobacter
Gibbsiella
Plesiomonas
Legionella
Amphritea
Enhydrobacter
Pseudomonas
Dyella
Pseudoxanthomonas
Microbacterium
Aureimonas
Methylobacterium
Rhizobium
Sphingomonas
Sphingomonas
Sphingomonas
Sphingomonas
Lautropia
Acidovorax
Polaromonas
Polaromonas
Oxalicibacterium
Pantoea
Acinetobacter
Leucobacter
Microbacterium
Arthrobacter
Gelidibacter
Planococcus
Sporosarcina
Cucumibacter
Palleronia
Herminiimonas
Arthrobacter
Promicromonospora
Bacillus
Enterobacter
unknown_Genus_of_Cryptosporangiaceae
Agreia
Saccharomonospora
unknown_Genus_of_Prochlorococcaceae
Amaricoccus

Rubellimicrobium
Acidovorax
unknown_Genus_of_Desulfurellaceae
Cystobacter
ambiguous_Genus_of_Polyangiaceae
Xanthomonas
Janibacter
Microbacterium
Tessaracoccus
unknown_Genus_of_Sanguibacteraceae
Sanguibacter
Sporichthya
Sporichthya
Flavobacterium
Haloanella
unknown_Genus_of_Parachlamydiaceae
unknown_Genus_of_Chlorobiaceae
ambiguous_Genus_of_ClostridialesFamilyXIX.IncertaeSedis
Fusobacterium
Phenylobacterium
Bartonella
Beijerinckia
Bosea
Aminobacter
Phyllobacterium
Agrobacterium
Vasilyevaea
Erythrobacter
Novosphingobium
Bilophila
Cystobacter
Pseudomonas
Lysobacter
Luteolibacter
unknown_Genus_of_Brevibacteriaceae
Microbacterium
Microbacterium
Isoptericola
Parapedobacter
Parapedobacter
Sphingopyxis
Thiobacillus
Pseudoalteromonas
Pseudoalteromonas
Marinomonas
Clostridium

unknown_Genus_of_Jiangellaceae
Jiangella
Pseudokineococcus
Nocardiopsis
Lentzea
Pseudonocardia
Streptomyces
Petrimonas
Hymenobacter
Tumebacillus
ambiguous_Genus_of_Bacillalesincertaesedis
Planifilum
Planifilum
Planifilum
Planifilum
Planifilum
Streptococcus
Pirellula
Mycoplana
ambiguous_Genus_of_Cohaesibacteraceae
Rhizobium
Roseomonas
Altererythrobacter
Croceicoccus
Erythrobacter
Sandaracinobacter
Sphingomonas
Eoetvoesia
Brachymonas
Comamonas
Hydrogenophaga
Pelomonas
Massilia
Massilia
Thiobacillus
Azovibrio
ambiguous_Genus_of_Cystobacteraceae
Archangium
Methylobacter
Georgenia
Demetria
Frondihabitans
Salinibacterium
Arthrobacter
Citricoccus
Citricoccus

Kocuria
Kocuria
Streptomyces
Flavobacterium
Gelidibacter
Arcticibacter
Planomicrobium
Enterococcus
Clostridium
Bosea
Mycoplana
Jannaschia
Jannaschia
Roseovarius
Sphingobium
Simplicispira
Variovorax
Thauera
Escherichia
Cellulomonas
Oerskovia
Leifsonia
Ruania
Algibacter
unknown_Genus_of_Anaerolineaceae
unknown_Genus_of_Thermosporotrichaceae
unknown_Genus_of_Trueperaceae
unknown_Genus_of_Pasteuriaceae
unknown_Genus_of_ClostridialesFamilyXVII.IncertaeSedis
unknown_Genus_of_Heliobacteriaceae
unknown_Genus_of_Thermoanaerobacteraceae
unknown_Genus_of_Phycisphaeraceae
Brevundimonas
unknown_Genus_of_Bartonellaceae
unknown_Genus_of_Brucellaceae
Devosia
Hyphomicrobium
Mesorhizobium
Nitratireductor
Kaistia
Rhizobium
Shinella
Sinorhizobium
Parvibaculum
Rhizobium
Ketogulonicigenium

Paracoccus
unknown_Genus_of_Sneathiellaceae
Altererythrobacter
Castellaniella
Castellaniella
Brachymonas
Comamonas
unknown_Genus_of_Methylophilaceae
Bacteriovorax
Bacteriovorax
unknown_Genus_of_Desulfohalobiaceae
Cystobacter
Melittangium
Nannocystis
unknown_Genus_of_Granulosicoccaceae
ambiguous_Genus_of_Halothiobacillaceae
Legionella
Kangiella
Pseudomonas
unknown_Genus_of_Salinisphaeraceae
Dokdonella
Acholeplasma
Acholeplasma
Acholeplasma
Opitutus
Arthrobacter
Streptomyces
Formosa
Kinneretia
Bdellovibrio
Bdellovibrio
Psychrobacter
Streptomyces
Williamsia
Wautersiella
Shimazuella
Abiotrophia
Ochrobactrum
Paenochrobactrum
Pseudochrobactrum
Donghicola
Alcaligenes
Bordetella
Paenalcaligenes
ambiguous_Genus_of_Aeromonadaceae
Zobellella

Raoultella
Methylophaga
Ignatzschineria
Ignatzschineria
Salinibacterium
Micropruina
Clostridium
Clostridium
Microvirga
ambiguous_Genus_of_Rhodospirillaceae
Sulfurimonas
Escherichia
Edaphobacter
Edaphobacter
Edaphobacter
Nocardia
Streptomyces
Pedobacter
Hydrogenophaga
ambiguous_Genus_of_Nitrosomonadaceae
Flavobacterium
Caulobacter
Corynebacterium
Kineococcus
Flavobacterium
Flavisolibacter
Phormidium
Carnobacterium
Methylobacterium
Rubellimicrobium
unknown_Genus_of_Anaplasmataceae
Tepidimonas
Aeromicrobium
Amycolatopsis
Thermocrispum
Hymenobacter
Sphingobacterium
Brevibacillus
Polycladomyces
Polycladomyces
Agaricicola
Roseomonas
Kaistobacter
Uliginosibacterium
Kluyvera
Olivibacter

Oxalobacter
Enterobacter
Corynebacterium
Prevotella
Pleomorphomonas
Phyllobacterium
Kaistia
Pseudorhodobacter
Phaeospirillum
Pusillimonas
Nitrosomonas
Solimonas
Leucobacter
Pseudoclavibacter
Arthrobacter
Arthrobacter
Micromonospora
Micromonospora
Pseudonocardia
Streptomyces
Streptomyces
Algoriphagus
Algoriphagus
Bergeyella
Planomicrobium
Martelella
Bartonella
Campylobacter
Eschericia/Shigella
Rickettsiella
Pseudomonas
Luteimonas
unknown_Genus_of_Alkalimonas
Corynebacterium
Citricoccus
Marmoricola
Saccharomonospora
Bizionia
Flavobacterium
Bhargavaea
Bhargavaea
Brevibacillus
Salinicoccus
Bradyrhizobium
Mycoplana
Methylobacterium

Thioclava
Methylotenera
ambiguous_Genus_of_Sulfuricellaceae
Aeromonas
Enterobacter
Rickettsiella
Methylomicrobium
Nicoletella
Varibaculum
Corynebacterium
Blastococcus
Modestobacter
Marinactinospora
Spinactinospora
Prevotella
Paenibacillus
Belnapia
Ralstonia
Aeromonas
Aeromonas
Arthrobacter
Streptomyces
Luteibacter

Species	CS_242	CS_249
unknown_Genus_of_Acidobacteriaceae	196	0
unknown_Genus_of_Microthrixaceae	51	0
unknown_Genus_of_Acidimicrobiaceae	283	21
unknown_Species_of_Aciditerrimonas	139	0
Aciditerrimonas sp.	189	0
unknown_Species_of_Ilumatobacter	318	0
unknown_Genus_of_Iamiaceae	279	0
unknown_Genus_of_Actinomycetaceae	0	0
unknown_Genus_of_Beutenbergiaceae	27	49
ambiguous_Genus_of_Beutenbergiaceae	106	75
unknown_Species_of_Beutenbergia	11	17
unknown_Species_of_Salana	0	0
unknown_Genus_of_Bogoriellaceae	22	0
unknown_Species_of_Georgenia	0	0
Georgenia deserti	0	0
Georgenia sp.	59	0
ambiguous_Species_of_Brevibacterium	1223	36
Brevibacterium jeotgali	121	0
Brevibacterium linens	11	0
ambiguous_Genus_of_Cellulomonadaceae	260	0
unknown_Species_of_Cellulomonas	0	12
ambiguous_Species_of_Cellulomonas	0	0
Cellulomonas cellasea	0	0
Cellulomonas flavigena	0	0
unknown_Species_of_Corynebacterium	1402	60
ambiguous_Species_of_Corynebacterium	458	11
Corynebacterium casei	262	10
Corynebacterium deserti	132	28
Corynebacterium glutamicum	340	12
Corynebacterium halotolerans	26	12
Corynebacterium marinum	26	13
Corynebacterium maris	61	0
Corynebacterium stationis	1191	403
ambiguous_Genus_of_Demequinaceae	32	0
unknown_Species_of_Demequina	24	0
Demequina aestuarii	0	0
unknown_Genus_of_Dermabacteraceae	0	0
ambiguous_Genus_of_Dermabacteraceae	347	23
unknown_Species_of_Brachybacterium	121	0
ambiguous_Species_of_Brachybacterium	2234	275
Brachybacterium conglomeratum	36	11

Brachybacterium paraconglomeratum	43	20
Brachybacterium saurashtrense	73	12
Brachybacterium sp.	83	14
Brachybacterium squillarum	3178	68
Brachybacterium tyrofermentans	221	0
unknown_Genus_of_Dermacoccaceae	0	0
ambiguous_Genus_of_Dermacoccaceae	283	0
unknown_Genus_of_Dermatophilaceae	48	26
unknown_Species_of_Austwickia	28	0
unknown_Species_of_Dermatophilus	0	31
unknown_Species_of_Kineosphaera	0	0
unknown_Species_of_Mobilicoccus	38	11
Mobilicoccus pelagius	304	30
unknown_Species_of_Piscicoccus	0	0
Piscicoccus intestinalis	0	0
unknown_Species_of_Tonsilliphilus	11	29
ambiguous_Genus_of_Dietziaceae	12	0
ambiguous_Species_of_Dietzia	657	141
Dietzia alimentaria	171	20
Dietzia cinnamea	31	10
Dietzia maris	10	0
Dietzia sp.	0	0
unknown_Genus_of_Frankiaceae	35	0
ambiguous_Genus_of_Geodermatophilaceae	73	0
unknown_Species_of_Blastococcus	0	0
ambiguous_Species_of_Blastococcus	61	10
Blastococcus aggregatus	0	0
Blastococcus endophyticus	11	0
Blastococcus saxobsidens	0	0
Geodermatophilus terrae	42	0
unknown_Genus_of_Intrasporangiaceae	222	137
ambiguous_Genus_of_Intrasporangiaceae	3740	190
Aquipuribacter hungaricus	44	37
Janibacter limosus	20	0
Janibacter melonis	0	14
Janibacter sp.	33	0
Janibacter terrae	55	0
Knoellia aerolata	102	0
Knoellia subterranea	40	0
unknown_Species_of_Kribbia	0	0
Kribbia sp.	87	106
Ornithinibacter aureus	111	0
unknown_Species_of_Ornithinicoccus	63	0
Ornithinicoccus hortensis	23	0
unknown_Species_of_Ornithinimicrobium	70	112
ambiguous_Species_of_Ornithinimicrobium	1175	387

Ornithinimicrobium murale	59	0
Ornithinimicrobium sp.	414	0
unknown_Species_of_Serinicoccus	93	0
Serinicoccus profundi	0	0
Serinicoccus sp.	1035	247
unknown_Species_of_Terrabacter	131	190
Terrabacter sp.	0	0
unknown_Species_of_Tetrasphaera	27	0
ambiguous_Species_of_Tetrasphaera	0	0
Tetrasphaera australiensis	106	0
Tetrasphaera remsis	312	0
ambiguous_Genus_of_Kineosporiaceae	66	0
ambiguous_Genus_of_Microbacteriaceae	6411	1655
unknown_Species_of_Agrococcus	0	0
ambiguous_Species_of_Agrococcus	644	658
Agrococcus baldri	88	0
Agrococcus casei	0	0
Agrococcus citreus	21	80
Agrococcus jejuensis	97	10
Agrococcus lahaulensis	42	14
Agrococcus sp.	0	0
Agrococcus terreus	0	0
Agrococcus versicolor	36	0
Clavibacter michiganensis	115	124
ambiguous_Species_of_Curtobacterium	156	253
Frigoribacterium faeni	421	130
Frigoribacterium sp.	230	69
ambiguous_Species_of_Leucobacter	0	0
unknown_Species_of_Microbacterium	103	0
ambiguous_Species_of_Microbacterium	532	870
Microbacterium ginsengisoli	320	0
Microbacterium hatanonis	0	13
Microbacterium murale	45	0
Microbacterium profundi	112	0
Microbacterium pygmaeum	0	0
Microbacterium testaceum	13	35
Okibacterium fritillariae	0	108
unknown_Species_of_Plantibacter	125	375
Pseudoclavibacter helvolus	25	0
ambiguous_Species_of_Rathayibacter	702	386
Rathayibacter festucae	66	27
Rathayibacter iranicus	60	10
unknown_Genus_of_Micrococcaceae	80	10
ambiguous_Genus_of_Micrococcaceae	5297	256
unknown_Species_of_Arthrobacter	76	0
ambiguous_Species_of_Arthrobacter	884	51

Arthrobacter roseus	36	0
unknown_Species_of_Enteractinococcus	684	0
Enteractinococcus coprophilus	685	0
ambiguous_Species_of_Kocuria	1938	149
Kocuria carniphila	62	0
Kocuria flava	354	0
Kocuria marina	0	0
Kocuria palustris	33	0
Kocuria rosea	0	0
Microbispora rosea	0	0
ambiguous_Species_of_Micrococcus	166	0
Micrococcus lactis	43	0
Micrococcus terreus	33	0
Yaniella fodinae	88	0
Yaniella sp.	365	0
unknown_Genus_of_Micromonosporaceae	34	0
ambiguous_Genus_of_Micromonosporaceae	77	0
Motilibacter peucedani	0	0
Motilibacter rhizosphaerae	0	0
unknown_Genus_of_Nocardiaceae	0	0
ambiguous_Genus_of_Nocardiaceae	42	333
unknown_Species_of_Rhodococcus	0	0
ambiguous_Species_of_Rhodococcus	456	154
Rhodococcus corynebacterioides	94	0
Rhodococcus fascians	86	71
unknown_Genus_of_Nocardioidaceae	461	32
ambiguous_Genus_of_Nocardioidaceae	490	0
ambiguous_Species_of_Aeromicrobium	336	0
Aeromicrobium erythreum	96	0
Aeromicrobium ponti	53	0
ambiguous_Species_of_Marmoricola	84	0
unknown_Species_of_Nocardioides	167	0
ambiguous_Species_of_Nocardioides	669	10
Nocardioides alkalitolerans	54	0
Nocardioides daphniae	129	0
unknown_Genus_of_Propionibacteriaceae	139	35
ambiguous_Genus_of_Propionibacteriaceae	86	62
unknown_Species_of_Aestuariimicrobium	0	0
unknown_Species_of_Luteococcus	0	0
Luteococcus peritonei	0	0
Propionibacterium acnes	1143	0
Tessaracoccus lubricantis	0	0
Tessaracoccus oleiagri	10	17
Tessaracoccus sp.	0	0
unknown_Genus_of_Pseudonocardiaceae	304	44
ambiguous_Species_of_Prauserella	226	52

Prauserella rugosa	198	82
unknown_Species_of_Saccharopolyspora	1729	149
ambiguous_Species_of_Saccharopolyspora	6686	693
Saccharopolyspora tripterygii	10715	0
Haloactinobacterium album	43	0
ambiguous_Genus_of_Sanguibacteraceae	323	282
ambiguous_Species_of_Sanguibacter	463	576
Sanguibacter inulinus	0	62
Sanguibacter sp.	0	0
unknown_Genus_of_Segniliparaceae	10	0
unknown_Genus_of_Sporichthyaceae	41	0
Sporichthya sp.	0	0
unknown_Genus_of_Streptomycetaceae	10	0
ambiguous_Genus_of_Streptomycetaceae	1233	0
unknown_Species_of_Streptomyces	95	0
Streptomyces avicenniae	218	64
unknown_Genus_of_Streptosporangiaceae	76	0
unknown_Genus_of_Coriobacteriaceae	85	0
unknown_Genus_of_Conexibacteraceae	0	0
unknown_Genus_of_Thermoleophilaceae	12	0
unknown_Genus_of_Rhodothermaceae	22	0
unknown_Genus_of_Porphyromonadaceae	448	10
unknown_Genus_of_Cyclobacteriaceae	0	60
ambiguous_Genus_of_Cyclobacteriaceae	0	0
unknown_Species_of_Algoriphagus	0	32
unknown_Species_of_Echinicola	0	0
unknown_Genus_of_Cytophagaceae	53	78
ambiguous_Genus_of_Cytophagaceae	0	0
unknown_Species_of_Adhaeribacter	65	11
Adhaeribacter aerophilus	0	0
Adhaeribacter aquaticus	13	0
Adhaeribacter terreus	0	74
unknown_Species_of_Pontibacter	0	13
Rufibacter tibetensis	0	0
unknown_Genus_of_Flammeovirgaceae	0	0
unknown_Species_of_Fluviicola	254	0
unknown_Genus_of_Flavobacteriaceae	5469	855
ambiguous_Genus_of_Flavobacteriaceae	2404	1634
unknown_Species_of_Aequorivita	15	10
Aequorivita antarctica	0	0
Bergeyella sp.	0	0
unknown_Species_of_Chryseobacterium	650	1072
ambiguous_Species_of_Chryseobacterium	51	290
Chryseobacterium antarcticum	0	46
Chryseobacterium anthropi	0	29
Chryseobacterium haifense	439	187

Chryseobacterium koreense	220	85
Chryseobacterium sp.	254	687
unknown_Species_of_Empedobacter	0	0
Empedobacter sp.	0	0
ambiguous_Species_of_Epilithonimonas	0	122
unknown_Species_of_Flavobacterium	1822	599
ambiguous_Species_of_Flavobacterium	96	154
Flavobacterium anatoliense	550	0
Flavobacterium araucananum	111	0
Flavobacterium cheniae	0	0
Flavobacterium cheonanense	24	37
Flavobacterium hercynium	86	0
Flavobacterium pectinovorum	15	0
Flavobacterium sp.	89	704
Flavobacterium succinicans	272	0
unknown_Species_of_Gillisia	0	0
Gillisia limnaea	0	11
Gillisia sp.	67	64
unknown_Species_of_Myroides	249	16
unknown_Species_of_Salinimicrobium	17	0
unknown_Species_of_Ulvibacter	0	0
unknown_Species_of_Vitellibacter	16	11
Wautersiella sp.	99	273
unknown_Genus_of_Chitinophagaceae	13	0
unknown_Genus_of_Sphingobacteriaceae	1806	671
ambiguous_Genus_of_Sphingobacteriaceae	27	155
Mucilaginibacter sp.	10	0
unknown_Species_of_Pedobacter	402	928
ambiguous_Species_of_Pedobacter	63	631
Pedobacter agri	0	333
Pedobacter bauzanensis	11	170
Pedobacter metabolipauper	39	68
Pedobacter oryzae	0	0
Pedobacter sp.	51	64
unknown_Species_of_Sphingobacterium	433	116
Sphingobacterium alimentarium	54	183
Sphingobacterium sp.	58	118
Sphingobacterium wenxiniae	0	0
unknown_Genus_of_Waddliaceae	0	27
unknown_Genus_of_Ardenscatenaceae	204	0
unknown_Genus_of_Thermogemmatisporaceae	20	131
unknown_Genus_of_Sphaerobacteraceae	152	46
unknown_Genus_of_Gloeobacteraceae	178	137
unknown_Genus_of_Nostocaceae	1312	2321
Deinococcus geothermalis	0	0
unknown_Genus_of_Bacillaceae	4368	315

ambiguous_Genus_of_Bacillaceae	10586	424
Amphibacillus indicireducens	366	0
Amphibacillus xylanus	10	0
unknown_Species_of_Bacillus	1447	0
ambiguous_Species_of_Bacillus	16880	68
Bacillus sp.	276	0
Bacillus weihenstephanensis	80	41
unknown_Species_of_Oceanobacillus	1045	0
unknown_Species_of_Ornithinibacillus	235	0
Virgibacillus soli	52	0
Gemella palaticanis	0	0
unknown_Genus_of_Planococcaceae	1909	17
ambiguous_Genus_of_Planococcaceae	5365	927
Caryophanon latum	274	118
ambiguous_Species_of_Kurthia	170	0
Kurthia massiliensis	451	0
Kurthia zopfii	1985	42
ambiguous_Species_of_Planomicrobium	95	0
Planomicrobium flavidum	105	0
Planomicrobium okeanokoites	274	0
Planomicrobium sp.	113	0
ambiguous_Species_of_Jeotgalicoccus	131	0
Jeotgalicoccus coquinae	497	0
Jeotgalicoccus halotolerans	111	0
Jeotgalicoccus psychrophilus	43	0
Jeotgalicoccus sp.	52	48
ambiguous_Species_of_Staphylococcus	4715	81
Staphylococcus equorum	321	0
Staphylococcus fleurettii	145	0
Staphylococcus saprophyticus	194	0
Staphylococcus sciuri	1380	224
Staphylococcus succinus	996	11
Staphylococcus vitulinus	202	0
Staphylococcus warneri	77	0
Staphylococcus xylosus	291	55
unknown_Genus_of_Aerococcaceae	286	0
ambiguous_Genus_of_Aerococcaceae	383	0
Aerococcus urinaeequi	1028	14
unknown_Species_of_Facklamia	348	24
Facklamia tabacinasalis	363	96
unknown_Genus_of_Carnobacteriaceae	2823	0
unknown_Species_of_Atopostipes	6517	0
ambiguous_Species_of_Trichococcus	80	0
Weissella confusa	830	28
ambiguous_Species_of_Streptococcus	413	44
Streptococcus equinus	49	11

unknown_Genus_of_Clostridiaceae	1193	471
ambiguous_Genus_of_Clostridiaceae	1526	36
unknown_Species_of_Clostridium	155	29
ambiguous_Species_of_Clostridium	1355	0
ambiguous_Species_of_Sarcina	0	0
unknown_Genus_of_ClostridialesFamilyXII.IncertaeSedis	0	36
unknown_Genus_of_ClostridialesFamilyXIII.IncertaeSedis	260	0
unknown_Species_of_Anaerostipes	169	0
unknown_Genus_of_Peptococcaceae	10	0
unknown_Genus_of_Peptostreptococcaceae	80	0
unknown_Species_of_Peptostreptococcus	0	0
unknown_Genus_of_Ruminococcaceae	1236	50
unknown_Genus_of_Syntrophomonadaceae	0	0
unknown_Genus_of_Natranaerobiaceae	51	0
unknown_Genus_of_Gemmatimonadaceae	53	0
unknown_Genus_of_Ignavibacteriaceae	0	0
unknown_Genus_of_Nitrospinaceae	0	0
unknown_Genus_of_Caulobacteraceae	37	0
ambiguous_Genus_of_Caulobacteraceae	1228	596
unknown_Species_of_Asticcacaulis	0	0
Asticcacaulis biprosthecium	0	0
Asticcacaulis excentricus	0	0
Asticcacaulis sp.	11	0
ambiguous_Species_of_Brevundimonas	605	558
Brevundimonas alba	0	0
Brevundimonas bullata	0	61
Brevundimonas diminuta	193	0
Brevundimonas faecalis	242	10
Brevundimonas halotolerans	0	23
Brevundimonas lenta	52	0
Brevundimonas poindexterae	0	0
Brevundimonas sp.	0	0
Brevundimonas staleyi	12	0
Brevundimonas subvibrioides	42	30
Brevundimonas terrae	242	72
Brevundimonas variabilis	0	0
unknown_Species_of_Caulobacter	0	0
Caulobacter daechungensis	0	0
ambiguous_Species_of_Phenylobacterium	12	0
ambiguous_Genus_of_Aurantimonadaceae	48	0
unknown_Species_of_Aurantimonas	0	78
Aurantimonas sp.	63	117
Aureimonas ferruginea	57	58
Aureimonas jatrophae	123	73
unknown_Genus_of_Beijerinckiaceae	15	0
ambiguous_Genus_of_Beijerinckiaceae	0	0

unknown_Species_of_Chelatococcus	0	28
unknown_Species_of_Methylocapsa	0	0
unknown_Genus_of_Bradyrhizobiaceae	0	0
ambiguous_Genus_of_Bradyrhizobiaceae	572	767
ambiguous_Species_of_Bosea	27	12
Bosea lathyri	25	14
ambiguous_Genus_of_Brucellaceae	0	10
unknown_Species_of_Daeguia	0	20
unknown_Species_of_Ochrobactrum	21	0
ambiguous_Species_of_Pseudochrobactrum	10	37
unknown_Genus_of_Hyphomicrobiaceae	132	30
ambiguous_Genus_of_Hyphomicrobiaceae	302	80
unknown_Species_of_Devosia	10	257
ambiguous_Species_of_Devosia	242	250
Devosia chinhatensis	0	20
Devosia crocina	0	0
Devosia geojensis	0	0
Devosia glacialis	0	55
Devosia limi	0	0
Devosia neptuniae	0	0
Devosia psychrophila	41	72
Devosia riboflavina	0	0
Devosia soli	0	37
Devosia sp.	67	411
Devosia subaequoris	0	0
Devosia submarina	0	0
Pelagibacterium halotolerans	12	11
Pelagibacterium luteolum	0	0
ambiguous_Species_of_Methylobacterium	811	1288
Methylobacterium adhaesivum	0	183
Methylobacterium bullatum	0	52
Methylobacterium goesingense	41	291
Methylobacterium marchantiae	25	115
ambiguous_Genus_of_Methylocystaceae	13	0
unknown_Species_of_Methylocystis	0	0
unknown_Species_of_Methylosinus	0	0
unknown_Genus_of_Phyllobacteriaceae	0	0
ambiguous_Genus_of_Phyllobacteriaceae	150	104
ambiguous_Species_of_Aminobacter	0	0
ambiguous_Species_of_Aquamicrobium	0	11
Aquamicrobium aerolatum	0	0
Hoeflea alexandrii	0	0
unknown_Species_of_Mesorhizobium	0	0
ambiguous_Species_of_Mesorhizobium	0	0
Mesorhizobium camelthorni	0	0
Mesorhizobium chacoense	0	0

Mesorhizobium plurifarium	67	34
Mesorhizobium sp.	0	0
ambiguous_Species_of_Nitratireductor	0	0
Nitratireductor pacificus	0	0
Phyllobacterium leguminum	0	24
Phyllobacterium sp.	0	42
Pseudaminobacter salicylatoxidans	0	0
unknown_Genus_of_Rhizobiaceae	0	0
ambiguous_Genus_of_Rhizobiaceae	1128	1163
ambiguous_Species_of_Kaistia	11	46
unknown_Species_of_Rhizobium	29	0
ambiguous_Species_of_Rhizobium	473	1619
Rhizobium giardinii	0	0
Rhizobium selenitireducens	0	0
Rhizobium skierniewicense	35	155
Rhizobium soli	202	1697
Rhizobium sp.	0	211
Rhizobium sphaerophysae	22	0
Shinella sp.	0	0
unknown_Genus_of_Rhodobiaceae	53	0
ambiguous_Genus_of_Rhodobiaceae	0	0
Labrys wisconsinensis	0	0
Vasilyevaea enhydra	10	0
unknown_Genus_of_Hyphomonadaceae	207	92
unknown_Genus_of_Rhodobacteraceae	810	830
ambiguous_Genus_of_Rhodobacteraceae	1373	671
unknown_Species_of_Citreicella	219	25
unknown_Species_of_Gemmobacter	71	28
Gemmobacter sp.	206	104
Gemmobacter tilapiae	0	0
unknown_Species_of_Jannaschia	0	0
Jannaschia rubra	0	0
Ketogulonicigenium robustum	96	43
ambiguous_Species_of_Loktanella	148	0
unknown_Species_of_Natronohydrobacter	208	0
unknown_Species_of_Oceaniovalibus	0	0
unknown_Species_of_Paracoccus	99	365
ambiguous_Species_of_Paracoccus	3553	3814
Paracoccus alcaliphilus	50	63
Paracoccus alkenifer	0	0
Paracoccus aminovorans	327	1103
Paracoccus halophilus	0	0
Paracoccus kocurii	22	12
Paracoccus marcusii	0	116
Paracoccus solventivorans	0	0
Paracoccus sp.	95	184

Paracoccus sphaerophysae	0	95
Paracoccus stylophorae	0	41
Paracoccus thiocyanatus	0	0
Paracoccus tibetensis	106	983
ambiguous_Species_of_Pseudorhodobacter	81	0
Pseudorhodobacter sp.	62	13
Pseudorhodobacter wandonensis	111	73
unknown_Species_of_Rhodobacter	240	0
ambiguous_Species_of_Rhodobacter	0	0
Rhodobacter aestuarii	61	0
Rhodobacter sp.	855	73
Rhodobacter veldkampii	12	59
unknown_Species_of_Rubellimicrobium	0	0
ambiguous_Species_of_Rubellimicrobium	79	10
unknown_Genus_of_Acetobacteraceae	71	0
ambiguous_Genus_of_Acetobacteraceae	0	0
unknown_Species_of_Roseomonas	0	0
ambiguous_Species_of_Roseomonas	10	0
Roseomonas aerophila	47	246
Roseomonas aquatica	0	0
unknown_Genus_of_Rhodospirillaceae	63	95
unknown_Genus_of_Rickettsiaceae	0	0
unknown_Genus_of_Erythrobacteraceae	64	130
ambiguous_Genus_of_Erythrobacteraceae	57	322
unknown_Species_of_Altererythrobacter	80	273
Altererythrobacter epoxidivorans	0	47
Altererythrobacter namhicola	10	0
Altererythrobacter sp.	57	53
unknown_Species_of_Erythrobacter	38	68
ambiguous_Species_of_Erythrobacter	0	23
Erythrobacter gangjinensis	0	77
Erythrobacter pelagi	0	10
Erythrobacter sp.	0	28
unknown_Species_of_Erythromicrobium	0	20
Erythromicrobium ramosum	0	0
unknown_Species_of_Porphyrobacter	0	28
Porphyrobacter sanguineus	140	62
unknown_Genus_of_Sphingomonadaceae	236	46
ambiguous_Genus_of_Sphingomonadaceae	11519	6769
Altererythrobacter troitsensis	35	176
Blastomonas sp.	0	0
Lutibacterium sp.	0	21
unknown_Species_of_Novosphingobium	437	67
ambiguous_Species_of_Novosphingobium	51	24
Novosphingobium lentum	0	12
Novosphingobium nitrogenifigens	0	0

Novosphingobium rosa	0	0
Novosphingobium soli	384	218
Novosphingobium sp.	0	13
Novosphingobium tardaugens	0	0
Sandaracinobacter sibiricus	66	0
ambiguous_Species_of_Sphingobium	64	0
Sphingobium faniae	0	0
Sphingobium fontiphilum	0	0
Sphingobium jiangsuense	0	0
Sphingobium sp.	0	0
Sphingobium vulgare	0	0
unknown_Species_of_Sphingomonas	37	108
ambiguous_Species_of_Sphingomonas	3896	17580
Sphingomonas aerolata	318	960
Sphingomonas aestuarii	13	297
Sphingomonas alpina	0	0
Sphingomonas asaccharolytica	0	14
Sphingomonas astaxanthinifaciens	33	22
Sphingomonas cynarae	45	518
Sphingomonas faeni	1084	1741
Sphingomonas glacialis	265	724
Sphingomonas hankookensis	136	169
Sphingomonas koreensis	305	62
Sphingomonas kwangyangensis	0	0
Sphingomonas molluscorum	0	14
Sphingomonas mucosissima	0	0
Sphingomonas pseudosanguinis	10	53
Sphingomonas sp.	462	1104
unknown_Species_of_Sphingopyxis	0	0
ambiguous_Species_of_Sphingopyxis	0	0
Sphingopyxis bauzanensis	0	12
unknown_Species_of_Sphingosinicella	0	0
unknown_Genus_of_Alcaligenaceae	1846	148
unknown_Species_of_Achromobacter	40	22
ambiguous_Species_of_Achromobacter	20	23
unknown_Species_of_Derxia	0	0
unknown_Species_of_Oligella	375	64
Oligella ureolytica	39	83
unknown_Species_of_Paenalcaligenes	304	137
unknown_Species_of_Pelistega	276	207
Pusillimonas noertemannii	0	10
ambiguous_Genus_of_Burkholderiaceae	133	0
unknown_Species_of_Burkholderia	0	13
ambiguous_Genus_of_Comamonadaceae	1140	847
unknown_Species_of_Acidovorax	24	23
ambiguous_Species_of_Acidovorax	0	0

Acidovorax defluvii	11	0
Acidovorax facilis	0	34
Acidovorax soli	0	0
Acidovorax sp.	95	261
unknown_Species_of_Comamonas	997	88
ambiguous_Species_of_Comamonas	413	232
Comamonas aquatica	348	105
Comamonas jiangduensis	84	0
Comamonas kerstersii	723	0
Diaphorobacter oryzae	69	0
Limnohabitans parvus	0	44
ambiguous_Species_of_Pseudorhodoferax	11	39
Pseudorhodoferax soli	0	0
unknown_Species_of_Rhodoferax	0	0
Rhodoferax ferrireducens	0	0
unknown_Species_of_Rubrivivax	0	0
Rubrivivax gelatinosus	0	0
ambiguous_Species_of_Variovorax	20	63
Xenophilus aerolatus	0	30
unknown_Genus_of_Oxalobacteraceae	26	10
ambiguous_Genus_of_Oxalobacteraceae	119	868
Janthinobacterium sp.	0	11
ambiguous_Species_of_Massilia	555	4210
Massilia aerilata	0	20
Massilia aurea	1300	1214
Massilia brevitalea	0	0
Massilia namucuonensis	0	1188
Massilia sp.	567	375
ambiguous_Genus_of_unclassifiedBurkholderiales	10	0
Aquabacterium commune	0	0
Piscinibacter aquaticus	0	0
unknown_Genus_of_Neisseriaceae	165	0
Neisseria flavescens	52	0
unknown_Genus_of_Rhodocyclaceae	0	25
unknown_Genus_of_Bacteriovoracaceae	59	0
Peredibacter starrii	0	0
unknown_Genus_of_Bdellovibrionaceae	0	16
unknown_Species_of_Bdellovibrio	0	0
Bdellovibrio bacteriovorus	0	29
unknown_Genus_of_Desulfobulbaceae	0	12
unknown_Genus_of_Polyangiaceae	10	0
unknown_Species_of_Campylobacter	59	0
unknown_Genus_of_Alteromonadaceae	136	61
unknown_Species_of_Marinimicrobium	40	104
ambiguous_Species_of_Marinobacter	16	64
Teredinibacter turnerae	0	0

unknown_Species_of_Idiomarina	0	0
ambiguous_Species_of_Idiomarina	169	245
Idiomarina indica	0	0
unknown_Species_of_Shewanella	0	24
unknown_Genus_of_Chromatiaceae	22	0
ambiguous_Genus_of_Chromatiaceae	0	0
ambiguous_Species_of_Rheinheimera	0	0
unknown_Genus_of_Enterobacteriaceae	0	0
ambiguous_Genus_of_Enterobacteriaceae	3788	6600
ambiguous_Species_of_Pantoea	3034	3455
Pantoea agglomerans	1271	1164
ambiguous_Species_of_Legionella	49	0
Legionella fallonii	0	0
unknown_Genus_of_Methylococcaceae	0	0
ambiguous_Genus_of_Methylococcaceae	0	0
unknown_Species_of_Methylococcus	0	0
unknown_Species_of_Alcanivorax	0	0
unknown_Species_of_Hahella	0	0
Zooshikella ganghwensis	0	0
Halomonas sp.	10	11
unknown_Genus_of_Oceanospirillaceae	56	33
ambiguous_Genus_of_Oceanospirillaceae	59	30
unknown_Genus_of_Saccharospirillaceae	0	0
ambiguous_Genus_of_Pasteurellaceae	0	0
Avibacterium sp.	0	0
unknown_Genus_of_Moraxellaceae	271	151
ambiguous_Genus_of_Moraxellaceae	2613	11895
unknown_Species_of_Acinetobacter	2101	441
ambiguous_Species_of_Acinetobacter	17139	11869
Acinetobacter calcoaceticus	174	82
Acinetobacter johnsonii	151	89
Acinetobacter junii	190	33
Acinetobacter lwoffii	4002	2161
Acinetobacter sp.	1683	1148
Acinetobacter ursingii	71	94
ambiguous_Species_of_Psychrobacter	36	0
unknown_Genus_of_Pseudomonadaceae	906	96
ambiguous_Genus_of_Pseudomonadaceae	413	367
unknown_Species_of_Cellvibrio	59	10
ambiguous_Species_of_Cellvibrio	311	137
Cellvibrio fibrivorans	54	0
Cellvibrio fulvus	0	0
Cellvibrio gandavensis	509	261
Cellvibrio mixtus	0	0
Cellvibrio ostraviensis	22	0
Cellvibrio sp.	415	231

unknown_Species_of_Pseudomonas	625	65
ambiguous_Species_of_Pseudomonas	4818	9980
Pseudomonas argentinensis	19	0
Pseudomonas formosensis	200	412
Pseudomonas pertucinogena	43	0
Pseudomonas rhizosphaerae	0	0
Pseudomonas sp.	590	967
Pseudomonas vranovensis	84	114
Rhizobacter fulvus	26	13
unknown_Genus_of_Piscirickettsiaceae	15	0
unknown_Species_of_Methylophaga	71	11
unknown_Species_of_Thiomicrospira	0	38
ambiguous_Genus_of_Sinobacteraceae	0	0
unknown_Species_of_Steroidobacter	13	0
unknown_Genus_of_Xanthomonadaceae	281	14
ambiguous_Genus_of_Xanthomonadaceae	435	4361
unknown_Species_of_Luteimonas	202	86
ambiguous_Species_of_Luteimonas	24	476
Luteimonas composti	0	60
Luteimonas mephitis	0	0
Luteimonas sp.	37	56
Luteimonas terricola	0	15
Luteimonas vadosa	0	0
unknown_Species_of_Lysobacter	116	61
ambiguous_Species_of_Lysobacter	68	64
Lysobacter antibioticus	0	0
Lysobacter arseniciresistens	0	0
Lysobacter bugurensis	0	0
Lysobacter sp.	0	15
unknown_Species_of_Pseudoxanthomonas	46	0
ambiguous_Species_of_Pseudoxanthomonas	0	0
Pseudoxanthomonas sp.	0	0
Pseudoxanthomonas suwonensis	26	0
unknown_Species_of_Stenotrophomonas	363	93
ambiguous_Species_of_Stenotrophomonas	10	629
Stenotrophomonas chelatiphaga	0	657
Stenotrophomonas ginsengisoli	274	0
Stenotrophomonas humi	177	0
Stenotrophomonas koreensis	441	185
Stenotrophomonas rhizophila	84	2999
Stenotrophomonas sp.	148	33
unknown_Species_of_Thermomonas	96	684
ambiguous_Species_of_Thermomonas	0	0
Thermomonas brevis	93	1118
Thermomonas fusca	11	0
Thermomonas koreensis	39	1377

Xanthomonas sp.	0	0
unknown_Genus_of_unclassifiedGammaproteobacteria	20	0
unknown_Genus_of_Brachyspiraceae	58	135
unknown_Genus_of_Spirochaetaceae	21	0
unknown_Genus_of_Acholeplasmataceae	18	11
unknown_Species_of_CandidatusPhytoplasma	0	0
CandidatusPhytoplasma sp.	0	0
unknown_Genus_of_Mycoplasmataceae	26	0
unknown_Genus_of_Puniceicoccaceae	0	0
unknown_Genus_of_Verrucomicrobiaceae	0	110
unknown_Genus_of_Solibacteraceae	194	0
Serinibacter salmoneus	98	0
unknown_Species_of_Brevibacterium	149	0
Brevibacterium antiquum	46	0
Brevibacterium aurantiacum	92	0
Brevibacterium senegalense	72	0
Brevibacterium sp.	0	0
Brevibacterium yomogidense	39	0
unknown_Genus_of_Cellulomonadaceae	33	0
Cellulomonas sp.	0	0
unknown_Genus_of_Corynebacteriaceae	44	0
Corynebacterium ammoniagenes	35	0
Corynebacterium efficiens	0	0
Corynebacterium glucuronolyticum	10	0
Corynebacterium humireducens	38	0
Corynebacterium sp.	0	0
Corynebacterium tuscaniense	0	0
Corynebacterium urealyticum	17	0
Corynebacterium variabile	79	0
unknown_Genus_of_Demequinaceae	0	0
Demequina aurantiaca	0	0
Demetria terragena	0	0
unknown_Species_of_Dietzia	0	0
Dietzia aerolata	0	0
ambiguous_Genus_of_Frankiaceae	43	0
unknown_Species_of_Geodermatophilus	0	0
unknown_Genus_of_Gordoniaceae	0	0
ambiguous_Genus_of_Gordoniaceae	0	0
unknown_Species_of_Gordonia	0	0
ambiguous_Species_of_Gordonia	0	0
Gordonia humi	0	0
Gordonia paraffinivorans	0	0
Gordonia shandongensis	0	0
ambiguous_Species_of_Janibacter	0	0
Knoellia locipacati	104	0
unknown_Species_of_Jonesia	15	0

Jonesia denitrificans	0	0
unknown_Genus_of_Kineosporiaceae	25	0
ambiguous_Species_of_Angustibacter	0	0
unknown_Genus_of_Microbacteriaceae	49	0
Agrococcus carbonis	10	0
Agromyces cerinus	0	0
Curtobacterium herbarum	12	0
Curtobacterium luteum	0	0
Herbiconiux moechotypicola	0	0
Microbacterium amylolyticum	63	0
Microbacterium arabinogalactanolyticum	0	0
Microbacterium flavum	0	32
Microbacterium gubbeenense	27	0
Microbacterium indicum	0	0
Microbacterium saperdae	10	0
Plantibacter flavus	0	0
Rathayibacter caricis	11	0
Rathayibacter tritici	48	0
Arthrobacter arilaitensis	207	0
Arthrobacter mysorens	0	11
Arthrobacter protophormiae	24	0
unknown_Species_of_Micrococcus	0	0
Nesterenkonia aethiopica	0	0
unknown_Species_of_Yaniella	207	0
Yaniella soli	65	0
unknown_Species_of_Nakamurella	33	0
unknown_Species_of_Aeromicrobium	0	0
Aeromicrobium sp.	0	0
Marmoricola korecus	0	0
Nocardioides iriomotensis	0	0
Nocardioides jensenii	0	0
Nocardioides lianchengensis	0	0
Nocardioides plantarum	34	0
Nocardioides sp.	325	0
ambiguous_Genus_of_Nocardiopsaceae	0	0
unknown_Species_of_Murinocardiopsis	0	0
Nocardiopsis composta	0	0
Nocardiopsis sp.	141	0
ambiguous_Species_of_Thermobifida	0	0
Thermobifida alba	0	0
ambiguous_Species_of_Promicromonospora	65	0
Promicromonospora endophytica	0	0
Promicromonospora thailandica	0	0
unknown_Species_of_Brooklawnia	0	0
ambiguous_Genus_of_Pseudonocardiaceae	371	0
unknown_Species_of_Actinomycetospora	11	0

unknown_Species_of_Pseudonocardia	38	0
Pseudonocardia ammonioxydans	0	0
Saccharomonospora viridis	0	0
Saccharopolyspora dendranthemae	0	0
Saccharopolyspora flava	0	10
Saccharopolyspora gloriosa	483	63
Sanguibacter soli	0	0
ambiguous_Species_of_Streptomyces	209	0
Streptomyces alanosinicus	0	0
Streptomyces sp.	164	0
unknown_Genus_of_Bifidobacteriaceae	0	0
Patulibacter minatonensis	0	0
unknown_Genus_of_Bacteroidaceae	392	0
unknown_Species_of_Bacteroides	0	0
Bacteroides graminisolvens	0	0
unknown_Species_of_Porphyromonas	0	0
Porphyromonas bennonis	0	0
unknown_Species_of_Proteiniphilum	0	0
unknown_Genus_of_Prevotellaceae	546	0
Alloprevotella rava	0	0
Prevotella buccalis	0	0
Prevotella sp.	0	0
Prevotella timonensis	0	0
unknown_Species_of_Hymenobacter	83	554
Hymenobacter sp.	96	68
unknown_Genus_of_Cryomorphaceae	345	0
Chryseobacterium hagamense	0	0
Chryseobacterium piscicola	0	72
ambiguous_Species_of_Empedobacter	67	100
Flavobacterium cauense	10	0
Flavobacterium rivuli	0	12
Myroides marinus	45	0
Myroides odoratus	0	0
unknown_Species_of_Wautersiella	71	0
unknown_Species_of_Mucilaginibacter	0	36
Pedobacter aquatilis	0	0
Pedobacter daejeonensis	0	0
Pedobacter ginsengisoli	0	0
Pedobacter panaciterrae	0	29
ambiguous_Species_of_Sphingobacterium	38	27
Sphingobacterium composti	11	0
Sphingobacterium shayense	0	0
Sphingobacterium thermophilum	10	0
unknown_Genus_of_Criblamydiaceae	0	0
ambiguous_Genus_of_Nostocaceae	0	0
unknown_Genus_of_Phormidiaceae	137	1284

unknown_Genus_of_Deinococcaceae	31	10
Fibrobacter succinogenes	76	0
unknown_Genus_of_Alicyclobacillaceae	0	0
unknown_Species_of_Amphibacillus	173	0
Bacillus anthracis	149	0
Bacillus cecembensis	179	13
Bacillus composti	0	0
Bacillus eiseniae	33	71
Bacillus gibsonii	0	0
Bacillus longiquaesitum	0	0
Gracilibacillus halotolerans	127	0
unknown_Species_of_Lysinibacillus	129	22
ambiguous_Species_of_Lysinibacillus	352	193
Lysinibacillus odysseyi	0	0
Lysinibacillus sinduriensis	271	10
Oceanobacillus chironomi	67	0
Oceanobacillus indicireducens	20	0
Oceanobacillus profundus	149	0
unknown_Species_of_Virgibacillus	736	0
unknown_Species_of_Gemella	0	0
ambiguous_Species_of_Gemella	0	0
unknown_Species_of_Paenibacillus	517	50
Paenibacillus hordei	148	86
Paenibacillus sp.	246	120
unknown_Species_of_Caryophanon	224	18
ambiguous_Species_of_Caryophanon	16	58
unknown_Species_of_Kurthia	320	0
Kurthia gibsonii	55	0
Kurthia sibirica	174	0
Lysinibacillus boronitolerans	0	0
ambiguous_Species_of_Planococcus	0	0
Planococcus donghaensis	39	0
Planococcus psychrotoleratus	0	0
Planococcus salinarum	70	0
Planococcus sp.	0	0
Planomicrobium glaciei	35	0
Planomicrobium stackebrandtii	0	0
Solibacillus silvestris	102	35
Viridibacillus arvi	141	15
unknown_Genus_of_Staphylococcaceae	0	0
unknown_Species_of_Jeotgalicoccus	203	0
Jeotgalicoccus pinnipedialis	30	0
ambiguous_Species_of_Macrococcus	43	0
Macrococcus caseolyticus	0	0
Salinicoccus alkaliphilus	0	0
Salinicoccus kunmingensis	0	0

unknown_Species_of_Staphylococcus	0	0
Staphylococcus arlettae	157	0
Staphylococcus cohnii	0	0
Staphylococcus lentus	18	0
unknown_Genus_of_Thermoactinomycetaceae	52	0
Facklamia hominis	0	10
unknown_Species_of_Globicatella	26	0
Globicatella sanguinis	0	0
Globicatella sulfidifaciens	96	75
ambiguous_Genus_of_Carnobacteriaceae	283	0
Atopostipes suicloacalis	329	0
ambiguous_Species_of_Carnobacterium	485	0
unknown_Species_of_Granulicatella	0	0
ambiguous_Species_of_Granulicatella	0	0
Granulicatella elegans	0	0
unknown_Genus_of_Enterococcaceae	0	0
ambiguous_Genus_of_Enterococcaceae	80	0
ambiguous_Species_of_Enterococcus	39	0
unknown_Genus_of_Lactobacillaceae	112	10
Lactobacillus equi	0	0
ambiguous_Species_of_Weissella	176	11
unknown_Species_of_Streptococcus	0	0
Streptococcus parauberis	0	0
Streptococcus salivarius	131	0
Streptococcus sp.	0	0
Clostridium hathewayi	352	0
Proteiniclasticum ruminis	0	0
unknown_Genus_of_ClostridialesFamilyXI.IncertaeSedis	276	0
ambiguous_Species_of_Anaerococcus	0	0
Anaerococcus hydrogenalis	0	0
Anaerococcus octavius	258	0
Finegoldia magna	48	0
Peptoniphilus coxii	0	0
Peptoniphilus harei	0	0
unknown_Species_of_Tissierella	16	0
Tissierella creatinini	10	0
unknown_Genus_of_Lachnospiraceae	1135	0
ambiguous_Genus_of_Lachnospiraceae	2021	17
ambiguous_Species_of_Blautia	38	0
unknown_Species_of_Coprococcus	228	0
Coprococcus eutactus	185	0
unknown_Genus_of_Peptoniphilaceae	119	0
ambiguous_Genus_of_Peptoniphilaceae	12	0
unknown_Species_of_Anaerococcus	44	0
unknown_Species_of_Finegoldia	27	0
unknown_Species_of_Helcococcus	11	0

unknown_Species_of_Peptoclostridium	0	0
unknown_Species_of_Ruminococcus	16	0
Ruminococcus gauvreauii	141	0
unknown_Genus_of_unclassifiedClostridiales	101	0
unknown_Genus_of_Erysipelotrichaceae	135	0
unknown_Genus_of_Veillonellaceae	32	0
unknown_Genus_of_Nitrospiraceae	14	0
unknown_Genus_of_Magnetococcaceae	44	0
unknown_Genus_of_Aurantimonadaceae	0	10
ambiguous_Genus_of_Bartonellaceae	12	10
Cucumibacter marinus	0	0
unknown_Species_of_Pelagibacterium	0	13
ambiguous_Species_of_Chelativorans	0	0
Nitratireductor lucknowense	0	0
Thermovum composti	0	0
Rhizobium radiobacter	0	107
Rhizobium tibeticum	0	12
Shinella daejeonensis	0	0
unknown_Genus_of_Xanthobacteraceae	21	0
ambiguous_Species_of_Xanthobacter	28	0
ambiguous_Genus_of_unclassifiedRhizobiales	11	0
Vasilyevaea mishustinii	0	0
Rhodobacter capsulatus	0	0
Rhodobacter sphaeroides	0	0
unknown_Species_of_Roseivivax	0	0
Thioclava sp.	0	0
unknown_Species_of_Tranquillimonas	0	0
Yangia pacifica	72	0
Novosphingobium barchaimii	0	63
Sphingomonas echinoides	33	0
Sphingomonas insulae	0	98
Sphingomonas melonis	39	48
Sphingomonas panni	0	41
Sphingomonas yunnanensis	0	0
ambiguous_Genus_of_Alcaligenaceae	27	91
Advenella incenata	0	0
Alcaligenes faecalis	0	0
Bordetella petrii	0	0
Paenalcaligenes hominis	42	144
unknown_Species_of_Pigmentiphaga	0	12
ambiguous_Species_of_Pigmentiphaga	0	13
unknown_Species_of_Pusillimonas	25	0
unknown_Genus_of_Burkholderiaceae	0	0
Burkholderia sp.	0	0
Comamonas sp.	262	0
Hydrogenophaga bisanensis	0	0

Hydrogenophaga sp.	0	0
Malikia spinosa	0	0
Pelomonas aquatica	0	0
Pelomonas puraquae	10	0
unknown_Species_of_Pseudacidovorax	0	110
Ramlibacter henchirensis	13	117
Variovorax boronicumulans	0	0
Variovorax paradoxus	98	36
ambiguous_Species_of_Duganella	0	322
Duganella phyllosphaerae	0	76
Duganella zoogloeoides	0	253
unknown_Species_of_Janthinobacterium	0	41
ambiguous_Species_of_Janthinobacterium	41	30
Janthinobacterium lividum	59	0
unknown_Species_of_Massilia	0	196
Massilia niabensis	0	123
Massilia timonae	0	180
Tepidiphilus thermophilus	0	0
unknown_Species_of_Neisseria	0	0
ambiguous_Species_of_Neisseria	0	0
unknown_Species_of_Azoarcus	0	0
Denitratisoma sp.	0	0
Petrobacter succinatimandens	0	0
unknown_Genus_of_Desulfobacteraceae	0	0
unknown_Genus_of_Desulfovibrionaceae	369	0
unknown_Genus_of_Geobacteraceae	73	0
unknown_Genus_of_Pelobacteraceae	23	0
unknown_Genus_of_Syntrophaceae	0	17
unknown_Genus_of_Syntrophobacteraceae	0	0
ambiguous_Species_of_Arcobacter	0	0
Arcobacter butzleri	0	0
Arcobacter cryaerophilus	25	0
Arcobacter skirrowii	0	0
Campylobacter ureolyticus	0	0
unknown_Genus_of_Helicobacteraceae	11	0
unknown_Genus_of_Aeromonadaceae	14	0
ambiguous_Species_of_Aeromonas	0	0
ambiguous_Species_of_Alishewanella	0	0
Alishewanella sp.	0	0
Marinimicrobium sp.	0	0
unknown_Species_of_Microbulbifer	236	35
unknown_Species_of_Teredinibacter	0	12
unknown_Genus_of_Idiomarinaceae	41	0
unknown_Genus_of_Ectothiorhodospiraceae	0	0
ambiguous_Species_of_Halothiobacillus	0	0
Citrobacter murliniae	0	0

Enterobacter sp.	0	0
unknown_Species_of_Erwinia	26	0
ambiguous_Species_of_Erwinia	270	16
Erwinia billingiae	0	0
Erwinia persicina	57	0
Erwinia rhapontici	188	0
Leclercia adecarboxylata	0	0
unknown_Species_of_Pantoea	0	0
Pantoea brenneri	202	50
Pantoea sp.	42	0
ambiguous_Species_of_Serratia	77	27
Serratia rubidaea	0	0
ambiguous_Genus_of_Legionellaceae	0	0
unknown_Species_of_Legionella	0	0
unknown_Genus_of_Methylothermaceae	69	13
unknown_Genus_of_Alcanivoracaceae	36	0
unknown_Genus_of_Hahellaceae	63	0
unknown_Genus_of_Halomonadaceae	0	0
Halomonas anticariensis	0	0
unknown_Species_of_Thalassolituus	0	0
Thalassolituus marinus	0	0
unknown_Genus_of_Orbaceae	38	0
unknown_Genus_of_Pasteurellaceae	0	0
unknown_Species_of_Actinobacillus	0	0
ambiguous_Species_of_Actinobacillus	0	0
Actinobacillus equuli	0	0
Actinobacillus indolicus	0	0
Acinetobacter baumannii	229	0
Acinetobacter beijerinckii	13	0
Acinetobacter haemolyticus	0	0
Acinetobacter schindleri	182	106
Acinetobacter towneri	48	658
Alkanindiges illinoisensis	87	11
unknown_Species_of_Moraxella	0	0
ambiguous_Species_of_Moraxella	0	0
Moraxella equi	0	0
Moraxella sp.	0	0
Azomonas agilis	79	0
Cellvibrio japonicus	0	0
Pseudomonas agarici	0	0
Pseudomonas bauzanensis	47	0
Pseudomonas caricapapayae	58	146
Pseudomonas chlororaphis	11	15
Pseudomonas cichorii	0	11
Pseudomonas cremoricolorata	44	0
Pseudomonas fuscovaginae	0	0

Pseudomonas graminis	98	11
Pseudomonas libanensis	31	13
Pseudomonas lutea	127	0
Pseudomonas orientalis	0	0
Pseudomonas punonensis	0	543
Pseudomonas savastanoi	13	196
Pseudomonas stutzeri	0	0
Pseudomonas viridiflava	77	0
Pseudomonas xiamenensis	0	0
Methylophaga lonarensis	0	0
unknown_Genus_of_Thiotrichaceae	13	0
unknown_Species_of_Achromatium	0	0
unknown_Species_of_Pseudoalteromonas	0	0
ambiguous_Genus_of_Vibrionaceae	10	0
unknown_Species_of_Dokdonella	10	0
Pseudoxanthomonas taiwanensis	0	0
Sphaerochaeta globosa	0	0
Acholeplasma pleciae	0	0
Corynebacterium callunae	18	0
Corynebacterium terpenotabidum	29	0
Brachybacterium muris	20	0
unknown_Species_of_Leucobacter	0	0
Leucobacter exalbidus	17	0
Micrococcus lylae	59	0
Nesterenkonia lacusekhoensis	25	0
unknown_Species_of_Zhihengliuella	0	0
Aeromicrobium tamlense	0	0
Nocardioides dokdonensis	0	0
Nocardioides dubius	44	0
Nocardioides exalbidus	62	0
Lysinibacillus sphaericus	0	0
unknown_Species_of_Sporosarcina	150	0
Staphylococcus gallinarum	0	0
Atopococcus tabaci	15	0
Enterococcus malodoratus	21	0
ambiguous_Genus_of_Eubacteriaceae	158	0
Eubacterium hallii	40	0
ambiguous_Genus_of_Peptostreptococcaceae	41	0
ambiguous_Species_of_Citreicella	0	0
Natronohydrobacter thiooxidans	0	0
unknown_Species_of_Paenirhodobacter	0	0
unknown_Species_of_Pseudorhodobacter	0	0
unknown_Species_of_Roseibaca	0	0
unknown_Species_of_Tabrizicola	17	0
Sphingopyxis italica	0	0
unknown_Species_of_Bordetella	0	0

unknown_Species_of_Castellaniella	0	0
Curvibacter delicatus	0	0
unknown_Genus_of_Acidithiobacillaceae	0	0
Zobellella taiwanensis	136	28
ambiguous_Genus_of_Alteromonadaceae	0	0
ambiguous_Species_of_Marinimicrobium	0	0
Aliidiomarina taiwanensis	38	0
ambiguous_Genus_of_Shewanellaceae	0	0
ambiguous_Genus_of_Ectothiorhodospiraceae	0	0
Erwinia aphidicola	0	0
ambiguous_Genus_of_Halomonadaceae	0	0
Pseudomonas seleniipraecipitans	0	0
unknown_Species_of_Spongiibacter	11	0
Georgenia satyanarayanai	0	0
ambiguous_Genus_of_Corynebacteriaceae	0	0
Brachybacterium sacelli	0	0
unknown_Genus_of_Dietziaceae	0	0
Dietzia lutea	0	0
unknown_Species_of_Stackebrandtia	0	0
unknown_Species_of_Janibacter	15	0
ambiguous_Species_of_Knoellia	0	0
Knoellia flava	48	0
Microbacterium aquimaris	0	0
Microbacterium sp.	0	0
Microbacterium suwonense	0	0
Arthrobacter bergerei	70	0
Arthrobacter sp.	62	0
unknown_Species_of_Citricoccus	0	0
Citricoccus nitrophenolicus	0	0
ambiguous_Species_of_Mycobacterium	14	0
unknown_Genus_of_Nakamurellaceae	15	0
Rhodococcus coprophilus	0	0
Nocardioides daejeonensis	0	0
ambiguous_Species_of_Nocardiopsis	168	0
Nocardiopsis listeri	0	0
ambiguous_Genus_of_Promicromonosporaceae	0	0
unknown_Species_of_Myceligenerans	0	0
unknown_Species_of_Microlunatus	0	0
unknown_Species_of_Propionibacterium	18	0
Propionibacterium propionicum	0	0
Saccharopolyspora sp.	1895	0
unknown_Species_of_Haloactinobacterium	0	0
Ruania albidiflava	0	0
Streptomyces daliensis	0	0
Streptomyces ramulosus	0	0
unknown_Species_of_Cytophaga	34	0

Flavobacterium myungsuense	0	0
Galbibacter marinus	0	0
Leeuwenhoekiella palythoae	0	0
ambiguous_Species_of_Subsaximicrobium	0	0
unknown_Species_of_Winogradskyella	0	0
unknown_Species_of_Parapedobacter	0	0
unknown_Species_of_Alkalibacillus	0	0
Bacillus thermolactis	14	0
Ornithinibacillus sp.	0	0
ambiguous_Species_of_Sporosarcina	102	0
ambiguous_Species_of_Aerococcus	0	0
Desemzia incerta	32	0
Isobaculum melis	33	0
unknown_Species_of_Enterococcus	43	0
Enterococcus asini	0	0
Enterococcus phoeniculicola	0	0
Weissella cibaria	67	0
unknown_Genus_of_Catabacteriaceae	0	0
unknown_Genus_of_Eubacteriaceae	387	0
unknown_Species_of_Saccharofermentans	0	0
unknown_Genus_of_Leptotrichiaceae	0	0
unknown_Species_of_Ketogulonicigenium	0	0
Paracoccus fistulariae	0	0
Paracoccus zeaxanthinifaciens	0	0
Sphingopyxis litoris	0	0
Sphingopyxis sp.	0	0
unknown_Genus_of_Campylobacteraceae	0	0
Acinetobacter radioresistens	64	102
Pseudomonas litoralis	0	0
ambiguous_Species_of_Methylophaga	0	0
Methylophaga muralis	0	0
Methylophaga thiooxydans	0	0
Luteimonas lutimaris	0	0
Simiduia areninigrae	0	0
unknown_Genus_of_Dethiosulfovibrionaceae	0	0
unknown_Genus_of_Synergistaceae	27	0
unknown_Species_of_Synergistes	0	0
unknown_Species_of_Actinomyces	0	0
Actinomyces dentalis	0	0
Actinomyces massiliensis	0	0
Actinomyces naeslundii	0	0
Actinomyces viscosus	0	0
Corynebacterium doosanense	0	0
Corynebacterium kroppenstedtii	0	0
Corynebacterium suicordis	0	0
Corynebacterium tuberculostearicum	209	0

ambiguous_Species_of_Dermacoccus	0	0
Dietzia cercidiphylli	0	0
Dietzia papillomatosis	0	0
Cryobacterium arcticum	0	0
Microbacterium lacticum	0	0
unknown_Species_of_Mycetocola	0	0
Kocuria salsicia	0	0
Micrococcus cohnii	0	0
Rothia dentocariosa	0	0
Rothia mucilaginosa	0	0
Aeromicrobium halocynthiae	0	0
Nocardiopsis salina	212	0
Propionibacterium granulosum	89	0
Saccharopolyspora gregorii	429	34
Saccharopolyspora halophila	55	97
unknown_Genus_of_Patulibacteraceae	0	0
unknown_Genus_of_Solirubrobacteraceae	10	0
Porphyromonas catoniae	0	0
ambiguous_Species_of_Prevotella	0	0
Olivibacter jilunii	0	0
Bacillus circulans	0	0
Bacillus simplex	42	0
ambiguous_Species_of_Exiguobacterium	0	0
Gemella haemolysans	0	0
Gemella morbillorum	0	0
Gemella sanguinis	0	0
Brochothrix thermosphacta	0	0
Paenibacillus camelliae	0	0
ambiguous_Species_of_Ureibacillus	0	0
Ureibacillus thermosphaericus	0	0
Staphylococcus capitis	0	0
Staphylococcus epidermidis	0	0
Staphylococcus hominis	46	0
Staphylococcus jettensis	0	0
Staphylococcus pasteuri	0	0
ambiguous_Species_of_Laceyella	0	0
Abiotrophia defectiva	0	0
Granulicatella adiacens	33	0
ambiguous_Genus_of_Lactobacillaceae	0	0
ambiguous_Species_of_Lactobacillus	26	0
Lactobacillus iners	0	0
ambiguous_Species_of_Lactococcus	61	0
Lactococcus lactis	133	0
Streptococcus constellatus	0	0
Streptococcus gordonii	0	0
Streptococcus infantis	0	0

Streptococcus pseudopneumoniae	18	0
Streptococcus sanguinis	0	0
Streptococcus thermophilus	0	0
Anaerococcus murdochii	0	0
Anaerococcus prevotii	0	0
Peptoniphilus gorbachii	0	0
Eubacterium nodatum	0	0
ambiguous_Species_of_Mogibacterium	0	0
Eubacterium hadrum	13	0
ambiguous_Species_of_Roseburia	0	0
ambiguous_Species_of_Peptoniphilus	21	0
Filifactor alocis	0	0
Peptostreptococcus stomatis	0	0
Faecalibacterium prausnitzii	0	0
Ruminococcus bromii	0	0
ambiguous_Species_of_Veillonella	0	0
unknown_Genus_of_Acidaminococcaceae	125	10
Negativicoccus succinicivorans	0	0
Selenomonas noxia	0	0
Veillonella alcalescens	0	0
Veillonella parvula	0	0
Veillonella rogosae	0	0
Veillonella tobetsuensis	0	0
Brevundimonas vancanneytii	0	0
ambiguous_Species_of_Caulobacter	0	0
Caulobacter segnis	0	0
Gemmiger formicilis	0	0
Methylobacterium radiotolerans	0	0
Paracoccus caeni	0	0
Bordetella sp.	18	0
Ralstonia pickettii	0	0
Oxalobacter sp.	0	0
Tepidimonas taiwanensis	0	0
ambiguous_Genus_of_Hydrogenophilaceae	10	0
Neisseria perflava	0	0
Neisseria subflava	0	0
ambiguous_Species_of_Campylobacter	0	0
ambiguous_Species_of_Haemophilus	0	0
Acinetobacter soli	0	0
Enhydrobacter aerosaccus	48	0
Moraxella osloensis	0	0
Photobacterium phosphoreum	0	0
Thermomonas hydrothermalis	0	0
unknown_Genus_of_Holophagaceae	14	0
unknown_Species_of_Flaviflexus	0	0
unknown_Species_of_Mobiluncus	0	0

Mobiluncus curtisii	0	0
Mobiluncus mulieris	0	0
ambiguous_Genus_of_Bogoriellaceae	0	0
Brevibacterium pityocampae	0	0
Corynebacterium epidermidicanis	0	0
Corynebacterium freneyi	0	0
Corynebacterium propinquum	0	0
Dermabacter hominis	0	0
ambiguous_Species_of_Geodermatophilus	17	0
unknown_Species_of_Knoellia	0	0
unknown_Species_of_Kineococcus	0	0
ambiguous_Species_of_Kineococcus	0	0
Kineococcus radiotolerans	0	0
Kineococcus sp.	0	0
ambiguous_Species_of_Pseudokineococcus	0	0
Agrococcus jenensis	0	0
Curtobacterium citreum	0	0
Curtobacterium pusillum	0	0
Gulosibacter molinativorax	0	0
Leucobacter sp.	0	0
Microbacterium phyllosphaerae	0	0
Arthrobacter gandavensis	14	0
Kocuria gwangalliensis	0	0
ambiguous_Species_of_Nesterenkonia	0	0
ambiguous_Species_of_Nocardia	0	0
Rhodococcus cerastii	0	10
Nocardioides alpinus	17	0
Nocardioides daedukensis	29	0
Nocardioides islandensis	111	0
Nocardioides oleivorans	101	0
Promicromonospora kroppenstedtii	0	0
Luteococcus japonicus	0	0
unknown_Species_of_Tessaracoccus	0	0
ambiguous_Species_of_Actinoalloteichus	302	0
Pseudonocardia sp.	120	0
Streptomyces macrosporus	63	0
unknown_Species_of_Gaiella	468	0
Bacteroides massiliensis	0	0
unknown_Species_of_Prevotella	73	0
Cytophaga sp.	0	0
Hymenobacter metalli	0	0
Hymenobacter soli	0	0
Chryseobacterium yonginense	95	0
Epilithonimonas lactis	0	0
ambiguous_Species_of_Myroides	0	0
Myroides odoratimimus	0	0

Myroides profundi	0	0
unknown_Genus_of_Saprospiraceae	0	0
Sphingobacterium nematocida	19	0
unknown_Genus_of_Dehalococcoidaceae	0	0
unknown_Genus_of_unclassifiedDehalococcoidia	105	0
unknown_Genus_of_Fibrobacteraceae	57	0
unknown_Species_of_Fibrobacter	88	0
Fibrobacter succinogenessubsp.succinogenes	59	0
Bacillus benzoevorans	0	0
Bacillus soli	0	0
Bacillus thermocloaceae	0	0
unknown_Species_of_Gracilibacillus	0	0
Oceanobacillus caeni	0	0
Streptohalobacillus salinus	0	0
ambiguous_Species_of_Paenibacillus	54	45
Paenibacillus illinoisensis	0	10
Caryophanon tenue	0	0
Planococcus kocurii	0	0
unknown_Species_of_Rummeliibacillus	18	0
Rummeliibacillus pycnus	47	0
Jeotgalicoccus halophilus	0	0
Staphylococcus simulans	0	0
ambiguous_Species_of_Thermoactinomyces	643	222
Thermoactinomyces vulgaris	702	536
unknown_Species_of_Aerococcus	0	0
unknown_Species_of_Alkalibacterium	0	0
Alkalibacterium iburiense	0	0
unknown_Species_of_Atopococcus	0	0
Enterococcus cecorum	0	0
Enterococcus gallinarum	0	0
Leuconostoc carnosum	42	0
Weissella paramesenteroides	0	0
Lactococcus taiwanensis	127	0
unknown_Genus_of_Christensenellaceae	120	0
Eubacterium ramulus	48	0
unknown_Genus_of_Gracilibacteraceae	33	0
Dorea formicigenerans	36	0
unknown_Species_of_Roseburia	233	0
[Ruminococcus] gnavus	33	0
[Ruminococcus] torques	0	0
unknown_Genus_of_Oscillospiraceae	0	0
unknown_Species_of_Peptoniphilus	0	0
unknown_Genus_of_Proteinivoraceae	46	0
ambiguous_Genus_of_Ruminococcaceae	31	0
unknown_Species_of_Faecalibacterium	0	0
Ruminococcus flavefaciens	183	0

unknown_Genus_of_Fusobacteriaceae	0	0
unknown_Species_of_Camelimonas	0	0
ambiguous_Species_of_Ochrobactrum	0	0
Pseudochrobactrum saccharolyticum	0	25
ambiguous_Genus_of_Methylobacteriaceae	0	0
Methylobacterium cerastii	0	0
Methylobacterium gossipiicola	0	78
Microvirga aerilata	157	0
Microvirga aerophila	0	0
Donghicola eburneus	0	0
Paracoccus marinus	0	0
unknown_Species_of_Sphingomicrobium	0	0
Sphingomonas abaci	0	56
Sphingomonas ginsengisoli	34	0
Sphingomonas kaistensis	0	0
Sphingomonas sediminicola	179	0
Alcaligenes sp.	46	0
unknown_Species_of_Eoetvoesia	0	0
Parapusillimonas granuli	0	0
ambiguous_Species_of_Pusillimonas	0	0
unknown_Genus_of_Comamonadaceae	320	10
Massilia jejuensis	11	0
unknown_Genus_of_Sutterellaceae	76	0
Iodobacter arcticus	0	0
unknown_Genus_of_Nitrosomonadaceae	40	0
unknown_Genus_of_Myxococcaceae	17	0
unknown_Genus_of_Succinivibrionaceae	0	0
unknown_Species_of_Aliidiomarina	0	0
Idiomarina sp.	0	0
ambiguous_Genus_of_Pseudoalteromonadaceae	0	0
ambiguous_Genus_of_Psychromonadaceae	0	0
ambiguous_Species_of_Shewanella	0	0
unknown_Genus_of_Arenicellaceae	84	0
unknown_Species_of_Rheinheimera	0	0
Rheinheimera aquimaris	0	0
Rheinheimera pacifica	0	0
Rheinheimera perlucida	0	0
Rheinheimera sp.	0	0
unknown_Genus_of_Thioalkalispiraceae	0	0
ambiguous_Species_of_Enterobacter	0	0
Erwinia tasmaniensis	0	39
unknown_Genus_of_Coxiellaceae	0	0
unknown_Species_of_Halomonas	0	0
Halomonas beimenensis	0	0
Halomonas pantelleriensis	0	0
Pseudomonas xanthomarina	0	0

ambiguous_Genus_of_Thiotrichaceae	0	0
Lysobacter xinjiangensis	81	0
Treponema calligyrum	0	0
Illumatobacter nonamiense	0	0
unknown_Species_of_Iamia	34	0
unknown_Genus_of_Acidothermaceae	35	0
ambiguous_Species_of_Actinomyces	0	0
unknown_Genus_of_Actinopolysporaceae	0	27
Catenulispora graminis	0	0
unknown_Genus_of_Geodermatophilaceae	36	0
Arsenicicoccus bolidensis	37	0
Tetrasphaera duodecadis	15	0
unknown_Species_of_Jiangella	0	0
ambiguous_Species_of_Agromyces	0	0
Leucobacter tardus	10	0
Mycetocola reblochoni	0	0
Longispora fulva	14	0
unknown_Genus_of_Mycobacteriaceae	10	38
ambiguous_Species_of_Kribbella	10	0
Nocardioides aestuarii	14	0
Nocardioides agariphilus	0	0
Nocardioides dilutus	21	0
Nocardioides halotolerans	0	0
unknown_Species_of_Nocardiopsis	62	0
Nocardiopsis alba	59	0
Nocardiopsis chromatogenes	0	0
Nocardiopsis dassonvillei	20	0
Nocardiopsis halotolerans	0	0
Nocardiopsis lucentensis	0	0
Nocardiopsis nikkonensis	80	0
unknown_Genus_of_Promicromonosporaceae	0	0
ambiguous_Species_of_Isoptericola	0	0
Actinomycetospora sp.	0	0
unknown_Species_of_Prauserella	0	0
ambiguous_Species_of_Pseudonocardia	13	10
Saccharomonospora azurea	103	0
Saccharopolyspora cebuensis	0	0
Saccharopolyspora hordei	34	0
Saccharopolyspora pogona	0	0
Saccharopolyspora rectivirgula	115	39
Saccharopolyspora spinosa	0	0
Streptomyces acidiscabies	11	0
Streptomyces carpaticus	76	0
Streptomyces cheonanensis	41	0
Streptomyces guanduensis	14	0
Streptomyces xiamenensis	62	0

unknown_Genus_of_Thermomonosporaceae	28	0
unknown_Genus_of_Gaiellaceae	484	0
unknown_Genus_of_Rubrobacteraceae	135	0
unknown_Species_of_Solirubrobacter	29	0
unknown_Genus_of_Marinilabiliaceae	0	0
unknown_Genus_of_Rikenellaceae	147	0
Chryseobacterium chaponense	0	0
Flavobacterium sinopsychrotolerans	0	0
unknown_Genus_of_Rhabdochlamydiaceae	0	0
unknown_Genus_of_Herpetosiphonaceae	0	0
Bacillus clausii	1244	0
Bacillus idriensis	65	0
Bacillus kribbensis	15	0
Bacillus licheniformis	4896	0
Bacillus rhizosphaerae	3373	0
ambiguous_Species_of_Gracilibacillus	0	0
ambiguous_Species_of_Marinococcus	0	0
Ornithinibacillus halophilus	158	0
ambiguous_Species_of_Psychrobacillus	0	0
ambiguous_Species_of_Terribacillus	47	0
Virgibacillus pantothenticus	0	0
unknown_Genus_of_Paenibacillaceae	144	0
ambiguous_Genus_of_Paenibacillaceae	36	0
Planococcus antarcticus	0	0
unknown_Species_of_Melghirimyces	0	0
Melghirimyces thermohalophilus	66	0
unknown_Species_of_Thermoactinomyces	65	0
Thermoactinomyces intermedius	0	0
unknown_Species_of_Thermoflavimicrobium	0	0
Enterococcus sp.	0	0
unknown_Genus_of_Leuconostocaceae	0	0
Clostridium sp.	29	0
unknown_Species_of_Acidaminobacter	0	0
unknown_Species_of_Eubacterium	0	0
Roseburia faecis	60	0
unknown_Species_of_Acetivibrio	95	0
Ruminococcus faecis	38	0
Ruminococcus sp.	0	0
unknown_Species_of_Nitrospira	20	0
Bradyrhizobium canariense	0	0
Rhodoblastus sphagnicola	0	0
ambiguous_Species_of_Salinarimonas	0	0
Rhodoplanes sp.	0	0
unknown_Species_of_Thermovum	30	0
Ensifer(Sinorhizobium) adhaerens	0	0
Bauldia litoralis	0	0

unknown_Species_of_Rhodovulum	0	0
Skermanella aerolata	120	0
unknown_Genus_of_Holosporaceae	13	0
unknown_Genus_of_Geminicoccus	67	0
Thiobacillus denitrificans	0	0
unknown_Genus_of_Chromobacteriaceae	0	0
unknown_Genus_of_Desulfonatronumaceae	0	0
unknown_Genus_of_Phaselicystidaceae	0	0
Hafnia paralvei	0	0
Rosenbergiella nectarea	171	0
Serratia quinivorans	20	0
Luteimonas abyssi	37	0
Frankia sp.	35	0
unknown_Genus_of_Glycomycetaceae	0	0
Propionibacterium sp.	0	0
unknown_Species_of_Saccharomonospora	0	0
Sanguibacter keddieii	10	103
Streptomyces sedi	0	0
Olivibacter terrae	0	0
Lactobacillus hayakitensis	84	0
unknown_Species_of_Jahnella	0	0
Cronobacter turicensis	0	10
unknown_Species_of_Serratia	0	0
Pseudomonas thermotolerans	0	0
Pseudoxanthomonas spadix	0	0
unknown_Species_of_Eggerthella	0	0
unknown_Species_of_Rufibacter	0	0
Aequorivita capsosiphonis	0	0
unknown_Species_of_Gelidibacter	0	0
Gelidibacter mesophilus	0	0
unknown_Species_of_Salegentibacter	0	0
Salegentibacter salarius	0	0
Salinimicrobium marinum	0	0
Vitellibacter aestuarii	0	0
unknown_Genus_of_Ardenticatenaceae	0	10
Lysinibacillus meyeri	157	0
unknown_Species_of_Saccharibacillus	0	0
Aureimonas phyllosphaerae	0	32
ambiguous_Species_of_Phyllobacterium	0	0
Paracoccus limosus	0	0
Sphingomonas endophytica	0	46
Sphingomonas paucimobilis	0	99
Sphingomonas yabuuchiae	0	0
Oxalicibacterium flavum	0	0
Aquabacterium citratiphilum	0	0
Tepidiphilus margaritifer	0	0

unknown_Genus_of_Sandaracinaceae	0	0
Rheinheimera chironomi	0	0
Citrobacter gillenii	0	0
Escherichia vulneris	0	0
Pantoea cypripedii	0	0
Yokenella regensburgei	0	0
unknown_Species_of_Amphritea	0	0
Pseudomonas abietaniphila	0	0
Pseudomonas psychrotolerans	0	135
Thioalkalimicrobium cyclicum	0	0
unknown_Genus_of_Sinobacteraceae	10	0
Stenotrophomonas terrae	0	0
unknown_Species_of_Opitutus	0	0
ambiguous_Species_of_Modestobacter	35	0
Kineococcus glutinatus	0	0
unknown_Species_of_Leifsonia	0	0
Microbacterium mitrae	0	0
Arthrobacter viscosus	0	0
Yaniella halotolerans	0	0
unknown_Species_of_Promicromonospora	0	0
ambiguous_Genus_of_Sporichthyaceae	0	0
Patulibacter ginsengiterrae	0	0
unknown_Species_of_Dyadobacter	0	0
unknown_Species_of_Lishizhenia	0	0
unknown_Species_of_Aquimarina	0	0
Empedobacter brevis	0	0
Flavobacterium flevense	0	0
Flavobacterium subsaxonicum	0	0
unknown_Species_of_Galbibacter	0	0
Ulvibacter antarcticus	0	0
unknown_Species_of_CandidatusRhabdochlamydia	0	0
Paenibacillus xinjiangensis	0	0
unknown_Species_of_Salinicoccus	0	0
Shimazuella kribbensis	0	0
Carnobacterium jeotgali	125	0
unknown_Species_of_Aureimonas	0	0
ambiguous_Species_of_Afipia	0	0
unknown_Species_of_Pseudochrobactrum	0	0
Pseudochrobactrum lubricantis	0	0
unknown_Species_of_Aminobacter	0	0
unknown_Species_of_Sphingobium	0	0
Sphingobium chungbukense	0	0
Sphingobium rhizovicinum	0	0
Advenella sp.	0	0
Bordetella ansorpii	0	0
Pusillimonas harenae	0	0

Comamonas terrigena	0	0
unknown_Species_of_Pseudorhodoferax	0	0
unknown_Species_of_Simplicispira	0	0
unknown_Species_of_Variovorax	0	19
ambiguous_Species_of_Herbaspirillum	0	49
Herbaspirillum frisingense	0	0
ambiguous_Genus_of_Bacteriovoracaceae	0	0
unknown_Genus_of_Cystobacteraceae	0	0
unknown_Species_of_Saccharophagus	0	0
unknown_Genus_of_Alteromonadalesgeneraincertaesedis	0	0
Serratia proteamaculans	0	0
unknown_Genus_of_Legionellaceae	0	0
ambiguous_Species_of_Halomonas	0	24
Halomonas muralis	0	0
unknown_Species_of_Halotalea	0	0
unknown_Species_of_Azotobacter	0	0
Pseudomonas aeruginosa	0	0
Pseudomonas borbori	0	0
Pseudomonas caeni	0	0
Pseudomonas pseudoalcaligenes	0	0
unknown_Genus_of_Vibrionaceae	0	0
Luteimonas aestuarii	0	0
Lysobacter concretionis	0	0
unknown_Genus_of_Bryobacter	13	0
Iamia sp.	10	0
ambiguous_Genus_of_Catenulisporaceae	14	0
ambiguous_Species_of_Cryptosporangium	10	0
Geodermatophilus siccatus	24	0
unknown_Species_of_Modestobacter	11	0
Humibacillus xanthopallidus	11	0
Tetrasphaera elongata	10	0
ambiguous_Genus_of_Jiangellaceae	10	0
Angustibacter peucedani	17	0
Frondihabitans sp.	30	0
Arthrobacter russicus	11	0
Virgisporangium ochraceum	37	0
ambiguous_Genus_of_Nakamurellaceae	11	0
Rhodococcus artemisiae	13	0
Nocardioides hwasunensis	61	0
unknown_Genus_of_Nocardiopsaceae	46	0
Nocardiopsis exhalans	10	0
Actinoalloteichus cyanogriseus	23	0
Actinoalloteichus hymeniacidonis	52	0
ambiguous_Species_of_Actinomycetospora	15	0
Saccharopolyspora shandongensis	32	0
ambiguous_Species_of_Streptacidiphilus	36	0

Streptomyces sodiiphilus	24	0
unknown_Genus_of_unclassifiedPseudonocardineae	12	0
unknown_Species_of_Denitrobacterium	12	0
unknown_Genus_of_Armatimonadaceae	15	0
Cloacibacterium normanense	17	0
Pseudosphingobacterium domesticum	10	0
unknown_Genus_of_Roseiflexaceae	10	0
unknown_Genus_of_Spirulinaceae	35	0
unknown_Species_of_Crinalium	103	0
Crinalium epipsammum	147	0
Bacillus litoralis	36	0
Bacillus sonorensis	10	0
Lysinibacillus contaminans	31	0
Oceanobacillus kimchii	37	0
Virgibacillus byunsanensis	30	0
ambiguous_Species_of_Ammoniphilus	12	0
Ammoniphilus oxalaticus	59	0
Paenibacillus alvei	20	0
Paenibacillus ginsengisoli	26	0
Sporosarcina sp.	12	0
ambiguous_Species_of_Leuconostoc	72	0
Clostridium butyricum	17	0
Clostridium saccharoperbutylacetonicum	10	0
unknown_Species_of_Salimesophilobacter	12	0
unknown_Species_of_Fusibacter	10	0
Eubacterium ventriosum	31	0
Butyrivibrio crossotus	12	0
unknown_Species_of_Lachnobacterium	11	0
Ruminococcus gnavus	136	0
Anaerococcus sp.	12	0
unknown_Species_of_Parvularcula	11	0
Methylocella sp.	16	0
unknown_Species_of_Hyphomicrobium	12	0
Methylobacterium jeotgali	12	0
Microvirga sp.	11	0
unknown_Species_of_Methylarcula	12	0
Pelagibius litoralis	41	0
unknown_Species_of_Skermanella	11	0
Sphingomonas japonica	12	0
Sphingomonas sanxanigenens	27	0
Polynucleobacter necessarius	10	0
Delftia litopenaei	43	0
unknown_Species_of_Methylibium	23	0
Polaromonas sp.	23	0
Variovorax sp.	10	0
Herminiimonas fonticola	129	0

unknown_Species_of_Thiobacillus	14	0
ambiguous_Species_of_Methylobacillus	10	0
Methylobacillus sp.	53	0
unknown_Species_of_CandidatusDactylopiibacterium	32	0
Thauera sp.	10	0
unknown_Genus_of_Desulfuromonadaceae	25	0
Geobacter psychrophilus	10	0
unknown_Genus_of_Anaeromyxobacteraceae	10	0
unknown_Genus_of_Kofleriaceae	11	0
ambiguous_Genus_of_Myxococcaceae	10	0
ambiguous_Species_of_Microbulbifer	10	0
Microbulbifer marinus	45	0
Trabulsiella odontotermitis	10	0
ambiguous_Species_of_Yersinia	19	0
Yersinia intermedia	26	0
Steroidobacter denitrificans	11	0
unknown_Species_of_Ignatzschineria	11	0
unknown_Genus_of_Chthoniobacter	12	0
Nesterenkonia alba	0	0
Prauserella sp.	0	0
unknown_Species_of_Geofilum	0	0
Mariniflexile sp.	0	0
unknown_Species_of_Sediminibacter	0	0
unknown_Species_of_Zobellia	0	0
unknown_Species_of_[Ruminococcus]	0	0
unknown_Species_of_Oscillibacter	0	0
unknown_Genus_of_Oligosphaeraceae	0	0
Pelistega sp.	0	0
Hydrogenophaga flava	0	0
ambiguous_Species_of_Thauera	0	0
Arcobacter trophiarum	0	0
Alcanivorax indicus	0	0
unknown_Species_of_Beggiatoa	0	0
Luteibacter rhizovicinus	0	0
Luteimonas marina	0	0
Arcanobacterium hippocoleae	0	0
Corynebacterium uterequi	0	0
unknown_Species_of_Oryzihumus	0	0
unknown_Species_of_Propioniferax	0	0
Rhizobium daejeonense	0	0
ambiguous_Species_of_Ketogulonicigenium	0	0
ambiguous_Genus_of_Methylophilaceae	0	0
unknown_Species_of_Desulfovibrio	0	0
unknown_Genus_of_Cardiobacteriaceae	0	0
Dyella sp.	0	0
Fulvimonas soli	0	0

unknown_Genus_of_Mariprofundaceae	0	0
unknown_Species_of_Treponema	0	0
Beutenbergia cavernae	0	0
Leifsonia kribbensis	0	0
Microbacterium foliorum	0	14
Yaniella flava	0	0
Leadbetterella byssophila	0	0
unknown_Genus_of_Caldilineaceae	0	0
Atopostipes sp.	0	0
unknown_Species_of_Jeotgalibaca	0	0
Trichococcus flocculiformis	0	0
Enterococcus casseliflavus	0	0
Enterococcus saccharolyticus	0	0
Devosia albogilva	0	0
Devosia terrae	0	0
Devosia yakushimensis	0	0
Loktanella hongkongensis	0	0
ambiguous_Species_of_Rhodovulum	0	0
Sphingopyxis alaskensis	0	0
Bordetella trematum	0	0
unknown_Species_of_Parapusillimonas	0	0
ambiguous_Species_of_Giesbergeria	0	0
unknown_Species_of_Hydrogenophaga	0	0
Simplicispira metamorpha	0	0
Vitreoscilla stercoraria	0	0
unknown_Genus_of_Thermithiobacillaceae	0	0
unknown_Species_of_Ectothiorhodosinus	0	0
ambiguous_Species_of_Buttiauxella	0	0
Enterobacter amnigenus	0	0
Plesiomonas shigelloides	0	47
Legionella lytica	0	0
unknown_Species_of_Methylohalobius	0	0
Thalassolituus oleivorans	0	0
unknown_Species_of_Perlucidibaca	0	0
Pseudomonas otitidis	0	0
Acholeplasma cavigenitalium	0	0
Modestobacter multiseptatus	0	0
unknown_Genus_of_Jonesiaceae	0	0
Labedella gwakjiensis	0	0
Rhodococcus sp.	0	0
Aeromicrobium fastidiosum	0	0
Actinomycetospora chiangmaiensis	0	0
unknown_Genus_of_Ruaniaceae	0	0
unknown_Species_of_Sanguibacter	0	0
Streptacidiphilus sp.	0	0
Streptomyces rapamycinicus	0	0

unknown_Genus_of_Nitriliruptoraceae	0	0
Bacteroides vulgatus	0	0
unknown_Species_of_Gilvibacter	0	0
Paenisporosarcina quisquiliarum	0	0
unknown_Genus_of_Victivallaceae	0	13
Brevundimonas aurantiaca	0	0
Brevundimonas basaltis	0	0
Phenylobacterium immobile	0	0
Bartonella rattaustraliani	0	0
unknown_Species_of_Rhodoplanes	0	0
Methylobacterium mesophilicum	0	39
Aliihoeflea aestuarii	0	0
Rhizobium rosettiformans	0	0
ambiguous_Genus_of_Xanthobacteraceae	0	0
unknown_Genus_of_unclassifiedRhizobiales	0	0
Paracoccus yeei	0	0
Rhodobacter blasticus	0	0
Roseococcus suduntuyensis	0	0
Sphingomonas pituitosa	0	0
unknown_Species_of_Stakelama	0	0
Achromobacter sp.	0	28
Oxalicibacterium horti	0	0
ambiguous_Genus_of_Chromobacteriaceae	0	0
ambiguous_Genus_of_Neisseriaceae	0	0
unknown_Species_of_Marinobacter	0	0
Marinobacter sp.	0	0
ambiguous_Species_of_Saccharophagus	0	0
unknown_Genus_of_Pseudoalteromonadaceae	0	0
Halomonas koreensis	0	0
Haemophilus haemolyticus	0	0
unknown_Species_of_Dasania	0	0
unknown_Species_of_Arenimonas	0	0
unknown_Species_of_Xanthomonas	0	0
unknown_Genus_of_OMGgroup	0	0
unknown_Genus_of_Opitutaceae	0	0
ambiguous_Genus_of_Brevibacteriaceae	0	0
Corynebacterium hansenii	0	0
Demequina lutea	0	0
unknown_Species_of_Helcobacillus	0	0
Modestobacter sp.	0	0
Janibacter corallicola	0	0
Leifsonia sp.	0	0
Leucobacter denitrificans	0	0
Microbacterium halophilum	0	0
Microbacterium sediminis	0	0
Pseudoclavibacter bifida	0	0

Pseudoclavibacter sp.	0	0
Arthrobacter monumenti	0	0
Arthrobacter pigmenti	0	0
Micrococcus luteus	0	0
Rothia nasimurium	0	0
Rothia terrae	0	0
ambiguous_Genus_of_Streptosporangiaceae	0	0
unknown_Genus_of_Hydrogenothermaceae	0	0
Hymenobacter perfusus	0	0
Aequorivita sp.	0	0
Chryseobacterium gregarium	0	0
ambiguous_Species_of_Mesonia	0	0
Salegentibacter sp.	0	0
Salinimicrobium sp.	0	0
Sphingobacterium kitahiroshimense	0	0
unknown_Genus_of_Pseudanabaenaceae	0	0
Bacillus graminis	0	10
Bacillus herbersteinensis	0	0
Marinococcus halotolerans	0	0
Kurthia sp.	0	0
ambiguous_Genus_of_Staphylococcaceae	0	0
unknown_Species_of_Macrococcus	0	0
Salinicoccus halitifaciens	0	0
Salinicoccus roseus	0	0
Facklamia languida	0	0
Ignavigranum ruoffiae	0	0
Marinilactibacillus psychrotolerans	0	0
unknown_Species_of_Bavariicoccus	0	0
unknown_Genus_of_Streptococcaceae	0	0
Lactococcus plantarum	0	0
Streptococcus dentasini	0	0
Streptococcus hyovaginalis	0	0
Streptococcus merionis	0	0
Streptococcus ovis	0	0
Streptococcus plurextorum	0	0
Streptococcus porci	0	0
Streptococcus pyogenes	0	0
Streptococcus rupicaprae	0	0
Streptococcus suis	0	0
Clostridium chromiireducens	0	0
Peptostreptococcus anaerobius	0	0
unknown_Species_of_Erysipelothrix	0	0
unknown_Species_of_Brevundimonas	0	0
Nitratireductor sp.	0	0
unknown_Species_of_Parvibaculum	0	0
unknown_Species_of_Loktanella	0	0

ambiguous_Species_of_Lautropia	0	0
Lautropia sp.	0	0
unknown_Species_of_Methylophilus	0	0
unknown_Species_of_Methylovorus	0	0
unknown_Species_of_Aquaspirillum	0	0
unknown_Species_of_Alysiella	0	0
ambiguous_Species_of_Alysiella	0	0
Alysiella crassa	0	0
unknown_Species_of_Bergeriella	0	0
Kingella denitrificans	0	0
Neisseria bacilliformis	0	0
Neisseria canis	0	0
Neisseria shayeganii	0	0
Neisseria wadsworthii	0	0
Uruburuella suis	0	0
unknown_Species_of_Denitromonas	0	0
Thauera phenylacetica	0	0
Bacteriovorax sp.	0	0
Arcobacter sp.	0	0
Suttonella ornithocola	0	0
Mangrovibacter plantisponsor	0	0
unknown_Species_of_Photorhabdus	0	0
Xenorhabdus bovienii	0	0
Legionella oakridgensis	0	0
unknown_Species_of_Pseudospirillum	0	0
Actinobacillus hominis	0	0
Actinobacillus lignieresii	0	0
Actinobacillus suis	0	0
unknown_Species_of_Aggregatibacter	0	0
unknown_Species_of_Avibacterium	0	0
unknown_Species_of_Lonepinella	0	0
Mannheimia varigena	0	0
Pasteurella caballi	0	0
Moraxella canis	0	0
Moraxella oblonga	0	0
unknown_Species_of_Pseudofulvimonas	0	0
unknown_Species_of_Sphaerochaeta	0	0
Sphaerochaeta sp.	0	0
ambiguous_Species_of_Acholeplasma	0	0
Acholeplasma hippikon	0	0
unknown_Genus_of_Spiroplasmataceae	0	0
Corynebacterium nuruki	0	0
Blastococcus sp.	0	0
Geodermatophilus soli	0	0
ambiguous_Species_of_Jonesia	0	0
Jonesia quinghaiensis	0	0

Nocardioides kongjuensis	0	0
Pseudonocardia antitumoralis	0	0
Sanguibacter marinus	0	0
Sanguibacter suarezii	0	0
unknown_Species_of_Adlercreutzia	0	0
unknown_Genus_of_Prolixibacteraceae	0	0
unknown_Species_of_Cryomorpha	0	0
Flavobacterium aquatile	0	0
Flavobacterium macrobrachii	0	0
Flavobacterium oncorhynchi	0	0
unknown_Species_of_Mariniflexile	0	0
Mesonia ostreae	0	0
unknown_Species_of_Muricauda	0	0
Sediminibacter sp.	0	0
Sphingobacterium bambusae	0	0
Psychrobacillus psychrodurans	0	0
Paenibacillus daejeonensis	0	0
Paenibacillus montaniterrae	0	0
Crocinobacterium jejui	0	0
Clostridium aminobutyricum	0	0
unknown_Species_of_Mogibacterium	0	0
unknown_Species_of_Alkalibacter	0	0
unknown_Genus_of_Cohaesibacteraceae	0	0
Devosia insulae	0	0
Nitratireductor basaltis	0	0
Phyllobacterium brassicacearum	0	0
Neorhizobium galegae	0	0
Rhodoligotrophos appendicifer	0	0
Xanthobacter tagetidis	0	0
ambiguous_Species_of_Hyphomonas	0	0
unknown_Species_of_Amaricoccus	0	0
Roseomonas sp.	0	0
ambiguous_Species_of_Rickettsia	0	0
Candidimonas nitroreducens	0	0
unknown_Species_of_Pandoraea	0	0
Inhella inkyongensis	0	0
Methylophilus flavus	0	0
unknown_Species_of_Nitrosomonas	0	0
unknown_Species_of_Denitratisoma	0	0
Thauera aminoaromatica	0	0
Uliginosibacterium gangwonense	0	0
unknown_Species_of_Nannocystis	0	0
Rheinheimera soli	0	0
Enterobacter nimipressuralis	0	0
Erwinia toletana	0	0
Salmonella enterica	0	0

Serratia marcescens	0	0
Methylobacter marinus	0	0
unknown_Species_of_Methylomicrobium	0	0
Perlucidibaca piscinae	0	0
Pseudomonas composti	0	0
Pseudomonas cuatrocienegasensis	0	0
ambiguous_Species_of_Vibrio	0	0
unknown_Species_of_Alkalimonas	0	0
unknown_Species_of_Pyramidobacter	0	0
Tetrasphaera sp.	0	0
Microbacterium terricola	0	11
Murinocardiopsis flavida	0	0
Nocardiopsis metallicus	0	0
Promicromonospora citrea	0	0
Promicromonospora sp.	0	0
Streptomyces sannurensis	0	0
Dyadobacter hamtensis	0	0
Sarcina maxima	0	0
Rhizobium pusense	0	0
ambiguous_Species_of_Porphyrobacter	0	0
Hydrogenophaga taeniospiralis	0	0
unknown_Species_of_Eionea	0	0
unknown_Species_of_Methylobacter	0	0
unknown_Species_of_Methylosarcina	0	0
Duganella radicis	0	0
Duganella sp.	0	0
Massilia plicata	0	0
Massilia varians	0	0
Rugamonas rubra	0	0
unknown_Species_of_Serinibacter	0	0
Brevibacterium iodinum	0	0
unknown_Species_of_Curtobacterium	0	0
ambiguous_Species_of_Herbiconiux	0	0
unknown_Species_of_Labedella	0	0
Labedella sp.	0	0
ambiguous_Species_of_Leifsonia	0	0
Microbacterium imperiale	0	0
Microbacterium luticocti	0	0
Microbacterium oleivorans	0	0
Microbacterium radiodurans	0	0
ambiguous_Species_of_Mycetocola	0	0
ambiguous_Species_of_Plantibacter	0	0
Salinibacterium xinjiangense	0	0
unknown_Species_of_Catelliglobosispora	0	0
Mycobacterium smegmatis	0	0
Mycobacterium sp.	0	0

Mycobacterium vaccae	0	0
Nocardioides albertanoniae	0	0
unknown_Species_of_Amycolatopsis	0	0
ambiguous_Species_of_Amycolatopsis	0	0
Pseudonocardia adelaidensis	0	0
Pseudonocardia eucalypti	0	0
Pseudonocardia parietis	0	0
Streptomyces aurantiacus	0	0
Dyadobacter alkalitolerans	0	0
ambiguous_Species_of_Elizabethkingia	0	0
Epilithonimonas sp.	0	0
Flavobacterium caeni	0	0
Flavobacterium hauense	0	0
Mucilaginibacter litoreus	0	0
Mucilaginibacter rigui	0	0
unknown_Species_of_Olivibacter	0	0
ambiguous_Species_of_Parapedobacter	0	0
Pedobacter cryoconitis	0	0
Pedobacter heparinus	0	0
Pedobacter himalayensis	0	0
Pedobacter koreensis	0	0
Pedobacter kwangyangensis	0	0
Pedobacter piscium	0	0
Pedobacter westerhofensis	0	0
Bacillus murimartini	0	0
Bacillus plakortidis	0	0
Lysinibacillus massiliensis	0	0
Oceanobacillus pacificus	0	0
unknown_Species_of_Ureibacillus	0	0
Weissella viridescens	0	0
unknown_Genus_of_Planctomycetaceae	0	0
Methylopila henanense	0	0
Phyllobacterium catacumbae	0	0
unknown_Species_of_Agrobacterium	0	0
unknown_Species_of_Neorhizobium	0	0
Neorhizobium huautlense	0	0
Rhizobium cellulosilyticum	0	0
Roseomonas cervicalis	0	0
Novosphingobium resinovorum	0	0
Sphingomonas wittichii	0	11
Achromobacter xylosoxidans	0	0
Azohydromonas lata	0	0
Pigmentiphaga litoralis	0	0
Acidovorax wohlfahrtii	0	16
ambiguous_Species_of_Caldimonas	0	0
Massilia yuzhufengensis	0	0

unknown_Species_of_Oxalicibacterium	0	0
unknown_Species_of_CandidatusNitrotoga	0	0
unknown_Genus_of_Nannocystaceae	0	0
Arsenophonus endosymbiont	0	0
Enterobacter pulveris	0	0
ambiguous_Species_of_Gibbsiella	0	0
unknown_Species_of_Plesiomonas	0	0
Legionella massiliensis	0	0
Amphritea sp.	0	0
unknown_Species_of_Enhydrobacter	0	0
Pseudomonas extremorientalis	0	0
unknown_Species_of_Dyella	0	0
Pseudoxanthomonas mexicana	0	0
Microbacterium arborescens	0	17
Aureimonas ureilytica	0	27
Methylobacterium haplocladii	0	12
Rhizobium larrymoorei	0	17
Sphingomonas fennica	0	10
Sphingomonas parapaucimobilis	0	221
Sphingomonas phyllosphaerae	0	12
Sphingomonas sanguinis	0	34
unknown_Species_of_Lautropia	0	10
Acidovorax delafieldii	0	12
ambiguous_Species_of_Polaromonas	0	49
Polaromonas aquatica	0	20
Oxalicibacterium solurbis	0	32
Pantoea septica	0	11
Acinetobacter baylyi	0	10
Leucobacter aerolatus	0	0
Microbacterium binotii	0	0
Arthrobacter psychrolactophilus	0	0
Gelidibacter gilvus	0	0
unknown_Species_of_Planococcus	0	0
Sporosarcina soli	0	0
unknown_Species_of_Cucumibacter	0	0
unknown_Species_of_Palleronia	0	0
Herminiimonas arsenicoxydans	0	0
Arthrobacter citreus	0	0
Promicromonospora aerolata	0	0
Bacillus psychrosaccharolyticus	0	0
Enterobacter asburiae	0	0
unknown_Genus_of_Cryptosporangiaceae	0	0
ambiguous_Species_of_Agreia	0	0
Saccharomonospora sp.	0	0
unknown_Genus_of_Prochlorococcaceae	0	0
Amaricoccus macauensis	0	0

Rubellimicrobium aerolatum	0	0
Acidovorax radicis	0	0
unknown_Genus_of_Desulfurellaceae	0	0
Cystobacter ferrugineus	0	0
ambiguous_Genus_of_Polyangiaceae	0	0
ambiguous_Species_of_Xanthomonas	0	0
Janibacter anophelis	0	0
Microbacterium lacus	0	0
Tessaracoccus flavescens	0	0
unknown_Genus_of_Sanguibacteraceae	0	0
Sanguibacter antarcticus	0	0
unknown_Species_of_Sporichthya	0	0
ambiguous_Species_of_Sporichthya	0	0
Flavobacterium noncentrifugens	0	0
unknown_Species_of_Haloanella	0	0
unknown_Genus_of_Parachlamydiaceae	0	0
unknown_Genus_of_Chlorobiaceae	0	0
ambiguous_Genus_of_ClostridialesFamilyXIX.IncertaeSedis	0	0
unknown_Species_of_Fusobacterium	0	0
Phenylobacterium haematophilum	0	0
ambiguous_Species_of_Bartonella	0	0
ambiguous_Species_of_Beijerinckia	0	0
unknown_Species_of_Bosea	0	0
Aminobacter sp.	0	0
unknown_Species_of_Phyllobacterium	0	0
Agrobacterium vitis	0	0
ambiguous_Species_of_Vasilyevaea	0	0
Erythrobacter litoralis	0	0
Novosphingobium hassiacum	0	0
Bilophila wadsworthia	0	0
unknown_Species_of_Cystobacter	0	0
Pseudomonas tuomuerensis	0	0
Lysobacter spongiicola	0	0
unknown_Species_of_Luteolibacter	0	0
unknown_Genus_of_Brevibacteriaceae	0	0
Microbacterium arthrosphaerae	0	0
Microbacterium halotolerans	0	0
Isoptericola sp.	0	0
Parapedobacter luteus	0	0
Parapedobacter soli	0	0
Sphingopyxis marina	0	0
ambiguous_Species_of_Thiobacillus	0	0
ambiguous_Species_of_Pseudoalteromonas	0	0
Pseudoalteromonas agarivorans	0	0
Marinomonas arctica	0	0
Clostridium chartatabidum	0	0

unknown_Genus_of_Jiangellaceae	0	0
Jiangella alba	0	0
Pseudokineococcus lusitanus	0	0
Nocardiopsis alkaliphila	0	0
Lentzea albida	0	0
Pseudonocardia rhizophila	0	0
Streptomyces yanglinensis	0	0
Petrimonas sulfuriphila	0	0
Hymenobacter norwichensis	0	0
Tumebacillus flagellatus	0	0
ambiguous_Genus_of_Bacillalesincertaesedis	0	0
unknown_Species_of_Planifilum	0	0
ambiguous_Species_of_Planifilum	0	0
Planifilum fimeticola	0	0
Planifilum sp.	0	0
Planifilum yunnanense	0	0
Streptococcus intermedius	0	0
unknown_Species_of_Pirellula	0	0
Mycoplana ramosa	0	0
ambiguous_Genus_of_Cohaesibacteraceae	0	0
Rhizobium subbaraonis	0	0
Roseomonas ludipueritiae	0	0
Altererythrobacter xinjiangensis	0	0
Croceicoccus marinus	0	0
Erythrobacter seohaensis	0	0
unknown_Species_of_Sandaracinobacter	0	0
Sphingomonas mali	0	0
Eoetvoesia caeni	0	0
unknown_Species_of_Brachymonas	0	0
Comamonas odontotermitis	0	0
Hydrogenophaga palleronii	0	0
ambiguous_Species_of_Pelomonas	0	0
Massilia alkalitolerans	0	0
Massilia niastensis	0	0
Thiobacillus thioparus	0	0
Azovibrio restrictus	0	0
ambiguous_Genus_of_Cystobacteraceae	0	0
Archangium gephyra	0	0
Methylobacter luteus	0	0
Georgenia daeguensis	0	0
unknown_Species_of_Demetria	0	0
Frondihabitans cladoniiphilus	0	0
unknown_Species_of_Salinibacterium	0	0
Arthrobacter psychrochitiniphilus	0	0
Citricoccus sp.	0	0
Citricoccus zhacaiensis	0	0

unknown_Species_of_Kocuria	0	0
Kocuria atrinae	0	0
Streptomyces thermoatroviridis	0	0
Flavobacterium antarcticum	0	0
Gelidibacter algens	0	0
Arcticibacter svalbardensis	0	0
unknown_Species_of_Planomicrobium	0	0
Enterococcus alcedinis	0	0
Clostridium sardiniense	0	0
Bosea genosp.	0	0
Mycoplana dimorpha	0	0
Jannaschia donghaensis	0	0
Jannaschia pohangensis	0	0
Roseovarius nanhaiticus	0	0
Sphingobium subterraneum	0	0
Simplicispira psychrophila	0	0
Variovorax ginsengisoli	0	0
Thauera chlorobenzoica	0	0
unknown_Species_of_Escherichia	0	0
Cellulomonas oligotrophica	0	0
unknown_Species_of_Oerskovia	0	0
Leifsonia lichenia	0	0
unknown_Species_of_Ruania	0	0
unknown_Species_of_Algibacter	0	0
unknown_Genus_of_Anaerolineaceae	0	0
unknown_Genus_of_Thermosporotrichaceae	0	0
unknown_Genus_of_Trueperaceae	0	0
unknown_Genus_of_Pasteuriaceae	0	0
unknown_Genus_of_ClostridialesFamilyXVII.IncertaeSedis	0	0
unknown_Genus_of_Heliobacteriaceae	0	0
unknown_Genus_of_Thermoanaerobacteraceae	0	0
unknown_Genus_of_Phycisphaeraceae	0	0
Brevundimonas abyssalis	0	0
unknown_Genus_of_Bartonellaceae	0	0
unknown_Genus_of_Brucellaceae	0	0
Devosia ginsengisoli	0	0
Hyphomicrobium zavarzinii	0	0
Mesorhizobium ciceri	0	0
unknown_Species_of_Nitratireductor	0	0
Kaistia granuli	0	0
Rhizobium etli	0	0
ambiguous_Species_of_Shinella	0	0
ambiguous_Species_of_Sinorhizobium	0	0
Parvibaculum lavamentivorans	0	0
Rhizobium halophytocola	0	0
Ketogulonicigenium vulgare	0	0

Paracoccus denitrificans	0	0
unknown_Genus_of_Sneathiellaceae	0	0
ambiguous_Species_of_Altererythrobacter	0	0
ambiguous_Species_of_Castellaniella	0	0
Castellaniella caeni	0	0
Brachymonas denitrificans	0	0
Comamonas denitrificans	0	0
unknown_Genus_of_Methylophilaceae	0	0
unknown_Species_of_Bacteriovorax	0	0
Bacteriovorax stolpii	0	0
unknown_Genus_of_Desulfohalobiaceae	0	0
ambiguous_Species_of_Cystobacter	0	0
Melittangium lichenicola	0	0
Nannocystis exedens	0	0
unknown_Genus_of_Granulosicoccaceae	0	0
ambiguous_Genus_of_Halothiobacillaceae	0	0
Legionella sp.	0	0
unknown_Species_of_Kangiella	0	0
Pseudomonas alcaligenes	0	0
unknown_Genus_of_Salinisphaeraceae	0	0
Dokdonella sp.	0	0
unknown_Species_of_Acholeplasma	0	0
Acholeplasma granularum	0	0
Acholeplasma sp.	0	0
Opitutus sp.	0	0
Arthrobacter sulfureus	0	0
Streptomyces nanshensis	0	0
Formosa sp.	0	0
Kinneretia asaccharophila	0	0
ambiguous_Species_of_Bdellovibrio	0	0
Bdellovibrio sp.	0	0
unknown_Species_of_Psychrobacter	0	0
Streptomyces ipomoeae	0	0
ambiguous_Species_of_Williamsia	0	0
Wautersiella falsenii	0	0
Shimazuella sp.	0	0
unknown_Species_of_Abiotrophia	0	0
Ochrobactrum gallinifaecis	0	0
ambiguous_Species_of_Paenochrobactrum	0	0
Pseudochrobactrum kiredjianiae	0	0
unknown_Species_of_Donghicola	0	0
unknown_Species_of_Alcaligenes	0	0
ambiguous_Species_of_Bordetella	0	0
Paenalcaligenes sp.	0	0
ambiguous_Genus_of_Aeromonadaceae	0	0
unknown_Species_of_Zobellella	0	0

Raoultella terrigena	0	0
Methylophaga frappieri	0	0
ambiguous_Species_of_Ignatzschineria	0	0
Ignatzschineria sp.	0	0
Salinibacterium sp.	0	0
unknown_Species_of_Micropruina	0	0
Clostridium sartagoforme	0	0
Clostridium litorale	0	0
Microvirga guangxiensis	0	0
ambiguous_Genus_of_Rhodospirillaceae	0	0
Sulfurimonas denitrificans	0	0
Escherichia coli	0	0
unknown_Species_of_Edaphobacter	0	0
Edaphobacter modestum	0	0
Edaphobacter modestus	0	0
Nocardia sp.	0	0
Streptomyces albiflaviniger	0	0
Pedobacter borealis	0	0
ambiguous_Species_of_Hydrogenophaga	0	0
ambiguous_Genus_of_Nitrosomonadaceae	0	0
Flavobacterium johnsoniae	0	0
Caulobacter mirabilis	0	0
Corynebacterium accolens	0	0
Kineococcus aurantiacus	0	0
Flavobacterium aquidurense	0	0
unknown_Species_of_Flavisolibacter	0	0
unknown_Species_of_Phormidium	0	0
Carnobacterium inhibens	0	0
Methylobacterium variabile	0	0
Rubellimicrobium roseum	0	0
unknown_Genus_of_Anaplasmataceae	0	0
Tepidimonas fonticaldi	0	0
Aeromicrobium flavum	0	0
Amycolatopsis sp.	0	0
Thermocrispum municipale	0	0
Hymenobacter xinjiangensis	0	0
Sphingobacterium faecium	0	0
ambiguous_Species_of_Brevibacillus	0	0
unknown_Species_of_Polycladomyces	0	0
Polycladomyces abyssicola	0	0
Agaricicola taiwanensis	0	0
Roseomonas riguiloci	0	0
unknown_Species_of_Kaistobacter	0	0
unknown_Species_of_Uliginosibacterium	0	0
ambiguous_Species_of_Kluyvera	0	0
Olivibacter oleidegradans	0	0

unknown_Species_of_Oxalobacter	0	0
Enterobacter ludwigii	0	0
Corynebacterium xerosis	0	0
Prevotella melaninogenica	0	0
unknown_Species_of_Pleomorphomonas	0	0
Phyllobacterium myrsinacearum	0	0
unknown_Species_of_Kaistia	0	0
Pseudorhodobacter aquimaris	0	0
ambiguous_Species_of_Phaeospirillum	0	0
Pusillimonas ginsengisoli	0	0
Nitrosomonas sp.	0	0
unknown_Species_of_Solimonas	0	0
Leucobacter komagatae	0	0
unknown_Species_of_Pseudoclavibacter	0	0
Arthrobacter gangotriensis	0	0
Arthrobacter rhombi	0	0
unknown_Species_of_Micromonospora	0	0
Micromonospora sp.	0	0
Pseudonocardia tropica	0	0
Streptomyces haliclonae	0	0
Streptomyces marinus	0	0
ambiguous_Species_of_Algoriphagus	0	0
Algoriphagus hitonicola	0	0
unknown_Species_of_Bergeyella	0	0
Planomicrobium alkanoclasticum	0	0
unknown_Species_of_Martelella	0	0
unknown_Species_of_Bartonella	0	0
Campylobacter gracilis	0	0
Eschericia/Shigella coli/dysenteriae	0	0
ambiguous_Species_of_Rickettsiella	0	0
Pseudomonas denitrificans	0	0
Luteimonas huabeiensis	0	0
unknown_Genus_of_Alkalimonas	0	0
Corynebacterium ulceribovis	0	0
ambiguous_Species_of_Citricoccus	0	0
unknown_Species_of_Marmoricola	0	0
ambiguous_Species_of_Saccharomonospora	0	0
Bizionia sp.	0	0
Flavobacterium frigoris	0	0
ambiguous_Species_of_Bhargavaea	0	0
Bhargavaea beijingensis	0	0
unknown_Species_of_Brevibacillus	0	0
ambiguous_Species_of_Salinicoccus	0	0
ambiguous_Species_of_Bradyrhizobium	0	0
unknown_Species_of_Mycoplana	0	0
Methylobacterium hispanicum	0	0

unknown_Species_of_Thioclava	0	0
unknown_Species_of_Methylotenera	0	0
ambiguous_Genus_of_Sulfuricellaceae	0	0
Aeromonas sharmana	0	0
Enterobacter oryzae	0	0
Rickettsiella symbiont	0	0
Methylomicrobium buryatense	0	0
Nicoletella semolina	0	0
Varibaculum cambriense	0	0
Corynebacterium lipophiloflavum	0	0
Blastococcus jejuensis	0	0
Modestobacter marinus	0	0
Marinactinospora thermotolerans	0	0
unknown_Species_of_Spinactinospora	0	0
Prevotella intermedia	0	0
Paenibacillus barengoltzii	0	0
Belnapia moabensis	0	0
ambiguous_Species_of_Ralstonia	0	0
Aeromonas hydrophila	0	0
Aeromonas media	0	0
Arthrobacter agilis	0	0
Streptomyces anulatus	0	0
Luteibacter anthropi	0	0

CS_273	CS_280	CS_282	CS_283	CS_288	CS_302 Cervical Star	CS_307
0	339	53	0	0	0	13
0	37	54	68	0	0	0
0	106	446	362	35	0	175
0	136	216	333	67	24	22
0	15	44	288	46	0	34
0	0	0	11	119	26	14
0	41	83	84	13	0	12
0	16	54	72	0	0	13
24	0	122	215	0	54	129
53	30	252	679	45	70	585
32	0	227	119	0	0	346
0	0	40	41	0	0	0
0	49	12	247	0	0	46
0	0	0	10	0	0	25
0	0	76	60	0	0	107
0	15	20	50	0	0	139
47	466	297	1793	70	685	609
0	0	0	0	0	0	503
0	75	17	107	0	41	0
0	437	117	76	0	12	52
0	458	0	417	0	0	0
0	100	29	22	0	0	14
0	130	11	37	23	0	16
0	0	0	0	0	0	0
390	141	508	1192	87	90	1952
92	50	213	195	0	103	643
25	127	158	242	0	61	200
61	26	80	156	0	41	175
140	91	211	265	0	108	509
16	21	47	108	0	0	146
25	14	11	90	0	0	336
17	0	0	89	0	0	48
418	579	648	1682	0	423	762
0	40	13	0	13	0	0
0	531	0	377	0	0	0
0	442	0	152	0	0	0
0	444	373	57	0	0	0
57	107	60	457	68	137	455
12	0	444	55	0	48	2203
701	1088	2231	4203	135	763	33318
0	166	28	177	0	77	131

0	149	12	197	0	58	147
0	188	0	66	0	32	227
0	85	12	120	0	85	63
759	0	1317	3205	78	404	22702
0	17	0	27	11	0	219
15	21	217	422	0	0	464
11	0	132	261	0	0	401
51	0	821	812	0	0	275
11	0	175	43	0	0	293
50	0	475	289	0	0	58
0	0	0	0	0	0	0
0	0	160	93	0	0	372
98	0	1169	681	0	30	953
0	0	268	132	0	0	19
0	0	214	72	0	0	50
42	0	893	1100	0	12	74
0	0	89	39	0	0	675
185	451	1785	1515	25	281	5876
23	0	352	193	0	12	2875
0	37	143	154	0	32	10
0	0	99	40	0	0	759
0	0	57	44	0	0	28
0	0	0	0	0	0	0
0	0	68	40	0	0	57
0	0	0	0	0	0	112
11	0	139	124	0	0	341
0	0	154	70	0	0	470
10	0	67	48	0	0	20
0	0	10	12	0	0	106
0	0	33	116	0	10	38
136	0	1911	2659	0	52	3146
292	134	3054	5174	377	332	4063
13	0	31	76	16	28	32
0	0	23	77	0	0	163
0	0	201	274	0	0	53
0	0	280	403	0	0	153
0	0	78	126	0	0	167
0	0	220	146	0	10	174
0	0	21	25	0	0	67
14	0	103	78	0	0	437
89	0	1088	811	0	0	4477
0	0	53	83	75	24	0
0	0	24	59	0	0	71
0	0	10	25	0	0	74
96	0	1285	1228	0	92	1143
586	39	5461	7179	119	286	11688

0	0	83	77	0	0	454
28	0	174	110	280	130	659
12	0	611	992	0	0	197
0	0	172	131	0	0	791
492	10	4265	9091	65	155	3221
59	0	35	166	20	10	0
0	10	24	0	0	0	0
0	18	101	95	0	0	0
0	0	33	64	0	0	0
0	28	52	10	0	12	0
0	0	52	98	38	0	21
0	0	65	59	0	32	49
1296	1656	2359	6913	731	1874	3009
0	0	0	13	0	10	0
307	63	851	2064	172	395	527
0	0	93	161	0	0	247
0	0	0	38	0	0	0
52	0	565	460	0	29	262
22	0	10	214	0	24	0
0	0	57	105	0	0	129
0	0	0	23	0	0	11
0	0	26	30	0	0	39
0	0	0	30	0	0	0
45	129	86	240	0	146	43
159	290	417	567	0	257	105
92	106	160	400	10	208	113
33	64	48	224	0	50	53
0	12	0	0	0	0	121
20	165	223	248	0	0	156
390	217	1111	1975	61	446	653
0	104	0	42	0	0	78
12	0	32	26	0	30	0
0	0	0	47	0	0	27
0	0	40	117	0	37	43
0	0	83	65	0	0	35
0	0	0	61	0	0	0
60	26	79	176	0	29	0
74	41	42	297	0	118	12
0	0	53	132	0	21	102
155	178	152	917	21	321	272
11	0	10	89	0	31	26
0	0	12	80	0	10	0
34	0	338	368	0	116	486
695	336	1866	3408	412	640	5780
12	0	23	22	0	27	219
242	211	90	244	0	472	1359

0	23	0	256	0	0	0
44	12	0	310	14	0	32
267	21	29	477	38	62	151
638	13	1572	1662	35	743	9011
13	0	77	51	0	29	601
62	0	63	135	18	395	1029
0	0	0	0	0	0	0
0	0	62	28	0	240	509
0	0	56	44	0	0	68
0	0	0	0	0	0	0
26	0	275	129	0	58	237
10	456	28	298	43	0	24
0	0	205	99	0	0	320
82	25	0	87	0	11	25
214	122	0	358	0	30	121
0	0	183	81	0	0	163
0	0	52	127	0	0	333
0	0	35	76	0	0	0
0	0	0	0	0	0	0
15	46	148	25	0	338	10
69	179	101	313	0	142	64
0	25	28	28	0	140	0
75	10	275	792	53	83	55
0	0	0	53	11	0	11
0	0	55	338	0	12	12
46	138	1057	1084	0	30	392
0	12	72	74	52	44	61
63	304	175	575	112	212	317
16	0	23	182	0	0	45
0	18	79	155	0	23	148
0	0	14	20	40	0	27
0	47	255	226	0	0	53
23	46	224	401	90	98	183
0	0	10	211	0	0	0
0	44	60	84	13	69	89
64	312	1470	1620	0	14	640
41	0	645	539	13	30	595
0	0	87	84	0	0	0
0	0	254	227	0	0	0
0	0	11	23	0	0	0
263	31	12	63	0	870	0
0	0	47	29	0	0	27
0	0	615	379	0	0	69
0	0	37	28	0	0	28
83	440	1967	1529	177	803	13
76	95	2793	68	132	74	15

135	118	5024	54	0	97	14
805	20	366	205	0	704	46
1389	195	15557	362	335	1546	365
389	896	733	268	1146	3166	106
0	0	43	104	0	0	51
77	81	985	422	34	73	268
161	109	422	751	132	121	302
0	22	0	47	0	0	0
0	0	0	36	0	0	91
0	0	40	29	0	0	0
0	0	63	81	0	0	141
0	0	54	32	0	0	10
0	0	252	120	0	0	0
131	145	613	431	283	207	544
0	0	0	22	0	0	21
273	0	2067	3096	23	42	309
0	0	0	12	0	0	12
0	0	0	11	0	0	0
0	0	0	12	0	0	0
0	177	26	11	0	0	0
0	20	50	245	0	0	0
381	922	0	317	0	221	364
0	256	586	77	0	0	1808
0	0	73	0	0	0	0
0	15	126	163	0	0	0
0	94	237	40	0	0	1309
34	2120	415	226	0	42	557
0	0	42	105	0	0	0
0	0	115	105	0	10	23
0	0	0	0	0	0	0
0	0	0	56	0	0	0
0	0	218	623	0	0	0
0	0	73	43	0	0	195
0	0	0	0	0	0	0
0	418	0	0	0	0	0
10	73	0	77	0	68	12
2016	11415	1840	7064	43	1999	3115
1198	2065	1225	2965	0	2051	2183
11	254	0	485	0	94	231
0	43	0	76	0	0	0
0	0	0	0	0	0	13
91	23	3977	3508	0	133	9558
58	66	443	394	12	252	438
23	37	0	223	0	45	14
0	0	149	132	0	0	135
110	0	1054	2142	0	55	1279

16	0	592	722	0	25	1540
267	0	2495	2359	0	0	2744
0	443	21	401	0	27	113
0	87	30	133	0	0	0
26	52	139	204	0	116	33
1054	846	1137	2589	0	1186	1258
41	0	184	1368	0	299	851
104	28	0	30	0	106	97
0	0	0	18	0	0	0
0	0	0	0	0	0	0
10	0	89	528	0	0	724
0	0	0	0	0	0	0
0	0	0	36	0	0	15
204	313	1151	891	0	381	456
0	0	0	39	0	35	0
0	0	0	112	0	0	0
0	0	29	212	0	0	0
0	0	139	393	0	0	434
96	104	72	217	0	171	469
0	82	0	78	0	0	0
0	23	41	215	0	0	0
0	173	0	351	0	0	0
95	167	43	66	0	207	15
0	1427	0	331	0	0	0
455	3678	344	1342	0	760	198
37	102	435	440	0	103	1082
0	0	42	30	0	0	67
188	24	2248	3240	0	213	3074
109	125	241	479	0	607	114
10	10	127	124	0	133	14
11	0	402	529	0	0	1288
49	11	20	368	0	68	17
0	0	35	27	0	0	16
11	11	175	101	0	24	35
66	144	665	420	0	282	661
0	0	192	106	0	60	85
36	0	294	97	0	115	153
0	0	60	39	0	35	0
0	0	58	0	0	0	0
29	63	507	768	0	94	215
55	221	1635	1039	0	32	105
14	14	578	369	30	29	513
0	0	59	42	0	0	107
720	129	1908	214	26	415	960
0	0	0	0	0	0	0
460	2611	1155	699	14	73	630

1567	173	385	1464	0	848	1788
0	0	11	66	0	0	12
53	30	13	172	0	38	0
129	0	0	154	0	42	1236
246	10	359	110	0	54	42
68	0	0	90	0	48	21
109	46	29	67	0	164	0
0	24	0	92	0	24	95
0	0	0	73	0	0	0
0	0	0	10	0	0	0
0	0	0	34	0	0	0
10	197	0	93	0	68	47
2349	404	545	1461	142	1845	8660
55	0	36	144	0	386	77
12	91	0	77	0	165	23
93	132	11	102	15	245	188
517	402	93	667	68	877	337
0	0	49	17	0	44	2104
0	0	0	12	0	46	701
0	0	67	103	0	113	2411
0	0	26	0	0	0	1753
16	46	0	75	0	10	220
82	30	31	282	0	55	947
11	11	0	56	0	0	412
0	12	0	20	0	28	410
107	0	31	261	0	33	102
438	661	478	3015	113	2028	3668
13	25	50	129	23	92	439
10	11	29	84	0	50	103
13	0	11	161	0	138	20
414	268	1602	2406	45	1711	1016
53	28	37	274	0	38	369
29	62	13	83	0	34	743
0	0	0	10	0	89	0
33	44	114	274	0	142	140
146	0	59	282	0	217	734
0	0	0	47	0	0	129
145	203	60	221	10	206	466
142	12	109	295	0	193	1132
142	138	129	400	0	46	558
137	99	43	824	0	70	305
134	87	0	876	0	75	499
0	0	0	0	0	0	200
379	100	13	96	0	441	224
221	0	177	132	0	158	664
40	17	47	47	0	40	119

256	2152	1248	819	14	207	375
277	11	194	376	11	29	399
1107	46	0	303	0	119	44
497	0	193	371	14	48	344
0	0	57	25	11	14	48
0	0	61	43	0	0	13
55	48	48	254	0	0	194
62	0	0	49	11	26	37
0	99	10	14	0	0	0
0	69	17	42	0	0	14
0	0	0	14	0	0	0
504	184	198	336	0	0	317
0	36	0	38	0	0	0
55	28	0	251	0	0	0
0	89	47	11	0	0	0
0	0	28	28	0	0	0
0	49	0	17	0	0	0
0	132	142	330	0	0	0
441	172	1519	2698	45	482	1478
0	343	33	147	0	0	0
0	0	31	22	0	0	0
0	295	70	195	0	0	0
0	0	16	0	0	0	0
293	216	1316	2510	0	677	1114
0	0	0	12	0	0	0
0	0	102	247	0	0	47
0	24	37	123	0	0	243
41	13	59	25	0	42	257
0	0	41	33	0	0	0
10	0	50	152	0	24	56
0	0	24	58	0	0	0
0	79	43	55	0	0	0
0	0	0	42	0	0	36
12	0	66	167	0	0	40
25	24	118	1051	0	14	624
0	0	32	46	0	0	27
0	0	36	0	0	0	16
0	0	0	18	0	0	0
0	0	35	49	0	0	10
10	0	57	62	0	30	17
0	0	22	0	0	0	0
0	0	158	179	0	0	128
38	0	42	126	32	19	84
36	0	147	125	0	15	63
0	0	26	10	0	0	0
0	0	0	0	0	0	0

16	230	177	229	0	0	101
0	0	11	27	0	0	0
11	20	0	0	0	0	12
721	183	3223	1991	197	1007	546
0	0	188	195	0	0	25
0	0	222	274	0	0	14
0	266	189	108	0	0	44
0	262	295	273	0	0	89
0	0	13	21	0	0	15
13	130	80	45	0	65	107
66	473	63	471	0	150	222
186	114	693	1125	148	150	310
65	932	1305	1273	0	155	363
267	55	2362	1587	42	554	367
0	32	188	199	0	0	85
0	0	24	26	0	0	0
0	0	0	10	0	0	0
0	16	553	219	0	22	0
0	0	0	10	0	0	0
0	16	41	24	0	11	0
52	29	190	268	0	212	203
0	0	0	11	0	0	0
12	165	202	289	0	46	101
232	340	1754	1833	0	1023	282
0	0	24	22	0	10	0
0	0	41	22	0	22	0
22	348	31	259	0	27	0
0	0	0	0	0	0	0
208	20	1645	1360	24	146	441
0	0	150	79	0	0	13
0	0	27	21	0	0	12
0	12	301	139	0	0	61
29	0	86	196	0	18	50
0	0	36	37	0	0	0
0	0	13	0	0	0	0
0	0	0	0	0	0	0
14	10	11	57	0	22	0
277	971	514	612	40	131	316
0	0	0	25	0	0	0
0	537	62	76	0	0	21
11	722	34	25	0	0	106
74	24	41	36	0	0	13
0	39	100	26	0	0	0
0	49	12	0	0	0	0
0	0	14	14	0	0	0
0	27	50	14	0	0	0

462	82	57	113	0	88	30
0	218	10	12	0	0	30
0	35	0	0	0	0	0
0	100	0	10	0	0	10
0	0	53	44	0	0	0
0	0	192	80	0	0	28
0	103	52	27	0	0	13
10	89	13	47	0	42	23
1267	772	4721	3491	176	1536	1203
130	16	104	82	25	68	41
10	14	100	74	0	67	120
399	428	5658	1543	25	665	442
0	0	12	262	0	0	0
0	0	0	124	0	0	0
0	17	872	78	0	38	21
582	847	3537	1382	0	1136	519
48	37	208	222	0	222	76
0	0	28	21	0	0	0
0	25	60	65	0	0	25
0	36	21	20	0	0	0
0	48	10	11	0	0	12
0	0	0	14	0	0	12
0	0	47	33	0	0	0
67	97	149	407	0	123	105
139	488	2752	2303	39	403	4237
652	385	1490	1856	52	1110	1546
68	16	95	127	10	41	156
47	139	44	38	0	108	47
125	333	165	133	0	160	651
0	51	0	0	0	0	0
0	0	0	0	0	0	0
0	0	64	75	0	0	57
61	0	11	49	0	14	95
63	0	0	36	0	54	67
89	0	72	135	0	150	147
0	749	0	55	0	0	29
65	26	2468	1489	0	0	4451
2664	496	16832	19031	203	733	39529
55	159	434	613	0	0	826
0	0	48	49	0	0	58
486	0	6528	4969	0	66	4356
10	0	54	41	0	0	464
16	0	263	157	10	0	159
55	0	622	426	0	0	258
0	0	33	0	0	0	34
43	97	1291	1071	0	15	490

26	0	901	379	0	0	46
23	0	276	371	0	0	165
0	0	15	0	0	0	40
379	20	3689	2904	0	176	2346
21	0	12	96	0	28	0
44	48	23	108	0	92	142
77	55	95	264	0	168	182
66	20	12	51	0	35	291
14	45	58	106	0	68	18
0	0	25	30	0	0	56
309	151	533	485	0	265	1660
46	0	560	631	0	0	271
0	0	220	232	0	0	15
0	0	131	86	42	32	56
0	1366	15	22	13	53	11
0	0	0	0	0	0	0
0	0	54	0	0	0	0
0	0	84	25	12	0	15
119	70	429	524	23	155	169
0	0	36	102	0	0	0
23	1703	1030	584	0	32	40
0	0	0	0	0	0	10
54	179	1163	989	0	80	1486
27	52	1934	1980	0	0	61
198	520	2025	2393	0	45	3475
0	0	272	308	0	0	0
0	0	24	0	0	0	10
22	48	247	996	0	16	82
0	0	301	302	0	0	0
0	0	166	110	0	0	0
47	0	535	611	0	0	0
0	0	83	59	0	0	0
0	50	232	139	0	0	0
14	0	15	92	0	126	22
0	0	14	0	0	0	0
0	0	99	80	0	0	60
25	0	632	753	0	0	142
10	394	543	380	0	56	365
2711	1109	13776	15083	1609	3908	7149
0	0	939	681	0	0	12
0	0	0	11	0	0	0
0	0	173	75	0	0	0
50	245	256	249	0	62	1227
0	0	53	10	0	0	120
0	0	98	142	0	0	0
0	0	10	23	0	0	0

0	0	28	73	0	0	0
36	29	309	399	0	88	756
0	0	146	196	0	0	60
0	0	41	43	0	0	0
0	0	41	87	0	0	41
0	11	66	146	0	81	34
0	0	68	61	0	0	24
0	0	13	23	0	19	0
0	0	0	0	0	0	0
0	0	78	55	0	0	37
0	55	30	70	0	0	0
10	13	669	536	0	0	1052
1853	2999	6235	5590	104	3991	3669
272	598	454	1005	0	458	413
35	0	1266	1192	0	0	254
0	0	72	41	0	0	0
0	0	73	40	0	52	0
0	0	36	0	0	30	20
46	77	317	152	0	99	56
378	478	855	1867	0	777	615
264	244	1265	1043	0	331	520
68	11	326	296	0	91	122
19	0	384	314	0	24	253
0	0	35	54	0	0	0
18	14	45	0	0	86	0
0	0	0	13	0	0	0
0	0	180	242	0	0	39
122	179	710	790	0	338	516
0	0	27	77	0	0	70
0	0	38	107	0	0	0
0	0	71	274	0	0	22
0	0	0	0	0	0	0
604	366	193	1567	0	136	161
10	0	47	71	0	0	0
0	10	43	120	0	96	0
0	18	36	0	0	0	14
252	155	22	453	0	30	72
264	38	25	166	0	41	21
460	210	28	2795	0	51	193
583	504	71	950	0	123	164
17	21	10	42	0	12	0
0	189	100	90	0	0	0
0	79	126	93	0	0	10
620	1289	1050	1410	0	1144	2384
0	0	11	67	0	0	37
37	92	94	158	0	0	0

0	0	24	15	0	0	14
0	0	119	461	0	0	0
0	0	24	12	0	0	0
66	0	2648	465	0	110	10
119	50	12	154	29	379	219
231	89	148	98	0	353	1012
126	128	39	148	0	345	146
0	0	28	10	0	0	204
124	0	10	82	0	162	615
12	0	35	379	0	0	23
0	0	429	267	0	0	14
16	0	177	179	0	20	231
0	0	17	38	0	10	95
0	0	0	0	0	0	0
0	0	77	21	0	0	0
0	0	11	25	0	0	0
0	0	0	0	0	0	0
0	0	183	213	0	0	212
0	0	20	48	0	0	30
57	0	16	0	0	0	30
375	810	870	170	0	230	219
0	0	41	0	0	0	0
1711	1020	3776	1377	0	1184	1312
0	0	26	0	0	0	0
851	186	2771	1333	75	553	706
0	0	0	17	0	0	0
635	47	554	110	0	119	210
383	169	1086	398	10	236	207
0	0	30	18	0	0	0
0	0	0	42	0	0	0
0	0	17	0	0	0	0
0	13	0	64	0	14	0
0	0	0	39	0	14	0
0	72	112	71	0	0	10
0	413	0	135	0	28	11
0	100	0	16	0	11	0
0	235	0	207	0	49	0
0	27	0	100	0	0	0
0	10	109	246	0	10	13
0	345	79	0	0	0	0
0	6629	36	0	0	0	0
0	0	0	63	0	0	0
12	14623	536	2162	51	103	166
34	254	0	3007	0	15	10
0	2928	0	573	0	0	17
0	14	0	28	0	0	0

35	0	0	83	0	0	0
1044	251	78	1216	23	283	115
144	109	0	236	0	59	0
0	102	11	110	0	0	178
25	171	0	75	0	22	32
12	26	423	67	0	29	39
0	0	12	53	0	26	137
0	250	0	39	0	25	245
3780	474	2607	7703	12	3928	2167
562	401	432	1259	0	6436	681
290	100	183	606	0	3294	238
23	209	471	278	0	0	117
0	0	23	0	0	0	15
0	162	0	16	0	0	36
0	126	0	0	0	0	0
0	162	0	119	0	25	149
13	14	0	22	0	16	0
0	57	0	63	0	25	13
0	27	10	0	0	0	0
63	0	0	175	0	0	0
52	320	233	388	14	151	194
273	276	34	348	0	53	390
0	32	13	0	0	0	18
0	0	0	27	0	0	0
0	0	0	0	0	0	0
374	174	560	701	0	713	77
3243	893	5042	7100	87	4818	4192
1232	572	376	790	10	2578	3465
13293	4216	7431	11079	236	14191	61172
408	341	59	73	0	438	208
371	212	164	49	0	452	785
213	23	73	89	0	172	1389
2973	1372	2920	1650	53	2910	5938
903	73	558	663	36	1965	1708
163	88	38	88	0	335	51
0	0	58	72	0	0	6974
153	17449	609	2280	148	549	1223
138	8925	665	957	86	550	1316
16	13311	204	692	0	264	850
268	2636	757	3854	0	387	1715
0	0	16	133	0	0	0
0	385	0	30	0	0	22
193	17248	1063	2656	109	396	3147
0	3044	109	84	0	43	63
0	0	11	140	0	0	0
191	32436	296	2129	116	413	713

266	1194	38	1413	0	384	184
3094	2876	4597	8155	59	2272	2523
0	0	56	84	0	33	10
1650	336	123	976	10	824	38
89	189	0	234	0	31	134
0	0	24	25	0	0	10
1148	2782	74	2666	10	581	355
48	28	73	79	0	25	62
0	0	69	42	0	0	14
0	436	0	228	0	0	0
50	1156	0	237	0	15	93
33	84	0	363	0	0	58
0	10	14	65	0	0	0
0	0	25	63	0	0	0
173	3760	1715	1492	14	27	123
615	5669	2301	5867	55	1332	657
24	135	471	954	38	10	162
123	305	876	1239	0	243	380
0	1280	297	350	0	20	174
0	10	0	16	0	0	27
30	4278	468	970	0	0	150
0	0	164	90	0	0	73
0	0	96	56	0	0	212
10	0	691	2205	0	10	22
66	338	153	144	0	763	35
0	0	11	69	0	0	0
0	0	0	30	0	0	0
0	0	34	197	0	0	0
0	0	152	1543	0	0	0
0	364	106	114	0	0	56
0	109	35	474	0	26	0
0	114	0	31	0	0	0
0	272	56	239	0	278	0
116	258	388	391	34	1207	150
115	355	499	200	0	66	40
64	16	274	105	0	68	24
50	25	271	230	0	81	529
0	0	86	11	0	0	108
126	30	379	175	51	958	353
47	82	362	1402	0	0	22
21	0	339	134	0	22	76
165	0	8378	5945	10	72	104
0	0	166	1756	0	0	0
172	0	8393	3751	0	87	216
0	0	24	582	0	36	15
144	10	4530	4688	0	60	74

0	0	254	137	0	0	0
13	113	171	153	0	0	0
24	84	1301	645	0	19	112
0	254	35	43	0	0	0
0	367	0	59	0	10	0
0	0	20	131	0	0	0
0	0	0	13	0	0	0
0	0	0	0	0	0	0
0	499	0	51	0	0	0
0	654	357	501	0	0	73
0	10	96	0	0	10	37
10	0	53	116	0	47	0
0	56	0	83	0	78	239
0	0	0	12	0	58	14
0	86	10	44	0	251	131
0	0	0	67	0	18	19
0	0	0	0	0	62	13
0	99	0	100	0	40	353
0	1381	47	184	0	0	0
0	95	11	10	0	0	0
0	11	0	257	0	13	223
0	20	0	0	0	0	37
0	0	11	0	0	0	14
0	0	0	0	0	0	0
29	54	84	82	0	0	72
0	0	0	0	0	0	0
0	0	0	0	0	0	0
0	0	0	61	0	0	44
0	0	10	0	0	0	0
0	360	0	17	0	0	0
0	30	0	38	0	0	0
0	0	0	0	0	0	0
0	0	10	0	0	0	0
0	17	0	0	0	0	23
0	0	10	0	0	11	56
0	0	0	0	0	0	0
0	0	0	0	0	0	0
0	0	0	0	0	0	0
0	0	0	0	0	0	0
0	0	10	10	0	0	0
0	0	0	0	0	0	0
0	0	0	0	0	0	0
0	0	0	0	0	0	0
0	0	0	90	0	0	17
0	0	0	0	0	40	79
0	46	33	0	0	16	0

0	28	0	0	0	0	0
0	0	0	10	0	0	0
0	0	0	0	0	0	0
0	77	0	66	0	0	18
0	0	11	11	0	0	38
0	0	0	0	0	0	0
0	0	14	38	0	61	14
0	0	0	10	0	0	0
0	0	0	0	0	0	0
0	0	0	0	0	15	33
0	92	0	0	0	0	0
15	0	33	23	0	18	0
0	0	0	0	0	0	15
0	0	0	0	0	0	0
0	0	0	10	0	0	0
0	0	0	17	0	0	0
0	0	0	34	0	0	0
0	0	0	34	0	0	0
25	23	0	22	0	11	188
28	0	0	52	0	51	0
0	0	11	11	0	0	207
0	0	39	0	0	10	50
0	14	0	112	0	0	0
29	0	0	104	0	11	15
0	0	0	0	0	0	0
0	40	0	27	0	0	0
0	58	0	0	0	11	0
0	11	0	0	0	0	0
0	0	0	0	0	0	0
0	0	0	0	0	0	0
0	19	0	0	0	0	10
0	0	0	0	0	0	0
0	0	0	69	0	12	0
0	51	50	16	19	0	28
0	0	158	0	0	0	0
0	93	36	20	0	15	0
0	0	91	0	0	0	0
61	0	0	84	41	685	0
0	0	13	0	0	0	0
0	0	0	0	0	0	91
10	666	94	51	46	0	15
0	179	0	12	48	0	0
15	476	50	29	0	11	0
0	11	0	46	0	0	0
24	0	481	0	0	0	28
0	0	0	18	0	0	0

0	86	113	0	0	49	21
0	0	78	0	0	0	0
0	0	0	15	0	0	137
0	22	0	0	0	0	0
142	0	2200	41	10	0	267
249	134	78	82	0	350	237
0	0	13	0	0	0	0
15	99	24	0	0	0	27
0	86	0	0	0	0	0
0	67	0	0	0	0	0
0	25	0	0	0	0	0
0	0	0	36	0	0	0
224	163	0	431	0	28	38
0	0	0	0	0	0	0
0	0	0	0	0	0	0
0	0	0	0	0	0	0
0	0	0	0	0	0	0
0	0	0	24	0	0	0
748	0	29	265	0	0	57
0	0	0	0	0	0	0
0	0	0	0	0	0	0
0	0	0	0	0	0	0
0	0	0	0	0	0	0
0	28	133	44	0	12	99
0	22	120	37	0	34	52
0	1712	0	114	0	150	53
0	0	0	41	0	0	0
0	0	0	83	0	40	0
51	29	0	29	0	89	125
0	0	0	0	0	0	0
0	0	0	76	0	15	0
12	40	0	38	0	91	0
0	0	0	0	0	0	0
53	133	15	82	0	127	596
0	0	141	14	0	0	17
0	0	0	0	0	0	0
0	0	0	0	0	0	0
0	0	10	11	0	18	0
0	30	0	52	0	13	11
0	0	85	13	0	209	0
0	0	0	0	0	12	0
0	0	0	0	0	0	58
0	0	0	10	0	447	0
0	11	10	23	0	0	0
0	0	0	0	0	0	0
146	0	401	25	0	38	234

0	2957	57	537	0	0	92
0	0	0	0	0	0	11
0	16	0	0	0	0	0
0	0	0	11	0	0	0
43	13	0	21	0	81	14
51	0	0	0	0	0	0
0	0	0	0	0	0	27
144	17	0	0	0	73	0
0	0	13	0	0	0	0
0	0	0	0	0	0	0
0	0	34	124	0	34	46
22	0	13	0	0	26	11
404	72	90	138	0	278	84
0	0	0	0	0	29	0
168	0	42	87	0	86	36
0	0	65	38	0	0	0
10	0	0	0	0	12	20
29	0	0	21	0	0	0
21	0	0	38	0	0	89
0	0	0	0	0	0	0
0	0	0	0	0	0	0
0	0	0	19	0	21	0
0	0	47	26	0	0	11
0	0	101	104	0	29	20
0	0	0	11	0	204	0
0	0	0	0	0	114	0
22	137	11	56	0	223	50
0	45	0	11	0	63	23
55	157	27	46	0	153	87
0	0	0	0	0	0	0
0	0	0	0	0	0	410
0	0	11	0	0	37	1031
0	0	0	0	0	0	106
0	0	0	15	0	34	1053
0	0	0	0	0	0	72
0	0	0	0	0	0	26
0	0	0	0	0	0	98
26	17	24	46	0	52	39
71	0	44	21	0	74	29
0	0	0	0	0	0	0
0	0	0	168	0	30	513
38	0	0	70	0	0	0
0	0	0	0	0	0	0
0	0	0	0	0	0	0
0	0	30	55	0	0	0
0	0	0	20	0	0	0

0	0	0	0	0	0	0
11	78	0	252	0	245	82
0	0	0	0	0	0	0
0	17	0	26	0	23	0
0	32	73	10	0	0	0
28	10	0	10	0	23	37
0	0	0	0	0	12	24
0	0	0	0	0	26	0
46	10	75	0	0	195	10
0	92	0	0	0	0	152
0	0	0	48	0	10	37
0	0	124	0	0	0	264
0	0	0	0	0	0	0
0	0	0	0	0	0	0
0	0	0	0	0	0	0
0	0	0	0	0	0	0
46	0	26	45	0	47	74
0	0	0	90	0	0	0
0	191	38	204	0	0	17
0	0	10	0	0	15	0
124	64	14	16	0	176	142
0	0	0	0	0	0	0
0	0	0	0	0	0	0
10	0	0	0	0	0	0
0	0	0	0	0	0	0
144	0	0	49	0	0	34
0	0	0	0	0	0	26
24	13	14	54	0	0	0
0	0	0	0	0	0	0
0	0	0	0	0	0	0
12	0	0	0	0	17	0
27	0	15	0	0	10	0
0	0	0	0	0	0	0
0	0	0	0	0	0	0
0	0	0	0	10	0	10
0	0	0	0	0	0	13
382	0	0	185	0	0	235
760	0	0	315	0	40	190
0	0	0	0	0	0	0
127	0	11	56	0	0	95
0	0	0	47	0	0	11
0	0	38	0	0	0	152
0	0	0	0	0	0	0
0	0	0	0	0	0	0
0	0	0	0	0	0	0
0	0	0	0	0	39	0

0	0	0	0	0	0	0
0	0	0	0	0	0	0
49	0	41	44	0	0	29
0	711	0	0	0	0	0
36	25	0	22	0	0	14
32	0	0	0	0	0	0
0	18	0	0	0	0	0
0	0	0	159	35	10	10
0	0	10	0	0	0	0
36	241	65	62	0	32	0
10	59	11	115	33	0	27
0	365	0	84	0	0	0
0	0	0	10	0	0	0
0	92	0	37	0	0	0
0	0	0	10	0	11	0
0	0	169	25	0	0	0
12	0	26	0	0	89	0
0	0	0	0	0	0	0
0	264	12	27	0	0	0
0	0	64	10	0	10	10
0	0	0	0	0	0	0
0	0	10	0	0	0	10
0	0	13	0	0	0	0
0	0	0	0	0	0	0
0	0	0	0	0	0	0
0	0	0	0	0	0	0
0	40	0	43	0	0	0
0	0	0	0	0	10	12
0	0	0	62	0	12	0
0	0	0	0	0	0	0
0	0	58	12	0	10	21
0	0	13	46	0	20	56
0	0	61	0	0	0	0
0	0	0	0	0	0	0
85	1475	89	343	0	85	30
0	31	0	0	0	0	13
0	0	0	10	0	0	0
0	52	0	16	0	0	0
272	33	0	133	0	31	45
0	0	15	14	0	0	0
0	0	68	33	0	0	0
0	12	0	224	0	0	0
0	93	22	0	0	0	0
0	0	0	17	0	0	0
38	0	0	10	26	55	154
0	0	0	0	0	10	10

0	956	10	0	0	20	0
0	810	0	0	0	0	0
0	0	0	0	0	0	0
0	0	0	0	0	22	0
15	0	28	35	0	0	25
21	0	35	59	0	35	26
0	0	0	0	0	0	0
0	0	64	78	0	0	11
0	0	23	0	0	0	0
0	0	0	0	0	0	0
0	0	0	0	0	0	0
0	10	0	0	0	0	0
0	425	171	21	0	82	16
15	0	112	0	0	29	0
0	75	47	10	0	0	46
11	42	113	46	0	13	0
0	0	123	10	0	10	0
0	0	0	0	0	0	0
0	0	0	26	0	0	0
0	0	0	0	0	0	0
0	0	0	0	0	0	0
0	0	0	0	0	0	0
13	0	0	0	0	13	0
0	747	0	140	0	0	10
0	219	27	33	0	0	0
0	492	20	55	13	37	72
0	32	0	0	0	0	0
12	182	0	63	0	0	0
0	454	0	40	0	0	0
0	0	0	0	0	0	0
0	0	0	0	0	0	0
0	0	0	0	0	0	72
0	0	0	0	0	0	0
0	0	0	0	0	0	0
0	100	0	0	0	0	0
0	105	0	24	0	0	14
0	0	25	27	0	80	0
0	0	91	0	0	0	0
0	0	51	0	0	0	0
0	0	0	0	0	0	0
25	229	52	138	0	141	321
0	653	0	75	0	42	0
10	0	0	83	0	0	0
0	1071	10	57	0	0	16
0	141	0	46	0	0	0
0	0	0	0	0	0	0

0	0	0	0	0	0	0
0	0	0	0	0	0	0
0	43	0	13	0	0	0
0	0	0	0	0	0	0
0	0	0	10	0	0	0
0	0	0	0	0	0	0
0	0	0	0	0	0	0
0	14	0	0	0	27	0
26	14	11	62	0	26	0
0	0	0	0	0	0	0
21	0	0	0	0	0	10
0	0	0	0	0	0	0
0	39	24	141	0	0	19
22	549	14	10	0	0	0
0	214	0	114	0	96	116
210	57	0	52	0	43	0
0	10	0	0	0	0	54
0	403	0	10	0	52	0
0	0	0	0	0	0	0
14	31	0	0	0	0	10
11	36	0	0	0	0	0
41	90	0	67	26	84	151
0	10	11	40	0	0	0
0	0	0	0	0	0	0
0	0	0	0	0	0	0
0	0	0	0	0	0	0
0	0	0	0	0	0	0
44	47	0	32	0	119	953
12	0	0	13	0	0	0
10	0	0	0	0	10	0
81	52	23	101	0	536	68
94	59	79	42	0	142	474
50	100	0	45	0	278	95
0	0	0	0	0	0	0
0	10	0	25	0	0	0
0	0	0	0	0	0	0
0	0	0	0	0	0	0
0	10	0	0	0	21	32
0	1205	0	0	0	0	0
0	0	0	0	0	0	0
0	16	0	0	0	0	0
0	0	114	166	0	0	23
0	0	0	0	0	0	0
0	0	21	43	0	0	0
0	0	0	0	0	0	0
0	0	0	0	0	0	0

0	0	0	47	0	0	0
0	0	0	12	0	0	0
0	0	15	0	0	0	0
0	0	0	0	0	0	0
0	10	198	14	0	25	0
0	0	0	25	0	0	0
0	143	0	0	0	0	0
0	0	0	0	0	0	0
0	89	0	62	0	11	21
104	262	0	10	0	0	0
0	392	10	22	0	0	20
25	74	0	270	0	57	17
0	0	0	0	0	0	0
0	0	0	0	0	0	0
0	1265	24	81	0	10	34
0	0	0	0	0	60	0
0	0	0	0	0	0	0
0	197	0	0	0	0	0
0	0	0	0	0	0	391
0	0	11	15	0	0	0
0	0	39	0	0	26	439
0	114	0	20	0	0	0
0	0	0	0	0	0	32
0	0	0	0	0	0	0
0	0	21	13	0	23	0
0	10	0	0	0	13	0
0	0	0	0	0	0	0
0	35	0	0	0	0	0
0	21	0	0	0	0	0
0	0	12	0	0	0	12
0	0	0	0	0	0	0
0	0	0	30	0	0	0
0	0	182	12	0	30	42
0	0	0	0	0	0	14
0	0	0	24	0	0	0
38	11	64	32	0	0	75
25	0	0	40	0	0	11
0	0	12	53	0	0	94
0	0	0	0	0	0	0
0	0	0	0	0	10	0
0	0	0	0	0	12	0
0	0	0	0	0	10	0
0	0	0	0	0	34	0
0	0	0	0	0	0	0
0	0	0	0	0	0	17
0	44	0	10	0	0	0

0	0	0	0	0	0	0
0	11	0	0	0	0	33
0	72	12	15	0	0	0
251	24	50	26	0	24	40
0	56	0	0	0	0	0
0	12	0	27	0	0	0
0	0	0	0	0	0	0
0	0	0	54	0	11	0
0	0	0	45	13	0	0
0	0	0	0	0	0	0
0	0	0	21	0	0	0
0	0	0	52	0	0	0
0	13	0	23	0	0	0
0	45	49	43	0	0	374
0	0	0	17	0	0	0
0	0	0	0	0	0	0
0	0	0	0	0	0	13
0	0	44	0	0	0	180
0	0	0	0	0	0	0
0	0	11	110	0	0	15
0	0	0	0	0	35	49
0	0	0	31	0	0	14
0	31	0	0	0	0	10
0	12	0	0	0	0	0
0	0	0	0	0	0	0
0	0	0	0	0	0	176
0	0	0	0	0	11	37
0	0	0	0	0	0	37
0	0	0	0	0	0	71
0	10	21	0	0	0	0
0	97	0	10	0	0	0
0	0	0	0	0	0	0
0	0	0	0	0	0	0
0	39	24	0	0	170	0
0	0	0	0	0	0	0
0	112	0	0	0	0	0
0	0	0	0	0	0	0
0	0	0	0	0	0	0
0	0	14	15	0	13	10
0	12	0	24	0	0	0
74	0	844	0	0	0	25
0	0	0	0	0	0	0
0	0	0	0	0	0	0
0	0	0	0	0	0	0
0	0	0	0	0	0	0
0	0	0	0	0	0	42

0	0	0	0	0	0	627
0	0	0	0	0	0	0
0	0	0	0	0	0	0
0	103	0	0	0	0	0
0	759	0	0	0	0	107
0	51	0	0	0	0	0
0	0	0	0	0	0	0
0	0	86	0	0	0	0
0	0	0	0	0	0	0
0	0	0	40	0	10	26
0	0	0	0	0	0	0
0	0	0	0	0	0	42
0	0	0	0	0	0	0
0	0	0	0	0	0	0
0	0	0	0	0	0	0
0	0	0	0	0	0	0
11	0	0	10	0	14	0
0	0	0	0	0	0	0
167	0	0	75	0	0	0
0	0	15	0	0	0	0
0	0	0	0	0	10	0
0	0	0	0	0	0	0
0	0	0	0	0	0	0
0	0	21	0	0	0	0
0	0	0	0	0	0	0
0	0	0	0	0	0	0
0	0	0	0	0	0	0
0	0	0	0	0	0	170
0	0	0	0	0	0	0
0	0	0	0	0	0	118
0	0	0	0	0	0	0
0	0	0	0	0	0	0
0	145	0	21	0	0	0
0	26	10	28	0	0	0
0	0	14	0	0	0	0
33	0	0	26	0	10	39
0	0	0	0	0	0	0
0	0	0	0	0	0	0
0	0	0	0	0	0	0
0	0	0	0	0	0	0
0	0	0	0	0	0	0
0	0	0	0	0	0	0
0	0	0	0	0	0	0
0	0	0	0	0	0	0
0	0	0	0	0	0	0
15	0	0	0	0	138	0

0	0	0	0	0	0	0
0	0	0	0	0	0	11
0	0	0	0	0	0	0
0	0	0	0	0	0	0
0	0	0	0	0	0	0
0	0	0	0	0	0	0
0	0	0	0	0	0	0
0	0	0	0	0	0	0
0	0	0	0	0	0	0
0	0	0	0	0	0	0
0	0	0	0	0	0	0
31	25	0	130	174	1948	0
0	0	0	0	0	29	0
170	0	80	38	0	62	0
24	0	1099	0	0	0	0
0	0	10	0	0	0	0
0	0	0	0	0	0	0
0	0	0	0	0	0	0
0	0	0	0	0	0	0
11	0	0	0	0	0	0
0	0	0	0	0	0	0
0	0	0	0	0	0	0
0	0	0	0	0	0	0
0	0	0	0	0	0	0
0	0	0	0	0	0	0
0	0	0	0	0	0	0
0	0	0	0	0	0	0
0	0	0	0	0	0	0
69	0	0	0	0	0	0
100	0	0	0	0	0	0
0	0	0	0	0	0	0
0	0	0	0	0	0	0
0	0	0	0	0	22	0
0	0	0	0	0	0	0
0	0	0	0	0	11	0
88	0	179	0	0	0	0
0	0	0	0	0	0	0
0	0	0	0	0	0	0
0	0	0	0	0	0	0
0	0	0	0	0	0	0
0	0	0	0	0	0	0
0	0	0	0	0	0	0
0	0	0	0	0	0	0
0	0	0	0	0	0	0
0	0	0	0	0	0	0
0	0	0	0	0	0	0

0	0	0	0	0	0	0
0	0	0	0	0	0	0
0	0	0	0	0	0	0
0	0	0	0	0	0	0
0	0	0	0	0	0	0
0	0	0	0	0	0	0
0	0	0	0	0	0	0
0	0	0	0	0	0	0
37	0	0	47	0	0	12
0	0	0	0	0	0	0
0	0	0	0	0	0	0
0	0	0	0	0	0	0
0	0	0	0	0	0	0
10	0	0	0	0	0	0
0	0	0	0	0	0	0
0	0	0	0	0	0	0
317	0	0	105	0	0	23
0	0	0	0	0	0	0
0	0	0	0	0	0	0
0	0	0	0	0	0	0
0	0	0	0	0	0	0
0	0	0	0	0	0	0
0	0	0	0	0	0	0
0	0	0	0	0	0	0
0	0	0	0	0	0	28
0	0	0	0	0	0	0
0	0	0	0	0	0	0
0	0	0	0	0	0	0
0	0	0	0	0	0	0
60	190	0	0	0	0	0
0	0	0	0	0	0	0
0	0	71	0	0	11	0
0	0	0	0	0	0	0
0	0	0	0	0	0	0
0	0	0	0	0	0	0
0	0	0	0	0	0	0
0	0	0	0	0	0	0
0	0	0	0	0	0	0
0	0	0	0	0	0	0
0	11	27	0	0	0	0
0	0	0	0	0	0	0
0	0	0	0	0	0	0
0	0	0	0	0	0	0
0	0	0	0	0	0	0
0	27	0	0	0	0	22
0	0	0	0	0	0	0

0	0	0	0	0	0	0
0	0	0	0	0	0	0
0	66	0	83	0	0	194
0	16	11	10	0	0	117
0	0	0	0	0	0	0
0	0	0	0	0	0	0
0	0	0	0	0	0	0
0	0	0	0	0	0	0
0	0	10	0	0	0	0
0	0	0	0	0	0	0
0	0	0	0	0	0	0
0	0	0	0	0	0	0
0	0	0	0	0	0	0
0	0	0	10	0	0	0
0	0	0	0	0	0	0
0	0	10	0	0	0	15
0	0	0	0	0	0	0
0	0	0	0	0	0	14
0	0	0	0	0	0	0
0	39	0	0	0	0	0
0	0	0	0	0	0	0
0	0	0	0	0	0	0
0	0	0	0	0	0	64
0	0	0	0	0	0	387
0	0	0	0	0	0	0
0	0	0	33	0	0	0
0	0	28	11	0	0	0
0	0	0	0	0	0	0
0	0	0	0	0	0	0
0	0	27	12	0	0	0
0	25	0	17	23	0	0
0	0	0	0	0	0	0
16	108	10	62	0	0	23
0	0	0	0	0	0	0
0	0	145	0	0	0	0
0	0	0	0	0	0	0
0	0	11	0	0	0	0
0	0	0	0	0	0	0
135	0	0	10	0	0	31
0	0	0	70	0	98	0
0	0	0	0	0	0	0
0	0	0	0	0	0	0
0	0	0	11	0	0	939
0	0	26	0	0	0	0
0	0	0	0	0	0	0
0	0	0	0	0	0	0

0	0	0	0	0	0	0
0	981	19	15	0	0	14
0	20	0	0	0	33	0
0	1873	33	0	21	0	0
0	0	0	0	12	0	0
25	1210	0	0	0	0	0
32	0	0	0	0	0	10
0	0	0	0	0	0	0
0	0	0	0	0	0	0
0	0	0	0	0	0	0
0	0	0	0	0	0	0
0	0	0	0	0	0	0
0	0	0	0	0	0	0
0	0	0	0	0	0	0
0	0	0	0	0	0	0
0	0	0	11	0	0	0
0	0	0	0	0	0	0
0	0	0	0	0	0	0
43	0	0	10	0	0	0
120	0	0	0	0	0	0
0	0	0	0	0	0	0
0	0	0	0	0	0	0
0	0	9664	0	0	0	0
0	0	18764	0	0	0	16
0	0	0	0	0	0	0
0	0	0	0	0	0	12
0	0	0	0	0	0	0
0	0	0	0	0	0	0
0	0	0	0	0	0	0
0	0	0	16	0	0	0
0	0	0	0	0	22	0
0	0	0	0	0	0	0
0	0	0	0	0	0	13
13	0	0	49	0	0	13
10	0	0	10	0	0	0
15	0	0	0	0	0	0
28	0	0	0	0	0	0
32	0	0	35	0	0	38
0	0	0	44	0	0	0
11	0	0	0	0	0	10
0	0	0	0	0	0	0
0	0	0	0	0	0	0
0	0	0	0	0	0	0
0	0	0	0	0	0	0
13	0	0	0	0	0	0
38	0	0	17	0	0	10

0	0	0	0	0	0	0
0	0	0	0	0	0	0
0	27	21	0	0	0	12
0	33	21	24	0	0	40
0	0	0	0	0	0	0
0	0	0	0	0	0	0
0	0	25	24	0	0	19
0	0	23	0	0	0	0
0	0	29	0	0	0	0
0	0	0	0	0	0	0
0	0	10	0	0	0	24
0	0	0	0	0	0	0
0	0	128	0	0	0	0
0	0	0	0	0	0	0
0	0	31	0	0	0	0
0	0	11	0	0	0	0
0	0	0	0	0	0	0
0	0	0	0	0	0	0
0	78	0	0	0	0	0
0	50	0	0	0	0	0
0	0	25	0	0	0	0
0	0	0	0	0	0	0
0	0	0	0	0	0	0
0	0	0	0	0	0	0
0	312	27	0	0	0	0
0	0	0	0	0	0	0
0	0	0	0	0	0	0
0	0	0	0	0	0	0
0	14	0	0	0	0	0
0	0	0	18	0	0	0
0	11	0	37	0	0	0
0	0	0	11	0	11	0
0	0	0	0	0	0	0
0	0	19	0	0	0	0
0	12	0	0	0	0	0
0	0	0	0	0	0	0
0	0	66	0	0	0	0
0	0	12	10	0	0	24
0	35	0	0	0	0	0
0	0	0	0	0	0	16
0	0	0	0	0	0	0
0	119	0	0	0	0	0
0	0	0	117	0	0	0
0	0	0	57	0	0	0
0	0	0	14	0	0	0
0	40	0	0	0	0	0

0	0	0	0	0	0	0
0	0	27	0	0	0	24
0	0	0	0	0	0	0
0	0	0	0	0	0	0
0	0	0	0	0	0	0
0	0	27	0	0	0	0
0	0	0	0	0	0	0
0	0	232	0	0	0	0
0	0	0	0	0	0	0
0	0	10	10	0	16	41
0	0	0	0	0	0	0
0	0	0	0	0	0	0
0	0	0	0	0	0	0
0	0	0	0	0	0	0
0	0	0	0	0	0	0
0	0	0	0	0	0	0
0	0	0	0	0	0	10
0	224	407	10	0	0	0
0	0	0	0	0	0	0
0	0	0	0	0	0	0
0	0	0	0	0	0	0
0	40	0	0	0	0	0
0	0	0	0	0	0	0
0	0	0	11	0	687	0
0	0	0	0	0	0	0
0	0	0	0	0	0	0
0	0	0	0	0	0	0
0	0	0	0	0	386	0
0	0	0	29	0	113	0
0	29	0	56	0	1758	0
0	0	23	0	0	0	0
0	0	0	0	0	0	0
0	0	0	0	0	0	0
0	211	21	10	0	38	0
0	45	0	0	0	0	0
0	0	0	0	0	0	0
0	0	0	0	0	0	0
0	0	1074	0	0	0	0
0	0	0	0	0	0	0
0	0	605	0	0	0	0
0	0	0	0	0	0	0
0	0	0	0	0	0	0
0	0	0	0	0	0	0
0	0	0	0	0	0	0
0	0	0	0	16	0	0
0	0	0	0	0	0	0

0	0	13	0	0	0	0
0	0	0	0	0	0	0
0	0	0	0	0	0	0
0	0	0	0	0	0	0
195	35	0	0	0	0	0
268	0	0	148	0	14	0
0	0	0	0	0	0	0
0	0	0	0	0	0	0
0	0	0	0	0	0	0
0	0	0	0	0	0	0
0	0	0	0	0	0	0
0	0	0	0	0	0	0
0	0	0	0	0	0	0
11	0	14	0	0	0	0
0	0	0	0	0	0	0
0	0	0	0	0	0	0
0	0	0	0	0	0	0
0	0	0	0	0	0	0
0	0	0	51	0	0	0
0	0	0	0	0	0	0
0	0	0	0	0	0	0
0	167	0	0	0	0	0
16	0	0	0	0	0	0
0	0	0	0	0	0	278
0	0	0	0	0	0	0
0	0	0	0	0	0	0
0	0	101	0	0	0	0
0	0	969	0	0	0	0
0	0	0	0	0	0	0
0	0	0	0	0	0	0
0	0	13	0	0	0	13
0	0	0	0	0	0	17
0	0	0	0	0	0	0
0	0	0	0	0	0	0
0	0	0	0	0	0	0
0	0	0	0	0	0	0
0	0	0	20	0	0	0
12	0	0	0	0	0	0
0	0	11	0	0	0	0
0	0	0	0	0	0	0
0	0	0	0	0	0	0
0	0	0	0	0	0	0
0	0	0	0	0	0	0
0	0	0	0	0	0	0
0	0	0	0	0	0	0
0	0	12	0	0	0	0

0	0	25	0	0	0	0
0	0	35	0	0	0	0
16	0	0	11	0	0	0
0	0	10	0	0	0	0
0	0	0	0	0	0	0
0	16	10	0	0	0	0
0	0	0	0	0	0	0
0	417	0	0	0	0	23
0	0	0	0	0	0	0
0	0	0	0	0	0	0
0	0	0	0	0	0	0
0	0	0	0	0	0	0
0	0	0	0	0	0	0
0	0	0	0	0	0	0
0	0	0	0	0	23	0
0	48	0	0	0	20	11
0	0	0	10	0	0	0
0	0	0	0	0	0	0
0	200	0	0	0	0	0
26	0	0	23	0	0	0
0	0	0	0	0	0	0
13	0	0	60	0	0	0
0	0	0	0	12	0	0
0	0	0	0	11	22	0
0	85	0	0	0	0	26
0	0	0	0	0	0	0
0	0	0	0	0	0	0
0	0	0	0	0	0	0
0	12	0	0	0	0	0
0	0	0	0	0	0	0
0	172	0	27	0	0	0
0	0	0	0	0	0	0
0	0	0	0	0	0	0
0	0	0	0	0	0	0
0	0	21	12	0	0	0
10	0	0	0	0	0	0
0	0	0	0	0	0	0
0	0	45	10	0	0	0
0	104	0	0	0	0	0
0	0	0	0	0	0	0
0	0	10	0	0	0	0
0	0	0	0	0	0	0
0	0	0	0	0	11	0
0	0	0	0	0	0	0
0	0	0	0	0	0	0
0	0	0	0	0	0	0

0	255	0	0	0	0	0
0	28	0	0	0	0	0
0	0	0	0	0	0	0
0	0	0	0	0	0	0
0	54	0	0	0	0	0
0	0	0	0	0	0	0
0	0	0	0	0	0	0
0	0	0	0	0	0	0
0	0	61	0	0	0	0
0	61	0	69	0	16	0
0	23	0	0	0	0	0
0	0	0	0	0	0	0
0	82	0	0	0	0	0
0	0	0	0	0	0	39
0	0	0	0	0	0	0
0	0	0	0	0	0	11
0	0	0	0	0	0	0
0	77	0	0	0	0	0
0	0	0	0	0	0	0
0	31	0	0	0	0	0
0	0	0	0	0	0	0
0	0	0	0	0	0	0
0	0	0	0	0	0	0
0	0	0	0	0	0	0
0	1993	0	13	0	0	0
0	0	0	0	0	0	11
0	0	0	0	0	0	172
31	0	0	12	0	44	0
0	0	0	0	0	0	0
0	0	0	0	0	0	0
0	0	0	0	0	0	0
0	0	0	0	0	0	0
0	0	0	0	0	0	0
0	0	0	0	0	0	0
0	0	0	0	0	0	0
0	0	0	0	0	0	0
0	0	13	10	0	0	0
0	0	0	0	0	0	38
0	0	0	0	0	0	0
0	12	0	24	0	0	0
0	0	0	0	0	0	10
0	0	0	0	0	0	40
0	0	0	0	0	0	0
0	0	0	0	0	0	0
0	0	0	0	0	0	0
0	0	0	0	0	0	0

0	0	0	0	0	0	25
0	0	0	0	0	0	0
0	0	0	0	0	0	24
0	0	56	0	0	0	0
0	34	0	0	0	0	0
0	118	0	0	0	0	0
0	98	0	0	0	0	0
0	23	0	0	0	0	0
0	0	0	0	0	0	0
0	44	0	0	0	0	0
0	0	0	0	0	0	0
0	13	12	0	0	0	0
46	0	0	129	0	0	0
0	0	0	0	0	0	0
0	0	0	0	0	0	0
0	0	0	0	0	0	0
0	29	0	0	0	0	0
0	23	54	0	0	0	0
0	0	0	0	0	0	11
0	0	0	0	0	0	0
0	0	0	0	0	0	0
0	72	20	0	0	0	11
0	0	0	0	0	0	0
0	0	11	0	0	0	0
0	0	0	0	0	0	0
0	0	0	0	0	0	11
0	0	0	0	0	0	0
0	0	0	0	0	0	0
0	0	0	0	0	0	0
0	0	0	0	0	0	0
0	0	0	0	0	0	0
0	0	0	0	0	0	29
0	0	0	0	0	0	0
0	0	0	0	0	0	0
0	0	0	0	0	0	0
0	0	0	0	0	0	0
0	0	0	0	0	0	0
0	0	0	0	0	0	0
0	0	39	0	0	0	0
0	0	0	0	0	0	0
0	0	0	0	0	0	0
0	0	0	0	0	0	0
0	0	0	0	0	0	0
0	0	0	0	0	0	0
0	0	0	0	0	0	0
0	0	0	0	0	0	12

0	0	0	0	0	0	0
0	0	0	0	0	0	0
0	0	0	0	0	0	0
0	0	0	0	0	0	0
0	0	0	0	0	0	0
0	23	0	0	0	0	0
0	0	0	0	0	0	0
0	0	0	0	0	0	0
0	0	0	0	0	0	0
0	0	0	0	0	0	0
0	0	0	0	0	0	0
0	0	0	0	0	0	0
0	0	0	0	0	0	0
0	0	0	0	0	0	0
0	0	0	0	0	0	0
0	0	0	0	0	0	0
0	0	0	0	0	0	0
0	0	0	0	0	0	0
0	0	0	0	0	0	0
0	0	0	0	0	0	0
0	0	20	0	0	0	0
0	0	47	0	0	14	48
0	0	0	0	0	0	0
0	0	0	0	0	0	0
0	0	0	0	0	0	0
0	0	0	0	0	0	0
0	0	0	0	0	0	0
0	0	0	0	0	0	0
0	0	0	122	0	0	0
0	0	0	0	0	0	0
0	0	0	0	0	0	0
0	0	0	0	0	0	0
0	63	21	0	0	0	0
0	0	0	0	0	0	0
0	0	0	0	0	0	0
0	0	0	0	0	0	28
0	0	0	0	0	0	0
0	0	0	0	0	0	0
0	0	0	0	0	0	0
0	0	0	0	0	0	0
0	0	0	0	0	0	0
0	0	0	0	0	16	0
0	0	0	0	0	0	0
0	0	0	0	0	0	0
0	0	0	0	0	0	0
0	0	0	0	0	0	0

0	0	25	0	0	0	0
0	0	0	0	0	0	0
0	0	0	0	0	0	0
0	0	0	0	0	0	0
0	0	0	0	0	0	0
27	147	0	0	0	0	13
0	0	0	0	0	0	0
0	44	0	0	0	0	0
0	42	0	0	0	0	0
0	0	0	0	0	0	0
0	0	0	0	0	0	0
0	0	0	0	0	0	0
10	0	0	0	0	0	0
0	0	0	0	0	0	0
0	0	0	0	0	0	0
0	0	0	10	0	0	0
0	0	0	0	0	0	0
0	0	0	0	0	0	0
0	23	0	23	0	0	0
0	0	51	0	0	0	0
0	65	0	0	0	0	0
0	0	21	0	0	0	0
0	16	0	0	0	0	0
0	0	10	0	0	0	0
0	0	0	0	0	0	0
12	0	0	0	0	0	0
0	0	0	0	0	0	0
0	0	0	0	0	0	0
0	885	15	0	0	0	0
0	0	0	0	0	0	0
0	0	0	0	0	0	0
0	29	0	0	0	0	0
0	0	81	0	0	0	0
0	0	0	0	0	0	0
0	967	92	0	0	0	35
0	0	0	0	0	0	0
0	0	0	0	0	0	0
0	0	0	0	0	0	0
0	0	0	0	0	0	0
0	0	0	0	0	0	0
0	0	0	0	0	0	0
0	156	0	0	0	0	0
0	0	0	0	0	0	0
0	402	0	0	0	0	0
0	0	0	0	0	0	0
0	0	0	0	0	0	0

0	0	0	0	0	0	0
0	0	0	0	0	0	0
0	0	0	0	0	0	0
0	0	0	0	0	0	0
0	0	0	0	0	0	0
0	0	0	0	0	0	0
0	0	0	0	0	0	0
0	123	24	0	0	0	0
0	0	0	0	0	0	0
0	0	0	0	0	0	0
0	0	0	0	0	0	0
0	0	0	0	0	0	0
0	0	0	0	0	0	0
0	0	0	0	0	0	0
0	68	184	0	0	0	13
0	0	10	0	0	10	10
0	0	0	0	0	0	0
0	0	0	0	0	0	0
0	0	0	0	0	0	0
0	0	0	0	0	0	0
0	63	0	0	0	0	0
0	0	0	0	0	0	126
0	200	0	0	0	0	0
0	71	0	0	0	0	0
0	0	0	0	0	0	0
0	0	0	0	0	0	0
0	0	0	0	0	11	0
0	0	0	0	0	0	0
0	0	0	0	0	0	0
12	0	0	0	0	0	39
0	0	0	0	0	0	0
0	0	0	0	0	0	0
11	0	0	0	0	0	0
0	58	0	0	0	0	0
0	0	0	0	0	34	0
0	0	0	0	0	0	0
0	0	0	11	0	0	0
0	10	0	186	0	0	0
0	0	0	13	0	0	0
0	0	34	21	0	0	0
0	0	11	11	0	0	11
0	0	0	33	0	0	0
0	0	0	21	0	0	0
0	0	0	18	0	0	15
0	0	0	40	0	0	0
0	0	0	34	0	0	0

0	0	0	10	0	0	0
0	0	0	29	0	0	0
0	104	0	16	0	0	0
0	0	0	11	0	0	0
0	0	69	27	0	0	0
0	0	0	10	0	0	79
0	0	0	29	0	0	0
0	0	0	13	0	0	0
0	0	10	11	0	0	0
0	0	0	31	0	0	0
0	0	10	12	0	0	0
0	0	25	15	0	0	0
0	0	0	135	0	0	0
0	0	10	15	0	0	10
0	10	0	11	0	0	0
0	0	0	10	0	0	0
0	0	60	10	0	0	10
0	0	0	11	0	0	0
0	0	0	12	0	0	0
0	0	12	11	0	0	13
0	0	0	11	0	12	0
72	0	63	11	0	0	0
0	0	10	14	0	0	0
0	0	0	21	0	0	0
0	397	0	78	0	0	0
0	30	0	47	0	0	0
0	0	0	26	0	0	0
0	0	0	54	0	0	0
0	0	0	32	0	0	0
0	0	0	12	0	0	0
0	0	0	12	0	0	0
0	0	0	43	0	0	0
0	94	0	55	0	0	0
0	47	0	10	0	0	0
0	1908	0	34	0	0	0
0	0	0	0	0	0	0
0	0	0	0	0	0	0
0	43	0	0	0	0	0
0	0	0	0	0	0	0
0	0	0	0	0	0	0
0	0	0	0	0	0	0
0	65	0	0	0	0	0
0	0	0	0	0	0	0
0	0	37	0	0	0	0
0	0	0	0	0	0	0
0	0	0	0	0	0	0

0	0	0	0	0	0	0
0	0	0	0	0	0	0
0	0	0	0	0	0	0
0	0	0	0	0	0	0
0	0	0	0	0	0	0
0	0	0	0	0	0	0
0	0	0	0	0	0	0
0	0	0	0	0	0	0
0	0	0	0	0	0	0
0	14	0	0	0	0	0
0	0	0	0	0	0	0
0	46	0	0	0	0	0
0	309	0	0	0	0	0
0	0	0	0	0	0	0
0	0	0	0	0	0	0
0	0	0	0	0	0	0
0	0	0	0	0	0	0
0	0	0	0	0	0	0
0	0	0	0	0	0	0
0	0	0	0	0	0	0
0	0	0	0	0	0	0
0	0	0	0	0	0	0
0	0	0	0	0	0	0
0	0	0	0	0	0	0
0	0	0	0	0	0	0
0	0	0	0	0	0	0
0	0	0	0	0	24	0
0	0	0	0	0	0	0
0	0	0	0	0	0	0
0	0	0	0	0	0	0
0	0	0	0	0	0	0
0	0	0	0	0	0	0
0	0	0	0	0	0	0
0	0	0	0	0	0	0
0	0	0	0	0	0	0
0	0	0	0	0	0	0
0	0	0	0	0	0	0
0	0	0	0	0	0	0
0	0	0	0	0	0	0
0	0	0	0	0	0	0
0	0	0	0	0	0	0
0	0	0	0	0	0	0
0	38	79	0	0	0	0
0	0	0	0	0	0	0
0	269	0	0	0	0	0
0	26	0	0	0	0	0

0	0	0	0	0	0	0
0	0	0	0	0	0	0
0	38	0	0	0	0	0
0	223	0	0	0	0	0
0	0	0	0	0	0	0
0	0	0	0	0	0	0
0	0	0	0	0	0	0
0	0	0	0	0	0	0
0	0	0	0	0	0	0
0	0	0	0	0	0	0
0	0	0	0	0	0	0
0	0	0	0	0	0	0
0	0	0	0	0	0	0
0	0	0	0	0	0	0
0	0	0	0	0	0	0
0	0	0	0	0	0	0
0	0	0	0	0	0	0
0	0	0	0	0	0	0
0	16	0	0	0	0	0
0	0	0	0	0	0	0
0	0	0	0	0	0	0
0	0	0	0	0	0	0
0	30	0	0	0	0	0
0	103	0	0	0	0	0
0	0	0	0	0	0	0
0	0	0	0	0	0	0
0	0	0	0	0	0	0
0	0	0	0	0	0	0
0	0	0	0	0	0	0
0	0	0	0	0	0	0
0	0	0	0	0	0	0
0	0	0	0	0	0	0
0	0	0	0	0	0	0
0	0	0	0	0	0	0
0	0	0	0	0	0	0
0	790	0	0	0	0	0
0	0	0	0	0	0	0
0	0	0	0	0	0	0
0	98	0	0	0	0	0
0	0	0	0	0	0	0
0	0	0	0	0	0	0
0	0	0	0	0	0	0
0	0	0	0	0	0	0
0	0	0	0	0	0	0
0	0	25	0	0	0	0
0	0	13	0	0	0	0

0	0	0	0	0	0	0
0	0	54	0	0	0	0
0	0	0	0	0	0	0
0	0	0	0	0	0	0
0	0	0	0	0	0	0
0	15	0	0	0	0	0
0	1037	0	0	0	0	0
0	0	0	0	0	0	0
0	0	0	0	0	0	0
0	0	0	0	0	0	0
0	769	0	0	0	0	0
0	199	0	0	0	0	0
0	56	0	0	0	0	0
0	73	0	0	0	0	0
0	0	13	0	0	0	0
0	0	0	0	0	0	0
0	0	0	0	0	0	0
0	0	0	0	0	0	0
0	0	0	0	0	0	0
0	0	0	0	0	0	0
0	0	0	0	0	0	0
0	0	0	0	0	0	0
0	0	0	0	0	0	0
0	0	13	0	0	10	14
0	43	0	0	0	0	0
0	0	0	0	0	0	0
0	0	0	0	0	0	0
0	0	0	0	0	0	0
0	0	0	0	0	0	0
0	0	0	0	0	0	0
0	15	0	0	0	0	0
0	0	0	0	0	0	0
0	0	0	0	0	0	0
0	0	0	0	0	0	0
0	11	0	0	0	0	0
0	0	0	0	0	0	0
0	0	0	0	0	0	0
0	0	0	0	0	0	0
0	0	0	0	0	0	11
0	0	0	0	0	0	0
0	0	0	0	0	0	0
0	105	0	0	0	0	0
0	0	0	0	0	0	0
0	0	0	0	0	0	0
0	0	0	0	0	0	0
0	0	0	0	0	0	0

0	0	0	0	0	0	0
0	0	0	0	0	0	0
0	0	0	0	0	0	0
0	0	0	0	0	0	89
0	0	0	0	0	0	0
0	0	0	0	0	0	0
0	0	0	0	0	0	0
0	0	0	0	0	0	0
0	0	0	0	0	0	0
0	0	0	0	0	0	0
0	0	0	0	0	10	0
0	11	0	0	0	0	0
0	0	0	0	0	0	0
10	13	0	0	0	0	0
0	0	0	0	0	0	0
0	0	0	0	0	0	0
0	0	10	0	0	0	0
0	0	0	0	0	0	0
0	0	0	0	0	0	10
0	0	0	0	0	0	0
0	0	0	0	0	0	0
0	0	0	0	0	0	0
0	0	0	0	0	0	0
0	0	0	0	0	0	0
0	0	0	0	0	0	0
0	0	0	0	0	0	0
0	0	0	0	0	0	0
0	0	0	0	0	0	0
0	0	0	0	0	0	0
0	0	0	0	0	0	0
0	0	0	0	0	0	0
0	0	0	0	0	0	0
0	0	0	0	0	0	0
0	0	0	0	0	0	0
0	0	0	0	0	0	0
0	39	0	0	0	0	0
0	0	0	0	0	0	0
0	15	0	0	0	0	0
0	0	0	0	0	0	0
0	0	0	0	0	0	0
0	0	0	0	0	0	0
0	0	0	0	0	0	0
0	0	0	0	0	0	39
0	0	0	0	0	0	0
0	0	0	0	0	0	0
0	0	0	0	0	0	0

0	0	0	0	0	0	0
0	0	0	0	0	0	0
0	0	0	0	0	17	0
0	0	0	0	0	0	0
0	0	0	0	0	0	0
0	16	0	0	0	0	0
0	0	0	0	0	0	0
0	0	0	0	0	0	0
0	0	0	0	0	0	0
0	0	0	0	0	0	0
0	0	0	0	0	0	0
0	0	0	0	0	0	0
0	0	57	0	0	0	0
0	0	0	0	0	0	0
0	0	0	0	0	0	0
0	85	0	0	0	0	0
0	0	0	0	0	0	0
0	0	0	0	0	0	0
0	0	0	0	0	0	0
0	0	0	0	0	0	0
0	0	0	0	0	10	0
0	0	0	0	0	0	0
0	0	0	0	0	0	0
0	0	0	0	0	0	0
0	0	0	0	0	0	0
0	0	0	0	0	0	0
0	0	0	0	0	0	0
0	0	0	0	0	0	0
0	0	0	0	0	0	0
0	0	0	0	0	0	0
0	112	0	0	0	0	0
0	10	0	0	0	0	0
0	10	0	0	0	0	0
0	0	0	0	0	0	0
0	0	0	0	0	15	0
0	0	0	0	0	0	0
0	46	0	0	0	46	0
0	0	29	0	0	0	0
0	0	0	0	0	0	0
0	129	0	0	0	0	0
0	0	0	0	0	0	0
0	0	0	0	0	0	0
0	0	0	0	0	0	0
0	0	0	0	0	0	0
0	0	0	0	0	0	0
0	0	0	0	0	0	0

0	0	0	0	0	0	0
0	0	0	0	0	0	0
0	0	0	0	0	0	0
0	0	0	0	0	0	0
0	0	0	0	0	0	0
0	0	0	0	0	0	0
0	28	0	0	0	0	0
0	11	0	0	0	0	36
0	0	0	0	0	0	0
0	0	0	0	0	0	0
0	0	19	0	0	0	10
0	0	0	0	0	0	0
0	15	0	0	0	0	0
0	0	28	0	0	0	0
0	0	32	0	0	0	11
0	0	0	0	0	0	0
0	0	0	0	0	0	0
0	0	0	0	0	0	0
0	0	0	0	0	0	0
0	0	0	0	0	0	0
0	0	0	0	0	0	0
0	0	35	0	0	0	36
0	0	0	0	0	0	0
0	0	0	0	0	0	0
0	0	0	0	0	0	0
0	0	10	0	0	0	0
0	0	0	0	0	0	0
0	0	0	0	0	0	0
0	0	0	0	0	0	12
0	0	0	0	0	0	0
0	0	0	0	0	0	0
0	0	0	0	0	0	0
0	0	0	0	0	0	345
0	0	0	0	0	0	0
0	0	0	0	0	0	0
0	0	0	0	0	0	0
0	0	0	0	0	0	0
0	0	0	0	0	0	0
0	0	0	0	0	0	0
0	0	0	0	0	0	0
24	0	0	0	0	0	0
0	0	0	0	0	0	0
0	0	0	0	0	0	0
0	0	0	0	0	0	10
0	0	0	0	0	0	0
0	0	27	0	0	0	0

0	0	0	0	0	0	12
0	0	0	0	0	0	0
0	0	57	0	0	0	0
0	0	0	0	0	0	0
0	0	0	0	0	0	0
0	0	0	0	0	0	0
0	0	0	0	0	0	0
0	0	0	0	0	0	0
0	0	0	0	0	0	0
0	81	0	0	0	0	0
0	0	0	0	0	0	0
0	0	36	0	0	0	71
0	0	0	0	0	0	0
0	0	0	0	0	0	0
0	0	0	0	0	0	0
0	0	0	0	0	0	0
0	0	0	0	0	0	0
0	0	0	0	0	0	0
0	0	0	0	0	0	0
0	0	0	0	0	0	0
0	0	0	0	0	0	0
0	0	12	0	0	0	0
0	0	10	0	0	0	13
0	0	0	0	0	0	0
0	0	0	0	0	0	0
0	0	0	0	0	0	0
0	0	0	0	0	0	0
0	0	0	0	0	0	0
0	0	13	0	0	0	0
0	0	0	0	0	0	0
0	0	0	0	0	0	0
0	0	0	0	0	0	0
0	0	0	0	0	0	10
0	172	0	0	0	0	0
0	0	25	0	0	0	0
0	0	0	0	0	0	0
0	0	0	0	0	0	0
0	0	0	0	0	0	0
0	0	0	0	0	0	0
0	0	0	0	0	0	0
0	0	0	0	0	0	0
0	0	0	0	0	0	0
0	0	0	0	0	0	0
0	0	0	0	0	0	0
0	0	0	0	0	0	0
0	0	0	0	0	10	0

0	0	97	0	0	0	0
0	0	0	0	0	0	0
0	0	0	0	0	0	0
0	0	0	0	0	0	0
0	0	0	0	0	0	0
0	0	0	0	0	0	0
0	0	36	0	0	0	0
0	0	0	0	0	0	10
0	0	0	0	0	0	0
0	0	0	0	0	0	0
0	0	0	0	0	0	0
0	0	64	0	0	0	0
0	0	60	0	0	0	0
0	0	27	0	0	0	0
0	0	284	0	0	0	0
0	0	13	0	0	0	0
0	0	0	0	0	0	0
0	13	0	0	0	0	0
0	33	10	0	0	0	0
0	0	0	0	0	0	0
0	14	0	0	0	0	0
0	0	0	0	0	0	0
0	48	0	0	0	0	0
0	0	10	0	0	0	0
0	0	10	0	0	0	0
0	0	0	0	0	0	0
0	0	0	0	0	0	123
0	0	0	0	0	0	0
0	0	0	0	0	0	0
0	0	0	0	0	0	0
0	0	0	0	0	0	0
0	0	0	0	0	0	0
0	0	0	0	0	0	0
0	0	0	0	0	0	0
0	0	0	0	0	0	0
0	0	0	0	0	0	0
0	32	0	0	0	0	0
0	0	0	0	0	0	0
0	0	0	0	0	0	0
0	0	0	0	0	0	11
0	0	0	0	0	0	45
0	0	0	0	0	0	120
0	0	0	0	0	0	10
0	0	0	0	0	0	12
0	0	0	0	0	0	48
0	0	0	0	0	0	42

0	0	0	0	0	0	11
0	0	0	0	0	0	97
0	0	0	0	0	0	110
0	0	0	0	0	0	11
0	0	0	0	0	0	10
0	0	0	0	0	0	87
0	0	0	0	0	0	72
0	0	0	0	0	0	11
0	0	0	0	0	0	11
0	0	0	0	0	0	58
0	51	0	0	0	0	11
0	0	0	0	0	0	85
0	0	0	0	0	0	12
0	0	0	0	0	0	62
0	0	0	0	0	0	170
0	0	0	0	0	0	15
0	0	0	0	0	0	10
0	0	0	0	0	0	12
0	0	0	0	0	0	10
0	14	0	0	0	0	0
0	18	0	0	0	0	0
0	51	0	0	0	0	0
0	10	0	0	0	0	0
0	52	0	0	0	0	0
0	354	0	0	0	0	0
0	14	0	0	0	0	0
0	202	0	0	0	0	0
0	28	0	0	0	0	0
0	29	0	0	0	0	0
0	11	0	0	0	0	0
0	11	0	0	0	0	0
0	141	0	0	0	0	0
0	40	0	0	0	0	0
0	113	0	0	0	0	0
0	55	0	0	0	0	0
0	10	0	0	0	0	0
0	14	0	0	0	0	0
0	11	0	0	0	0	0
0	16	13	0	0	0	0
0	12	0	0	0	0	0
0	22	0	0	0	0	0
0	45	0	0	0	0	0
0	14	0	0	0	0	0
0	65	0	0	0	0	0
0	31	0	0	0	0	0
0	48	0	0	0	0	0

0	10	0	0	0	0	0
0	10	0	0	0	0	0
0	47	0	0	0	0	0
0	32	0	0	0	0	0
0	12	0	0	0	0	0
0	34	0	0	0	0	0
0	12	0	0	0	0	0
0	68	0	0	0	0	0
0	27	0	0	0	0	0
0	27	0	0	0	0	0
0	37	0	0	0	0	0
0	25	0	0	0	0	0
0	13	0	0	0	0	0
0	59	0	0	0	0	0
0	212	0	0	0	0	0
0	25	0	0	0	0	0
0	12	0	0	0	0	0
0	46	0	0	0	0	0
0	11	0	0	0	0	0
0	45	0	0	0	0	0
0	11	0	0	0	0	0
0	207	0	0	0	0	0
0	41	0	0	0	0	0
0	71	0	0	0	0	0
0	43	0	0	0	0	0
0	0	0	0	0	0	0
0	0	0	0	0	0	0
0	0	0	0	0	0	0
0	0	0	0	0	0	0
0	0	0	0	0	0	0
0	0	0	0	0	0	0
0	0	0	0	0	0	0
0	0	0	0	0	0	0
0	0	0	0	0	0	0
0	0	0	0	0	0	0
0	0	0	0	0	0	0
0	0	0	0	0	0	0
0	0	10	0	0	0	0
0	0	0	0	0	0	0
0	0	0	0	0	0	0
0	0	0	0	0	0	0
0	0	14	0	0	0	0
0	0	0	0	0	0	0
0	0	0	0	0	0	0
0	0	0	0	0	0	0
0	0	0	0	0	0	0

0	0	0	0	0	0	0
0	0	0	0	0	0	0
0	0	0	0	0	0	0
0	0	0	0	0	0	0
0	0	0	0	0	0	0
0	0	0	0	0	0	0
0	0	0	0	0	0	0
0	0	0	0	0	0	0
0	0	0	0	0	0	0
0	0	0	0	0	0	0
0	0	0	0	0	0	0
0	0	0	0	0	0	0
0	0	0	0	0	0	0
0	0	0	0	0	0	0
0	0	0	0	0	0	0
0	0	0	0	0	0	0
0	0	0	0	0	0	0
0	0	0	0	0	0	0
0	0	0	0	0	0	0
0	0	0	0	0	0	0
0	0	0	0	0	0	0
0	0	0	0	0	0	0
0	0	0	0	0	0	0
0	0	0	0	0	0	0
0	0	0	0	0	0	0
0	0	0	0	0	0	0
0	0	0	0	0	0	0
0	0	0	0	0	0	0
0	0	0	0	0	0	0
0	0	0	0	0	0	0
0	0	0	0	0	0	0
0	0	0	0	0	0	0
0	0	0	0	0	0	0
0	0	10	0	0	0	0
0	0	12	0	0	0	0
0	0	17	0	0	0	0
0	0	11	0	0	0	0
0	0	14	0	0	0	0
0	0	66	0	0	0	0
0	0	48	0	0	0	0
0	0	24	0	0	0	0
0	0	12	0	0	0	0
0	0	12	0	0	0	0
0	0	10	0	0	0	0
0	0	13	0	0	0	0
0	0	21	0	0	0	0
27	0	0	0	0	0	0

55	0	0	0	0	0	0
14	0	0	0	0	0	0
0	0	0	0	0	0	0
0	0	0	0	0	0	0
0	0	0	0	0	0	0
0	0	0	0	0	0	0
0	0	0	0	0	0	0
0	0	0	0	0	0	0
0	0	0	0	0	0	0
0	0	0	0	0	0	0
0	0	0	0	0	0	0
0	0	0	0	0	0	0
0	0	0	0	0	0	0
0	0	0	0	0	0	0
0	0	0	0	0	0	0
0	0	0	0	0	0	0
0	0	0	0	0	0	0
0	0	0	0	0	0	0
0	0	0	0	0	0	0
0	0	0	0	0	0	0
0	0	0	0	0	0	0
0	0	0	0	0	0	0
0	0	0	0	0	0	0
0	0	0	0	0	0	0
0	0	0	0	0	0	0
0	0	0	0	0	0	0
0	0	0	0	0	0	0
0	0	0	0	0	0	0
0	0	0	0	0	0	0
0	0	0	0	0	0	0
0	0	0	0	0	0	0
0	0	0	0	0	0	0
0	0	0	0	0	0	0
0	0	0	0	0	0	0
0	0	0	0	0	0	0
0	0	0	0	0	0	0
0	0	0	0	0	0	0
0	0	0	0	0	0	0
0	0	0	0	0	0	0
0	0	0	0	0	0	0
0	0	0	0	0	0	0
0	0	0	0	0	0	0
0	0	0	0	0	0	0
0	0	0	0	0	0	0
0	0	0	0	0	0	0
0	0	0	0	0	0	0

0	0	0	0	0	0	0
0	0	0	0	0	0	0
0	0	0	0	0	0	0
0	0	0	0	0	0	0
0	0	0	0	0	0	0
0	0	0	0	0	0	0
0	0	0	0	0	0	0
0	0	0	0	0	0	0
0	0	0	0	0	0	0
0	0	0	0	0	0	0
0	0	0	0	0	0	0
0	0	0	0	0	0	0
0	0	0	0	0	0	0
0	0	0	0	0	0	0
0	0	0	0	0	0	0
0	0	0	0	0	0	0
0	0	0	0	0	0	0
0	0	0	0	0	0	0
0	0	0	0	0	0	0
0	0	0	0	0	0	0
0	0	0	0	0	0	0
0	0	0	0	0	0	0
0	0	0	0	0	0	0

CS_324	CS_329	CS_332	CS_333	CS_340	CS_341	CS_348
0	0	0	61	0	0	0
0	0	0	11	0	0	0
0	12	38	12	64	12	93
0	0	0	13	40	28	338
0	0	0	31	0	12	240
0	0	0	17	0	0	48
0	0	0	12	0	13	33
0	0	0	0	28	0	0
0	0	0	36	74	10	0
10	0	16	82	90	106	173
0	0	0	0	79	0	0
0	0	0	0	0	0	0
0	0	0	24	0	0	0
0	21	0	0	0	0	0
0	14	0	0	0	53	0
0	0	0	0	10	0	16
478	158	14	973	353	2639	953
0	14	28	0	13	167	247
0	0	0	36	27	21	0
0	130	0	108	145	41	202
0	0	0	58	0	0	12
0	0	0	58	11	0	54
0	0	0	57	0	0	270
0	0	0	0	0	0	0
702	571	345	1101	2468	432	10130
98	20	66	274	1160	70	498
117	35	0	299	1583	248	377
13	13	0	71	882	57	271
41	66	12	336	2625	186	1186
0	0	0	29	30	0	0
0	0	0	46	32	0	11
0	0	0	45	672	0	0
597	0	0	1022	4833	1406	146
0	0	0	0	0	0	0
0	0	0	23	0	12	250
0	0	0	23	0	0	0
0	0	0	44	0	0	23
179	57	38	775	152	199	169
0	13	81	24	51	181	86
2095	129	211	2177	3740	1889	697
43	0	0	123	223	87	0

77	0	0	87	232	149	0
20	0	0	220	345	141	96
0	0	0	355	203	243	131
795	1379	948	448	1294	1184	2209
100	577	96	440	93	857	790
0	0	0	0	108	0	0
0	59	73	115	13	0	43
0	0	0	0	186	0	0
0	0	0	14	63	0	0
0	0	0	0	90	0	0
0	0	0	0	0	0	0
0	0	0	0	54	0	0
94	24	55	68	162	38	0
0	0	0	0	41	0	0
0	0	0	0	20	0	0
0	0	0	0	259	0	0
0	0	0	0	18	0	0
229	86	58	1264	1089	165	1501
56	99	79	364	147	54	826
0	0	0	81	10	0	0
0	0	0	29	88	0	0
0	0	0	0	0	0	0
0	0	0	0	0	0	0
0	0	33	10	0	26	10
0	0	0	0	28	0	0
0	0	0	0	0	0	0
0	0	0	0	28	0	0
0	0	0	0	0	0	0
0	0	0	0	0	0	0
0	0	45	30	26	12	80
0	0	0	13	592	25	0
285	260	348	589	866	624	404
10	0	12	114	13	27	37
0	0	0	0	47	0	0
0	0	0	0	39	0	0
0	0	0	0	40	0	0
0	0	0	0	55	0	0
0	0	0	0	70	0	0
0	0	0	0	0	0	0
0	0	0	0	38	0	0
0	0	0	0	388	10	0
0	0	0	31	10	24	0
0	0	0	0	0	0	0
0	0	0	0	0	0	0
0	0	0	27	371	19	0
93	265	174	308	1595	242	150

0	0	0	11	22	0	0
19	48	84	142	55	128	250
0	0	0	10	21	14	0
0	0	0	0	49	0	0
124	249	123	108	1003	290	135
0	0	0	55	0	57	10
0	0	0	11	0	0	0
0	0	0	10	0	0	0
0	0	0	0	0	0	0
0	0	0	0	0	0	0
37	0	10	118	0	65	78
0	0	0	51	38	0	0
753	635	590	7985	2571	1976	6643
0	0	0	0	0	0	0
42	82	59	947	366	225	504
0	0	0	0	74	13	24
0	0	0	113	51	38	49
0	0	0	0	302	12	0
0	0	0	43	13	0	55
0	0	0	0	96	0	0
0	0	0	0	14	0	0
0	0	0	0	10	0	0
0	27	0	0	0	15	73
0	0	0	333	108	27	0
0	0	0	283	360	135	82
0	0	0	529	178	64	74
0	0	0	311	100	27	59
0	0	0	16	74	0	32
10	0	0	41	98	19	66
40	167	0	1203	517	367	970
0	0	0	71	24	12	191
0	0	0	86	0	0	0
0	80	0	11	0	116	97
0	72	41	120	13	202	608
0	0	0	0	26	0	0
0	0	0	42	10	0	0
0	0	0	119	31	14	11
0	0	0	154	48	21	33
0	0	0	0	73	0	0
21	0	0	1148	450	139	217
0	0	0	54	40	0	10
12	0	0	114	0	0	25
0	0	0	34	93	0	16
1804	1912	1338	3429	2215	1377	28772
0	0	0	56	171	0	329
124	0	62	448	1500	135	1583

0	0	0	26	0	0	10
32	0	0	518	204	10	55
93	10	0	451	311	60	160
997	134	370	650	982	459	159
0	0	0	11	66	0	0
271	35	104	130	68	54	38
0	0	0	0	0	0	0
0	0	0	0	26	13	0
0	0	0	0	24	0	0
0	0	0	0	0	0	0
53	0	0	40	63	27	79
0	46	10	194	11	0	271
0	0	0	0	76	0	0
0	0	0	55	519	0	0
0	0	0	321	719	0	0
0	0	0	0	0	0	0
0	0	0	30	31	0	11
0	0	0	0	0	0	0
0	0	0	0	0	0	0
0	0	0	0	0	0	0
53	0	0	146	37	91	64
0	0	0	0	0	0	0
32	0	0	365	162	75	326
0	0	0	0	0	0	0
0	0	0	62	36	10	0
0	0	0	71	412	33	54
51	0	29	349	45	85	159
160	69	64	745	343	449	906
0	41	15	42	0	27	98
0	0	0	24	119	15	29
12	0	0	0	0	0	14
80	23	11	190	14	25	387
20	0	51	127	359	0	183
0	0	0	33	0	16	60
54	25	0	228	522	11	287
0	0	0	122	213	12	38
0	0	0	0	214	14	0
0	0	0	0	0	0	0
0	0	0	20	0	0	0
0	0	0	0	21	0	0
0	0	0	238	0	54	0
0	0	0	0	13	0	0
0	0	0	0	75	0	0
0	0	0	0	0	0	0
0	34	0	57	11	22	180
12	0	0	54	0	258	2116

0	0	0	47	0	70	812
0	0	10	71	0	76	1274
107	113	768	582	200	976	33269
23	0	142	797	14	1962	6270
0	0	12	0	71	0	0
50	320	0	125	184	20	603
21	78	102	238	125	151	178
0	0	0	0	0	0	0
0	0	0	0	0	0	0
0	0	0	0	0	0	0
0	0	0	0	0	0	0
0	0	0	0	0	0	0
0	72	0	0	0	0	228
135	393	181	630	119	871	2319
0	0	0	0	0	45	315
47	103	26	26	341	201	11
0	0	0	0	0	0	10
0	0	0	10	0	0	180
0	0	0	0	0	0	0
0	0	0	0	0	0	0
0	0	0	0	0	0	0
96	0	0	621	134	0	466
0	0	0	0	125	0	12
0	0	0	0	0	0	0
0	0	0	0	53	0	26
0	0	0	69	52	10	69
0	0	0	58	823	0	38
0	0	0	0	0	0	0
0	0	0	0	0	0	0
0	0	0	0	0	0	0
0	0	0	0	0	0	0
0	0	0	0	11	0	0
0	0	0	0	11	0	0
0	0	0	0	0	0	0
0	0	0	0	0	0	0
88	16	0	72	619	10	46
789	380	85	4132	8476	368	1268
241	238	14	2104	5585	221	1204
0	0	0	131	166	0	43
0	0	0	0	0	0	0
0	0	0	30	0	0	34
60	172	234	168	1084	74	102
21	0	0	464	682	63	68
0	0	0	65	43	14	0
0	0	0	0	137	0	0
43	199	128	31	489	69	25

12	75	41	0	269	22	0
0	98	110	29	1776	24	13
0	40	11	37	486	10	628
0	0	0	32	0	0	0
0	0	0	246	47	33	0
315	60	36	2676	3890	210	223
0	0	29	260	232	51	106
0	0	0	62	96	0	0
0	0	0	0	0	27	0
0	0	0	0	0	0	0
0	0	0	0	819	0	0
0	0	0	0	0	0	0
0	0	22	0	0	0	0
0	0	0	478	365	116	158
0	0	0	0	0	47	10
0	0	0	0	0	0	0
0	0	0	0	44	0	0
0	0	10	0	70	0	0
359	290	68	1442	1268	105	263
0	0	0	0	0	0	76
0	0	0	121	0	0	0
0	0	0	97	0	0	28
0	0	0	175	1405	0	0
0	0	0	71	100	14	0
464	704	22	1298	2730	696	1003
0	0	0	82	326	45	35
0	0	0	0	0	0	0
0	118	161	502	808	262	113
0	0	40	527	134	108	92
11	0	0	252	42	27	45
0	54	0	32	218	0	128
0	0	0	188	39	39	36
0	0	0	0	26	0	0
0	0	0	72	48	32	35
146	1339	90	1144	2088	163	1491
0	0	0	120	305	0	23
0	0	0	145	238	0	34
0	0	0	36	15	0	0
0	0	0	0	39	0	0
0	0	27	337	126	59	89
0	0	0	226	115	17	64
0	0	0	76	114	0	93
0	0	46	0	53	0	0
236	259	1243	790	242	322	458
0	0	0	0	0	0	0
0	40	19	775	3529	0	187

157	37	61	3447	3333	71	92
0	0	0	55	32	0	0
0	0	0	290	209	0	0
603	1044	197	569	1711	0	697
0	0	0	228	152	0	0
0	0	0	87	60	0	40
41	15	0	142	196	0	0
85	0	0	454	642	0	79
0	0	0	52	24	0	0
0	0	0	14	0	0	0
0	0	0	229	0	0	0
0	0	0	181	126	0	0
230	160	276	3161	1687	384	502
27	0	0	570	437	47	12
0	0	0	219	46	45	0
57	12	49	355	79	103	166
66	0	66	1340	291	314	163
0	0	0	34	42	0	0
0	0	0	59	13	28	64
0	0	22	68	90	32	151
0	0	23	0	12	0	33
11	0	0	59	441	66	40
267	55	37	253	1068	385	387
69	34	36	77	302	65	1791
18	0	27	55	412	18	519
0	0	0	76	125	33	11
3689	496	393	1622	3910	10642	9926
375	77	45	110	645	1983	689
67	0	0	30	198	344	321
27	0	0	183	93	662	38
269	0	38	2137	673	2087	399
614	59	25	248	344	2533	262
232	0	12	29	1060	842	438
0	0	0	97	0	0	36
243	0	0	82	362	1489	59
14	0	0	222	961	10	218
0	0	0	47	111	0	0
336	43	42	248	4221	629	1252
112	36	41	360	1141	40	1531
132	0	0	347	2729	178	293
44	0	52	898	2267	65	38
61	0	51	865	2035	21	15
0	0	13	0	387	27	0
36	0	10	118	239	53	33
32	0	34	218	202	14	89
0	0	0	25	43	0	0

13	0	0	450	827	12	267
450	93	130	80	13	30	79
13	0	0	131	294	0	0
179	66	216	98	147	22	174
33	0	0	35	0	0	21
0	0	0	0	0	0	0
12	10	15	48	55	31	164
28	0	28	58	49	0	43
0	0	0	82	11	0	0
0	0	0	36	0	0	59
0	0	0	0	0	0	0
278	12	88	445	40	0	175
0	61	41	0	0	0	688
36	0	0	72	0	0	15
0	0	0	0	0	0	0
0	0	0	0	22	0	0
0	0	122	24	0	0	241
0	0	0	21	0	0	85
249	302	339	825	1039	442	299
0	0	0	0	0	0	0
0	0	0	0	0	0	0
0	0	0	0	0	0	0
0	0	0	0	0	0	0
11	0	116	409	1251	146	61
0	0	0	0	0	0	0
0	0	0	0	45	0	0
15	0	0	48	146	0	0
12	0	0	69	131	0	0
0	0	0	0	26	0	0
0	0	0	0	37	0	0
0	0	0	0	0	0	0
0	0	0	0	0	0	0
0	0	0	0	0	0	0
0	0	0	0	15	0	0
0	61	0	170	288	15	125
0	0	0	0	10	0	0
0	0	0	0	0	13	0
0	0	0	0	0	0	0
0	0	0	0	0	0	0
0	0	0	0	0	33	19
0	0	0	97	0	0	0
0	0	0	38	10	0	0
22	0	33	72	24	62	69
42	0	29	239	33	52	53
0	0	0	0	0	0	0
0	0	0	0	0	0	0

0	11	0	100	58	16	124
0	0	0	0	0	0	0
0	0	0	30	0	0	89
343	184	270	3813	264	1230	1526
0	0	0	12	18	17	0
0	38	0	33	38	0	17
0	0	0	66	30	0	45
0	0	0	90	57	39	93
0	21	0	11	10	136	169
0	0	0	71	449	0	0
0	73	0	107	139	23	170
95	979	87	463	224	227	1717
0	51	0	75	527	22	472
79	360	157	267	383	376	728
0	0	0	30	104	0	101
0	0	0	0	0	0	0
0	0	0	0	0	0	0
0	0	0	0	97	13	11
0	0	0	0	0	0	0
0	0	0	0	0	0	0
0	0	0	41	58	90	23
0	0	0	0	0	0	0
0	0	0	68	71	0	86
0	0	0	70	459	125	218
0	0	0	0	0	0	0
0	0	0	0	11	0	0
0	0	0	41	15	13	14
0	0	0	0	0	0	0
183	173	240	1020	232	471	639
0	0	0	39	0	0	0
0	0	0	11	37	0	0
0	0	0	155	46	0	0
0	0	0	62	27	12	0
0	0	0	0	0	0	0
0	0	0	0	0	0	0
0	0	0	0	0	0	0
0	0	0	0	0	0	0
41	439	61	358	333	383	1838
0	0	0	0	0	0	0
0	0	0	17	100	0	14
0	31	0	23	101	31	344
0	0	0	12	22	0	39
0	0	0	0	24	0	0
0	0	0	0	0	0	0
0	0	0	0	0	0	0
0	0	0	0	0	0	0

13	0	0	144	94	10	0
0	0	0	0	0	0	41
0	0	0	0	0	0	0
0	0	0	0	0	0	0
0	0	0	0	26	0	0
0	0	0	0	42	0	0
0	0	0	0	0	0	0
0	0	0	31	209	125	10
357	764	213	2889	1558	932	2795
79	95	25	214	27	79	181
0	0	0	71	207	0	0
46	381	239	849	442	311	828
0	0	0	0	0	0	0
0	0	0	0	0	14	0
0	0	0	69	0	0	0
0	0	17	2291	526	420	260
0	0	0	76	0	0	0
0	0	0	0	25	0	0
0	0	0	12	22	0	11
0	0	0	26	25	0	0
0	0	0	0	0	0	0
0	25	0	0	0	11	88
0	13	0	0	0	37	78
18	0	0	386	51	59	166
60	38	494	166	1474	180	245
363	288	114	1476	2318	264	900
73	130	26	119	234	40	436
11	0	0	57	440	0	0
56	0	30	155	810	0	140
0	0	0	12	23	0	0
0	0	0	0	10	0	0
0	0	0	0	12	0	0
0	0	0	46	198	0	0
73	54	0	58	62	0	62
134	48	0	292	494	48	102
0	0	0	52	0	0	11
0	27	23	0	894	40	56
874	2009	1565	957	11929	1153	1765
29	58	21	141	330	121	246
0	0	0	0	15	0	0
35	123	184	24	2290	99	31
0	0	0	0	19	0	0
0	11	0	0	45	0	0
0	0	0	0	271	0	0
0	0	0	0	0	0	0
0	0	0	15	269	109	12

0	0	0	0	294	0	0
0	0	0	0	157	0	0
0	0	0	0	0	0	0
42	15	59	10	1852	45	0
0	14	0	54	0	21	37
0	0	0	114	1057	0	14
0	0	0	222	214	43	34
54	126	10	39	665	0	52
0	0	0	92	124	37	0
0	0	0	0	55	0	10
134	203	69	248	1562	45	570
18	0	0	0	181	12	0
0	0	0	0	31	0	0
18	0	31	12	10	0	10
0	0	0	0	55	0	11
0	0	0	0	0	0	0
0	0	0	0	0	0	0
0	0	0	0	0	0	0
40	32	14	451	60	125	244
0	0	0	0	0	0	14
0	0	0	124	37	0	16
0	0	0	0	0	0	0
0	0	0	41	742	0	0
0	0	0	0	397	0	0
14	0	38	39	1479	0	0
0	0	0	0	57	0	0
0	0	0	0	0	0	0
0	0	0	0	103	0	0
0	0	0	12	15	0	0
0	0	0	0	42	0	0
0	0	0	0	93	0	0
0	0	0	0	32	0	0
0	0	0	0	22	0	0
0	0	0	21	0	0	0
0	0	0	0	0	0	0
0	0	0	0	90	0	0
0	20	38	13	182	14	0
0	27	0	88	444	0	71
1884	1378	1988	15145	4419	4095	6679
0	0	0	0	128	0	0
0	0	0	0	0	0	0
0	0	0	0	32	0	0
38	31	0	153	101	15	123
0	55	10	12	110	65	300
0	0	0	0	36	0	0
0	0	0	0	0	0	0

0	0	0	0	0	0	0
21	0	0	90	657	25	0
0	0	0	14	53	0	0
0	0	0	0	0	0	0
0	0	0	0	139	0	0
0	0	0	11	13	53	53
0	0	0	0	0	0	0
0	27	0	0	0	0	12
0	0	0	0	0	0	0
0	59	0	33	0	0	108
0	0	0	0	11	33	0
0	0	0	102	444	0	0
187	0	187	8206	3148	1150	799
10	0	12	694	318	104	195
0	0	0	0	316	0	0
0	0	0	0	0	0	0
0	0	0	0	34	0	0
0	0	0	0	17	0	0
0	0	16	254	123	10	0
35	22	70	2161	824	494	309
15	0	29	1271	453	128	288
51	22	31	438	47	109	104
56	292	38	23	105	12	361
0	0	0	0	11	0	0
0	0	0	15	0	0	0
0	0	0	0	0	0	0
0	0	0	0	77	0	0
0	0	10	1187	285	89	122
0	0	0	21	257	0	38
0	0	0	0	94	62	28
0	0	0	0	34	0	0
0	0	0	0	0	0	0
258	41	27	1656	1040	71	141
0	0	0	17	0	150	22
0	0	0	57	0	42	16
0	0	0	0	0	0	0
16	0	0	651	710	0	29
0	0	0	128	238	0	0
0	0	10	310	887	40	205
74	0	0	1019	1477	25	13
0	0	0	89	345	0	0
0	0	0	0	0	0	31
0	0	37	13	34	0	11
118	137	20	1075	3783	47	147
0	0	0	139	0	0	0
0	0	0	0	0	0	0

0	0	0	0	0	0	0
0	0	0	33	0	0	0
0	0	0	0	0	0	0
0	0	14	171	89	0	10
170	105	0	784	712	21	205
36	69	11	414	1187	10	87
10	0	0	586	745	14	0
0	0	0	17	252	0	0
258	1251	58	284	299	0	352
0	0	20	0	29	10	12
0	0	0	0	32	0	0
0	0	0	59	296	0	0
0	0	0	89	94	0	0
0	0	0	26	0	0	0
0	0	0	0	0	0	0
0	0	0	0	0	0	0
0	0	0	0	0	0	0
0	22	0	66	215	0	10
0	0	0	14	36	0	0
0	0	0	139	0	0	0
0	0	1282	1235	164	95	0
0	0	57	0	16	0	0
45	0	515	2923	1117	532	62
0	0	0	33	0	0	0
78	0	190	3185	583	701	320
0	0	12	12	0	0	0
0	0	175	158	116	0	0
61	0	274	1813	133	475	142
0	0	0	30	0	0	0
0	0	0	0	0	0	0
0	0	0	0	0	0	0
12	0	0	64	0	0	423
0	0	0	0	0	0	79
0	0	0	48	16	0	0
0	0	0	74	130	0	41
0	0	0	0	44	0	0
0	0	0	110	12	0	92
0	0	0	0	0	0	11
0	0	0	27	10	0	16
0	0	0	0	840	0	0
0	0	0	0	0	13	1179
156	0	0	70	0	0	59
0	11	12	722	578	126	1905
0	0	12	50	26	14	0
0	54	0	129	22	14	111
0	0	0	0	59	28	0

0	0	0	16	44	0	0
112	11	0	282	3019	40	87
27	0	0	198	1344	0	0
0	0	0	75	113	27	167
0	0	0	0	51	0	28
0	0	0	59	424	54	22
0	0	0	12	242	56	180
0	0	0	101	207	40	90
515	817	214	16314	3155	1240	3069
68	53	469	4772	2070	575	211
0	27	177	2140	692	367	69
0	0	0	26	0	47	111
0	0	0	0	0	0	0
0	63	0	25	182	65	165
0	0	0	0	0	0	0
0	66	11	30	42	234	98
0	0	0	27	81	0	0
0	0	0	67	38	0	0
0	0	0	13	34	0	0
0	0	0	0	0	0	0
0	14	0	185	132	13	168
85	39	11	214	348	41	272
0	0	0	45	157	0	124
0	0	0	69	0	0	0
0	0	0	0	0	0	0
108	0	0	522	1895	40	26
877	2214	272	2279	9652	1003	544
520	254	158	2106	2044	158	617
4842	10633	4176	9712	25809	2504	12527
40	0	0	132	687	33	0
47	0	0	139	706	0	0
0	0	0	170	217	0	0
660	1615	309	2681	7013	412	598
799	216	108	1610	2615	381	373
25	0	0	184	472	0	0
0	0	0	0	123	0	0
314	2674	86	2608	6643	695	5872
61	247	119	1445	3706	271	4736
26	345	12	1215	729	348	2251
0	124	101	457	422	667	1586
0	0	0	0	0	0	47
0	0	0	0	0	0	0
150	3363	255	1092	2679	1123	7290
0	33	0	140	14	0	175
0	0	0	0	0	48	0
87	166	100	840	4226	388	2837

91	90	34	785	1477	33	638
351	287	870	28920	46669	3036	3434
0	0	0	23	0	30	0
50	0	0	1253	31948	110	21
27	44	38	154	277	29	344
0	0	0	70	61	0	0
249	131	14	1868	2421	88	777
0	0	33	553	235	13	0
0	0	0	0	0	0	0
165	0	0	75	46	34	89
451	38	12	215	602	82	473
0	24	0	92	1057	25	97
0	0	0	0	0	0	0
0	0	0	0	30	0	0
0	32	26	152	81	0	299
138	127	23	583	3361	266	149
189	242	32	274	318	266	1841
10	0	0	56	397	72	68
0	0	0	83	81	0	78
0	0	0	60	25	28	153
28	11	0	195	291	94	248
0	0	0	0	115	0	0
0	0	0	0	34	0	0
0	0	0	11	21	0	0
24	0	0	337	3765	0	0
0	0	0	0	0	0	0
0	0	0	0	0	0	0
0	0	0	0	26	0	0
0	0	0	0	0	0	0
0	0	0	64	0	0	464
0	0	0	28	325	0	0
0	0	0	0	0	0	0
0	0	0	463	697	0	11
23	0	0	573	4606	31	36
0	181	0	119	40	105	103
0	0	0	89	27	44	66
24	12	0	124	144	0	31
156	134	0	16	0	0	102
215	218	44	685	3995	58	178
0	0	0	99	59	35	0
0	0	63	83	33	22	11
0	11	17	10	343	86	0
0	0	0	0	0	0	0
0	0	0	0	463	29	0
0	0	0	0	255	0	0
0	0	0	12	517	17	0

0	0	0	0	0	0	0
30	0	0	0	297	0	53
0	0	11	54	230	13	49
0	0	0	0	61	0	0
0	0	0	362	119	0	10
0	0	0	0	0	0	0
0	0	0	0	0	0	0
0	0	0	0	0	0	0
0	0	0	43	0	0	0
0	0	0	0	421	0	58
0	0	0	16	0	0	0
0	0	0	143	0	0	0
11	0	0	95	52	492	35
0	26	11	239	0	541	80
0	0	0	207	94	932	64
65	0	0	93	18	46	0
0	12	0	40	0	178	42
0	0	0	63	154	14	0
0	0	0	65	0	0	0
0	0	0	16	0	0	0
0	0	0	10	68	12	527
14	0	0	47	308	34	29
0	0	0	11	53	0	19
0	0	0	162	0	0	0
0	0	0	60	93	0	0
0	0	0	54	0	0	11
0	0	0	13	0	0	0
0	0	0	29	0	20	0
242	0	0	11	58	0	0
0	0	0	67	0	0	0
0	0	0	10	0	0	11
0	0	0	19	0	0	0
0	0	0	41	23	0	0
0	0	0	22	16	0	51
10	0	33	22	0	0	35
0	0	0	10	0	0	0
0	0	0	22	0	0	0
0	0	0	36	0	0	0
0	0	0	45	0	0	0
0	0	0	58	0	0	0
0	0	0	27	0	0	0
0	0	0	26	0	0	0
0	0	0	28	0	0	0
0	0	0	11	0	0	0
0	0	0	10	0	0	0
0	0	0	53	0	0	0

0	0	0	12	0	0	0
0	0	0	24	0	0	0
0	0	0	33	0	0	0
0	0	0	21	219	0	71
0	0	0	10	0	0	0
0	0	0	10	0	0	0
0	0	0	129	24	10	102
0	0	0	12	0	22	13
0	0	0	13	0	0	0
0	0	0	29	58	0	28
0	0	0	12	0	0	0
0	0	0	11	0	0	0
0	31	0	62	0	87	204
0	0	0	22	0	11	0
23	0	0	25	0	0	0
0	0	0	22	0	0	0
0	0	0	27	0	0	14
0	0	0	41	0	0	0
12	0	0	85	233	30	662
0	0	0	41	0	0	0
0	0	0	10	0	0	0
0	0	0	12	11	0	0
0	0	0	90	0	0	0
0	0	0	100	27	0	0
0	0	0	12	0	0	0
0	0	0	10	0	0	26
0	0	0	98	0	10	23
0	0	0	71	0	0	0
0	0	0	34	0	0	0
0	0	0	26	0	0	0
0	0	0	10	209	0	0
0	0	0	23	0	0	64
0	0	0	39	0	0	38
0	0	24	125	10	0	84
0	0	0	11	0	0	0
0	0	0	10	0	162	42
0	0	0	12	0	0	0
0	0	0	16	0	27	37
0	0	0	13	0	0	0
0	0	0	27	0	0	0
0	438	11	84	45	3084	48
0	0	0	10	0	905	0
0	0	0	11	10	498	0
0	0	0	18	0	0	0
0	0	0	18	0	0	249
0	0	0	22	0	0	0

0	0	0	10	0	28	13
0	0	0	14	0	63	170
0	0	0	25	0	0	0
0	0	0	16	0	22	0
11	0	178	49	0	33	3688
0	0	113	280	247	397	10031
0	0	0	10	63	0	0
0	0	0	26	0	216	126
0	0	0	36	0	37	0
0	0	0	35	0	10	52
0	0	0	38	0	0	0
0	0	0	12	10	0	0
0	0	0	91	55	0	45
0	0	0	11	0	0	0
0	0	0	11	0	0	0
0	0	0	88	0	0	0
0	0	0	101	0	0	0
0	0	0	95	0	0	0
14	0	0	14	114	0	166
0	0	0	11	0	0	0
0	0	0	12	0	0	0
0	0	0	41	0	0	0
0	0	0	98	0	0	0
0	0	0	155	72	0	0
0	0	0	67	14	0	0
20	0	0	10	998	0	15
0	0	0	51	0	0	0
0	0	0	54	0	0	0
15	0	0	46	187	0	10
0	0	0	54	0	0	0
0	0	0	43	0	0	0
0	0	0	63	465	0	0
0	0	0	10	0	0	0
110	117	0	177	420	0	120
0	0	0	98	0	0	24
0	0	0	44	0	0	0
0	0	0	10	0	0	0
0	0	0	50	0	11	0
0	0	0	24	0	0	0
0	0	0	82	0	0	0
0	0	0	10	163	0	0
0	53	0	122	0	36	24
0	0	0	41	0	0	0
0	0	0	38	10	0	0
0	0	0	10	0	0	0
11	0	126	111	39	0	0

0	0	0	230	0	0	0
0	0	0	23	0	0	0
0	0	0	10	0	0	0
0	0	0	36	37	0	0
0	0	0	182	0	0	0
0	0	0	10	41	0	0
0	0	0	11	0	0	0
0	0	0	57	78	0	0
0	0	0	21	0	0	0
0	0	0	28	0	0	0
0	0	0	130	0	0	0
0	0	0	15	75	0	0
0	0	0	195	466	0	0
0	0	0	62	0	0	0
0	0	0	57	204	0	0
0	0	0	34	45	0	0
0	0	0	51	174	0	0
0	0	0	342	234	0	0
0	0	0	77	789	0	0
0	0	0	37	0	0	0
0	0	0	26	0	0	0
0	0	0	60	42	0	0
0	0	0	24	25	0	0
0	0	0	109	55	11	0
0	10	0	131	55	0	11
0	0	0	60	26	0	0
0	0	0	332	58	49	10
0	0	0	60	0	13	0
0	0	0	187	65	24	0
0	0	0	29	0	0	0
0	0	0	68	0	0	68
0	0	0	10	39	0	0
0	0	10	33	0	0	56
0	0	0	21	39	0	15
0	0	0	53	0	0	0
0	0	0	17	0	0	0
0	0	0	21	0	0	0
0	0	0	119	132	13	0
0	0	0	69	203	0	0
0	0	0	18	0	0	0
0	0	0	69	88	41	0
0	0	0	84	64	0	0
0	0	0	94	0	0	0
0	0	0	11	0	0	0
0	0	0	31	0	0	0
0	0	0	10	0	0	0

10	0	0	11	0	13	0
0	0	0	35	53	170	0
0	0	0	43	0	0	0
0	0	0	11	0	0	0
0	0	0	28	0	31	0
0	0	0	15	173	0	0
0	0	0	56	0	0	0
0	0	0	13	0	0	0
0	0	0	247	32	78	10
0	14	13	60	491	0	164
0	0	0	50	701	0	0
0	61	0	40	0	0	96
0	0	0	27	0	0	0
0	0	0	89	0	0	0
0	0	0	14	0	0	0
0	0	0	73	0	0	0
17	0	11	72	375	0	105
0	0	0	18	205	0	0
0	0	0	76	62	0	0
0	0	0	32	84	0	0
0	0	0	12	158	23	0
0	0	12	20	0	0	0
0	0	0	46	0	0	0
0	0	0	12	0	0	0
0	0	0	11	0	0	0
101	33	54	10	24	0	13
0	0	0	14	0	0	0
0	0	11	30	0	0	12
0	0	0	85	0	0	0
0	0	0	43	0	0	0
0	0	0	28	0	0	0
0	0	0	353	0	0	0
0	0	0	109	0	0	0
0	0	0	16	0	0	0
0	0	0	85	0	0	0
0	0	0	14	0	0	0
70	0	0	10	60	0	32
611	96	259	35	160	21	139
0	0	0	12	0	0	0
94	38	81	12	42	0	23
0	0	0	12	0	0	0
0	0	0	13	0	0	63
0	0	0	36	0	0	0
0	0	0	11	0	0	0
0	0	0	40	0	0	0
0	0	0	31	0	11	13

0	0	0	11	0	0	0
0	0	0	11	0	0	0
20	0	10	10	64	0	30
10	0	0	21	0	0	54
0	0	0	22	31	0	0
10	0	0	10	13	0	0
0	0	0	22	0	0	0
111	235	0	33	1573	0	320
0	0	0	14	0	0	0
0	53	0	30	44	0	89
0	40	14	54	28	0	510
0	0	0	32	0	0	0
0	0	0	44	0	0	0
0	0	0	23	0	0	0
0	0	0	45	0	0	0
0	0	0	15	0	0	0
0	0	0	11	0	12	0
0	0	0	10	0	0	0
0	0	0	41	0	0	0
16	0	0	43	0	25	28
0	0	0	13	0	0	0
0	0	0	23	0	16	74
0	0	0	13	11	0	0
0	0	0	14	32	0	0
0	0	0	21	0	0	0
0	0	0	14	0	0	0
0	0	0	44	0	0	41
0	0	0	43	55	0	15
0	0	0	32	0	0	0
0	0	0	10	0	0	0
0	0	0	75	67	0	0
0	0	0	88	24	11	24
0	0	0	11	0	0	0
0	0	0	15	0	0	0
0	68	0	233	1137	225	283
0	216	0	15	0	252	10
0	0	0	20	0	0	0
0	0	0	10	0	36	0
0	14	0	211	2068	95	23
0	0	0	41	23	0	10
0	0	0	22	10	0	0
0	0	0	82	104	0	0
0	0	0	26	0	0	0
0	0	125	10	0	0	0
0	0	0	173	285	0	0
0	0	0	36	169	59	159

0	0	0	403	349	0	135
0	0	0	38	23	0	11
0	0	0	17	0	0	0
0	0	0	119	0	0	0
0	0	0	48	0	0	0
0	0	0	85	10	15	0
0	0	0	28	0	0	0
0	0	0	71	0	0	0
0	0	0	52	0	0	0
0	0	23	25	0	0	0
0	0	139	17	0	0	0
0	0	0	54	0	0	0
0	0	347	27	0	0	0
0	0	273	94	0	0	0
0	0	239	144	0	15	0
0	0	0	66	0	0	0
0	0	0	22	32	0	0
0	0	0	63	0	0	0
0	0	0	11	0	0	0
0	0	0	10	0	0	0
0	0	0	11	0	0	0
0	0	0	13	0	0	0
0	0	0	189	0	0	0
0	0	0	26	1386	0	0
0	0	0	87	104	0	74
63	39	0	164	498	0	46
0	0	0	21	36	0	0
0	0	0	11	219	0	0
0	0	0	29	0	0	0
0	0	0	216	0	29	0
0	0	0	18	0	0	0
0	0	0	25	108	0	0
0	0	0	47	0	0	0
0	0	0	37	0	0	0
0	0	0	24	0	0	12
0	0	0	71	13	0	11
0	0	0	44	0	0	0
0	0	0	11	112	0	0
0	0	0	13	65	0	0
0	0	0	14	0	0	0
35	77	0	260	1809	72	234
0	0	0	138	0	11	13
44	0	0	21	59	0	0
0	0	0	39	0	0	97
0	0	0	89	0	0	0
0	0	0	201	0	0	0

0	0	0	43	0	0	0
0	0	0	10	0	0	0
0	0	0	150	187	0	12
0	0	0	23	12	0	0
0	0	0	111	21	0	0
0	0	0	63	285	10	0
0	0	0	652	0	0	0
0	0	0	101	10	0	0
0	0	18	202	68	0	12
0	0	0	10	0	0	0
0	10	0	94	0	0	0
0	0	0	29	0	0	0
0	55	0	16	0	20	83
0	0	0	10	0	0	11
40	0	0	58	5185	0	38
0	0	0	44	239	0	0
29	29	0	14	100	0	90
0	0	0	61	2934	0	202
0	0	0	10	0	0	0
0	0	0	10	71	0	0
0	0	0	10	149	0	0
23	0	0	321	1219	15	26
0	0	0	16	0	0	0
0	0	0	117	0	0	0
0	0	0	11	0	0	0
0	0	0	10	0	0	0
0	0	0	26	0	0	0
116	27	67	157	391	28	244
11	0	0	12	20	0	0
0	0	0	58	26	0	0
31	0	0	285	208	13	0
0	0	0	65	188	0	0
0	0	0	133	163	0	0
0	0	0	180	0	0	0
0	0	0	257	0	0	0
0	0	0	107	0	0	0
0	0	0	87	0	0	0
0	0	0	49	63	0	32
0	0	0	11	0	99	76
0	0	0	10	0	0	0
0	0	0	27	210	0	62
0	0	0	97	47	30	0
0	0	0	11	48	0	0
0	0	0	433	111	0	0
0	0	0	226	0	51	0
0	0	0	19	10	0	0

0	0	0	454	137	25	0
0	0	0	31	0	0	0
0	0	0	39	0	11	0
0	0	0	122	0	0	0
0	0	0	22	0	34	0
0	0	0	112	0	12	0
0	0	0	34	0	0	24
0	0	0	34	0	0	0
0	0	0	54	111	0	74
48	0	0	39	104	0	23
0	0	0	51	10	0	0
0	0	0	44	48	0	42
0	0	0	17	21	0	0
0	0	0	58	0	0	0
0	0	0	47	32	0	46
0	0	0	160	0	0	0
0	0	0	66	0	0	0
0	0	0	13	0	0	0
0	0	0	0	163	0	288
25	0	0	0	34	0	0
0	0	0	0	48	0	0
0	0	0	0	95	0	24
11	0	0	0	0	0	163
0	0	0	0	0	0	0
0	0	0	0	0	28	0
0	0	0	0	82	0	0
0	0	0	0	14	0	0
0	0	0	0	180	0	0
0	0	0	0	0	0	0
0	0	0	0	0	0	0
0	0	0	0	10	0	0
0	0	0	0	262	0	0
26	0	0	0	0	50	52
0	0	0	0	0	0	0
0	0	0	0	0	0	0
0	0	27	0	62	0	68
0	0	0	0	29	0	12
11	0	0	0	0	15	0
0	0	0	0	0	0	0
0	0	0	0	61	0	0
0	0	0	0	299	0	0
0	0	0	0	74	0	0
0	0	0	0	31	0	0
0	0	0	0	86	0	0
0	0	0	0	345	11	26
0	17	0	0	10	31	0

0	0	0	0	75	0	0
0	0	0	0	109	0	0
0	0	0	0	1292	0	38
93	17	41	0	375	0	0
0	66	0	0	283	65	946
0	0	0	0	963	0	0
0	0	0	0	0	0	0
0	0	0	0	137	0	0
0	0	0	0	0	0	0
0	0	0	0	0	0	0
0	0	0	0	329	0	0
0	0	0	0	88	0	0
0	0	0	0	61	0	0
0	0	0	0	0	0	0
0	0	0	0	22	0	10
0	0	0	0	0	0	0
0	0	0	0	0	0	0
0	0	0	0	13	0	0
0	0	0	0	0	21	0
0	0	0	0	0	0	0
0	0	0	0	10	0	0
0	0	0	0	0	0	0
0	0	0	0	25	0	23
0	0	0	0	0	23	0
0	0	0	0	0	105	20
0	0	0	0	14	0	293
0	29	0	0	42	0	51
0	0	0	0	0	0	0
0	0	0	0	0	0	0
0	0	0	0	0	11	0
0	0	0	0	0	0	0
0	0	0	0	10	0	0
0	0	0	0	0	0	0
0	0	0	0	0	55	378
0	55	0	0	0	84	0
0	0	0	0	0	242	0
0	0	0	0	0	45	0
0	0	0	0	0	0	0
0	0	0	0	0	0	37
0	0	0	0	0	0	12
0	0	11	0	0	0	1473
0	0	0	0	0	0	0
0	0	0	0	0	0	0
0	0	0	0	0	19	0
0	0	0	0	0	21	0
0	0	0	0	0	0	0

0	11	0	0	0	0	335
0	0	0	0	0	326	24
0	0	0	0	0	0	0
0	23	0	0	0	12	287
0	0	0	0	0	45	181
0	0	0	0	0	0	0
0	0	0	0	0	0	0
0	0	0	0	0	0	0
0	0	0	0	0	0	0
0	0	0	0	0	0	0
0	0	0	0	0	0	0
0	0	0	0	0	0	0
0	0	0	0	11	0	0
0	0	0	0	0	0	0
0	0	0	0	0	0	0
0	0	0	0	0	0	0
0	0	0	0	0	0	0
0	0	0	0	0	0	11
11	0	0	0	16	0	77
0	0	0	0	0	0	195
0	0	0	0	0	0	0
0	0	0	0	45	0	0
0	0	0	0	0	0	0
0	0	0	0	0	0	0
0	0	0	0	0	0	0
0	0	0	0	0	15	57
26	0	0	0	0	0	118
45	0	11	0	0	0	42
0	0	0	0	0	0	14
115	76	18	0	0	159	476
0	0	0	0	0	0	0
0	0	0	0	0	0	0
0	0	0	0	0	0	0
0	0	0	0	92	41	39
0	0	0	0	0	0	73
0	0	0	0	26	0	0
0	0	0	0	0	0	0
0	0	0	0	0	0	0
0	0	0	0	0	0	0
0	0	0	0	0	0	0
0	0	0	0	0	0	0
0	0	0	0	0	0	0
0	0	0	0	0	0	0
0	0	0	0	0	0	0
0	0	0	0	0	0	0
0	0	0	0	0	0	0

0	0	0	0	0	0	0
0	0	0	0	0	0	0
0	0	0	0	0	0	0
0	0	0	0	0	0	0
0	0	0	0	0	0	0
0	0	0	0	0	0	0
0	0	0	0	0	0	0
0	0	0	0	0	0	0
0	0	0	0	0	0	0
0	0	0	0	0	0	0
0	0	0	0	0	0	0
0	0	0	0	0	16	11
0	0	0	0	0	0	0
0	0	0	0	0	0	355
0	0	0	0	0	0	588
0	0	0	0	0	0	0
0	0	0	0	0	0	0
0	0	0	0	0	0	0
0	0	0	0	0	0	0
0	0	0	0	0	0	0
0	0	0	0	0	0	0
0	0	0	0	0	0	0
0	0	0	0	0	0	0
0	0	0	0	0	0	0
0	0	0	0	0	0	0
0	0	0	0	0	0	0
0	0	0	0	0	0	0
0	0	0	0	0	0	0
0	0	0	0	0	0	0
0	0	0	0	0	0	0
0	0	0	0	0	0	0
0	0	0	0	0	0	0
0	0	0	0	0	0	10
0	0	0	0	0	0	0
0	0	0	0	0	0	12
0	0	0	0	0	0	0
0	0	0	0	0	0	0
0	0	0	0	0	0	0
0	0	0	0	0	0	0
0	0	0	0	0	12	0
0	0	0	0	0	0	0
0	0	0	0	0	0	0
0	0	0	0	0	0	24
0	0	0	0	0	0	0
0	0	0	0	0	0	0
0	0	0	0	0	0	0

0	0	0	0	0	0	0
0	0	0	0	0	0	0
0	0	0	0	0	0	0
0	0	0	0	0	0	0
0	0	0	0	0	0	0
0	0	0	0	0	0	0
0	0	0	0	0	0	0
0	0	0	0	0	0	0
30	0	0	0	16	0	0
0	0	0	0	0	0	0
0	0	0	0	0	0	0
0	0	0	0	0	0	0
0	0	0	0	0	0	0
0	0	0	0	0	0	0
0	0	0	0	0	0	0
0	0	0	0	0	0	0
0	0	0	0	165	0	0
0	0	0	0	0	0	0
0	0	0	0	0	0	0
0	0	0	0	0	0	0
0	0	0	0	0	0	0
0	0	0	0	0	0	0
0	0	0	0	0	0	0
0	0	0	0	0	0	0
0	0	0	0	0	0	0
0	0	0	0	0	0	0
0	0	0	0	0	0	0
0	0	0	0	0	0	0
0	0	0	0	0	0	0
0	0	0	0	0	0	0
0	0	0	0	0	0	0
0	0	0	0	0	0	10
0	0	0	0	0	0	0
0	0	0	0	0	0	0
0	0	0	0	0	0	0
0	0	0	0	0	0	0
0	0	0	0	0	0	0
0	0	0	0	0	0	0
0	0	0	0	0	0	0
0	0	0	0	0	0	0
0	0	0	0	0	0	0
0	0	0	0	0	0	0
0	0	0	0	0	0	0
0	0	0	0	0	0	0
0	0	0	0	48	0	0
0	0	0	0	0	0	0

0	0	0	0	0	0	0
0	0	0	0	0	0	0
0	0	0	0	0	11	0
0	0	0	0	0	0	0
0	0	0	0	0	0	0
0	0	0	0	0	0	0
0	0	0	0	0	0	0
0	0	0	0	0	0	0
0	0	0	0	0	0	0
0	0	0	0	0	0	0
0	0	0	0	0	0	0
0	0	0	0	11	0	0
0	0	0	0	0	0	0
0	0	0	0	0	0	0
0	0	0	0	0	0	0
0	0	0	0	12	0	0
0	0	0	0	47	0	23
0	0	0	0	0	0	169
0	0	0	0	10	0	0
0	0	0	0	54	0	0
0	0	0	0	0	0	13
0	0	0	0	12	0	0
0	0	0	0	10	0	0
0	67	0	0	0	0	11
0	0	0	0	0	150	40
0	0	0	0	0	0	0
0	0	0	0	0	0	0
0	0	0	0	44	0	0
0	0	0	0	0	0	0
0	0	0	0	0	0	0
0	49	0	0	0	716	75
0	0	0	0	0	0	0
0	0	0	0	49	0	0
0	0	0	0	0	0	11
0	0	14	0	0	0	0
0	0	0	0	0	0	0
0	0	0	0	0	0	0
0	0	0	0	0	0	0
0	0	0	0	0	0	0
0	0	0	0	0	0	0
0	0	0	0	0	0	0
0	0	0	0	0	0	0
0	0	90	0	11	0	10
0	0	0	0	0	0	0
0	0	0	0	0	0	0
0	0	0	0	31	0	0

0	0	0	0	31	0	0
0	0	0	0	0	0	0
0	0	0	0	53	0	0
0	0	0	0	0	0	0
0	0	0	0	0	0	0
0	0	0	0	0	0	0
0	0	0	0	11	0	0
0	0	0	0	0	0	0
0	0	0	0	0	0	0
0	0	0	0	0	0	0
0	0	0	0	0	0	0
0	0	0	0	0	0	0
0	0	0	0	0	0	0
0	0	0	0	0	0	0
0	0	0	0	10	0	0
0	0	0	0	0	0	0
0	0	0	0	37	0	0
0	0	0	0	0	0	0
0	0	0	0	0	0	0
0	0	0	0	0	0	0
0	0	0	0	0	0	0
0	0	0	0	0	0	0
0	0	0	0	0	0	0
0	0	0	0	0	0	0
0	0	0	0	0	0	0
0	0	0	0	10	0	0
0	0	0	0	0	0	0
0	0	0	0	0	0	0
0	0	0	0	38	0	0
0	0	0	0	0	0	0
0	0	0	0	0	59	0
0	0	0	0	10	0	0
0	0	0	0	0	0	0
10	0	0	0	0	0	85
0	0	0	0	0	0	0
0	0	10	0	0	0	0
0	0	0	0	0	0	0
48	12	40	0	13	0	18
0	0	0	0	0	0	0
12	0	0	0	0	0	0
0	0	0	0	0	0	0
0	0	0	0	0	0	87
0	0	0	0	0	0	0
0	0	0	0	10	0	0
0	0	0	0	0	0	0
24	0	26	0	11	0	10

0	0	0	0	0	0	0
0	0	0	0	11	0	0
0	0	0	0	0	45	11
0	0	0	0	13	0	0
0	0	0	0	0	0	0
0	0	0	0	0	0	0
0	0	0	0	0	0	0
0	0	0	0	0	0	0
0	0	0	0	0	0	0
0	0	0	0	0	0	0
0	0	0	0	0	0	0
0	0	0	0	0	0	0
0	0	0	0	0	0	0
0	0	0	0	0	0	0
0	0	0	0	0	0	0
0	0	0	0	0	0	0
0	0	0	0	46	0	0
0	0	0	0	0	0	25
0	0	0	0	0	0	0
0	0	0	0	11	0	38
0	0	0	0	71	0	16
0	0	73	0	0	0	0
0	0	0	0	13	0	0
0	0	0	0	0	53	0
0	0	0	0	0	0	0
0	0	0	0	36	0	26
0	0	0	0	0	0	0
0	0	0	0	13	0	0
0	0	0	0	0	0	54
0	0	0	0	0	0	0
0	0	0	0	40	0	0
0	0	0	0	11	0	10
0	0	0	0	47	0	0
0	65	0	0	0	51	423
0	0	0	0	0	0	48
0	0	0	0	0	17	169
0	0	0	0	46	0	45
0	13	0	0	10	76	337
0	0	0	0	69	0	0
0	0	0	0	0	22	76
0	0	0	0	0	0	0
0	0	0	0	0	0	0
0	0	0	0	0	0	0
0	0	0	0	0	0	0
0	0	0	0	0	0	0
0	0	0	0	0	0	0

0	0	0	0	10	0	0
0	0	0	0	0	0	0
0	0	0	0	0	0	0
0	0	0	0	0	0	0
0	0	0	0	0	0	0
0	0	0	0	0	0	0
0	0	0	0	0	0	0
0	0	0	0	0	0	0
0	0	0	0	0	0	0
0	0	0	0	0	0	0
0	0	0	0	0	0	0
0	0	0	0	0	0	0
0	0	0	0	0	0	0
0	0	0	0	0	0	0
0	0	0	0	0	0	0
0	0	0	0	0	0	0
0	0	0	0	0	0	0
0	0	0	0	0	0	0
0	0	0	0	0	0	0
0	0	0	0	0	0	0
0	0	0	0	0	0	0
0	0	0	0	0	0	0
0	0	0	0	0	0	0
0	21	0	0	0	0	0
0	0	0	0	0	0	77
0	0	0	0	0	0	0
0	0	0	0	0	0	0
0	0	0	0	0	0	0
0	0	0	0	0	0	0
0	0	0	0	0	14	0
0	0	0	0	0	0	0
0	0	0	0	0	22	37
0	0	0	0	0	0	0
0	0	0	0	0	150	93
0	0	0	0	0	10	83
0	0	0	0	0	0	0
0	0	0	0	0	0	0
0	0	0	0	0	0	0
0	0	0	0	0	0	0
0	0	0	0	0	0	0
0	0	0	0	0	0	0
0	39	0	0	0	0	0
0	0	0	0	0	0	0
0	0	0	0	0	0	0
0	0	0	0	0	0	0
0	0	0	0	0	0	0

0	0	0	0	0	0	15
0	0	0	0	0	0	0
0	0	0	0	0	0	0
0	0	0	0	0	0	0
0	0	0	0	83	0	0
0	0	0	0	112	0	0
0	0	0	0	0	0	0
0	0	0	0	0	0	0
0	0	0	0	0	0	0
0	0	0	0	0	0	0
0	0	0	0	0	0	0
0	0	0	0	0	0	29
0	0	0	0	0	0	0
0	0	0	0	0	0	0
0	0	0	0	0	0	0
0	0	0	0	0	0	0
0	0	0	0	0	0	0
0	0	0	0	0	0	0
0	10	0	0	0	0	0
0	0	0	0	0	0	0
0	0	0	0	0	0	0
0	0	0	0	80	0	0
0	0	0	0	0	0	0
0	0	0	0	0	0	0
0	0	0	0	0	0	0
0	0	0	0	0	0	0
0	0	0	0	0	0	0
0	0	0	0	0	0	0
0	0	0	0	0	0	0
0	0	0	0	0	0	0
0	0	0	0	0	0	0
0	0	0	0	0	0	0
0	0	0	0	0	0	0
0	0	0	0	0	0	0
0	0	0	0	0	0	0
0	0	0	0	0	0	0
0	0	54	0	0	0	0
0	0	0	0	0	0	0
0	0	0	0	0	0	0
0	0	0	0	0	0	0
0	0	0	0	0	0	0
0	0	0	0	0	0	0
0	0	0	0	0	0	0
0	0	0	0	0	0	0
0	0	0	0	0	0	0
0	0	0	0	0	0	0

0	0	0	0	0	0	0
0	0	0	0	0	0	15
22	0	0	0	0	0	0
0	0	0	0	0	0	0
0	0	0	0	0	0	0
0	0	0	0	0	0	0
0	0	0	0	0	0	0
0	0	0	0	0	0	44
0	0	0	0	0	0	0
0	0	0	0	0	0	0
0	0	0	0	0	0	0
0	0	0	0	0	0	11
0	0	0	0	0	0	0
0	0	0	0	0	0	0
0	0	0	0	0	0	0
0	0	0	0	0	16	0
0	0	0	0	0	0	0
0	0	0	0	0	0	42
0	0	0	0	0	20	0
0	0	0	0	53	0	0
0	0	0	0	0	0	0
0	0	0	0	0	0	0
0	0	0	0	0	546	0
0	0	0	0	0	0	0
0	0	0	0	0	0	0
0	0	0	0	0	0	0
0	0	0	0	0	0	0
0	0	0	0	0	0	0
0	0	0	0	0	13	26
0	0	0	0	0	0	0
0	0	0	0	0	0	0
0	0	0	0	0	0	0
0	0	0	0	0	0	0
0	0	0	0	0	0	0
0	0	0	0	0	0	0
0	0	0	0	0	0	0
0	0	0	0	0	0	0
0	0	0	0	0	0	0
0	0	0	0	0	94	0
0	0	0	0	0	0	0
0	0	0	0	0	0	0
0	0	0	0	0	0	0
0	0	0	0	0	0	0
0	0	0	0	0	0	0
0	0	0	0	0	0	0
0	0	0	0	0	0	0

0	0	0	0	0	0	0
0	0	0	0	16	0	0
0	0	0	0	0	0	0
0	0	0	0	0	0	0
0	0	0	0	0	0	0
0	0	0	0	0	0	0
0	0	0	0	0	0	11
0	0	0	0	0	0	0
0	0	0	0	0	0	0
0	0	0	0	0	0	0
0	0	0	0	0	0	20
0	0	0	0	0	0	0
0	0	0	0	0	0	0
0	0	0	0	0	0	0
0	0	0	0	0	0	0
0	0	0	0	0	0	0
0	10	0	0	0	0	80
0	0	0	0	0	36	55
0	0	0	0	0	0	0
0	0	0	0	0	10	0
0	0	0	0	0	0	0
0	0	0	0	0	0	13
0	0	0	0	10	0	0
0	0	0	0	0	0	0
0	0	0	0	0	0	29
0	0	0	0	0	0	0
0	48	0	0	0	0	645
0	0	0	0	12	0	0
0	0	0	0	0	11	0
0	0	0	0	0	0	0
0	0	0	0	0	0	0
0	0	0	0	0	0	0
0	0	0	0	0	0	0
0	0	0	0	0	0	0
0	0	0	0	0	0	0
0	0	0	0	0	0	0
0	0	0	0	0	0	0
0	0	0	0	16	0	0
0	0	0	0	0	0	15
0	0	0	0	0	10	0
0	10	0	0	0	0	0
0	0	0	0	0	0	35
0	0	0	0	0	0	0
0	68	0	0	0	0	0
0	153	0	0	0	24	14
0	11	0	0	0	0	17

0	106	0	0	0	0	0
0	0	0	0	13	0	0
0	0	0	0	0	0	0
0	0	0	0	0	0	0
0	0	0	0	10	0	0
0	0	0	0	0	0	0
0	0	0	0	55	0	0
0	0	0	0	0	0	0
0	0	0	0	0	0	94
0	0	0	0	40	0	0
0	0	0	0	0	0	0
0	0	0	0	0	0	0
0	0	0	0	0	0	0
0	0	0	0	0	0	0
0	0	0	0	0	0	0
0	0	0	0	0	0	0
0	0	0	0	0	0	0
0	0	0	0	0	0	29
0	0	0	0	0	0	43
0	0	0	0	0	0	0
0	0	0	0	0	0	0
0	0	0	0	0	0	30
0	0	0	0	0	0	0
0	0	0	0	0	0	0
0	0	0	0	0	0	0
0	0	0	0	0	0	0
0	0	0	0	0	0	0
0	0	0	0	0	0	0
0	0	0	0	0	0	0
0	0	0	0	0	0	0
0	0	0	0	0	0	0
0	0	0	0	0	0	0
0	0	0	0	0	0	0
0	0	0	0	0	0	0
0	0	0	0	0	0	0
0	0	0	0	0	0	0
0	0	0	0	0	0	0
0	0	0	0	0	0	0
0	0	0	0	0	0	0
0	0	0	0	0	0	0
0	0	0	0	0	0	58
0	0	0	0	0	0	0
0	0	0	0	0	0	0
0	0	0	0	0	0	0
0	0	0	0	0	0	0
0	0	0	0	0	0	0

0	0	0	0	0	0	40
0	0	0	0	0	0	0
0	0	0	0	0	0	0
0	0	0	0	0	0	0
0	0	0	0	0	0	0
0	0	0	0	0	60	0
0	0	0	0	0	0	0
0	0	0	0	0	0	0
0	0	0	0	0	0	0
0	0	0	0	0	0	0
0	0	0	0	0	0	0
0	0	0	0	0	0	0
0	0	0	0	0	0	0
0	0	0	0	0	0	0
0	0	0	0	0	0	0
0	0	0	0	0	0	0
0	0	0	0	0	0	0
0	0	0	0	0	0	0
0	0	0	0	0	0	0
0	0	0	0	0	0	0
0	0	0	0	0	0	0
0	0	0	0	10	0	0
0	0	0	0	0	0	0
0	0	0	0	0	0	0
0	0	0	0	0	0	0
0	0	0	0	0	0	0
0	0	0	0	0	0	0
0	0	0	0	0	0	0
0	0	0	0	0	0	0
0	0	0	0	0	0	0
0	0	0	0	0	0	0
0	0	0	0	0	0	0
0	0	0	0	0	0	12
0	0	0	0	0	0	0
0	0	0	0	0	0	0
0	0	0	0	0	0	0
0	0	0	0	0	0	0
0	0	0	0	0	0	0
0	0	0	0	0	0	0
0	0	0	0	0	0	0
0	0	0	0	0	0	0
0	0	0	0	11	0	0
0	0	0	0	0	0	0
0	0	0	0	0	0	0
0	0	0	0	0	0	0
0	0	0	0	0	0	0

0	0	0	0	0	0	0
40	0	0	0	0	0	0
120	0	0	0	0	0	0
0	0	0	0	0	0	0
0	0	0	0	0	0	0
0	0	0	0	0	0	0
0	0	0	0	0	0	0
0	0	0	0	0	0	0
0	0	0	0	0	0	11
0	0	0	0	0	0	15
0	0	0	0	0	0	0
0	0	0	0	0	0	0
0	39	0	0	0	0	0
0	0	0	0	0	0	0
0	0	0	0	0	0	0
0	0	0	0	0	0	0
0	0	0	0	0	0	0
0	0	0	0	0	0	0
0	0	0	0	0	0	0
0	0	0	0	0	0	0
0	0	0	0	0	0	0
0	0	0	0	0	0	0
0	0	0	0	0	0	0
0	0	0	0	0	0	0
0	0	0	0	0	0	0
0	0	0	0	0	0	0
0	0	0	0	0	0	0
0	0	0	0	0	0	0
0	0	0	0	0	0	30
0	0	0	0	0	0	0
0	0	0	0	0	0	0
0	0	0	0	0	0	10
0	0	0	0	0	0	0
0	0	0	0	0	14	0
0	0	0	0	0	0	0
0	0	0	0	0	0	0
0	0	0	0	0	0	0
0	0	0	0	0	0	0
0	0	0	0	0	0	0
0	0	0	0	0	0	0
0	0	0	0	46	0	0
0	0	0	0	0	0	0
0	0	0	0	0	0	0
0	0	0	0	0	0	18
0	0	0	0	0	0	0
0	0	0	0	0	0	0

0	0	0	0	0	0	0
0	0	0	0	0	0	0
0	0	0	0	10	0	0
0	0	0	0	25	0	0
0	0	0	0	10	0	0
0	0	0	0	15	0	0
0	0	0	0	23	0	0
0	0	0	0	10	0	0
0	0	0	0	11	0	0
0	0	0	0	12	0	0
0	0	0	0	10	0	0
0	0	0	0	11	0	0
0	0	0	0	27	0	0
0	0	0	0	27	0	0
0	0	0	0	10	0	0
0	0	0	0	12	0	0
0	0	0	0	25	0	0
0	0	0	0	44	0	0
0	0	0	0	68	0	0
0	0	0	0	17	0	0
0	0	0	0	28	0	0
0	0	0	0	55	0	0
0	0	0	0	25	0	0
0	0	0	0	12	0	0
0	0	0	0	10	0	0
0	0	0	0	12	0	0
0	0	0	0	55	0	0
0	0	0	0	38	0	0
0	0	0	0	18	0	0
0	0	0	0	29	0	0
0	0	0	0	15	0	0
0	0	0	0	55	0	0
0	0	0	0	10	0	0
0	0	0	0	11	0	0
0	0	0	0	21	0	0
0	0	0	0	10	0	0
0	0	0	0	0	0	0
0	0	0	0	0	0	0
0	0	0	0	0	0	0
0	0	0	0	0	0	0
0	0	0	0	0	0	12
0	0	0	0	0	0	0
0	0	0	0	0	0	0
0	0	0	0	0	0	0
0	0	0	0	0	0	0
0	0	0	0	0	0	0

0	0	0	0	0	0	0
0	0	0	0	0	0	0
0	0	0	0	0	0	0
0	0	0	0	0	0	0
0	0	0	0	0	0	0
0	0	0	0	0	0	0
0	0	0	0	0	0	0
0	0	0	0	0	0	0
0	0	0	0	0	0	0
0	0	0	0	0	0	0
0	0	0	0	0	0	0
0	0	0	0	0	0	0
0	0	0	0	0	0	0
0	0	0	0	0	43	0
0	0	0	0	0	0	0
0	0	0	0	0	0	0
0	0	0	0	0	0	51
0	0	0	0	0	0	0
0	0	0	0	0	0	0
0	0	0	0	0	0	0
0	0	0	0	0	0	0
0	0	0	0	0	0	0
0	0	0	0	0	0	0
0	0	0	0	0	0	0
0	0	0	0	0	0	0
0	0	0	0	0	0	0
0	0	0	0	0	0	0
0	0	0	0	0	0	0
0	0	0	0	0	0	0
0	0	0	0	0	0	0
0	0	0	0	0	0	0
0	0	0	0	0	0	0
0	0	0	0	0	0	0
0	0	0	0	0	0	0
0	0	0	0	0	0	0
0	0	0	0	0	0	11
0	0	0	0	0	0	0
0	0	0	0	0	0	43
0	0	0	0	0	0	0
0	0	0	0	0	0	0
0	0	0	0	0	0	0
0	0	0	0	0	0	0
0	0	0	0	0	0	0
0	0	0	0	0	0	0
0	0	0	0	0	0	0
0	0	0	0	0	0	0

0	0	0	0	0	0	0
0	0	0	0	0	0	0
0	0	0	0	0	0	0
0	0	0	0	0	0	0
0	0	0	0	0	0	0
0	0	0	0	0	0	0
0	0	0	0	0	0	0
0	0	0	0	0	0	0
0	0	0	0	0	0	0
0	0	0	0	0	0	0
0	0	0	0	0	0	0
0	0	0	0	0	0	0
0	0	0	0	0	0	0
0	0	0	0	0	0	0
0	0	0	0	0	0	0
0	0	0	0	0	0	0
0	0	0	0	0	0	0
0	0	0	0	0	0	0
0	0	0	0	0	0	0
0	0	0	0	0	0	0
0	0	0	0	0	20	0
0	0	0	0	0	0	0
0	0	0	0	0	0	0
0	0	0	0	0	0	0
0	0	0	0	0	0	0
0	0	0	0	0	0	0
0	0	0	0	0	0	0
0	0	0	0	0	0	0
0	0	0	0	0	0	0
0	0	0	0	0	0	0
0	0	0	0	0	0	0
0	0	0	0	0	0	0
0	0	0	0	0	0	0
0	0	0	0	0	0	0
0	0	0	0	0	0	0
0	0	0	0	0	0	0
0	0	0	0	0	0	0
0	0	0	0	0	0	0
0	0	0	0	0	0	0
0	0	0	0	0	0	0
0	0	0	0	0	0	0
0	0	0	0	0	0	0
0	0	0	0	0	0	0
0	0	0	0	0	0	0
0	0	0	0	0	0	0
0	0	0	0	0	0	0

0	0	0	0	0	0	0
0	0	0	0	0	0	0
0	0	0	0	0	0	18
0	0	0	0	0	0	0
0	0	0	0	0	0	0
0	0	0	0	0	0	0
0	0	0	0	0	0	0
0	0	0	0	0	0	0
0	0	0	0	0	0	0
0	0	0	0	0	0	0
0	0	0	0	0	0	0
0	0	0	0	0	0	0
0	0	0	0	0	0	0
0	0	0	0	0	0	0
0	0	0	0	0	0	0
0	0	0	0	0	0	0
0	0	0	0	0	0	0
0	0	0	0	0	0	0
0	0	0	0	0	0	0
0	0	0	0	0	0	0
0	0	0	0	0	0	0
0	0	0	0	0	0	0
0	0	0	0	0	0	0
0	0	0	0	0	0	0
0	0	0	0	0	0	0
0	0	0	0	0	0	0
0	0	0	0	0	0	0
0	0	0	0	0	0	0
0	0	0	0	0	0	0
0	0	0	0	0	0	0
0	0	0	0	0	0	0
0	0	0	0	0	0	0
0	0	0	0	0	0	0
0	0	0	0	0	0	0
0	0	0	0	0	0	0
0	0	0	0	0	0	0
0	0	0	0	0	0	0
0	0	0	0	0	0	0
0	0	0	0	0	0	0
0	0	0	0	0	0	0
0	0	0	0	0	0	0
0	0	0	0	0	0	0
0	0	0	0	0	0	0
0	0	0	0	0	0	0
0	0	0	0	0	0	14
0	0	0	0	0	0	0

0	0	0	0	0	0	43
0	0	0	0	0	26	51
0	0	0	0	0	0	0
0	0	0	0	0	0	0
0	0	0	0	0	0	0
0	0	0	0	0	0	0
0	0	0	0	0	0	0
0	0	0	0	0	0	0
0	0	0	0	0	0	0
0	0	0	0	0	0	0
0	0	0	0	0	0	0
0	0	0	0	0	0	0
0	0	0	0	0	0	0
0	0	0	0	0	14	10
0	0	0	0	0	0	0
0	0	0	0	0	0	0
0	0	0	0	0	0	0
0	0	0	0	0	0	0
0	0	0	0	0	0	0
0	0	0	0	0	0	0
0	0	0	0	0	0	0
0	0	0	0	0	0	0
0	0	0	0	0	0	0
0	0	0	0	0	0	0
0	0	0	0	0	0	11
0	0	0	0	0	0	0
0	0	0	0	0	0	10
0	0	0	0	0	0	0
0	0	0	0	0	0	0
0	0	0	0	0	0	0
0	0	0	0	0	0	13
0	0	0	0	0	0	0
0	0	0	0	0	0	0
0	0	0	0	0	0	16
0	0	0	0	0	0	0
0	0	0	0	0	0	0
0	0	0	0	0	0	0
0	0	0	0	0	0	0
0	0	0	0	0	0	0
0	0	0	0	0	0	0
0	0	0	0	0	0	0
0	0	0	0	0	0	0
0	0	0	0	0	0	0
0	0	0	0	0	0	0
0	0	0	0	0	0	0
0	0	0	0	0	0	0

0	0	0	0	0	0	0
0	0	0	0	0	0	0
0	0	0	0	0	0	0
0	0	0	0	0	0	0
0	0	0	0	0	0	0
0	0	0	0	0	0	11
0	0	0	0	0	0	0
0	0	0	0	0	0	0
0	0	0	0	0	0	0
0	0	0	0	0	0	0
0	0	0	0	0	0	0
0	0	0	0	0	11	0
0	0	0	0	0	0	0
0	0	0	0	0	0	0
0	0	0	0	0	0	0
0	0	0	0	0	0	0
0	0	0	0	0	0	0
0	0	0	0	0	0	0
0	0	0	0	0	0	0
0	0	0	0	0	0	11
0	0	0	0	0	0	17
0	0	0	0	0	0	0
0	0	0	0	0	0	0
0	0	0	0	0	0	0
0	0	60	0	0	0	0
0	0	23	0	0	0	0
0	0	110	0	0	0	0
0	0	39	0	0	0	0
0	0	22	0	0	0	0
0	0	0	0	0	0	0
0	0	0	0	0	0	0
0	0	0	0	0	0	0
0	0	0	0	0	0	0
0	0	0	0	0	0	0
0	0	0	0	0	0	0
0	0	0	0	0	0	0
0	0	0	0	0	0	0
0	0	0	0	0	0	0
0	0	0	0	0	0	0
0	0	0	0	0	0	0
0	0	0	0	0	0	0
0	0	0	0	0	0	0
0	0	0	0	0	0	0
0	0	0	0	0	0	0
0	0	0	0	0	0	0
0	0	0	0	0	0	0

0	0	0	0	0	0	0
0	0	0	0	0	0	0
0	0	0	0	0	0	13
0	0	0	0	0	0	89
0	0	0	0	0	0	0
0	0	0	0	0	0	0
0	0	0	0	0	0	0
0	0	0	0	0	10	0
0	0	0	0	0	0	0
0	0	0	0	0	0	0
0	0	0	0	0	0	0
0	0	0	0	0	0	0
0	0	0	0	0	0	0
0	0	0	0	0	0	0
0	0	0	0	0	0	0
0	0	0	0	0	89	0
0	0	0	0	0	0	0
0	0	0	0	0	0	0
0	0	0	0	0	0	0
0	0	0	0	0	0	0
0	0	0	0	0	0	0
0	0	0	0	0	0	0
0	0	0	0	0	10	0
0	0	0	0	0	0	0
0	0	0	0	0	0	0
0	0	0	0	0	0	0
0	0	0	0	0	0	0
0	0	0	0	0	0	0
0	0	0	0	0	0	0
0	0	0	0	0	0	0
0	0	0	0	0	0	0
0	0	0	0	0	0	0
0	0	0	0	0	28	0
0	0	0	0	0	0	0
0	0	0	0	0	0	0
0	0	0	0	0	0	0
0	0	0	0	0	0	0
0	0	0	0	0	0	0
0	0	0	0	0	0	0
0	0	0	0	0	0	0
0	0	0	0	0	13	0
0	0	0	0	0	0	0
0	0	0	0	0	0	0
0	0	0	0	0	0	0
0	0	0	0	0	0	0
0	0	0	0	0	0	0

0	0	0	0	0	0	0
0	0	0	0	0	0	0
0	0	0	0	0	0	0
0	0	0	0	0	0	0
0	0	0	0	0	0	0
0	0	0	0	0	0	0
0	0	0	0	0	0	0
0	0	0	0	0	0	0
0	0	0	0	0	0	0
0	0	0	0	0	0	0
0	0	0	0	0	0	0
0	0	0	0	0	0	0
0	0	0	0	0	0	0
0	0	0	0	0	0	0
0	0	0	0	0	0	0
0	0	0	0	0	0	0
0	0	0	0	0	0	0
0	0	0	0	0	0	0
0	0	0	0	0	0	0
0	0	0	0	0	0	0
0	0	0	0	0	0	0
0	0	0	0	0	0	0
0	0	0	0	0	0	0
0	0	0	0	0	0	0
0	0	0	0	0	0	0
0	0	0	0	0	0	0
0	0	0	0	0	0	0
0	0	0	0	0	0	0
0	0	0	0	0	0	15
0	0	0	0	0	0	37
0	0	0	0	0	0	48
0	0	0	0	0	0	16
0	0	0	0	0	0	0
0	0	0	0	0	0	58
0	0	0	0	0	0	0
0	0	0	0	0	0	36
0	0	0	0	0	0	67
0	132	0	0	0	0	14
0	272	0	0	0	10	0
0	32	0	0	0	0	0
0	10	0	0	0	0	0
0	0	0	0	0	0	0
0	0	0	0	0	0	0
0	0	0	0	0	0	0
0	0	0	0	0	0	0
0	0	0	0	0	0	0

0	0	0	0	0	0	0
0	0	0	0	0	0	0
0	0	0	0	0	0	0
0	0	0	0	0	0	0
0	0	0	0	0	0	0
0	0	0	0	0	0	0
0	0	0	0	0	0	0
0	0	0	0	0	0	0
0	0	0	0	0	0	0
0	0	0	0	0	0	0
0	0	0	0	0	0	0
0	0	0	0	0	0	0
0	0	0	0	0	0	0
0	0	0	0	0	0	0
0	0	0	0	0	0	0
0	0	0	0	0	0	0
0	0	0	0	0	0	0
0	0	0	0	0	0	0
0	0	0	0	0	0	0
0	0	0	0	0	0	0
0	0	0	0	0	0	0
0	0	0	0	0	0	0
0	0	0	0	0	0	0
0	0	0	0	0	0	0
0	0	0	0	0	0	0
0	0	0	0	0	0	0
0	0	0	0	0	0	0
0	0	0	0	0	0	0
0	0	0	0	0	0	0
0	0	0	0	0	0	0
0	0	0	0	0	0	0
0	0	0	0	0	0	0
0	0	0	0	0	0	0
0	0	0	0	0	0	0
0	0	0	0	0	0	0
0	0	0	0	0	0	0
0	0	0	0	0	0	0
0	0	0	0	0	0	0
0	0	0	0	0	37	0
0	0	0	0	0	0	0
0	0	0	0	0	0	0
0	0	0	0	0	0	0
0	0	0	0	0	16	0
0	0	0	0	0	0	0
0	0	0	0	0	10	0
0	0	0	0	0	0	0

0	0	0	0	0	0	0
0	0	0	0	0	0	0
0	0	0	0	0	0	0
0	0	0	0	0	0	0
0	0	0	0	0	0	0
0	0	0	0	0	0	0
0	0	0	0	0	0	0
0	0	0	0	0	0	0
0	0	0	0	0	0	0
0	0	0	0	0	0	0
0	0	0	0	0	0	0
0	0	0	0	0	0	0
0	0	0	0	0	0	0
0	0	0	0	0	0	0
0	0	0	0	0	0	0
0	0	0	0	0	0	0
0	0	0	0	0	0	0
0	0	0	0	0	0	0
0	0	0	0	0	0	0
0	0	0	0	0	0	0
0	0	0	0	0	0	13
0	0	0	0	0	0	0
0	0	0	0	0	0	36
0	0	0	0	0	0	0
0	0	0	0	0	0	0
0	0	0	0	0	0	0
0	0	0	0	0	0	0
0	0	0	0	0	0	0
0	0	0	0	0	0	0
0	0	0	0	0	0	0
0	0	0	0	0	0	0
0	0	0	0	0	0	0
0	0	0	0	0	0	0
0	0	0	0	0	0	0
0	0	0	0	0	0	0
0	0	0	0	0	0	0
0	0	0	0	0	0	0
0	0	0	0	0	0	0
0	0	0	0	0	0	0
0	0	0	0	0	0	0
0	0	0	0	0	0	0
0	0	0	0	0	0	0
0	0	0	0	0	0	0
0	0	0	0	0	0	0
0	0	0	0	0	0	0
0	0	0	0	0	0	0

0	0	0	0	0	0	0
0	0	0	0	0	0	0
0	0	0	0	0	0	0
0	0	0	0	0	0	0
0	0	0	0	0	0	0
0	0	0	0	0	0	0
0	0	0	0	0	0	0
0	0	0	0	0	0	0
0	0	0	0	0	0	0
0	0	0	0	0	0	0
0	0	0	0	0	0	0
0	0	0	0	0	0	0
0	0	0	0	0	0	0
0	0	0	0	0	0	0
0	0	0	0	0	0	0
0	0	0	0	0	0	0
0	0	0	0	0	0	0
0	0	0	0	0	0	0
0	0	0	0	0	0	0
0	0	0	0	0	0	0
0	0	0	0	0	0	0
0	0	0	0	0	0	0
0	0	0	0	0	0	0
0	0	0	0	0	0	0
0	0	0	0	0	0	0
0	0	0	0	0	0	0
0	0	0	0	0	0	0
0	0	0	0	0	0	0
0	0	0	0	0	0	0
0	0	0	0	0	0	0
0	0	0	0	0	0	0
0	0	0	0	0	0	0
0	0	0	0	0	0	0
0	0	0	0	0	0	0
0	0	0	0	0	0	0
0	0	0	0	0	0	0
0	0	0	0	0	0	0
0	0	0	0	0	0	0
0	0	0	0	0	0	0
0	0	0	0	0	0	0
0	0	0	0	0	0	0
0	0	0	0	0	0	0
0	0	0	0	0	0	0
0	0	0	0	0	0	0
0	0	0	0	0	0	0
0	0	0	0	0	0	13

0	0	0	0	0	0	0
0	0	0	0	0	0	0
0	0	0	0	0	0	0
0	0	0	0	0	0	0
0	0	0	0	0	0	0
0	0	0	0	0	0	0
0	0	0	0	0	0	0
0	0	0	0	0	0	0
0	0	0	0	0	0	0
0	0	0	0	0	0	0
0	0	0	0	0	0	0
0	0	0	0	0	0	0
0	0	0	0	0	0	0
0	0	0	0	0	0	0
0	0	0	0	0	0	0
0	0	0	0	0	0	0
0	0	0	0	0	0	0
0	0	0	0	0	0	0
0	0	0	0	0	0	0
0	0	0	0	0	0	0
0	0	0	0	0	0	0
0	0	0	0	0	0	0
0	0	0	0	0	0	0
0	0	0	0	0	0	0
0	0	0	0	0	0	0
0	0	0	0	0	0	12
0	0	0	0	0	0	72
0	0	0	0	0	0	11
0	0	0	0	0	0	28
0	0	0	0	0	0	15
0	0	0	0	0	0	10
0	0	0	0	0	0	0
0	0	0	0	0	0	0
0	0	0	0	0	11	0
0	0	0	0	0	0	0
0	0	0	0	0	0	0
0	0	0	0	0	0	0
0	0	0	0	0	0	0
0	0	0	0	0	0	0
0	0	0	0	0	0	0
0	0	0	0	0	0	0
0	0	0	0	0	0	0
0	0	0	0	0	0	0
0	0	0	0	0	0	0
0	0	0	0	0	0	0
0	0	0	0	0	0	0

0	0	0	0	0	0	0
0	0	0	0	0	0	0
0	0	0	0	0	0	0
0	0	0	0	0	0	0
0	0	0	0	0	0	0
0	0	0	0	0	0	0
0	0	0	0	0	0	0
0	0	0	0	0	0	0
0	0	0	0	0	0	0
0	0	0	0	0	0	0
0	0	0	0	0	0	0
0	0	0	0	0	0	0
0	0	0	0	0	15	0
0	0	0	0	0	25	0
0	0	0	0	0	12	0
0	0	0	0	0	10	0
0	0	0	0	0	30	0
0	0	0	0	0	14	0
0	0	0	0	0	11	0
0	0	0	0	0	10	0
0	0	0	0	0	0	0
0	0	0	0	0	0	0
0	0	0	0	0	0	0
0	0	0	0	0	0	0
0	0	0	0	0	0	0
0	0	0	0	0	0	0
0	0	0	0	0	0	0
0	0	0	0	0	0	0
0	0	0	0	0	0	0
0	0	0	0	0	0	0
0	0	0	0	0	0	0
0	0	0	0	0	0	0
0	0	0	0	0	0	0
0	0	0	0	0	0	0
0	0	0	0	0	0	0
0	0	0	0	0	0	0
0	0	0	0	0	0	0
0	0	0	0	0	0	0
0	0	0	0	0	0	0
0	0	0	0	0	0	0
0	0	0	0	0	0	0
0	0	0	0	0	0	0
0	0	0	0	0	0	0
0	0	0	0	0	0	0
0	0	0	0	0	0	0
0	0	0	0	0	0	0

0	0	0	0	0	0	0
0	0	0	0	0	0	0
0	0	0	0	0	0	0
0	0	0	0	0	0	0
0	0	0	0	0	0	0
0	0	0	0	0	0	0
0	0	0	0	0	0	0
0	0	0	0	0	0	0
0	0	0	0	0	0	0
0	0	0	0	0	0	0
0	0	0	0	0	0	0
0	0	0	0	0	0	0
0	0	0	0	0	0	0
0	0	0	0	0	0	0
0	0	0	0	0	0	0
0	0	0	0	0	0	0
0	0	0	0	0	0	0
0	0	0	0	0	0	0
0	0	0	0	0	0	0
0	0	0	0	0	0	0
0	0	0	0	0	0	0
0	0	0	0	0	0	0
0	0	0	0	0	0	0
0	0	0	0	0	0	0
0	0	0	0	0	0	0
0	0	0	0	0	0	0
0	0	0	0	0	0	0
0	0	0	0	0	0	0
0	0	0	0	0	0	0
0	0	0	0	0	0	0
0	0	0	0	0	0	0
0	0	0	0	0	0	0
0	0	0	0	0	0	0
0	0	0	0	0	0	0
0	0	0	0	0	0	0
0	0	0	0	0	0	0
0	0	0	0	0	0	0
0	0	0	0	0	0	0
0	0	0	0	0	0	0
0	0	0	0	0	0	0
0	0	0	0	0	0	0
0	0	0	0	0	0	0
0	0	0	0	0	0	0
0	0	0	0	0	0	0
0	0	0	0	0	0	0
0	0	0	0	0	0	0

0	0	0	0	0	0	0
0	0	0	0	0	0	0
0	0	0	0	0	0	0
0	0	0	0	0	0	0
0	0	0	0	0	0	0
0	0	0	0	0	0	0
0	0	0	0	0	0	0
0	0	0	0	0	0	0
0	0	0	0	0	0	0
0	0	0	0	0	0	0
0	0	0	0	0	0	0
0	0	0	0	0	0	0
0	0	0	0	0	0	0
0	0	0	0	0	0	0
0	0	0	0	0	0	0
0	0	0	0	0	0	0
0	0	0	0	0	0	0
0	0	0	0	0	0	0
0	0	0	0	0	0	0
0	0	0	0	0	0	0
0	0	0	0	0	0	0
0	0	0	0	0	0	0
0	0	0	0	0	0	0

						Pr
PH_242	PH_249	PH_273	PH_280	PH_282	PH_283	PH_302
0	0	0	27	46	11	0
0	0	0	29	65	74	0
116	81	0	133	385	607	10
43	10	0	123	232	271	22
28	0	0	155	96	144	23
195	0	0	0	0	10	28
61	0	0	12	66	168	0
0	0	0	17	58	74	0
0	0	11	16	162	84	0
60	114	71	196	144	265	14
0	38	0	0	137	87	10
0	0	0	0	17	71	0
0	0	0	59	28	35	0
0	0	0	10	29	10	0
0	15	0	0	64	94	0
0	0	0	0	0	41	0
355	183	42	394	354	208	401
43	0	0	0	0	13	0
0	18	0	21	11	12	35
0	0	25	256	13	17	15
0	0	0	172	0	55	10
0	0	0	76	24	11	34
0	0	0	195	0	22	0
0	0	0	0	0	13	0
332	366	278	302	919	272	159
59	174	145	95	372	64	171
14	113	0	53	183	84	62
14	187	11	49	273	42	29
63	388	37	100	616	117	59
0	11	0	33	85	29	19
0	0	0	15	46	11	0
0	0	0	0	0	12	0
152	711	38	694	1109	394	467
22	0	0	54	17	10	0
0	0	0	430	0	37	0
0	10	0	203	0	24	0
0	0	0	430	39	14	0
106	31	46	262	95	94	165
11	21	11	40	85	142	71
326	891	114	632	2058	1703	1740
0	12	0	71	54	12	106

0	61	0	34	44	96	69
0	25	0	67	18	12	78
0	20	0	69	31	43	54
1148	435	315	70	809	1929	682
126	0	0	68	0	10	11
0	48	0	0	252	225	11
73	0	0	0	77	281	11
0	227	0	0	604	796	134
0	27	0	0	116	32	45
0	99	0	0	286	259	30
0	0	0	0	0	11	0
0	35	0	0	165	81	85
65	98	51	0	477	729	183
0	28	0	0	140	144	35
0	18	0	0	102	70	45
0	181	0	0	516	853	23
0	22	0	0	94	53	0
83	282	1142	163	1706	865	224
57	51	0	0	195	254	0
0	10	208	0	153	36	28
0	0	0	0	67	30	13
0	0	0	0	74	11	0
0	0	0	0	43	10	0
38	0	0	0	15	74	28
0	0	0	0	13	52	0
85	34	0	0	176	235	26
0	19	0	0	59	128	0
25	0	0	0	14	27	0
0	0	0	0	82	34	0
0	0	0	0	0	12	0
40	510	21	0	1644	1964	357
2105	929	208	191	2218	3525	1007
116	0	0	11	13	24	0
0	10	0	0	54	48	0
0	47	0	0	202	179	46
0	23	0	0	194	272	28
0	0	0	0	103	81	18
0	10	0	0	228	105	115
0	10	0	0	84	12	36
0	33	0	0	167	78	10
10	234	0	0	1012	777	127
29	39	0	0	55	170	81
0	0	0	0	15	47	0
0	0	0	0	28	10	0
0	298	37	0	985	833	236
391	1076	227	80	3750	5193	787

0	0	0	0	62	75	11
245	40	0	37	110	131	308
14	47	0	0	279	1549	53
0	12	0	0	210	131	0
662	876	418	52	2452	6197	381
0	14	141	23	11	35	0
0	0	0	0	35	11	0
0	10	0	0	150	88	12
0	29	0	0	44	108	0
0	0	0	0	41	15	35
54	0	0	50	73	35	37
10	0	0	0	86	23	91
2798	1191	1296	1902	1645	2467	742
0	0	0	0	0	16	0
242	386	646	315	1058	1392	182
10	54	0	0	133	144	35
0	0	0	54	0	11	12
0	200	0	0	961	523	96
37	0	20	0	80	40	0
0	18	0	0	206	99	12
0	0	0	0	26	45	0
10	0	0	0	29	25	0
29	0	0	31	0	15	39
26	36	10	49	68	45	49
10	125	42	207	235	96	92
76	30	13	22	102	81	100
33	0	0	60	47	53	22
0	0	0	0	10	21	0
0	112	0	79	101	124	0
201	644	543	248	847	858	124
199	13	31	334	0	41	0
0	11	0	12	15	25	0
0	0	344	14	0	35	0
19	0	15	0	10	47	0
0	0	0	0	68	70	0
0	58	0	0	14	15	0
0	23	0	39	69	23	21
0	81	74	85	30	68	80
12	41	0	0	60	50	0
227	112	101	145	219	199	126
0	12	0	0	10	22	0
28	16	10	11	10	23	0
63	80	27	0	215	444	178
1520	924	581	620	1157	1916	1063
0	10	10	0	47	32	46
22	879	20	28	270	67	1006

0	0	0	99	0	12	0
255	0	0	115	10	47	0
272	0	0	125	22	30	81
535	1155	453	65	1616	1234	1470
0	10	0	0	130	55	86
132	85	73	0	50	107	408
0	0	27	15	0	20	0
0	0	114	0	51	61	312
0	24	0	0	58	40	0
0	0	0	0	0	11	0
43	47	196	13	167	94	140
13	14	12	1544	22	64	32
0	53	0	0	47	82	75
15	0	0	0	0	11	0
32	10	0	76	22	49	21
0	0	0	0	131	123	22
20	35	0	34	73	136	0
0	0	0	0	28	41	0
0	0	0	0	0	10	0
0	0	104	0	56	102	57
0	76	38	251	111	94	49
0	0	0	12	0	10	40
151	134	78	65	264	173	12
43	0	0	0	0	10	0
24	72	0	26	101	49	0
140	163	0	284	677	959	13
81	10	17	11	21	11	67
122	92	22	384	154	168	92
48	16	28	0	45	29	0
12	36	0	67	42	107	0
31	0	0	0	0	10	22
10	24	0	85	95	122	0
173	79	25	115	229	238	99
15	0	26	26	0	12	10
22	0	13	56	37	13	107
0	118	0	142	828	1221	197
14	92	13	0	364	359	133
0	0	0	0	67	74	0
0	0	0	0	141	223	0
0	21	0	0	0	28	0
0	42	4192	294	38	58	1349
0	0	0	0	52	37	12
0	50	0	0	345	221	46
0	0	0	0	33	36	0
151	184	126	177	753	331	258
195	287	161	368	1100	12	0

44	502	64	143	2354	15	18
665	85	740	0	232	60	171
3499	1080	1519	384	7542	91	459
7374	92	272	1491	262	15	812
37	0	0	0	35	64	0
58	46	53	197	288	205	76
132	71	228	187	641	348	38
0	0	0	21	75	20	0
0	0	0	0	47	32	0
0	0	0	0	63	33	0
12	0	0	0	60	89	0
0	0	0	0	50	28	0
0	0	0	11	130	160	0
344	26	96	519	161	349	251
39	0	0	0	0	42	0
62	484	94	0	974	2799	49
35	0	0	0	10	33	0
0	0	0	0	0	11	0
0	0	0	0	0	11	0
0	0	0	0	37	37	0
0	0	0	0	72	119	0
54	29	14	959	176	11	91
0	555	0	0	252	136	0
0	0	0	0	10	24	0
0	65	0	0	283	239	0
0	36	0	47	51	107	0
0	45	0	23	452	368	94
0	0	0	0	50	110	0
0	0	0	0	141	271	12
0	0	0	0	0	12	0
0	0	0	0	10	115	0
0	67	0	0	361	1318	39
0	0	0	0	194	77	0
0	0	0	0	26	27	0
0	0	0	0	0	47	0
0	0	13	10	0	31	90
445	1341	795	2420	1165	4042	2795
57	984	438	619	1130	2552	3158
0	21	0	95	70	417	87
0	0	0	0	12	119	0
0	0	0	0	0	12	0
69	1390	84	67	4474	5892	304
0	125	63	42	704	263	145
0	0	27	0	40	62	42
0	62	0	0	281	230	38
109	419	65	31	1412	3522	185

38	158	91	0	642	1227	116
64	1087	118	0	3615	3856	149
0	0	0	167	23	66	77
0	0	0	175	0	14	0
0	55	51	41	56	58	90
0	673	325	287	371	934	1351
12	26	30	29	376	1240	111
0	15	47	0	0	32	143
27	0	0	15	0	73	157
0	0	0	0	0	12	0
0	0	0	0	113	458	78
0	0	0	0	0	39	90
0	0	0	0	43	80	0
0	184	75	173	28	125	167
127	0	0	50	0	90	266
0	0	0	0	0	169	0
0	0	0	0	73	157	0
0	54	0	0	247	438	13
0	227	74	93	52	71	305
0	0	0	0	0	107	0
0	0	0	144	0	44	0
0	10	0	156	0	85	11
0	82	22	66	35	15	489
0	0	0	47	23	117	0
25	169	222	950	103	327	998
0	106	29	40	449	308	73
0	20	17	0	61	43	0
58	573	97	33	2086	4282	63
0	144	81	98	118	60	146
0	81	16	44	105	10	73
0	164	0	0	562	618	32
10	16	47	15	49	34	0
0	0	0	0	29	58	0
0	0	10	42	0	38	39
0	286	153	43	206	377	486
0	84	0	0	50	57	140
0	133	55	0	109	128	65
0	0	0	0	0	11	30
0	0	0	0	39	31	0
15	21	90	115	81	716	29
10	206	129	303	879	1583	83
11	261	0	108	530	351	43
0	51	48	36	51	40	0
110	432	817	462	2161	377	50
0	0	0	0	0	42	134
303	130	69	118	859	520	549

1149	252	389	502	374	386	2354
39	11	0	70	0	31	11
0	0	0	155	26	43	82
156	62	0	94	24	59	187
2896	22	438	48	89	53	92
0	32	11	0	17	25	264
0	14	34	36	30	38	471
145	11	0	87	10	57	11
27	0	0	0	0	31	47
0	0	0	0	0	10	0
0	0	0	0	0	36	0
143	0	0	11	11	40	265
408	411	751	357	450	217	3198
0	0	51	11	0	23	808
25	49	0	136	0	10	287
32	60	11	163	0	32	311
306	280	153	636	55	106	1256
28	10	0	25	27	11	265
60	10	0	0	26	15	109
80	14	0	0	71	35	385
12	12	0	0	0	12	92
0	12	0	0	23	10	30
49	35	63	27	55	86	47
0	10	0	43	29	48	13
0	0	12	0	10	23	26
0	63	0	0	33	10	55
802	760	2897	1417	2560	642	1424
115	10	10	106	85	58	167
14	52	0	29	76	30	65
76	43	10	14	71	12	10
315	2072	230	596	1408	369	1550
197	274	28	121	165	46	87
10	0	0	35	35	23	40
64	0	500	0	7246	10	351
136	266	33	124	173	55	58
0	26	11	44	54	75	74
38	0	0	11	10	39	0
152	330	89	86	223	57	421
23	45	23	60	149	64	120
46	53	48	241	171	108	44
250	63	14	124	12	148	54
535	28	0	69	11	255	77
31	0	0	0	0	22	53
40	64	49	118	128	34	2073
41	96	1501	93	388	104	239
10	0	0	0	98	39	45

51	230	102	340	1136	663	107
134	14	51	21	158	84	59
31	18	0	56	0	48	73
87	0	125	10	136	62	82
0	0	0	0	59	43	0
0	0	0	0	52	50	0
31	84	0	31	45	12	0
14	0	0	0	0	24	11
13	0	0	0	0	11	0
0	0	0	0	51	13	0
0	0	0	0	0	12	0
79	43	18	230	242	348	35
0	0	0	0	0	29	0
0	0	31	11	0	41	0
0	0	0	10	86	43	12
0	13	0	0	80	61	0
0	0	0	0	0	10	0
0	0	0	15	110	884	0
192	364	1031	220	1333	2355	856
0	0	0	0	36	622	0
0	0	0	0	10	55	0
0	0	0	0	53	681	0
0	0	0	0	17	23	0
70	220	64	98	1813	1892	878
0	0	0	0	0	12	0
0	0	0	0	133	210	0
13	0	0	30	93	51	454
0	0	0	18	68	13	307
0	0	0	0	64	39	0
0	0	0	0	88	149	15
0	0	0	0	27	48	0
0	0	0	0	47	75	0
0	0	0	0	0	23	0
0	0	0	0	52	130	0
0	0	0	0	212	399	446
0	0	0	0	87	37	0
0	0	0	0	10	32	0
0	0	0	0	0	10	0
0	12	0	24	20	68	0
34	10	0	13	35	35	0
0	11	0	0	78	21	0
0	22	0	0	246	10	0
59	13	0	14	26	41	0
129	29	0	12	146	51	14
0	0	0	0	28	23	0
0	0	0	0	0	10	0

0	20	0	105	237	142	31
0	0	0	0	39	24	0
0	0	12	56	0	12	0
241	443	744	645	723	623	238
0	0	0	47	208	436	0
10	11	0	0	123	504	0
0	15	0	61	238	141	0
0	34	0	31	257	328	24
0	0	0	57	14	13	0
0	14	0	71	97	14	115
0	22	72	370	24	198	50
86	110	177	611	557	1114	70
0	201	10	321	1596	1954	20
219	303	351	222	1113	2086	109
0	23	0	26	199	262	14
0	0	0	0	0	21	0
0	35	0	0	0	23	0
0	33	0	17	108	192	0
0	0	0	0	0	10	0
0	0	0	11	37	30	0
0	26	44	10	185	250	20
0	0	0	0	0	35	0
0	64	0	191	266	470	44
0	341	126	116	2058	2546	102
0	17	0	0	34	32	0
0	12	0	0	76	32	0
0	0	0	278	32	60	24
0	0	0	0	0	11	0
312	515	272	145	3947	463	124
0	19	0	0	358	14	0
0	0	0	0	174	10	10
0	77	0	0	533	54	0
0	29	0	0	440	56	0
0	12	0	0	29	107	0
0	0	0	0	11	21	0
0	0	0	0	0	15	0
0	33	15	54	0	12	0
44	211	16	956	436	582	129
0	0	0	0	0	16	0
0	35	0	212	103	49	0
0	0	0	280	43	10	43
0	0	0	0	62	44	0
0	0	0	0	50	76	0
0	10	0	0	10	40	0
0	0	0	0	0	11	0
0	0	0	0	70	15	0

0	13	0	85	71	10	57
0	0	0	156	0	30	0
0	0	0	0	0	12	0
0	0	0	0	21	12	0
0	0	0	0	50	43	0
0	40	0	0	126	225	0
0	0	0	51	53	26	0
0	130	0	127	23	26	43
312	539	468	1393	2484	2631	597
41	21	40	74	38	44	0
0	12	0	74	44	12	94
1790	397	257	229	1124	259	153
0	0	0	0	29	43	0
0	0	0	45	0	10	0
400	18	0	34	100	11	0
21	520	228	660	1120	430	463
0	33	13	18	234	50	13
0	0	0	0	0	66	0
0	0	0	0	70	63	0
12	10	0	30	41	33	0
0	0	0	11	0	41	0
0	0	14	0	0	12	0
0	11	0	23	48	34	0
31	38	138	77	63	286	28
39	649	246	162	2901	2564	275
126	470	383	302	1103	1750	812
19	79	18	18	64	130	51
0	23	10	27	45	20	186
0	70	68	58	145	62	238
0	0	0	0	0	11	11
0	0	0	0	0	34	0
0	0	0	0	41	189	0
0	27	0	0	12	37	35
0	10	38	0	0	12	34
0	71	88	64	117	93	126
0	0	0	216	0	13	0
0	561	0	24	2744	2119	92
1028	5397	968	378	16789	23985	1937
0	232	13	55	649	686	30
0	13	0	0	134	35	0
26	1068	111	0	4402	7851	401
0	0	0	0	29	58	0
0	36	0	0	144	280	0
0	128	0	0	721	484	0
0	0	0	0	44	27	0
15	240	10	14	1452	1526	57

0	135	0	0	637	770	41
0	103	0	0	436	396	0
0	0	0	0	52	10	0
46	1003	41	0	4265	3051	294
0	0	13	0	0	53	0
0	0	0	24	87	50	147
11	61	31	52	60	117	100
0	0	0	11	71	50	61
26	12	38	35	106	33	25
0	0	0	0	27	56	11
40	419	78	116	573	514	476
0	169	0	0	657	913	0
0	0	0	0	121	160	48
67	15	0	0	72	95	0
13	86	0	45	15	126	12
0	0	0	0	0	78	0
0	0	0	0	0	20	0
0	0	0	0	10	12	0
39	11	82	68	207	107	84
0	0	0	0	82	170	18
0	119	0	140	643	643	0
0	0	0	0	0	12	0
0	289	0	43	1035	1317	114
0	397	0	17	2706	3190	50
12	492	33	10	2184	3315	225
0	109	0	0	611	336	0
0	0	0	0	22	35	53
0	91	0	0	421	1352	79
0	41	0	0	533	497	10
0	15	0	0	240	221	0
0	70	0	0	555	929	0
0	27	0	0	107	111	0
0	44	0	0	358	314	0
0	11	0	13	15	21	25
0	0	0	0	38	10	0
0	0	0	0	133	85	0
13	134	13	0	512	1197	0
0	197	11	56	376	527	80
3294	3278	2358	2039	11044	16337	2401
0	208	0	0	1247	955	15
0	0	0	0	0	49	0
0	10	0	0	202	116	0
11	0	40	93	353	371	109
0	0	0	0	113	50	0
0	35	0	0	112	242	0
0	0	0	0	0	75	0

0	0	0	0	41	121	0
0	122	15	48	411	345	98
0	27	0	0	200	181	10
0	0	0	0	38	51	0
0	0	0	0	45	149	0
25	0	0	66	123	209	0
0	10	0	0	83	145	0
0	0	0	0	18	32	0
0	0	0	0	0	10	0
0	0	0	0	26	62	0
0	0	0	12	79	105	0
12	111	0	0	652	668	34
329	2092	676	1427	7141	2215	2270
112	198	132	247	595	221	169
0	375	10	0	1827	1858	65
0	0	0	0	34	148	0
0	0	0	0	84	62	0
10	60	0	0	58	24	39
0	70	10	0	379	14	27
208	176	194	271	971	406	376
31	203	63	123	749	284	214
86	118	97	39	255	252	49
53	45	13	47	367	516	24
0	0	0	0	60	43	0
0	0	0	0	42	10	31
0	0	0	0	43	68	0
0	40	0	0	273	336	0
29	299	29	49	715	296	252
0	0	0	0	26	133	11
0	0	0	25	154	105	28
0	43	0	0	182	377	0
0	0	0	13	0	22	0
289	112	141	811	53	186	92
0	0	15	135	0	10	0
0	0	16	109	0	13	13
0	0	0	0	131	28	10
13	37	13	338	0	69	78
0	25	0	78	20	15	73
90	11	0	250	25	218	95
38	63	42	510	78	142	127
10	0	0	129	0	12	15
85	45	45	225	127	113	0
0	22	0	41	170	98	0
64	590	200	273	1610	1014	1474
14	0	0	0	175	63	33
21	0	0	0	82	74	0

0	10	0	0	31	35	0
0	0	0	0	67	92	13
0	0	0	0	32	28	0
0	77	53	13	156	150	98
27	93	70	185	36	55	308
0	321	64	80	64	10	718
0	73	80	126	52	18	569
0	0	0	22	0	28	31
33	52	158	30	12	22	202
19	0	0	0	67	186	12
0	0	0	0	81	53	22
0	28	0	0	267	190	0
0	36	0	0	69	74	0
0	0	0	0	0	29	0
0	0	0	0	78	40	0
0	0	0	0	36	48	0
0	0	0	0	0	18	0
0	79	0	0	345	274	0
0	10	0	0	31	29	0
0	0	0	0	0	24	13
41	166	78	0	599	17	63
0	0	0	0	0	10	0
64	485	1216	76	5633	393	731
0	0	0	0	25	11	0
116	211	670	153	5523	444	536
0	0	0	0	0	73	12
0	92	187	0	270	45	57
47	121	572	35	1536	110	140
16	16	30	0	18	32	23
0	53	0	0	35	34	0
0	0	0	0	0	25	0
151	0	0	0	12	33	13
32	0	43	0	0	13	45
0	20	0	0	89	48	0
0	0	0	24	0	87	23
0	0	0	0	0	42	0
0	0	0	15	158	111	0
0	0	0	11	26	94	0
0	0	0	10	108	342	0
0	0	0	0	167	14	21
0	10	0	185	125	17	0
59	0	0	0	10	24	0
0	265	0	4911	389	1112	123
0	35	0	164	0	476	13
17	0	0	333	0	90	10
0	0	0	0	0	47	0

0	0	0	0	0	14	0
0	54	37	195	146	154	194
0	41	0	155	0	11	132
0	0	0	32	0	68	37
0	42	0	43	10	24	0
0	130	0	30	58	29	11
0	40	27	0	60	46	31
0	0	0	213	28	95	0
993	1361	453	850	2400	812	4435
537	700	156	773	3531	252	495
317	369	48	305	1489	124	169
0	0	0	140	71	15	0
0	0	0	0	0	28	0
0	0	0	146	59	29	22
0	0	0	0	10	13	0
0	0	0	63	11	39	0
0	0	0	0	0	43	0
0	0	0	0	0	78	13
0	0	0	0	10	10	0
0	0	0	0	0	10	0
0	62	11	68	176	218	87
15	24	0	153	72	264	15
0	0	0	15	60	150	10
0	0	11	0	0	78	0
0	0	0	0	0	11	0
10	214	34	57	1516	640	378
302	1484	1110	531	4799	4002	4778
201	163	447	573	280	153	3463
1421	8911	4547	2287	4287	2930	19919
0	124	59	77	98	12	618
0	2337	74	34	149	14	812
0	0	15	87	90	10	198
280	1367	2912	830	2202	604	5442
163	955	452	188	561	229	2969
0	15	68	48	35	29	716
0	0	0	0	93	14	45
40	523	31	4296	613	1460	842
20	182	29	687	352	218	524
0	66	0	986	373	557	400
44	373	158	274	697	7618	446
0	0	18	0	22	842	0
0	0	0	0	11	245	0
44	266	44	1013	400	4185	457
0	136	0	230	13	54	44
0	0	77	0	38	624	0
0	175	0	3725	141	496	441

45	133	0	425	41	160	186
1341	3865	821	2063	4040	1702	1572
0	0	0	18	0	16	21
11	258	23	216	157	181	744
0	17	0	78	0	24	37
0	0	0	0	0	41	37
104	365	162	1162	286	427	534
14	26	0	20	74	11	21
0	0	0	0	47	44	0
0	0	0	203	0	47	0
0	28	0	368	0	42	0
0	11	0	77	0	107	48
0	47	0	0	40	83	0
0	23	0	0	78	78	0
74	120	0	589	1545	1823	30
1412	533	259	571	1305	1566	551
12	224	11	240	800	934	35
0	179	18	120	768	577	119
0	46	0	51	341	264	10
0	0	0	0	20	33	0
0	97	0	268	457	482	43
0	30	0	0	159	242	14
0	0	0	0	92	32	0
0	54	0	0	535	907	24
0	75	13	78	143	69	664
0	0	0	0	72	247	0
0	0	0	0	0	31	0
0	0	0	0	52	100	0
0	25	0	0	176	154	0
0	0	0	201	234	442	0
0	0	0	80	94	116	42
0	0	0	73	0	36	0
0	0	0	85	155	348	269
11	131	35	174	229	92	1041
0	22	11	115	108	15	26
0	91	47	74	31	43	39
0	0	0	0	86	37	138
0	0	0	24	0	12	40
71	141	86	199	137	90	671
0	65	23	196	91	167	10
14	13	34	60	384	95	50
26	1778	101	0	8001	8112	209
0	0	0	0	203	153	0
0	1135	44	0	6834	4569	257
0	0	0	0	0	72	0
0	1078	19	0	4446	5206	169

0	12	0	0	211	106	0
0	67	10	56	89	170	0
0	115	82	109	931	949	52
0	16	0	28	67	52	11
0	20	0	225	0	10	11
0	0	0	0	30	44	0
0	0	0	0	0	11	0
0	0	0	0	0	12	0
0	11	0	17	0	10	0
0	77	12	181	554	598	41
63	0	0	10	36	0	42
10	0	0	45	37	0	0
0	15	0	27	14	0	37
36	0	0	37	19	0	11
0	54	0	47	166	0	100
0	0	0	23	10	0	44
0	11	0	0	53	0	0
0	13	0	13	17	0	56
0	0	0	712	0	0	11
0	0	0	81	0	0	0
0	0	0	0	18	0	0
0	0	0	0	43	0	11
0	23	0	0	40	0	0
0	12	213	10	0	0	33
0	10	0	13	108	0	0
0	0	28	0	56	0	0
0	0	10	0	0	0	0
19	0	0	0	22	0	0
0	0	0	0	43	0	12
0	0	0	59	29	0	0
0	0	0	12	0	0	0
0	0	0	0	0	0	0
0	0	14	0	0	0	0
0	0	0	0	10	0	0
76	10	10	0	0	0	0
0	0	0	0	0	0	0
0	0	0	0	0	0	0
0	0	12	0	0	0	0
0	0	0	0	0	0	0
0	0	250	0	20	0	0
0	0	0	0	0	0	0
0	0	57	0	15	0	0
0	0	0	0	0	0	0
0	0	0	0	15	0	39
14	0	0	0	0	0	99
0	0	0	68	0	0	0

0	0	0	57	0	0	0
0	0	0	0	0	0	0
0	0	0	0	0	0	0
0	18	0	103	0	0	13
0	0	0	0	67	0	0
0	0	0	0	0	0	0
0	0	22	0	11	0	0
0	0	0	0	0	0	0
0	0	0	0	0	0	0
0	64	0	0	25	0	0
0	0	0	40	0	0	0
0	11	0	10	11	0	0
0	0	0	0	0	0	0
0	0	0	43	0	0	10
27	57	0	0	0	0	0
34	0	0	0	0	0	0
67	0	0	0	0	0	0
0	0	0	0	0	0	0
0	40	0	0	31	0	115
0	35	0	0	28	0	125
0	0	0	0	0	0	0
0	0	0	0	0	0	52
0	0	0	38	28	0	0
83	0	0	0	0	0	0
0	0	0	0	0	0	0
11	0	0	0	0	0	0
0	0	0	70	0	0	0
0	0	0	0	0	0	0
0	0	0	0	0	0	0
0	0	0	0	0	0	0
0	0	0	0	0	0	0
0	0	0	0	23	0	0
0	0	0	0	0	0	0
30	0	0	118	66	0	0
0	0	0	0	383	0	0
0	0	0	91	0	0	0
0	10	0	0	131	0	0
29	29	31	16	31	0	46
0	0	0	0	93	0	0
0	0	0	0	0	0	0
28	0	31	618	0	0	10
0	0	0	423	0	0	0
0	0	0	245	0	0	15
0	0	0	0	0	0	0
126	0	11	0	133	0	0
0	0	0	0	0	0	0

0	0	0	118	29	0	0
0	0	0	11	63	0	0
0	0	0	0	0	0	0
0	0	0	22	0	0	0
0	32	112	28	323	0	0
83	79	129	207	195	0	75
0	0	0	0	15	0	0
0	0	0	61	0	0	0
0	0	0	43	0	0	0
53	0	0	13	0	0	0
0	0	0	25	0	0	0
0	0	0	0	0	0	0
10	0	140	138	0	0	0
0	0	0	0	0	0	0
0	0	0	0	0	0	0
0	0	175	0	0	0	0
0	0	152	0	0	0	0
0	0	0	0	24	0	0
84	23	11	10	21	0	101
0	0	0	0	0	0	0
0	0	38	0	0	0	0
0	0	106	0	0	0	0
0	0	204	29	0	0	0
0	0	0	0	135	0	10
0	0	0	0	39	0	30
0	11	0	86	10	0	127
0	0	0	0	0	0	0
0	0	0	0	0	0	24
0	43	30	0	14	0	176
0	0	0	0	0	0	44
0	0	0	0	0	0	0
0	0	0	10	0	0	154
0	0	0	0	0	0	0
0	12	10	64	36	0	176
0	0	0	0	24	0	0
0	0	0	0	0	0	0
0	0	0	0	0	0	0
0	0	0	11	0	0	0
0	0	0	0	12	0	0
0	17	0	23	10	0	21
0	0	0	0	0	0	49
0	0	0	0	0	0	0
0	0	0	0	0	0	36
0	0	0	0	0	0	0
0	0	0	0	0	0	0
41	177	72	0	147	0	388

0	0	0	1013	213	0	24
0	0	0	0	0	0	0
0	0	0	0	0	0	0
0	0	0	36	0	0	0
0	0	0	38	0	0	213
0	0	0	0	0	0	12
0	0	0	0	19	0	0
0	11	11	0	0	0	197
0	0	0	0	0	0	0
0	0	0	0	0	0	0
0	0	0	60	91	0	13
0	12	13	0	0	0	151
0	33	60	0	67	0	804
0	0	0	0	0	0	42
0	22	55	0	64	0	309
29	0	0	0	0	0	27
0	0	0	0	0	0	40
12	0	0	45	0	0	0
99	0	0	0	0	0	0
0	0	0	0	0	0	0
0	0	24	0	0	0	0
0	0	0	17	19	0	23
0	0	0	23	34	0	0
10	11	30	39	66	0	0
0	0	0	0	0	0	167
0	0	0	0	0	0	122
12	117	0	207	25	0	532
0	0	0	38	0	0	99
0	51	0	114	15	0	358
0	0	0	0	0	0	0
10	0	0	0	19	0	10
0	13	0	0	26	0	231
0	0	0	0	0	0	0
10	0	0	0	32	0	171
0	0	0	0	10	0	0
0	0	0	0	11	0	23
0	0	0	0	0	0	0
0	59	0	25	48	0	134
0	42	25	0	31	0	203
0	21	0	0	0	0	0
0	0	0	12	0	0	0
0	0	0	0	53	0	38
0	0	0	0	0	0	0
0	0	0	0	0	0	0
0	11	0	0	0	0	0
0	0	0	0	0	0	0

0	0	0	0	0	0	0
11	56	0	246	132	0	60
0	0	0	0	0	0	0
0	0	0	13	0	0	16
20	0	0	32	64	0	0
0	0	14	0	17	0	16
0	0	0	0	0	0	11
0	0	0	0	0	0	0
0	14	11	0	13	0	99
0	0	0	35	11	0	121
12	0	0	13	0	0	0
0	0	0	0	55	0	47
0	0	0	0	0	0	0
0	0	12	0	0	0	0
0	0	85	0	0	0	0
0	0	0	0	0	0	0
21	0	0	12	10	0	95
0	0	53	0	0	0	0
25	28	0	43	28	0	0
0	0	0	10	0	0	0
0	35	10	11	51	0	792
0	0	0	0	0	0	0
0	0	0	21	0	0	0
0	0	269	30	0	0	0
0	0	0	0	0	0	0
0	0	40	0	0	0	13
0	0	0	0	0	0	0
0	0	0	10	0	0	0
0	0	229	0	45	0	0
0	0	80	0	0	0	0
0	0	480	14	0	0	25
0	21	1338	66	21	0	43
0	13	339	0	0	0	0
0	10	117	0	0	0	0
20	0	0	10	0	0	0
0	0	0	0	0	0	0
0	38	0	10	0	0	59
48	0	161	10	0	0	63
10	0	48	0	0	0	0
64	0	17	0	0	0	21
0	0	0	0	0	0	0
0	0	105	0	0	0	13
0	0	222	0	0	0	0
0	0	138	0	0	0	0
32	0	91	0	0	0	0
11	0	0	0	0	0	0

0	0	0	0	0	0	0
0	0	0	0	0	0	0
0	0	30	0	0	0	0
10	0	0	20	13	0	0
0	11	13	13	0	0	10
0	0	0	0	0	0	0
0	0	0	0	0	0	0
0	0	0	14	0	0	27
0	0	0	0	0	0	0
0	0	0	145	56	0	14
0	0	0	288	0	0	0
0	0	0	95	35	0	0
0	0	0	0	0	0	0
0	0	0	44	0	0	0
0	0	0	0	0	0	0
0	14	0	10	37	0	0
0	0	0	0	0	0	0
0	0	0	0	0	0	0
0	0	0	59	0	0	0
0	12	0	0	39	0	0
0	0	0	0	0	0	0
10	0	0	0	11	0	0
0	0	0	0	16	0	0
0	0	0	0	0	0	0
13	0	0	0	0	0	0
0	0	0	0	0	0	0
0	0	0	205	0	0	0
0	0	0	0	0	0	0
0	0	0	0	43	0	0
0	0	0	10	0	0	0
0	10	0	0	86	0	33
0	0	0	0	99	0	0
0	0	0	0	27	0	0
0	0	0	0	0	0	0
0	0	10	947	57	0	24
0	0	0	81	10	0	0
0	0	0	15	0	0	0
0	0	0	174	0	0	0
0	0	0	63	0	0	21
18	0	14	0	0	0	0
0	0	0	0	0	0	0
0	0	0	105	0	0	0
0	0	0	0	0	0	0
0	0	0	0	0	0	0
10	38	0	22	0	0	135
0	0	0	0	14	0	43

0	0	0	102	56	0	95
0	0	0	0	0	0	27
0	0	0	0	13	0	0
0	128	108	32	86	0	30
0	10	0	0	14	0	0
0	0	0	0	23	0	28
0	0	0	0	0	0	0
0	61	0	0	169	0	0
0	12	0	0	13	0	0
0	0	0	0	0	0	0
0	63	0	0	18	0	0
0	0	0	0	0	0	0
0	0	0	0	50	0	11
0	0	0	0	10	0	0
19	25	122	0	117	0	0
0	38	58	0	53	0	0
0	11	0	0	117	0	0
10	0	723	0	0	0	41
0	0	0	0	0	0	0
0	0	68	0	0	0	43
0	0	0	0	10	0	0
0	0	0	0	0	0	0
0	11	1705	0	0	0	136
0	0	0	36	0	0	10
48	0	0	12	0	0	0
27	12	0	46	0	0	109
0	0	0	0	26	0	0
0	0	0	10	24	0	0
0	0	0	170	0	0	0
0	0	0	73	0	0	0
0	0	0	0	0	0	0
0	0	10	0	0	0	16
0	0	0	0	0	0	0
0	0	91	0	0	0	0
0	0	0	13	0	0	0
0	0	0	28	0	0	0
0	0	13	0	0	0	0
0	65	0	0	35	0	0
0	10	0	0	12	0	0
0	0	0	0	0	0	0
0	73	0	169	101	0	235
0	99	0	194	0	0	17
0	0	0	0	35	0	0
0	0	0	73	35	0	0
0	0	0	15	0	0	0
0	0	0	0	0	0	29

0	0	0	0	0	0	10
0	0	0	0	0	0	0
0	28	0	29	0	0	0
0	0	0	0	0	0	0
0	0	0	0	0	0	10
0	0	0	0	11	0	0
0	0	0	0	0	0	372
0	0	0	20	86	0	0
25	26	11	0	24	0	0
0	15	0	0	756	0	0
744	20	0	11	0	0	42
0	0	0	0	0	0	11
0	0	0	43	0	0	0
0	20	0	85	12	0	0
0	0	0	16	0	0	113
0	0	0	10	0	0	55
0	85	0	18	0	0	0
0	0	0	48	0	0	82
0	0	0	0	0	0	0
0	0	0	0	0	0	28
0	0	0	0	0	0	0
0	0	0	45	0	0	41
0	0	0	0	0	0	0
0	0	0	0	0	0	0
0	0	0	0	0	0	0
0	0	0	0	0	0	0
0	0	0	0	0	0	0
11	64	0	0	54	0	306
0	0	0	0	0	0	71
0	0	0	0	0	0	98
0	45	0	49	12	0	536
0	52	0	16	84	0	176
0	0	0	40	0	0	231
0	0	0	0	13	0	0
0	0	0	0	0	0	0
0	0	0	0	0	0	0
0	0	0	0	0	0	0
0	0	0	10	0	0	0
0	0	0	0	0	0	0
0	0	0	0	0	0	0
0	0	0	0	0	0	0
0	194	10	0	129	0	0
0	0	0	0	0	0	0
0	27	0	0	0	0	0
0	0	0	0	0	0	0
0	0	0	0	0	0	0

0	0	0	0	0	0	10
0	0	0	0	0	0	0
0	0	0	0	142	0	0
0	0	0	0	0	0	0
0	15	0	41	10	0	12
0	0	0	0	33	0	0
0	0	0	0	0	0	0
0	16	0	0	0	0	0
0	0	0	13	0	0	0
0	0	0	62	0	0	0
33	0	0	0	0	0	0
0	0	0	114	0	0	26
0	0	0	0	0	0	0
0	0	247	0	0	0	0
0	0	0	130	46	0	21
0	0	106	0	0	0	22
0	0	0	0	0	0	0
0	0	0	0	0	0	0
0	0	0	0	0	0	0
0	0	0	0	0	0	0
0	22	0	0	14	0	211
0	0	0	36	0	0	0
0	0	0	38	0	0	0
36	0	54	0	0	0	0
0	0	28	0	37	0	0
0	0	0	0	0	0	11
0	0	0	0	0	0	0
0	0	0	0	0	0	0
70	0	0	10	0	0	0
0	0	13	0	0	0	0
0	0	0	0	0	0	0
0	0	0	0	0	0	0
0	21	0	0	48	0	23
12	0	0	0	0	0	0
0	0	0	0	0	0	0
24	11	22	10	75	0	24
0	0	0	0	0	0	0
0	0	0	0	0	0	0
0	0	0	0	0	0	0
0	0	0	0	0	0	0
0	0	0	0	0	0	0
0	0	0	0	0	0	0
0	0	0	0	0	0	19
0	0	0	0	0	0	13
0	0	0	0	0	0	21
0	0	0	85	0	0	0

0	0	0	36	0	0	0
0	0	0	0	0	0	0
0	0	0	0	0	0	0
0	0	50	0	0	0	24
0	21	0	0	0	0	0
0	0	0	0	0	0	0
38	0	0	0	0	0	0
0	0	0	0	0	0	0
0	0	0	0	0	0	0
0	0	0	0	0	0	0
0	0	0	0	0	0	0
0	51	0	0	0	0	0
0	0	0	11	0	0	0
0	0	0	35	10	0	0
0	0	0	0	0	0	0
0	0	0	0	0	0	0
0	0	15	0	0	0	0
0	0	0	0	58	0	0
0	0	0	0	0	0	0
0	0	0	32	0	0	33
0	0	0	0	25	0	52
0	0	0	0	14	0	21
0	0	0	0	0	0	0
0	0	0	10	0	0	0
0	0	0	0	0	0	0
0	0	0	0	0	0	10
0	71	0	0	12	0	16
0	0	0	0	0	0	0
0	0	0	0	0	0	0
0	0	0	0	61	0	0
0	0	0	61	0	0	0
0	0	0	0	0	0	0
0	0	0	0	0	0	0
0	0	0	53	51	0	0
0	0	0	0	0	0	0
0	0	0	79	0	0	0
0	0	0	0	0	0	0
0	0	0	0	0	0	0
0	0	74	0	0	0	41
0	0	0	0	0	0	0
652	0	27	0	87	0	0
0	0	0	13	0	0	0
0	0	0	0	0	0	0
0	0	0	0	0	0	0
0	0	0	0	0	0	0
0	0	0	0	0	0	0

0	0	0	0	0	0	0
0	0	0	0	0	0	0
0	0	0	0	0	0	0
0	0	0	0	0	0	0
0	0	0	0	0	0	0
0	0	0	0	0	0	0
0	0	0	0	25	0	0
12	0	0	0	10	0	0
0	0	0	0	0	0	0
0	0	0	0	0	0	0
0	0	0	0	0	0	0
0	0	0	0	0	0	0
0	0	0	0	0	0	0
0	0	0	0	0	0	0
0	0	0	0	0	0	0
0	0	0	0	0	0	0
0	0	0	0	0	0	328
0	11	0	0	0	0	0
52	0	24	21	0	0	0
169	0	0	0	0	0	0
0	0	10	0	0	0	0
0	10	0	0	0	0	14
0	0	0	0	0	0	0
0	0	0	0	0	0	0
0	0	0	0	0	0	0
0	0	0	0	0	0	0
10	0	0	0	0	0	0
0	0	0	0	13	0	18
0	0	0	0	0	0	0
0	13	0	23	0	0	0
0	0	0	0	0	0	0
0	0	0	0	0	0	0
0	12	0	67	0	0	0
0	0	0	0	0	0	0
0	0	0	0	0	0	0
0	44	11	0	11	0	30
0	0	0	0	0	0	0
0	0	96	22	0	0	0
0	0	12	0	0	0	0
0	0	12	0	0	0	0
0	0	56	0	0	0	0
0	0	54	0	0	0	0
0	0	116	0	0	0	0
0	0	23	0	0	0	0
0	0	123	0	0	0	0
0	0	429	25	10	0	333

0	0	29	0	0	0	0
0	0	45	0	0	0	0
0	0	32	0	0	0	0
0	0	41	0	0	0	0
0	0	44	0	0	0	0
0	0	10	0	0	0	0
0	0	33	0	0	0	0
0	0	11	0	11	0	0
0	0	46	0	0	0	0
0	0	188	0	0	0	0
0	0	11	0	0	0	0
0	52	45	37	59	0	109
0	0	118	0	0	0	0
95	43	131	0	84	0	12
0	165	13	0	581	0	0
0	0	10	13	0	0	0
10	0	10	0	0	0	0
0	0	10	0	0	0	0
0	0	10	0	0	0	0
0	0	14	0	0	0	0
0	0	49	0	0	0	0
0	0	11	0	0	0	0
0	0	37	0	11	0	10
0	0	117	0	0	0	0
0	0	13	0	0	0	0
0	0	22	0	0	0	0
0	0	10	0	0	0	0
0	0	97	0	0	0	0
0	0	10	0	0	0	0
0	0	37	0	0	0	0
0	0	41	0	0	0	0
0	0	40	0	0	0	47
0	0	133	13	0	0	96
0	0	25	0	0	0	0
33	0	46	0	110	0	53
0	0	39	0	95	0	0
0	0	101	0	81	0	0
0	0	65	0	0	0	0
0	0	40	0	0	0	0
0	0	12	0	0	0	0
0	0	31	0	0	0	0
0	61	10	0	0	0	0
0	0	72	0	0	0	0
0	0	42	0	0	0	0
0	0	83	0	45	0	39
0	0	30	0	0	0	0

0	0	77	22	33	0	0
0	0	228	11	12	0	0
0	0	34	0	0	0	0
0	0	19	0	0	0	0
0	0	16	0	0	0	0
0	0	97	0	0	0	0
0	0	65	0	0	0	0
0	0	20	0	0	0	0
0	0	10	0	0	0	0
0	0	12	0	0	0	0
0	0	38	0	0	0	11
0	0	12	0	0	0	0
0	0	22	0	0	0	0
0	0	23	0	0	0	0
0	0	16	0	0	0	0
0	0	29	0	0	0	0
0	0	11	0	13	0	52
0	0	49	13	0	0	0
0	0	12	0	0	0	0
0	0	58	0	0	0	10
0	0	47	0	0	0	0
0	0	27	0	0	0	0
0	0	10	0	0	0	0
0	0	189	0	0	0	0
0	0	226	0	0	0	0
0	0	60	0	0	0	0
0	0	14	0	0	0	0
0	0	24	0	0	0	0
0	0	14	0	0	0	0
0	0	24	368	0	0	0
0	0	26	0	0	0	0
0	32	12	0	0	0	0
0	0	41	0	0	0	0
0	0	57	0	12	0	0
0	0	19	0	0	0	0
0	0	120	0	0	0	0
0	0	14	0	0	0	0
0	0	16	0	0	0	0
0	0	13	0	0	0	10
0	0	165	88	15	0	39
0	0	12	0	0	0	0
0	0	17	0	0	0	0
0	0	24	0	0	0	23
0	0	0	0	0	0	11
0	0	0	31	0	0	0
0	0	0	0	0	0	0

0	0	0	0	0	0	0
0	0	0	0	0	0	0
0	0	0	73	24	0	43
0	14	0	0	0	0	0
0	0	0	0	0	0	0
0	0	0	0	0	0	0
0	0	0	0	0	0	0
0	0	0	0	0	0	0
0	0	0	0	32	0	14
0	0	0	0	0	0	0
0	0	0	0	0	0	0
0	0	0	0	0	0	0
0	0	0	0	0	0	0
0	0	0	0	0	0	0
0	0	0	0	40	0	0
0	0	0	0	28	0	0
0	0	0	0	0	0	0
0	0	0	0	0	0	0
0	0	0	0	0	0	0
0	0	0	0	0	0	0
0	0	0	0	0	0	0
0	0	0	0	22	0	0
0	0	0	0	13	0	0
0	0	0	0	0	0	0
0	0	0	49	0	0	0
0	0	0	0	0	0	0
0	0	0	0	0	0	0
0	0	0	0	0	0	12
0	0	0	0	0	0	0
0	0	0	0	0	0	0
0	0	0	73	0	0	0
0	0	0	0	0	0	0
0	0	0	10	28	0	21
39	0	0	0	0	0	0
56	26	0	0	96	0	0
0	0	0	0	0	0	0
270	0	0	0	11	0	27
0	0	0	0	0	0	0
0	0	0	0	0	0	0
0	0	0	0	0	0	0
0	0	0	0	0	0	0
0	0	0	0	0	0	0
0	0	0	0	10	0	0
0	0	0	0	0	0	0
0	0	0	0	0	0	0
0	0	0	0	0	0	0

0	0	0	0	0	0	0
0	0	0	196	27	0	0
0	10	0	12	0	0	28
0	0	0	198	80	0	0
10	0	0	0	0	0	0
0	0	0	12	0	0	0
0	0	0	0	0	0	11
0	0	0	0	0	0	0
0	0	0	0	0	0	0
0	0	0	0	0	0	0
0	0	0	0	0	0	0
0	0	0	0	0	0	0
0	0	0	0	11	0	0
0	0	0	0	0	0	0
0	0	0	0	0	0	0
0	0	0	34	0	0	0
0	0	0	0	0	0	122
0	0	0	0	0	0	0
0	0	0	10	0	0	0
0	0	0	0	0	0	42
0	0	0	0	0	0	0
0	0	0	0	0	0	0
224	417	0	0	3197	0	0
143	795	0	0	6282	0	0
0	0	0	0	0	0	0
0	0	0	0	0	0	0
0	0	0	0	0	0	0
0	0	0	0	0	0	0
0	0	0	0	0	0	0
0	0	0	0	0	0	0
0	0	0	0	0	0	48
0	0	0	0	0	0	0
0	62	0	0	0	0	11
0	11	0	0	0	0	0
0	0	0	0	0	0	0
11	0	0	0	0	0	0
0	0	0	0	0	0	0
66	0	0	0	0	0	0
0	0	0	0	0	0	0
15	0	0	0	0	0	0
0	0	0	0	0	0	0
0	0	0	0	0	0	0
0	0	0	0	0	0	0
0	0	0	0	0	0	0
0	0	0	0	0	0	0
0	0	0	0	0	0	0

0	0	0	0	0	0	0
0	0	0	0	0	0	0
0	10	0	43	22	0	0
0	19	0	0	30	0	0
0	0	0	0	0	0	0
0	0	0	0	23	0	0
0	0	0	0	259	0	0
59	0	0	0	11	0	14
10	0	0	0	0	0	0
0	0	0	0	0	0	0
0	0	0	0	25	0	0
0	0	0	0	12	0	0
0	0	0	0	338	0	0
0	0	0	0	0	0	15
0	0	0	0	44	0	10
0	14	0	0	12	0	48
0	0	0	12	0	0	0
0	0	0	0	0	0	0
0	0	0	34	0	0	0
0	0	0	0	10	0	0
48	10	0	10	38	0	37
0	0	0	0	11	0	0
0	0	0	0	11	0	0
0	0	0	0	0	0	0
0	0	0	61	0	0	0
0	0	0	0	0	0	0
0	0	0	0	0	0	0
0	0	0	0	0	0	0
0	0	0	0	0	0	0
0	0	0	0	0	0	0
0	0	0	0	0	0	0
0	0	0	0	0	0	0
0	0	0	0	0	0	0
0	0	0	0	44	0	0
0	0	0	0	11	0	0
0	0	0	0	11	0	0
0	54	0	0	0	0	0
0	0	0	0	27	0	0
0	0	0	0	0	0	0
0	0	0	0	0	0	0
0	0	0	16	0	0	0
0	0	0	0	0	0	0
0	0	0	0	0	0	0
0	0	0	0	0	0	0
0	0	0	0	0	0	0
0	0	0	0	0	0	0

0	0	0	0	0	0	0
0	0	0	0	12	0	34
0	0	0	0	0	0	0
13	0	0	0	0	0	0
0	0	0	0	0	0	0
0	0	0	0	0	0	10
0	0	0	0	0	0	0
0	24	0	0	141	0	0
0	0	0	0	0	0	0
0	0	0	0	0	0	0
0	0	0	0	0	0	0
0	0	0	0	0	0	0
51	0	0	0	0	0	0
29	0	0	0	0	0	0
0	0	0	0	13	0	0
0	0	0	0	0	0	0
0	0	0	0	0	0	0
11	45	0	62	215	0	0
0	0	0	0	0	0	0
10	0	0	0	0	0	0
0	0	0	0	0	0	0
0	0	0	13	0	0	0
0	0	0	0	0	0	0
0	21	0	0	36	0	0
0	0	0	0	0	0	0
0	0	0	0	0	0	0
0	0	0	0	0	0	0
0	0	0	0	0	0	31
0	0	0	0	0	0	0
24	0	0	56	29	0	74
0	0	0	0	0	0	0
0	0	0	0	0	0	0
34	0	0	0	0	0	0
0	0	0	325	28	0	0
0	0	0	167	28	0	0
0	0	0	0	0	0	0
0	0	0	0	0	0	0
0	27	0	0	455	0	0
0	0	0	0	0	0	0
15	72	0	0	219	0	0
0	0	0	0	0	0	0
0	0	0	0	0	0	0
0	0	0	0	0	0	0
12	0	0	0	0	0	0
29	0	0	0	0	0	0
84	0	0	0	0	0	0

42	0	0	0	0	0	0
53	0	0	0	11	0	0
0	0	0	0	0	0	0
0	0	0	0	0	0	0
0	0	0	13	11	0	16
0	10	0	0	0	0	0
0	0	0	0	0	0	0
0	0	0	0	0	0	0
0	0	0	0	0	0	0
0	0	0	0	0	0	0
145	0	0	0	0	0	0
14	0	0	0	0	0	0
0	0	0	0	0	0	0
418	0	0	0	0	0	0
1266	0	0	0	0	0	0
0	0	0	0	0	0	0
0	0	0	0	0	0	0
11	0	0	0	0	0	0
0	0	0	0	0	0	0
0	0	0	0	0	0	0
0	0	0	0	0	0	0
0	0	0	0	173	0	12
0	0	0	0	0	0	0
0	0	0	0	0	0	0
0	0	0	0	0	0	0
10	0	0	0	0	0	0
0	10	0	0	27	0	0
0	0	0	0	800	0	0
0	0	0	0	0	0	0
0	0	0	0	0	0	0
0	0	0	0	0	0	0
11	0	0	0	0	0	0
0	0	0	0	0	0	0
0	0	0	0	0	0	0
0	0	0	0	0	0	0
0	0	0	0	0	0	0
28	11	0	0	0	0	0
0	0	0	0	0	0	0
0	0	0	0	0	0	0
0	0	0	0	0	0	0
0	0	0	0	0	0	0
0	0	0	0	0	0	0
0	0	0	0	0	0	0
22	0	0	0	0	0	0
0	0	0	0	0	0	0
0	0	0	0	0	0	0

0	0	0	0	0	0	0
27	0	0	0	0	0	0
0	0	0	0	0	0	0
68	0	0	0	0	0	0
0	0	0	0	0	0	0
0	0	0	0	0	0	0
0	0	0	0	0	0	0
0	0	0	0	0	0	0
0	0	0	0	0	0	0
0	0	0	0	0	0	0
125	0	0	0	0	0	0
12	0	0	0	0	0	0
0	0	0	0	0	0	0
0	0	0	0	0	0	11
0	0	0	0	0	0	0
0	0	0	20	0	0	0
0	0	0	0	0	0	0
0	0	0	0	0	0	0
0	0	0	152	0	0	0
0	0	0	0	0	0	23
0	0	0	0	0	0	0
0	0	0	0	0	0	0
0	0	0	32	0	0	0
0	0	0	0	0	0	15
0	0	0	186	0	0	0
0	10	0	0	0	0	0
0	11	0	0	0	0	0
0	16	0	0	0	0	0
0	28	0	0	11	0	0
0	12	0	0	0	0	0
0	28	0	0	0	0	0
0	13	0	0	0	0	0
0	24	0	0	0	0	0
0	15	0	0	0	0	0
0	16	0	0	0	0	0
0	11	0	0	0	0	11
0	23	0	0	0	0	0
0	11	0	0	91	0	0
0	14	0	86	0	0	0
0	11	0	0	0	0	0
0	10	0	0	28	0	0
0	13	0	0	0	0	0
0	44	0	0	0	0	0
0	16	0	0	0	0	0
0	11	0	0	0	0	0
0	15	0	0	0	0	0

0	10	0	14	0	0	0
0	10	0	0	0	0	0
0	54	0	0	0	0	0
0	11	0	0	0	0	0
0	22	0	33	0	0	0
0	47	0	0	0	0	0
0	86	0	0	0	0	0
0	15	0	0	0	0	0
0	21	0	0	44	0	0
0	10	0	0	35	0	0
0	17	0	0	15	0	0
0	10	0	0	0	0	19
0	60	0	0	0	0	0
0	0	0	0	12	0	0
0	0	0	0	0	0	0
0	0	0	0	0	0	0
0	0	0	0	0	0	0
0	0	0	0	0	0	0
0	0	0	0	0	0	0
0	0	0	0	0	0	0
0	0	0	0	0	0	0
0	0	0	0	0	0	0
0	0	0	0	0	0	0
0	0	0	0	0	0	0
0	0	0	29	0	0	0
0	0	0	0	0	0	0
0	0	0	0	0	0	0
0	0	0	11	0	0	13
0	0	0	0	0	0	0
0	0	0	0	0	0	0
0	0	0	0	0	0	0
0	0	0	0	0	0	0
0	0	0	0	0	0	0
0	0	0	0	0	0	0
0	0	0	0	0	0	0
0	0	0	0	0	0	0
0	0	0	0	11	0	0
0	0	0	0	0	0	0
0	0	0	0	0	0	0
0	0	0	0	0	0	0
0	0	0	0	29	0	0
0	0	0	0	0	0	0
0	0	0	0	0	0	0
0	0	0	0	0	0	0
0	0	0	0	0	0	0
0	0	0	0	0	0	0

0	0	0	0	0	0	0
0	0	0	0	0	0	0
0	0	0	0	10	0	0
0	0	0	0	19	0	0
0	0	0	0	0	0	28
0	0	0	0	0	0	0
0	0	0	0	0	0	0
0	0	0	0	0	0	0
0	0	0	0	0	0	0
0	0	0	0	0	0	0
0	0	0	0	0	0	0
0	0	0	0	10	0	0
0	0	0	0	0	0	0
0	0	0	0	0	0	0
0	0	0	0	0	0	0
0	0	0	0	0	0	0
0	0	0	35	0	0	0
0	0	0	0	0	0	0
0	0	0	0	0	0	0
0	0	0	0	0	0	0
0	0	0	0	0	0	0
0	0	0	0	0	0	0
0	0	0	0	0	0	0
0	0	0	0	10	0	0
0	0	0	0	0	0	0
0	0	0	0	10	0	0
21	0	0	0	14	0	0
0	0	0	0	0	0	0
0	0	0	0	0	0	0
0	0	0	0	0	0	0
0	0	0	0	23	0	0
0	0	0	0	0	0	0
0	0	0	0	0	0	0
11	0	0	0	0	0	0
28	0	0	0	0	0	0
0	0	0	0	0	0	0
0	0	0	0	0	0	0
0	0	0	0	0	0	0
0	0	0	0	11	0	0
0	0	0	0	0	0	0
0	0	0	0	0	0	0
0	0	0	0	0	0	0
0	0	0	0	0	0	0
0	0	0	0	0	0	0
0	0	0	0	0	0	0
0	0	0	0	0	0	0

0	0	0	0	0	0	0
0	0	0	0	44	0	0
0	0	0	0	0	0	0
0	0	0	0	0	0	0
0	0	0	0	0	0	0
0	0	0	36	0	0	0
0	0	0	0	65	0	0
0	0	0	0	0	0	0
0	0	0	0	0	0	0
0	0	0	0	0	0	0
0	0	0	0	0	0	0
0	0	0	0	0	0	0
0	0	0	0	0	0	0
0	0	0	0	0	0	0
0	0	0	0	0	0	0
0	0	0	0	0	0	0
0	0	0	0	0	0	0
0	0	0	0	0	0	0
0	0	0	0	0	0	0
0	0	0	0	0	0	0
0	0	0	0	0	0	0
0	0	0	0	63	0	0
0	0	0	0	0	0	0
0	0	0	0	0	0	0
0	0	0	0	0	0	0
0	0	0	0	0	0	0
0	0	0	0	0	0	0
0	0	0	0	0	0	0
0	0	0	0	0	0	0
0	0	0	0	0	0	0
0	0	0	0	0	0	0
0	0	0	0	0	0	0
0	0	0	0	0	0	0
0	0	0	0	0	0	0
0	0	0	0	0	0	0
0	0	0	0	0	0	0
0	0	0	0	0	0	11
0	0	0	0	0	0	0
0	0	0	0	0	0	0
0	0	0	0	0	0	0
0	0	0	0	0	0	0
16	0	0	0	0	0	0
0	0	0	0	0	0	12
0	0	0	0	0	0	0
0	0	0	0	0	0	0
0	0	0	0	0	0	0
0	0	0	0	0	0	0

0	0	0	0	116	0	0
0	0	0	0	0	0	0
0	0	0	0	0	0	0
11	0	0	0	0	0	0
0	0	0	0	42	0	0
0	0	0	0	21	0	10
0	0	0	0	0	0	0
0	0	0	0	0	0	0
0	0	0	0	10	0	0
0	0	0	0	0	0	0
0	0	0	0	0	0	0
0	0	0	0	0	0	0
0	0	0	0	0	0	0
14	0	0	0	0	0	0
17	0	0	0	0	0	0
0	0	0	0	0	0	0
0	0	0	0	0	0	0
0	0	0	0	0	0	0
0	0	0	0	0	0	0
0	0	0	0	15	0	0
0	0	0	0	0	0	0
0	0	0	0	68	0	0
0	0	0	0	25	0	0
0	0	0	0	38	0	0
0	0	0	0	0	0	0
0	0	0	0	0	0	0
0	0	0	0	0	0	0
0	0	0	0	0	0	0
0	0	0	0	10	0	0
0	0	0	0	35	0	11
0	0	0	0	0	0	0
0	0	0	0	0	0	0
0	0	0	0	50	0	0
0	0	0	11	0	0	0
0	0	0	94	94	0	0
0	0	0	0	0	0	0
0	0	0	0	0	0	0
0	0	0	0	0	0	0
0	0	0	0	0	0	0
0	0	0	0	0	0	0
0	0	0	0	0	0	42
0	0	0	0	0	0	0
0	0	0	0	0	0	0
0	0	0	11	0	0	0
0	0	0	0	0	0	0
0	0	0	0	0	0	0

0	0	0	0	0	0	0
0	0	0	0	0	0	0
0	0	0	0	0	0	0
0	0	0	0	0	0	0
0	0	0	0	0	0	0
0	0	0	0	0	0	0
0	0	0	0	0	0	0
0	0	0	0	0	0	0
0	0	0	0	0	0	0
0	0	0	0	0	0	11
0	0	0	0	0	0	0
0	0	0	0	0	0	0
0	0	0	0	0	0	0
0	0	0	0	0	0	0
0	0	0	0	30	0	0
0	0	0	0	0	0	0
0	0	0	0	0	0	0
0	0	0	0	0	0	0
0	0	0	0	0	0	10
0	0	0	0	0	0	0
0	0	0	0	0	0	0
0	0	0	0	0	0	0
0	0	0	0	0	0	0
0	0	0	0	0	0	0
0	0	0	0	0	0	0
0	0	0	0	0	0	0
0	0	0	0	0	0	0
0	0	0	0	0	0	0
0	0	0	0	0	0	0
0	0	0	0	0	0	0
0	0	0	10	0	0	0
0	0	0	0	0	0	0
0	0	0	0	0	0	0
0	0	0	121	0	0	0
0	0	0	0	0	0	0
0	0	0	0	0	0	0
0	0	0	0	11	0	0
0	0	0	0	13	0	0
0	0	0	0	0	0	0
0	0	0	0	31	0	0
0	0	0	0	17	0	0
0	0	0	0	0	0	0
0	0	0	43	0	0	0
0	0	0	0	0	0	0
0	0	0	0	0	0	0
0	0	0	0	0	0	0

0	0	0	10	0	0	0
0	0	0	0	0	0	0
0	0	0	26	0	0	0
0	0	0	0	0	0	0
0	0	0	0	100	0	0
0	0	0	0	20	0	0
0	0	0	0	25	0	0
0	0	0	0	0	0	0
11	0	0	34	0	0	0
0	0	0	21	0	0	0
0	0	0	0	40	0	0
0	0	0	0	53	0	0
0	0	0	0	0	0	0
0	0	0	11	10	0	0
0	0	0	0	0	0	0
11	0	0	0	0	0	0
0	0	0	0	60	0	0
0	0	0	0	10	0	0
0	0	0	0	0	0	0
0	0	0	0	0	0	0
0	0	0	0	0	0	0
0	0	0	0	0	0	0
0	0	0	0	0	0	0
0	0	0	0	0	0	21
0	0	0	13	0	0	0
0	0	0	0	0	0	0
0	0	0	0	0	0	0
0	0	0	0	0	0	0
0	0	0	0	0	0	0
0	0	0	0	0	0	0
0	0	0	0	0	0	0
0	0	0	0	0	0	0
0	0	0	0	14	0	0
0	0	0	0	0	0	0
0	0	0	86	0	0	0
0	0	0	10	0	0	0
0	0	0	0	0	0	0
0	0	0	55	0	0	0
0	0	0	0	0	0	0
0	0	0	0	0	0	0
0	0	0	0	0	0	0
0	0	0	0	0	0	0
0	0	0	0	0	0	0
0	0	0	0	0	0	0
0	0	0	0	0	0	0
0	0	0	0	0	0	0

0	0	0	0	0	0	0
0	0	0	0	0	0	0
0	0	0	0	0	0	0
0	0	0	0	0	0	0
0	0	0	0	0	0	0
0	0	0	0	0	0	0
0	0	0	0	0	0	0
0	0	0	0	0	0	0
0	0	0	0	0	0	0
0	0	0	0	0	0	0
0	0	0	0	0	0	0
0	0	0	0	0	0	0
0	0	0	0	0	0	0
0	0	0	0	0	0	0
0	0	0	10	0	0	0
0	0	0	0	314	0	0
0	0	0	0	0	0	0
0	0	0	0	0	0	0
0	0	0	0	0	0	0
0	0	0	0	0	0	25
0	0	0	0	0	0	0
0	0	0	0	0	0	0
0	0	0	0	0	0	0
0	0	0	0	0	0	0
0	0	0	0	0	0	0
0	0	0	0	0	0	0
0	0	0	0	0	0	0
0	0	0	0	0	0	0
0	0	0	0	0	0	0
0	0	0	0	0	0	0
0	0	0	0	0	0	0
0	0	0	0	0	0	0
0	0	0	0	0	0	0
0	0	0	0	0	0	0
0	0	0	0	0	0	0
0	0	0	0	0	0	0
0	0	0	0	0	0	0
0	0	0	0	0	0	0
0	0	0	0	0	0	0
0	0	0	0	0	0	0
0	0	0	0	0	0	0
0	0	0	0	0	0	0
0	0	0	0	58	0	0
0	0	0	0	0	0	0
0	0	0	0	0	0	0
0	0	0	0	0	0	0

0	0	0	11	0	0	0
0	0	0	0	0	0	0
0	0	0	10	0	0	0
0	0	0	0	0	0	0
0	0	0	0	0	0	0
0	0	0	0	0	0	0
0	0	0	0	0	0	0
0	0	0	0	0	0	0
0	0	0	0	0	0	0
0	0	0	0	0	0	0
0	0	0	0	0	0	0
0	0	0	0	0	0	0
0	0	0	0	0	0	0
0	0	0	0	0	0	0
0	0	0	0	0	0	0
0	0	0	0	0	0	0
0	0	0	0	37	0	0
0	0	0	0	0	0	0
0	0	0	11	0	0	0
0	0	0	0	0	0	0
0	0	0	0	0	0	0
0	0	0	0	0	0	0
0	0	0	0	0	0	0
0	0	0	0	0	0	0
0	0	0	0	0	0	0
0	0	0	0	0	0	0
0	0	0	0	0	0	0
0	0	0	0	0	0	0
0	0	0	0	0	0	0
0	0	0	0	0	0	0
0	0	0	0	0	0	0
0	0	0	0	0	0	0
0	0	0	0	0	0	0
0	0	0	0	0	0	0
0	0	0	0	0	0	0
0	0	0	26	0	0	0
0	0	0	0	0	0	0
0	0	0	0	0	0	0
0	0	0	0	0	0	0
0	0	0	0	0	0	0
0	0	0	0	0	0	0
0	0	0	0	0	0	0
0	0	0	0	0	0	0
0	0	0	0	0	0	0
0	0	0	0	0	0	0
0	0	0	0	0	0	0

0	0	0	0	0	0	0
0	0	0	0	49	0	0
0	0	0	0	0	0	0
0	0	0	0	0	0	0
0	0	0	0	0	0	0
0	0	0	0	0	0	0
0	0	0	14	0	0	0
0	0	0	0	10	0	0
0	0	0	0	0	0	0
0	0	0	0	0	0	0
0	0	0	25	0	0	0
0	0	0	0	0	0	0
0	0	0	0	0	0	0
0	0	0	12	0	0	0
0	0	0	0	0	0	0
0	0	0	0	0	0	0
0	0	0	0	0	0	0
0	0	0	0	0	0	0
0	0	0	0	0	0	0
0	0	0	0	0	0	0
0	0	0	0	0	0	0
0	0	0	0	0	0	0
0	0	0	0	0	0	0
0	0	0	0	0	0	0
0	0	0	0	0	0	0
0	0	0	0	0	0	0
0	0	0	0	0	0	0
0	0	0	0	0	0	0
0	0	0	0	0	0	0
0	0	0	0	0	0	0
0	0	0	0	0	0	0
0	0	0	0	0	0	0
0	0	0	0	0	0	0
0	0	0	0	0	0	0
0	0	0	0	0	0	0
0	0	0	0	0	0	0
0	0	0	0	0	0	0
0	0	0	30	0	0	0
0	0	0	0	0	0	0
0	0	0	0	13	0	0
0	0	0	0	0	0	0
0	0	0	0	0	0	0
0	0	0	0	0	0	0
0	0	0	0	0	0	0
0	0	0	0	0	0	0
0	0	0	0	0	0	0

0	0	0	0	0	0	0
0	0	0	0	16	0	0
0	0	0	0	0	0	0
0	0	0	33	0	0	0
0	0	0	0	0	0	0
0	0	0	0	0	0	0
0	0	0	0	0	0	0
0	0	0	0	0	0	0
0	0	0	0	0	0	0
0	0	0	0	10	0	0
0	0	0	0	0	0	0
0	0	0	0	0	0	0
0	0	0	0	0	0	0
0	0	0	0	0	0	0
0	0	0	0	0	0	0
0	0	0	0	0	0	0
0	0	0	0	0	0	0
0	0	0	0	0	0	0
0	0	0	0	0	0	13
0	0	0	0	0	0	0
0	0	0	0	0	0	0
0	0	0	0	0	0	0
0	0	0	0	0	0	0
0	0	0	0	0	0	0
0	0	0	0	0	0	0
0	0	0	0	0	0	0
0	0	0	0	0	0	0
0	0	0	0	0	0	0
0	0	0	0	0	0	0
0	0	0	0	0	0	0
0	0	0	0	0	0	0
0	0	0	0	0	0	0
0	0	0	0	0	0	0
0	0	0	0	0	0	0
0	0	0	0	0	0	0
0	0	0	21	0	0	0
0	0	0	0	0	0	0
0	0	0	10	0	0	0
0	0	0	0	0	0	0
0	0	0	0	0	0	0
0	0	0	0	0	0	0
0	0	0	0	0	0	0
0	0	0	0	0	0	0
0	0	0	0	0	0	0
0	0	0	0	0	0	0
0	0	0	0	0	0	0

0	0	0	0	0	0	0
0	0	0	0	0	0	0
0	0	0	0	0	0	0
0	0	0	18	0	0	0
0	0	0	0	0	0	0
0	0	0	34	0	0	0
0	0	0	0	0	0	0
0	0	0	0	0	0	0
0	0	0	0	0	0	0
0	0	0	0	0	0	0
0	0	0	0	0	0	0
0	0	0	0	0	0	0
0	0	0	0	0	0	0
0	0	0	0	0	0	0
0	0	0	10	0	0	0
0	0	0	129	0	0	0
0	0	0	0	0	0	0
0	0	0	0	0	0	0
0	0	0	0	0	0	0
0	0	0	0	0	0	0
0	0	0	0	0	0	0
0	0	0	0	0	0	0
0	0	0	0	0	0	0
0	0	0	0	0	0	0
0	0	0	0	0	0	0
0	0	0	0	0	0	0
0	0	0	0	0	0	0
0	0	0	0	0	0	0
0	0	0	0	0	0	0
0	0	0	0	0	0	0
0	0	0	0	82	0	0
0	0	0	0	0	0	0
0	0	0	43	0	0	0
0	0	0	0	0	0	0
0	0	0	0	0	0	0
0	0	0	0	20	0	0
0	0	0	0	0	0	0
0	0	0	0	0	0	0
0	0	0	0	0	0	0
0	0	0	0	0	0	0
0	0	0	58	0	0	0
0	0	0	0	0	0	0
0	0	0	0	0	0	0
0	0	0	0	0	0	0
0	0	0	0	0	0	0
0	0	0	0	0	0	0

0	0	0	0	0	0	0
0	0	0	0	0	0	0
0	0	0	0	0	0	0
0	0	0	0	0	0	0
0	0	0	0	0	0	0
0	0	0	0	0	0	0
0	0	0	0	0	0	0
0	0	0	0	0	0	0
0	0	0	0	0	0	0
0	0	0	16	0	0	0
0	0	0	12	0	0	0
0	0	0	0	0	0	0
0	0	0	103	0	0	0
0	0	0	0	14	0	0
0	0	0	0	71	0	0
0	0	0	0	0	0	0
0	0	0	0	0	0	0
0	0	0	0	10	0	0
0	0	0	0	0	0	0
0	0	0	0	0	0	0
0	0	0	0	0	0	0
0	0	0	0	25	0	0
0	0	0	0	0	0	0
0	0	0	0	0	0	0
0	0	0	0	0	0	0
0	0	0	0	0	0	0
0	0	0	0	0	0	0
0	0	0	0	0	0	0
0	0	0	0	14	0	0
0	0	0	10	0	0	0
0	0	0	0	0	0	0
0	0	0	0	0	0	0
0	0	0	0	0	0	0
0	0	0	0	0	0	0
0	0	0	0	0	0	0
0	0	0	0	0	0	0
0	0	0	0	0	0	0
0	0	0	0	0	0	0
0	0	0	0	0	0	0
0	0	0	0	0	0	0
0	0	0	0	0	0	0
25	0	0	0	0	0	0
10	0	0	0	0	0	0
14	0	0	0	0	0	0
33	0	0	0	0	0	0
10	0	0	0	70	0	0

60	0	0	0	0	0	0
367	0	0	0	0	0	0
21	0	0	0	0	0	0
11	0	0	0	0	0	0
12	0	0	0	0	0	0
118	0	0	0	0	0	0
0	0	0	0	0	0	0
0	0	0	0	0	0	0
0	0	0	0	0	0	0
0	0	0	53	0	0	0
0	0	0	0	0	0	0
0	0	0	0	11	0	0
0	0	0	0	11	0	0
0	0	0	0	0	0	0
0	0	0	0	0	0	0
0	0	0	0	0	0	0
0	0	0	0	11	0	0
0	0	0	0	0	0	0
0	0	0	0	0	0	0
0	0	0	0	0	0	0
0	0	0	0	0	0	0
0	0	0	0	0	0	0
0	0	0	0	11	0	0
0	0	0	0	14	0	0
0	0	0	0	0	0	0
0	0	0	0	0	0	0
0	0	0	0	0	0	0
0	0	0	0	0	0	0
0	0	0	0	10	0	0
0	0	0	0	0	0	0
0	0	0	0	11	0	0
0	0	0	0	0	0	0
0	0	0	0	0	0	0
0	0	0	0	0	0	0
0	0	0	0	42	0	0
0	0	0	0	0	0	0
0	0	0	0	0	0	0
0	0	0	0	0	0	0
0	0	0	0	0	0	0
0	0	0	0	0	0	0
0	0	0	0	0	0	0
0	0	0	0	38	0	0
0	0	0	0	0	0	0
0	0	0	0	0	0	0
0	0	0	0	0	0	0
0	0	0	0	0	0	0

0	0	0	0	75	0	0
0	0	0	0	14	0	0
0	0	0	0	14	0	0
0	0	0	0	13	0	0
0	0	0	0	11	0	0
0	0	0	0	31	0	0
0	0	0	0	21	0	0
0	0	0	0	10	0	0
0	0	0	0	10	0	0
0	0	0	0	14	0	0
0	0	0	0	11	0	0
0	0	0	0	104	0	0
0	0	0	0	43	0	0
0	0	0	0	163	0	0
0	0	0	0	355	0	0
0	0	0	0	11	0	0
0	0	0	0	11	0	0
0	0	0	0	14	0	0
0	0	0	0	12	0	0
0	0	0	0	12	0	0
0	0	0	0	10	0	0
0	0	0	0	14	0	0
0	0	0	0	10	0	0
0	0	0	0	34	0	0
0	0	0	0	28	0	0
0	0	0	0	13	0	0
0	0	0	0	10	0	0
0	0	0	0	13	0	0
0	0	0	0	12	0	0
0	0	0	0	10	0	0
0	0	0	0	34	0	0
0	0	0	0	25	0	0
0	0	0	0	12	0	0
0	0	0	0	50	0	0
0	0	0	0	15	0	0
0	0	0	0	11	0	0
0	0	0	0	10	0	0
0	0	0	0	20	0	0
0	0	0	0	11	0	0
0	0	0	0	0	0	0
0	0	0	0	0	0	0
0	0	0	0	0	0	0
0	0	0	0	0	0	0
0	0	0	0	0	0	0
0	0	0	0	0	0	0
0	0	0	0	0	0	0

0	0	0	0	0	0	12
0	0	0	0	0	0	0
0	0	0	0	0	0	0
0	0	0	0	0	0	0
0	0	0	0	0	0	0
0	0	0	0	0	0	0
0	0	0	0	0	0	0
0	0	0	0	0	0	0
0	0	0	0	0	0	0
0	0	0	0	0	0	0
0	0	0	0	0	0	0
0	0	0	0	0	0	0
0	0	0	0	0	0	0
0	0	0	0	0	0	0
0	0	0	0	0	0	0
0	0	0	0	0	0	0
0	0	0	0	0	0	0
0	0	0	0	0	0	0
0	0	0	0	0	0	0
0	0	0	0	0	0	0
0	0	0	0	0	0	0
0	0	0	0	0	0	0
0	0	0	10	0	0	0
0	0	0	10	0	0	0
0	0	0	0	0	0	0
0	0	0	0	0	0	0
0	0	0	35	0	0	0
0	0	0	0	0	0	0
0	0	0	0	0	0	0
0	0	0	0	0	0	0
0	0	0	0	0	0	0
0	0	0	11	0	0	0
0	0	0	0	0	0	0
0	0	0	0	0	0	0
0	0	0	0	0	0	0
0	0	0	0	0	0	0
0	0	0	0	0	0	0
0	0	0	0	0	0	0
0	0	0	0	0	0	0
0	0	0	0	0	0	0
0	0	0	0	0	0	0
0	0	0	0	0	0	0
0	0	0	0	0	0	0
0	0	0	0	0	0	0
0	0	0	0	0	0	0
0	0	0	0	0	0	0

0	0	0	0	0	0	0
0	0	0	0	0	0	0
0	0	0	0	0	0	0
0	0	0	0	0	0	0
0	0	0	0	0	0	0
0	0	0	0	0	0	0
0	0	0	0	0	0	0
0	0	0	0	0	0	0
0	0	0	0	0	0	0
0	0	0	0	0	0	0
0	0	0	0	0	0	0
0	0	0	0	0	0	0
0	0	0	0	0	0	0
0	0	0	0	0	0	0
0	0	0	0	0	0	0
0	0	0	0	0	0	0
0	0	0	0	0	0	0
0	0	0	0	0	0	0
0	0	0	0	0	0	0
0	0	0	0	0	0	0
0	0	0	0	0	0	0
0	0	0	0	0	0	0
0	0	0	0	0	0	0
0	0	0	0	0	0	0
0	0	0	0	0	0	0
0	0	0	0	0	0	0
0	0	0	0	0	0	0
0	0	0	0	0	0	0
0	0	0	0	0	0	0
0	0	0	11	0	0	0
0	0	0	14	0	0	13
0	0	0	0	0	0	0
0	0	0	0	0	0	0
0	0	0	0	0	0	0
0	0	0	0	0	0	0
0	0	0	0	0	0	0
0	0	0	0	0	0	0
0	0	0	0	0	0	0
0	0	0	0	0	0	0
0	0	0	0	0	0	0
0	0	0	0	0	0	0
0	0	0	0	0	0	0
0	0	0	0	0	0	0
0	0	0	0	0	0	0
0	0	0	0	0	0	0

0	0	0	0	0	0	0
0	0	0	0	0	0	0
0	0	0	0	0	0	0
0	0	0	0	0	0	0
0	0	0	0	0	0	0
0	0	0	0	0	0	0
0	0	0	0	0	0	0
0	0	0	0	0	0	0
0	0	0	0	0	0	0
0	0	0	10	0	0	0
0	0	0	0	0	0	0
0	0	0	0	0	0	0
0	0	0	0	0	0	0
0	0	0	0	0	0	0
0	0	0	0	0	0	0
0	0	0	0	0	0	0
0	0	0	0	0	0	0
0	0	0	0	0	0	0
0	0	0	0	0	0	0
0	0	0	0	0	0	0
0	0	0	0	0	0	0
0	0	0	0	0	0	0
0	0	0	0	0	0	10
0	0	0	0	0	0	10
0	0	0	0	0	0	39
0	0	0	0	0	0	10
0	0	0	0	0	0	170
0	0	0	0	0	0	10
0	0	0	0	0	0	14
0	0	0	0	0	0	10
0	0	0	0	0	0	97
0	0	0	0	0	0	43
0	0	0	0	0	0	0
0	0	0	0	0	0	0
0	0	0	0	0	0	0
0	0	0	0	0	0	0
0	0	0	0	0	0	0
0	0	0	0	0	0	0
0	0	0	0	0	0	0
0	0	0	0	0	0	0
0	0	0	0	0	0	0
0	0	0	0	0	0	0
0	0	0	0	0	0	0
0	0	0	0	0	0	0
0	0	0	0	0	0	0
0	0	0	0	0	0	0

0	0	0	0	0	0	0
0	0	0	0	0	0	0
0	0	0	10	0	0	0
0	0	0	32	0	0	0
0	0	0	14	0	0	0
0	0	0	31	0	0	0
0	0	0	23	0	0	0
0	0	0	10	0	0	0
0	0	0	48	0	0	0
0	0	0	34	0	0	0
0	0	0	22	0	0	0
0	0	0	10	0	0	0
0	0	0	0	0	0	0
0	0	0	0	0	0	0
0	0	0	0	0	0	0
0	0	0	0	0	0	0
0	0	0	0	0	0	0
0	0	0	0	0	0	0
0	0	0	0	0	0	0
0	0	0	0	0	0	0
0	0	0	0	0	0	0
0	0	0	0	0	0	0
0	0	0	0	0	0	0
0	0	0	0	0	0	0
0	0	0	0	0	0	0
0	0	0	0	0	0	0
0	0	0	0	0	0	0
0	0	0	0	0	0	0
0	0	0	0	0	0	0
0	0	0	0	0	0	0
0	0	0	0	0	0	0
0	0	0	0	0	0	0
0	0	0	0	0	0	0
0	0	0	0	0	0	0
0	0	0	0	0	0	0
0	0	0	0	0	0	0
0	0	0	0	0	0	0
0	0	0	0	0	0	0
0	0	0	0	0	0	0
0	0	0	0	0	0	0
0	0	0	0	0	0	0
0	0	0	0	0	0	0
0	0	0	0	0	0	0
0	0	0	0	0	0	0
0	0	0	0	0	0	0
0	0	0	0	0	0	0
0	0	0	0	0	0	0

0	0	0	0	0	0	0
0	0	0	0	0	0	0
0	0	0	0	0	0	0
0	0	0	0	0	0	0
0	0	0	0	0	0	0
0	0	0	0	0	0	0
0	0	0	0	0	0	0
0	0	0	0	0	0	0
0	0	0	0	0	0	0
0	0	0	0	0	0	0
0	0	0	0	0	0	0
0	0	0	0	0	0	0
0	0	0	0	0	0	0
0	0	0	0	0	0	0
0	0	0	0	0	0	0
0	0	0	0	0	0	0
0	0	0	0	0	0	0
0	0	0	0	0	0	0
0	0	0	0	0	0	0
0	0	0	0	0	0	0
0	0	0	0	0	0	0
0	0	0	0	0	0	0
0	0	0	0	0	0	0

regnant Horn						
PH_307	PH_324	PH_329	PH_331	PH_332	PH_333	PH_340
0	0	0	0	0	160	0
0	0	0	0	0	13	0
188	26	92	0	0	59	48
62	0	102	17	0	111	11
50	0	19	0	0	11	0
23	0	0	0	0	12	14
53	0	32	16	0	13	0
33	0	0	0	0	0	0
423	0	67	0	0	54	0
617	11	315	0	0	249	197
381	0	36	0	0	0	31
0	0	0	0	0	0	20
88	0	17	0	0	11	0
61	0	21	0	10	0	0
96	0	68	0	10	0	25
143	0	34	0	0	13	31
1796	1605	1188	216	245	1082	545
1160	77	355	28	65	11	41
13	42	0	0	0	11	0
55	31	161	11	0	81	148
0	0	0	0	0	40	0
32	0	0	0	0	34	0
19	0	0	0	0	11	13
0	0	0	0	0	0	0
1582	484	1029	214	160	1868	1381
655	240	162	0	11	413	266
276	1315	480	12	0	164	237
86	121	169	0	0	204	159
310	252	460	0	12	626	464
110	0	11	0	0	57	12
174	0	36	0	0	60	14
64	46	0	0	0	77	183
1343	5683	373	79	140	944	971
0	0	0	0	0	0	0
0	0	37	0	0	64	0
0	0	0	0	0	0	0
39	0	16	0	0	26	0
686	157	3928	41	58	632	200
6235	117	478	19	266	0	133
39351	3305	9698	344	284	1170	999
263	357	29	0	0	75	0

191	464	0	0	0	60	16
485	179	493	0	41	52	55
366	193	335	0	43	65	16
38334	678	5800	821	638	287	2013
854	214	1129	197	176	37	43
547	0	146	0	0	0	55
354	0	664	0	37	0	57
275	0	226	0	0	0	61
225	0	10	0	0	0	0
0	0	85	0	0	0	0
0	0	0	0	0	0	0
276	0	11	0	0	0	0
1101	0	147	0	18	50	284
32	0	33	0	0	0	0
43	0	0	0	0	0	0
0	0	251	0	0	0	77
274	0	23	0	0	0	27
2431	199	799	112	99	1086	537
1998	71	532	181	103	26	133
12	0	0	0	0	122	12
354	0	83	11	0	0	0
0	0	0	0	0	0	0
0	0	0	0	0	0	0
110	31	0	0	18	44	0
81	0	0	0	0	0	0
229	0	67	0	0	0	28
165	0	85	0	0	0	0
75	0	11	0	0	10	0
33	0	0	0	0	0	0
33	0	0	0	0	178	43
3203	133	904	0	0	0	446
5741	535	3195	306	280	573	1624
0	0	12	0	0	34	29
253	15	277	0	0	0	36
0	0	61	0	0	0	0
113	0	207	0	0	0	28
395	12	426	0	0	0	11
260	0	218	0	0	0	0
118	12	47	0	0	16	0
909	0	25	0	0	0	0
4162	13	574	0	28	0	212
40	25	37	0	10	33	31
46	0	186	0	0	0	0
37	0	92	0	0	0	0
857	103	371	0	0	0	160
14273	278	2933	120	271	293	2344

219	0	462	0	0	0	11
982	58	294	91	108	89	116
448	23	120	0	0	0	353
789	0	18	0	0	0	0
2836	423	2291	41	93	163	2957
12	0	0	0	0	92	10
0	0	0	0	0	0	0
22	0	0	0	0	0	0
0	0	30	0	0	0	0
17	0	13	0	0	0	0
39	0	44	78	0	141	48
119	10	30	0	12	0	0
5264	1228	3306	439	467	12122	4036
0	40	0	0	0	44	0
634	136	1233	66	33	2138	680
373	15	300	0	0	0	40
57	49	147	0	0	56	146
267	0	682	0	0	0	111
0	0	11	0	0	155	46
193	0	254	0	0	0	33
35	0	10	0	0	0	0
32	0	32	0	0	0	11
49	0	0	10	0	0	0
142	47	31	0	0	337	72
384	82	118	0	26	443	115
222	76	100	10	0	588	164
156	23	96	0	0	365	105
68	0	12	0	0	22	0
41	86	404	0	0	66	10
617	190	1871	88	0	1860	603
10	0	46	0	0	119	61
0	0	0	0	0	54	0
0	0	146	0	10	35	31
20	0	125	0	0	288	31
0	0	22	0	0	0	0
0	0	0	0	0	52	10
26	0	0	0	0	114	23
70	32	112	0	0	285	45
33	0	61	0	0	23	0
470	104	216	39	29	1563	259
31	13	0	0	0	84	0
31	0	0	0	0	143	28
1464	51	161	0	10	25	83
11135	2704	7586	464	1912	3063	2434
694	166	391	0	66	52	30
3852	2105	3327	11	411	474	199

0	0	0	0	0	54	0
148	33	35	0	0	676	635
408	63	93	21	0	843	649
8000	1570	828	323	223	1037	880
465	0	45	0	0	0	13
1510	110	111	14	32	125	194
0	0	0	0	0	0	0
592	36	46	0	0	0	12
34	0	38	0	0	0	0
0	0	0	0	0	0	0
213	0	69	0	0	127	88
56	0	33	0	20	200	37
144	0	104	0	0	0	0
82	0	0	0	0	86	233
316	49	42	0	0	473	1181
172	0	0	0	0	0	0
186	0	53	0	0	13	45
0	0	0	0	0	0	0
0	0	0	0	0	0	0
10	0	0	0	0	0	0
136	23	112	0	0	166	24
36	0	0	0	0	0	0
139	10	49	0	18	623	216
0	0	0	13	0	41	31
0	0	0	0	0	100	10
167	36	175	30	0	43	189
169	85	82	0	0	209	106
306	193	244	0	70	559	1008
18	0	61	0	0	92	36
69	39	96	0	0	47	120
46	0	11	0	0	0	0
84	116	94	0	12	137	190
249	33	82	12	13	128	167
0	0	46	0	0	75	11
170	96	30	24	15	123	1042
147	25	469	0	0	59	92
164	19	288	0	0	0	63
0	0	0	0	0	0	0
30	13	40	0	0	0	0
0	0	0	0	0	0	0
175	0	14	42	0	1248	0
10	0	0	0	0	0	0
14	0	111	0	0	0	14
0	0	0	0	0	0	0
161	0	12	23	0	159	29
66	0	0	44	0	69	0

91	0	0	0	0	23	0
169	0	33	0	189	114	0
1036	61	327	35	426	656	768
260	0	0	10	117	936	29
77	0	116	0	0	0	84
199	50	303	12	0	251	204
408	14	130	30	15	322	102
0	0	0	0	0	0	0
51	0	27	0	0	0	0
0	0	0	0	0	0	0
60	0	10	0	0	0	0
12	0	11	0	0	0	0
0	0	0	0	0	10	0
1307	163	1001	91	155	288	364
10	0	63	0	0	0	0
85	49	769	0	36	48	555
32	0	0	0	0	0	11
29	0	0	0	0	24	0
0	0	0	0	0	0	0
0	0	0	0	0	0	0
27	0	0	0	0	0	0
317	0	90	21	0	510	10
133	0	90	0	0	0	0
0	0	0	0	0	0	0
0	0	89	0	0	0	0
175	0	55	0	0	27	0
188	59	243	0	0	22	30
0	0	0	0	0	0	0
0	0	0	0	0	0	0
0	0	0	0	0	0	0
0	0	0	0	0	0	0
0	0	61	0	0	0	0
10	0	14	0	0	0	0
0	0	0	0	0	0	0
24	0	0	0	0	12	0
86	217	40	0	0	130	270
3870	2439	6472	24	27	4997	2772
1286	1995	4016	0	81	2101	1028
123	0	139	0	0	283	10
0	0	26	0	0	11	0
0	0	0	0	0	0	0
3542	198	4553	55	27	288	222
425	24	760	15	0	442	132
46	0	149	0	0	65	0
56	0	621	0	0	0	0
548	32	6160	68	14	35	196

710	0	1719	38	0	0	70
1582	60	2665	61	0	38	233
212	20	195	0	0	11	91
0	0	0	0	0	135	11
64	0	168	0	0	298	52
1365	1909	2904	14	93	1761	940
402	47	1056	13	0	372	15
104	85	25	0	0	75	48
135	64	48	12	0	0	10
0	0	0	0	0	0	0
39	0	667	0	0	0	28
77	16	0	0	0	0	0
73	0	0	0	0	0	0
436	121	1880	0	0	1095	126
331	107	78	119	0	0	35
0	0	38	0	0	0	0
0	0	130	0	0	0	0
44	0	441	0	0	0	0
715	561	1258	46	14	247	316
59	0	23	0	0	0	0
0	0	0	0	0	165	0
0	0	0	0	0	104	0
45	117	10	0	0	112	125
0	0	12	0	0	68	0
617	1617	2576	92	26	1495	995
285	46	563	0	0	145	11
37	0	91	0	0	0	0
1946	60	2687	0	11	520	214
229	32	311	0	0	576	49
133	10	53	0	0	288	39
363	0	906	0	0	0	0
96	0	73	0	0	338	48
10	0	35	0	0	0	0
0	67	0	0	0	79	0
1024	526	2021	39	40	154	239
156	161	190	0	0	48	12
102	38	118	0	0	43	0
10	0	0	0	0	65	0
0	0	0	0	0	0	0
256	0	445	10	0	446	147
47	0	28	0	0	290	73
272	0	97	10	0	109	48
98	0	20	0	0	0	0
1004	51	609	60	33	859	180
0	0	0	0	0	0	0
2138	285	294	11	75	1942	2444

5017	554	867	33	102	7026	2171
15	0	0	0	0	181	51
35	0	0	0	0	458	206
1646	1347	1336	39	94	1158	2663
280	47	65	23	0	85	112
145	50	10	0	0	185	11
28	128	16	0	0	159	90
526	21	68	13	34	1340	389
71	0	0	0	0	174	42
0	0	0	0	0	57	0
0	0	0	0	0	327	0
332	272	193	0	11	425	116
10499	729	1633	11	300	4597	907
173	199	95	0	0	722	46
159	77	35	0	16	180	37
338	421	256	27	116	298	70
781	732	403	45	245	1230	287
3239	12	378	15	74	0	13
862	0	143	12	39	37	36
2440	31	700	0	137	41	79
2689	0	223	14	80	27	12
348	255	222	0	11	45	64
949	970	728	62	69	261	337
543	162	381	44	51	56	123
583	265	294	0	17	52	53
128	38	0	0	0	202	79
12368	23936	5263	2194	1219	2250	2188
1265	2075	2018	353	187	176	330
427	800	302	26	26	63	49
102	119	10	105	0	127	107
2451	1074	195	50	295	3327	344
1214	3708	865	521	64	99	312
1919	4642	773	184	162	27	95
115	0	0	0	0	133	29
256	961	101	222	0	109	367
329	42	41	0	0	435	162
51	0	0	0	0	73	46
1338	3147	867	94	142	441	422
646	178	245	42	59	444	256
328	545	191	43	23	683	401
895	70	125	12	0	1799	1371
1469	146	262	12	0	1929	1100
156	0	127	0	0	0	29
259	459	244	0	26	97	60
330	0	117	23	0	532	55
76	0	48	0	0	51	0

338	0	188	0	0	590	82
334	0	120	66	10	105	23
53	0	0	0	0	367	82
397	12	105	0	0	146	78
33	0	16	0	0	0	0
11	0	0	0	0	13	0
33	10	81	0	0	158	74
44	0	77	0	0	39	48
0	0	0	0	0	59	0
38	0	0	13	0	40	0
0	0	0	0	0	0	0
164	0	69	0	0	626	45
0	0	40	0	0	0	0
10	0	0	0	0	200	22
0	0	0	0	0	0	0
10	0	0	0	0	0	0
0	0	0	0	0	44	0
0	0	155	0	0	78	0
1283	423	1512	228	47	1048	849
0	0	0	0	0	0	0
0	0	0	0	0	0	0
0	0	0	0	0	0	0
0	0	0	0	0	0	0
763	352	773	37	10	408	441
0	0	0	0	0	0	0
27	0	60	14	0	13	0
226	127	39	0	0	0	89
287	131	22	0	0	40	100
0	0	25	0	0	0	0
37	0	71	0	0	0	0
10	0	0	0	0	0	0
0	0	13	0	0	0	0
0	0	13	0	0	0	0
18	0	48	0	0	0	0
475	139	514	10	0	14	126
0	0	22	0	0	0	0
0	0	0	0	0	0	0
0	0	0	0	0	0	0
24	0	42	0	0	0	0
30	0	0	43	0	0	0
0	0	0	0	0	31	0
66	13	12	0	0	33	0
109	37	24	21	0	102	29
69	11	42	20	0	67	52
0	0	0	0	0	0	0
0	0	0	0	0	0	0

104	0	121	0	0	49	34
0	0	0	0	0	0	0
0	0	0	0	0	16	0
1494	214	596	70	108	4574	1013
23	0	133	0	0	27	0
0	0	135	0	0	0	10
0	0	66	0	0	31	0
13	0	48	0	0	20	0
0	0	24	0	0	12	0
86	64	0	0	0	26	55
111	10	871	0	16	111	16
372	49	889	116	19	359	219
146	0	765	0	0	66	34
304	45	703	85	15	458	205
59	0	225	0	0	0	34
0	0	0	0	0	0	0
0	0	0	0	0	0	0
36	15	111	0	0	19	10
0	0	0	0	0	0	0
0	0	0	0	0	0	0
73	0	81	0	0	56	24
0	0	0	0	0	0	0
27	0	59	0	0	70	0
123	0	871	0	0	131	36
0	0	0	0	0	0	0
0	0	0	0	0	0	0
11	0	42	0	0	49	0
0	0	0	0	0	0	0
581	129	207	38	20	1118	374
0	0	0	0	0	10	0
11	0	0	0	0	24	0
106	0	0	0	0	86	0
40	0	13	0	0	86	11
0	0	11	0	0	0	0
0	0	0	0	0	0	0
0	0	0	0	0	0	0
0	0	0	0	0	0	0
367	22	392	37	0	242	155
0	0	0	0	0	0	0
15	0	84	0	0	10	0
36	0	127	0	0	0	10
35	0	24	0	0	0	0
0	0	45	0	0	0	0
0	0	0	0	0	0	0
0	0	0	0	0	0	0
0	0	0	0	0	0	0

28	0	10	0	0	141	40
58	0	0	0	0	0	0
0	0	0	0	0	0	0
0	0	0	0	0	0	0
0	0	12	0	0	0	0
0	0	297	0	0	0	0
12	0	10	0	0	0	0
95	19	23	0	0	31	50
1412	323	1630	88	43	2989	1433
79	0	58	23	0	181	68
124	72	13	0	0	37	50
419	85	880	103	0	1084	247
0	0	32	0	0	0	0
25	0	0	0	0	0	0
20	20	74	0	0	78	0
1109	247	576	0	0	1981	341
12	0	58	0	0	258	0
0	0	0	0	0	0	0
10	0	12	0	0	10	0
0	0	0	0	0	40	11
0	0	0	0	0	0	0
0	0	0	0	0	35	0
0	0	0	0	0	0	0
125	12	199	0	0	742	113
2657	224	2635	0	0	225	246
1252	666	1806	28	21	1656	1079
118	95	256	0	0	152	270
49	143	11	0	0	24	165
507	358	209	0	46	125	244
21	0	0	0	0	0	0
0	0	0	0	0	0	0
19	0	27	0	0	0	0
36	99	0	0	10	32	47
65	73	0	0	0	56	116
130	195	81	21	0	271	699
28	0	11	0	0	34	0
2419	0	1094	0	10	0	34
24976	966	37686	338	275	869	3127
735	54	1817	10	13	0	57
11	0	22	0	0	0	0
3243	0	4132	0	25	201	445
222	0	49	0	0	0	0
153	13	54	0	0	0	27
121	0	391	0	0	0	12
0	0	0	0	0	0	0
234	21	1101	10	10	0	51

25	0	327	0	0	0	11
86	0	452	0	0	0	0
73	0	0	0	0	0	0
984	59	3203	11	33	30	165
11	11	19	0	0	91	22
92	83	102	0	0	84	285
78	21	252	0	0	189	69
86	110	224	0	0	90	227
43	12	66	0	0	83	39
99	65	59	0	0	0	39
1408	759	1090	0	62	339	424
104	0	641	0	0	0	32
0	0	0	0	0	0	0
34	0	40	10	0	0	0
0	0	0	0	0	25	61
0	0	0	0	0	11	0
0	0	0	0	0	0	0
12	0	0	0	0	33	0
198	0	74	12	10	335	51
0	0	0	0	0	0	0
74	0	0	0	0	111	10
0	0	0	0	0	21	0
389	10	1236	0	0	10	28
0	0	750	0	0	0	0
1302	43	1838	0	0	48	242
0	0	129	0	0	0	0
0	0	36	0	0	0	0
48	0	514	0	0	0	11
12	16	136	0	0	0	0
0	0	22	0	0	0	0
0	0	141	0	0	0	0
10	0	23	0	0	0	0
0	0	47	0	0	0	0
50	0	228	0	0	11	0
0	0	0	0	0	0	0
50	0	167	0	0	0	0
119	0	706	17	0	0	56
295	220	932	0	0	82	41
7988	1682	7242	1368	506	16781	6786
20	0	194	0	0	0	0
0	0	0	0	0	0	0
0	0	10	0	0	0	0
325	127	466	0	14	40	45
10	71	122	0	0	21	72
0	0	163	0	0	0	0
0	0	0	0	0	0	0

11	0	72	0	0	0	0
189	216	368	0	0	102	392
0	0	159	0	0	10	0
0	0	94	0	0	0	0
0	0	22	0	0	0	0
0	0	63	0	0	68	0
49	0	53	0	0	0	0
0	0	11	0	0	26	0
0	0	0	0	0	0	0
11	0	128	0	0	0	0
0	0	56	0	0	0	0
367	10	764	0	0	36	11
4784	1024	1802	225	145	6339	1324
552	51	191	135	18	818	320
65	10	710	0	0	0	20
0	0	59	0	0	0	0
0	0	41	0	0	0	0
29	0	0	0	0	0	0
150	0	59	0	0	151	12
1204	308	473	57	42	1993	600
605	115	279	0	14	748	315
106	50	104	12	0	253	81
257	48	653	10	0	45	96
0	0	50	0	0	0	0
0	0	0	0	0	54	0
0	0	27	0	0	0	0
37	0	211	0	0	0	0
578	99	320	0	0	1104	98
73	0	395	0	0	0	88
12	0	94	0	0	21	12
0	0	216	0	0	0	0
0	0	0	0	0	0	0
376	129	160	14	0	2350	585
10	0	0	12	0	59	0
12	0	15	0	0	57	21
0	0	32	0	0	0	0
164	26	96	0	0	471	111
21	0	0	0	0	143	0
74	0	91	0	0	472	443
266	79	176	0	0	900	276
0	0	0	0	0	132	155
10	0	24	0	0	0	12
0	0	30	0	0	0	12
950	644	2403	38	21	859	643
30	0	35	0	0	84	0
65	0	20	0	0	21	0

0	0	10	0	0	0	0
0	0	0	0	0	0	0
0	0	0	0	0	0	0
20	43	0	0	0	202	70
184	152	252	12	12	650	700
369	387	473	24	0	274	210
69	115	35	0	0	626	231
19	35	37	0	0	10	36
265	392	558	62	23	248	220
12	0	0	0	0	0	0
0	0	0	0	0	0	0
11	35	544	0	0	41	94
0	0	183	0	0	23	14
0	0	0	0	0	0	0
0	0	0	0	0	0	0
0	0	0	0	0	0	0
0	0	0	0	0	0	0
83	0	740	0	0	48	92
0	0	125	0	0	0	0
64	0	25	0	0	73	0
927	55	740	0	0	356	31
16	0	0	0	0	0	0
3206	257	991	0	15	2057	375
36	0	0	0	0	49	0
1984	206	596	68	0	2793	673
0	0	0	0	0	0	0
536	11	251	0	0	132	0
1063	50	295	39	0	1436	301
27	0	0	0	0	31	0
0	0	0	0	0	10	0
0	0	0	0	0	0	0
0	0	0	0	0	74	0
30	0	0	0	0	0	0
35	0	0	0	0	44	0
28	14	27	0	0	179	0
0	0	10	0	0	0	0
15	0	91	0	0	31	0
0	0	0	0	0	42	0
10	0	0	0	0	0	0
0	117	0	0	0	0	54
10	0	0	11	0	0	0
10	0	11	32	12	35	0
322	0	501	0	13	958	256
14	0	0	0	47	131	0
49	0	54	0	0	192	0
0	0	0	0	0	0	0

0	0	0	0	0	26	20
143	89	185	0	21	727	1692
0	0	10	0	0	331	528
59	0	175	0	0	69	68
0	0	0	0	0	0	0
0	0	60	0	0	10	52
22	0	137	0	0	10	0
102	0	28	0	0	74	108
1214	1719	1960	275	147	9396	1115
717	115	595	176	237	4475	927
286	57	350	23	78	1748	537
21	0	0	0	0	37	12
11	0	0	0	0	36	0
0	0	20	0	0	0	47
0	0	0	0	0	0	0
65	0	287	0	17	52	37
0	0	0	0	0	23	10
10	0	0	0	0	42	0
0	0	37	0	0	0	0
0	0	10	0	0	0	0
208	70	386	0	12	446	66
99	0	795	23	0	409	124
46	0	10	0	0	96	16
0	0	0	0	0	11	0
0	0	0	0	0	0	0
209	221	863	10	22	560	1540
3000	2744	5140	86	375	2948	4633
2191	2804	2333	11	447	2421	779
21608	14782	40358	1156	3475	10674	6292
95	293	432	17	0	149	60
169	705	444	30	0	187	32
120	0	50	12	0	146	75
5806	2749	2719	417	322	2603	1298
1680	3766	1464	85	127	1919	1378
35	123	14	0	0	191	110
2740	13	1018	0	0	0	0
1304	529	4308	224	284	2408	1734
1217	348	2504	10	46	1366	1156
1097	54	1174	41	129	1196	139
1423	77	1179	11	117	763	93
0	0	0	0	0	0	0
50	0	0	0	0	0	0
1043	201	6485	147	652	1139	643
54	0	373	0	0	75	0
0	0	0	0	0	0	0
419	280	710	11	72	1082	595

286	204	1277	0	18	663	1048
3551	1431	7310	1204	405	22555	22703
0	21	39	0	0	13	10
292	154	25	0	0	1281	23876
10	12	267	0	18	153	93
0	0	65	0	0	131	38
297	282	1941	24	0	2272	1002
80	51	512	13	13	407	80
0	0	0	0	0	0	0
42	18	60	0	0	85	79
29	103	364	0	16	120	244
42	29	287	0	0	61	404
0	0	29	0	0	0	0
0	0	0	0	0	0	0
251	0	102	0	0	244	31
2985	406	1074	48	10	568	466
171	98	812	39	19	124	130
399	28	346	0	0	44	38
130	0	70	0	0	18	0
15	0	106	0	10	0	0
129	62	366	0	0	139	23
0	0	35	0	0	0	0
0	0	0	0	0	0	0
11	0	70	0	0	0	0
55	97	120	0	0	220	481
0	0	0	0	0	0	0
0	0	0	0	0	0	0
0	0	0	0	0	0	0
0	0	80	0	0	0	0
75	0	13	0	0	42	0
12	0	10	0	0	29	0
0	0	0	0	0	0	0
50	0	0	0	0	787	44
234	178	0	0	0	505	498
11	0	372	34	0	82	0
13	0	202	26	0	287	25
401	205	375	45	23	98	13
78	129	633	67	0	38	0
556	450	1111	119	0	721	1611
35	0	0	20	0	117	10
50	0	10	13	0	130	17
25	0	465	29	0	28	135
0	0	0	0	0	0	0
65	13	251	0	0	12	34
12	0	0	0	0	0	12
37	0	378	0	0	29	49

0	0	12	0	0	0	0
0	0	153	0	0	11	114
55	0	110	10	0	106	53
0	0	0	0	0	25	0
0	0	0	0	0	1095	46
0	0	0	0	0	10	0
0	0	0	0	0	0	0
0	0	0	0	0	0	0
0	0	0	0	0	42	0
26	0	50	18	0	11	23
32	0	0	0	0	0	0
69	0	0	0	0	242	17
965	374	121	31	11	37	33
104	26	246	51	19	45	10
484	183	862	17	116	19	0
139	293	11	0	0	74	54
54	16	86	12	19	0	0
751	395	121	0	0	32	24
144	0	0	0	0	38	0
0	0	0	0	0	0	0
144	31	68	11	0	23	30
48	209	55	0	0	23	22
14	0	11	0	0	11	0
0	0	0	0	0	38	0
31	0	32	0	0	84	0
35	0	0	0	0	12	0
0	0	0	0	0	0	0
80	0	0	0	0	159	22
22	212	0	11	0	0	30
0	0	0	0	0	12	0
0	0	0	0	0	0	0
0	0	0	0	0	0	0
66	0	0	0	28	0	0
28	0	12	0	0	0	0
112	25	0	0	21	41	0
0	0	0	0	0	0	0
0	0	0	0	0	0	0
0	0	0	0	0	0	0
0	0	0	0	0	0	0
0	0	0	0	0	75	0
0	0	0	0	0	0	0
0	0	0	0	0	33	0
0	0	0	0	0	0	0
71	0	0	0	0	0	0
252	16	29	0	12	0	0
0	0	22	0	0	58	0

0	0	0	0	0	16	0
0	25	0	0	0	0	12
10	0	0	0	0	0	0
0	0	89	0	0	29	71
34	0	10	0	0	0	0
0	0	0	0	0	0	0
99	22	0	0	0	115	45
40	0	0	0	0	26	0
0	0	0	0	0	56	0
45	118	146	0	0	24	11
0	0	11	0	0	0	0
10	0	0	0	0	0	0
25	22	131	19	0	22	11
0	0	0	0	0	10	0
0	27	0	0	0	62	0
0	0	25	0	0	0	0
67	0	0	0	0	77	35
0	0	0	0	0	43	0
726	263	954	0	44	74	41
12	0	0	0	0	48	0
347	0	67	10	0	0	0
41	0	0	0	0	0	0
0	0	0	0	0	138	0
102	0	128	32	0	195	191
0	0	0	0	0	56	54
0	0	0	0	0	58	61
0	0	0	0	0	62	0
0	0	0	0	0	35	0
0	0	0	0	0	11	0
0	0	0	0	0	0	0
27	16	0	0	0	0	34
0	0	0	0	0	44	0
14	0	0	0	0	54	31
49	0	0	0	0	56	35
0	0	0	0	0	0	0
49	0	0	0	0	0	0
0	0	0	0	0	0	0
0	0	0	0	0	51	0
0	0	0	0	0	13	0
62	0	0	0	0	11	0
35	0	1344	49	26	36	118
0	0	0	0	10	0	36
16	0	242	0	0	0	0
0	0	0	0	0	0	0
47	0	28	0	0	0	0
0	0	0	0	0	0	0

22	0	0	0	0	0	0
0	0	0	0	0	0	0
199	0	0	0	0	52	0
0	0	0	0	0	0	0
685	0	10	0	10	28	0
598	24	323	0	178	94	391
0	0	0	0	0	0	0
50	0	718	0	0	0	0
0	0	0	0	0	0	0
0	0	0	0	0	0	0
0	0	0	0	0	56	0
0	0	0	0	0	24	10
111	10	0	0	0	22	0
0	0	0	0	0	0	0
0	0	0	0	0	0	0
0	0	0	0	0	41	0
0	0	0	0	0	87	0
28	0	11	0	0	82	0
88	16	47	0	0	0	0
0	0	0	0	0	0	0
0	0	0	0	0	26	0
0	0	0	0	0	40	0
0	0	0	0	0	108	0
265	0	0	0	0	31	0
125	0	12	0	0	54	0
55	360	634	0	0	96	82
19	0	0	0	0	13	0
76	0	0	0	0	108	0
41	177	55	0	15	49	26
0	0	0	0	0	83	0
27	0	0	0	0	126	0
0	29	0	0	0	44	94
0	0	0	0	0	0	0
157	639	486	0	0	78	37
0	0	118	0	0	83	0
0	0	0	0	0	47	0
0	0	0	0	0	27	0
11	0	0	0	0	47	0
11	0	14	0	0	20	0
18	0	0	0	0	109	0
45	98	0	0	0	0	0
159	32	412	0	0	0	0
62	0	0	0	0	90	0
0	0	44	0	0	26	0
0	0	0	0	0	0	0
220	0	188	0	0	99	14

100	0	0	0	0	164	0
10	0	0	0	0	0	0
0	0	0	0	0	0	0
0	0	0	0	0	135	10
143	60	0	0	0	50	11
26	0	0	0	0	14	0
65	0	0	0	0	0	0
16	16	0	0	0	71	0
0	0	0	0	0	0	0
0	0	0	0	0	67	0
374	0	0	0	0	124	11
68	0	0	0	0	44	14
224	44	32	0	0	293	54
0	0	0	0	0	115	0
104	31	22	0	0	58	89
14	10	0	0	0	67	0
95	0	0	0	0	98	22
0	0	0	0	0	683	308
700	0	13	11	0	267	801
0	0	0	0	0	25	0
0	0	0	0	0	64	0
16	0	73	0	0	225	0
0	0	0	0	0	184	0
0	0	0	0	0	421	41
14	0	0	0	0	192	0
0	0	0	0	0	61	0
200	207	60	0	35	165	26
63	96	88	0	0	41	0
175	151	108	0	12	125	0
0	0	0	0	0	11	0
595	0	21	0	10	10	0
1005	0	165	0	15	0	0
109	0	17	0	0	0	0
1429	0	256	0	53	0	0
0	0	0	0	0	0	0
28	0	0	0	15	10	0
189	0	24	0	0	0	0
150	25	25	0	0	93	0
115	11	0	0	0	13	15
10	0	0	0	0	10	0
515	46	0	0	0	160	62
43	10	0	0	0	96	11
64	0	65	0	0	196	0
46	0	34	0	0	53	0
0	0	0	0	0	79	0
0	0	0	0	0	23	0

32	119	0	0	16	0	0
382	93	0	0	0	134	68
11	0	0	0	0	34	0
27	10	0	0	0	16	0
0	0	0	0	0	0	0
0	23	0	0	0	23	0
51	0	0	0	0	0	0
0	0	0	0	0	0	0
60	33	0	27	10	254	41
187	25	206	0	19	37	68
98	0	0	0	0	250	135
44	0	114	0	0	26	0
0	0	0	0	0	0	0
0	0	0	0	0	28	0
0	0	0	0	0	0	0
10	0	0	0	0	40	0
41	44	71	0	0	74	99
12	0	0	0	0	120	25
24	0	0	0	0	134	49
25	0	0	0	0	0	11
103	205	64	0	0	14	0
0	0	0	0	0	154	0
0	0	0	0	0	0	0
0	0	0	0	0	125	0
0	0	0	0	0	20	0
17	11	24	0	0	28	0
15	0	18	17	0	19	0
0	0	0	0	0	58	0
0	0	0	0	0	74	0
0	0	0	0	0	11	0
0	0	0	0	0	120	0
19	0	0	56	0	341	18
0	0	0	24	0	75	0
0	0	0	0	0	11	0
0	0	0	0	0	68	0
0	0	0	0	0	18	0
126	12	59	0	0	32	0
160	37	233	0	11	164	101
10	0	0	0	0	12	0
41	0	41	0	0	45	26
0	0	0	0	0	0	0
89	0	10	0	0	76	22
0	0	0	0	0	38	0
0	0	0	0	0	13	0
0	0	0	10	0	26	0
43	0	0	0	0	17	0

0	0	0	0	0	0	0
0	0	0	0	0	0	0
35	0	30	0	0	37	64
0	0	0	0	0	13	0
34	0	26	0	0	50	0
24	0	0	0	0	48	0
0	0	0	0	0	40	0
46	71	68	0	0	66	1830
0	0	0	0	0	0	0
46	10	10	0	0	17	0
99	0	15	0	0	108	45
0	0	0	0	0	28	0
13	0	0	0	0	63	0
0	0	0	0	0	10	0
28	0	0	0	0	43	0
0	0	0	0	0	11	0
0	0	0	0	0	0	0
0	0	0	0	0	0	0
11	0	0	0	0	29	0
19	0	16	0	0	72	29
0	0	0	0	0	0	0
0	0	0	0	0	0	0
0	0	0	0	0	0	0
0	14	0	0	0	11	29
0	0	0	0	0	0	0
0	0	0	0	0	0	0
0	0	0	0	0	59	0
41	0	0	0	0	14	65
0	0	10	0	0	16	0
38	0	0	0	0	0	0
12	0	23	0	0	12	0
27	0	0	0	0	43	10
0	0	0	0	0	41	0
0	0	0	0	0	0	0
0	0	370	0	0	179	177
32	0	149	35	0	0	13
0	0	10	0	0	20	12
0	0	0	0	0	0	0
54	40	83	0	0	317	490
19	0	22	0	0	68	0
0	0	20	0	0	12	0
12	0	0	0	0	141	31
0	0	10	0	0	0	0
0	0	0	0	0	27	0
45	43	51	0	0	193	89
0	0	0	0	0	31	33

70	0	0	0	0	115	126
0	0	0	0	0	0	0
0	0	0	0	0	0	0
93	11	0	53	0	55	0
0	0	0	0	0	41	0
38	0	10	0	0	45	10
26	0	0	0	0	0	0
83	0	21	0	0	49	0
0	0	57	0	0	11	0
0	0	21	0	0	0	0
0	0	74	0	0	39	0
13	0	10	0	0	27	10
85	0	90	0	0	0	0
89	0	0	0	0	81	0
272	0	208	0	0	115	12
13	0	0	0	0	70	10
61	0	0	0	0	0	0
34	0	0	0	0	177	0
0	0	0	0	0	88	0
0	0	0	25	0	11	0
0	0	0	0	0	0	0
0	0	0	0	0	0	0
20	0	0	0	0	366	0
36	54	0	0	0	26	284
47	0	0	0	0	83	27
99	441	43	11	0	166	394
0	0	0	0	0	0	0
0	0	0	0	0	37	43
0	0	0	0	0	45	0
0	0	0	0	0	220	0
0	0	0	0	0	0	0
0	0	0	0	0	31	0
0	0	0	0	0	34	0
0	0	0	0	0	34	0
0	0	0	0	0	0	0
18	0	0	0	0	29	0
0	0	0	0	0	68	0
0	0	0	0	0	0	0
0	0	0	0	0	0	0
0	0	0	0	0	0	0
259	113	1021	10	73	163	185
0	0	0	0	0	167	0
0	0	0	0	0	74	20
33	0	12	0	0	27	0
0	0	0	0	0	73	0
0	0	0	0	0	41	0

0	0	0	0	0	82	12
0	0	0	0	0	0	0
23	0	0	12	19	462	144
0	11	57	0	0	11	10
0	0	0	0	0	332	37
16	0	12	0	0	90	220
0	0	0	0	0	120	0
21	0	0	0	0	65	33
10	0	15	0	0	138	64
0	0	0	0	0	0	0
10	0	22	10	0	55	0
0	0	0	0	0	10	0
0	0	11	0	0	0	0
62	0	0	0	0	10	0
95	309	62	0	0	78	1501
15	0	0	0	0	84	64
13	0	12	0	0	0	121
43	147	50	0	0	78	677
0	0	0	0	0	0	0
11	0	102	0	0	33	0
0	18	81	0	0	28	11
86	79	227	0	0	448	217
0	0	0	0	0	0	0
0	0	0	0	0	10	0
0	0	0	0	0	15	0
0	0	0	0	0	0	0
0	0	0	0	0	11	0
461	195	1178	52	72	59	45
0	38	0	0	0	66	0
0	12	0	0	0	0	0
133	191	0	0	0	170	137
19	0	26	0	0	41	0
127	34	0	0	30	105	34
0	0	0	0	0	0	0
12	0	0	0	0	198	0
0	0	0	0	0	47	0
0	0	0	0	0	87	0
12	110	494	0	0	44	55
17	0	228	0	12	0	0
0	0	0	0	0	10	0
0	0	51	10	0	44	106
17	10	169	0	0	115	58
12	0	0	0	0	10	0
0	0	12	0	0	373	80
0	0	72	0	0	142	11
0	0	0	0	0	13	0

0	0	0	0	0	333	124
14	0	11	0	0	41	10
0	0	0	0	0	109	0
0	0	0	0	0	109	0
0	0	0	10	0	33	0
11	0	0	19	0	149	0
43	0	35	0	0	0	0
0	0	0	0	0	60	0
15	0	168	0	0	35	28
0	22	36	0	0	12	13
63	0	122	0	0	14	0
0	0	100	0	0	104	67
0	0	0	0	0	81	0
0	0	0	0	0	16	0
41	0	61	0	0	49	0
35	0	0	0	0	152	0
0	0	0	0	0	63	0
0	0	0	0	0	12	0
206	75	137	10	22	37	26
0	88	0	0	0	0	11
361	0	33	0	0	0	10
0	0	0	0	0	16	40
0	0	30	0	0	0	41
0	0	0	0	0	0	41
0	0	0	0	0	0	27
10	0	0	0	0	0	10
0	0	0	0	0	0	56
0	0	0	0	0	0	51
0	12	0	0	0	42	75
16	0	0	0	0	0	41
0	0	0	0	0	0	36
0	0	0	0	0	65	201
281	63	0	0	0	44	30
98	0	0	0	0	0	11
15	0	0	0	0	0	10
27	0	28	0	0	0	35
13	0	0	0	0	14	37
46	0	0	0	0	0	15
0	0	0	0	0	0	197
0	10	0	0	0	10	61
11	0	0	0	0	0	76
0	0	0	0	0	0	12
0	0	0	0	0	23	51
0	0	0	0	0	0	12
23	0	77	0	0	0	257
0	0	12	0	0	30	11

25	0	0	0	0	0	10
0	18	0	0	0	0	21
0	14	0	0	0	0	419
0	61	901	0	0	16	51
0	0	61	0	0	0	95
0	0	0	0	0	0	275
0	0	0	0	0	0	13
0	0	0	0	0	13	90
14	0	13	0	0	20	21
0	0	0	0	0	31	10
0	0	0	0	0	0	140
0	0	0	0	0	0	44
0	0	0	0	0	0	10
219	0	0	0	0	0	0
26	44	0	0	0	0	0
0	0	0	0	0	0	0
0	0	0	0	0	0	0
57	0	0	0	0	0	0
0	0	0	0	0	0	0
126	0	93	0	0	0	0
106	0	10	0	0	0	0
44	22	38	0	0	0	0
11	0	16	0	0	0	0
0	15	40	0	0	18	0
0	0	0	0	0	0	0
326	0	422	0	21	0	0
248	162	50	0	0	54	0
72	0	62	0	0	0	0
126	0	53	0	0	0	0
0	0	0	0	0	0	0
0	0	15	0	0	0	0
0	0	0	0	0	0	0
0	40	0	0	0	0	0
79	0	196	0	0	0	0
0	0	150	0	0	0	0
0	0	13	0	0	0	0
0	0	188	0	0	0	0
0	0	0	0	0	0	0
34	0	59	0	0	0	0
10	0	0	0	0	0	0
198	0	0	0	11	0	0
0	0	0	0	0	0	0
0	0	0	0	0	0	0
0	0	0	0	0	0	0
0	0	11	0	0	0	0
13	0	85	0	0	11	0

50	0	113	0	0	0	0
0	0	292	0	20	0	0
0	0	0	0	0	0	0
13	0	77	0	0	0	0
12	0	119	0	0	0	0
0	0	0	0	0	0	0
0	0	0	0	0	11	0
25	0	0	0	0	10	0
41	0	0	0	0	73	0
311	0	0	0	0	58	0
0	0	0	0	0	0	0
94	0	42	0	0	0	0
11	0	0	0	0	0	0
0	0	0	0	0	0	0
0	0	0	0	0	0	0
0	0	0	0	0	0	0
0	36	30	0	0	0	0
0	0	0	0	0	0	0
92	0	10	0	0	0	0
0	0	0	0	0	0	0
0	0	0	0	0	0	0
0	11	0	0	0	0	0
0	0	0	0	0	0	0
0	0	0	0	0	0	0
0	0	0	0	0	0	0
0	0	88	0	0	0	0
0	0	0	0	0	0	0
120	351	484	0	39	0	0
0	0	0	0	0	0	0
31	10	444	0	36	0	0
0	0	24	0	0	0	0
0	0	0	0	0	0	0
0	0	0	0	0	0	0
21	0	61	0	0	0	0
0	0	0	0	0	0	0
11	0	0	0	0	0	0
0	0	0	0	0	0	0
0	0	0	10	0	11	0
0	0	0	0	0	0	0
0	0	0	0	0	0	0
0	0	0	0	0	0	0
0	0	0	0	0	0	0
0	0	0	0	0	27	0
0	0	0	0	0	0	0
0	0	0	0	0	47	0
0	0	0	0	0	78	0

0	0	0	0	0	0	0
0	0	0	0	0	24	0
0	0	0	0	0	0	0
0	0	0	0	0	0	0
0	0	0	0	0	20	0
0	0	0	0	0	0	0
0	0	0	0	0	10	0
0	0	0	0	0	0	0
0	0	0	0	0	0	0
0	0	0	0	0	25	0
0	0	0	0	0	0	0
0	0	0	0	0	38	0
12	0	0	0	0	48	0
26	0	0	0	0	0	0
29	0	0	0	0	0	0
0	0	0	0	0	0	0
0	0	0	0	0	0	0
0	0	0	0	0	0	0
0	0	0	0	0	0	0
0	0	0	0	0	0	0
0	0	0	0	0	0	0
0	0	0	0	0	0	0
0	0	0	0	0	0	0
0	0	0	0	0	33	0
0	0	0	0	0	0	0
0	0	0	0	0	0	0
0	0	0	0	0	0	0
0	0	0	0	0	0	0
0	0	0	0	0	0	0
0	0	0	0	0	0	0
0	0	0	0	0	0	0
0	0	0	0	0	0	0
37	0	82	0	0	0	0
0	0	0	0	0	0	0
0	0	0	0	0	0	0
0	0	0	0	0	0	0
0	0	0	0	0	0	0
0	0	0	0	0	0	0
0	0	11	0	0	0	0
0	0	0	0	0	0	0
0	0	0	0	0	0	0
0	0	0	0	0	0	0
47	0	0	0	0	45	0
0	0	0	0	0	0	0
0	0	0	0	0	0	0
0	0	0	0	0	0	0

0	0	0	0	0	12	0
0	0	0	70	0	34	0
0	0	0	0	0	0	0
0	0	0	0	0	0	0
0	0	0	0	0	0	0
0	0	0	0	0	0	0
0	0	0	0	0	0	0
0	0	0	0	0	10	0
0	0	11	0	0	30	0
0	0	0	0	0	0	0
0	0	0	0	0	11	0
0	0	0	0	0	0	0
0	0	0	0	0	14	0
0	0	0	0	0	0	0
0	0	0	0	0	0	0
0	0	0	0	0	0	0
0	0	21	0	0	10	0
0	0	0	0	0	24	0
0	0	0	0	0	0	0
0	0	0	0	0	0	0
0	0	0	0	0	0	0
0	0	0	0	0	0	0
0	0	0	0	0	0	0
0	0	0	0	0	0	0
0	0	0	0	0	0	0
0	0	0	0	0	0	0
0	0	0	0	0	10	0
10	0	0	0	0	0	0
0	0	0	0	0	0	0
0	0	37	0	0	0	0
0	0	0	0	0	0	0
33	0	12	0	0	0	0
0	0	0	0	0	30	0
0	0	0	0	0	10	0
0	0	0	0	0	0	0
0	0	0	0	0	0	0
0	0	0	0	0	0	0
0	0	0	0	0	0	0
0	0	0	0	0	13	0
0	0	0	0	0	32	0
0	0	0	0	0	0	0
0	0	0	0	0	0	0
0	0	0	10	0	0	0
0	0	0	0	0	0	0
23	0	0	0	0	0	0
0	0	0	0	0	0	0

0	0	0	0	0	0	0
0	0	0	0	0	0	0
113	0	11	0	0	0	0
224	13	10	0	0	0	0
0	0	0	0	0	0	0
0	0	0	0	0	0	0
0	0	0	0	0	0	0
0	0	0	0	0	0	0
0	0	0	0	0	0	0
0	0	0	0	0	0	0
0	0	0	0	0	0	0
0	0	0	0	0	0	0
0	0	0	0	0	0	0
0	0	0	0	0	0	0
0	0	0	0	0	0	0
39	0	49	0	0	0	0
14	0	0	0	0	0	0
0	0	10	0	0	0	0
0	0	0	0	0	0	0
22	10	0	0	0	0	0
0	0	22	0	0	0	0
24	0	21	0	0	0	0
48	0	0	0	0	0	0
561	0	12	0	10	0	0
0	0	0	0	0	0	0
0	0	0	0	0	20	0
0	0	0	0	0	0	0
38	0	32	0	0	0	0
0	0	0	0	0	0	0
0	0	0	0	0	0	0
28	0	69	0	0	0	0
0	0	0	0	0	0	0
10	0	25	0	0	0	0
0	0	0	0	0	0	0
31	0	0	0	0	0	0
0	0	0	0	0	0	0
10	0	0	15	0	14	0
0	0	0	0	0	0	0
0	0	0	0	0	0	0
21	0	126	0	0	14	0
0	0	0	0	0	0	0
0	0	0	0	0	0	0
280	29	236	11	0	0	0
0	0	28	0	0	0	0
0	0	0	0	0	0	0
0	0	90	0	0	0	0

0	0	31	0	0	0	0
108	0	52	0	0	0	0
10	73	0	0	0	17	0
0	0	0	0	0	10	0
0	0	0	0	0	0	0
0	0	0	0	0	0	0
0	0	0	0	0	0	0
0	0	0	0	0	0	0
0	0	0	0	0	0	0
0	0	0	0	0	0	0
0	0	0	0	0	0	0
13	0	0	0	0	21	0
0	0	0	0	0	0	0
33	0	0	0	0	0	0
37	0	0	0	0	67	0
10	0	0	0	0	121	0
14	11	0	0	0	0	0
0	0	0	0	0	0	0
16	0	0	0	0	0	0
34	0	0	0	0	0	0
0	0	0	0	0	0	0
14	0	0	0	0	22	0
0	0	31	0	0	0	0
0	0	30	0	0	0	0
0	0	0	0	0	0	0
0	0	21	0	0	18	0
0	0	0	0	0	0	0
0	0	0	0	0	0	0
0	0	0	0	0	0	0
0	0	0	0	0	22	0
66	0	0	28	0	0	0
0	68	0	0	0	0	0
0	0	13	0	0	0	0
13	0	0	0	0	0	0
11	0	0	0	0	0	0
0	0	0	0	0	0	0
0	0	0	0	0	0	0
13	0	11	0	0	12	0
0	0	0	0	0	0	0
0	0	0	0	0	0	0
0	0	0	0	0	0	0
0	0	0	0	0	0	0
0	0	0	0	0	0	0
0	0	0	0	0	0	0
0	0	0	0	0	0	0
0	0	51	0	0	0	0

0	0	0	0	0	11	0
0	0	0	0	0	0	0
0	0	0	0	0	0	0
0	0	0	0	0	0	0
0	0	0	0	0	31	0
0	0	0	0	0	0	0
0	0	0	0	0	0	0
0	0	11	0	0	0	0
27	0	0	0	0	0	0
0	0	0	0	0	0	0
12	0	0	0	0	0	0
0	0	0	0	0	0	0
30	0	0	0	0	0	0
0	0	0	0	0	0	0
0	0	0	0	0	0	0
0	0	0	0	0	0	0
0	0	0	0	0	27	0
0	0	0	0	0	0	0
0	0	0	0	0	0	0
0	0	55	0	0	0	0
25	0	57	11	0	43	0
0	0	0	0	0	0	0
0	0	0	0	0	0	0
0	0	0	0	0	0	0
0	0	0	0	0	0	0
0	0	0	0	0	0	0
0	0	0	0	0	0	0
0	0	0	0	0	0	0
0	0	0	0	0	0	0
0	0	0	0	0	0	0
0	0	0	0	0	0	0
0	0	11	0	0	0	0
0	0	0	0	0	0	0
0	0	30	0	0	0	0
0	0	21	0	0	0	0
0	0	52	0	0	0	0
0	0	0	0	0	10	0
0	0	80	0	0	0	0
0	0	0	0	0	0	0
0	0	0	0	0	0	0
0	0	0	0	0	0	0
0	0	17	0	0	0	0
0	0	32	0	0	0	0
0	0	0	0	0	0	0
0	0	0	0	0	0	0
0	0	0	0	0	0	0

0	0	0	0	0	0	0
0	0	0	0	0	0	0
0	0	0	0	0	0	0
0	0	0	0	0	0	0
0	0	13	0	0	0	0
41	0	0	0	0	0	0
0	0	0	0	0	0	0
0	0	0	0	0	0	0
0	0	0	0	0	0	0
65	13	0	0	0	0	0
0	0	0	0	0	0	0
0	0	11	0	0	0	0
0	0	0	0	0	0	0
0	0	0	0	0	0	0
0	0	0	0	0	0	0
0	0	0	0	0	0	0
55	0	0	0	0	0	0
0	0	0	0	0	0	0
0	0	0	0	0	0	0
0	0	0	0	0	0	0
0	0	0	0	0	0	0
0	0	0	0	0	0	0
0	0	0	0	0	0	0
0	0	31	0	0	0	0
0	0	0	0	0	0	0
0	0	0	0	0	0	0
0	0	0	0	0	0	0
0	0	0	0	0	0	0
0	0	0	0	0	0	0
0	0	0	0	0	29	0
13	0	47	0	0	0	0
0	0	0	0	0	0	0
0	0	0	0	0	0	0
0	0	0	0	0	0	0
10	0	0	0	0	0	0
34	0	0	0	0	0	0
0	0	0	0	0	0	0
0	0	0	0	0	0	0
0	0	0	0	0	0	0
0	0	0	0	0	0	0
0	0	0	0	0	0	0
11	0	0	0	0	0	0
0	0	0	0	0	0	0
0	0	0	0	0	0	0
0	0	0	0	0	0	0
0	0	0	0	0	0	0

10	0	0	0	0	0	0
0	0	0	0	0	0	0
11	0	0	0	0	0	0
0	0	0	0	0	0	0
0	0	10	0	0	0	0
0	0	0	0	0	0	0
27	0	0	11	0	0	0
0	0	0	0	0	0	0
0	0	0	0	0	0	0
0	0	0	0	0	0	0
0	0	0	0	0	0	0
14	0	0	0	0	11	0
0	0	0	0	0	0	0
0	0	0	0	0	0	0
0	0	0	0	0	0	0
0	0	0	0	0	0	0
0	0	0	0	0	0	0
10	0	0	0	0	55	0
0	0	35	0	0	0	0
0	0	0	0	0	0	0
0	0	0	0	0	0	0
11	0	60	0	0	0	0
0	0	0	0	0	0	0
125	0	29	0	0	0	0
0	0	0	0	0	0	0
0	0	0	0	0	0	0
0	0	0	0	0	0	0
0	0	0	0	0	0	0
0	0	0	0	0	0	0
0	0	0	0	0	0	0
0	0	0	0	0	0	0
11	0	0	0	0	0	0
0	0	0	0	0	0	0
0	0	0	0	0	0	0
0	0	0	0	0	0	0
0	0	0	0	0	0	0
0	0	0	0	0	0	0
0	0	0	0	0	0	0
0	0	0	0	0	0	0
0	0	0	0	0	0	0
0	0	0	0	0	0	0
0	0	0	0	0	0	0
0	0	0	0	0	0	0
0	0	0	0	0	0	0
0	0	0	0	0	0	0
0	0	0	0	0	0	0

0	0	28	0	0	0	0
0	0	0	0	0	0	0
0	0	0	0	0	0	0
0	0	0	0	0	0	0
0	0	0	0	0	0	0
0	0	0	0	0	0	0
0	0	0	0	0	0	0
0	0	0	0	0	0	0
0	0	0	0	0	0	0
16	0	0	0	0	0	0
0	0	0	0	0	10	0
0	0	0	0	0	0	0
0	0	0	0	0	0	0
0	0	0	0	0	0	0
0	0	0	0	0	0	0
0	0	0	0	0	13	0
0	0	0	0	0	10	0
0	0	0	0	0	0	0
0	0	0	0	0	0	0
0	0	0	0	0	23	0
0	0	0	0	0	0	0
0	0	0	0	0	0	0
0	0	0	0	0	0	0
0	0	0	0	0	31	0
208	0	0	0	0	0	0
0	0	0	0	0	0	0
0	0	0	0	0	0	0
0	0	0	0	0	0	0
0	0	0	0	0	0	0
0	0	0	0	0	0	0
0	0	0	0	0	0	0
0	0	0	0	0	0	0
0	0	0	0	0	0	0
0	0	0	0	0	0	0
0	0	0	0	0	13	0
15	0	19	0	0	0	0
0	0	0	0	0	0	0
0	0	0	0	0	22	0
28	0	0	0	0	0	0
0	0	0	0	0	0	0
0	0	0	0	0	0	0
0	0	0	0	0	0	0
0	0	0	0	0	0	0
58	0	0	0	0	0	0
0	0	0	0	0	0	0
0	0	0	0	0	0	0

0	0	0	0	0	0	0
0	0	0	0	0	0	0
0	0	0	0	0	0	0
0	0	0	0	0	0	0
0	0	0	0	0	14	0
0	0	0	0	0	0	0
0	0	0	0	0	0	0
26	0	0	0	0	0	0
0	0	0	0	0	0	0
0	0	0	0	0	0	0
0	0	0	0	0	13	0
0	0	0	0	0	0	0
0	0	0	0	0	0	0
11	0	10	0	0	0	0
0	0	16	0	0	0	0
0	0	31	0	0	11	0
56	0	31	0	0	0	0
0	0	33	0	0	0	0
23	0	48	0	0	0	0
0	0	207	0	0	0	0
0	0	25	0	0	0	0
0	0	13	0	0	0	0
0	0	15	0	0	0	0
0	0	45	0	0	0	0
29	0	27	0	0	24	0
0	0	33	0	0	0	0
0	0	52	0	0	0	0
22	0	40	0	0	49	0
0	0	31	0	0	0	0
0	0	25	0	0	0	0
0	0	11	0	0	0	0
0	0	10	0	0	0	0
23	0	11	0	0	0	0
0	0	30	0	0	0	0
0	0	29	0	0	0	0
0	0	33	0	0	0	0
0	0	16	0	0	0	0
26	0	164	0	0	0	0
0	0	10	0	0	0	0
0	0	18	0	0	0	0
47	0	77	0	0	10	0
0	0	39	0	0	0	0
0	0	40	0	0	0	0
0	0	29	0	0	0	0
0	0	63	0	0	0	0
0	0	10	0	0	0	0

15	0	49	0	0	0	0
0	0	48	0	0	0	0
0	0	14	0	0	0	0
0	0	15	0	0	0	0
12	0	11	0	0	0	0
0	0	45	0	0	0	0
0	0	12	0	0	0	0
0	0	17	0	0	0	0
0	0	70	0	0	0	0
0	0	73	0	0	0	0
0	0	11	0	0	0	0
0	0	39	0	0	0	0
0	0	39	0	0	0	0
0	0	30	0	0	0	0
0	0	28	0	0	0	0
0	0	73	0	0	0	0
19	0	14	0	0	0	0
0	0	40	0	0	0	0
35	0	62	0	0	0	0
0	0	301	0	0	0	0
0	0	33	0	0	0	0
0	0	15	0	0	0	0
0	0	49	0	0	0	0
0	0	0	0	0	0	0
0	0	0	0	0	0	0
0	0	0	0	0	0	0
0	0	0	0	0	0	0
0	0	0	0	0	0	0
13	0	0	0	0	0	0
0	0	0	0	0	0	0
0	0	0	0	0	0	0
49	0	0	0	0	0	0
0	0	0	0	0	0	0
0	0	0	0	0	20	0
0	0	0	0	0	0	0
0	0	0	0	0	0	0
0	0	0	0	0	0	0
0	0	0	0	0	0	0
0	0	0	0	0	0	0
0	0	0	0	0	0	0
0	0	0	0	0	0	0
0	0	0	0	0	0	0
0	0	0	0	0	0	0
0	0	0	0	0	0	0
0	0	0	0	0	0	0
0	0	0	0	0	0	0

0	0	0	0	0	0	0
0	0	0	0	0	0	0
0	0	0	0	0	0	0
0	0	0	0	0	0	0
0	0	0	0	0	0	0
35	0	0	0	0	0	0
0	0	0	0	0	0	0
32	0	0	0	0	0	0
0	0	0	0	0	0	0
0	0	0	0	0	0	0
0	0	0	0	0	0	0
0	0	0	0	0	0	0
0	0	0	0	0	0	0
0	0	0	0	0	0	0
0	0	0	0	0	0	0
0	0	0	0	0	0	0
0	0	0	0	0	0	0
0	0	0	0	0	0	0
0	0	0	0	0	0	0
0	0	0	0	0	0	0
0	0	0	0	0	0	0
88	0	0	0	0	0	0
0	0	0	0	0	0	0
0	0	0	0	0	0	0
0	0	0	0	0	0	0
0	0	0	0	0	0	0
0	0	0	0	0	0	0
0	0	0	0	0	0	0
0	0	0	0	0	0	0
0	0	0	0	0	0	0
0	0	0	0	0	0	0
0	0	0	0	0	0	0
0	0	0	12	0	0	0
0	0	0	0	0	0	0
0	0	0	0	0	0	0
20	18	0	0	0	0	0
0	0	0	0	0	0	0
0	0	0	0	0	0	0
0	0	0	0	0	0	0
0	0	0	0	0	0	0
0	0	0	0	0	0	0
0	0	0	0	0	0	0
0	0	0	0	0	0	0
0	0	0	0	0	0	0
0	0	0	0	0	0	0
0	0	0	0	0	0	0

10	0	0	0	0	0	0
0	83	0	0	0	0	0
12	177	0	0	0	0	0
0	0	0	0	0	0	0
0	0	0	0	0	0	0
0	0	0	0	0	0	0
0	0	0	0	0	0	0
0	0	0	0	0	0	0
0	0	0	0	0	0	0
0	0	0	0	0	0	0
0	0	0	0	0	0	0
0	0	0	0	0	0	0
0	0	0	0	0	0	0
0	0	0	0	0	0	0
0	0	0	0	0	0	0
0	0	0	0	0	0	0
0	0	0	0	0	0	0
0	0	0	0	0	0	0
0	0	0	0	0	0	0
0	0	0	0	0	0	0
0	0	0	0	0	0	0
0	0	0	0	0	0	0
0	0	0	0	0	0	0
0	0	0	0	0	0	0
0	0	0	0	0	0	0
0	0	0	0	0	0	0
0	0	0	0	0	0	0
0	0	0	0	0	0	0
0	0	0	0	0	0	0
13	0	0	0	0	0	0
0	0	0	0	0	0	0
0	0	0	0	0	0	0
0	0	0	0	0	0	0
13	0	0	0	0	0	0
70	0	0	0	0	0	0
0	0	0	0	0	0	0
0	0	0	0	0	0	0
0	0	0	0	0	0	0
0	0	0	0	0	0	0
0	0	0	0	0	0	0
0	26	0	0	0	0	0
0	0	0	0	0	0	0
0	0	0	0	0	0	0
0	0	0	0	0	0	0
0	0	0	0	0	0	0
0	0	0	0	0	0	0

0	0	0	0	0	0	0
10	0	0	0	0	0	0
0	0	0	0	0	0	0
0	0	0	0	0	0	0
0	0	0	0	0	0	0
0	0	0	0	0	12	0
0	0	0	0	0	0	0
0	0	0	0	0	0	0
0	0	0	0	0	0	0
0	0	0	0	0	0	0
0	0	0	0	0	0	0
0	0	0	0	0	0	0
0	0	0	0	0	0	0
0	0	0	0	0	0	0
0	0	0	0	0	0	0
0	0	0	0	0	0	0
0	0	0	0	0	0	0
0	10	0	0	0	0	0
0	0	0	0	0	11	0
0	0	0	0	0	0	0
0	0	0	0	0	0	0
0	0	0	0	0	0	0
0	0	0	0	0	0	0
0	0	0	0	0	0	0
0	0	0	0	0	0	0
0	0	0	0	0	0	0
0	0	0	0	0	0	0
0	0	0	0	0	0	0
0	0	0	0	0	0	0
0	0	0	0	0	0	0
0	0	0	0	0	0	0
0	0	0	0	0	0	0
0	0	0	0	0	0	0
10	0	0	0	0	10	0
0	0	0	0	0	0	0
0	0	0	0	0	0	0
10	0	0	0	0	0	0
0	0	0	0	0	0	0
0	0	0	0	0	0	0
0	0	0	0	0	0	0
0	0	0	0	0	0	0
0	0	0	0	0	0	0
0	0	0	0	0	10	0
0	0	0	0	0	0	0
0	0	0	0	0	0	0
0	0	0	0	0	0	0

0	0	0	0	0	0	0
0	0	0	0	0	0	0
0	0	0	0	0	34	0
0	0	0	0	0	0	0
0	0	0	0	0	0	0
0	0	0	0	0	0	0
0	0	0	0	0	0	0
0	0	0	0	0	0	0
0	0	0	0	0	0	0
0	0	0	0	0	0	0
0	0	0	0	0	0	0
0	0	0	0	0	0	0
0	0	0	0	0	0	0
0	0	0	0	0	0	0
0	0	0	0	0	0	0
0	0	0	0	0	0	0
0	0	0	0	0	0	0
0	0	0	0	0	0	0
0	0	0	0	0	0	0
0	0	0	0	0	0	0
0	0	0	0	0	0	0
0	16	0	0	0	58	0
0	0	0	0	0	0	0
0	0	0	0	0	0	0
0	0	0	0	0	0	0
0	0	0	0	0	0	0
0	0	0	0	0	0	0
0	0	0	0	0	0	0
0	0	0	0	0	0	0
0	0	0	0	0	0	0
0	0	0	0	0	0	0
0	0	0	0	0	0	0
0	0	0	0	0	0	0
0	0	0	0	0	0	0
0	0	0	0	0	0	0
0	0	0	0	0	0	0
11	0	0	0	0	0	0
0	0	0	0	0	0	0
0	0	0	0	0	0	0
0	0	0	0	0	0	0
0	0	0	0	0	0	0
0	0	0	0	0	0	0
0	0	0	0	0	0	0
0	0	0	0	0	0	0
0	0	0	0	0	0	0
0	0	0	0	0	0	0

0	0	0	0	0	0	0
0	0	0	0	0	0	0
0	0	0	0	0	0	0
0	0	0	0	0	0	0
0	0	0	0	0	0	0
0	0	0	0	0	59	0
0	0	0	0	0	0	0
0	0	0	0	0	0	0
0	0	0	0	0	0	0
0	0	0	0	0	0	0
0	0	0	0	0	0	0
0	0	0	0	0	0	0
0	0	0	0	0	0	0
0	0	0	0	0	0	0
0	0	0	0	0	0	0
11	0	0	0	0	0	0
0	0	0	0	0	0	0
0	0	0	0	0	0	0
0	0	0	0	0	0	0
0	0	0	0	0	0	0
0	0	0	0	0	0	0
0	0	0	0	0	0	0
0	0	0	0	0	0	0
0	0	0	0	0	0	0
0	0	0	0	0	0	0
0	0	0	0	0	0	0
0	0	0	0	0	0	0
0	0	0	0	0	0	0
0	0	0	0	0	0	0
0	0	0	0	0	0	0
0	0	0	0	0	0	0
0	0	0	0	0	0	0
0	0	0	0	0	0	0
0	0	0	0	0	0	0
0	0	0	0	0	0	0
0	0	0	0	0	0	0
0	0	0	0	0	0	0
0	0	0	0	0	10	0
0	0	0	0	0	0	0
0	0	0	0	0	0	0
0	0	0	0	0	0	0
0	0	0	0	0	14	0
0	0	0	0	0	0	0
0	0	0	0	0	0	0
0	0	0	0	0	51	0
12	0	0	0	0	0	0

0	0	0	0	0	0	0
0	0	0	0	0	10	0
0	0	0	0	0	16	0
0	0	0	0	0	0	0
0	0	0	0	0	0	0
0	0	0	0	0	0	0
0	0	0	0	0	0	0
0	0	0	0	0	33	0
0	0	0	0	0	0	0
0	0	0	0	0	0	0
0	0	0	0	0	0	0
0	0	0	0	0	0	0
0	0	0	0	0	0	0
0	0	0	0	0	0	0
0	0	0	0	0	0	0
0	0	0	0	0	0	0
0	0	0	0	0	0	0
0	0	0	0	0	14	0
0	0	0	0	0	0	0
0	0	0	0	0	0	0
0	0	0	0	0	0	0
0	0	0	0	0	0	0
0	0	0	0	0	0	0
0	0	0	0	0	0	0
0	0	0	0	0	0	0
0	0	0	0	0	0	0
0	0	0	0	0	0	0
0	0	0	0	0	0	0
0	0	0	0	0	0	0
0	0	0	0	0	12	0
0	0	0	0	0	0	0
0	0	0	0	0	0	0
0	0	0	0	0	0	0
0	0	0	0	0	0	0
0	0	0	0	0	0	0
22	0	0	0	0	0	0
0	0	0	0	0	23	0
0	0	0	0	0	0	0
0	0	0	0	0	0	0
0	0	0	0	0	0	0
0	0	0	0	0	13	0
0	0	0	0	0	0	0
0	0	0	0	0	0	0
0	0	0	0	0	0	0
0	0	0	0	0	0	0
0	0	0	0	0	0	0

0	0	0	0	0	12	0
0	0	0	0	0	0	0
0	0	0	0	0	0	0
0	0	0	0	0	0	0
0	0	0	0	0	0	0
10	0	0	0	0	0	0
0	0	0	0	0	0	0
0	0	0	0	0	0	0
0	0	0	0	0	0	0
0	0	0	0	0	0	0
0	0	0	0	0	0	0
0	0	0	0	0	0	0
0	0	0	0	0	0	0
0	0	0	0	0	0	0
0	0	0	0	0	0	0
0	0	0	0	0	0	0
0	0	0	0	0	0	0
0	0	0	0	0	0	0
0	0	0	0	0	0	0
0	0	0	0	0	0	0
0	0	0	0	0	0	0
0	0	0	0	0	0	0
0	0	0	0	0	0	0
0	0	0	0	0	0	0
0	0	0	0	0	0	0
0	0	0	0	0	0	0
0	0	0	0	0	0	0
0	0	0	0	0	0	0
0	0	0	0	0	0	0
0	0	0	0	0	0	0
0	0	0	0	0	0	0
0	0	0	0	0	0	0
0	0	0	0	0	0	0
0	0	0	0	0	0	0
0	0	0	0	0	0	0
0	0	0	0	0	0	0
0	0	0	0	0	0	0
11	0	0	0	0	0	0
0	0	0	0	0	0	0
0	0	0	0	0	0	0
0	0	0	0	0	0	0
0	0	0	0	0	0	0
0	0	0	0	0	0	0
0	0	0	0	0	0	0
0	0	0	0	0	0	0
0	0	0	0	0	0	0

0	0	0	0	0	0	0
0	0	0	0	0	0	0
0	0	0	0	0	0	0
0	0	0	0	0	0	0
0	0	0	0	0	0	0
0	0	0	0	0	0	0
0	0	0	0	0	0	0
0	0	0	0	0	0	0
0	0	0	0	0	0	0
0	0	0	0	0	0	0
0	0	0	0	0	0	0
16	0	0	0	0	0	0
0	0	0	0	0	0	0
0	0	0	0	0	0	0
0	0	0	0	0	0	0
0	0	0	0	0	0	0
0	0	0	0	0	0	0
0	0	0	0	0	0	0
0	0	0	0	0	0	0
0	0	0	0	0	0	0
0	0	0	0	0	0	0
0	0	0	0	0	0	0
0	0	0	0	0	0	0
0	0	0	0	0	0	0
0	0	0	0	0	0	0
0	0	0	0	0	0	0
0	0	0	0	0	0	0
0	0	0	0	0	0	0
0	0	0	0	0	0	0
0	0	0	0	0	0	0
0	0	0	0	0	0	0
0	0	0	0	0	11	0
0	0	0	0	0	0	0
0	0	0	0	0	0	0
0	0	0	0	0	0	0
79	0	0	0	0	0	0
0	0	0	0	0	0	0
0	0	0	0	0	0	0
0	0	0	0	0	0	0
0	0	0	0	0	0	0
0	0	0	0	0	0	0
0	0	0	0	0	0	0
44	0	0	0	0	0	0
0	0	0	0	0	0	0
0	0	0	0	0	0	0
0	0	0	0	0	0	0

0	0	0	0	0	0	0
0	0	0	0	0	0	0
0	0	0	0	0	0	0
11	0	0	0	0	0	0
0	0	0	0	0	0	0
0	0	0	0	0	0	0
0	0	0	0	0	0	0
0	0	0	0	0	0	0
0	0	0	0	0	0	0
0	0	0	0	0	0	0
0	0	0	0	0	0	0
0	0	0	0	0	0	0
0	0	0	0	0	0	0
0	0	0	0	0	0	0
0	0	0	0	0	0	0
0	0	0	0	0	0	0
0	0	0	0	0	0	0
0	0	0	0	0	0	0
0	0	0	0	0	0	0
0	0	0	0	0	0	0
0	0	0	0	0	0	0
0	0	0	0	0	0	0
0	0	0	0	0	0	0
0	0	0	0	0	31	0
0	0	0	0	0	0	0
0	0	0	0	0	0	0
0	0	0	0	0	0	0
0	0	0	0	0	0	0
0	0	0	0	0	0	0
0	0	0	0	0	0	0
0	0	0	0	0	0	0
0	0	0	0	0	0	0
0	0	0	0	0	0	0
0	0	0	0	0	0	0
0	0	0	0	0	0	0
0	0	0	0	0	0	0
0	0	0	0	0	0	0
0	0	0	0	0	11	0
0	0	0	0	0	0	0
0	0	0	0	0	0	0
0	0	0	0	0	0	0
0	0	0	0	0	0	0
0	0	0	0	0	0	0
0	0	0	0	0	0	0
0	0	0	0	0	0	0
26	0	0	0	0	0	0

0	0	0	0	0	0	0
0	0	0	0	0	0	0
0	0	0	0	0	0	0
0	0	0	0	0	0	0
0	0	0	0	0	0	0
0	0	0	0	0	0	0
0	0	0	0	0	0	0
0	0	0	0	0	0	0
0	0	0	0	0	0	0
0	0	0	0	0	0	0
29	0	0	0	0	0	0
0	0	0	0	0	0	0
0	0	0	0	0	0	0
0	0	0	0	0	0	0
0	0	0	0	0	0	0
0	0	0	0	0	0	0
0	0	0	0	0	0	0
0	0	0	0	0	0	0
0	0	0	0	0	0	0
0	0	0	0	0	0	0
0	0	0	0	0	0	0
0	0	0	0	0	0	0
0	0	0	0	0	0	0
0	0	0	0	0	0	0
0	0	0	0	0	0	0
0	0	0	0	0	0	0
0	0	0	0	0	0	0
0	0	0	0	0	0	0
0	0	0	0	0	0	0
0	0	0	0	0	0	0
0	0	0	0	0	0	0
0	0	0	0	0	0	0
338	0	0	0	0	0	0
0	0	0	0	0	0	0
0	0	0	0	0	0	0
0	0	0	0	0	0	0
0	0	0	0	0	0	0
0	0	0	0	0	0	0
0	0	0	0	0	0	0
0	0	0	0	0	0	0
0	0	0	0	0	0	0
21	0	0	0	0	0	0
0	0	0	0	0	0	0
16	0	0	0	0	0	0
0	0	0	0	0	0	0
0	0	0	0	0	0	0

0	0	0	0	0	0	0
184	0	0	0	0	0	0
0	0	0	0	0	0	0
0	0	0	0	0	0	0
0	0	0	0	0	0	0
364	33	0	0	0	0	0
0	0	0	0	0	0	0
0	0	0	0	0	0	0
0	0	0	0	0	0	0
0	0	0	0	0	0	0
0	0	0	0	0	0	0
15	0	0	0	0	0	0
0	0	0	0	0	0	0
0	0	0	0	0	0	0
0	0	0	0	0	0	0
0	0	0	0	0	0	0
0	0	0	0	0	0	0
0	0	0	0	0	0	0
0	0	0	0	0	0	0
0	0	0	0	0	0	0
0	0	0	0	0	0	0
0	0	0	0	0	0	0
0	0	0	0	0	0	0
0	0	0	0	0	0	0
0	0	0	0	0	0	0
0	0	0	0	0	0	0
0	0	0	0	0	0	0
0	0	0	0	0	0	0
0	0	0	0	0	0	0
0	0	0	0	0	0	0
0	0	0	0	0	0	0
0	0	0	0	0	0	0
23	0	0	0	0	0	0
0	0	0	0	0	0	0
0	0	0	0	0	0	0
0	0	0	0	0	0	0
0	0	0	0	0	0	0
0	0	0	0	0	0	0
0	0	0	0	0	0	0
0	0	0	0	0	0	0
0	0	0	0	0	0	0
0	0	0	0	0	0	0
0	0	0	0	0	0	0
0	0	0	0	0	0	0
0	0	0	0	0	0	0
0	0	0	0	0	0	0

0	0	0	0	0	0	0
0	0	0	0	0	0	0
0	0	0	0	0	0	0
0	0	0	0	0	0	0
0	0	0	0	0	0	0
0	0	0	0	0	0	0
0	0	0	0	0	0	0
0	0	0	0	0	0	0
22	0	0	0	0	0	0
0	0	0	0	0	0	0
0	0	0	0	0	0	0
0	0	0	0	0	0	0
0	0	0	0	0	0	0
0	0	0	0	0	0	0
0	0	0	0	0	0	0
0	0	0	0	0	0	0
0	0	0	0	0	0	0
0	0	0	0	0	0	0
0	0	0	0	0	0	0
0	0	0	0	0	0	0
0	0	0	0	0	0	0
10	0	0	0	0	0	0
0	0	0	0	0	0	0
0	0	0	0	0	0	0
0	0	0	0	0	0	0
0	0	0	0	0	0	0
0	0	0	0	0	0	0
0	0	0	0	0	0	0
0	0	0	0	0	0	0
0	0	0	0	0	0	0
0	0	0	0	0	0	0
0	0	0	0	0	0	0
0	0	0	0	0	0	0
0	0	0	0	0	0	0
0	0	0	0	0	0	0
0	0	0	0	0	0	0
0	0	0	0	0	0	0
0	0	0	0	0	0	0
0	0	0	0	0	0	0
0	0	0	0	0	0	0
42	0	0	0	0	0	0
0	0	0	0	0	0	0
0	0	0	0	0	0	0
11	0	0	0	0	13	0
50	0	0	0	0	0	0
60	0	0	0	0	0	0

58	0	0	0	0	0	0
44	0	0	0	0	0	0
0	0	0	0	0	0	0
0	0	0	0	0	0	0
0	0	0	0	0	0	0
0	0	0	0	0	0	0
208	0	0	0	0	0	0
0	0	0	0	0	0	0
0	0	0	0	0	0	0
0	0	0	0	0	0	0
0	0	0	0	0	0	0
55	0	0	0	0	0	0
0	0	0	0	0	0	0
33	0	0	0	0	0	0
0	0	0	0	0	0	0
0	0	0	0	0	0	0
0	0	0	0	0	0	0
0	0	0	0	0	0	0
0	0	0	0	0	0	0
0	0	0	0	0	0	0
0	0	0	0	0	0	0
0	0	0	0	0	0	0
0	0	0	0	0	0	0
0	0	0	0	0	0	0
0	0	0	0	0	0	0
0	0	0	0	0	0	0
0	0	0	0	0	0	0
0	0	0	0	0	0	0
0	0	0	0	0	0	0
0	0	0	0	0	0	0
0	0	0	0	0	0	0
0	0	0	0	0	0	0
0	0	0	0	0	0	0
0	0	0	0	0	0	0
0	0	0	0	0	0	0
0	0	0	0	0	0	0
0	0	0	0	0	0	0
0	0	0	0	0	0	0
0	0	0	0	0	0	0
0	0	0	0	0	0	0
0	0	0	0	0	0	0
0	0	0	0	0	0	0
0	0	0	0	0	0	0
0	0	0	0	0	0	0
0	0	0	0	0	0	0
0	0	0	0	0	0	0

0	0	0	0	0	0	0
0	0	0	0	0	10	0
0	0	0	0	0	0	0
0	0	0	0	0	0	0
0	0	0	0	0	0	0
0	0	0	0	0	0	0
0	0	0	0	0	19	0
0	0	0	0	0	0	0
0	0	0	0	0	0	0
0	0	0	0	0	0	0
0	0	0	0	0	0	0
0	0	0	0	0	0	0
0	0	0	0	0	0	0
0	0	0	0	0	0	0
0	0	0	0	0	0	0
0	0	0	0	0	0	0
0	0	0	0	0	0	0
0	0	0	0	0	0	0
0	0	0	0	0	0	0
0	0	0	0	0	0	0
15	0	0	0	0	0	0
0	0	0	0	0	0	0
0	0	0	0	0	0	0
0	0	0	0	0	0	0
0	0	0	0	0	0	0
0	0	0	0	0	0	0
0	0	0	0	0	0	0
0	0	0	0	0	0	0
0	0	0	0	0	0	0
0	0	0	0	0	0	0
0	0	0	0	0	13	0
0	0	0	0	0	0	0
0	0	0	0	0	0	0
0	0	0	0	0	0	0
0	0	0	0	0	0	0
0	0	0	0	0	0	0
0	0	0	0	0	0	0
0	0	0	0	0	0	0
0	0	0	0	0	0	0
0	0	0	0	0	0	0
0	0	0	0	0	0	0
0	0	0	0	0	0	0
0	0	0	0	0	0	0
0	0	0	0	0	0	0
0	0	0	0	0	0	0
0	0	0	0	0	0	0

0	0	0	0	0	0	0
0	0	0	0	0	0	0
0	0	0	0	0	0	0
0	0	0	0	0	0	0
15	0	0	0	0	18	0
0	0	0	0	0	13	0
0	0	0	0	0	11	0
0	0	0	0	0	13	0
0	0	0	0	0	11	0
0	0	0	0	0	10	0
0	0	0	0	0	35	0
0	0	0	0	0	11	0
0	0	0	0	0	0	0
0	0	0	0	0	0	0
0	0	0	0	0	0	0
0	0	0	0	0	0	0
0	0	0	0	0	0	0
0	0	0	0	0	0	0
0	0	0	0	0	0	0
0	0	0	0	0	0	0
0	19	0	0	0	0	0
0	12	0	0	0	0	0
0	0	0	0	0	0	0
0	0	0	0	0	0	0
11	0	0	0	0	0	0
0	0	0	0	0	0	0
0	0	0	0	0	0	0
0	0	0	0	0	0	0
17	0	0	0	0	0	0
0	0	0	0	0	0	0
0	0	0	0	0	0	0
0	0	0	0	0	0	0
0	0	0	0	0	0	0
0	0	0	0	0	0	0
0	0	0	0	0	0	0
0	0	0	0	0	0	0
0	0	0	0	0	0	0
0	0	0	0	0	0	0
0	0	0	0	0	0	0
0	0	0	0	0	0	0
0	0	0	0	0	0	0
0	0	0	0	0	0	0
0	0	0	0	0	0	0
0	0	0	0	0	0	0
0	0	0	0	0	0	0
0	0	0	0	0	0	0

0	0	0	0	0	0	0
0	0	0	0	0	0	0
17	0	0	0	0	0	0
0	0	0	0	0	0	0
0	0	0	0	0	0	0
0	0	0	0	0	0	0
0	0	0	0	0	0	0
0	0	0	0	0	0	0
0	0	0	0	0	0	0
0	0	0	0	0	0	0
0	0	0	0	0	0	0
0	0	0	0	0	0	0
0	0	0	0	0	0	0
0	0	0	0	0	0	0
0	0	0	0	0	0	0
0	0	0	0	0	0	0
0	0	0	0	0	0	0
0	0	0	0	0	0	0
0	0	0	0	0	0	0
0	0	0	0	0	0	0
0	0	0	0	0	0	0
0	0	0	0	0	0	0
0	0	0	0	0	0	0
0	0	0	0	0	0	0
0	0	0	0	0	0	0
0	0	0	0	0	0	0
0	0	0	0	0	0	0
0	0	0	0	0	0	0
0	0	0	0	0	0	0
0	0	0	0	0	0	0
0	0	0	0	0	0	0
0	0	0	0	0	0	0
0	0	0	0	0	0	0
0	0	0	0	0	0	0
13	0	0	0	0	0	0
52	0	0	0	0	0	0
11	0	0	0	0	0	0
36	0	0	0	0	0	0
11	0	0	0	0	0	0
10	0	0	0	0	0	0
11	0	0	0	0	0	0
10	0	0	0	0	0	0
11	0	0	0	0	0	0
10	0	0	0	0	0	0
10	0	0	0	0	0	0
13	0	0	0	0	0	0
11	0	0	0	0	0	0

11	0	0	0	0	0	0
11	0	0	0	0	0	0
22	0	0	0	0	0	0
38	0	0	0	0	0	0
12	0	0	0	0	0	0
11	0	0	0	0	0	0
24	0	0	0	0	0	0
60	0	0	0	0	0	0
0	0	0	0	0	0	0
0	0	0	0	0	0	0
0	0	0	0	0	0	0
0	0	0	0	0	0	0
0	0	0	0	0	0	0
0	0	0	0	0	0	0
0	0	0	0	0	0	0
0	0	0	0	0	0	0
0	0	0	0	0	0	0
0	0	0	0	0	0	0
0	0	0	0	0	0	0
0	0	0	0	0	0	0
0	0	0	0	0	0	0
0	0	0	0	0	0	0
0	0	0	0	0	0	0

PH_348	UC_242	UC_249	UC_261	UC_273	UC_280	UC_282
0	90	0	0	0	0	0
0	0	28	13	0	0	0
0	168	0	0	0	14	10
81	111	0	0	0	32	0
88	80	0	0	0	0	0
0	379	0	0	0	0	0
11	162	0	0	0	0	0
0	12	0	0	0	0	0
0	27	13	0	14	309	0
275	205	34	53	47	301	0
0	10	0	0	25	0	0
0	0	0	0	0	0	0
0	43	0	0	0	0	0
0	0	0	0	0	0	0
0	12	0	0	0	0	0
29	0	11	0	0	35	17
552	858	205	704	15	853	43
124	81	0	0	0	0	0
0	11	29	22	0	70	0
102	24	0	0	0	71	0
0	0	0	0	0	12	0
0	0	0	0	0	0	33
91	12	0	0	0	11	0
0	0	0	0	0	0	0
4899	882	754	1304	190	195	335
162	514	43	381	160	121	152
211	170	28	439	0	14	11
90	68	23	168	21	19	33
559	146	53	491	54	65	60
0	36	11	0	0	41	65
0	13	19	16	0	0	0
0	22	0	11	0	0	0
0	1345	1041	1666	167	1239	295
0	12	0	0	0	0	0
248	0	0	0	0	0	0
10	0	26	0	0	0	0
10	0	0	0	0	33	0
122	507	56	110	0	161	0
35	40	22	56	0	24	31
437	1699	550	1689	326	869	178
0	55	97	132	0	168	12

0	43	68	157	0	109	0
95	29	26	58	0	39	11
33	62	0	42	0	20	11
1937	2282	60	921	141	40	38
387	205	0	251	0	0	0
0	12	0	0	14	0	0
0	153	0	21	0	0	0
0	15	0	27	51	0	0
0	36	0	0	0	0	0
0	0	0	22	55	0	0
0	0	0	0	0	0	0
0	10	0	0	0	0	0
0	126	0	148	13	10	0
0	0	0	0	0	0	0
0	0	0	0	0	0	0
0	0	17	23	52	0	0
0	41	0	0	0	0	0
412	496	268	264	191	582	182
190	149	0	94	0	0	0
0	0	0	0	47	44	11
0	0	0	0	0	0	0
0	0	0	0	0	0	0
0	0	0	0	0	0	0
0	70	0	0	0	0	0
0	0	0	0	0	0	0
0	81	0	0	0	0	0
0	13	0	0	0	0	0
13	33	0	0	0	0	0
0	0	0	0	0	0	0
0	43	0	10	0	0	0
0	250	22	67	173	0	0
179	3196	121	536	323	192	60
0	98	0	0	0	118	0
0	35	0	16	0	0	0
0	0	0	0	0	0	0
0	28	0	0	0	0	0
0	35	0	10	0	0	0
0	120	0	0	0	0	0
0	50	0	0	0	0	0
0	0	0	0	0	0	0
0	69	49	11	66	0	0
0	132	0	0	0	0	0
0	0	0	0	0	0	0
0	17	0	0	0	0	0
0	128	26	81	133	0	11
88	1394	160	317	397	153	73

0	0	0	0	0	0	0
230	478	11	16	0	11	0
0	59	0	27	52	0	0
0	0	0	0	0	0	0
52	1499	74	476	302	29	45
0	103	38	0	94	241	0
0	0	0	0	0	0	0
0	0	0	0	0	0	0
0	0	0	0	0	0	0
0	41	0	0	0	26	0
36	234	0	15	0	48	0
0	59	0	0	0	12	0
2854	4197	505	1066	1547	12196	108
0	0	0	0	0	88	0
64	555	87	151	427	1073	0
0	18	0	32	0	0	0
104	0	0	0	0	0	0
0	64	0	10	10	0	0
0	41	0	10	0	222	0
0	24	0	14	0	0	0
0	0	0	0	0	0	0
0	10	0	0	0	26	0
39	53	0	40	0	0	0
0	93	74	12	67	479	0
0	94	59	0	235	8585	14
10	211	35	44	165	945	0
0	104	0	13	41	356	0
0	0	0	0	0	0	0
11	14	0	19	37	138	0
191	359	347	191	476	5888	28
125	168	0	0	0	32	0
0	0	0	0	0	190	0
75	14	0	0	0	34	0
297	31	0	39	0	153	0
0	0	0	0	0	0	0
0	0	0	0	11	139	0
0	11	24	0	71	190	0
0	55	42	17	189	547	0
0	0	0	0	15	174	0
25	380	106	132	256	1564	20
0	29	10	10	28	148	0
0	42	0	18	12	68	0
0	63	0	38	35	0	0
35016	2832	215	1152	496	818	134
292	25	0	21	0	0	0
1364	238	78	163	294	115	110

85	0	0	0	0	0	11
54	254	0	0	0	12	0
175	377	105	39	0	152	25
79	1081	102	597	349	103	92
0	34	0	28	0	0	0
0	254	0	109	25	17	0
0	0	0	0	0	0	0
0	31	0	12	11	0	0
0	0	0	0	39	0	0
0	0	0	0	0	0	0
27	183	13	58	42	0	0
223	34	32	0	0	54	27
0	0	0	0	0	0	0
0	49	15	0	0	44	0
0	167	25	25	0	97	13
0	31	0	0	0	0	0
0	134	0	36	0	0	0
0	0	0	0	0	0	0
0	0	0	0	0	0	0
0	0	0	0	66	46	23
0	30	41	0	151	755	0
0	0	0	0	0	40	0
47	228	174	97	94	854	0
0	10	0	0	0	0	0
0	30	76	0	29	456	0
54	233	12	21	33	85	45
57	371	11	10	11	91	0
472	227	10	113	15	816	13
0	49	0	0	0	142	0
29	32	0	0	0	116	0
0	108	0	0	0	0	0
267	54	0	0	0	42	0
24	314	0	0	11	152	0
11	38	0	0	0	295	0
63	114	15	0	0	44	0
0	134	12	133	40	0	10
0	41	20	33	26	0	10
0	0	0	0	0	0	0
0	0	0	0	0	0	0
0	0	0	0	0	0	0
10	265	40	0	3520	107	0
0	0	0	0	0	0	0
0	0	18	10	10	0	0
0	0	0	0	0	0	0
86	413	399	0	191	208	293
274	442	444	45	226	1008	479

30	205	1250	0	533	748	1399
47	1884	433	0	2237	113	78
3475	9018	4521	465	2706	1790	2978
845	12189	302	0	707	9987	50
0	81	0	38	0	0	0
296	201	51	0	58	564	29
126	399	78	60	330	900	0
0	35	0	0	10	161	0
0	0	0	0	0	0	11
0	0	0	0	0	0	0
0	13	0	0	0	0	0
0	0	0	0	0	0	0
42	0	0	0	0	11	0
920	1033	16	95	26	649	0
0	0	0	0	0	0	0
0	224	49	173	134	0	23
0	47	0	0	0	0	0
19	102	0	0	0	0	0
0	0	0	0	0	0	0
0	0	0	0	0	0	0
0	0	0	0	0	0	0
57	7106	943	1512	258	183	903
0	0	0	18	0	0	0
0	0	0	0	0	0	0
0	0	0	0	0	0	0
74	0	0	0	0	10	0
0	0	49	0	0	79	11
0	0	0	0	0	0	0
0	28	0	0	0	0	0
0	0	0	0	0	0	0
0	0	0	0	0	0	0
0	0	0	0	0	0	0
0	0	0	0	0	0	0
0	0	0	0	0	0	0
0	0	0	0	0	0	11
17	0	0	0	0	11	0
873	2193	1129	871	1854	1883	569
296	830	984	321	3835	6285	128
0	10	0	0	166	152	27
0	0	0	0	0	0	0
30	0	0	0	0	0	0
0	480	90	233	123	1006	57
0	29	53	0	116	2163	0
0	0	10	0	58	981	0
0	0	0	49	0	0	0
0	282	38	658	28	0	41

0	142	12	252	41	0	0
0	246	55	250	160	23	63
201	0	25	16	0	137	23
0	10	12	0	0	32	26
0	12	39	0	39	2057	0
137	485	464	112	1129	3010	138
13	234	29	37	151	1286	0
0	34	0	0	423	0	0
0	33	0	0	0	0	0
0	0	0	0	0	0	0
0	10	0	0	77	0	0
0	0	0	0	0	0	0
0	16	0	0	0	0	0
45	103	24	0	519	2702	0
49	75	0	50	0	0	0
0	0	0	0	0	0	0
0	0	0	0	13	0	0
0	26	0	0	0	0	0
238	107	32	88	135	44	76
108	0	0	0	0	0	0
0	0	25	0	0	0	0
0	0	28	0	0	30	0
0	108	182	22	266	110	0
0	0	39	0	0	0	0
694	492	843	469	475	1108	128
0	0	0	22	55	1054	0
0	0	0	0	0	144	0
29	275	56	342	211	2338	40
0	14	101	0	116	3171	10
0	0	54	0	12	890	0
82	0	0	50	0	0	0
0	41	16	0	57	554	0
0	0	0	0	0	0	0
0	14	13	0	66	403	0
621	65	24	177	238	211	13
0	0	0	12	76	61	0
0	0	0	68	85	22	0
0	0	0	0	0	0	29
0	0	0	0	0	0	0
0	114	0	117	40	854	0
10	10	0	70	33	122	16
36	64	24	26	49	0	11
0	13	0	10	17	0	0
67	584	170	786	383	139	77
0	0	0	0	0	0	0
77	1509	239	70	103	470	320

102	4608	1723	187	4245	1563	231
0	24	0	0	0	15	0
0	83	34	0	0	54	11
505	728	110	112	55	210	23
0	6810	97	0	67	253	0
0	134	314	0	242	99	0
0	35	0	21	190	29	0
117	419	38	0	118	90	68
0	62	0	0	0	0	0
0	0	0	0	0	0	0
0	0	10	0	0	0	0
0	663	116	11	17	268	39
232	2944	2235	757	3926	2116	112
0	246	50	40	184	26	0
0	465	63	51	46	680	0
78	562	94	184	59	545	0
86	2381	352	570	227	2281	28
0	144	14	29	10	11	0
20	87	0	33	0	0	0
10	276	11	34	21	25	0
0	113	0	23	0	0	0
0	49	0	37	48	0	0
129	209	14	427	219	0	20
789	81	0	68	11	0	10
207	27	0	20	0	0	0
0	148	54	0	12	11	0
2996	6856	1534	4979	1873	1352	303
217	417	132	1114	0	107	0
72	139	0	44	0	17	0
0	1081	80	649	0	10	0
69	2639	697	227	520	671	255
55	1550	161	3176	0	40	34
65	134	0	560	20	0	0
23	40	0	0	244	0	0
0	482	220	1266	0	15	24
22	1618	0	1130	83	45	31
0	135	0	44	16	0	0
338	906	39	313	255	268	0
579	517	0	451	169	54	34
93	357	60	336	220	95	32
17	849	25	53	1092	45	0
0	1779	26	29	1731	128	0
0	13	0	26	12	0	0
0	363	255	127	330	134	0
71	279	81	128	617	99	149
0	40	13	0	0	16	15

28	1141	415	325	98	2051	696
49	1022	516	254	11	55	245
0	164	118	11	22	50	11
82	994	88	105	66	10	274
0	67	0	51	0	12	0
0	10	0	10	0	0	22
24	323	152	214	10	206	173
12	183	11	40	0	0	35
0	41	47	13	0	0	177
0	328	0	286	0	0	37
0	0	0	0	0	0	0
34	1113	93	189	83	38	798
445	0	0	0	0	0	0
40	84	0	0	0	0	0
0	25	0	0	0	0	0
0	0	0	0	0	0	0
560	0	0	0	0	0	0
0	0	0	0	0	56	0
202	364	107	296	423	1503	21
0	0	0	0	0	0	0
0	0	0	0	0	0	0
0	0	0	0	0	0	0
0	0	0	0	0	0	0
0	141	76	118	169	987	31
0	0	0	0	0	0	0
0	12	0	59	0	182	0
0	24	0	0	0	0	0
0	27	0	0	40	0	0
0	0	0	0	0	78	0
0	0	0	12	0	0	0
0	0	0	0	0	0	0
0	0	0	0	0	0	0
0	0	0	0	0	0	0
0	0	0	0	0	58	0
42	29	0	67	0	0	0
0	0	0	0	0	0	0
0	0	0	0	0	0	0
0	0	0	0	0	0	0
0	12	0	20	0	0	0
0	25	0	45	0	374	0
0	10	0	0	0	0	0
0	70	22	0	0	364	0
0	67	0	0	13	133	0
27	90	0	16	0	36	0
0	0	0	0	0	0	0
0	0	0	0	0	0	0

42	11	10	13	11	45	12
0	0	0	0	0	0	0
0	17	0	0	0	21	0
380	440	247	84	1026	3425	92
0	0	0	0	0	0	0
0	11	0	30	0	0	0
0	0	22	0	0	0	0
21	0	28	0	0	30	11
33	0	0	10	0	13	0
0	11	25	0	0	58	23
52	0	29	10	52	1662	11
888	126	71	178	102	1027	27
142	55	36	60	53	222	0
238	295	49	199	228	1138	44
23	0	0	0	26	13	0
0	0	0	0	0	0	0
0	0	0	0	0	0	0
0	0	0	0	37	213	0
0	0	0	0	0	0	0
0	0	0	0	0	11	0
0	52	12	12	74	348	0
0	0	0	0	0	0	0
10	0	12	14	0	87	24
62	69	50	39	389	2886	22
0	0	0	0	0	31	0
0	0	0	0	0	162	0
0	10	47	0	0	52	26
0	0	0	0	0	0	0
160	409	359	192	179	1146	58
0	0	32	0	0	40	0
0	0	21	0	0	105	0
0	0	76	21	0	199	27
0	12	23	14	32	418	0
0	29	0	0	0	42	0
0	0	0	0	0	0	0
0	0	0	0	0	0	0
0	0	0	0	13	513	0
1256	150	72	186	10	523	47
0	0	0	0	0	0	0
0	0	0	0	0	108	0
139	12	0	0	0	39	0
10	0	0	0	0	0	0
0	0	0	0	0	64	0
0	0	0	0	0	0	0
0	0	0	0	0	0	0
0	0	0	0	0	0	0

0	21	31	0	11	174	38
0	0	0	0	0	49	0
0	0	0	0	0	0	0
0	0	0	0	0	0	0
0	0	0	0	0	0	0
0	0	0	0	0	392	0
0	0	0	0	0	0	0
0	0	0	0	0	93	0
1117	971	278	646	1085	6768	194
137	44	0	33	0	635	0
0	0	0	0	25	330	0
245	371	346	152	431	2817	45
0	0	0	0	0	0	0
0	0	0	0	0	37	0
0	35	11	0	23	260	0
11	125	513	12	1476	6706	94
0	10	0	0	26	1417	0
0	0	0	39	0	0	0
0	0	0	0	0	0	0
0	12	0	0	0	0	0
0	0	0	0	0	0	0
0	0	0	0	0	0	0
0	22	0	11	0	0	0
70	88	0	0	147	734	0
78	392	299	991	270	666	156
399	346	170	308	884	4984	35
235	47	0	47	44	31	0
0	0	0	0	79	0	0
46	49	48	58	143	62	52
0	0	0	0	0	0	0
0	0	0	0	0	0	0
0	0	0	0	0	0	0
0	0	10	0	72	0	0
27	33	0	0	68	0	0
57	69	0	35	99	21	0
0	0	0	0	0	33	0
114	41	0	12	0	237	58
907	2854	853	2316	674	1150	1299
96	43	25	87	12	35	69
0	0	0	0	0	0	13
16	357	365	473	132	0	142
0	0	0	0	0	0	0
0	39	0	13	0	0	0
0	0	0	12	11	0	26
0	0	0	0	0	0	0
0	78	35	0	12	52	10

0	0	16	39	14	0	0
0	0	0	0	0	0	0
0	0	0	0	0	0	0
0	122	205	162	121	29	213
0	10	0	0	18	29	0
0	10	0	0	140	33	11
0	45	14	36	98	1464	0
30	21	0	22	122	0	0
0	0	0	0	33	449	13
0	0	0	0	0	0	0
377	199	0	136	840	530	0
0	12	0	15	18	0	14
0	0	27	0	0	0	0
0	128	0	0	0	0	0
0	68	0	0	11	27	0
0	24	0	0	0	0	0
0	0	0	0	0	246	0
0	0	0	0	15	186	0
38	31	26	13	69	975	0
0	12	0	0	0	0	0
0	45	314	46	10	524	316
0	0	0	0	0	0	0
0	38	0	58	0	304	0
0	0	0	0	0	0	0
0	152	12	65	91	11	21
0	0	0	0	0	0	0
0	0	0	0	0	0	0
0	83	0	0	0	10	0
0	0	0	0	13	0	0
0	0	0	0	0	0	0
0	0	0	0	40	0	0
0	0	0	0	0	0	0
0	0	0	0	0	0	0
0	0	35	0	0	262	0
0	0	0	0	0	0	0
0	0	0	0	0	0	0
0	112	0	29	0	0	0
31	10	0	20	26	495	10
1705	6476	1006	1381	2324	9405	485
0	0	0	0	0	0	0
0	0	0	0	0	0	0
0	0	0	0	0	0	0
75	40	0	11	169	37	38
135	33	0	0	0	36	0
0	0	0	0	0	0	0
0	0	0	0	0	0	0

0	0	0	0	0	0	0
0	58	27	29	39	154	0
0	0	0	0	0	0	0
0	0	0	0	0	0	0
0	0	0	0	0	0	0
0	128	0	0	0	469	0
0	0	0	0	0	0	0
10	0	0	0	0	41	0
0	0	0	0	0	0	0
48	0	0	0	0	0	0
0	0	0	0	0	108	0
0	116	24	0	0	57	0
68	1687	2062	488	2587	15149	423
34	320	153	34	423	1575	55
0	26	34	0	0	0	0
0	0	0	0	0	0	0
0	0	0	0	0	52	0
0	36	0	0	0	0	0
0	0	75	24	53	103	0
34	762	295	124	748	2766	47
32	140	138	25	218	2416	76
40	70	53	14	41	242	10
259	84	0	76	27	0	0
0	0	0	0	0	0	0
0	0	0	0	0	122	0
0	0	0	0	0	0	0
0	0	0	0	10	0	0
0	311	131	12	185	2460	16
0	0	0	0	0	0	0
0	0	0	0	0	12	0
0	0	0	0	0	0	0
0	0	0	0	0	0	0
113	425	195	122	119	299	11
0	0	0	53	0	341	0
0	14	0	0	0	760	0
0	0	0	0	0	0	0
40	142	105	35	30	98	14
0	12	148	17	12	28	10
147	109	218	0	0	140	104
0	184	369	112	120	189	62
0	10	0	12	0	0	0
25	59	0	0	0	0	0
0	0	0	0	0	0	0
104	256	490	884	856	2077	68
0	0	0	0	0	225	0
0	0	0	0	0	83	0

0	0	0	0	0	0	0
0	0	0	0	0	42	0
0	0	0	0	0	0	0
0	0	12	0	21	1422	0
110	279	189	169	375	238	12
60	44	221	181	590	395	11
0	78	132	28	370	191	19
0	0	0	135	47	16	0
386	127	26	36	99	47	0
0	11	0	40	0	0	0
0	0	0	10	0	243	0
0	10	0	0	0	47	0
0	0	0	0	0	0	0
0	0	0	0	0	49	0
0	0	0	0	0	0	0
0	0	0	0	0	0	0
0	0	0	0	0	0	0
0	68	0	72	0	555	0
0	0	0	0	0	219	0
0	78	30	0	0	0	263
0	10	31	0	250	1545	0
0	0	0	0	0	0	0
0	247	512	619	2500	6816	131
0	0	0	0	12	72	0
87	402	170	279	966	4137	27
0	0	0	0	0	0	0
0	18	35	0	781	708	10
10	140	27	34	252	2540	0
0	12	0	0	0	0	0
0	0	0	0	0	0	0
0	0	0	0	0	0	0
30	3336	94	1903	10	1030	0
0	927	88	811	15	537	10
0	0	0	0	0	0	0
114	14	0	0	10	156	0
0	0	0	0	0	49	0
0	0	0	0	0	87	0
0	0	0	0	0	0	0
0	10	0	0	0	0	0
0	0	0	0	0	0	0
0	144	0	0	13	24	81
15	1291	13	437	102	0	32
696	0	88	0	1196	724	462
0	61	236	0	0	69	10
95	0	249	0	0	182	0
0	0	0	0	0	0	0

0	0	17	0	28	0	16
22	222	454	68	12	266	569
0	0	77	10	0	10	0
37	11	0	26	33	39	44
0	35	280	10	0	47	268
0	0	0	0	122	38	98
59	0	0	175	355	12	0
53	0	0	0	0	1287	0
883	2963	3154	456	3687	5240	349
27	3624	843	627	336	4788	78
10	3393	85	159	149	352	12
38	16	0	10	0	184	0
0	0	0	0	0	0	0
28	26	0	0	0	90	16
0	0	0	0	0	0	0
33	0	0	0	0	295	112
0	0	11	0	0	41	0
0	0	0	0	0	0	0
0	0	0	0	0	22	10
0	0	35	0	0	0	0
100	0	0	25	548	60	134
53	38	102	60	0	126	43
0	39	0	0	0	0	77
0	0	0	0	0	0	0
0	0	0	0	0	0	0
10	218	401	59	37	74	42
175	3043	2768	1035	2448	1845	1693
391	1642	638	428	2153	533	335
7104	13183	10130	6014	12557	6964	2290
0	115	16	124	295	36	33
10	51	46	106	720	11	23
0	100	0	45	210	14	503
322	1508	3442	1455	1823	365	150
138	1244	840	600	1157	317	174
0	34	51	20	246	32	0
0	58	0	110	0	0	0
3081	216	185	90	740	750	1075
1326	351	184	57	104	847	1393
527	91	70	11	74	927	1374
255	327	673	236	518	162	633
0	28	0	26	24	0	0
0	0	16	0	15	0	0
2764	261	227	254	225	1051	3061
64	0	22	0	0	55	41
0	21	70	0	0	0	0
974	168	316	81	29	1373	1506

817	160	303	14	231	321	192
1712	3140	1814	2072	1753	7937	1221
0	18	0	0	68	410	0
0	328	230	95	137	263	325
445	57	67	10	279	55	18
0	0	0	15	11	0	0
992	172	819	102	546	406	500
0	52	37	81	11	331	0
0	0	0	0	0	0	0
86	28	11	0	0	34	0
301	107	32	32	0	98	15
54	38	64	0	15	139	54
0	0	0	0	0	0	0
0	0	0	0	0	0	0
108	194	54	0	12	224	54
96	229	348	502	297	6572	32
1460	76	51	23	12	66	11
0	0	111	176	75	808	59
0	0	39	0	0	50	72
60	0	0	0	0	0	0
126	0	58	0	0	233	98
0	0	0	0	0	0	0
0	0	0	0	0	0	0
0	57	15	11	0	0	0
0	78	167	0	86	253	62
0	0	0	0	0	0	0
0	0	0	0	0	0	0
0	0	0	0	0	0	0
0	0	0	0	0	0	0
112	0	0	0	0	10	10
0	0	0	0	0	20	22
0	0	0	0	0	167	0
0	44	34	10	11	253	53
0	157	244	56	210	621	82
60	0	26	66	17	2374	0
0	12	19	14	26	902	0
0	44	0	52	72	0	0
127	0	0	0	0	0	0
168	241	146	88	135	527	31
0	10	12	10	15	1688	0
30	12	0	20	60	1169	0
0	64	192	117	74	0	131
0	0	0	0	0	0	0
0	39	236	72	65	0	182
0	0	0	0	0	18	0
0	0	307	18	59	0	206

0	0	0	0	0	0	0
17	0	0	10	384	0	0
33	60	10	80	30	12	10
0	0	0	0	0	0	12
0	0	0	14	0	55	0
0	0	0	0	0	0	0
0	0	0	0	0	0	0
0	0	0	0	0	0	0
0	0	15	0	0	28	0
0	54	56	23	10	190	25
0	177	0	0	0	0	0
0	41	30	0	0	98	27
11	35	0	34	0	52	0
44	44	0	12	0	0	0
0	76	0	124	0	86	0
12	41	0	27	0	33	0
38	14	0	34	0	0	0
0	10	0	12	0	51	0
0	0	34	0	0	66	13
0	0	0	0	0	50	0
156	448	146	1106	0	0	11
12	0	0	80	0	0	0
0	12	14	0	0	0	0
0	45	0	0	201	11	0
0	0	64	22	0	27	45
0	0	14	0	28	0	0
0	0	0	0	0	0	0
0	32	13	0	12	16	0
0	12	0	25	0	0	0
0	0	0	0	0	0	0
0	0	0	0	0	0	0
0	0	0	0	0	0	0
0	0	0	0	0	0	0
0	0	0	0	0	0	0
36	70	0	0	0	0	0
0	0	0	0	0	0	0
0	0	0	0	0	0	0
0	0	0	0	0	0	0
0	0	0	0	0	0	0
0	0	0	0	26	0	0
0	0	0	0	0	0	0
0	0	0	0	0	0	0
0	0	0	0	0	0	0
0	0	0	0	0	15	0
0	109	0	0	0	0	0
0	32	0	0	0	56	0

0	0	0	0	0	65	0
0	33	37	13	0	0	0
0	12	0	0	0	0	0
13	0	0	0	0	76	0
0	0	0	0	0	48	0
0	0	0	0	0	0	0
64	34	0	0	0	103	0
0	15	0	0	0	58	0
0	0	0	0	0	0	0
0	0	0	0	0	0	0
0	0	0	0	0	0	0
0	0	14	0	14	442	0
94	0	0	43	0	0	0
0	21	0	0	0	19	0
0	0	0	0	0	0	0
0	0	0	0	0	140	0
0	31	0	0	0	36	0
0	11	0	0	10	143	0
498	51	0	34	32	0	0
0	0	0	0	28	13	0
0	25	0	0	0	0	0
0	0	0	0	0	0	0
0	0	71	0	0	31	40
0	140	0	0	0	0	0
0	0	0	0	0	0	0
16	0	0	0	0	0	0
0	0	0	0	0	101	0
0	0	0	0	0	65	0
0	0	0	0	0	0	0
0	0	0	0	0	0	0
0	0	0	0	0	0	0
10	0	0	0	0	11	0
0	0	0	0	0	35	0
95	156	0	0	0	0	0
0	0	68	0	27	0	16
0	0	0	0	14	498	0
0	0	0	0	0	0	0
0	735	106	57	0	92	0
0	0	0	0	0	0	0
0	0	0	0	0	0	0
22	31	0	125	0	717	0
0	15	0	46	0	249	0
0	0	0	37	10	446	0
0	0	0	0	0	0	0
23	364	38	0	55	33	40
0	0	0	0	0	0	0

0	34	0	0	0	418	10
0	21	0	0	0	38	0
0	0	0	0	0	0	0
0	0	0	0	0	149	0
694	0	273	0	374	93	170
632	412	214	151	829	1835	34
0	0	0	0	0	0	0
0	126	0	10	21	337	0
0	0	0	0	0	318	0
0	104	0	11	0	340	0
0	11	0	0	0	0	0
0	0	0	0	0	32	0
0	370	905	107	134	37	245
0	0	0	0	0	0	0
0	0	0	0	0	0	0
0	0	36	0	100	10	0
0	0	0	0	125	0	0
0	0	0	0	0	0	47
22	824	1441	81	14	12	775
0	0	0	0	0	0	0
0	0	0	0	0	0	0
0	11	0	0	19	0	0
0	39	0	0	124	0	0
0	0	0	0	0	400	0
0	0	0	0	40	29	0
10	0	41	0	0	67	0
0	0	0	0	0	0	0
0	0	11	0	0	1168	0
0	49	79	17	165	26	0
0	0	0	0	0	0	0
0	0	0	0	0	367	0
0	21	10	0	76	64	0
0	0	0	0	0	0	0
62	11	0	31	58	0	17
0	0	0	0	0	107	0
0	0	0	0	0	0	0
0	0	0	0	0	0	0
0	0	0	0	0	332	0
0	0	0	0	23	339	0
0	47	13	20	0	69	0
0	0	0	0	12	0	0
0	0	23	0	0	0	0
0	0	36	21	0	78	10
0	0	0	0	18	178	0
0	0	0	0	0	0	0
12	83	104	87	128	12	22

0	0	43	0	0	79	31
0	17	0	0	0	0	0
0	0	0	0	0	0	0
0	44	0	0	0	0	0
0	225	11	46	35	259	0
0	0	47	0	0	28	0
0	0	0	0	0	0	29
0	24	126	0	18	59	0
0	0	0	0	0	21	0
0	0	0	0	0	0	0
0	374	203	10	11	248	131
0	68	115	0	19	105	0
0	169	1097	0	275	221	0
0	14	0	0	33	0	0
0	153	366	10	65	101	0
0	45	0	0	16	0	0
0	38	35	0	0	77	25
0	25	0	0	0	60	0
0	448	0	0	40	23	11
0	0	0	0	0	0	0
0	0	0	0	11	0	0
0	0	0	10	50	0	0
0	0	0	0	0	36	0
0	0	0	0	0	142	0
0	164	57	0	0	31	0
0	22	79	0	19	0	0
0	304	45	131	49	625	0
0	157	0	14	0	214	0
0	199	104	97	71	610	0
0	10	24	0	0	169	0
110	16	0	0	0	0	0
0	12	0	0	0	0	0
0	42	0	0	0	0	0
0	88	0	0	0	0	0
12	0	0	0	0	0	0
0	0	0	0	0	0	0
0	0	0	0	0	0	0
0	58	273	0	13	0	0
0	60	300	23	46	312	0
0	27	165	0	0	35	0
0	438	34	0	0	176	10
0	145	26	0	0	0	0
0	31	0	0	0	0	0
0	0	0	0	0	0	0
0	41	83	0	0	0	53
0	23	69	0	0	0	25

0	0	0	0	0	0	0
0	563	152	14	0	420	0
0	0	0	0	11	0	0
0	38	38	0	0	40	0
0	153	0	0	0	0	0
0	58	10	57	35	0	11
0	0	0	0	0	49	0
0	32	0	0	0	10	0
0	116	46	42	0	161	0
59	93	0	0	25	10	0
0	117	0	0	0	0	0
10	10	0	0	0	0	0
0	0	0	0	0	0	0
0	0	0	0	0	0	0
0	0	0	0	23	0	0
0	0	0	0	0	0	0
53	74	0	0	40	15	41
0	38	11	0	10	25	0
0	39	140	0	0	0	0
0	27	0	86	0	12	0
0	47	129	53	143	56	0
0	0	0	0	0	0	0
0	0	0	10	0	35	0
59	0	0	0	67	0	0
0	0	0	0	0	0	0
0	107	0	0	12	0	44
0	17	0	0	0	0	0
0	256	0	0	0	0	79
0	10	0	0	76	0	0
0	15	0	0	20	0	0
0	38	0	0	159	0	0
0	184	0	0	598	61	0
0	34	0	0	71	0	0
0	0	0	0	102	0	0
0	23	0	0	0	0	0
0	0	0	0	0	0	0
0	486	121	130	0	0	224
59	1395	132	132	62	0	317
0	79	0	0	0	0	11
11	115	14	49	0	0	35
0	114	0	0	0	0	67
0	381	11	123	149	0	0
0	0	0	0	31	0	0
0	0	0	0	85	0	0
0	183	35	33	84	0	0
0	765	74	532	0	13	0

0	0	0	0	0	0	0
0	43	0	0	0	0	0
26	118	22	37	10	16	49
0	121	0	0	10	0	24
0	119	24	24	15	0	10
0	72	0	15	0	0	20
0	0	0	0	0	0	0
748	12	0	0	0	14	0
0	0	0	0	0	17	0
41	10	0	0	0	272	0
453	0	10	0	0	0	0
0	0	79	0	0	11	30
0	0	0	0	0	15	0
0	0	0	0	0	10	10
0	0	13	0	0	42	12
0	0	15	0	12	19	0
0	52	0	25	0	85	0
0	0	0	0	0	0	0
0	0	0	0	0	0	0
0	0	0	0	0	0	0
0	0	0	0	0	0	0
0	12	0	0	0	0	0
0	0	0	0	0	0	0
0	0	0	0	0	0	0
0	0	0	0	0	0	0
0	0	0	0	0	0	0
33	0	0	0	0	0	0
0	12	0	0	0	0	0
0	0	0	0	0	97	0
0	0	0	0	0	0	0
0	0	0	0	26	381	0
0	0	0	0	27	109	0
0	0	0	0	0	0	0
0	0	0	0	0	0	0
142	32	55	120	15	1453	53
0	0	0	97	0	128	0
0	0	0	0	0	0	0
0	0	0	0	0	195	0
37	51	268	24	24	44	29
0	0	0	11	0	260	0
0	0	0	0	0	480	0
10	18	0	0	0	10	0
0	0	0	0	0	11	0
0	0	0	0	0	0	0
10	22	0	12	95	0	0
0	0	0	0	0	0	0

0	0	0	0	0	0	55
0	0	0	0	0	0	50
0	0	0	0	0	0	0
0	0	0	0	10	0	0
0	0	0	0	14	324	0
0	0	0	0	0	276	0
0	10	0	0	0	0	0
0	21	0	0	0	52	0
0	0	0	0	0	0	0
0	0	0	0	0	0	0
0	0	0	0	0	0	0
0	0	0	0	0	65	0
0	0	0	0	101	302	0
0	24	0	0	0	0	0
0	0	0	0	0	174	0
0	0	0	0	124	126	0
0	0	0	0	91	215	0
11	0	0	0	204	0	0
0	0	0	0	0	0	0
0	0	0	0	20	0	0
0	0	0	0	0	0	17
0	0	0	0	0	0	0
0	0	0	0	737	0	0
0	0	44	0	0	0	53
42	112	0	0	0	0	54
33	12	0	0	0	0	10
0	0	0	0	0	0	0
0	0	12	0	0	29	0
0	0	0	0	0	0	0
0	0	0	0	0	10	0
0	0	0	0	0	0	0
0	0	0	0	30	29	0
0	0	0	0	0	0	0
0	0	0	0	22	0	0
0	25	0	0	12	0	0
0	0	0	0	0	0	0
0	0	0	0	581	10	0
0	0	0	0	10	0	0
0	0	0	0	14	0	0
0	0	0	0	0	0	0
92	11	50	22	41	93	229
32	0	86	0	0	122	164
35	0	22	0	26	0	0
0	0	0	0	0	24	20
0	0	0	0	0	20	10
0	0	0	0	0	0	0

0	0	0	0	0	10	0
0	0	0	0	0	0	0
0	237	0	0	0	44	0
0	26	0	10	0	0	0
0	58	0	0	0	0	0
0	168	130	0	10	0	0
0	0	0	0	0	0	0
0	18	0	0	0	73	0
0	140	24	20	40	44	0
0	109	0	0	0	20	0
0	40	0	0	10	0	0
0	0	0	0	0	0	0
0	0	0	0	0	35	0
0	0	0	0	0	46	0
0	69	13	0	15	127	18
10	26	28	0	0	56	0
62	0	0	0	0	0	0
10	0	0	0	17	109	0
0	0	0	0	0	0	0
0	0	0	0	0	0	0
0	0	0	0	0	0	0
0	0	0	11	15	72	0
0	12	0	0	0	0	0
0	0	0	0	0	0	0
0	0	0	0	0	0	0
0	0	0	0	0	0	0
0	0	0	0	0	0	0
140	108	0	283	44	10	0
0	0	0	0	0	0	0
0	0	0	0	26	21	0
0	188	221	50	140	182	0
0	14	114	25	123	0	234
0	135	128	12	16	277	0
0	0	342	0	0	0	72
0	33	0	0	0	14	24
0	0	0	0	0	0	0
0	0	0	0	0	0	0
0	0	0	0	15	0	0
0	0	0	0	0	25	0
0	0	0	0	0	0	0
153	0	0	0	50	0	0
0	39	0	0	0	39	0
0	10	0	0	0	64	0
0	10	0	0	0	38	0
0	58	0	0	0	0	0
0	0	0	0	0	0	0

0	50	0	0	23	10	0
0	0	0	0	0	93	0
0	0	0	0	0	0	0
0	0	0	0	0	0	0
0	20	40	21	14	81	0
0	0	0	0	0	128	0
14	0	0	0	0	12	11
0	0	0	0	0	29	0
69	0	0	0	128	0	0
37	0	0	0	0	22	0
0	0	0	0	0	0	0
28	0	25	0	0	52	0
0	0	0	0	0	0	0
13	0	0	0	53	23	0
0	0	28	0	0	49	20
0	17	0	0	11	143	0
0	0	0	0	0	0	0
0	0	0	0	0	0	0
100	30	0	69	0	0	0
0	0	0	0	0	0	0
0	0	0	0	0	0	0
11	0	0	0	0	38	0
126	17	0	0	0	0	0
0	37	0	0	0	0	0
0	20	57	24	0	0	12
0	0	0	0	0	10	0
0	0	0	0	0	0	0
0	0	0	0	0	0	0
0	28	0	0	0	0	0
0	36	0	0	0	0	0
0	0	31	0	0	0	0
0	83	0	0	0	0	0
0	26	26	0	10	0	0
0	64	0	0	0	0	0
0	0	0	0	0	0	0
0	262	218	0	0	0	267
0	83	29	0	0	0	46
0	39	0	0	0	0	0
0	0	0	0	0	0	0
0	0	0	0	11	0	0
0	0	0	0	0	0	0
0	0	0	0	0	0	0
0	0	0	0	0	0	0
0	0	0	0	10	0	0
10	0	0	0	0	0	0
0	0	0	0	0	54	0

0	0	0	0	0	0	11
0	0	0	0	0	0	0
15	11	0	0	0	0	0
0	79	58	61	16	16	0
416	0	10	0	0	26	0
0	0	10	0	0	0	0
0	27	0	0	0	0	0
0	0	18	0	0	0	0
0	0	0	0	0	38	0
0	35	0	0	0	0	0
0	0	0	0	0	0	0
0	0	0	0	0	14	0
0	0	0	0	0	0	0
0	0	0	12	0	0	0
0	55	0	183	0	0	0
0	0	0	0	0	0	0
0	0	0	0	22	0	0
0	0	0	0	0	0	0
0	0	0	0	82	45	0
0	0	0	12	0	22	0
0	0	0	0	0	0	0
0	67	0	0	0	0	0
0	0	0	0	0	13	0
0	0	0	0	0	13	0
31	0	0	0	0	0	0
195	11	0	0	0	0	0
108	0	0	13	0	0	0
0	0	0	0	0	0	0
0	0	0	0	0	0	0
0	10	0	0	0	266	0
0	0	0	0	0	0	0
0	0	0	0	0	0	0
0	0	0	0	0	0	0
22	143	24	109	0	195	0
0	0	0	0	0	0	0
0	0	0	11	0	149	0
0	0	0	0	0	0	0
0	0	0	0	0	0	0
0	0	0	10	70	0	0
0	0	0	0	0	0	34
134	2274	202	0	240	28	34
0	0	0	0	0	0	0
0	0	0	0	0	0	0
0	0	0	0	0	0	0
0	0	0	0	0	0	0
0	0	0	0	49	0	0

38	0	0	0	0	467	0
11	0	0	0	0	0	0
0	0	0	0	0	0	0
86	0	0	0	0	0	0
35	0	0	0	0	0	0
0	0	0	0	0	0	0
0	37	10	0	0	0	13
0	105	0	0	0	0	0
0	15	0	0	0	0	28
0	388	108	0	10	73	37
0	0	0	0	0	0	0
0	0	0	0	0	0	0
0	53	0	0	0	35	0
0	11	0	0	0	27	0
0	0	0	0	0	0	0
0	0	0	0	0	0	0
0	23	0	0	32	0	0
0	37	0	0	0	0	18
0	178	307	91	27	0	108
48	51	63	55	0	0	0
0	147	52	40	12	0	0
0	0	0	0	28	0	0
0	0	0	0	0	0	0
0	0	0	0	0	0	0
0	0	0	0	0	0	0
13	0	0	0	0	0	0
0	189	10	38	23	0	0
17	60	0	0	0	0	0
0	0	0	0	0	0	0
171	0	0	0	0	0	0
0	0	0	0	0	0	0
0	0	0	0	0	0	0
0	0	0	10	0	11	32
0	0	0	0	0	0	26
0	0	145	36	0	0	0
0	51	423	44	0	39	288
0	0	0	0	0	0	0
0	0	0	0	13	0	0
0	0	0	0	0	0	0
0	0	0	0	0	0	0
0	0	0	0	0	0	0
0	23	0	0	0	0	0
0	0	0	0	92	0	0
0	0	0	0	0	0	0
0	0	0	0	72	0	0
0	34	0	0	343	0	0

0	0	0	0	0	0	0
0	0	0	0	0	0	0
0	0	0	0	0	0	0
0	0	0	0	0	0	0
0	0	0	0	10	0	0
0	0	0	0	0	0	0
0	0	0	0	0	0	0
0	0	0	0	0	0	0
0	0	0	0	0	0	0
0	0	0	14	30	0	0
0	0	0	11	0	22	0
0	430	573	81	0	304	0
0	0	0	0	120	0	0
13	315	84	12	573	0	0
94	61	611	0	50	0	289
0	0	0	0	0	0	0
0	10	0	0	0	0	0
0	0	0	0	0	0	0
0	0	0	0	53	0	0
0	0	0	0	0	0	0
0	0	0	0	0	0	0
0	45	0	0	0	0	0
0	0	0	0	0	0	0
0	0	0	0	41	0	0
0	0	0	0	0	0	0
0	0	0	0	0	0	0
0	0	0	0	0	0	0
0	0	0	0	0	0	0
0	0	0	0	47	10	0
0	22	10	0	32	82	0
0	0	0	0	0	0	0
0	0	0	0	59	0	0
0	0	0	0	18	0	0
0	0	0	0	0	0	0
0	39	0	0	0	0	0
0	30	22	0	0	0	0
0	0	0	0	38	0	0
0	0	0	0	35	0	0
0	0	0	0	23	0	0
0	0	0	0	0	0	0
0	0	0	0	11	0	0
0	0	0	0	11	0	0
15	0	0	0	31	0	0
0	0	0	0	0	0	0
0	0	0	0	30	0	0
0	0	0	0	0	0	0

0	10	0	0	32	0	0
0	28	0	0	41	0	0
0	0	0	0	0	0	0
0	0	0	0	10	0	0
0	0	0	0	10	0	0
0	11	0	0	44	10	0
0	0	0	0	0	0	0
0	0	0	0	0	0	0
0	63	0	0	14	0	12
0	0	0	0	0	0	0
0	0	0	0	27	0	0
0	0	0	0	0	0	0
0	11	0	0	0	0	0
0	79	0	0	0	0	184
0	0	0	0	0	0	0
0	0	0	0	0	0	0
0	115	83	0	0	0	432
0	0	0	0	22	0	0
0	0	0	0	0	0	0
0	27	0	0	0	12	0
0	21	0	0	0	0	0
0	0	0	0	0	0	0
0	0	0	0	0	0	0
0	0	0	0	126	0	0
0	0	0	0	186	0	0
0	0	0	0	52	0	0
0	0	0	0	0	0	0
0	0	0	0	11	0	0
0	0	0	0	0	0	0
0	0	0	0	0	400	0
0	0	0	0	11	0	0
0	0	0	0	0	21	0
0	0	0	0	0	0	0
0	0	0	0	61	0	0
0	0	0	0	0	0	0
0	0	0	0	18	0	0
0	0	0	0	0	0	0
0	0	0	0	0	0	0
0	0	0	0	0	0	0
0	55	0	0	49	45	0
0	0	0	0	11	0	0
0	0	0	0	0	0	0
0	16	0	0	0	0	0
0	0	0	0	0	0	0
0	0	0	0	0	0	0
0	79	0	0	0	0	0

0	27	0	0	0	0	0
0	32	0	0	0	0	0
0	0	0	0	0	10	0
0	0	0	0	0	40	0
0	0	0	0	0	0	0
0	0	0	0	0	0	0
0	0	0	0	0	0	0
0	0	0	0	0	0	0
18	25	0	0	0	0	0
0	0	0	0	0	0	0
0	0	0	0	0	0	0
0	0	0	0	0	156	0
0	0	0	0	0	20	0
0	0	0	0	0	89	0
0	0	0	0	0	0	0
0	0	0	0	0	0	0
0	0	0	0	0	118	0
52	0	0	0	0	0	0
0	0	0	0	0	0	0
0	0	0	0	0	0	0
0	0	0	0	0	366	0
0	0	15	0	0	0	0
0	0	0	0	0	0	0
0	11	0	0	0	0	0
0	0	0	0	0	10	0
0	0	0	0	0	63	0
0	0	0	0	0	0	0
0	0	0	0	0	0	0
0	80	0	0	0	0	0
0	18	0	0	0	0	0
0	0	0	28	0	61	0
0	0	0	0	0	0	0
0	0	11	0	0	0	0
0	111	0	13	0	0	0
0	97	0	23	0	0	0
0	65	0	0	0	0	0
0	462	0	0	0	0	0
0	0	0	0	0	0	0
0	87	0	0	0	0	0
0	0	0	0	28	415	0
0	0	0	0	0	0	0
0	0	0	0	0	0	0
0	67	0	10	0	0	0
0	0	0	0	0	330	0
0	0	0	0	0	0	0
0	0	0	0	0	0	0

0	0	0	0	0	0	0
0	10	40	0	0	66	0
0	0	15	0	31	13	0
0	11	0	0	0	108	0
0	116	0	0	0	0	0
0	0	0	0	0	0	0
0	0	0	0	0	0	0
0	0	0	0	0	0	0
0	12	0	0	0	0	0
0	0	0	0	0	0	0
0	0	0	0	0	0	0
0	22	0	0	0	14	0
0	0	0	0	0	0	0
0	0	0	0	0	0	0
0	0	12	0	25	0	0
0	11	0	0	0	0	0
0	0	0	0	12	0	0
0	0	0	0	0	0	0
0	33	0	11	13	0	0
0	74	21	16	32	50	0
0	0	0	0	0	0	0
0	0	0	0	0	0	0
0	952	6065	0	0	0	950
0	1385	15719	0	20	0	2122
0	0	0	0	0	0	0
0	0	0	0	0	0	0
0	0	0	0	0	0	0
0	0	0	0	0	0	0
0	0	0	0	0	0	0
0	0	0	0	0	0	0
0	45	0	0	0	10	0
0	0	0	0	0	0	0
0	33	0	10	0	0	0
30	25	0	0	0	0	14
0	66	0	0	0	0	10
0	84	0	0	0	0	10
0	27	0	0	0	0	28
0	132	10	41	0	0	67
0	0	33	0	0	0	30
0	44	0	0	0	0	0
0	0	0	0	0	0	10
0	350	0	0	0	0	0
0	0	0	0	0	0	0
0	0	0	0	0	0	25
0	52	0	0	43	0	10
15	393	0	0	0	0	109

0	0	0	0	0	0	0
0	0	0	0	0	0	0
0	0	0	0	0	12	0
0	0	0	0	0	0	0
0	0	0	0	0	0	0
0	0	0	0	0	0	0
0	0	0	0	0	11	0
0	70	0	0	0	0	0
0	32	0	0	0	0	0
0	0	0	0	0	0	0
0	0	0	0	0	0	0
0	0	0	0	0	0	0
0	0	0	0	0	0	0
0	55	0	0	0	0	0
0	0	0	0	0	0	0
0	200	0	0	0	0	0
0	0	0	0	0	0	0
71	0	0	0	0	0	0
0	0	0	0	0	0	0
10	0	0	0	0	0	0
0	113	0	0	0	126	0
0	10	0	0	0	0	0
0	29	0	0	0	0	0
0	0	0	0	0	0	0
0	0	0	0	0	0	0
12	0	0	0	0	0	0
0	0	0	0	0	0	0
0	0	0	0	0	0	0
110	0	0	0	0	0	0
0	0	0	0	0	0	0
0	0	0	0	12	0	0
0	0	0	0	10	10	0
0	30	0	0	0	0	0
220	0	0	0	36	0	0
0	0	0	0	0	0	0
47	0	0	0	0	0	0
21	0	0	0	0	0	0
109	0	0	11	96	0	0
0	0	0	0	0	21	0
17	0	0	0	0	0	10
0	0	0	0	0	0	0
0	0	0	0	0	0	0
0	0	0	0	0	0	0
0	0	0	0	0	0	0
0	0	0	0	0	0	0
0	0	0	0	0	0	0

0	0	0	0	0	0	0
0	14	0	0	0	0	0
0	0	20	0	0	0	0
0	34	0	0	0	0	0
0	45	0	0	0	0	0
0	62	0	0	0	0	0
0	12	0	0	0	0	0
0	15	188	0	0	0	83
0	11	0	0	0	0	0
0	20	0	0	0	0	0
0	11	0	0	0	0	0
0	28	0	0	0	0	0
0	18	0	0	0	0	0
0	28	0	0	0	0	0
0	12	0	0	0	0	0
0	74	0	0	0	0	0
0	13	0	0	0	0	0
0	17	370	0	0	0	167
0	10	0	0	0	0	0
0	76	0	0	0	0	0
0	10	0	0	0	0	0
0	10	0	0	0	0	0
0	10	0	0	0	0	0
0	170	326	10	92	10	0
10	12	0	13	0	0	0
0	11	0	0	0	32	0
0	22	0	0	0	0	0
0	55	0	0	0	42	0
0	24	0	0	0	38	0
0	398	13	42	0	190	0
0	21	0	0	0	10	0
47	11	0	0	0	0	0
0	10	0	0	0	0	0
0	100	0	0	0	1511	0
0	22	0	0	0	267	0
0	117	0	0	0	0	0
0	10	0	0	0	0	0
0	47	29	0	0	0	119
0	46	0	0	0	0	0
0	105	242	0	0	0	130
0	31	0	0	0	0	0
0	10	0	0	0	0	0
0	30	0	0	0	0	0
0	49	0	0	0	0	0
0	42	0	0	0	0	0
0	40	0	0	0	0	0

0	32	0	0	125	70	10
0	306	0	0	0	0	0
0	65	0	0	0	0	0
0	10	0	0	0	0	0
0	10	49	0	0	47	68
0	64	356	11	28	0	0
0	13	0	0	0	0	0
0	47	0	0	0	0	0
0	15	0	0	0	0	0
0	104	0	0	0	0	0
0	944	0	0	0	0	0
0	12	0	0	0	0	0
0	30	0	0	0	0	0
0	1159	0	0	0	0	0
0	3156	0	0	0	0	0
0	10	0	0	0	0	0
0	14	0	0	0	0	0
0	12	0	0	0	0	0
0	10	0	0	0	0	0
0	24	0	0	0	0	0
0	14	0	0	0	0	0
0	15	0	0	10	0	0
0	48	0	0	38	0	0
0	64	0	0	0	0	0
0	13	0	0	0	0	0
0	137	0	0	0	0	0
0	53	154	0	0	0	0
0	39	36	0	0	0	14
0	11	0	0	0	0	0
0	10	0	0	0	15	0
0	23	0	0	0	0	0
0	26	0	0	0	0	0
0	14	0	0	0	0	0
0	23	0	0	0	0	0
0	11	0	0	0	0	11
0	15	0	0	0	0	0
0	50	0	0	0	0	13
0	12	0	0	0	0	0
0	12	0	0	0	0	0
0	13	0	0	0	0	0
0	11	0	0	0	0	0
0	13	0	0	0	0	0
0	10	0	0	0	0	0
0	37	0	0	0	0	0
0	10	0	0	0	0	0
0	29	0	0	0	0	0

0	18	0	0	0	0	0
0	73	0	11	0	0	0
0	27	0	0	0	0	0
0	10	0	0	0	0	0
0	12	0	0	0	0	0
0	10	0	0	0	0	0
0	22	0	0	0	0	0
0	29	0	0	0	0	0
0	12	0	0	0	0	0
0	197	0	0	0	0	0
0	15	0	0	0	0	0
0	35	0	24	0	0	0
0	0	0	0	0	0	0
0	0	0	0	58	27	0
0	0	114	0	0	0	0
0	0	0	0	0	214	0
0	0	0	0	37	104	0
0	0	0	0	0	41	0
0	0	0	0	0	84	0
0	0	0	77	0	71	10
0	0	0	0	0	0	0
0	0	10	0	14	10	0
0	0	0	13	0	254	0
0	0	0	0	0	26	0
0	0	0	0	0	421	0
0	0	0	0	0	0	0
0	0	0	0	0	0	0
0	0	0	0	0	0	0
39	0	0	0	0	0	0
0	0	0	0	0	0	0
0	0	0	0	0	0	0
0	0	0	0	0	0	0
0	0	0	0	0	0	0
0	0	0	0	0	0	0
0	0	0	0	0	0	0
0	0	45	0	10	0	0
0	0	0	0	0	0	0
0	0	0	0	0	10	0
0	0	0	0	0	201	0
0	0	0	0	0	0	0
0	0	0	0	0	0	0
0	0	0	0	0	0	0
0	0	0	0	0	0	0
0	0	0	0	0	0	0
0	0	0	0	0	0	0
0	0	0	0	0	0	0

0	0	0	0	0	16	0
0	0	0	0	18	0	0
0	0	0	0	0	0	0
0	0	0	0	0	0	0
0	0	46	0	0	60	0
0	0	0	0	0	0	0
0	0	0	0	0	15	13
0	0	0	0	0	16	0
0	0	12	0	0	0	0
0	0	103	0	0	0	907
0	0	0	0	0	37	0
0	0	0	0	0	0	0
0	0	0	0	0	10	0
0	0	0	0	12	0	0
0	0	0	0	0	0	0
0	0	0	0	0	0	0
52	0	0	0	0	0	0
0	0	0	0	0	0	0
0	0	0	0	0	0	0
0	0	0	0	0	34	0
0	0	0	0	0	0	0
0	0	0	0	0	0	0
0	0	0	0	0	39	0
0	0	0	0	0	0	0
0	0	0	0	0	13	0
0	0	0	0	0	0	0
102	0	0	0	0	0	0
0	0	0	0	38	751	0
0	0	0	0	0	0	0
0	0	0	0	0	0	0
0	0	0	0	0	0	0
0	0	0	0	0	0	0
0	0	11	0	0	0	0
0	0	0	0	0	0	0
0	0	0	0	0	0	0
0	0	0	0	0	0	0
0	0	0	0	0	0	0
0	0	0	0	0	0	0
0	0	0	0	0	0	0
0	0	0	0	0	0	0
0	0	0	0	0	0	0
0	0	0	0	0	0	0
0	0	0	0	0	0	0
13	0	0	0	0	0	0
0	0	0	0	0	0	0
0	0	0	0	0	0	0

22	0	0	0	0	0	0
0	0	0	0	0	0	0
0	0	0	0	0	0	0
0	0	0	0	0	15	0
0	0	0	0	0	0	0
0	0	0	0	0	0	0
0	0	0	0	0	46	0
0	0	0	0	0	0	0
53	0	0	0	0	0	0
0	0	0	0	0	0	0
0	0	0	0	0	0	0
0	0	0	0	0	0	0
0	0	10	0	0	0	0
0	0	0	0	0	0	0
0	0	0	0	0	0	0
0	0	0	0	0	0	0
0	0	0	0	0	0	0
0	0	0	0	0	0	0
101	0	0	0	0	0	0
0	0	0	0	0	0	0
0	0	0	0	0	0	0
0	0	0	0	0	0	0
0	0	0	0	0	0	0
0	0	0	0	0	0	0
0	0	0	0	0	0	0
0	0	0	0	0	0	0
0	0	0	0	0	0	0
0	0	0	0	0	0	0
0	0	0	0	0	0	0
0	0	0	0	0	0	0
0	0	0	0	0	0	0
0	0	0	0	0	0	0
0	0	0	0	0	0	0
0	0	0	0	0	0	0
0	0	0	0	0	0	0
0	0	0	0	0	0	0
0	0	0	0	0	0	0
0	0	0	0	0	0	0
0	0	0	0	0	0	0
0	0	0	0	0	0	0
0	0	0	13	0	0	0
0	0	0	36	0	0	0
0	0	0	0	0	0	0
0	0	0	0	0	0	0
0	0	0	0	0	0	0
0	0	0	0	0	0	0

0	0	0	0	0	0	0
0	0	0	0	0	0	0
0	0	0	0	0	0	0
0	0	0	0	0	0	0
0	0	0	0	0	0	0
0	0	0	0	0	77	0
0	0	0	0	0	0	0
0	0	0	0	0	0	0
0	0	0	0	0	0	0
0	0	0	0	0	0	0
0	0	0	0	0	0	0
0	0	0	0	0	0	0
0	0	0	0	0	0	0
0	0	0	0	0	0	0
0	0	0	0	0	0	0
0	0	0	0	0	0	0
0	0	0	0	0	0	0
0	0	0	0	0	0	0
0	0	0	0	0	0	0
0	0	0	0	0	0	0
0	0	0	0	0	0	0
0	0	49	0	0	0	0
0	0	0	0	0	0	0
0	0	0	0	0	0	0
0	0	0	0	0	0	0
0	0	0	0	0	0	0
0	0	0	0	0	0	0
0	0	0	0	0	0	0
0	0	0	0	0	0	0
0	0	0	0	0	0	0
0	0	0	0	0	0	0
0	0	0	0	0	0	0
0	0	0	0	0	0	0
0	0	0	0	0	0	0
0	0	0	0	0	0	0
0	0	0	0	0	0	0
0	0	0	0	0	0	0
0	0	0	0	0	0	0
0	0	0	0	0	0	0
0	0	0	0	0	0	0
0	0	0	0	0	0	0
0	0	0	0	0	0	0
0	0	0	0	0	0	0
0	0	0	0	0	0	0
0	0	0	0	0	0	0
0	0	0	0	0	0	0

0	0	0	0	0	0	0
0	0	0	0	0	0	0
0	0	0	0	0	0	0
0	0	0	0	0	0	0
0	0	0	0	0	0	0
0	0	0	0	0	0	0
0	0	0	0	0	0	0
0	0	0	0	0	0	0
0	0	0	0	0	94	0
0	0	0	0	0	0	0
0	0	0	0	0	0	0
0	0	0	0	0	0	0
0	0	0	0	0	0	0
0	0	0	0	0	0	0
0	0	0	0	0	0	0
0	0	0	0	0	0	0
0	0	0	0	0	0	0
0	0	0	0	0	0	0
0	0	26	0	0	23	31
0	0	0	0	0	0	10
0	0	0	0	0	0	26
0	0	0	0	0	0	19
0	0	0	0	0	0	11
0	0	0	0	0	0	10
0	0	33	0	0	0	72
0	0	26	0	26	0	149
0	0	0	0	0	0	27
0	0	0	0	0	0	39
0	0	0	0	0	0	10
0	0	0	0	0	0	47
0	0	0	0	0	0	28
0	0	0	0	0	0	35
0	0	0	0	0	0	23
0	0	0	0	0	0	18
0	0	0	0	0	30	35
0	0	0	0	0	0	0
0	0	0	33	0	0	0
0	0	0	0	0	0	0
0	0	0	0	0	0	0
0	0	0	0	0	10	0
0	0	0	0	0	0	0
0	0	0	0	0	0	0
0	0	0	0	0	0	0
0	0	0	0	11	70	0
0	0	0	0	0	44	0
0	0	0	0	0	33	0

0	0	0	0	0	0	0
0	0	0	0	0	0	0
0	0	0	0	0	0	0
0	0	0	0	0	0	0
0	0	0	0	0	0	0
0	0	0	0	0	0	0
0	0	0	0	0	0	0
0	0	0	0	0	0	0
0	0	0	0	0	0	0
0	0	0	0	0	0	0
0	0	0	0	0	0	0
0	0	0	0	0	0	0
0	0	0	0	0	0	0
0	0	0	0	0	0	0
0	0	0	0	0	41	0
0	0	0	0	0	0	0
0	0	0	0	0	0	0
0	0	0	0	0	125	0
0	0	0	0	0	0	0
0	0	0	0	0	0	0
0	0	0	0	0	0	0
0	0	0	0	0	0	0
0	0	0	0	0	0	0
0	0	0	0	0	0	0
0	0	0	0	11	0	0
0	0	0	0	0	0	0
0	0	0	17	0	0	0
0	0	0	0	0	0	0
0	0	0	0	0	0	0
0	0	0	0	0	0	0
0	0	0	0	0	0	0
0	0	0	0	0	0	0
0	0	0	0	0	0	0
0	0	0	0	0	521	0
0	0	0	0	0	10	0
0	0	0	0	0	0	0
0	0	0	0	0	0	0
0	0	0	0	0	0	0
0	0	0	0	0	61	0
0	0	0	0	0	0	0
0	0	0	0	0	31	0
0	0	0	0	0	0	0
0	0	0	0	0	0	0
0	0	0	0	0	0	0
0	0	0	0	0	0	0
0	0	0	0	0	0	0

0	0	0	0	0	0	0
0	0	0	0	0	0	0
0	0	0	0	0	16	0
0	0	0	0	0	0	0
0	0	0	0	0	0	0
0	0	0	0	0	0	0
0	0	0	0	0	0	0
0	0	0	0	0	0	0
0	0	0	0	0	0	0
0	0	0	0	0	0	0
0	0	10	0	0	0	0
0	0	0	11	0	0	0
0	0	0	0	0	0	0
0	0	0	0	0	0	0
0	0	0	0	0	0	0
0	0	0	0	0	104	0
0	0	0	0	0	0	0
0	0	0	0	0	0	0
0	0	0	0	0	0	0
0	0	0	0	0	0	0
0	0	0	0	0	240	0
0	0	0	0	0	92	0
0	0	0	0	0	0	0
0	0	0	0	0	0	0
0	0	0	0	0	0	0
0	0	0	0	0	0	0
0	0	0	0	0	0	0
0	0	0	0	13	129	0
0	0	0	0	0	0	0
0	0	0	0	0	0	0
0	0	0	0	0	0	0
0	0	0	0	0	0	0
0	0	0	0	0	0	0
0	0	0	0	0	0	0
0	0	0	0	0	0	0
0	0	0	0	0	0	0
0	0	0	0	0	0	0
0	0	0	0	0	0	0
0	0	0	0	0	0	0
0	0	0	0	0	0	0
0	0	0	0	0	0	0
0	0	0	0	0	0	0
0	0	0	0	0	0	0
0	0	0	0	0	0	0
0	0	0	0	0	0	0
0	0	0	0	0	0	0

0	0	0	0	0	0	0
0	0	0	0	0	0	0
0	0	0	0	0	0	0
0	0	0	0	0	0	0
0	0	0	0	0	0	0
0	0	0	0	0	0	0
0	0	0	0	0	12	0
0	0	0	0	0	0	0
0	0	0	0	0	0	0
0	0	0	0	0	0	0
0	0	0	0	0	61	0
0	0	0	0	0	0	0
0	0	0	0	0	0	0
0	0	0	0	0	0	0
0	0	0	0	0	11	0
0	0	0	0	10	0	0
0	0	0	0	0	0	0
0	0	0	0	0	0	0
0	0	0	0	0	0	0
0	0	0	0	0	13	0
0	0	0	0	0	0	0
0	0	0	0	0	0	0
0	0	15	0	0	0	0
0	0	15	0	0	0	0
0	0	0	0	0	0	0
0	0	0	0	0	0	0
0	0	23	0	0	0	0
0	0	0	0	0	0	0
0	0	0	0	0	0	0
0	0	0	0	0	0	0
0	0	0	0	0	0	0
0	0	0	0	0	0	0
0	0	0	0	0	0	0
0	0	0	0	0	0	0
0	0	0	0	0	0	0
0	0	0	0	0	0	0
0	0	0	0	0	10	0
0	0	0	0	0	0	0
0	0	0	0	0	0	0
0	0	0	0	0	0	0
0	0	0	0	0	0	0
0	0	0	0	0	0	0
0	0	0	0	0	10	0
0	0	0	0	0	26	0
0	0	0	0	0	0	0
0	0	0	0	0	0	0

0	0	0	0	0	0	0
0	0	0	0	0	0	0
0	0	0	0	0	0	0
0	0	0	0	0	0	0
0	0	0	0	0	0	0
0	0	0	0	0	0	0
0	0	0	0	0	0	0
0	0	0	0	0	0	0
0	0	0	0	0	0	0
0	0	0	0	0	0	0
0	0	0	0	0	0	0
0	0	0	0	0	0	0
0	0	0	0	0	0	0
0	0	0	0	0	0	0
0	0	0	0	0	0	0
0	0	0	0	0	0	0
0	0	0	0	0	0	0
0	0	0	0	0	12	0
0	0	0	0	0	11	0
0	0	0	0	0	0	0
0	0	0	0	0	0	0
0	0	0	0	0	0	0
0	0	0	0	0	46	0
0	0	0	0	0	11	0
0	0	0	0	0	0	0
0	0	0	0	0	0	0
0	0	0	0	0	0	0
0	0	0	0	0	0	0
0	0	0	0	0	0	0
0	0	0	0	0	0	0
0	0	0	0	0	0	0
0	0	0	0	0	0	0
0	0	0	0	0	0	0
0	0	0	0	0	0	0
0	0	0	0	0	0	0
0	0	0	0	0	47	0
0	0	0	0	0	0	0
0	0	0	0	0	0	0
0	0	0	0	0	0	0
0	0	0	0	0	0	0
0	0	0	0	0	0	0
0	0	0	0	0	0	0
0	0	0	0	0	0	0
0	0	0	0	0	0	0
0	0	0	0	0	0	0
0	0	0	0	0	0	0

0	0	0	0	0	0	0
0	0	0	0	0	75	0
0	0	0	0	0	0	0
0	0	0	0	0	0	0
0	0	0	0	0	0	0
0	0	0	0	0	10	0
0	0	0	0	0	0	0
0	0	0	0	0	0	0
0	0	0	0	0	0	0
0	0	0	0	0	34	0
0	0	0	0	0	0	0
0	0	0	0	0	0	0
0	0	0	0	0	0	0
0	0	0	0	0	0	0
0	0	0	0	0	0	0
0	0	0	0	0	0	0
0	0	0	0	0	0	0
0	0	0	0	0	0	0
0	0	0	0	0	0	0
0	0	0	0	0	0	0
0	0	0	0	0	0	0
0	0	0	0	0	0	0
0	0	0	0	0	0	0
0	0	0	0	0	11	0
12	0	0	0	0	0	0
0	0	0	0	0	0	0
0	0	0	0	0	0	0
0	0	0	0	0	0	0
0	0	0	0	0	0	0
0	0	0	0	0	0	0
0	0	0	0	0	0	0
0	0	0	0	0	31	0
0	0	0	0	0	0	0
10	0	0	0	0	0	0
0	0	0	0	0	0	0
0	0	0	0	0	0	0
0	0	0	0	0	0	0
0	0	0	0	0	0	0
0	0	0	0	0	0	0
0	0	0	0	0	0	0
0	0	0	0	0	0	0
0	0	0	0	0	0	0
0	0	0	0	0	0	0
0	0	0	0	0	0	0
0	0	44	12	0	0	0
0	0	0	0	0	0	0

0	0	0	0	0	0	0
0	0	0	0	0	0	0
0	0	0	0	0	0	0
0	0	0	0	0	76	0
0	0	0	0	33	0	0
0	0	0	0	0	0	0
0	0	0	0	0	0	0
0	0	0	0	25	0	0
0	0	0	0	0	0	0
0	0	0	0	0	0	0
0	0	0	0	0	122	0
0	0	0	0	0	116	0
0	0	0	0	0	38	0
0	0	0	0	0	54	0
0	0	0	0	0	40	0
0	0	0	0	0	0	0
0	0	0	0	0	78	0
0	0	0	25	0	0	0
0	0	0	0	0	0	0
0	0	0	0	0	0	0
0	0	0	0	0	0	0
0	0	0	0	0	0	0
0	0	0	0	0	0	0
0	0	0	0	0	0	0
0	0	0	0	0	0	0
0	0	0	0	0	0	0
0	0	0	0	0	0	0
0	0	0	0	0	0	0
0	0	0	0	0	0	0
0	0	0	0	0	22	0
0	0	0	0	0	11	0
0	0	0	0	0	24	0
0	0	0	0	0	27	0
0	0	0	0	0	31	0
0	0	0	0	0	38	0
0	0	0	0	0	113	0
0	0	0	0	0	13	0
0	0	0	0	0	30	0
0	0	0	0	0	271	0
0	0	0	0	0	104	0
0	0	0	0	0	12	0
0	0	0	0	0	63	0
0	0	0	0	0	24	0
0	0	0	0	0	12	0
0	0	0	0	0	42	0
0	0	0	0	0	32	0

0	0	0	0	0	10	0
0	0	0	0	0	44	0
0	0	0	0	0	12	0
0	0	0	0	0	158	0
0	0	0	0	0	27	0
0	0	0	0	0	97	0
0	0	0	0	0	28	0
0	0	0	0	0	26	0
0	0	0	0	0	13	0
0	0	0	0	0	31	0
0	0	0	0	0	75	0
0	0	0	0	0	36	0
0	0	0	0	0	72	0
0	0	0	0	0	29	0
0	0	0	0	0	21	0
0	0	0	0	0	73	0
0	0	0	0	0	30	0
0	0	0	0	0	11	0
0	0	0	0	0	129	0
0	0	0	0	0	20	0
0	0	0	0	0	77	0
0	0	0	0	0	43	0
0	0	0	0	0	105	0
0	0	0	0	0	12	0
0	0	0	0	0	22	0
0	0	0	0	0	24	0
0	0	0	0	0	45	0
0	0	0	0	0	10	0
0	0	0	0	0	50	0
0	0	0	0	0	12	0
0	0	0	0	0	24	0
0	0	0	0	0	10	0
0	0	0	0	0	41	0
0	0	0	0	0	37	0
0	0	0	0	0	102	0
0	0	0	0	0	66	0
0	0	0	13	0	79	0
0	0	0	0	0	125	0
0	0	0	0	0	32	0
0	0	0	0	0	16	0
0	0	0	0	0	57	0
0	0	0	0	0	10	0
0	0	0	0	0	169	0
0	0	0	0	0	15	0
0	0	0	0	0	11	0
0	0	0	0	0	16	0

0	0	0	0	0	10	0
0	0	0	0	0	25	0
0	0	0	0	0	11	0
0	0	0	0	0	64	0
0	0	0	0	0	15	0
0	0	0	0	0	18	0
0	0	0	0	0	108	0
0	0	0	0	0	24	0
0	0	0	0	0	12	0
0	0	0	0	0	14	0
0	0	0	0	0	254	0
0	0	0	0	0	41	0
0	0	0	0	0	61	0
0	0	0	0	0	0	0
0	0	0	0	0	0	0
0	0	0	0	0	0	0
0	0	0	0	0	0	0
0	0	0	0	0	0	0
0	0	0	0	0	0	0
0	0	0	0	0	0	0
0	0	0	0	0	0	0
0	0	0	0	0	0	0
0	0	0	0	0	0	0
0	0	0	0	0	0	0
0	0	0	0	0	0	0
0	0	0	0	0	0	0
0	0	0	0	0	0	0
0	0	0	0	0	0	0
13	0	0	0	0	0	0
11	0	0	0	0	0	0
23	0	0	0	0	0	0
33	0	0	0	0	0	0
10	0	0	0	0	0	0
11	0	0	0	0	0	0
10	0	0	0	0	0	0
39	0	0	0	0	0	0
44	0	0	0	0	0	0
0	0	0	0	0	0	0
0	0	0	0	0	0	0
0	0	0	0	0	0	0
0	0	0	0	0	0	0
0	0	0	0	0	0	0
0	0	0	0	0	0	0
0	0	0	0	0	0	0
0	0	0	0	0	0	0
0	0	0	0	0	0	0

0	0	0	0	0	0	0
0	0	0	0	0	0	0
0	0	0	0	0	0	0
0	0	0	0	0	0	0
0	0	0	0	0	0	0
0	0	0	0	0	0	0
0	0	0	0	0	0	0
0	0	0	0	0	0	0
0	0	0	0	0	0	0
0	0	0	0	0	0	0
0	0	0	0	0	0	0
0	0	0	0	0	0	0
0	0	0	0	0	0	0
0	0	0	0	0	0	0
0	0	0	0	0	0	0
0	0	0	0	0	0	0
0	0	0	0	0	0	0
0	0	0	0	0	0	0
0	0	0	0	0	0	0
0	0	0	0	0	0	0
0	0	0	0	0	0	0
0	0	0	0	0	0	0
0	0	0	0	0	0	0
0	0	0	0	0	0	0
0	0	0	0	0	0	0
0	0	0	0	0	0	0
0	0	0	0	0	0	0
0	0	0	0	0	0	0
0	0	0	0	0	0	0
0	0	0	0	0	0	0
0	0	0	0	0	0	0
0	0	0	0	0	0	0
0	0	0	0	0	0	0
0	0	0	0	0	0	0
0	0	0	0	0	0	0
0	0	0	0	0	0	0
0	0	0	0	0	0	0
0	0	0	0	0	0	0
0	0	0	0	0	0	0
0	0	0	0	0	0	0
0	0	0	0	0	0	0
0	0	0	0	0	0	0
0	0	0	0	0	0	0
0	0	0	0	0	0	0
0	0	0	0	0	0	0
0	0	0	0	0	0	0

0	0	0	0	0	0	0
0	0	0	0	0	0	0
0	0	0	0	0	0	0
0	0	0	0	0	0	0
0	0	0	0	0	0	0
0	0	0	0	0	0	0
0	0	0	0	0	0	0
0	0	0	0	0	0	0
0	0	0	0	0	0	0
0	0	0	0	0	0	0
0	0	0	0	0	0	0
0	0	0	0	0	0	0
0	0	0	0	0	0	0
0	0	0	0	0	0	0
0	0	0	0	0	0	0
0	0	0	0	0	0	0
0	0	0	0	0	0	0
0	0	0	0	0	0	0
0	0	0	0	0	0	0
0	0	0	0	0	0	0
0	0	0	0	0	0	0
0	0	0	0	0	0	0
0	0	0	0	0	0	0
0	0	0	0	0	0	0
0	0	0	0	0	0	0
0	0	0	0	0	0	0
0	0	0	0	0	0	0
0	0	0	0	0	0	0
0	0	0	0	0	0	0
0	0	0	0	0	0	0
0	0	0	0	0	0	0
0	0	0	0	0	0	0
0	0	0	0	0	0	0
0	0	0	0	0	0	0
0	0	0	0	0	0	0
0	0	0	0	0	0	0
0	0	0	0	0	0	0
0	0	0	0	0	0	0
0	0	0	0	0	0	0
0	0	0	0	0	0	0
0	0	0	0	0	0	0
0	0	0	0	0	0	0
0	0	0	0	0	0	0
0	0	0	0	0	0	0
0	0	0	0	0	0	0
0	0	0	0	0	0	0

0	0	0	0	0	0	0
0	0	0	0	0	0	0
0	0	0	0	0	0	0
0	0	0	0	0	0	0
0	0	0	0	0	0	0
0	0	0	0	0	0	0
0	0	0	0	0	0	0
0	0	0	0	0	0	0
0	0	0	0	0	0	0
0	0	0	0	0	0	0
0	0	0	0	0	0	0
0	0	0	0	0	0	0
0	0	0	0	0	0	0
0	0	0	0	0	0	0
0	0	0	0	0	0	0
0	0	0	0	0	0	0
0	0	0	0	0	0	0
0	0	0	0	0	0	0
0	0	0	0	0	0	0
0	0	0	0	0	0	0
0	0	0	0	0	0	0
0	0	0	0	0	0	0
0	0	0	0	0	0	0
0	0	0	0	0	0	0
0	0	0	0	0	0	0
0	0	0	0	0	0	0
0	0	0	0	13	0	0
0	0	0	0	0	0	0
0	0	0	0	0	0	0
0	0	0	0	0	0	0
0	0	0	0	0	0	0
0	0	0	0	0	0	0
0	0	0	0	0	0	0
0	0	0	0	0	0	0
0	0	0	0	0	0	0
0	0	0	0	0	0	0
0	0	0	0	0	0	0
0	0	0	0	0	0	0
0	0	0	0	0	0	0
0	0	0	0	0	0	0
0	0	0	0	0	0	0
0	0	0	0	0	0	0
0	0	0	0	0	0	0
0	0	0	0	0	0	0
0	0	0	0	0	0	0
0	0	0	0	0	0	0

0	0	0	0	0	0	0
0	0	0	0	0	0	0
0	0	0	0	0	0	0
0	0	0	0	0	0	0
0	0	0	0	0	0	0
0	0	0	0	0	0	0
0	0	0	0	0	0	0
0	0	0	0	0	0	0
0	0	0	0	0	0	0
0	0	0	0	0	0	0
0	0	0	0	0	0	0
0	0	0	0	0	0	0
0	0	0	0	0	0	0
0	0	0	0	0	0	0
0	0	0	0	0	0	0
0	0	0	0	0	0	0
0	0	0	0	0	0	0
0	0	0	0	0	0	0
0	0	0	0	0	0	0
0	0	0	0	0	0	0
0	0	0	0	0	0	0
0	0	0	0	0	0	0
0	0	0	0	0	0	0
0	0	0	0	0	0	0
0	0	0	0	0	0	0
0	0	0	0	0	0	0
0	0	0	0	0	0	0
0	0	0	0	0	0	0
0	0	0	0	0	0	0
0	0	0	0	0	0	0
0	0	0	0	0	0	0
0	0	41	0	0	0	0
0	0	0	0	0	0	0
0	0	0	0	0	0	0
0	0	0	0	0	0	0
0	0	0	0	0	0	0
0	0	0	0	0	0	0
0	0	0	0	0	0	0
0	0	0	0	0	0	0
0	0	0	0	0	0	0
0	0	0	0	0	0	0
0	0	0	0	0	0	0
0	0	0	0	0	0	0
0	0	0	0	0	0	0
0	0	0	0	0	0	0
0	0	0	0	0	0	0
0	0	0	0	0	0	0

0	0	0	0	0	0	0
0	0	0	0	0	0	0
0	0	0	0	0	0	0
0	0	0	0	0	0	0
0	0	0	0	0	0	0
0	0	0	0	0	0	0
0	0	0	0	0	0	0
0	0	0	0	0	0	0
0	0	0	0	0	0	0
0	0	0	0	0	0	0
0	0	0	0	0	0	0
0	0	0	0	0	0	0
0	0	0	0	0	0	0
0	0	0	0	0	0	0
0	0	0	0	0	0	0
0	0	0	0	0	0	0
0	0	0	0	0	0	0
0	0	0	0	0	0	0
0	0	0	0	0	0	0
0	0	0	0	0	0	0
0	0	0	0	0	0	0
0	0	0	0	0	0	0
0	0	0	0	0	0	0
0	0	0	0	0	0	0
0	0	0	0	0	0	0
0	0	0	0	0	0	0
0	0	0	0	0	0	0
0	0	0	0	0	0	0
0	0	0	0	0	0	0
0	0	0	0	0	0	0
0	0	0	0	0	0	0
0	0	0	0	0	0	0
0	0	0	0	0	0	0
0	0	0	0	0	0	0
0	0	0	0	0	0	0
0	0	0	0	0	0	0
0	0	0	0	0	0	0
0	0	0	0	31	0	0
0	0	0	0	0	0	0
0	0	0	0	0	0	0
0	0	0	0	0	0	0
0	0	0	0	0	0	0
0	0	0	0	0	0	0
0	0	0	0	0	0	0
0	0	0	0	0	0	0
0	0	0	0	0	0	0

0	0	0	0	0	0	0
0	0	0	0	0	0	0
0	0	0	0	0	0	0
0	0	0	0	0	0	0
0	0	0	0	0	0	0
0	0	0	0	0	0	0
0	0	0	0	0	0	0
0	0	0	0	0	0	0
0	0	0	0	0	0	0
0	0	0	0	0	0	0
0	0	0	0	0	0	0
0	0	0	0	0	0	0
0	0	0	0	0	0	0
0	0	0	0	0	0	0
0	0	0	0	0	0	0
0	0	0	0	0	0	0
0	0	0	0	0	0	0
0	0	0	0	0	0	0
0	0	0	0	0	0	0
0	0	0	0	0	0	0
0	0	0	0	0	0	0
0	0	0	0	0	0	0
0	0	0	0	0	0	0
0	0	0	0	0	0	0
0	0	0	0	0	0	0
0	0	0	0	0	0	0
0	0	0	0	0	0	0
0	0	0	0	0	0	0
0	0	0	0	0	0	0
0	0	0	0	0	0	0
0	0	0	0	0	0	0
0	0	0	0	0	0	0
0	0	0	0	0	0	0
0	0	0	0	0	0	0
0	0	0	0	0	0	0
0	0	0	0	0	0	0
0	0	0	0	0	0	0
0	0	0	0	0	0	0
0	0	0	0	0	0	0
0	0	0	0	0	0	0
0	0	0	0	0	0	0
0	0	0	0	0	0	0
0	0	0	0	0	0	0
0	0	0	0	0	0	0
0	0	0	0	0	0	0
0	0	0	0	0	0	0

0	0	0	0	0	0	0
0	0	0	0	0	0	0
0	0	0	0	0	0	0
0	0	0	0	0	0	0
0	0	0	0	0	0	0
0	0	0	0	0	0	0
0	0	0	0	0	0	0
0	0	0	0	0	0	0
0	0	0	0	10	0	0
0	0	0	0	20	0	0
0	0	0	0	21	0	0
0	0	0	0	17	0	0
0	0	0	0	431	0	0
0	0	0	0	119	0	0
0	0	0	0	25	0	0
0	0	0	0	293	0	0
0	0	0	0	26	0	0
0	0	0	0	11	0	0
0	0	0	0	11	0	0
0	0	0	0	11	0	0
0	0	0	0	0	0	0
0	0	0	0	0	0	0
0	0	0	0	0	0	0

Umbilical Cord						
UC_283	UC_288	UC_302	UC_307	UC_324	UC_329	UC_332
0	25	10	0	0	14	0
120	0	0	0	0	0	0
324	168	0	108	0	83	0
289	191	20	58	0	81	0
64	145	0	32	0	26	0
11	317	20	0	0	0	0
42	103	0	11	0	0	0
148	0	0	0	0	27	0
116	12	34	64	0	0	0
202	84	22	413	0	177	34
211	0	0	68	0	21	22
53	0	0	0	0	0	0
50	0	0	38	0	12	0
41	0	0	11	0	39	36
81	0	0	80	0	33	53
24	0	0	84	0	0	0
313	156	546	1474	885	1372	1069
0	0	0	481	39	204	90
33	0	40	16	14	11	14
10	48	0	131	15	206	36
0	0	0	16	12	87	60
12	0	54	125	13	85	0
41	81	0	69	0	37	0
0	0	0	0	0	0	0
463	482	371	1903	2741	3072	1008
54	72	253	1061	1019	993	312
41	10	38	601	840	776	126
48	18	41	166	292	771	122
95	54	127	431	899	2125	533
29	0	12	50	0	78	0
17	46	18	215	42	60	10
0	0	0	85	234	70	39
564	139	1444	2942	6867	1229	949
10	15	0	0	0	0	0
0	0	0	0	0	167	45
0	0	0	11	0	83	69
13	0	0	0	0	58	62
139	137	136	768	306	325	170
14	22	0	2401	100	373	156
4876	442	1186	11201	5332	4964	2093
102	0	142	231	795	58	56

113	0	128	161	942	48	31
21	0	53	499	433	836	491
49	0	107	444	340	872	498
1631	280	203	9793	540	3685	1067
0	94	0	862	214	2268	1442
759	0	12	123	0	13	0
169	0	0	323	0	170	85
1754	0	0	209	0	23	0
98	34	0	160	0	30	0
631	0	0	22	0	12	0
0	0	0	0	0	0	0
153	0	0	211	0	44	0
502	46	0	635	0	103	13
302	0	0	34	0	0	0
268	0	0	30	0	0	0
1646	0	10	35	0	43	0
40	0	0	138	0	0	0
1362	233	447	1498	459	1232	572
174	14	0	910	89	355	155
97	0	41	28	50	22	0
129	0	0	214	10	58	28
0	0	0	0	0	0	0
59	0	0	0	0	0	0
0	53	0	78	14	0	0
30	0	0	18	0	13	0
76	26	0	160	0	15	0
104	0	0	107	0	12	0
31	0	0	0	0	0	0
45	0	0	0	0	0	0
0	23	0	44	0	10	0
2864	0	56	1380	28	235	29
3231	1106	395	3550	360	1094	447
0	63	0	35	0	27	0
180	0	0	161	0	25	0
420	0	0	23	0	0	0
443	0	0	141	0	53	0
177	0	0	339	0	75	0
284	0	52	254	0	92	21
33	0	10	43	0	0	0
129	0	10	120	0	0	0
1177	0	21	1219	10	163	32
59	179	26	50	0	0	0
71	0	0	54	0	55	0
57	0	0	41	0	24	0
1683	23	105	1016	0	139	0
5098	342	300	5210	121	997	203

156	0	10	248	0	132	156
80	698	74	608	39	189	225
194	0	0	924	0	0	0
255	0	0	193	0	18	0
3293	214	132	1795	57	548	54
0	281	14	0	0	12	0
60	0	0	0	0	0	0
161	13	10	0	0	0	0
63	0	0	0	0	0	0
0	13	48	10	0	13	12
10	169	24	90	28	99	50
70	12	53	76	0	13	35
1118	2564	1649	4846	984	2480	1463
0	0	0	0	12	0	0
651	627	230	600	194	245	109
134	0	0	303	0	48	0
0	0	0	48	21	56	24
712	0	30	107	0	65	0
0	13	0	10	0	0	0
136	0	0	144	0	26	0
25	0	0	0	0	0	0
0	0	0	38	0	0	0
0	25	0	42	0	37	0
48	11	127	117	73	75	62
99	23	302	490	82	155	327
48	41	214	232	68	118	102
25	12	56	100	35	122	55
0	0	0	93	24	120	51
383	0	0	152	36	185	88
808	324	321	829	136	983	376
0	11	0	22	0	93	0
0	0	22	0	0	0	0
0	0	0	48	11	155	0
0	77	0	89	45	107	74
86	0	0	10	0	0	0
10	0	0	0	0	0	0
35	0	39	39	0	27	56
74	43	132	62	53	63	59
135	0	0	74	0	24	0
107	150	551	475	154	254	194
0	0	37	38	11	13	0
0	34	20	22	0	0	11
453	0	21	263	21	101	22
930	1477	542	8533	2509	7389	6576
10	0	12	339	177	531	437
118	12	723	3443	2013	3997	4572

11	31	0	28	0	14	62
0	59	0	213	51	129	40
14	133	152	562	113	182	165
1747	248	477	5416	739	536	460
110	0	28	296	0	12	0
78	90	45	744	44	73	52
0	0	0	0	0	0	0
68	0	35	267	0	21	0
67	0	0	51	0	15	0
0	0	0	0	0	0	0
98	52	45	229	27	37	17
0	110	26	33	0	187	134
171	0	20	86	0	0	0
0	0	41	133	0	44	55
23	48	36	324	114	197	150
94	0	0	57	0	0	0
45	0	0	121	0	10	0
62	0	0	0	0	0	0
0	0	0	0	0	0	0
0	84	502	0	508	203	28
140	81	162	164	78	53	29
26	68	209	26	0	0	0
103	182	32	99	37	74	70
0	0	0	10	0	0	0
43	0	35	20	0	0	0
1815	124	23	149	13	139	11
41	344	40	351	53	86	56
124	378	96	560	151	337	113
0	39	0	66	0	74	24
81	0	0	149	0	15	11
0	120	0	48	0	0	0
100	36	0	160	57	90	66
381	423	78	214	44	48	23
0	35	0	13	0	0	0
161	71	47	214	51	68	50
1805	0	52	209	185	555	43
1256	55	42	161	0	207	0
59	0	0	0	0	0	0
86	0	0	72	0	0	0
28	0	0	0	0	0	0
230	0	448	79	80	0	0
292	0	0	0	0	0	0
567	0	12	22	0	22	0
73	0	0	11	0	0	0
296	1360	976	0	0	46	0
0	709	130	59	11	21	13

20	43	315	39	0	11	0
42	534	920	118	27	73	183
27	2676	2358	1289	175	602	931
10	9046	3284	331	64	51	366
56	0	0	43	0	22	0
355	192	68	178	35	197	94
346	505	175	265	21	196	77
0	0	0	0	0	0	0
58	0	0	46	0	32	0
48	0	0	0	0	0	0
148	0	0	50	0	0	0
59	0	0	0	0	0	0
247	0	0	61	0	104	25
157	1240	271	1457	207	1131	413
12	129	12	11	0	119	30
1765	60	36	135	0	281	43
0	31	0	0	0	0	0
0	0	0	10	0	13	0
30	0	0	0	0	0	0
73	0	0	0	0	0	0
477	0	0	0	0	0	0
1274	148	971	854	7108	3546	995
586	0	0	32	0	12	0
0	0	0	0	0	0	0
637	0	0	13	0	58	0
100	12	0	85	0	93	27
1112	0	131	122	81	194	0
117	0	0	0	0	0	0
1403	0	12	0	0	0	0
0	0	0	0	0	0	0
285	0	26	0	0	0	0
3593	0	0	0	0	0	0
328	0	0	0	0	0	0
69	0	0	0	0	0	0
25	0	0	0	0	0	0
25	0	37	49	83	87	0
9123	610	2552	3891	1643	7812	1272
7187	191	3417	1378	1119	3427	962
848	0	64	194	0	161	40
116	0	0	0	0	0	0
34	0	0	0	0	0	0
6920	124	158	1241	99	460	57
1592	73	170	194	83	82	15
43	0	39	24	14	16	0
1563	0	0	34	0	51	0
4235	31	65	410	11	470	11

1703	0	21	339	10	218	0
4635	11	28	834	15	223	0
518	0	191	156	34	461	87
15	11	16	0	0	39	10
89	36	113	58	24	57	34
3452	185	1586	1431	1402	3571	926
2294	58	110	296	46	230	30
23	0	233	123	17	72	47
0	0	0	66	22	0	10
0	0	0	0	0	0	0
981	0	0	34	0	50	0
0	0	0	26	0	0	0
77	0	0	19	0	0	0
244	12	333	379	98	717	355
0	0	0	151	31	11	42
432	0	0	0	0	0	0
401	0	0	0	0	0	0
927	0	0	39	0	44	0
217	59	229	760	344	1350	487
112	0	0	62	0	127	36
0	0	0	0	0	10	0
25	0	14	0	0	29	28
328	17	335	57	105	127	54
25	0	43	0	0	89	39
1550	161	1288	727	1390	5224	872
973	27	84	182	10	256	35
36	0	0	24	0	0	0
3476	51	219	1068	10	480	44
40	89	345	177	153	160	89
10	87	171	109	47	58	40
1329	0	10	226	0	178	0
27	52	42	38	13	41	13
132	0	0	0	0	0	0
158	0	77	12	57	10	0
1208	50	409	1064	436	3327	869
346	0	173	156	150	189	88
437	0	95	152	41	331	177
21	0	69	0	0	0	0
107	0	0	0	0	0	0
689	39	125	152	38	70	22
1466	74	30	25	0	92	0
487	220	37	144	0	44	0
33	0	0	33	0	0	17
81	186	93	154	149	377	70
0	0	11	11	0	0	0
2481	113	862	4940	106	382	1040

580	273	1586	3546	453	1097	875
0	16	0	51	0	90	0
0	0	96	20	26	21	14
0	112	176	1649	793	1340	807
21	15	91	481	18	164	104
46	0	148	154	0	22	58
57	10	158	30	82	33	24
0	87	246	1374	79	114	387
0	0	22	58	0	0	0
0	0	12	13	0	0	0
61	0	0	0	0	0	0
0	10	258	389	89	398	296
1135	919	1868	6489	644	1734	1261
113	59	406	83	107	138	77
83	36	292	172	34	96	211
97	225	319	466	194	874	507
385	739	1050	1278	440	1215	1214
12	0	67	1516	0	225	117
0	0	24	487	0	102	57
41	13	86	1630	18	408	193
15	0	0	1747	0	147	41
33	0	15	274	307	236	56
69	29	0	1211	885	744	251
31	0	0	372	160	299	220
15	0	0	463	196	254	234
35	0	110	158	54	28	70
1268	518	3938	13645	11834	9155	6868
72	112	86	1305	1085	5648	1337
27	0	80	384	523	362	341
26	24	53	91	68	35	32
485	267	3321	4948	1775	924	1687
41	83	33	805	1322	2494	319
129	0	60	1910	3007	1540	1572
59	0	0	0	0	0	0
81	36	58	243	501	477	110
126	15	260	377	2483	688	375
0	0	44	58	190	112	12
119	103	456	1157	3370	1875	1390
135	85	192	702	1197	989	395
50	48	72	678	1311	743	235
11	104	56	487	321	655	174
10	22	106	1073	883	926	312
20	0	0	86	0	87	0
102	43	1021	266	373	501	579
216	201	92	234	53	174	45
74	0	51	60	14	53	0

2498	121	704	199	160	311	57
52	228	49	243	72	232	57
45	73	135	84	90	77	0
33	369	19	149	66	396	56
0	26	0	10	11	0	0
182	0	0	0	0	0	0
18	15	36	79	0	176	34
14	74	0	41	26	64	0
0	11	0	10	109	103	0
20	16	0	135	49	102	50
72	0	0	0	10	0	0
408	547	222	382	686	416	64
0	37	0	0	0	149	0
0	40	0	82	17	165	22
105	0	0	0	0	0	0
100	0	0	0	0	0	0
0	35	0	0	0	26	0
471	36	0	0	0	36	0
1683	249	226	1427	386	511	228
528	0	0	0	0	0	0
37	0	0	0	0	0	0
420	0	0	0	0	0	0
0	0	0	0	0	0	0
2294	29	239	1200	366	397	130
0	0	0	0	0	0	0
110	0	0	27	0	18	0
187	0	45	394	77	67	0
73	10	34	393	90	43	15
84	0	0	0	0	0	0
92	0	0	0	0	0	0
55	0	0	0	0	0	0
52	0	0	0	0	0	0
36	0	0	0	0	0	0
91	0	0	0	0	12	0
537	0	52	744	116	582	123
106	0	0	0	0	0	0
0	0	0	0	0	0	0
0	0	0	0	0	0	0
36	0	0	0	0	0	0
15	12	0	0	0	0	0
0	0	0	10	0	0	0
10	0	0	10	10	0	0
15	28	16	39	16	20	11
32	0	0	40	0	40	23
30	0	0	0	0	0	0
0	0	0	0	0	0	0

255	10	45	63	0	264	45
54	0	0	0	0	0	0
12	0	0	0	0	0	0
360	1271	492	1118	347	466	511
471	0	0	11	0	39	0
266	0	0	10	0	49	0
276	0	10	24	0	258	0
322	0	12	60	0	104	0
11	39	0	0	12	156	16
164	0	76	61	54	0	0
216	10	28	192	68	585	137
382	359	53	387	71	623	99
2362	11	75	184	0	1269	97
1074	279	133	292	81	644	107
367	0	35	72	0	231	61
13	0	0	0	0	0	0
29	0	0	0	0	0	0
346	0	0	0	0	90	0
0	0	0	0	0	0	0
62	0	10	0	0	0	0
216	10	41	50	26	51	23
34	0	0	0	0	0	0
378	0	28	60	11	99	0
2627	0	52	126	32	450	0
83	0	0	0	0	0	0
91	0	0	0	0	0	0
10	0	14	49	0	38	0
0	0	0	0	0	0	0
129	220	217	598	181	287	120
0	0	0	0	0	0	0
0	0	25	26	11	24	10
34	0	12	75	48	75	14
23	0	11	37	16	12	20
46	0	0	0	0	0	0
24	0	0	0	0	0	0
0	0	0	0	0	0	0
0	0	0	0	0	0	0
617	223	142	442	138	877	187
25	0	0	0	0	0	0
147	0	0	0	0	159	34
10	0	21	94	0	530	90
82	0	0	31	0	48	0
73	0	0	0	0	0	0
81	0	0	0	12	24	0
34	0	0	0	22	0	0
95	0	0	0	0	10	0

64	0	76	91	63	123	14
0	0	0	80	0	0	0
0	0	0	0	0	0	0
0	0	0	0	0	38	0
153	0	0	0	0	0	0
210	0	0	0	0	26	0
69	0	0	14	0	87	0
65	0	43	81	13	0	0
1601	906	870	1617	514	1522	529
10	112	31	66	49	132	54
79	0	38	96	27	11	0
225	329	357	512	222	522	102
31	0	0	0	0	0	0
15	0	0	0	0	0	0
0	0	14	14	13	0	0
368	173	1160	992	434	543	616
62	0	59	24	97	32	0
39	0	0	0	18	0	0
88	0	0	0	0	28	0
53	0	0	0	0	0	0
12	0	0	0	0	0	0
0	0	0	10	0	13	0
32	11	0	26	0	10	0
201	45	115	113	30	33	13
2839	56	157	839	205	2450	235
1359	254	705	1071	437	1625	477
96	63	30	141	24	225	35
185	0	96	61	75	66	0
304	28	139	428	249	526	248
0	0	0	32	0	0	0
10	0	0	0	0	0	0
16	0	0	0	0	0	0
29	0	93	49	46	91	43
0	13	41	71	11	40	0
85	87	137	178	99	107	34
0	24	0	26	0	122	0
2266	0	0	496	0	246	14
19538	821	794	9618	984	8499	996
476	28	10	377	233	2461	271
53	0	0	10	0	0	0
9943	15	60	909	0	483	53
20	0	0	78	0	0	18
61	24	0	22	0	12	0
763	0	14	61	0	56	0
12	0	10	0	0	0	0
1237	19	48	65	0	426	114

1439	0	0	24	0	24	0
542	0	0	76	0	31	0
17	0	0	0	0	0	0
5031	49	218	367	54	362	89
0	26	0	12	14	0	0
181	0	115	89	51	258	23
109	19	76	50	48	165	56
166	11	46	202	44	288	48
20	60	58	64	0	29	0
75	0	13	90	0	42	14
779	73	306	1437	376	1417	448
515	0	0	116	0	37	0
88	0	0	0	0	10	0
32	140	31	24	10	26	0
23	56	0	0	0	33	0
0	0	0	0	0	0	0
0	0	0	0	0	0	0
0	0	0	0	0	0	0
73	203	133	174	31	88	50
134	0	0	0	0	0	0
1145	13	94	0	31	140	23
0	0	0	0	0	0	0
2151	0	16	57	0	103	0
3410	0	0	23	0	27	0
3053	0	35	367	0	257	0
770	0	27	0	0	0	0
33	0	0	0	0	11	0
1245	13	64	0	0	25	0
548	0	0	17	0	44	11
417	0	0	0	0	0	0
968	0	0	11	0	0	0
160	0	0	0	0	0	0
443	0	0	0	0	0	0
0	0	26	13	10	15	0
0	0	0	0	0	0	0
342	0	0	0	0	0	0
708	47	0	55	0	42	0
993	0	183	305	74	520	139
6418	5456	2475	3841	1497	2971	1510
1699	0	23	0	0	0	0
14	0	0	0	0	0	0
322	0	0	0	0	0	0
537	0	44	191	78	465	129
75	0	0	34	16	67	33
175	0	0	0	0	0	0
17	0	0	0	0	0	0

106	0	0	0	0	0	0
379	10	88	118	61	85	12
217	0	0	0	0	33	26
54	0	0	0	0	0	0
37	0	0	0	0	0	0
108	18	0	35	12	10	0
61	0	0	0	0	22	0
0	0	0	0	0	28	0
0	0	0	0	0	0	0
53	0	0	40	0	45	0
71	0	0	0	0	0	0
985	0	24	78	30	69	0
2063	508	5109	3471	1779	1820	1406
211	50	547	458	164	207	134
1953	0	0	83	0	40	0
104	0	0	0	0	0	0
128	0	0	0	0	0	0
25	13	41	36	0	0	10
25	0	75	28	97	64	25
353	102	954	877	381	474	282
265	43	471	444	149	211	135
48	52	40	99	0	95	24
321	36	0	331	33	285	95
129	0	0	0	0	0	0
0	0	36	0	0	0	0
43	0	0	0	0	0	0
387	0	0	0	0	12	0
234	259	348	390	92	212	66
170	0	11	112	0	158	11
285	0	12	21	0	0	0
329	0	0	0	0	12	0
0	0	0	0	0	0	0
175	203	177	458	181	2285	114
0	35	0	0	10	0	0
0	25	0	12	21	11	0
17	0	0	0	0	24	0
18	30	110	177	117	1446	36
10	0	69	67	44	323	0
117	56	119	175	38	1330	289
88	71	215	357	225	2763	98
16	10	21	0	0	23	0
38	0	0	0	0	0	0
82	0	0	0	0	43	0
2728	94	1094	1158	527	1824	535
10	0	0	0	0	0	0
97	0	0	0	0	10	0

0	0	0	0	0	0	0
174	0	0	0	0	0	0
36	0	0	0	0	0	0
45	42	81	0	0	10	0
208	239	194	164	103	251	114
577	25	487	613	368	928	378
326	85	347	140	114	132	74
399	0	15	10	11	76	0
117	37	104	423	238	655	215
83	0	0	0	0	0	0
126	0	14	0	0	0	0
258	0	14	58	11	105	0
106	0	0	0	0	32	0
0	0	0	0	0	0	0
39	0	0	0	0	0	0
16	0	0	0	0	0	0
0	0	0	0	0	0	0
235	0	21	39	0	49	0
74	0	0	0	0	35	0
0	0	0	0	21	16	0
322	0	175	62	0	11	81
227	0	0	0	0	0	0
747	158	1536	700	465	610	653
0	0	0	0	0	0	11
424	445	617	715	264	383	230
145	0	0	0	0	0	0
199	0	125	130	50	73	129
14	243	158	257	52	162	61
63	0	0	0	0	0	0
24	0	0	0	0	0	0
11	0	0	0	0	0	0
1081	516	48	76	337	614	102
623	140	71	0	241	364	67
55	0	0	0	0	22	0
813	12	0	35	0	127	0
310	0	10	0	0	0	0
237	11	16	21	0	122	0
142	0	0	0	0	0	0
255	0	0	0	0	0	0
193	0	37	0	0	104	0
0	0	168	12	0	35	0
151	175	26	187	1959	1184	412
1104	202	35	240	38	1335	187
390	52	40	14	0	0	450
11	76	18	0	0	494	15
200	0	0	0	0	30	0

0	0	0	27	17	23	0
126	273	301	321	97	953	224
0	21	115	0	0	275	10
307	10	13	87	0	158	0
134	0	103	0	12	82	25
0	55	22	0	0	58	0
53	0	0	49	0	141	0
49	55	0	94	0	44	0
1178	595	1954	1690	1121	5839	1254
720	104	928	768	285	940	1178
222	26	568	268	81	306	344
23	10	13	44	11	95	0
0	0	0	0	0	0	0
18	0	0	0	0	282	0
48	0	0	0	0	0	0
81	139	19	110	40	849	234
0	10	0	14	0	14	12
57	0	0	11	0	0	0
0	0	0	0	0	82	0
0	0	0	0	0	0	0
518	32	92	259	18	556	71
190	78	133	255	29	1893	86
0	0	0	145	0	0	0
89	0	0	0	0	0	0
0	0	0	0	0	0	0
1294	88	248	195	105	140	71
11331	1630	3945	3588	2231	2894	2089
426	473	1723	2237	1865	5252	2011
9200	3820	11051	27022	14164	50809	21998
53	0	400	77	341	852	70
109	0	464	157	586	822	147
63	25	203	55	0	85	15
1459	550	2822	6147	2155	5491	1865
398	456	1283	1414	2670	2230	972
132	0	434	23	123	80	21
181	0	0	633	0	199	0
1564	361	385	1425	642	5508	1664
991	542	437	1642	195	3107	592
843	125	338	1429	360	2295	697
10866	180	383	1846	103	1729	278
237	28	0	29	0	12	0
190	0	0	45	0	15	0
3300	418	552	1643	702	6940	2547
110	0	52	59	0	735	103
328	0	0	0	0	0	0
1462	564	455	693	285	2371	745

305	119	102	349	120	1911	299
1769	946	1682	2742	1870	6195	3590
26	28	11	0	16	11	11
294	134	526	508	243	284	196
31	73	48	90	76	1076	283
0	0	10	0	0	33	38
218	269	444	300	438	5954	442
37	0	26	32	58	123	129
49	0	0	0	0	0	0
38	15	0	26	67	193	34
23	65	43	148	621	1618	257
106	48	62	53	12	475	106
37	0	0	0	0	0	0
90	0	0	0	0	0	0
1134	40	73	103	10	902	0
2790	102	455	616	382	2272	399
496	88	0	229	138	1702	343
943	12	70	167	55	694	11
317	0	10	191	0	126	28
36	0	0	14	0	363	116
579	32	60	150	180	1343	417
241	0	0	0	0	0	0
54	0	0	0	0	0	0
779	14	0	12	0	0	0
910	50	320	111	57	474	63
181	0	0	0	0	0	0
0	0	0	0	0	0	0
159	0	0	0	0	0	0
164	0	0	11	0	11	0
199	19	0	136	0	69	0
284	0	16	0	0	17	0
52	0	0	12	0	0	0
315	36	161	105	142	228	0
1108	207	556	282	147	189	99
40	12	40	40	16	539	0
0	42	34	42	0	47	20
117	0	136	665	127	1198	224
0	0	0	229	46	195	89
996	161	285	764	287	963	246
128	0	12	14	0	13	0
235	29	10	22	0	18	0
5682	43	51	36	0	61	0
400	0	0	0	0	0	0
8437	14	101	51	0	75	0
86	0	0	0	0	0	0
11807	0	81	12	0	79	0

135	0	0	0	0	0	0
312	0	0	10	13	307	13
878	36	13	0	0	32	0
168	0	0	0	0	10	0
10	54	0	10	0	70	0
104	0	0	0	0	0	0
15	0	0	0	0	0	0
0	0	0	0	0	0	0
0	0	0	0	0	0	0
3436	25	21	10	0	205	0
0	12	0	0	0	0	0
0	48	29	82	0	0	10
0	0	55	552	195	104	57
0	0	10	70	0	128	113
0	0	118	413	115	1084	938
0	18	22	221	158	21	0
0	0	27	21	0	84	41
0	0	64	430	265	57	23
11	0	27	0	11	67	77
0	0	10	0	20	13	18
221	10	158	211	2416	760	66
10	0	0	120	146	148	10
0	0	0	12	0	53	0
0	0	0	0	0	0	0
24	0	30	84	11	125	10
0	0	10	18	0	0	0
0	0	0	0	0	0	0
0	15	291	266	10	0	11
0	0	0	80	427	0	0
0	0	0	15	0	0	0
0	0	0	0	0	0	12
0	0	0	0	0	0	0
0	0	0	17	10	12	0
0	0	0	0	0	34	32
0	36	0	72	0	0	0
0	0	0	0	0	0	0
0	0	0	0	0	0	0
0	0	0	0	0	0	0
0	0	0	0	0	26	11
0	0	12	27	13	11	0
0	0	0	0	0	35	0
0	0	31	12	0	20	0
0	0	0	0	0	0	0
51	0	25	25	0	34	32
0	22	75	90	0	19	24
0	10	0	0	0	0	0

0	0	29	0	0	0	0
14	0	0	0	0	0	0
0	0	0	0	0	0	0
10	0	0	57	10	185	0
10	0	0	12	0	0	0
0	0	0	0	0	0	0
0	23	0	75	0	0	17
0	0	11	28	0	0	0
0	0	0	0	0	0	0
0	0	13	77	64	89	96
0	0	0	13	0	34	13
0	0	0	0	0	0	0
0	0	0	92	10	178	50
0	0	0	0	0	0	0
0	0	0	10	10	0	0
0	0	0	0	0	0	0
0	30	0	50	0	0	10
0	0	0	0	0	0	0
0	0	46	634	304	863	813
16	0	61	21	0	25	25
0	0	0	113	0	16	0
30	0	0	17	0	0	0
0	0	26	0	0	0	0
0	0	0	66	0	63	0
0	0	0	0	0	0	0
0	0	0	29	0	0	0
0	0	23	48	47	67	10
0	0	0	10	21	28	0
0	0	0	0	0	0	0
0	0	0	0	0	0	0
44	0	14	24	0	0	0
0	0	0	0	0	0	0
0	18	0	12	0	0	0
51	99	26	45	0	23	0
0	0	38	0	0	0	0
0	69	69	43	0	0	0
0	0	0	0	0	0	0
0	383	249	0	0	0	0
0	0	0	10	0	0	0
0	0	0	33	0	0	0
0	419	10	85	0	1728	253
0	203	0	0	0	51	33
0	28	60	35	0	585	110
0	0	0	0	14	56	0
0	0	35	91	0	11	0
0	0	0	0	0	0	0

0	10	41	75	15	0	0
0	50	0	0	0	0	0
0	0	80	498	0	0	0
0	76	20	0	0	0	0
0	92	164	750	24	56	95
10	233	525	516	119	731	892
11	0	0	0	0	0	0
0	0	32	148	95	1123	209
0	0	27	56	0	0	0
0	14	0	38	70	0	0
0	0	0	0	0	0	0
0	0	0	0	0	0	0
263	0	454	611	411	500	166
0	0	0	0	0	0	0
0	0	0	0	0	0	0
84	0	79	0	157	117	43
0	0	0	0	0	0	0
0	21	0	37	0	0	0
444	25	469	62	575	969	69
0	0	0	0	0	0	0
0	0	0	0	0	0	0
0	0	0	0	0	0	0
0	0	0	0	0	0	0
0	0	216	38	42	36	0
0	0	56	48	11	0	0
35	0	168	60	154	420	122
0	0	0	0	0	0	0
0	0	58	0	0	0	0
79	0	180	74	73	142	59
0	0	0	0	0	0	0
0	0	46	0	0	0	0
570	0	121	11	0	0	0
0	0	0	0	0	0	0
41	0	303	154	394	1453	370
35	0	0	0	0	192	51
0	0	0	0	0	0	0
0	0	0	0	0	0	0
0	0	0	0	0	13	0
0	0	15	0	0	13	0
13	34	83	49	28	33	0
80	0	63	27	40	0	0
0	0	0	134	78	1269	192
25	24	153	133	41	48	0
10	0	31	0	0	0	0
0	0	0	0	0	0	0
13	0	10	63	64	272	0

0	10	11	80	0	274	11
0	0	0	0	0	0	0
0	0	0	0	0	0	0
0	0	27	10	0	0	24
0	103	79	224	0	33	82
127	0	34	0	0	0	0
0	0	64	304	0	0	70
22	0	81	29	0	29	0
0	0	0	0	0	0	0
0	0	0	0	0	0	0
11	30	296	408	27	10	127
0	0	43	19	0	0	0
146	23	394	186	26	86	58
0	0	13	0	0	0	0
36	13	88	149	29	39	14
0	10	10	11	10	10	52
0	0	165	130	13	12	19
0	0	0	0	0	34	0
0	28	75	1019	0	13	99
0	0	0	0	0	0	0
11	0	0	0	0	0	0
23	0	16	10	0	0	0
0	0	0	0	0	0	10
0	0	0	10	0	0	0
0	0	59	19	0	0	18
0	0	133	0	0	0	0
54	67	304	251	96	199	361
12	0	119	87	65	160	116
99	41	299	260	155	417	266
0	0	0	0	0	0	0
0	0	0	376	0	0	12
0	0	47	653	0	112	80
0	0	0	195	0	16	0
0	0	12	815	0	141	69
0	0	0	0	0	0	0
0	0	34	0	0	0	0
0	0	0	78	0	12	0
29	0	47	122	25	136	40
30	0	145	66	0	21	12
0	0	43	59	0	0	12
40	37	396	545	23	11	83
11	0	94	42	23	0	49
0	0	0	109	0	0	32
0	0	0	49	0	0	0
0	0	38	12	0	0	0
0	0	63	0	0	0	0

0	0	0	148	19	0	14
89	23	609	644	97	11	42
0	0	171	22	0	0	0
23	0	158	56	0	0	0
0	45	0	10	0	0	0
0	0	0	34	143	78	11
0	0	0	51	81	108	26
0	0	23	13	0	0	0
0	41	168	58	32	11	104
12	0	41	201	18	385	153
0	0	26	121	40	54	47
0	0	0	110	0	43	36
0	0	0	0	0	0	0
0	0	0	0	0	0	0
0	0	0	0	0	0	0
0	0	0	0	0	11	0
0	46	24	108	51	258	45
0	46	0	31	0	34	0
37	0	0	26	0	53	31
0	25	10	46	0	0	0
40	0	479	132	182	153	230
0	0	0	0	0	0	0
0	0	0	0	0	0	0
0	0	0	0	0	0	0
0	0	0	0	0	0	0
0	26	0	29	10	174	0
0	0	0	19	0	0	0
13	80	0	0	41	67	0
0	0	0	0	0	0	0
0	0	0	0	0	0	0
0	0	0	10	0	0	0
0	0	0	0	0	0	0
0	0	0	0	0	0	0
0	0	0	0	0	0	0
0	145	0	10	0	0	0
0	23	0	0	0	0	0
10	24	23	37	12	710	10
28	256	70	132	100	554	50
0	0	0	0	0	10	0
12	27	22	42	28	154	12
0	0	0	14	0	12	0
54	17	12	132	601	528	33
0	30	0	0	0	0	0
0	0	0	0	0	0	0
71	0	0	0	74	314	40
24	28	0	111	38	67	51

0	0	0	0	0	0	0
0	0	0	0	0	0	0
13	77	15	0	35	84	0
44	10	0	32	39	28	48
0	0	14	0	13	155	0
0	0	0	0	0	27	0
0	0	0	0	0	0	0
26	68	21	33	33	263	46
0	0	0	0	0	0	0
25	0	0	0	39	171	0
0	106	0	37	0	107	36
12	0	14	0	0	31	0
0	10	0	57	11	26	0
0	0	0	0	0	0	0
0	0	12	44	35	30	0
0	0	0	0	0	0	0
0	0	0	0	0	0	0
0	0	0	0	0	0	0
0	0	0	12	0	87	0
0	0	0	30	0	12	0
0	0	0	10	0	0	0
0	0	0	0	0	0	0
0	0	0	0	0	0	0
0	0	0	0	0	0	0
0	0	0	0	0	0	0
0	0	0	0	0	0	0
0	41	0	34	0	48	0
32	0	10	31	0	0	0
11	0	0	0	0	0	0
0	0	0	0	0	0	0
14	0	13	41	0	15	11
0	0	10	0	0	30	0
0	0	0	0	0	0	0
0	0	0	0	0	0	0
80	14	101	92	53	2559	122
0	10	0	47	0	704	54
0	0	0	0	0	162	0
0	0	0	0	0	20	0
39	47	98	42	90	846	41
0	0	0	0	0	0	0
0	0	0	0	0	11	0
0	0	0	12	0	15	0
0	0	0	0	0	27	0
0	0	0	0	0	12	0
0	10	63	146	43	168	45
0	0	0	12	0	0	0

0	15	13	70	0	49	0
0	0	0	0	0	0	0
0	0	0	0	0	0	0
0	0	0	31	11	0	0
0	0	0	11	0	0	0
0	0	55	39	10	10	0
0	0	0	10	0	0	0
0	16	0	0	0	0	0
11	0	0	0	0	0	0
0	0	0	0	0	0	0
0	0	0	0	0	0	0
0	0	0	0	0	0	0
0	0	68	17	0	0	0
0	0	0	13	0	0	0
0	0	0	0	0	0	0
0	0	0	0	0	0	0
12	0	0	11	0	0	79
0	0	0	0	56	0	0
0	0	0	0	0	0	0
0	0	0	0	0	0	0
0	0	0	0	0	0	0
0	0	0	0	0	11	0
0	0	37	0	418	0	0
31	0	50	21	0	83	0
92	0	0	40	30	138	0
0	36	81	48	101	242	0
45	0	0	0	0	30	0
0	0	11	0	34	123	25
10	0	0	0	0	41	0
0	0	0	0	0	0	0
0	0	0	0	0	0	0
0	0	0	0	0	0	0
0	0	0	0	0	0	0
0	0	0	0	0	0	0
0	0	0	0	0	0	0
0	0	0	0	0	0	0
0	0	0	0	0	0	0
0	0	0	0	0	0	0
0	0	0	0	0	0	0
0	0	0	0	0	0	0
165	22	210	207	220	740	462
34	13	82	0	0	36	0
0	0	0	33	0	23	0
27	0	16	23	0	0	0
0	13	0	0	0	10	0
0	0	0	0	0	0	0

0	0	15	0	0	0	0
0	0	0	0	0	0	0
0	0	0	0	0	23	80
0	0	0	0	0	0	0
0	0	0	0	0	0	21
0	0	0	0	21	0	52
0	0	0	0	0	0	0
0	10	0	26	0	0	0
43	0	20	46	0	64	28
0	0	0	0	0	0	28
0	0	0	10	0	164	0
0	0	0	0	0	0	0
0	0	0	42	0	42	0
0	0	0	0	0	0	0
1196	54	131	195	155	84	43
31	0	25	0	84	183	14
0	0	0	26	24	78	0
280	0	77	24	436	35	0
0	0	0	0	0	0	0
0	0	0	0	0	240	0
0	0	12	0	0	99	0
23	49	78	98	10	52	0
0	0	0	0	0	0	0
0	0	0	0	0	0	0
0	0	0	0	0	0	0
0	0	0	0	0	0	0
0	0	0	0	0	0	0
34	40	75	372	239	1514	420
0	0	19	0	32	0	0
0	0	27	0	0	0	0
121	17	271	223	177	72	160
64	0	189	0	0	47	14
116	28	196	223	47	69	112
42	0	12	0	0	0	0
78	0	0	0	0	0	0
0	0	0	0	0	0	0
0	0	0	0	0	0	0
167	0	12	67	21	55	32
0	0	0	0	0	654	55
0	0	0	0	0	0	0
0	0	0	12	0	191	43
0	0	16	17	0	13	33
0	0	0	24	0	0	0
0	0	0	0	0	0	29
0	0	0	0	0	40	0
0	0	0	0	0	0	0

0	0	0	0	0	0	0
0	0	0	0	0	0	0
0	0	0	0	0	0	0
0	0	0	0	0	0	0
0	11	0	0	11	95	0
10	0	0	0	0	0	0
0	0	0	30	0	167	0
0	0	0	0	0	0	10
0	0	13	29	17	558	116
0	0	0	16	76	239	36
11	0	0	0	0	51	0
16	40	36	12	0	556	27
0	0	0	0	0	0	0
0	0	0	0	0	39	0
0	28	45	42	0	293	0
0	0	35	64	81	15	0
0	0	0	0	0	0	0
0	0	0	0	0	43	0
0	0	12	167	165	290	161
0	0	0	26	161	0	0
20	0	0	177	0	23	0
0	0	10	11	0	56	0
0	0	0	0	0	51	13
0	0	0	19	0	0	0
0	0	0	0	0	0	0
0	0	13	31	0	0	0
0	0	0	0	0	0	0
0	0	0	0	0	0	0
0	13	0	0	0	0	10
0	64	0	0	0	0	0
0	0	0	0	0	0	0
0	0	0	10	0	0	0
0	0	102	265	112	0	0
0	17	12	126	0	0	44
0	0	0	0	0	0	0
15	56	25	30	0	46	0
0	24	0	11	0	37	10
0	12	0	52	0	11	0
0	0	0	0	0	0	0
0	0	0	0	0	0	0
14	0	31	0	0	0	0
0	0	11	0	0	0	0
0	10	0	10	0	0	0
0	0	0	0	0	0	0
43	0	0	12	0	36	0
0	0	0	0	0	34	0

13	0	0	0	0	42	0
10	0	0	0	22	35	0
26	0	0	0	0	43	0
0	21	27	25	44	2148	22
0	0	0	16	13	144	0
21	0	12	0	0	0	16
0	16	0	10	0	133	0
0	0	0	0	0	38	0
0	29	58	0	0	0	0
0	0	0	0	0	0	0
0	0	0	0	0	0	0
0	0	0	0	0	10	14
0	0	0	0	0	12	0
61	0	0	122	0	50	18
114	0	0	23	639	109	17
0	0	0	0	0	0	27
51	0	0	23	0	0	16
23	0	0	38	0	46	30
0	11	0	0	0	0	68
56	0	13	84	0	64	24
0	0	22	27	0	0	12
12	0	13	31	0	20	20
0	0	0	33	0	33	20
0	0	0	11	10	71	14
0	0	0	0	0	26	26
0	0	0	198	10	182	448
0	0	24	118	91	51	28
0	0	0	38	0	47	16
13	0	0	63	0	40	13
0	0	10	0	0	0	10
0	0	0	0	0	61	15
0	0	0	0	0	0	11
0	0	0	16	12	0	10
0	94	0	20	195	273	108
0	0	0	0	0	367	136
0	0	0	0	0	25	15
0	0	0	0	0	256	51
0	0	0	0	0	0	21
98	0	12	131	0	77	70
0	0	0	0	0	20	35
0	0	41	206	0	0	39
0	0	0	0	0	0	12
0	0	0	0	0	29	48
0	0	0	0	0	44	12
0	0	0	0	0	96	30
23	0	0	34	0	14	34

0	0	0	101	0	210	12
0	0	0	0	0	119	120
0	0	0	0	0	43	10
0	21	0	29	0	133	10
0	0	0	37	0	219	13
0	0	0	0	0	37	106
0	0	41	33	0	0	13
0	0	12	95	0	0	33
0	0	15	33	0	0	13
0	0	234	272	25	12	77
0	0	0	0	0	0	12
0	0	0	80	0	73	78
0	0	23	26	0	0	24
0	0	0	0	0	0	10
0	0	0	0	0	0	28
0	0	0	0	0	0	21
0	0	75	0	10	17	34
52	0	0	0	55	17	10
45	0	24	11	12	216	10
0	46	103	117	241	90	52
61	0	38	0	340	642	28
0	0	40	21	12	14	14
0	0	0	0	0	33	13
0	0	0	0	0	0	37
0	0	0	0	0	13	11
0	0	0	29	0	65	14
197	0	0	74	811	392	100
0	0	0	83	309	1711	472
0	0	0	0	0	0	11
0	0	0	76	22	544	246
0	0	0	0	0	54	11
0	0	0	0	0	0	10
0	10	0	10	22	46	13
0	0	0	0	0	179	53
163	0	0	37	194	435	265
49	0	99	75	77	109	38
0	0	0	0	0	29	10
0	0	0	0	0	0	0
0	0	0	0	0	0	0
0	0	0	0	0	0	0
0	0	0	0	0	0	0
0	0	0	0	0	0	0
0	0	0	0	0	0	0
0	0	0	0	0	0	0
0	0	0	0	0	0	0
17	0	13	0	0	0	0

0	0	0	0	0	0	0
0	0	0	0	0	0	0
0	0	0	0	0	0	0
0	0	0	0	0	0	0
0	0	0	0	0	0	0
0	0	0	0	0	0	0
0	0	0	0	0	0	0
0	0	0	0	0	0	0
0	0	0	0	0	0	0
0	0	0	0	0	0	0
0	0	0	0	0	0	0
0	1568	315	0	0	0	0
0	0	0	0	0	0	0
0	0	47	86	0	23	0
0	0	196	67	0	0	0
0	0	0	0	0	0	0
0	0	0	0	0	0	0
0	0	0	0	0	0	0
0	0	0	0	0	0	0
0	0	0	0	0	0	0
0	0	0	0	0	0	0
0	0	0	0	0	0	0
0	0	0	0	0	0	0
0	0	0	0	0	0	0
0	0	0	0	0	0	0
0	0	0	0	0	0	0
0	0	0	0	11	0	0
0	0	0	0	0	0	0
0	0	0	0	0	0	0
0	0	0	11	0	0	0
0	0	0	0	0	0	0
0	0	0	0	0	0	0
0	0	0	0	0	0	0
0	0	0	0	0	0	0
0	0	0	0	0	0	0
0	0	0	0	0	0	0
0	0	0	0	0	0	0
0	0	0	0	0	0	0
0	0	0	0	0	0	0
0	0	0	0	0	0	0
0	0	0	0	0	0	0
0	0	0	0	0	0	0
0	0	0	11	0	0	0
0	0	0	0	0	0	0
0	0	0	0	0	0	0
0	0	0	0	0	0	0

0	0	0	0	0	0	0
0	0	0	0	0	0	0
0	0	0	0	0	0	0
0	0	0	0	0	0	0
0	0	0	0	0	0	0
0	0	0	0	0	0	0
0	0	0	0	0	0	0
0	0	0	0	0	0	0
0	10	0	26	10	28	0
0	0	0	0	0	0	0
0	0	0	0	0	0	0
0	0	0	0	0	0	0
0	0	0	0	0	0	0
0	0	0	0	0	35	0
0	0	0	0	0	0	0
0	0	0	0	0	0	0
11	0	0	0	0	48	0
0	0	0	0	0	0	0
0	0	0	0	0	0	0
0	0	0	0	0	0	0
0	0	0	0	0	0	0
0	0	0	0	0	0	0
0	0	0	0	0	0	0
0	0	0	0	0	0	0
0	0	0	0	0	0	0
0	0	0	0	0	0	0
0	0	0	0	0	0	0
0	0	0	0	0	0	0
0	0	0	0	0	0	0
0	14	0	0	0	279	0
0	0	0	0	0	0	0
0	0	0	0	0	0	0
0	0	0	0	0	0	0
0	0	0	0	0	0	0
0	0	0	0	0	0	0
0	0	0	0	0	0	0
0	0	0	0	0	0	0
0	0	0	0	0	0	0
0	0	0	0	0	0	0
0	0	0	27	33	0	0
0	0	0	0	0	0	0
0	0	0	0	0	0	0
0	0	0	0	0	0	0
0	27	0	0	0	0	0
0	0	0	0	0	59	0
10	0	0	0	0	64	0

41	0	0	15	72	0	0
25	0	0	10	89	0	0
0	0	12	135	11	48	0
0	0	41	35	14	0	0
0	0	0	0	0	0	0
0	0	40	15	16	0	0
0	0	0	11	0	0	0
0	0	0	0	0	0	0
0	0	0	10	0	0	0
0	0	0	0	0	0	0
0	0	0	0	0	0	0
0	0	0	0	0	12	0
0	0	0	0	0	0	0
0	0	0	0	0	0	0
0	0	0	0	0	0	0
38	0	0	28	0	0	0
0	0	0	25	0	0	0
0	0	0	0	0	0	0
0	0	0	0	0	0	0
0	0	0	0	0	21	0
0	0	0	0	0	11	0
15	0	10	55	0	0	0
30	0	0	55	0	0	0
0	0	0	222	0	0	0
0	17	0	14	0	0	0
0	0	0	0	0	0	0
47	0	0	10	0	0	0
0	42	0	0	0	0	0
0	0	0	0	0	0	0
0	54	0	16	0	0	0
0	123	0	16	0	98	0
0	0	0	0	0	0	0
18	0	0	0	13	162	0
0	0	0	0	66	0	0
0	25	0	0	0	0	0
0	10	0	0	0	0	0
0	16	0	12	0	0	0
0	0	0	0	0	0	0
0	0	0	0	0	0	0
0	0	11	12	0	0	0
0	0	0	0	0	0	0
0	0	0	0	0	0	0
203	0	0	221	0	51	0
18	0	0	0	0	0	0
0	0	0	0	0	52	0
0	0	0	0	0	181	0

0	0	0	0	0	100	0
12	0	11	36	0	115	0
67	0	69	0	41	0	0
10	33	0	0	0	0	0
45	60	0	0	0	0	0
0	0	0	0	0	23	0
0	0	0	0	0	11	0
0	0	0	0	0	0	0
0	0	0	0	0	0	0
0	0	0	0	0	0	0
0	0	0	0	0	0	0
0	0	0	43	0	0	0
0	0	0	11	0	0	0
0	0	0	42	0	0	0
0	0	0	0	0	0	0
0	0	0	0	0	0	0
0	0	55	0	11	0	0
0	0	0	0	0	0	0
26	0	0	0	11	0	0
156	0	40	52	0	0	0
0	0	0	10	0	0	0
0	0	0	46	0	0	0
0	0	106	0	0	0	0
0	0	217	0	0	12	0
0	0	0	0	0	0	0
0	0	0	0	12	10	0
0	0	0	0	0	0	0
0	0	0	0	0	0	0
0	0	0	0	0	0	0
0	0	0	0	0	0	0
0	0	0	26	0	0	0
0	0	0	49	30	0	0
0	0	0	11	0	0	0
22	0	0	0	18	21	0
0	24	0	0	0	0	0
0	10	0	0	0	10	0
0	10	0	0	0	0	0
0	16	0	12	21	34	0
0	0	0	0	0	25	0
0	0	0	0	0	11	0
0	0	0	0	0	12	0
0	0	0	14	242	118	0
0	0	10	0	0	0	0
0	0	0	0	0	0	0
0	0	0	0	0	0	0
0	76	0	0	0	33	0

224	0	0	0	0	0	0
0	0	0	21	0	28	0
0	0	0	20	0	92	0
11	0	0	0	0	30	0
0	0	0	0	0	0	0
0	0	0	0	0	0	0
0	0	0	0	0	0	0
0	26	0	0	0	0	0
0	0	0	0	0	0	0
0	0	0	0	0	0	0
0	0	0	0	0	0	0
0	0	0	0	0	0	0
0	0	0	0	0	0	0
24	0	18	0	0	0	0
0	0	0	0	0	0	0
0	0	0	10	0	0	0
0	0	0	0	0	46	0
0	0	0	0	0	18	0
0	0	0	0	0	31	0
0	0	0	0	0	436	0
12	0	12	0	0	10	0
0	0	0	0	0	0	0
0	0	0	0	0	0	0
0	0	0	0	0	0	0
0	0	0	0	0	0	0
15	0	0	0	0	11	0
0	0	0	0	0	0	0
0	0	0	0	0	0	0
0	0	0	0	0	29	0
0	0	0	0	0	88	0
0	0	0	0	0	11	0
0	0	0	0	0	0	0
0	0	0	0	0	0	0
0	0	0	0	0	16	0
0	0	0	0	0	33	0
0	0	0	16	0	124	0
0	0	0	0	0	11	0
0	0	0	32	0	133	0
0	0	0	0	0	48	0
0	0	0	12	0	27	0
0	0	0	0	0	0	0
0	0	0	0	0	0	0
0	0	0	0	0	0	0
0	0	0	0	0	0	0
0	0	0	0	0	0	0
0	0	0	0	0	33	0

10	0	0	0	0	87	0
20	0	0	18	0	0	0
0	0	33	11	128	0	0
0	0	0	0	0	0	0
0	0	0	0	0	0	0
0	0	0	0	0	0	0
0	0	0	0	0	0	0
0	0	46	0	0	0	0
0	0	0	0	0	0	0
10	0	0	54	22	0	0
0	0	0	0	0	0	0
0	0	0	0	0	0	0
0	0	0	0	0	0	0
0	0	0	0	0	0	0
0	0	0	0	0	12	0
0	0	0	0	0	0	0
0	0	0	22	0	0	0
81	0	68	0	0	30	0
0	0	0	0	0	0	0
0	0	0	0	0	0	0
0	0	0	0	0	0	0
0	0	0	0	0	0	0
0	0	0	0	0	0	0
0	176	59	0	0	77	0
0	0	0	11	32	0	0
0	0	0	0	0	0	0
0	0	0	0	0	0	0
0	38	95	0	0	0	0
0	82	54	0	0	0	0
0	207	373	0	0	0	0
10	0	0	0	0	45	0
0	0	0	0	0	0	0
0	0	0	0	0	0	0
0	114	43	0	0	0	0
0	90	0	46	12	0	0
0	0	0	32	0	0	0
0	0	0	0	0	0	0
0	0	0	0	0	0	0
0	0	0	0	0	0	0
0	0	63	0	0	0	0
0	0	0	0	0	0	0
0	24	0	0	0	0	0
0	0	0	10	15	0	0
0	0	0	0	17	0	0
0	34	0	0	0	0	0
0	0	0	0	36	0	0

0	0	0	0	0	0	0
0	0	0	0	0	0	0
0	0	0	0	0	0	0
0	0	0	0	0	0	0
35	0	11	0	17	194	0
72	0	128	0	13	35	0
0	0	0	0	0	0	0
0	0	0	0	0	0	0
0	0	0	0	0	0	0
0	0	0	0	0	0	0
0	0	0	0	0	0	0
0	0	0	0	0	11	0
0	0	0	0	0	0	0
0	0	0	0	0	0	0
0	0	0	0	0	0	0
0	0	0	0	0	0	0
0	0	0	0	0	0	0
0	0	0	0	0	10	0
0	0	0	24	0	0	0
0	0	0	0	0	0	0
0	0	0	0	0	0	0
0	0	0	0	10	10	0
0	0	0	0	0	0	0
0	0	0	138	0	0	0
0	0	0	0	0	0	0
0	0	0	0	0	0	0
0	0	0	0	0	0	0
0	0	0	0	0	0	0
0	0	0	0	0	0	0
0	0	0	0	0	0	0
0	0	0	0	0	0	0
0	11	0	10	0	16	0
0	0	0	0	0	0	0
0	0	0	0	0	0	0
0	0	0	0	0	0	0
0	0	0	0	0	0	0
0	0	0	0	0	13	0
0	12	0	0	0	0	0
0	0	0	0	0	0	0
0	0	0	0	0	0	0
0	0	0	0	0	0	0
0	0	0	0	0	0	0
0	0	0	0	0	0	0
0	0	0	0	0	0	0
0	0	0	0	0	0	0
0	0	0	0	0	0	0

0	0	0	0	0	22	0
0	0	0	0	0	0	0
0	0	0	0	0	0	0
0	0	0	0	0	0	0
0	0	0	0	0	0	0
0	0	0	0	0	0	0
0	0	0	0	0	0	0
0	0	0	0	0	31	0
0	0	0	0	0	0	0
17	0	11	0	0	0	0
0	0	0	0	0	0	0
0	0	0	0	0	0	0
0	10	0	0	0	0	0
0	98	0	0	0	0	0
0	27	50	0	383	607	0
0	12	0	0	0	0	0
0	13	0	0	0	0	0
0	26	0	0	0	0	0
0	33	0	0	0	0	0
0	29	35	10	0	0	0
0	11	0	0	0	0	0
0	46	0	0	0	0	0
0	60	0	0	0	0	0
0	85	0	23	0	0	0
0	12	0	255	10	0	0
0	0	0	0	0	0	0
13	0	0	0	0	0	0
0	0	0	0	0	0	0
0	0	0	0	0	32	0
0	0	0	0	0	0	0
0	0	0	0	0	11	0
48	0	0	0	0	0	0
0	0	0	0	0	0	0
0	0	0	0	0	0	0
55	0	0	0	0	0	0
0	0	0	0	0	0	0
0	0	0	0	0	0	0
0	0	0	0	0	0	0
0	0	0	44	0	0	0
0	0	0	0	0	0	0
0	0	0	0	0	0	0
0	0	0	0	0	0	0
0	0	0	24	0	0	0
0	0	0	0	0	0	0
39	0	0	0	0	0	0
0	0	0	0	0	0	0

0	0	0	0	0	0	0
0	0	0	0	0	0	0
0	0	0	0	0	0	0
0	0	0	0	0	0	0
0	0	0	23	0	0	0
0	0	0	0	0	0	0
0	0	0	115	0	0	0
0	0	0	49	17	0	0
0	0	0	0	0	0	0
14	0	32	0	0	0	0
0	0	0	0	10	0	0
0	0	0	0	0	0	0
0	0	0	0	0	56	0
0	0	0	0	0	0	0
0	0	0	0	0	0	0
0	0	0	0	0	34	0
0	0	0	52	16	18	0
0	0	0	0	0	62	0
0	0	0	0	0	0	0
0	0	0	0	0	198	0
0	0	0	0	0	0	0
10	0	0	12	0	28	0
0	0	0	0	0	0	0
0	0	0	0	0	0	0
0	0	0	15	0	15	0
0	0	0	0	0	46	0
0	0	0	10	0	113	0
0	0	43	13	0	0	0
0	0	0	0	0	0	0
0	0	0	0	0	0	0
0	0	0	0	0	0	0
0	0	0	0	0	0	0
0	0	0	0	0	0	0
0	0	0	0	0	22	0
0	0	0	25	0	0	0
0	0	0	0	0	0	0
42	0	0	0	0	0	0
0	0	0	0	0	64	0
0	0	0	0	0	44	0
28	0	0	0	0	25	0
0	0	0	15	0	20	0
0	0	0	0	0	50	0
14	0	0	0	0	0	0
0	0	0	20	0	128	0
0	0	0	0	0	285	0
0	0	0	0	0	20	0

0	0	0	21	0	13	0
0	0	0	0	0	0	0
11	0	0	14	0	0	0
0	0	0	0	0	0	0
0	0	0	0	0	10	0
0	0	0	0	0	76	0
31	0	0	0	0	60	0
41	0	0	0	0	43	0
0	0	0	0	0	29	0
0	0	0	0	0	205	0
0	0	0	0	0	66	0
0	0	0	0	0	0	0
0	0	0	0	0	0	0
0	0	0	0	0	0	0
0	0	0	0	0	0	0
0	0	0	0	0	0	0
0	0	0	0	0	10	0
0	0	0	0	0	70	0
0	0	0	53	0	478	0
0	0	0	0	0	0	0
0	0	0	0	0	0	0
0	0	0	49	0	99	0
0	0	0	0	0	0	0
0	0	0	0	0	0	0
0	0	0	0	0	0	0
0	0	0	22	0	10	0
0	0	0	0	0	0	0
0	0	0	0	0	0	0
0	0	0	0	0	0	0
0	0	0	0	0	0	0
0	0	11	0	0	0	0
0	0	0	34	0	0	0
0	0	0	0	0	0	0
0	0	0	0	0	0	0
0	0	0	0	0	0	0
0	0	0	0	0	0	0
0	0	0	0	0	0	0
0	0	0	0	0	0	0
0	0	0	11	0	0	0
0	0	0	0	0	0	0
0	0	0	0	51	0	0
0	0	0	10	43	0	0
0	0	0	0	0	0	0
0	0	0	0	0	0	0
0	0	0	0	0	0	0
0	0	0	0	0	0	0

0	0	0	0	0	0	0
0	0	0	0	0	0	0
0	0	0	0	0	0	0
0	0	12	0	0	0	0
0	0	0	0	0	0	0
0	0	0	16	0	0	0
0	0	0	0	0	0	0
0	0	0	0	0	0	0
0	0	0	0	0	0	0
0	0	0	0	0	0	0
0	0	0	0	0	0	0
0	0	0	0	0	0	0
0	0	0	0	0	0	0
0	0	0	0	0	0	0
0	0	0	0	0	0	0
0	0	0	0	0	0	0
0	0	0	0	0	0	0
0	0	0	0	0	0	0
0	0	0	0	0	0	0
0	0	0	0	0	0	0
0	0	0	0	0	0	0
15	0	0	0	0	0	0
0	0	0	0	0	0	0
0	0	0	0	0	0	0
0	0	0	0	0	0	0
0	0	0	0	0	0	0
0	0	0	0	0	0	0
0	0	0	0	0	0	0
0	0	0	0	0	0	0
0	0	0	0	0	0	0
0	0	0	0	0	0	0
0	0	0	0	0	0	0
0	0	0	0	0	0	0
0	0	0	0	0	0	0
0	0	0	0	0	0	0
0	0	0	30	10	10	0
0	0	0	0	0	0	0
0	0	0	0	0	0	0
0	0	0	0	0	0	0
0	0	0	0	0	0	0
0	0	0	0	0	0	0
0	0	0	11	0	0	0
0	0	0	0	0	0	0
0	0	0	0	0	0	0
0	0	0	0	0	0	0
0	0	0	0	0	0	0

0	0	0	0	0	0	0
0	0	0	0	120	10	0
0	0	0	10	174	0	0
0	0	0	0	0	0	0
0	0	0	0	0	0	0
0	0	0	13	0	29	0
0	0	0	0	0	0	0
0	0	0	0	0	0	0
10	0	0	0	0	0	0
0	0	0	0	0	0	0
0	0	0	0	0	0	0
0	0	0	0	0	0	0
0	0	0	0	0	0	0
0	0	0	0	0	0	0
0	0	0	0	0	0	0
22	0	0	0	0	0	0
0	0	0	0	0	0	0
0	0	0	0	0	0	0
0	0	23	0	0	0	0
0	0	0	0	0	0	0
0	0	0	0	0	0	0
0	0	29	0	0	0	0
0	0	0	0	0	0	0
0	0	0	0	0	0	0
0	0	0	0	0	0	0
0	0	0	0	0	0	0
0	0	0	0	0	0	0
0	0	0	0	0	0	0
0	0	31	0	0	28	0
0	0	34	0	0	0	0
0	0	0	0	0	0	0
0	0	0	0	0	0	0
0	0	0	0	0	0	0
13	0	0	40	0	0	0
0	0	12	68	0	0	0
0	0	0	0	47	0	0
0	0	0	0	11	0	0
0	0	0	0	35	31	0
0	0	19	0	17	64	0
0	0	0	0	13	0	0
34	0	0	0	11	0	0
0	0	0	0	12	0	0
13	0	0	0	11	0	0
31	0	0	0	172	0	0
0	0	0	0	10	0	0
0	0	0	0	10	0	0

0	0	0	0	10	0	0
0	0	0	0	69	0	0
0	0	0	0	0	0	0
0	0	0	0	0	0	0
0	0	0	0	0	0	0
0	0	0	0	0	0	0
0	0	0	0	0	0	0
41	0	12	0	0	0	0
0	0	0	0	0	0	0
0	0	0	0	0	25	0
0	0	0	0	0	0	0
0	0	0	0	0	0	0
0	0	0	0	0	0	0
0	0	0	0	0	0	0
0	0	0	0	0	0	0
0	0	11	0	0	0	0
0	0	10	0	0	0	0
19	0	0	26	0	0	0
0	0	0	0	0	0	0
0	0	0	0	0	26	0
0	0	0	0	0	0	0
0	0	0	0	0	0	0
0	0	0	0	0	0	0
0	0	0	0	0	0	0
0	0	0	0	0	0	0
0	0	0	0	0	0	0
0	0	0	0	0	0	0
0	0	0	0	0	0	0
0	0	0	0	0	0	0
0	0	0	0	0	86	0
0	0	0	0	0	102	0
0	0	0	0	0	0	0
0	0	0	0	0	49	0
0	0	0	0	0	0	0
0	0	0	0	0	0	0
0	0	0	0	0	0	0
0	0	0	0	0	0	0
30	0	0	0	0	0	0
0	0	0	0	0	0	0
37	0	0	0	0	0	0
27	0	0	28	0	54	0
0	0	0	0	0	0	0
0	0	0	0	0	0	0
33	0	0	0	0	0	0
0	0	0	0	0	0	0
0	0	0	0	0	0	0

0	0	0	0	0	0	0
0	0	0	0	0	0	0
0	0	0	0	0	16	0
0	0	0	0	0	0	0
43	0	0	0	0	0	0
13	0	0	0	0	0	0
0	0	0	0	0	0	0
0	0	0	0	0	0	0
0	0	0	0	0	0	0
0	0	0	0	0	0	0
0	0	0	10	0	0	0
58	0	0	0	0	0	0
0	0	0	0	0	0	0
0	0	0	0	0	0	0
0	0	0	0	0	0	0
0	0	0	0	0	0	0
0	0	12	36	0	0	0
0	0	0	10	0	0	0
17	0	0	0	0	0	0
0	0	0	0	0	0	0
0	0	0	0	0	0	0
0	0	0	0	0	0	0
0	0	0	0	0	0	0
0	0	0	0	0	0	0
0	0	0	0	0	16	0
0	0	0	0	0	0	0
0	0	0	0	0	0	0
0	0	11	0	0	0	0
0	0	0	0	0	0	0
0	0	0	0	0	0	0
0	0	0	0	0	0	0
0	0	0	0	0	0	0
21	0	0	0	0	0	0
0	0	0	0	0	0	0
0	0	0	0	0	77	0
0	0	0	0	0	0	0
0	0	0	0	0	0	0
0	0	0	0	0	0	0
0	0	0	0	0	0	0
0	0	0	0	0	0	0
0	0	0	0	0	0	0
0	0	0	0	0	0	0
0	0	0	0	0	0	0
0	0	10	0	0	0	0
0	0	0	0	0	0	0
0	0	0	0	0	0	0

0	0	0	0	0	0	0
0	0	0	0	0	0	0
0	0	0	0	0	0	0
0	0	0	0	0	0	0
0	0	0	0	0	0	0
0	0	0	0	0	0	0
0	0	0	0	0	0	0
0	0	0	0	0	0	0
0	0	0	0	0	0	0
0	0	15	0	0	0	0
0	0	0	0	0	0	0
0	0	0	0	0	0	0
0	0	0	0	0	0	0
0	0	0	0	0	0	0
0	0	0	0	0	0	0
0	0	0	0	0	0	0
0	0	0	0	0	0	0
0	0	0	0	0	0	0
0	0	0	0	0	0	0
0	0	17	0	0	0	0
0	0	0	65	0	67	0
0	0	0	0	0	0	0
0	0	13	0	0	0	0
0	0	0	0	0	0	0
0	0	0	0	0	0	0
0	0	0	0	0	0	0
0	0	0	0	0	0	0
0	0	0	0	0	0	0
0	0	0	0	0	0	0
0	0	0	0	0	0	0
0	0	0	0	0	0	0
0	0	0	0	0	0	0
0	0	0	0	0	0	0
0	0	0	0	0	0	0
0	0	0	0	0	0	0
0	0	0	0	0	0	0
0	0	0	0	0	0	0
0	0	0	0	0	0	0
0	0	0	0	0	0	0
0	0	0	0	0	0	0
0	0	0	0	0	0	0
0	0	0	0	0	0	0
0	0	0	0	0	0	0
0	0	0	0	0	0	0
0	0	0	0	0	0	0
0	0	0	0	0	29	0

0	0	0	0	0	0	0
0	0	0	0	0	0	0
0	0	0	24	0	0	0
0	0	0	0	0	0	0
0	0	0	0	0	0	0
0	0	0	0	0	0	0
0	0	0	0	0	0	0
0	0	0	0	0	0	0
0	0	0	0	0	0	0
0	0	0	0	0	0	0
0	0	0	0	0	0	0
0	0	0	0	0	0	0
0	0	0	0	0	0	0
0	0	0	0	0	0	0
0	0	0	0	0	0	0
0	0	0	0	0	0	0
0	0	10	0	0	0	0
0	0	0	0	0	0	0
0	0	0	0	0	0	0
0	0	0	0	0	0	0
0	0	0	0	0	0	0
0	0	0	0	0	0	0
0	0	0	0	0	0	0
0	0	0	0	0	0	0
0	0	0	0	0	0	0
0	0	0	0	0	0	0
0	0	0	0	0	0	0
0	0	0	0	0	0	0
0	0	0	0	0	0	0
0	0	0	0	0	0	0
0	0	0	0	0	0	0
0	0	0	0	0	0	0
0	0	0	0	0	0	0
0	0	0	0	0	0	0
0	0	0	0	0	0	0
0	0	0	18	0	47	0
0	0	0	0	0	0	0
0	0	0	0	0	0	0
0	0	0	0	0	0	0
0	0	0	0	0	0	0
0	0	0	0	0	0	0
0	0	0	0	0	0	0
0	0	0	0	0	0	0
0	0	0	0	0	0	0
0	0	0	0	0	0	0
0	0	0	0	0	0	0

0	0	0	0	0	0	0
0	0	12	0	0	0	0
24	0	0	0	0	0	0
0	0	0	10	0	0	0
0	0	0	0	0	0	0
0	0	0	0	0	0	0
0	0	0	0	0	41	0
0	0	0	0	0	0	0
0	0	0	0	0	0	0
0	0	0	0	0	0	0
10	0	0	0	0	0	0
0	0	0	0	0	0	0
0	0	0	11	0	0	0
0	0	0	0	0	0	0
0	0	0	0	0	0	0
0	0	0	0	0	0	0
0	0	0	0	0	0	0
0	0	0	0	0	0	0
0	0	0	0	0	0	0
0	0	0	0	0	0	0
0	0	0	0	0	0	0
0	0	0	0	0	0	0
10	0	0	0	0	0	0
0	0	0	0	0	0	0
0	0	0	0	0	0	0
0	0	0	0	0	0	0
0	0	0	0	0	0	0
0	0	0	0	0	0	0
0	0	0	0	0	0	0
0	0	0	0	0	0	0
0	0	0	0	0	0	0
0	0	0	0	0	0	0
0	0	0	0	0	0	0
0	0	0	0	0	0	0
0	0	0	0	0	0	0
0	0	0	0	0	0	0
0	0	0	0	0	0	0
0	0	0	0	0	0	0
0	0	0	0	0	0	0
0	0	0	0	0	0	0
0	0	0	0	0	0	0
0	0	0	11	0	0	0
0	0	0	0	0	0	0
0	0	0	0	0	0	0
0	0	0	0	0	0	0
0	0	0	0	0	0	0

0	0	0	0	0	0	0
0	0	0	0	0	0	0
0	0	0	0	0	0	0
0	0	0	46	0	12	0
0	0	0	0	0	0	0
0	0	0	0	0	0	0
0	0	0	0	0	0	0
0	0	0	0	0	0	0
0	0	0	0	0	0	0
0	0	11	0	0	0	0
54	0	21	0	0	0	0
0	0	13	0	0	0	0
0	0	11	0	0	0	0
0	0	40	0	0	0	0
0	0	33	0	0	0	0
0	0	10	0	0	0	0
0	0	13	0	0	0	0
0	0	52	0	0	0	0
0	0	13	17	0	0	0
0	0	10	0	0	0	0
0	0	35	0	0	0	0
0	0	15	0	0	0	0
0	0	52	0	0	0	0
0	0	11	0	0	0	0
0	0	0	0	0	0	0
0	0	0	0	0	0	0
0	0	0	0	0	0	0
0	0	0	0	0	0	0
0	0	0	0	0	0	0
0	0	0	0	0	0	0
0	0	0	0	0	0	0
0	0	0	0	0	0	0
0	0	0	0	0	0	0
0	0	0	0	0	0	0
0	0	0	0	0	0	0
0	0	0	0	0	34	0
0	0	0	0	0	0	0
0	0	0	0	0	0	0
0	0	0	0	0	0	0
0	0	0	0	0	0	0
0	0	0	0	0	0	0
0	0	0	0	0	0	0
0	0	0	0	0	0	0
0	0	0	0	0	0	0
0	0	0	0	0	0	0
0	0	0	0	0	0	0

0	0	0	0	0	0	0
0	0	0	0	0	0	0
0	0	0	0	0	0	0
0	0	0	25	0	0	0
0	0	0	0	0	0	0
0	0	0	0	0	0	0
0	0	0	0	0	0	0
0	0	0	16	0	0	0
0	0	0	0	0	0	0
0	0	0	0	0	0	0
0	0	0	0	0	0	0
0	0	0	0	0	0	0
0	0	0	0	0	0	0
0	0	0	0	0	0	0
0	0	0	0	0	0	0
0	0	0	0	0	0	0
0	0	0	0	0	0	0
0	0	0	0	0	0	0
0	0	0	0	0	0	0
0	0	0	0	0	0	0
0	0	0	0	0	0	0
0	0	0	0	0	0	0
0	0	0	0	0	0	0
0	0	0	0	0	0	0
0	0	0	0	0	0	0
0	0	0	0	0	0	0
0	0	0	0	0	0	0
0	0	0	0	0	0	0
0	0	0	0	0	0	0
0	0	0	0	0	0	0
58	0	0	0	0	0	0
0	0	0	0	0	0	0
0	0	0	0	0	0	0
0	0	0	0	0	0	0
0	0	0	0	0	0	0
0	0	0	0	0	0	0
0	0	0	11	0	12	0
0	0	0	0	0	0	0
0	0	0	0	0	0	0
0	0	0	0	0	25	0
0	0	0	0	0	0	0
0	0	0	0	0	0	0
0	0	0	0	0	0	0
0	0	0	0	0	0	0
0	0	0	0	0	0	0
0	0	0	0	0	0	0

0	0	0	0	0	0	0
0	0	0	0	0	0	0
0	0	0	0	0	0	0
0	0	0	0	0	0	0
0	0	0	0	0	0	0
0	0	0	0	0	0	0
0	0	0	0	0	12	0
0	0	0	0	0	0	0
0	0	0	0	0	0	0
0	0	0	0	0	0	0
0	0	0	13	0	0	0
0	0	0	0	0	0	0
0	0	0	0	0	0	0
0	0	0	0	0	0	0
0	0	0	0	0	0	0
0	0	0	0	0	0	0
0	0	0	0	0	0	0
0	0	0	0	0	0	0
0	0	0	0	0	0	0
0	0	0	0	0	0	0
0	0	0	0	0	0	0
10	0	0	0	0	0	0
0	0	0	0	0	0	0
0	0	0	0	0	0	0
0	0	0	0	0	0	0
0	0	0	0	0	0	0
0	0	0	0	0	0	0
0	0	0	0	0	0	0
0	0	0	0	0	0	0
0	0	0	0	0	11	0
0	0	0	0	0	0	0
0	0	0	0	0	0	0
0	0	0	118	0	0	0
0	0	0	0	0	0	0
0	0	0	0	0	0	0
0	0	0	0	0	0	0
0	0	0	0	0	0	0
0	0	0	0	0	0	0
0	0	0	0	0	0	0
0	0	0	0	0	0	0
0	0	0	0	0	0	0
0	0	0	0	0	0	0
0	0	0	0	0	0	0
0	0	0	0	0	0	0
0	0	0	0	0	0	0
0	0	0	0	0	0	0

0	0	0	0	0	0	0
0	0	0	0	0	0	0
0	0	0	0	0	0	0
0	0	0	0	0	0	0
0	0	0	0	0	0	0
0	0	0	0	0	0	0
19	0	0	0	0	0	0
14	0	0	0	0	0	0
43	0	0	0	0	0	0
10	0	0	0	0	0	0
44	0	0	0	0	0	0
59	0	0	24	0	0	0
14	0	0	0	0	0	0
14	0	0	0	0	0	0
11	0	0	0	0	0	0
26	0	0	0	0	0	0
17	0	0	0	0	0	0
13	0	0	0	0	0	0
39	0	0	0	0	0	0
13	0	0	0	0	0	0
10	0	0	0	0	21	0
10	0	0	0	0	0	0
29	0	0	0	0	0	0
13	0	0	0	0	0	0
10	0	0	0	0	0	0
13	0	0	0	0	0	0
15	0	0	0	0	0	0
26	0	0	0	0	0	0
21	0	0	0	0	0	0
15	0	0	0	0	0	0
12	0	0	0	0	0	0
109	0	0	0	0	0	0
10	0	0	0	0	0	0
104	0	0	0	0	0	0
0	0	0	0	0	0	0
0	0	0	0	0	0	0
0	0	0	0	0	21	0
0	0	0	0	0	0	0
0	0	0	0	0	0	0
0	0	0	0	0	11	0
0	0	0	0	0	0	0
0	0	0	0	0	0	0
0	0	0	0	0	0	0
0	0	0	0	0	0	0
0	0	0	0	0	0	0
0	0	0	0	0	0	0

0	0	0	0	0	0	0
0	0	0	0	0	0	0
0	0	0	0	0	0	0
0	0	0	0	0	0	0
0	0	0	0	0	0	0
0	0	0	0	0	0	0
0	0	0	0	0	0	0
0	0	0	0	0	0	0
0	0	0	0	0	0	0
0	0	0	0	0	0	0
0	0	0	0	0	0	0
0	0	0	0	0	0	0
0	0	0	0	0	0	0
0	0	0	0	0	0	0
0	0	0	0	0	0	0
0	0	0	0	0	0	0
0	0	0	0	0	0	0
0	0	0	0	0	11	0
0	0	0	0	0	0	0
0	0	0	0	0	0	0
0	0	0	0	0	11	0
0	0	0	0	0	0	0
0	0	0	0	0	0	0
0	0	0	0	0	0	0
0	0	0	0	0	0	0
0	0	0	0	0	0	0
0	0	0	0	0	0	0
0	0	0	0	0	0	0
0	0	0	0	0	0	0
0	0	0	0	0	0	0
0	0	0	0	0	0	0
0	0	0	0	0	0	0
0	0	0	0	0	0	0
0	0	0	0	0	0	0
0	0	0	0	0	0	0
0	0	0	0	0	0	0
0	0	0	0	0	0	0
0	0	0	0	0	0	0
0	0	0	0	0	0	0
0	0	0	0	0	0	0
0	0	0	0	0	0	0
0	0	0	0	0	0	0
0	0	0	0	0	0	0
0	0	0	11	0	0	0
0	0	0	11	0	0	0
0	0	0	24	0	0	0

0	0	0	61	0	0	0
0	0	0	21	0	0	0
0	0	0	0	0	0	0
0	0	0	0	0	0	0
0	0	0	0	0	0	0
0	0	0	0	0	0	0
0	0	0	68	0	0	0
0	0	0	0	0	0	0
0	0	0	0	0	0	0
0	0	0	0	0	0	0
0	0	0	0	0	0	0
0	0	0	0	0	0	0
0	0	0	0	0	0	0
0	0	0	0	0	0	0
0	0	0	0	0	0	0
0	0	0	0	0	0	0
0	0	0	0	0	0	0
0	0	0	0	0	0	0
0	0	0	0	0	0	0
0	0	0	31	0	0	0
0	0	0	0	0	0	0
0	0	0	0	0	0	0
0	0	0	0	0	0	0
0	0	0	0	0	0	0
0	0	0	0	0	0	0
0	0	0	0	0	0	0
0	0	0	0	0	38	0
0	0	0	0	0	0	0
0	0	0	0	0	14	0
0	0	0	0	0	0	0
0	0	0	0	0	0	0
0	0	0	0	0	0	0
0	0	0	0	0	0	0
0	0	0	0	0	0	0
0	0	0	0	0	24	0
0	0	0	0	0	0	0
0	0	0	0	0	0	0
0	0	0	0	0	0	0
0	0	0	0	0	0	0
0	0	0	0	0	0	0
0	0	0	0	0	0	0
0	0	0	0	0	0	0
0	0	0	0	0	0	0
0	0	0	0	0	0	0
0	0	0	0	0	0	0
0	0	0	0	0	15	0

0	0	0	0	0	0	0
0	0	0	0	0	0	0
0	0	0	0	0	0	0
0	0	0	0	0	0	0
0	0	0	0	0	0	0
0	0	0	0	0	0	0
0	0	0	0	0	10	0
0	0	0	0	0	0	0
0	0	0	0	0	0	0
0	0	0	0	0	0	0
0	0	0	0	0	0	0
0	0	0	0	0	0	0
0	0	0	0	0	0	0
0	0	0	0	0	0	0
0	0	0	0	0	0	0
0	0	0	0	0	0	0
0	0	0	0	0	0	0
0	0	0	0	0	0	0
0	0	0	0	0	0	0
0	0	0	0	0	0	0
0	0	0	0	0	53	0
0	0	0	0	0	0	0
0	0	0	0	0	11	0
0	0	0	0	0	0	0
0	0	0	0	0	0	0
0	0	0	0	0	0	0
0	0	0	0	0	0	0
0	0	0	0	0	0	0
0	0	0	0	0	0	0
0	0	0	0	0	0	0
0	0	0	0	0	0	0
0	0	0	0	0	0	0
0	0	0	0	0	12	0
0	0	0	0	0	10	0
0	0	0	0	0	44	0
0	0	0	0	0	19	0
0	0	0	0	0	12	0
0	0	0	0	0	34	0
0	0	0	0	0	12	0
0	0	0	0	0	10	0
0	0	0	0	0	33	0
0	0	0	0	0	29	0
0	0	0	0	0	10	0
0	0	0	0	0	20	0
0	0	0	0	0	26	0
0	0	0	0	0	54	0

0	0	0	0	0	25	0
0	0	0	0	0	53	0
0	0	0	0	0	21	0
0	0	0	0	0	62	0
0	0	0	0	0	0	0
0	0	0	0	0	0	0
0	0	0	0	0	0	0
0	0	0	0	0	0	0
0	0	0	0	0	0	0
0	0	0	0	0	0	0
0	0	0	0	0	0	0
0	0	0	0	0	0	0
0	0	0	0	0	0	0
0	0	0	0	0	0	0
0	0	0	0	0	0	0
0	0	0	0	0	0	0
0	0	0	0	0	0	0
0	0	0	0	0	0	0
0	0	0	0	0	0	0
0	0	0	0	0	0	0
0	0	0	0	0	0	0
0	0	0	0	0	0	0
0	0	0	0	0	0	0
0	0	0	0	0	0	0
0	0	0	0	0	0	0
0	0	0	0	0	0	0
0	0	0	0	0	0	0
0	0	0	0	0	0	0
0	0	0	0	0	0	0
0	0	0	0	0	0	0
0	0	0	0	0	0	0
0	0	0	0	0	0	0
0	0	0	0	0	0	0
0	0	0	0	0	0	0
0	0	0	0	0	0	0
0	0	0	0	0	0	0
0	0	0	0	0	0	0
0	0	0	0	0	0	0
0	0	0	0	0	0	0
0	0	0	0	0	0	0
0	0	0	0	0	0	0
0	0	0	0	0	0	0
0	0	0	0	0	0	0
0	0	0	0	0	0	0
0	0	0	0	0	0	0
0	0	0	0	0	0	0

0	0	0	0	0	0	0
0	0	0	0	0	0	0
0	0	0	0	0	0	0
0	0	0	0	0	0	0
0	0	0	0	0	0	0
0	0	0	0	0	0	0
0	0	0	0	0	0	0
0	0	0	0	0	0	0
0	0	0	0	0	0	0
0	0	0	0	0	0	0
0	0	0	0	0	0	0
0	0	0	0	0	0	0
0	0	0	0	0	0	0
0	0	0	0	0	0	0
0	0	0	0	0	0	0
0	0	0	0	0	0	0
0	0	0	0	0	0	0
0	0	0	0	0	0	0
0	0	0	0	0	0	0
0	0	0	0	0	0	0
0	0	0	0	0	0	0
0	0	0	0	0	0	0
0	0	0	0	0	0	0
0	0	0	0	0	0	0
0	0	0	0	0	0	0
0	0	0	0	0	0	0
0	0	0	0	0	0	0
0	0	0	0	0	0	0
0	0	0	0	0	0	0
0	0	0	0	0	0	0
0	0	0	0	0	0	0
0	0	0	0	0	0	0
0	0	0	0	0	0	0
0	0	0	0	0	0	0
0	0	0	0	0	0	0
0	0	0	0	0	0	0
0	0	0	0	0	0	0
0	0	0	0	0	0	0
0	0	0	0	0	0	0
0	0	0	0	0	0	0
0	0	0	0	0	0	0
0	0	0	0	0	0	0
0	0	0	0	0	0	0
0	0	0	0	0	0	0
0	0	0	0	0	0	0
0	0	0	0	0	0	0

0	0	0	0	0	0	0
0	0	0	0	0	0	0
0	0	0	0	0	0	0
0	0	0	0	0	0	0
0	0	0	0	0	0	0
0	0	0	0	0	0	0
0	0	0	0	0	0	0
0	0	0	0	0	0	0
0	0	0	0	0	0	0
0	0	0	0	0	0	0
0	0	0	0	0	0	0
0	0	0	0	0	0	0
0	0	0	0	0	0	0
0	0	0	0	0	0	0
0	0	0	0	0	0	0
0	0	0	0	0	0	0
0	0	0	0	0	0	0
0	0	0	0	0	0	0
0	0	0	0	0	0	0
0	0	0	0	0	0	0
0	0	0	12	0	0	0
0	0	0	16	0	0	0
0	0	0	35	0	0	0

UC_333	UC_340	UC_341	UC_342	UC_348
80	0	0	37	0
14	0	0	0	0
104	100	0	133	62
101	112	34	216	284
25	82	0	210	79
39	120	0	76	0
79	10	0	13	0
0	58	0	39	100
70	100	12	122	0
142	296	136	473	130
0	102	10	47	0
0	0	0	10	0
28	27	0	18	27
31	0	0	0	42
0	13	19	64	0
61	38	0	41	0
1543	1600	2074	3752	683
0	89	121	112	195
44	47	50	50	10
231	90	48	103	378
191	0	10	13	57
283	25	15	33	12
60	15	0	134	69
0	0	0	32	0
1662	4178	408	1979	10987
698	1769	179	559	2649
209	1009	370	432	1435
138	751	144	300	954
336	1978	311	820	3468
57	84	0	104	0
96	88	23	19	33
149	171	21	18	0
1887	4206	3120	1266	759
0	13	0	24	0
36	32	34	60	215
40	0	41	0	72
87	33	0	22	44
828	500	244	918	138
0	83	232	64	218
2658	5982	4666	5235	2772
281	225	390	189	12

140	281	795	246	13
224	590	577	157	854
271	384	641	171	568
282	3666	1083	3383	1688
125	191	1339	399	797
35	73	14	23	0
35	147	36	240	0
0	248	0	108	0
0	69	14	13	0
0	103	11	34	0
0	0	0	0	0
0	127	14	28	0
23	436	37	462	0
0	57	0	0	0
0	27	0	0	0
0	274	17	92	0
0	42	0	13	0
1434	1291	305	1393	1759
67	242	14	169	452
131	63	0	86	0
0	40	0	0	104
10	0	0	0	0
0	0	0	0	0
61	45	0	35	0
10	11	0	0	43
0	93	0	53	0
0	34	0	0	0
0	0	0	0	0
0	0	0	0	0
62	138	10	83	0
59	1001	149	499	0
582	2780	532	2873	229
61	95	10	60	0
33	101	11	0	0
0	51	0	25	0
0	56	0	83	0
0	77	0	17	0
23	214	13	14	0
96	75	0	14	0
0	63	0	11	0
21	565	129	113	0
31	114	0	30	0
10	28	0	39	0
0	12	0	0	0
12	533	127	181	0
561	3546	620	1941	103

10	63	57	25	74
132	410	100	354	148
0	108	10	302	0
0	69	0	0	0
109	2898	383	3690	29
30	25	13	242	0
39	0	0	0	0
20	25	0	23	0
0	16	0	0	0
35	33	0	75	0
257	160	62	169	37
42	136	11	0	0
8190	9562	1193	9400	3561
0	48	0	65	10
725	1083	153	1788	168
0	168	31	74	88
104	107	0	46	164
44	239	39	59	0
69	150	0	158	0
0	110	0	32	0
0	0	0	0	0
0	32	0	24	0
0	53	0	40	31
515	447	45	86	72
792	983	237	730	353
878	854	69	229	111
477	485	18	127	94
21	87	0	10	510
83	223	31	158	448
1108	1517	507	1908	918
49	117	0	201	79
50	46	0	15	0
50	58	33	97	43
36	79	111	416	310
0	16	0	19	0
52	71	0	30	0
179	182	26	64	0
218	181	39	165	32
39	142	0	0	0
1870	1810	193	527	221
85	115	0	46	27
55	69	12	43	13
55	234	0	150	182
1956	6096	1636	7534	21777
43	228	11	86	1398
661	1521	711	1060	14403

41	0	0	23	0
401	392	20	79	87
594	568	110	377	203
571	2077	523	4531	105
0	90	0	44	0
115	263	59	347	29
0	0	0	0	0
43	106	28	28	0
0	31	0	0	0
0	0	0	0	0
133	259	41	346	67
207	120	10	126	112
0	99	27	77	0
195	313	16	16	48
545	809	118	324	146
0	60	0	62	0
0	155	0	124	31
0	0	0	56	0
0	0	0	0	0
43	10	0	51	13
181	207	62	299	29
0	11	0	25	0
400	564	89	581	90
26	104	0	27	0
184	94	13	103	20
103	477	90	666	341
167	142	62	397	52
550	491	306	1724	631
55	72	22	141	23
31	71	15	295	10
10	47	0	0	0
72	114	15	249	270
251	412	42	174	131
12	100	33	228	0
183	140	27	299	137
108	446	74	340	578
0	237	129	84	0
0	0	0	12	0
12	15	0	73	0
0	0	0	0	0
341	216	0	0	15
0	0	0	0	0
0	100	51	66	0
0	0	0	0	0
20	141	22	320	115
64	31	201	520	242

85	0	175	258	590
40	19	104	733	153
297	937	769	2728	7947
573	127	1123	2709	2606
0	84	0	167	10
223	352	95	953	223
439	464	156	1260	71
23	28	0	69	0
18	15	0	49	27
0	0	0	0	0
0	20	0	13	12
0	0	0	10	0
0	38	0	64	127
476	934	540	2438	1144
0	0	23	116	156
33	912	150	1819	0
30	0	0	0	10
0	0	0	0	52
0	0	0	0	0
0	0	0	0	0
0	0	0	0	0
1853	404	476	1770	10958
0	10	15	12	23
0	58	17	224	0
0	35	26	30	238
0	75	19	103	73
285	85	46	186	206
0	0	0	62	0
0	37	0	0	0
0	0	0	0	0
0	0	0	0	0
0	24	0	41	0
0	0	11	0	0
0	0	0	0	0
15	0	0	0	0
95	140	0	152	49
4921	3809	1086	4835	4334
2753	2405	631	2306	2302
311	60	41	80	118
0	0	0	0	53
0	0	0	0	81
276	618	110	607	23
444	360	22	218	23
57	91	16	117	0
0	44	25	10	0
26	534	147	810	14

0	167	50	355	0
25	398	50	258	12
274	114	67	112	1284
101	55	0	42	0
235	172	43	165	23
2319	2412	579	2089	1663
217	276	76	733	364
110	84	0	368	0
0	29	0	22	11
0	0	0	0	0
0	113	0	37	0
0	22	0	0	0
0	11	11	97	0
739	792	134	762	439
0	0	0	77	38
65	0	0	0	0
0	0	0	29	0
0	11	0	112	0
354	519	118	327	443
29	10	0	0	481
79	0	0	0	0
127	0	0	0	0
346	483	56	75	57
61	0	32	15	0
2349	1297	846	1517	3353
167	81	106	169	96
0	0	0	93	0
312	334	201	1310	336
632	412	99	449	43
234	120	33	103	36
0	42	14	83	276
179	68	53	234	22
0	0	0	0	0
102	18	29	72	0
465	280	360	1004	1996
88	47	51	123	236
120	111	105	244	243
80	0	0	55	0
0	0	0	0	0
296	394	38	639	56
174	276	57	762	47
124	190	16	150	53
10	58	0	0	0
1192	1110	156	332	110
0	0	0	0	0
2130	3592	160	1359	358

5780	4060	252	2527	623
83	141	0	0	0
334	100	18	50	0
695	1638	43	479	966
386	870	13	329	21
254	285	0	196	54
93	160	28	129	11
1247	944	16	212	160
107	114	0	0	0
23	41	0	0	0
1052	0	0	0	0
487	364	48	454	161
4985	3227	627	4116	1033
759	493	69	398	64
533	111	62	305	52
598	348	217	440	214
2583	958	606	1530	438
72	141	45	24	89
28	129	22	56	114
172	260	70	121	281
48	115	0	33	0
68	227	98	105	302
204	1376	382	463	629
50	389	64	105	2603
13	416	65	107	1839
225	193	96	63	34
2818	8661	16468	11350	15512
152	1272	3087	425	1385
112	143	428	392	812
98	338	1085	766	61
4536	1370	2917	6084	1016
57	1594	4002	4885	254
45	853	2602	325	3784
68	0	0	0	0
79	1570	1904	2276	95
837	958	40	691	2522
56	197	0	41	507
426	2506	1162	1242	2705
563	1157	109	880	5043
446	2393	441	297	1889
1506	2196	90	347	229
1559	2784	170	212	311
19	178	48	259	35
159	211	118	243	116
590	149	0	140	152
53	41	0	16	48

1071	1513	270	2420	868
142	215	45	156	97
501	320	36	82	0
384	380	162	222	86
12	11	0	11	23
10	0	0	38	0
90	285	15	806	443
33	113	39	79	17
96	74	0	87	450
62	10	0	34	312
0	47	0	41	38
606	691	142	1037	1847
0	11	0	0	298
63	74	0	11	0
10	0	0	0	0
0	0	0	0	0
0	10	0	40	268
22	13	0	169	0
693	940	184	1321	257
0	0	0	85	0
0	0	0	21	0
0	0	0	124	0
0	0	0	0	0
501	793	179	645	129
0	0	0	0	0
14	51	0	41	0
67	16	0	12	25
59	41	12	44	11
0	0	0	10	0
0	11	0	12	0
0	0	0	0	0
0	0	0	0	0
0	0	0	0	0
0	43	0	0	0
135	120	67	156	297
0	0	0	0	0
0	0	0	0	0
0	0	0	0	0
0	0	0	0	0
11	17	0	52	0
0	13	0	0	0
116	169	23	89	0
62	107	34	166	0
63	109	33	149	13
0	0	0	0	0
0	0	0	0	0

98	104	57	141	221
0	0	0	0	0
0	0	0	26	26
2218	2479	519	3875	477
34	28	0	109	0
10	25	0	108	0
88	42	48	187	207
81	63	37	282	133
0	13	17	121	164
86	43	27	14	0
112	68	20	296	346
274	377	178	942	640
107	283	183	645	1266
236	525	277	1217	508
55	53	37	85	294
0	0	0	0	0
0	0	0	0	0
37	88	75	62	123
0	0	0	0	0
0	0	0	0	11
67	111	33	161	22
0	0	0	0	0
69	93	16	241	100
101	402	191	616	451
0	0	0	0	0
0	0	0	0	0
116	71	0	66	45
0	0	0	0	0
1073	1298	250	1186	211
38	66	0	30	0
31	69	0	0	13
150	209	46	25	45
101	93	0	73	12
0	0	0	27	0
0	0	0	0	0
0	0	0	0	0
0	0	0	0	0
470	470	380	665	1155
0	0	0	0	0
52	88	49	25	388
45	145	61	68	1091
34	0	0	20	74
0	0	0	10	0
26	13	0	0	22
0	0	0	0	0
0	0	0	0	13

401	24	23	47	24
31	0	11	79	0
0	0	0	0	11
0	0	0	0	0
0	0	0	0	0
0	0	0	34	0
0	0	0	0	135
78	10	0	44	0
2582	2117	620	4510	1363
94	125	36	170	126
85	22	0	84	27
932	1014	310	1399	256
0	0	0	12	0
0	0	0	41	0
97	119	0	119	0
2619	2204	613	1427	540
105	104	0	130	0
0	0	0	0	0
10	14	0	40	40
0	0	0	0	0
0	0	0	0	0
12	14	0	60	21
0	15	0	95	0
134	254	33	401	87
265	453	439	426	341
1554	1542	699	1788	927
142	152	38	237	194
154	79	12	44	74
195	341	130	165	486
25	30	0	0	36
0	0	0	0	0
0	0	0	0	0
52	89	24	11	34
40	55	0	73	15
224	158	64	319	68
117	58	0	27	63
0	280	133	117	62
835	8097	2667	7077	1716
45	259	378	437	810
0	0	0	0	0
12	1049	271	980	0
0	19	0	14	0
0	50	0	64	0
0	95	15	0	0
25	0	0	0	0
97	246	648	166	116

0	118	0	65	0
0	56	0	24	0
0	0	0	0	0
86	944	320	252	48
41	51	12	102	0
153	156	48	55	279
199	232	53	160	105
59	110	22	128	175
67	66	0	182	39
0	20	0	33	40
551	831	228	928	969
0	121	21	76	0
12	40	0	36	0
34	125	10	48	0
93	10	0	0	26
0	0	0	0	0
0	0	0	0	0
0	0	0	28	0
232	318	29	441	90
0	24	0	10	0
201	73	20	146	102
0	0	0	0	0
81	183	28	237	0
0	83	0	57	0
142	406	59	375	0
0	15	0	15	0
0	10	0	24	10
43	86	14	141	0
0	0	0	10	28
0	0	0	0	0
0	12	0	31	0
0	0	0	0	0
0	0	0	0	0
30	25	0	46	0
0	0	0	0	0
0	0	0	0	0
13	119	14	293	0
119	164	14	175	374
10781	12351	2052	12896	2194
0	60	0	42	0
0	0	0	0	0
0	0	0	0	0
42	80	22	198	327
13	10	62	25	127
0	0	0	29	0
0	0	0	0	0

0	0	0	11	0
118	162	16	158	35
0	0	0	0	23
0	25	0	10	0
0	0	0	58	0
36	49	10	105	0
0	0	0	21	16
0	10	0	48	0
0	0	0	0	0
11	10	0	56	10
0	0	0	0	0
111	217	28	28	0
12195	8892	2483	3830	1908
1255	1134	159	484	535
0	119	0	74	0
0	0	0	0	0
0	0	0	0	0
0	30	0	0	0
642	284	83	48	41
3698	2603	463	1732	421
1473	1244	216	594	303
190	186	43	278	11
43	95	24	122	147
0	0	0	0	0
37	0	0	32	0
0	0	0	0	0
0	18	0	56	0
1044	1013	190	584	178
0	21	0	10	61
10	0	0	39	0
0	0	0	14	0
0	0	0	0	0
1506	1629	75	262	212
18	10	37	100	0
46	48	44	101	0
0	0	0	0	0
618	770	11	0	123
161	253	18	0	55
517	612	160	36	761
1178	1532	125	37	135
100	36	12	72	0
0	18	0	89	15
0	62	0	21	0
1632	1394	367	1867	754
91	74	0	46	0
0	0	0	36	0

0	0	0	0	0
22	0	0	24	0
0	0	0	0	0
198	142	0	290	0
626	362	28	498	128
724	472	87	793	507
684	399	32	308	59
25	68	0	211	14
139	177	21	246	425
0	34	0	141	0
31	17	13	28	0
0	86	0	33	0
0	15	0	0	0
0	0	0	0	0
0	0	0	0	0
0	0	0	0	0
0	0	0	30	0
25	93	0	43	0
0	0	0	0	0
54	60	101	24	0
647	616	12	373	58
0	0	0	0	0
4531	3808	487	988	427
18	17	0	15	0
3524	2794	240	1770	229
0	0	0	12	0
261	381	36	79	28
1271	931	70	783	34
0	0	0	0	0
0	0	0	33	0
0	0	0	0	0
985	0	0	0	4053
179	0	12	0	2694
87	51	0	0	0
67	70	20	34	109
0	23	0	0	0
55	62	0	38	138
0	0	0	10	0
27	0	0	93	0
24	12	0	0	182
12	36	24	232	72
346	281	19	378	506
1148	225	144	228	2376
76	31	34	0	0
236	82	38	0	262
0	0	58	22	18

0	150	0	0	13
715	1659	218	238	238
222	527	0	35	0
114	72	78	226	148
32	12	13	14	167
103	365	117	801	127
16	319	189	1879	916
91	126	0	155	36
15001	6972	2636	12586	7705
10085	9166	761	2459	516
5259	3731	205	1389	195
149	91	110	308	169
0	0	0	0	0
66	0	69	51	729
0	0	21	242	0
145	26	121	134	179
74	35	0	0	0
47	0	0	16	0
11	0	11	52	0
0	10	0	0	0
385	69	70	282	362
246	444	125	305	355
109	14	0	127	0
703	0	0	54	0
0	0	0	0	0
619	310	15	141	128
4440	7133	2822	4467	1475
2007	1550	468	2020	2145
14089	18082	8440	21198	35837
96	372	148	191	38
186	276	147	331	138
105	340	34	465	10
3253	2824	1195	3264	1803
1286	1318	523	2644	1215
171	248	70	94	11
0	63	0	71	37
2380	1852	1552	2898	6029
2418	1268	687	2405	5159
1672	885	1222	1559	2629
1220	1194	2434	4971	1623
0	49	89	459	13
0	0	10	77	22
2078	1922	5165	4369	11199
124	66	92	59	581
0	58	0	234	0
2008	1866	1437	1610	3495

747	630	100	162	1520
36490	27414	3183	9335	9240
23	60	0	108	0
1403	1034	313	721	451
268	557	132	43	1320
308	223	12	0	15
2009	2409	379	775	2597
1029	620	34	46	91
0	13	0	0	0
25	193	41	124	61
264	1582	279	447	1120
131	359	102	136	353
0	0	0	26	0
0	0	0	0	0
448	51	0	477	375
958	929	666	1985	968
236	273	359	474	1729
104	238	251	323	256
170	50	148	108	187
0	37	143	30	638
261	246	393	160	1583
0	0	0	0	0
0	0	0	0	0
77	30	0	90	0
552	303	127	10	208
0	0	12	0	0
0	0	0	0	0
0	0	0	0	0
10	0	0	0	0
143	0	10	328	163
163	0	0	23	0
38	0	0	0	0
942	14	118	110	74
1129	493	115	254	116
80	62	100	142	75
109	91	37	246	0
148	103	93	154	413
11	0	0	84	70
838	377	140	329	395
105	84	35	532	0
52	53	0	198	0
45	282	114	877	0
0	0	0	0	0
47	276	136	208	0
24	0	0	22	0
19	291	131	219	0

0	0	0	11	0
0	217	0	0	136
139	129	17	216	53
35	0	46	0	40
459	57	0	21	16
0	12	0	0	0
0	0	0	0	0
0	0	0	0	0
11	0	0	0	0
69	138	116	92	164
11	59	0	0	0
180	107	10	204	0
224	185	206	248	68
0	35	265	156	34
59	200	1637	308	399
121	79	51	144	36
0	36	154	51	26
213	265	105	102	55
255	41	13	57	26
103	0	12	0	0
341	1269	146	125	2234
39	172	74	31	217
56	30	0	0	140
0	0	0	0	0
198	129	21	10	57
36	55	0	11	0
0	0	0	0	0
129	62	10	94	0
0	175	0	260	0
100	0	0	16	0
0	0	0	0	11
0	0	0	0	0
0	37	0	0	0
0	12	0	0	108
11	42	0	0	12
0	11	0	0	0
0	0	0	0	0
0	0	0	0	0
0	0	0	0	0
47	0	0	0	0
0	0	0	0	0
72	0	0	0	0
0	0	0	0	0
14	32	30	32	0
30	124	0	0	0
81	55	0	80	15

56	0	0	37	0
20	12	0	0	0
10	0	0	32	0
97	73	10	11	282
10	39	0	0	0
0	0	0	0	0
72	169	0	205	47
0	0	0	16	0
14	28	0	40	0
27	70	0	21	386
10	37	0	0	70
0	0	0	0	0
84	47	67	82	170
50	0	0	60	0
40	10	0	84	0
0	34	0	31	0
67	36	0	37	0
29	63	0	12	0
63	291	109	177	3044
72	26	0	134	0
0	31	0	119	0
0	40	0	29	0
204	0	12	26	0
90	93	13	49	0
0	10	0	0	0
20	40	0	14	0
190	11	26	0	89
134	10	10	0	0
0	0	0	0	0
25	0	0	0	0
20	0	0	0	26
0	11	0	18	0
36	77	0	53	0
60	138	0	162	36
0	0	0	0	0
0	38	72	210	99
0	0	0	0	0
14	0	0	185	0
0	0	0	0	0
38	0	0	13	0
0	515	1279	676	35
0	86	347	133	0
12	146	549	60	35
0	44	0	0	39
0	0	0	12	329
10	0	0	0	0

0	10	20	90	41
0	0	0	35	32
212	10	0	0	0
0	0	0	12	0
10	0	62	266	1773
112	950	562	986	9304
0	0	0	11	0
11	61	454	227	232
0	42	50	28	10
0	79	0	514	111
12	0	0	22	0
22	17	0	20	0
1218	2454	121	514	1830
36	0	0	0	0
13	0	0	0	0
98	45	0	0	412
0	0	0	0	0
101	0	0	0	0
328	981	98	2860	4197
0	0	0	0	0
10	0	0	0	0
0	0	0	0	0
0	0	0	0	0
429	290	0	107	20
130	105	0	0	0
142	70	0	64	187
20	11	0	0	0
67	56	0	112	0
94	54	28	56	77
0	0	0	0	0
87	36	0	16	0
109	43	0	24	0
0	0	0	0	0
112	223	0	49	632
64	0	0	97	130
27	0	0	0	0
0	0	0	0	0
36	11	0	49	0
61	42	13	0	0
160	13	13	0	28
61	0	0	0	0
10	0	137	11	252
278	14	0	0	0
39	39	0	0	0
0	0	0	0	0
419	856	31	0	34

256	0	43	127	34
154	0	35	0	0
0	0	0	0	0
32	44	0	55	0
260	53	46	75	10
44	65	0	0	48
47	51	0	10	0
146	95	0	30	0
0	0	0	0	0
52	0	0	0	0
388	482	21	240	29
65	51	0	17	0
683	461	23	309	49
112	32	0	63	0
138	105	12	186	13
100	63	0	52	37
173	186	0	0	13
350	160	0	0	0
245	2069	0	149	15
219	0	0	0	0
547	0	0	0	0
59	75	0	50	0
27	175	0	31	0
141	262	0	172	0
255	76	0	47	10
174	101	29	12	0
531	210	161	128	143
141	56	29	83	73
553	200	105	69	150
53	0	0	0	0
0	13	0	0	212
27	77	12	0	75
11	19	0	12	70
27	138	0	12	115
0	0	0	40	36
39	32	0	0	0
0	0	0	0	96
225	140	17	124	41
205	64	16	46	0
122	0	0	0	33
228	321	63	258	27
205	51	32	21	0
1774	256	12	22	0
629	87	0	0	0
92	0	0	157	0
66	0	0	70	0

110	0	23	0	32
233	903	512	353	15
325	53	27	0	0
54	27	29	0	0
0	0	0	10	10
47	118	30	0	37
222	15	0	32	0
52	0	11	10	0
540	53	128	35	0
130	337	16	58	353
329	355	12	34	51
0	21	0	10	100
147	0	0	0	0
212	0	0	0	0
11	0	0	0	0
408	21	0	0	0
111	505	35	113	165
140	189	0	98	11
242	62	0	33	0
0	0	0	0	0
52	109	70	24	48
875	0	0	0	0
40	34	0	0	0
11	0	0	0	0
188	0	0	0	0
28	51	15	13	31
12	0	0	78	0
19	61	0	54	72
0	0	0	0	0
0	0	0	0	0
0	0	0	0	0
67	27	0	0	0
0	0	0	0	0
0	0	0	0	0
45	0	0	40	0
0	0	0	0	0
62	102	56	95	92
49	365	152	195	130
0	23	0	25	0
0	49	25	48	0
0	48	11	253	14
514	758	0	815	1453
14	0	0	0	0
0	0	0	0	0
13	51	0	0	83
346	19	12	125	164

0	0	0	0	0
0	0	0	0	0
33	115	56	52	0
0	57	0	72	142
85	0	0	88	123
0	0	0	0	36
23	0	0	14	0
61	188	0	39	215
0	0	0	0	0
49	91	15	0	178
57	130	0	142	292
49	21	0	0	0
38	59	0	0	0
37	10	0	0	10
97	49	0	0	0
23	34	0	0	0
0	0	0	43	0
0	0	0	0	0
12	15	0	0	0
34	63	0	12	27
0	0	0	11	0
0	0	0	61	0
0	14	0	0	0
0	0	0	0	0
0	0	0	0	0
0	0	0	0	0
44	32	0	36	35
32	34	0	32	0
39	26	0	10	0
0	0	0	0	0
75	45	0	0	16
91	46	0	16	13
25	38	0	12	0
0	0	0	0	0
499	528	606	149	648
0	24	259	75	48
12	0	0	0	0
0	0	27	0	0
267	529	169	64	180
22	0	0	23	22
10	17	0	0	0
107	25	0	0	0
106	0	0	34	0
0	0	0	0	0
179	15	0	92	86
45	0	0	23	0

337	0	0	106	14
69	0	0	0	0
0	0	0	0	0
13	0	0	13	0
50	87	0	12	10
97	86	15	0	10
0	11	0	0	0
72	88	0	13	0
43	51	0	0	0
36	12	0	0	0
10	12	0	0	0
0	0	0	0	0
42	53	0	0	0
80	58	0	0	0
47	71	0	0	0
35	34	0	47	0
47	73	0	39	0
23	0	0	0	0
344	0	0	0	0
189	0	0	0	0
64	0	0	0	0
0	0	0	0	0
46	0	0	0	0
75	53	0	10	134
35	126	14	63	361
146	85	0	10	281
0	12	0	0	22
39	56	0	0	68
51	0	0	0	0
259	0	0	49	0
56	0	0	0	0
45	51	0	93	0
115	0	0	0	0
0	0	0	0	0
11	0	0	0	112
34	11	0	41	0
93	0	0	110	0
10	56	0	96	0
0	64	0	121	0
0	0	0	0	0
277	282	452	142	1195
260	51	52	116	37
18	125	0	0	0
61	0	0	145	10
25	0	0	29	0
0	0	0	0	0

40	67	0	229	0
0	0	0	0	0
473	545	12	196	24
38	218	0	27	0
132	104	0	31	0
273	1075	0	62	15
40	0	0	0	0
89	90	0	0	0
569	370	11	75	36
12	0	0	47	0
45	0	74	609	0
10	0	61	164	0
0	0	0	268	108
112	0	0	90	10
174	148	73	24	139
57	134	0	0	501
0	130	10	43	99
123	73	14	42	2662
0	0	0	0	0
24	34	0	0	0
34	31	0	0	0
318	63	26	295	50
134	78	0	0	0
498	0	0	0	0
409	0	0	0	0
22	0	0	0	0
297	0	0	0	0
65	186	55	195	1052
0	35	0	48	0
33	42	0	0	0
337	150	39	100	66
140	147	91	53	0
313	67	32	0	89
410	0	0	0	0
2133	0	0	0	0
574	0	0	0	0
415	0	0	0	0
14	0	0	32	84
24	142	386	44	169
12	30	0	0	0
45	73	16	0	435
219	230	22	41	28
35	34	26	0	0
543	674	0	50	0
112	139	0	103	0
37	34	0	0	0

618	562	21	84	27
13	26	17	0	0
75	15	0	13	0
143	129	0	0	0
32	39	40	27	10
115	73	0	134	0
0	95	11	31	77
354	53	0	25	0
15	376	55	0	926
30	263	42	38	269
38	0	0	10	50
92	281	0	45	72
109	40	0	0	0
35	0	29	0	99
120	47	0	39	175
250	144	0	0	0
69	0	0	35	0
10	0	0	0	0
48	167	26	0	2531
0	24	0	87	0
0	99	22	35	0
71	35	0	0	187
12	57	0	0	171
11	28	0	40	0
0	16	18	101	0
43	0	11	0	0
0	0	0	25	0
21	0	0	0	0
51	0	0	28	0
0	31	0	0	0
0	12	0	0	0
48	102	0	0	0
72	329	91	121	0
12	280	0	0	0
0	0	0	0	0
45	118	59	268	92
12	20	0	27	10
0	22	0	0	0
0	0	0	0	0
42	0	0	27	0
14	0	0	0	0
0	0	0	0	0
38	0	0	0	0
0	0	0	0	0
13	11	0	0	36
0	10	17	10	0

14	60	0	11	52
0	0	0	0	12
0	11	0	0	163
32	414	0	0	79
0	147	92	449	372
0	0	10	0	0
0	58	0	0	48
0	92	0	0	0
57	68	0	0	0
44	77	0	0	0
0	10	0	0	0
25	91	0	304	0
0	33	0	0	0
25	43	11	0	35
48	27	0	0	181
0	0	10	0	0
0	35	0	10	60
0	0	0	0	99
0	0	0	14	0
44	53	12	29	64
0	48	0	0	0
13	15	0	14	0
17	31	11	0	186
0	42	12	13	121
0	0	53	0	29
10	65	72	0	3098
39	136	18	153	162
0	29	11	20	21
11	21	11	0	15
0	0	52	0	23
12	33	0	0	33
0	0	0	13	0
0	0	0	0	21
0	42	74	192	181
0	0	22	93	0
0	57	156	44	0
0	0	62	0	0
0	0	0	0	0
0	133	22	27	291
35	44	0	11	66
0	0	0	13	1390
0	12	0	0	0
0	0	11	0	0
0	0	0	0	0
0	0	37	116	0
19	0	0	0	10

23	0	16	24	383
0	34	861	0	146
0	0	0	0	27
0	0	23	11	221
0	14	64	0	356
0	0	39	0	0
36	27	0	29	0
12	244	0	0	0
43	90	0	32	27
169	312	0	91	38
0	0	0	0	0
0	0	0	0	34
13	29	0	13	0
0	0	0	0	0
0	0	0	0	0
0	0	0	0	0
0	0	0	23	0
0	234	0	84	50
36	87	0	695	821
52	0	26	159	1277
12	10	0	0	164
10	10	0	0	0
0	0	17	0	0
0	0	28	0	0
0	0	29	0	77
10	0	42	0	123
0	112	0	136	769
0	0	42	0	679
0	0	10	0	122
0	21	83	0	671
0	0	0	0	66
0	0	0	0	12
15	20	38	0	175
0	0	206	0	78
0	664	0	352	2196
40	27	22	0	200
0	31	0	10	182
0	0	0	0	0
0	0	0	0	0
0	0	0	0	0
0	0	0	0	0
0	0	0	0	0
0	0	0	0	0
0	0	0	0	0
0	0	0	0	0
16	0	0	0	0

0	0	0	0	0
0	0	0	0	0
0	0	0	0	0
0	0	0	0	0
0	0	0	0	0
0	0	0	0	0
0	0	0	0	0
0	0	0	0	0
0	0	0	0	0
0	0	0	0	0
0	0	0	0	0
0	0	0	177	0
0	0	0	0	0
0	0	51	107	298
0	0	0	0	182
13	0	0	0	0
0	0	0	0	0
0	0	0	0	0
0	0	0	0	0
0	0	0	0	0
0	0	0	0	0
0	0	0	0	0
32	42	0	0	0
0	0	0	0	0
0	0	0	0	0
0	0	0	0	0
0	0	0	0	0
0	0	0	0	0
0	0	0	0	0
22	0	0	12	0
0	0	0	0	0
0	0	0	0	0
0	0	0	0	0
0	0	0	0	0
0	0	0	0	0
0	0	0	0	0
0	0	0	0	0
0	0	0	0	0
0	0	0	0	0
0	0	0	0	0
0	0	0	0	0
0	0	0	0	0
0	0	0	0	0
0	0	0	0	0
0	0	0	0	0
0	0	0	0	0

72	0	0	0	0
27	0	0	0	0
0	0	0	0	0
0	0	0	0	0
0	0	0	0	0
0	0	0	0	0
0	0	0	0	0
0	0	0	0	0
0	42	0	0	0
0	0	0	0	0
0	0	0	0	0
0	0	0	0	0
178	29	0	0	0
0	0	0	0	0
0	0	0	0	0
0	0	0	0	0
0	0	59	0	0
0	0	0	0	0
0	0	0	0	0
0	0	0	0	0
0	0	0	0	0
0	0	0	0	0
0	0	0	0	0
0	0	0	0	0
0	0	0	0	0
0	0	0	0	0
0	0	0	0	0
0	0	0	0	0
0	0	0	0	0
0	0	108	23	22
0	0	0	0	0
0	0	0	0	0
0	0	0	0	0
0	0	0	0	0
0	0	0	0	0
0	0	0	0	0
0	0	0	0	0
0	0	0	0	0
0	0	0	0	0
31	0	0	0	0
0	0	0	0	0
0	0	0	0	0
0	0	0	0	0
0	11	0	0	0
0	38	0	0	18
0	12	0	0	183

0	51	0	0	54
0	58	0	10	80
25	47	21	26	83
67	43	11	11	0
26	13	0	0	0
34	12	0	0	0
35	10	0	0	0
28	42	0	13	0
27	10	0	0	0
0	10	0	0	0
0	23	0	0	0
36	34	0	0	14
0	33	0	0	0
10	11	0	0	0
12	13	0	44	0
0	10	0	0	0
0	16	0	0	0
0	42	0	0	100
0	14	0	0	67
26	73	0	0	67
14	10	0	0	38
0	11	0	0	0
0	11	0	0	0
0	10	0	30	0
0	38	13	52	0
11	12	0	12	0
0	15	0	0	0
0	12	0	0	0
0	24	0	0	0
0	30	0	14	0
0	46	178	127	21
0	19	0	0	0
56	90	0	0	179
0	12	0	0	0
0	16	0	23	0
0	10	0	0	0
13	50	0	0	0
0	25	0	0	0
98	15	14	12	0
0	13	0	79	0
14	17	0	0	0
0	11	0	0	0
0	21	0	25	0
12	15	0	0	0
0	16	0	0	0
0	54	0	0	0

0	43	0	0	0
49	15	41	108	10
46	17	0	21	0
10	10	0	47	0
0	22	0	0	222
35	13	169	0	10
0	22	194	0	0
0	10	34	0	0
10	17	0	0	0
0	31	0	0	0
10	91	0	0	0
0	104	0	22	0
23	39	0	22	0
0	110	0	0	0
28	52	0	31	0
10	11	0	13	0
43	57	0	0	0
0	42	0	0	0
24	25	0	0	0
107	50	0	13	0
0	13	0	0	0
125	10	0	0	0
0	10	0	0	0
0	15	0	0	0
0	10	0	0	0
10	48	0	0	0
0	10	0	0	0
0	11	0	0	0
0	21	0	0	0
17	10	0	0	0
0	20	0	0	0
0	32	0	0	0
0	12	0	31	0
0	115	0	904	524
0	24	0	0	0
0	10	0	0	0
0	10	0	0	0
0	59	0	172	12
0	12	0	0	0
0	13	0	10	0
0	26	0	0	15
85	12	0	27	292
0	60	0	151	31
0	21	0	15	0
0	10	0	0	0
0	71	29	0	0

14	23	0	0	0
30	10	0	49	11
0	10	102	0	39
0	26	11	0	18
0	10	0	0	0
10	15	0	0	0
25	20	0	0	0
13	34	0	10	0
0	11	0	0	0
0	16	0	0	23
0	12	0	0	0
0	12	0	0	0
26	10	0	0	0
0	31	0	0	0
0	11	0	0	0
44	21	0	0	0
21	44	0	0	0
0	11	0	0	36
0	28	0	0	0
0	71	25	0	314
58	12	0	11	0
0	17	0	0	0
0	33	0	26	57
0	14	12	53	0
0	21	0	0	0
0	16	0	0	30
0	57	0	0	0
0	13	0	0	0
0	46	0	0	173
0	35	0	0	0
0	45	0	0	0
0	42	11	81	0
19	13	0	0	0
0	13	52	237	541
0	34	47	37	235
0	67	63	116	309
0	67	0	399	10
0	65	115	533	1183
0	10	0	0	0
0	24	57	58	115
11	20	0	39	0
0	42	0	0	0
0	36	10	0	0
0	27	0	0	0
0	11	0	0	0
0	14	0	0	10

0	12	0	0	26
34	34	0	0	0
22	35	0	0	0
0	0	0	0	0
0	0	0	0	0
0	0	0	0	0
0	0	0	0	0
0	0	0	0	0
0	0	0	0	0
0	0	0	0	0
0	0	0	0	0
0	0	0	0	0
0	0	0	0	0
0	0	0	0	0
0	0	0	0	59
0	0	0	0	0
0	0	0	0	0
0	0	0	0	18
0	0	0	0	0
0	0	0	0	0
0	0	0	0	0
0	0	0	0	0
0	0	0	0	0
0	0	0	33	0
0	0	0	0	40
0	0	0	0	0
0	0	0	0	0
0	0	0	11	0
0	0	0	0	0
32	0	37	115	0
0	0	11	0	0
0	0	56	33	24
0	0	0	0	0
0	0	145	175	42
0	0	11	61	0
0	0	0	0	0
0	0	0	0	0
0	0	0	0	0
0	0	0	0	0
0	0	0	12	0
0	0	0	0	0
0	0	0	0	0
0	0	0	0	0
0	0	0	0	0
0	0	0	0	0
0	0	0	0	25

0	0	0	10	0
0	0	0	0	0
0	0	0	0	0
0	0	0	0	0
15	0	12	0	627
0	0	130	0	31
0	0	0	0	0
0	0	0	0	0
0	0	0	0	0
0	0	0	0	0
0	0	0	0	0
0	0	0	0	47
0	0	0	0	0
0	0	0	0	0
0	0	0	0	0
0	0	0	0	0
0	0	0	0	0
17	0	0	0	0
0	0	0	789	0
0	0	0	0	0
0	0	0	0	0
55	0	0	0	0
0	0	0	0	0
0	0	0	0	0
0	0	0	0	0
0	0	0	0	0
0	0	0	0	0
0	0	0	0	0
0	0	0	0	0
0	0	0	0	0
0	0	0	0	0
0	0	0	16	0
0	0	0	0	0
0	0	0	10	0
0	0	0	0	0
0	0	0	0	0
0	0	0	12	0
0	0	0	0	0
0	0	0	0	0
0	0	0	0	0
0	0	0	0	0
0	0	0	0	0
0	0	0	0	0
0	0	0	0	0
0	0	0	0	0
0	0	0	0	0

0	0	0	0	0
0	0	0	0	0
0	0	0	0	0
0	0	0	10	0
0	0	13	0	0
24	0	0	10	0
0	0	0	0	0
0	0	0	0	12
0	0	0	0	0
0	0	0	0	0
0	0	0	0	0
0	0	0	10	86
0	0	0	0	0
0	0	0	0	0
0	0	0	0	305
0	0	32	0	0
0	0	0	10	0
0	0	0	14	12
0	0	182	0	0
10	0	0	13	14
0	0	0	0	0
13	0	0	0	0
0	0	37	44	0
34	0	0	0	0
0	0	0	157	0
0	0	0	0	0
0	0	0	0	0
0	0	0	0	0
0	0	13	0	67
0	0	0	0	0
0	0	0	0	0
0	0	0	0	0
0	0	0	0	0
0	0	0	0	0
0	0	0	38	0
0	0	0	29	0
0	0	0	0	0
0	0	0	38	0
0	0	0	63	13
0	0	0	0	0
0	0	0	0	0
0	0	0	0	0
0	0	0	0	0
0	0	0	0	13
0	0	0	0	0
0	0	0	0	0

0	0	0	0	0
0	0	0	57	0
0	0	0	246	0
0	0	0	0	0
36	0	0	18	0
0	0	0	16	0
27	0	0	57	37
0	0	14	0	0
0	0	0	0	0
41	0	0	0	0
10	0	12	57	0
0	0	0	0	0
0	0	0	0	0
0	0	0	0	0
0	0	0	0	0
0	0	0	0	25
32	0	0	0	68
0	0	46	36	65
0	0	0	0	0
0	0	0	0	0
0	0	0	0	0
0	0	0	10	20
0	0	0	0	0
0	0	0	32	0
28	0	0	12	13
0	0	0	0	0
0	0	0	0	145
30	0	0	22	11
0	0	120	0	0
0	0	0	0	0
0	0	0	0	0
0	0	0	0	0
0	0	0	11	0
0	0	0	0	0
0	0	0	0	0
0	0	0	0	0
0	0	0	0	0
0	0	0	0	37
0	0	0	0	80
0	0	35	0	0
0	0	0	42	0
0	0	0	12	41
0	0	0	0	0
0	0	0	12	0
0	0	28	18	0
0	0	0	0	12

0	0	0	0	0
0	0	0	0	0
0	0	0	0	0
13	0	0	0	11
0	0	0	0	0
0	0	0	0	0
15	0	0	0	11
0	0	0	59	0
13	0	0	0	203
0	0	0	0	0
0	0	0	0	0
0	0	0	0	0
0	0	0	0	0
0	0	0	0	0
0	0	0	0	0
0	0	0	0	0
0	0	16	12	45
0	0	0	38	59
0	0	0	0	252
0	0	0	0	0
0	0	34	0	0
13	0	38	33	135
0	0	0	0	0
0	0	0	0	0
0	0	0	0	0
0	0	0	0	24
0	0	0	0	0
0	0	0	0	0
0	0	0	0	0
0	0	0	0	0
0	0	0	0	0
0	0	0	10	0
0	0	0	0	0
0	0	0	0	0
0	0	0	0	0
0	0	0	0	0
0	0	0	0	0
0	0	0	13	0
0	0	0	0	0
0	0	0	0	0
0	0	0	0	0
0	0	0	0	0
0	0	0	0	0
0	0	0	0	0
0	0	0	0	0
0	0	0	11	0

0	0	0	0	0
0	0	0	0	0
0	0	0	0	0
0	0	0	0	0
0	0	0	0	0
0	0	13	10	0
0	0	0	0	0
0	0	0	0	0
0	0	0	0	0
0	0	0	0	0
0	0	0	0	0
0	0	0	0	0
0	0	0	0	0
0	0	0	0	0
0	0	0	0	0
0	0	0	0	0
0	0	0	0	0
0	0	0	0	0
0	0	0	0	0
0	0	0	0	0
0	0	0	0	0
0	0	0	0	0
0	0	0	0	0
0	0	0	0	0
0	0	0	33	0
0	0	0	0	0
0	0	0	0	0
0	0	0	0	0
0	0	0	0	0
0	0	0	0	0
0	0	0	0	0
0	0	0	0	0
0	0	0	32	0
0	0	0	0	0
0	0	0	0	0
0	0	0	0	14
0	0	0	0	0
0	0	0	0	0
0	0	0	0	0
0	0	0	0	0
0	0	0	0	0
0	0	0	0	0
0	0	0	0	0
0	0	0	0	0
0	0	0	0	0
0	0	0	0	0

17	0	0	0	0
0	0	0	0	0
0	0	0	0	0
0	0	0	0	0
0	0	0	48	0
0	0	0	0	21
0	0	0	0	0
0	0	0	0	0
0	0	0	0	0
0	0	0	0	0
0	0	0	0	0
0	0	0	0	0
0	0	0	0	0
0	0	0	0	0
0	0	0	0	0
12	0	0	0	0
0	0	0	0	0
0	0	0	0	0
73	0	0	0	0
0	0	0	0	0
0	0	0	0	0
12	0	0	10	0
27	0	0	31	0
0	0	0	10	0
0	0	0	0	0
0	0	0	0	19
0	0	0	0	0
0	0	0	0	0
17	0	0	0	30
0	0	0	16	0
0	0	0	0	0
14	0	0	11	0
0	0	0	0	0
0	0	12	0	11
32	0	35	142	41
0	0	0	0	400
0	0	0	0	0
55	0	0	0	0
0	0	0	10	98
0	0	0	0	0
26	0	0	0	16
0	0	0	0	0
0	0	0	0	0
0	0	22	22	857
0	0	0	0	0
0	0	0	0	0

0	0	0	0	27
0	0	37	0	0
0	0	0	0	0
0	0	0	0	0
0	0	0	0	38
36	0	0	0	0
16	0	0	0	0
0	0	0	0	0
0	0	0	0	0
0	0	0	0	61
0	0	0	0	0
0	0	0	0	0
0	0	0	0	0
0	0	0	0	0
0	0	0	0	0
0	0	0	10	0
0	0	0	0	0
36	0	0	0	0
10	0	0	0	0
0	0	0	0	0
10	0	0	0	0
11	0	0	0	0
0	0	0	0	0
0	0	0	33	0
0	0	0	0	0
0	0	0	0	0
0	0	0	0	11
0	0	0	0	0
0	0	0	0	0
0	0	13	0	48
0	0	0	10	0
0	0	0	0	0
0	0	0	0	0
41	0	0	0	0
0	0	0	0	0
0	0	0	0	0
0	0	0	0	0
0	0	0	0	0
0	0	0	0	0
0	0	0	0	0
14	0	0	0	0
0	0	0	0	0
13	0	0	0	0
0	0	0	10	0
0	0	0	0	0
0	0	0	0	0

0	0	0	0	0
0	0	0	0	0
45	0	0	0	16
0	0	0	0	0
0	0	0	0	0
0	0	0	0	0
0	0	0	0	0
0	0	0	0	0
0	0	0	10	0
0	0	0	0	0
0	0	0	0	0
0	0	0	0	0
0	0	0	21	0
0	0	0	0	0
0	0	0	0	0
0	0	0	0	0
0	0	0	152	0
0	0	0	0	0
0	0	0	0	0
0	0	0	0	0
0	0	0	0	0
0	0	0	55	0
0	0	0	0	0
139	0	0	0	0
0	0	0	0	0
0	0	0	0	0
0	0	0	0	0
11	0	0	0	0
0	0	0	0	0
0	0	0	0	0
0	0	0	0	28
0	0	0	0	0
0	0	0	0	0
0	0	0	0	0
12	0	0	0	0
95	0	0	0	0
11	0	0	0	0
12	0	0	40	0
10	0	0	0	0
12	0	0	0	0
61	0	0	0	0
22	0	0	0	20
11	0	0	0	0
12	0	0	0	0
10	0	0	0	0
10	0	0	0	0

10	0	0	0	0
49	0	0	0	0
10	0	0	0	0
30	0	0	0	0
10	0	0	0	0
54	0	0	0	0
25	0	0	0	0
16	0	0	0	0
14	0	0	0	0
13	0	0	0	0
10	0	0	0	0
13	0	0	10	0
15	0	0	0	0
20	0	0	0	0
10	0	0	0	0
48	0	0	0	0
25	0	0	0	0
23	0	0	0	0
12	0	0	0	0
18	0	0	0	0
67	0	50	10	0
46	0	0	0	0
26	0	0	0	0
13	0	0	0	0
42	0	0	0	0
11	0	0	0	0
10	0	0	0	0
56	0	0	0	0
122	0	0	0	0
66	0	0	0	0
24	0	0	0	0
11	0	0	0	0
56	0	0	0	0
59	0	0	0	0
48	0	0	0	0
48	0	0	0	0
10	0	0	0	0
122	0	0	0	0
45	0	0	0	0
39	0	0	0	0
46	0	0	0	0
10	0	0	0	0
28	0	0	0	0
12	0	0	0	0
42	0	0	0	0
11	0	0	0	11

40	0	0	0	0
82	0	0	0	0
14	0	0	22	0
15	0	0	0	0
33	0	0	0	0
43	0	0	0	0
128	0	0	0	0
271	0	0	0	0
13	0	0	0	0
68	0	0	0	0
13	0	0	0	0
18	0	0	0	0
39	0	0	0	0
18	0	0	0	0
50	0	0	0	0
43	0	0	0	0
12	0	0	0	0
14	0	0	0	0
10	0	0	0	0
10	0	0	0	0
14	0	0	0	0
39	0	0	0	0
12	0	0	0	0
10	0	0	0	0
29	0	0	0	0
24	0	0	0	0
11	0	0	0	0
22	0	0	0	0
11	0	0	0	0
39	0	0	0	0
28	0	0	0	0
12	0	0	0	0
10	0	0	0	0
90	0	0	0	0
23	0	0	0	0
30	0	0	0	11
33	0	0	0	0
10	0	0	0	0
10	0	0	0	0
10	0	0	0	0
13	0	0	0	0
0	0	0	41	0
0	0	0	11	0
0	0	0	27	0
0	0	0	106	0
0	0	0	60	0

0	0	0	14	0
0	0	0	33	38
0	0	0	34	0
0	0	0	28	0
0	0	0	10	0
0	0	0	25	0
0	0	0	76	0
0	0	0	41	0
0	0	0	10	0
0	0	0	37	0
0	0	0	79	0
0	0	0	12	0
0	0	0	12	0
0	0	0	16	0
0	0	0	11	0
0	0	0	21	0
0	0	0	33	0
0	0	0	12	0
0	0	0	39	0
0	0	0	29	0
0	0	0	66	0
0	0	0	12	0
0	0	0	15	0
0	0	0	12	0
0	0	0	14	0
0	0	0	12	0
0	0	0	31	0
0	0	0	11	0
0	0	0	12	0
0	0	0	28	0
0	0	0	11	44
0	0	0	10	0
0	0	0	295	0
0	0	0	10	0
0	0	0	22	0
0	0	0	14	0
0	0	0	10	0
0	0	0	28	0
0	0	0	54	0
0	0	0	69	0
0	0	0	14	0
0	0	0	33	0
0	0	0	10	0
0	0	0	23	0
0	0	0	52	0
0	0	0	10	0

0	0	0	24	0
0	0	0	42	0
0	0	0	30	0
0	0	0	20	0
0	0	12	236	0
0	0	0	16	0
0	0	0	25	0
0	0	0	74	0
0	0	0	20	52
0	0	0	0	0
0	0	0	0	0
0	0	24	0	56
0	0	0	0	0
0	0	0	0	0
0	0	0	0	0
0	0	0	0	0
0	0	0	0	0
0	0	0	0	0
0	0	0	0	0
0	0	0	0	0
0	0	0	0	12
0	0	0	0	0
0	0	0	0	0
0	0	0	0	0
0	0	0	0	0
0	0	0	0	0
0	0	0	0	0
0	0	0	0	0
0	0	0	0	0
0	0	0	0	0
0	0	0	0	0
0	0	0	0	19
0	0	0	0	0
0	0	0	0	0
0	0	0	0	0
0	0	0	0	30
0	0	0	0	0
0	0	30	0	0
0	0	0	0	0
0	0	0	0	0
0	0	0	0	0
0	0	0	0	0
0	0	0	0	0
0	0	0	0	0
0	0	0	0	0
0	0	0	0	0

0	0	0	0	0
0	0	0	0	0
0	0	0	0	0
0	0	0	0	35
0	0	0	0	0
0	0	0	0	0
0	0	0	0	0
0	0	0	0	0
0	0	0	0	0
0	0	0	0	0
0	0	0	0	0
0	0	0	0	0
0	0	0	0	0
0	0	0	0	0
0	0	0	0	0
0	0	161	0	0
0	0	0	0	0
0	0	0	0	0
0	0	0	0	0
0	0	0	0	0
0	0	0	0	0
0	0	0	0	0
0	0	0	0	0
0	0	0	0	0
0	0	0	0	0
0	0	0	0	0
0	0	0	0	0
0	0	0	0	0
0	0	0	0	0
0	0	0	0	0
0	0	0	0	0
0	0	0	0	0
0	0	0	0	0
0	0	0	0	0
0	0	0	0	0
0	0	0	0	0
0	0	15	0	29
0	0	0	0	0
0	0	0	0	0
0	0	0	0	0
0	0	15	0	0
0	0	0	0	0
0	0	0	0	0
0	0	0	0	0
0	0	0	0	0
0	0	0	0	0

0	0	0	0	0
0	0	0	0	0
0	0	0	0	0
0	0	0	0	0
0	0	0	0	0
0	0	0	0	0
0	0	0	0	0
0	0	40	0	0
0	0	0	0	0
0	0	0	0	0
0	0	0	0	0
0	0	0	0	0
0	0	0	0	0
0	0	0	0	0
0	0	0	0	0
0	0	0	0	0
0	0	0	0	0
0	0	0	0	0
0	0	0	0	0
0	0	0	0	0
0	0	0	0	0
0	0	0	0	0
0	0	0	0	0
0	0	0	0	0
0	0	0	0	0
0	0	0	0	0
0	0	0	0	0
0	0	0	0	0
0	0	0	0	0
0	0	0	0	22
0	0	0	0	49
0	0	0	0	10
0	0	0	0	0
0	0	0	0	132
0	0	0	0	0
0	0	0	0	10
0	0	0	0	36
0	0	0	0	0
0	0	0	0	0
0	0	0	0	0
0	0	0	0	0
0	0	0	0	0
0	0	0	0	0
0	0	0	0	0
0	0	0	0	0
0	0	0	0	0

0	0	0	0	0
0	0	0	0	0
0	0	0	0	0
0	0	0	0	0
0	0	0	0	0
0	0	0	0	13
0	0	0	0	0
0	0	0	0	0
0	0	0	0	0
0	0	0	0	0
0	0	0	0	0
0	0	0	0	0
0	0	0	0	0
0	0	0	0	0
0	0	0	0	0
0	0	0	0	0
0	0	0	0	0
0	0	0	0	0
0	0	0	0	0
0	0	0	0	0
0	0	0	0	0
0	0	0	0	0
0	0	0	0	0
0	0	0	0	0
0	0	0	0	0
0	0	0	0	0
0	0	0	0	0
0	0	0	0	0
0	0	0	0	0
0	0	0	0	0
0	0	20	0	11
0	0	0	0	0
0	0	0	0	0
0	0	0	0	0
0	0	15	0	0
0	0	10	0	0
0	0	39	0	0
0	0	16	0	0
0	0	11	0	0
0	0	51	0	0
0	0	11	0	21
0	0	24	0	0
0	0	126	0	0
0	0	16	0	0
0	0	11	0	0
0	0	0	0	0

0	0	0	0	0
0	0	0	0	0
0	0	0	0	0
0	0	0	0	0
0	0	0	0	0
0	0	0	0	0
0	0	0	0	0
0	0	0	0	0
0	0	0	0	0
0	0	0	0	0
0	0	0	0	0
0	0	0	0	0
0	0	0	0	0
0	0	0	0	0
0	0	0	0	0
0	0	0	0	0
0	0	0	0	0
0	0	0	0	0
0	0	0	0	0
0	0	0	0	0
0	0	0	0	0
0	0	0	0	0
0	0	0	0	54
0	0	0	0	0
0	0	0	0	0
0	0	0	0	0
0	0	0	0	0
0	0	0	0	0
0	0	0	0	0
0	0	0	0	0
0	0	0	0	0
0	0	0	0	0
0	0	0	0	0
0	0	0	0	0
0	0	0	0	0
0	0	0	0	0
0	0	0	0	0
0	0	0	0	0
0	0	0	0	0
0	0	0	0	0
0	0	0	0	0
0	0	0	0	0
0	0	0	0	0
0	0	0	0	0
0	0	0	0	0
0	0	0	0	0

0	0	0	0	0
0	0	0	0	0
0	0	0	0	0
0	0	0	0	0
0	0	0	0	0
0	0	0	0	0
0	0	0	0	0
0	0	0	0	0
0	0	0	0	0
0	0	0	0	0
0	0	0	0	0
0	0	0	0	0
0	0	0	0	0
0	0	0	0	0
0	0	0	0	0
0	0	0	0	0
0	0	0	0	0
0	0	0	0	0
0	0	0	0	0
0	0	0	0	0
0	0	0	0	0
0	0	0	0	0
0	0	0	0	0
0	0	0	0	0
0	0	0	0	0
0	0	0	0	0
0	0	0	0	14
0	0	0	0	0
0	0	0	0	39
0	0	0	0	0
0	0	0	0	0
0	0	0	0	0
0	0	0	0	0
0	0	0	0	0
0	0	0	0	23
0	0	0	0	0
0	0	0	0	0
0	0	0	0	0
0	0	0	0	0
0	0	0	0	0
0	0	0	0	0
0	0	0	0	0
0	0	0	0	0
0	0	0	0	0
0	0	0	0	0
0	0	0	0	0

0	0	0	0	0
0	0	0	0	0
0	0	0	0	0
0	0	0	0	0
0	0	0	0	0
0	0	0	0	0
0	0	0	0	0
0	0	0	0	0
0	0	0	0	0
0	0	0	0	0
0	0	0	0	0
0	0	0	0	0
0	0	0	0	0
0	0	0	0	0
0	0	0	0	0
0	0	0	0	0
0	0	0	0	0
0	0	0	0	0
0	0	0	0	0
0	0	0	0	0
0	0	0	0	16
0	0	0	0	0
0	0	0	0	0
0	0	0	0	0
0	0	0	0	0
0	0	0	0	10
0	0	0	0	24
0	0	0	0	0
0	0	0	0	0
0	0	0	0	0
0	0	0	0	0
0	0	0	0	0
0	0	0	0	0
0	0	0	0	0
0	0	0	0	0
0	0	0	0	0
0	0	0	0	0
0	0	0	0	61
0	0	0	0	11
0	0	0	0	0
0	0	0	0	12
0	0	0	0	0
0	0	0	0	0
0	0	0	0	0
0	0	0	0	0
0	0	0	0	0

0	0	0	0	0
0	0	0	0	0
0	0	0	0	0
0	0	0	0	0
0	0	0	0	0
0	0	0	0	0
0	0	0	0	0
0	0	0	0	0
0	0	0	0	0
0	0	0	0	0
0	0	0	0	0
0	0	0	0	0
0	0	0	0	0
0	0	0	0	0
0	0	0	0	0
0	0	0	0	0
0	0	0	0	0
0	0	0	0	0
0	0	0	0	0
0	0	0	0	0
0	0	0	0	0
0	0	0	0	0
0	0	0	0	0
0	0	0	0	0
0	0	0	0	0
0	0	0	0	0
0	0	0	0	0
0	0	0	0	0
0	0	0	0	0
0	0	0	0	0
0	0	0	0	0
0	0	0	0	0
0	0	0	0	0
0	0	0	0	0
0	0	0	0	0
0	0	0	0	0
0	0	0	0	0
0	0	0	0	0
0	0	0	0	0
0	0	0	0	0
0	0	0	0	0
0	0	0	0	0
0	0	0	0	0
0	0	0	0	0
0	0	0	0	0
0	0	0	0	0

0	0	0	0	0
0	0	0	0	0
0	0	0	0	0
0	0	0	0	0
0	0	0	0	0
0	0	0	0	0
0	0	0	0	0
0	0	0	0	0
0	0	0	0	0
0	0	0	0	0
0	0	0	0	0
0	0	0	0	0
0	0	0	0	15
0	0	0	0	53
0	0	0	0	22
0	0	0	0	13
0	0	0	0	16
0	0	0	0	71
0	0	0	0	23
0	0	0	0	27
0	0	0	0	10
0	0	0	0	50
0	0	0	0	20
0	0	0	0	33
0	0	0	0	47
0	0	0	0	30
0	0	0	0	12
0	0	0	0	17
0	0	0	0	11
0	0	0	0	11
0	0	0	0	18
0	0	0	0	44
0	0	0	0	19
0	0	0	0	0
0	0	0	0	0
0	0	0	0	0
0	0	0	0	0
0	0	0	0	0
0	0	0	0	0
0	0	0	0	0
0	0	0	0	0
0	0	0	0	0
0	0	0	0	0
0	0	0	0	0
0	0	0	0	0
0	0	0	0	0

0	0	0	0	0
0	0	0	0	0
0	0	0	0	0
0	0	0	0	0
0	0	0	0	0
0	0	0	0	0
0	0	0	0	0
0	0	0	0	0
0	0	0	0	0
0	0	0	0	0
0	0	0	0	0
0	0	0	0	0
0	0	0	0	0
0	0	0	0	0
0	0	0	0	0
0	0	0	0	0
0	0	0	0	0
0	0	0	0	0
0	0	0	0	0
0	0	0	0	0
0	0	0	0	0
0	0	0	0	0
0	0	0	0	0

Phylum	Genus	CS_242	CS_249	CS_273	CS_280
Proteobacteria	Acinetobacter	1122	1164	1396	822
Actinobacteria	Brachybacterium	0	0	0	0
Bacteroidetes	Chryseobacterium	805	709	207	103
Proteobacteria	Comamonas	0	0	0	0
Actinobacteria	Corynebacterium	1730	86	420	403
Proteobacteria	Devosia	118	852	361	1598
Bacteroidetes	Flavobacterium	1157	907	390	341
Firmicutes	Kurthia	0	0	0	0
Proteobacteria	Luteimonas	263	693	177	7047
Proteobacteria	Paracoccus	266	876	293	282
Firmicutes	Planomicrobium	0	0	0	0
Proteobacteria	Pseudomonas	1200	1114	403	1692
Actinobacteria	Saccharopolyspora	4223	329	1215	42
Proteobacteria	Sphingomonas	1663	4232	854	1136
Proteobacteria	Stenotrophomonas	0	0	0	0

Table S2: Top 10 abundant genera in each placental region.

CS_282	CS_283	CS_288	CS_302	CS_307	CS_324	CS_329
436	487	0	2204	4098	315	27
0	0	0	0	0	0	0
2238	3707	12	417	4345	76	274
0	0	0	0	0	0	0
836	1303	0	451	2615	550	134
4505	4149	0	1521	1071	0	51
1424	2998	0	880	2942	0	59
0	0	0	0	0	0	0
2484	3675	38	273	1224	227	253
6357	4596	10	15	6917	29	96
0	0	0	0	0	0	0
593	2204	0	509	444	118	134
5194	284	10	766	338	11	0
5892	5722	0	1539	3322	132	314
0	0	0	0	0	0	0

CS_332	CS_333	CS_340	CS_341	CS_348	PH_242	PH_249
78	1202	2915	74	286	0	0
0	0	0	0	0	0	0
259	614	1631	168	103	38	345
0	0	0	0	0	70	577
78	1258	7693	615	2679	169	918
0	284	1349	250	911	0	717
51	843	1524	241	1254	166	225
0	0	0	0	0	0	0
32	668	1261	460	2418	12	576
44	156	2294	270	314	15	887
0	0	0	0	0	180	46
105	3125	2714	268	1171	59	412
199	136	0	131	7378	0	0
136	4158	2321	463	1094	333	1543
0	0	0	0	0	0	0

PH_273	PH_280	PH_282	PH_283	PH_302	PH_307	PH_324
0	0	0	0	0	0	0
0	0	0	0	0	0	0
181	42	1677	1782	365	1593	53
372	465	164	133	1963	966	1124
863	393	1897	359	729	2111	2470
180	692	4559	5768	200	464	15
152	278	560	2137	1025	1669	440
0	0	0	0	0	0	0
29	773	2639	2532	221	914	188
23	69	4254	4245	128	1660	88
0	25	135	73	874	9258	43
10	630	287	252	324	530	298
0	0	0	0	0	0	0
354	505	6330	4800	985	2785	383
0	0	0	0	0	0	0

PH_329	PH_331	PH_332	PH_333	PH_340	PH_348	UC_242
0	0	0	0	0	0	670
0	0	0	0	0	0	229
3485	64	0	615	202	0	484
1455	98	35	2001	1486	588	0
1553	33	45	2043	1435	1134	474
2112	0	0	342	138	237	176
3907	144	0	1591	262	145	0
0	0	0	0	0	0	1687
1735	39	29	325	191	1646	111
4213	20	23	0	158	96	201
1444	41	345	115	140	30	0
2754	52	49	2632	1686	1599	314
0	0	0	0	0	0	0
3491	157	32	3264	963	365	0
0	0	0	0	0	0	476

UC_249	UC_261	UC_273	UC_280	UC_282	UC_283	UC_288
448	653	1804	306	793	576	82
213	445	0	360	65	319	22
141	561	375	4335	63	9739	84
0	0	0	0	0	0	0
186	830	605	128	154	186	89
110	125	579	3780	46	6358	21
0	0	0	0	0	0	0
306	477	225	2687	0	345	369
259	233	87	1198	307	2371	142
76	163	49	324	176	6869	71
0	0	0	0	0	0	0
144	127	617	1346	29	104	197
0	0	0	0	0	0	0
0	0	0	0	0	0	0
447	306	535	7281	113	2624	451

UC_302	UC_307	UC_324	UC_329	UC_332	UC_333	UC_340
2082	990	1807	5183	1319	1133	1796
430	3913	2610	2210	1232	916	1662
316	1646	122	641	15	593	1137
0	0	0	0	0	0	0
505	1916	2344	2371	489	1240	2939
241	492	69	2190	181	436	1030
0	0	0	0	0	0	0
1350	1236	544	1746	1461	2374	925
152	878	395	4373	928	831	864
82	1209	233	3289	430	167	1123
0	0	0	0	0	0	0
124	359	195	2908	717	3863	3954
0	0	0	0	0	0	0
0	0	0	0	0	0	0
1073	2058	623	3162	678	2472	1263

UC_341	UC_342	UC_348
626	1477	1994
2657	862	1665
163	1095	35
0	0	0
689	1366	5367
535	1810	2267
0	0	0
574	1025	632
1405	1280	4830
1191	887	988
0	0	0
438	1295	3417
0	0	0
0	0	0
520	1939	1069
